# Electrochemical One-Step Synthesis of Alkyne Sulfonates and Sulfonamides: Building Blocks for Highly Substituted Alkenes and 1,3-Butadienes

**DOI:** 10.1021/jacsau.5c00972

**Published:** 2025-10-23

**Authors:** Meysam Azizzade, Florian A. Breitschaft, Siegfried R. Waldvogel, Till Opatz

**Affiliations:** † Department of Chemistry, 9182 Johannes Gutenberg-University Mainz, 55128 Mainz, Germany; ‡ Department of Electrosynthesis, 28313 Max-Planck-Institute for Chemical Energy Conversion, Stiftstraße 34−36, 45470 Mülheim an der Ruhr, Germany; § Karlsruhe Institute of Technology, Institute of Biological and Chemical Systems − Functional Molecular Systems (IBCS-FMS), Kaiserstraße 12, 76131 Karlsruhe, Germany

**Keywords:** alkynes, sulfonates, sulfur dioxide, electrochemistry, copper catalysis

## Abstract

Alkyne motifs have a central place in organic synthesis due to their versatility as building blocks for complex molecule construction. The development of efficient and sustainable strategies for the synthesis of functionalized alkynes remains an utmost challenge in modern organic chemistry. Herein, we report a novel and efficient electrochemical multicomponent reaction (eMCR) that enables the one-step synthesis of valuable alkyne sulfonates and sulfonamides using SO_2_ stock solutions and electric current as a clean oxidant. The generated alkynyl sulfonates and sulfonamides serve as powerful synthetic intermediates, undergoing diverse downstream transformations, including regio- and stereoselective copper-catalyzed dimerizations, hydroarylations, and cyclizations, to access 1,3-dienes, highly substituted alkenes, and heterocycles. Moreover, this method enables late-stage functionalization of complex molecules and pharmaceuticals, demonstrating its significant potential for natural product synthesis, medicinal chemistry, and materials science. The study underscores the promise of electrochemical MCRs as sustainable and versatile tools in modern synthetic methodology.

## Graphical abstract

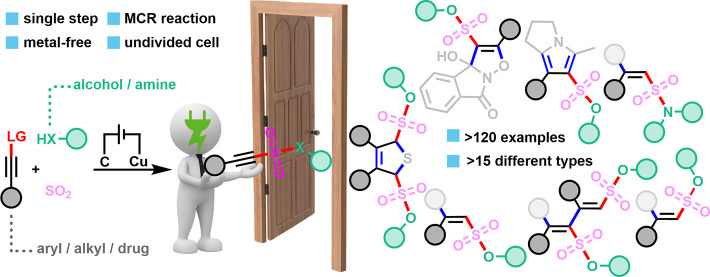

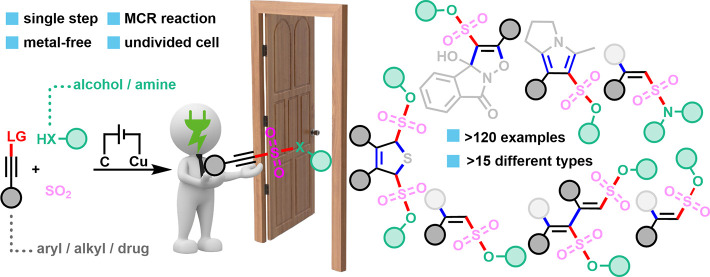

Alkynes serve as versatile building blocks in organic chemistry and therefore play a crucial role in the construction of complex molecules.^1^ Their synthetic utility has resulted in countless methods, providing facile access to a wider array of alkynyl structures. Besides the direct formation of triple bonds through eliminations or methodologies like the Corey-Fuchs reaction^2^ and the Seyferth-Gilbert homologation,^3^ modifying pre-existing triple bonds is a common approach.^4^ Nevertheless, many procedures for the synthesis of alkynyl motifs from simple and accessible alkynes heavily depend on the use of aggressive organometallic reagents,^5^ noble metal catalysts,^6^ iodine,^7^ or harsh reaction conditions. The metal-free synthesis of new functionalized alkynes is quite challenging and thus, developing new methods to generate diverse arrays has remained a topic of interest in the last few decades.^8^ Remarkable advances have been made in the realm of organic electrosynthesis recently, a metal-free approach, propelled by the facilitation of redox chemistry without external oxidants or reducing agents.^9^ Although a few protocols have been developed for the synthesis of new alkynyl motifs from simpler alkynes under electrochemical conditions,^10^ metal-free, multicomponent reactions for this purpose are both still underexplored and desirable. In this scenario, multicomponent reactions (MCRs), which can generate multiple carbon–carbon or carbon-heteroatom bonds from simple starting materials in a single synthetic operation, represent an attractive strategy in organic synthesis and often allow to enhance reaction efficiency and productivity.^11^ In this regard, Lei’s group very recently reported electrochemical MCRs for the functionalization of alkynes through a Sonogashira-type reaction using palladium catalysts (Scheme 1A).^12^ As part of our efforts to valorize SO_2_, recently gaining significant attention in electrochemical syntheses (Scheme 1B),^13^ traditionally considered a waste product, and in view of the importance of sulfonates and sulfonamides in areas like the materials sciences,^14^ agrochemistry^15^ (serving e.g., as herbicides,^16^ insecticides,^17^ antifungals,^18^ or agents against plant pathogenic bacteria and viruses^19^), and pharmaceuticals,^20^ we sought for a one-step synthesis of alkyne sulfonates and alkyne sulfonamides, which are conventionally accessed via tedious and harsh thionyl chloride chemistry (Scheme 1C). Here, we present a novel and versatile MCR electrooxidation method for the generation of alkynyl sulfonates and sulfonamides (Scheme 1D). These compounds proved to be useful building blocks.

**Scheme 1:**
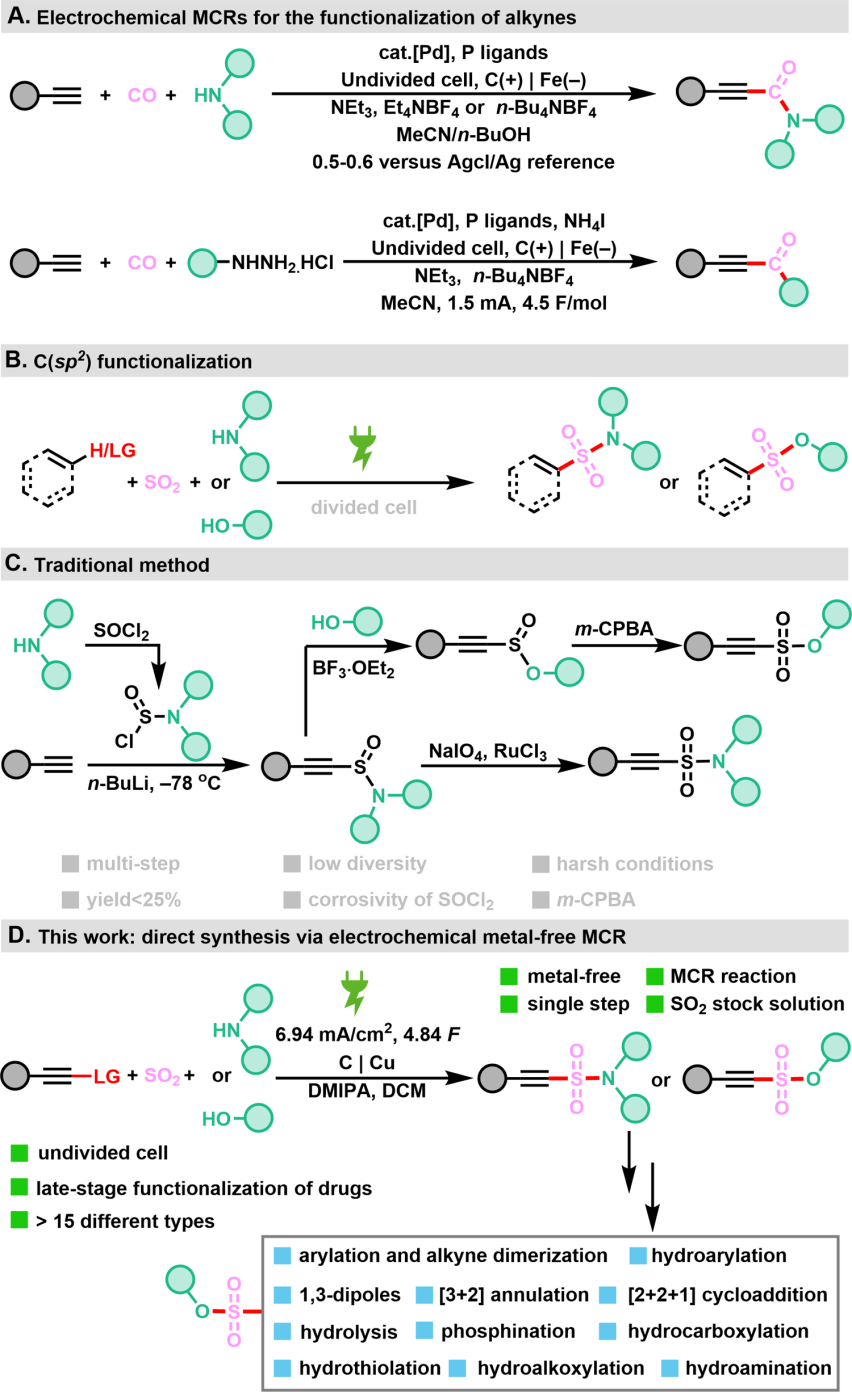
Synthesis of Alkyne Derivatives; (A) Electrochemical MCRs for Alkyne Functionalization; (B) C­(sp^2^) Functionalization; (C) Traditional Synthesis Strategy; (D) Present Work

## Results and Discussion

Our study began with the investigation of the electrochemical decarboxylative coupling between 3-phenylpropiolic acid **1a**, SO_2_ stock solution (5 M in acetonitrile), and neopentyl alcohol **2h** (*N*,*N*-diisopropylethylamine (DIPEA) as base, Bu_4_NBF_4_ as electrolyte, constant current (10 mA, 4.63 mA/cm^2^, 2.16 cm^2^), graphite (C) anode and platinum plate cathode, room temperature, undivided cell), see Table S1. Different solvents were examined (Table S1), showing dichloromethane (DCM) to be optimal (Table 1, entry 1), Since the stock solution can be conveniently prepared by simply bubbling SO_2_ gas into the solvent, we also explored various SO_2_ solutions and found that the reaction could generate a yield up to 18% when solution in DMSO was used (entry 2). The change of the current from 10 mA (4.63 mA/cm^2^, 2.16 cm^2^) to 15 mA (6.94 mA/cm^2^, 2.16 cm^2^) had a significant effect on the yield (entries 4,6). After an extensive evaluation of the reaction conditions (Table S3), it turned out that using DBU as a base, along with its role in the formation of a Lewis adduct^21^ with SO_2_, provided the highest efficiency compared to other amines (entry 7). However, poor reproducibility was encountered when DBU, DBN, 1,2-dimethyl-1,4,5,6-tetrahydropyrimidine, or 1,1,3,3-tetramethylguanidine were used (Table S3), most likely due to side reactions of the desired product (Table 1B). In contrast, the use of *N*,*N*-dimethylisopropylamine (DMIPA) provided a good and reproducible yield of 59% (entry 10). A systematic investigation of different supporting electrolytes, including control experiments performed in their absence (entry 11), revealed that the yield improved when no additional supporting electrolyte was used (71%). The replacement of graphite with other anode materials (glassy carbon (GC) and reticulated vitreous carbon (RVC)) showed a detrimental effect on the formation of **3ah** (entry 12), but gratifyingly, Ni, Cu, and stainless steel also proved to be suitable cathode materials for the reaction (entry 13).^22^ The desired product was also successfully synthesized using AA batteries as the power source (entry 15, 54%), showcasing the robustness and simplicity of our method. In addition, control experiments indicated that open-air conditions are crucial to the reaction. After systematically screening various conditions (see Supporting Information for details), the desired product **3ah** could be obtained in 71% yield through constant current electrolysis at 15 mA (6.94 mA/cm^2^), in the presence of 3.2 equiv of DMIPA in DCM at r.t. for 315 min (*Q*_applied_ = 280 C, *Q*_Faraday_ = 4.84 F) under air (entry 14).

**Table 1:**
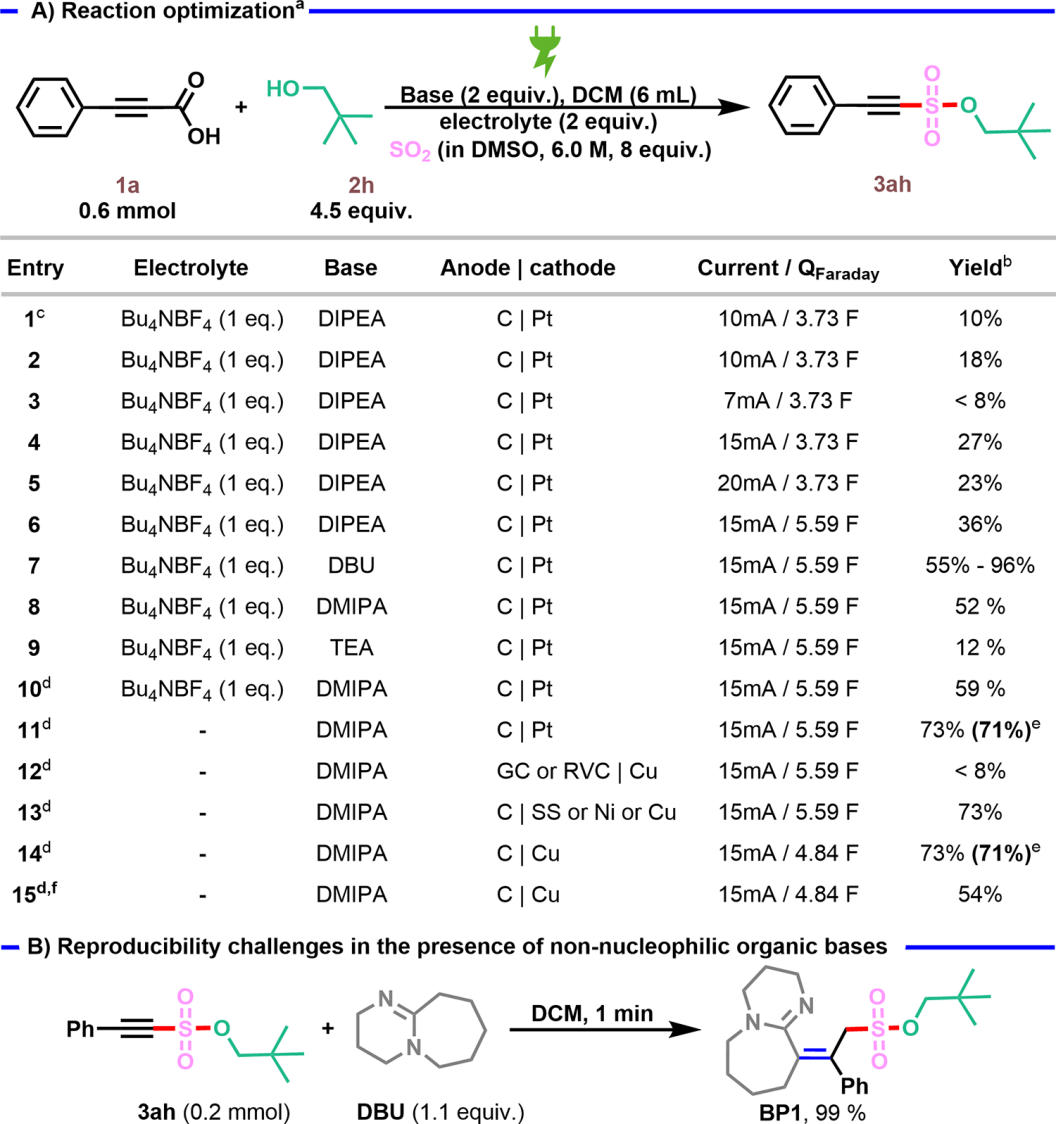
Reaction Optimization

a Reaction conditions: **1a** (0.6 mmol), **2h** (4.5 equiv), SO_2_ in DMSO (0.8 mL, 6.0 M, 8 equiv), base (2 equiv), and DCM (6 mL) in an undivided cell.

b NMR yield.

c SO_2_ in MeCN.

d DMIPA (3.2 equiv).

e Isolated yield.

f 4 × AA batteries as the power source.

### Reaction Scope of the Electrochemical Synthesis of Alkyne Sulfonates and Sulfonamides

We evaluated the scope of this electrochemical MCR first using various alcohols (Scheme 2). Generally, a wide range of primary alcohols bearing different functional groups, such as ether (**3al**, **3am**, **3an**), phenyl (**3ap**, **3aq**), halide (**3aj**, **3ak**, **3an**), and cyclic acetal (**3ao**), were all found to be compatible. Moreover, primary alcohols of different chain lengths and steric hindrance (**3aa**–**3ai**) were smoothly converted to the corresponding alkenyl sulfonate products in 38–74% yields. Secondary alcohols gave slightly lower yields due to their increased hindrance (**3ar**–**3aza**). The natural products borneol, menthol, and coprostanol were also amenable to this two-bond-forming strategy, producing 59%(**3ay**), 54% (**3ay**), and 27% (**3aza**) yield. Gratifyingly, this electrosynthesis was also applicable to amines, enabling the one-pot generation of alkenyl sulfonamides. We explored its versatility with a range of cyclic and acyclic secondary amines, yielding products **3azb** to **3azg** in isolated yields of 29 to 57%. Following the successful development of a decarboxylative MCR on propiolic acid derivatives, alkynyl bromides were also evaluated as coupling partners. Gratifyingly, the reaction exhibited very good compatibility with various functional groups in the bromoalkyne. Aliphatic alkynes with different chain lengths are also suitable substrates for this reaction (**3ka**, **3la**). A broad array of synthetic handles showed good functional group robustness, including halide (**3la**), nitrile (**3ma**), ether (**3pa**, **3ra**), ester (**3na**, **3qa**, **3ra**, **3sa**), sulfonate ester­(**3oa**), and ketone (**3ra**). Promisingly, substrates based on the anti-inflammatory drugs isoxepac (**3ra**) and α-tocopherol (**3sa**) proved to be well tolerated. In the reaction of 1,4-bis­(bromoethynyl)­benzene, a monoaddition product was isolated (**3ta**) in 41% yield, and the unreacted bromoalkyne unit can subsequently be subjected to coupling reactions. Importantly, compound **3ta** also enables the construction of symmetrical or unsymmetrical bis­(alkynyl) sulfonates (**3ua**). We also attempted to synthesize target compound **3ah** from phenylacetylene; however, the yield did not exceed 35% (Table S4).

**Scheme 2:**
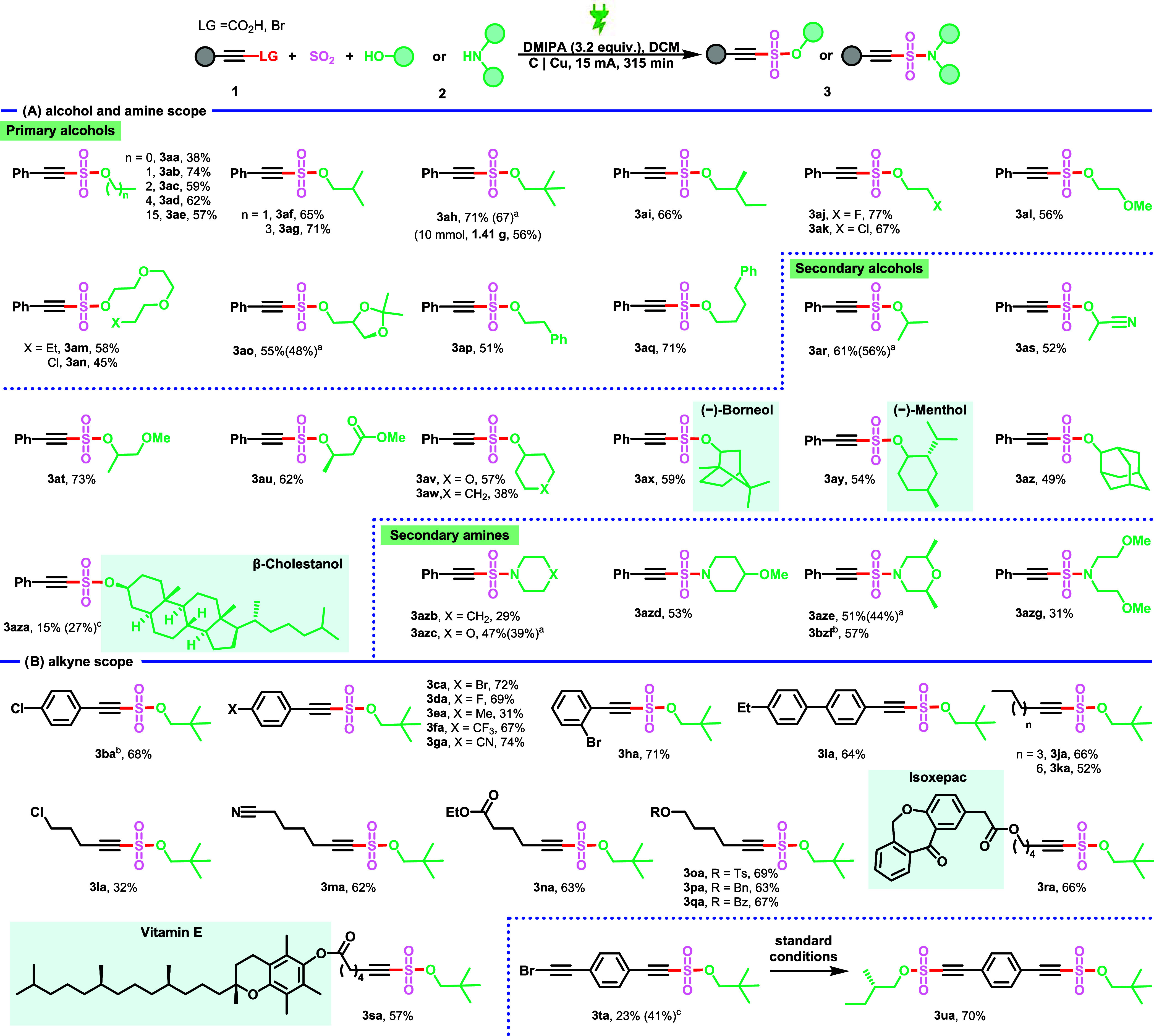
Scope of the Electrochemical Synthesis of Alkyne Sulfonates and Sulfonamides; (A) Alcohol and Amine Scope; (B) Alkyne Scope^d^

a with (bromoethynyl)­benzene.
b with 3-(4-chlorophenyl)­propiolic acid.
c 7 h (*Q*_Faraday_ = 6.45 F).
d Reaction conditions: **1** (0.6 mmol), **2** (4.5 equiv), SO_2_ in DMSO (0.8 mL, 6.0 M, 8 equiv), DMIPA (3.2 equiv), and DCM (6 mL) in an undivided cell with graphite (C) anode and copper plate cathode (each 2.7 cm × 0.8 cm), room temperature, 15 mA, 315 min (*Q*_applied_ = 280 C, *Q*_Faraday_ = 4.84 F).

### Investigation of the Reaction Mechanism

After developing a general method for the eMCR assembly of alkyne sulfonates and sulfonamides, subsequent efforts were directed toward exploring the reaction mechanism (Scheme 3). Cyclic voltammetry (CV) measurements showed that the oxidation potentials increase in the following order: intermediate *O*-alkyl sulfite, 3-phenylpropiolate, and alkyne sulfonate **3ah**. Notably, **3ah** is not prone to overoxidation due to its significantly higher oxidation potential compared to the intermediate *O*-alkyl sulfite (Scheme 3B). Evidence for the involvement of radical species in the reaction mechanism was obtained through trapping experiments employing an excess of 2,6-di-*tert*-butyl-4-methylphenol (BHT) (Scheme 3C). Under these conditions, the formation of **3aa** was not observed; instead, **3va** was isolated in 37% yield, further confirming the presence of **Int II** as a key intermediate. Control experiments revealed that both the electric charge and base are essential for the reaction (Scheme 3D). The radical pathway is evidenced by the behavior of radical probe substrate **3wa** (alkenyl radical addition), as shown in Scheme 3D. Based on these results and previous literature reports, we propose the following mechanism (Scheme 3A). As shown in Scheme 3A, sulfur dioxide and the alcohol form Lewis acid–base adducts, generating the intermediate *O*-alkyl sulfite (**Int I**) via deprotonation by DMIPA. Initial anodic oxidation of **Int I** produces the radical **Int II**. This radical then attacks either the acetylenic acid anion or the bromoalkyne, giving rise to radical intermediates **Int III** or **Int IV**. **Int III** undergoes further oxidation, leading to decarboxylation and formation of the desired product **3**, whereas **Int IV** may undergo cathodic reduction to yield **3**. As the cathodic reaction, most likely the SO_2_ reduction or hydrogen evolution process occurs.

**Scheme 3:**
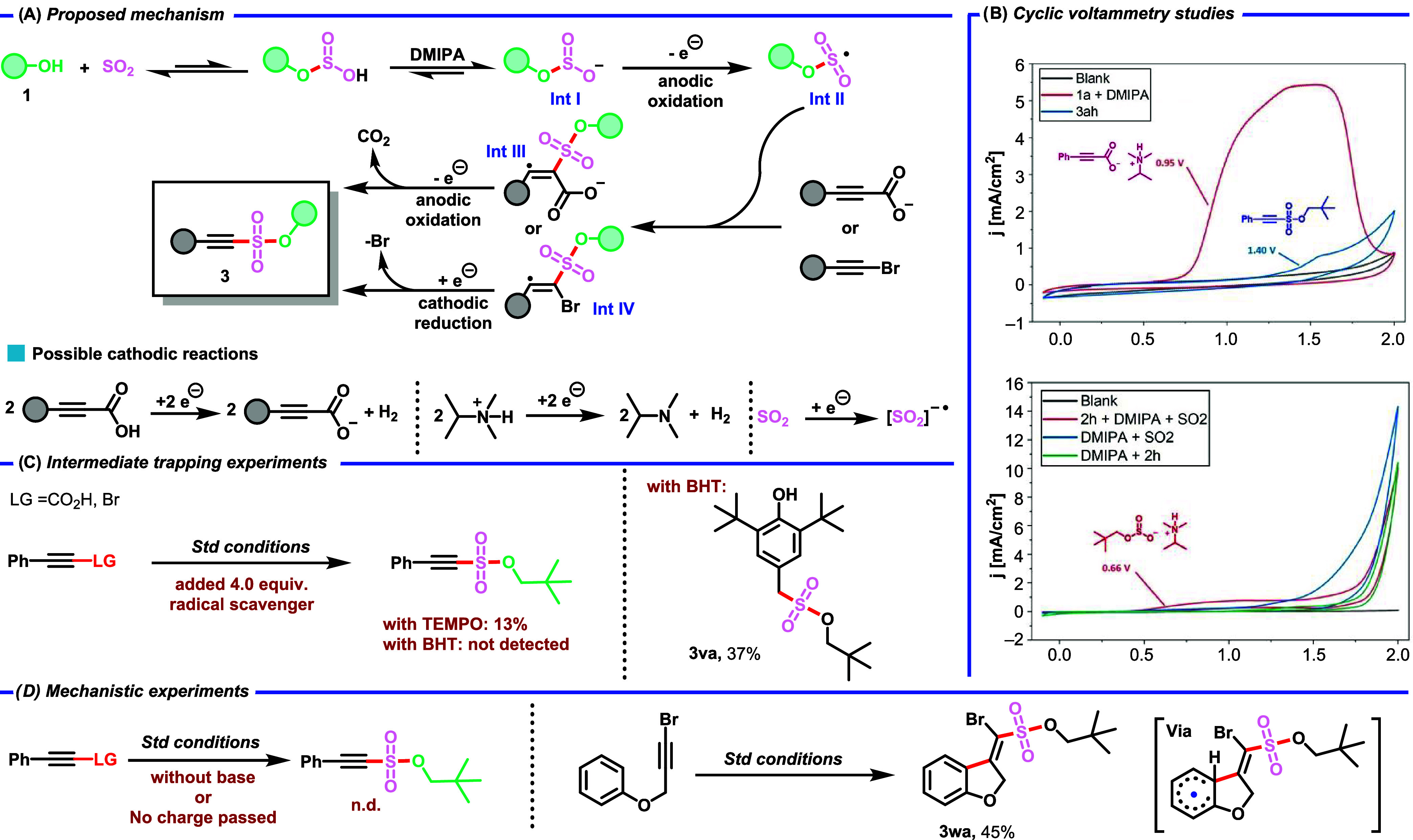
Mechanistic Studies; (A) Proposed Mechanism; (B) Cyclic Voltammetry Studies; (C) Intermediate Trapping Experiments; (D) Mechanistic Experiments

### Synthesis of 1,3-Butadienes via Alkyne Dimerization and Arylation with RB­(OH)_2_

The straightforward one-pot synthesis of alkenyl sulfonates and sulfonamides offers the chance to develop modular synthetic methods based on C–C triple bonds through further manipulations of these functional groups. The combination of arylation and alkyne dimerization^23^ provides a good preparative method for various conjugated 1,3-dienes, which constitute essential scaffolds in natural products and pharmaceuticals,^24^ as well as functional materials.^25^ Additionally, they serve as valuable intermediates in multifunctionalization^26^ and the syntheses of carbocycles and heterocycles.^27^ However, previous approaches for the synthesis of 1,3-dienes involve nonregioselective reactions utilizing costly catalysts such as Pd, Au, and Ru, which are sensitive to air and moisture.^28^ In addition, the absence of effective methods for the synthesis of alkenyl sulfonates hampered the preparation of 1,3-dienes with sulfonate substituents. These challenges drove us to find a novel strategy to overcome these limitations. We first investigated the reactivity and regio- and *E*/*Z*-selectivity of alkenyl sulfonates in reaction with phenylboronic acid (Scheme 4A). As alkyne dimerization has historically involved a wide range of catalysts, established conditions from the literature^29^ (entries 1–3, Table S5) were applied to this novel alkynyl motif, yet all of these conditions failed to yield the desired 1,3-butadiene. After careful optimization of different catalysts, it was found that a high yield can be achieved by carbocupration with 10 mol % Cu­(OAc)_2_ as an inexpensive catalyst in MeOH at room temperature (entry 14, Table S5). Contrary to previous reports, complete control over the regioselectivity of the triple-bond dimerization was achieved, owing to the polarization of the acetylene fragment by the electron-withdrawing sulfonate moiety. The stereoselective formation of highly substituted 1,3-butadiene can be rationalized as a migratory insertion of the alkyne into the Ar–Cu bond. With the optimized conditions at hand, the scope of this regio- and *E*-selective dimerization was explored. Arylboronic acids, regardless of their electronic nature, consistently afforded the products in synthetically useful yields. A broad range of functional groups could be employed, such as alkenes (**4g**), halogens (**4h**–**4m**), esters (**4n**), aldehydes (**4o**), and imides (**4p**), as well as heterocycles such as indole (**4q**, **4r**) and carbazole (**4s**). Notably, the alkenylation of **3ah** with 1-phenylvinylboronic acid under similar conditions gave linear 1,3,5-triene **4t** in 68% yield.

**Scheme 4:**
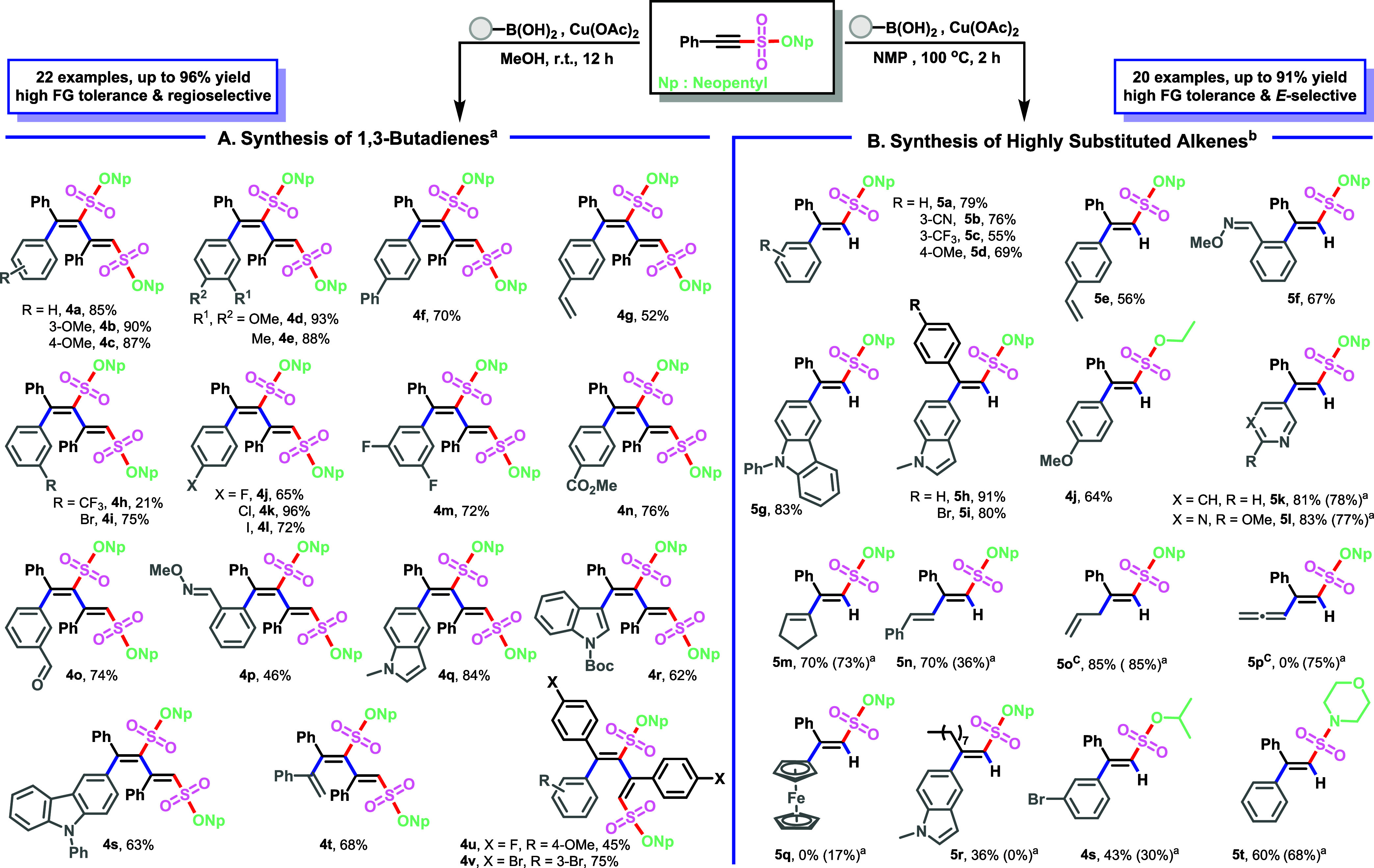
Copper-Catalyzed Dimerizations and Hydroarylations; (A) Synthesis of 1,3-Butadienes; (B) Synthesis of Highly Substituted Alkenes

a Reaction conditions A: **3ah** (0.1 mmol), RB­(OH)_2_ (0.3 mmol), Cu­(OAc)_2_ (10 mol %) and MeOH (0.5 mL).
b Reaction conditions B: **3** (0.1 mmol), RB­(OH)_2_ (0.3 mmol), Cu­(OAc)_2_ (10 mol %), and NMP (0.5 mL).
c from boronic acid pinacol esters.

### Synthesis of Highly Substituted Alkenes via Hydroarylation of Alkenyl Sulfonates with RB­(OH)_2_

During the synthesis of various 1,3-diene derivatives, hydroarylated side products were consistently observed in 5–20% yield. The previously reported stereoselective synthesis of highly substituted alkene sulfonates and sulfonamides^30^ involved a three-step process that required the use of precious metal: first, a photoredox-catalyzed fluorosulfonyl-borylation followed by a Pd-catalyzed Suzuki–Miyaura cross-coupling in the presence of SPhos, and finally a SuFEx click reaction to ligate sulfonyl fluoride with alcohols and amines. On the other hand, direct methods for the preparation of alkenyl sulfonates either suffer from poor *E*/Z-selectivity or are limited to the synthesis of (*E*)-2-arylethene-1-sulfonates.^13c^ Therefore, further optimization studies were undertaken to redirect the hydroarylation pathway from a byproduct to the major product. Increasing temperature and changing solvent were found to be beneficial to the reaction, and the desired coupling product (**5a**) was ultimately obtained in 82% yield when the reaction was conducted in NMP at 100 °C (entry 7, Table S5). To broaden the generality of this coupling reaction, a variety of arylboronic acids were coupled with **3ah** under standard conditions (Scheme 4B). Alkene sulfonates **5k**–**5t** were obtained in moderate to excellent yields regardless of whether the reaction was conducted in MeOH at room temperature (conditions A) or in NMP at 100 °C (conditions B); however, the yield was slightly higher under conditions B.

Notably, for these particular boronic acids, no dimerization products **4** were observed under either set of conditions. As shown in Scheme 4B, this protocol can be used not only for the hydroarylation but also for facile hydroallylation (**5o**), hydroallenylation (**5p**), and hydroferrocenylation (**5q**) of the alkenyl sulfonates. The importance of the allyl and allenyl groups relates to their presence in biologically active natural products and their wide synthetic versatility, serving as key intermediates for derivatization and coupling reactions.^31^ The absence of 1,3-diene formation under conditions A for compounds **5r**–**5t** indicates that 1,3-diene formation is highly dependent on the steric hindrance of the substituents on the alkyne. To determine the origin of the hydrogen atom in copper-catalyzed reactions, a series of control experiments were carried out (Figures S4 and S5).

### Other Product Transformations

To demonstrate the practicality of our electrochemical protocol in the synthesis of heterocycles (Scheme 5A), we first conducted a 1,3-dipolar reaction between an in situ generated Münchnone (from acetic anhydride and proline) and alkenyl sulfonate **3ah**. The corresponding 2,3-dihydro-1*H*-pyrrolizine **6a** was obtained in 72% yield.^32^ Pyrazolo­[1,5-*a*]­pyridine **6b** was synthesized via a desulfonylation reaction. Furthermore, the transformations in Scheme 5A represent a nucleophilic phosphine-catalyzed [3 + 2] annulation between alkenyl sulfonate **3ah** and *N*-hydroxyphthalimide (NHPI), yielding the fused tricyclic product **6c**.^33^ Hydrolysis of alkenyl sulfonate also proceeded smoothly, generating β-keto sulfonate **6d** in 87% yield, a synthetically valuable motif for further functionalization. The use of octasulfur in the synthesis of compound **6e** represents the first report of a [2 + 2 + 1] strategy for the synthesis of valuable dihydrothiophenes, which are important motifs in natural or non-natural product synthesis.^34^ A catalyst-free desulfonative C­(sp)–P coupling reaction formed the corresponding alkynyl di­(phenyl)­phosphine oxide **6f** in 58% yield. Moreover, fully regio- and stereoselective functionalization of the electrochemically generated **3ah** was conducted via the reaction with *N*, *O*, and *S*-nucleophiles (Scheme 5B). These transformations highlight the significant synthetic potential of this electrochemical MCR reaction. An efficient transformation of **3ah** into β-aminovinyl sulfonates takes place successfully in the presence of a range of *N*-nucleophiles, affording **6g**–**6j** in yields of up to 89%. Alkynyl motifs were found to be suitable substrates for hydrothiolation and, contrary to previous reports,^30b^ exclusively afforded the *Z*-isomers (**6k**–**6o**) in high yields with excellent stereoselectivity. Gram-scale reactions were also conducted, affording 0.800 g (99%) and 0.786 g (98%) of compounds **6m** and **6o**, respectively. With compound **6o** in hand, we attempted a metal-free desulfonative synthesis of *N*-fused benzimidazo­[2,1-*b*]­thiazoles, which have shown promising biological activities.^35^ In the presence of Cs_2_CO_3_ as a base, *N*-fused heterocycle **7a** was synthesized in 80% yield. Interestingly, hydroalkoxylation involving 4-hydroxycoumarin yielded the *Z*-isomer **6p**, in contrast to the *syn*-addition products reported in previous studies.^36^ The intermolecular addition of hydroxybenzotriazole to **3ah** at room temperature within 20 min, regio- and stereoselectively gives vinyl ether **6q** without requiring a gold catalyst.^37^ Subsequently, access to sulfonate-substituted enol esters (**6r**–**6w**) was explored through the nucleophilic modification of **3ah**. The versatility of this hydrocarboxylation method was then demonstrated by applying it to the modification of pharmaceutical compounds (**6s**–**6w**). We have shown that employing nucleophilic addition on our electrochemical products would provide an exciting opportunity for linking complex molecules and synthesizing bifunctional compounds (**6v**,**6w**), thereby making this a very useful protocol for chemical biology applications. It is worth mentioning that the formation of *E*-isomers in compounds **6g**, **6h**, and **6q**, synthesized without base, suggests an intramolecular proton transfer mechanism (*syn*-addition),^38^ in contrast to the *Z*-selectivity observed in other nucleophilic addition products (**6i**–**6p**, **6r**–**6w**) where Et_3_N was used as the base, likely involving an intermolecular proton transfer (*anti*-addition).

**Scheme 5:**
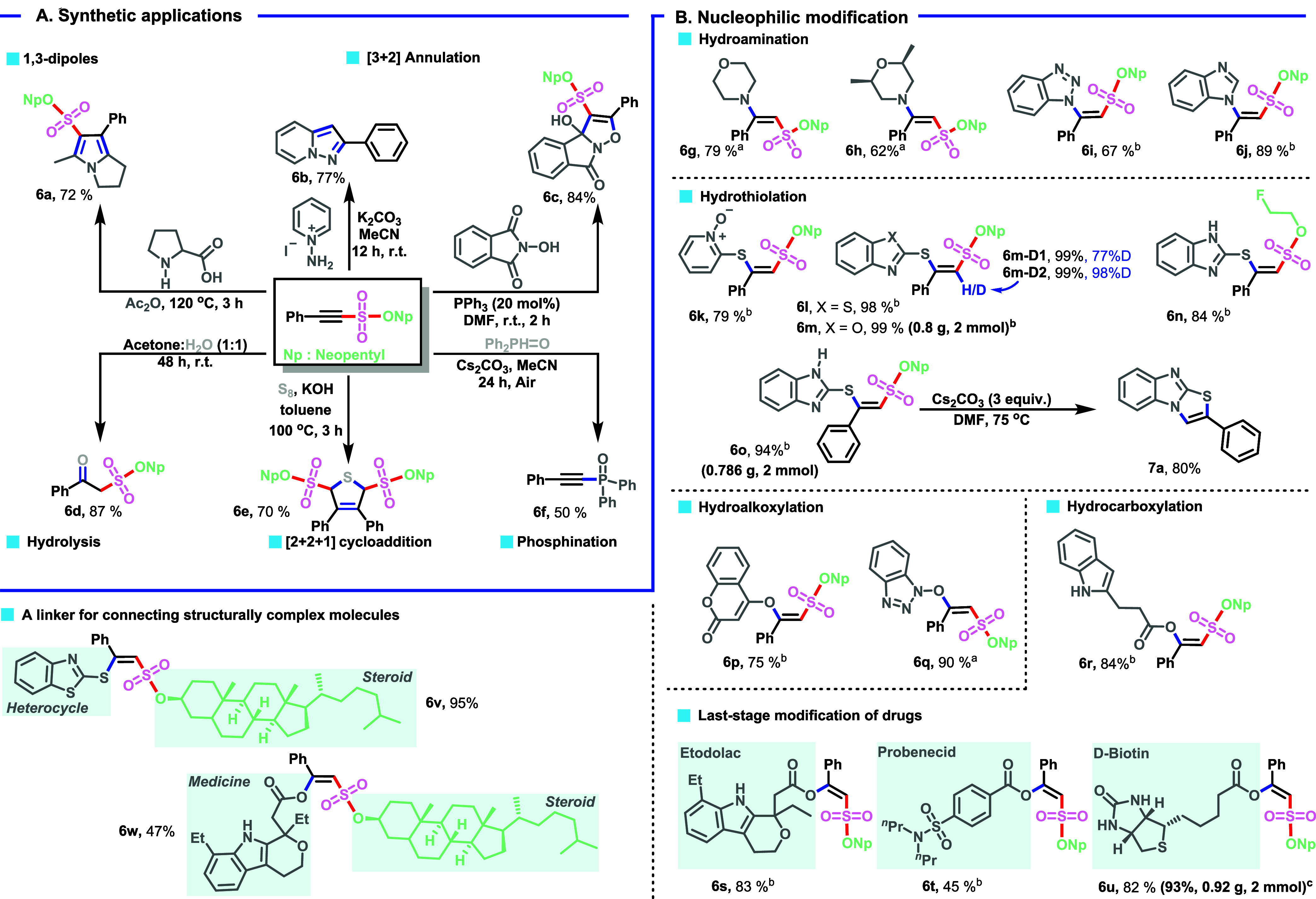
Product Transformations; (A) Synthetic Applications; (B) Nucleophilic Modification[Fn s5fn4]

a without base in MeCN as solvent.
b Et_3_N (2 equiv) as base in MeCN as solvent.
c Et_3_N (2 equiv) as base in DMF as solvent.
d
Supporting Information for detailed experimental conditions.

## Conclusion

In summary, here we have developed the first electrochemical multicomponent reaction (eMCR) approach to valuable alkyne sulfonates and sulfonamides in a one-step synthesis using SO_2_ stock solutions as the sulfur source. This metal- and catalyst-free strategy utilizes electricity as a green oxidant without requiring any supporting electrolyte, offers excellent functional group tolerance and a broad substrate scope, and enables the efficient synthesis of 1,3-dienes, highly substituted alkenes, and various heterocycles. The practicality of the electro-oxidative reaction was confirmed through gram-scale reactions and numerous downstream functionalizations to indicate the potential of the electrochemical products to be valuable intermediates in natural product synthesis, pharmaceutical discovery, and materials construction. The current research is an indication of the strength of electrochemical MCRs to be potent tools in modern synthetic methodology.

## Supplementary Material



## Notes

Experimental procedures, ^1^H, ^19^F, ^13^C, 2D NMR spectra, characterization data of compounds, and crystal structures (PDF)
