# Supplementary material for: Electrochemical One-Step Synthesis of Alkyne Sulfonates and Sulfonamides: Building Blocks for Highly Substituted Alkenes and 1,3-Butadienes
Source: JACS Au. 2025 Oct 23;5(11):5493–503. doi: 10.1021/jacsau.5c00972 (PMC12648319; doi:10.1021/jacsau.5c00972)
Supplement: Supplementary file 1 [file au5c00972_si_001.pdf]

Electrochemical One-Step Synthesis of Alkyne Sulfonates and Sulfonamides: Building Blocks for Highly Substituted Alkenes & 1,3-Butadienes

Meysam Azizzade, Florian A. Breitschaft, Siegfried R. Waldvogel, and Till Opatz*

Table of Contents

Contents

General Methods and Materials S3

Preparation of the SO_2 stock solutions S3

Optimization of electrochemical reaction S3

Optimization of copper catalyzed reactions S7

Radical Trapping Experiment S8

Cyclic Voltammetry Studies S9

Mechanistic Proposal for Electrochemical Reactions S9

Deuteration Experiments S10

Mechanistic Proposal for Copper-Catalyzed Reactions S14

General Procedures S16

X-ray Structure of BP1 .... S21

X-ray Structure of 4k S22

Compounds Characterization Data S24

References S241

General Methods and Materials

Reagents were purchased from Sigma-Aldrich, BLD Pharm, TCI, Fisher Sci., and used without further purificaon. Glassware was dried in an oven or with a flame and under vacuum. Column flash chromatography was performed using silica gel 60 (230−400 mesh). Analytical thin-layer chromatography (TLC) was performed using silica gel aluminum sheets with a fluorescence indicator. Compounds were visualized on TLC by UV-light, or even sprayed with either $KMnO_4$ reagent. Reaction chemical yields refer to chromatographically and spectroscopically pure compounds unless otherwise noted. ^{1}H, ^{13}C NMR, NOESY spectra were recorded on Bruker NMR spectrometers (300, 400 or 600 MHz for ^{1}H, 75, 101 or 150 MHz for ^{13}C and 377 MHz for ^{19}F). ^{1}H NMR spectra are calibrated with respect to the corresponding solvent residual peak ($CHCl_3$: 7.26 ppm). ^{13}C NMR spectra were recorded with complete proton decoupling and the spectra are calibrated with respect to the corresponding solvent residual peak ($^{13}CDCl_3$: 77.16 ppm),Chemical shifts (δ) are reported in parts per million relatively to the residual solvent signals , coupling constants (*J*) are reported in Hertz. The following abbreviations indicate the multiplicity of ^{1}H signals: (s), singlet; (d), doublet; (t), triplet; (q), quartet; (sept), septet; (m), multiplet; and combinations thereof. Accurate mass determinations were made on a G6545A Q-ToF (Agilent GmbH, Waldbronn, Germany) with electrospray ionization (ESI). Sample inlet was via a 1260 Infinity II HPLC system (Agilent GmbH, Waldbronn, Germany) with G7111B 1260 Quaternary Pump, G7129A 1260 Vial sampler, and G7116A 1260 Multicolumn Thermostat. Mass calibration was performed on the day of measurement using an external standard. The mass accuracy of the measurement results is better than 5 ppm.

Preparation of the SO_2 stock solutions

In a gas-inlet apparatus connected to a washing bottle filled with aq. NaOH (20 wt-%) with Woulff bottles placed before and after the washing bottle, MeCN or DMSO (300 mL, anhydrous) was enriched with SO_2 at 0 °C under constant stirring for 30-60 minutes. After usage, the gas-inlet apparatus was flushed with Argon and molecular sieve (3 Å) was added to the obtained solution. The stock solution was sealed by a septum stopper and stored in a fridge at 4 °C. The SO_2 molarity was determined according to the principles of the "Excess Iodine Method" described by Ferguson.[1] To a solution of I_2 (1.27 g, 5.00 mmol) and KI (2.20 g, 13.3 mmol) in H_2O (100 mL) was slowly added the freshly prepared SO_2 stock solution (0.5 mL). The solution was then back titrated with a freshly prepared solution of $Na_2S_2O_3$ (aq., 0.2 M) as titrant. After full reduction of the iodine, the concentration of SO_2 (mostly 4–9 M) was calculated according to the previously reduced iodine by aq. SO_2. The titration was conducted three successive times, and the molarity of the stock solution was determined by averaging the three measurements.

Failed Scope Examples

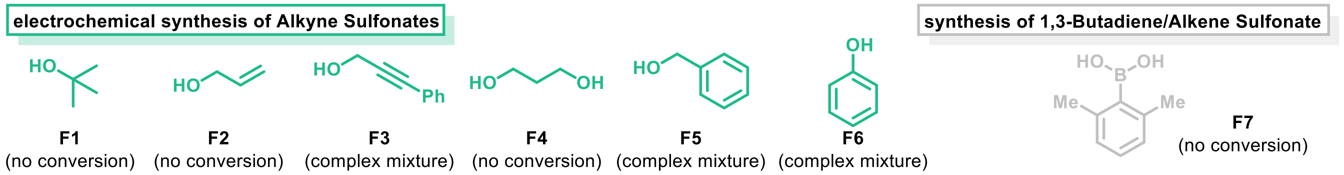

Figure S1: Failed substrates.

Optimization of electrochemical reaction

Solvent optimization

An oven-dried 10 mL Electrasyn 2.0 vial equipped with a stir bar, was charged with 3-phenylpropiolic acid (88 mg, 0.6 mmol), 2,2-dimethyl-1-propanol (264 mg, 3.0 mmol, 5 equiv.), tetrabutylammonium tetrafluoroborate (198 mg, 1 equiv.), then 6 mL dry **solvent**, DIPEA (209 µL, 151 mg, 2 equiv.) and SO_2 in MeCN (0.96 mL, 5.0 M, 8 equiv.) were added. The vial was then connected to an IKA ElectraSyn 2.0 stirring plate, and electrolysis was carried out at 10 mA (27 mm × 8 mm was fully submerged into the solution, 2.16 cm^2, 4.63 mA/cm^2), 0.6 mmol, 6 h, 800 rpm. An ElectraSyn 2.0 cap, fitted with a graphite (SK-50) (2 mm x 8 mm x 52 mm) anode and a platinum foil electrode

cathode (0.2 mm x 8 mm x 52 mm), was used to close the vial. Upon completion of the programmed electrolysis, 26.4 mg of ethylene carbonate was introduced into the reaction mixture as an internal standard. The solution was stirred for 5 min at room temperature and then 200 µL of the mixture was taken for qNMR analysis using a ^{1}H NMR spectrum at 300 MHz.

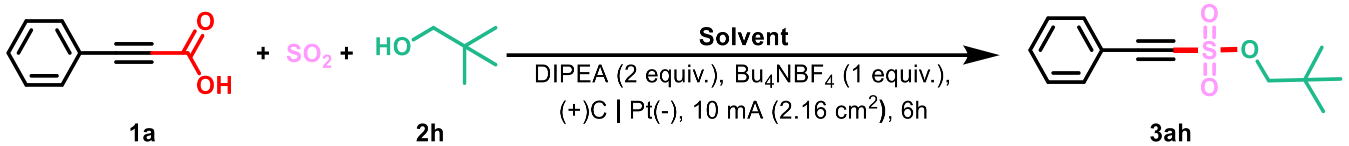

1a 2h 3ah

Entry	solvent	Yield (%)[a]
1	ACN	< 8 %
2	DMF	n.d.
3	Acetone	n.d.
4	DMSO	n.d.
5	Dioxane	n.d.
6	DCM	10%
7	PhCl	< 8 %
8[b]	DCM	18%

n.d., not detected, [a] NMR yield, [b] SO_2 in DMSO (0.8 mL, 6.0 M, 8 equiv.)

Table S1: Solvent screening.

Current density and* amount of applied charge *optimization

An oven-dried 10 mL Electrasyn 2.0 vial equipped with a stir bar, was charged with 3-phenylpropiolic acid (88 mg, 0.6 mmol), 2,2-dimethyl-1-propanol (264 mg, 3.0 mmol, 5 equiv.), tetrabutylammonium tetrafluoroborate (198 mg, 1 equiv.), then 6 mL dry DCM, DIPEA (234 µL, 167 mg, 3.2 equiv.) and SO_2 in DMSO (0.8 mL, 6.0 M, 8 equiv.) were added. The vial was then connected to an IKA ElectraSyn 2.0 stir plate, and electrolysis was carried out at **I mA** (27 mm × 8 mm was fully submerged into the solution, 2.16 cm^2, **j mA/cm^2**), 0.6 mmol**, time (Amount of charge Q)**, 800 rpm. An ElectraSyn 2.0 cap, fitted with a graphite (SK-50) (2 mm x 8 mm x 52 mm) anode and a platinum foil electrode cathode (0.2 mm x 8 mm x 52 mm), was used to close the vial. Upon completion of the programmed electrolysis, 26.4 mg of ethylene carbonate was introduced into the reaction mixture as an internal standard. The solution was stirred for 5 min at room temperature and then 200 µL of the mixture was taken for qNMR analysis using a ^{1}H NMR spectrum at 300 MHz.

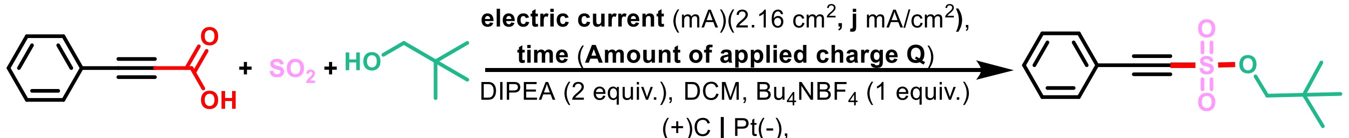

1a 2h 3ah

entry	**electric current** (mA) (2.16 cm^2, **j** mA/cm^2)	**time** (Amount of applied charge Q)	**yield (%)**[a]
1	7 mA (3.24 mA/cm^2)	515 min ($Q_{applied}$ = 216 C, $Q_{Faraday}$ = 3.73 F)	< 8 %
2	10 (4.63 mA/cm^2)	360 min ($Q_{applied}$ = 216 C, $Q_{Faraday}$ = 3.73 F)	18%
3	12 (5.55 mA/cm^2)	300 min ($Q_{applied}$ = 216 C, $Q_{Faraday}$ = 3.73 F)	21%
4	15 (6.94 mA/cm^2)	240 min ($Q_{applied}$ = 216 C, $Q_{Faraday}$ = 3.73 F)	27%
5	18 (8.33 mA/cm^2)	200 min ($Q_{applied}$ = 216 C, $Q_{Faraday}$ = 3.73 F)	27%
6	20 (9.26 mA/cm^2)	180 min ($Q_{applied}$ = 216 C, $Q_{Faraday}$ = 3.73 F)	23%
7	15 (6.94 mA/cm^2)	300 min ($Q_{applied}$ = 270 C, $Q_{Faraday}$ = 4.66 F)	31%
8	15 (6.94 mA/cm^2)	360 min ($Q_{applied}$ = 324 C, $Q_{Faraday}$ = 5.59 F)	36%
9	15 (6.94 mA/cm^2)	420 min ($Q_{applied}$ = 378 C, $Q_{Faraday}$ = 3.73 F)	20%

[a] NMR yield.

Table S2: Current and amount of applied charge screening.

Base optimization

An oven-dried 10 mL Electrasyn 2.0 vial equipped with a stir bar, was charged with 3-phenylpropiolic acid (88 mg, 0.6 mmol), 2,2-dimethyl-1-propanol (264 mg, 3.0 mmol, 5 equiv.), tetrabutylammonium tetrafluoroborate (198 mg, 1 equiv.), then 6 mL dry DCM, **base** and SO_2 in DMSO (0.8 mL, 6.0 M, 8 equiv.) were added. The vial was then connected to an IKA ElectraSyn 2.0 stir plate, and electrolysis was carried out at 15 mA (27 mm × 8 mm was fully submerged into the solution, 2.16 cm^2, 6.94 mA/cm^2), 0.6 mmol, 360 min, 800 rpm. An ElectraSyn 2.0 cap, fitted with a graphite (SK-50) (2 mm x 8 mm x 52 mm) anode and a platinum foil electrode cathode (0.2 mm x 8 mm x 51 mm), was used to close the vial. Upon completion of the programmed electrolysis, 26.4 mg of ethylene carbonate was introduced into the reaction mixture as an internal standard. The solution was stirred for 5 min at room temperature and then 200 μL of the mixture was taken for qNMR analysis using a ^1H NMR spectrum at 300 MHz.

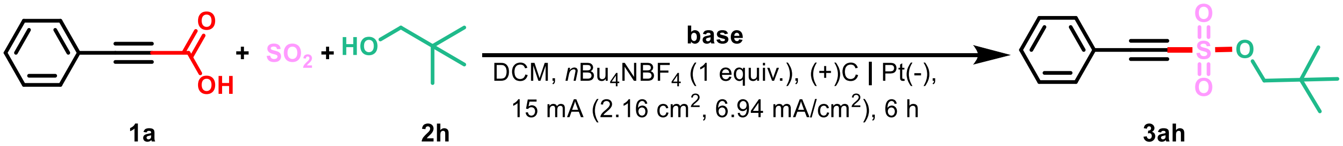

1a **2h** **3ah**

entry	base	yield (%)[a]
1	*N,N*-Diisopropylethylamine (DIPEA)	36%
2[b]	1,8-Diazabicyclo(5.4.0)undec-7-ene (DBU)	55% - 96%
3[b,c]	1,8-Diazabicyclo(5.4.0)undec-7-ene (DBU)	25%
4[b]	1,5-Diazabicyclo(4.3.0)non-5-en (DBN)	30% - 60%
5	Triethylamine (TEA)	12%
6	*N,N*-Dimethylisopropylamine (DMIPA)	52%
7	1-Methylpiperidine	49%
8	1-Methylpyrrolidine	49%
9[b]	1,1,3,3-Tetramethylguanidine	30% - 60%
10	2-*tert*-Butyl-1,1,3,3-tetramethylguanidine (BTMG)	n.d.
11	2,4,6-Collidine	47%
12	2,6-Lutidine	45%
13[c]	2,4,6-Collidine	32%
14	1,2-Dimethyl-1,4,5,6-tetrahydropyrimidine	30% - 70%
15[c]	*N,N*-Dimethylisopropylamine (DMIPA)	59%

n.d., not detected, [a] NMR yield,[b] The yield of the reaction was not reproducible and showed significant variation over a broad range, [c] base (3.2 equiv.).

Table S3: Base screening.

Note 1: Reaction yields were found to be irreproducible when DBU, DBN, 1,1,3,3-Tetramethylguanidine or 1,2-Dimethyl-1,4,5,6-tetrahydropyrimidine were used as bases, most likely due to their side reactions with the final product, particularly in the presence of trace amount of water. To demonstrate this effect, the following reaction was carried out:

*A 10 mL reaction tube was charged with 2-phenylethyne-1-sulfonate (**3ah**) (50.4 mg, 0.2 mmol), DBU (33 μL,335 mg, 0.22 mmol, 1.1 equiv.), DCM (0.5 mL) and a magnetic stir bar. Subsequently, the reaction mixture was stirred for 1 min at room temperature. Once the time has passed, the reaction mixture was diluted with Diethyl ether(1 mL), then the mixture was placed in the freezer. After 12 hours, the formed crystals were washed with a small amount of pentane to afford the final product as a pure colorless crystals. Yield 99% (80 mg) (**BP1**, see spectra).*

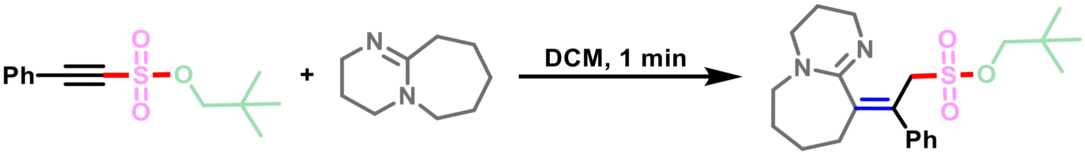

3ah (0.2 mmol) **DBU** (1.1 equiv.) **BP1**, 99 %

Note 2: variation of the base loading between 2.0 and 4.0 equivalents demonstrated that the highest yield was achieved when 3.2 equivalents of base were employed.

Electrode and electrolyte optimization

An oven-dried 10 mL Electrasyn 2.0 vial equipped with a stir bar, was charged with 3-phenylpropiolic acid (88 mg, 0.6 mmol), 2,2-dimethyl-1-propanol (264 mg, 3.0 mmol, 5 equiv.), **supporting electrolyte**, then 6 mL dry DCM, DMIPA (234 µL, 167 mg, 3.2 equiv.) and SO_2 in DMSO (0.8 mL, 6.0 M, 8 equiv.) were added. The vial was then connected to an IKA ElectraSyn 2.0 stir plate, and electrolysis was carried out at 15 mA (27 mm × 8 mm was fully submerged into the solution, 2.16 cm^2, 6.94 mA/cm^2), 0.6 mmol, 360 min, 800 rpm. An ElectraSyn 2.0 cap, fitted with **electrodes**, was used to close the vial. Upon completion of the programmed electrolysis, 26.4 mg of ethylene carbonate was introduced into the reaction mixture as an internal standard. The solution was stirred for 5 min at room temperature and then 200 µL of the mixture was taken for qNMR analysis using a ^1H NMR spectrum at 300 MHz.

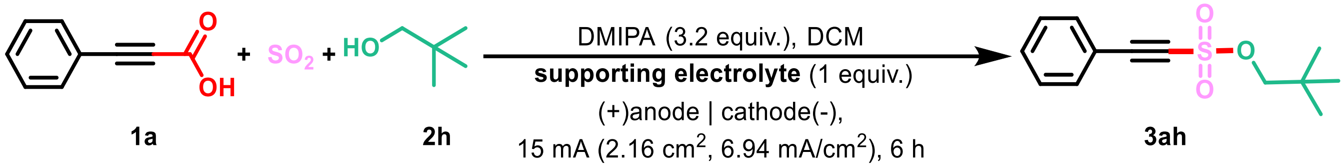

entry	electrolyte	Anode | cathode	yield (%)[a]
1	Bu_4NBF_4 (1 equiv.)	C | Pt	59%
2	Bu_4NBF_4 (0.5 equiv.)	C | Pt	63%
3	Me_4NBF_4 (0.5 equiv.)	C | Pt	63%
4	Bu_4NPF_6 (0.5 equiv.)	C | Pt	50%
5	Bu_4NClO_4 (0.5 equiv.)	C | Pt	< 8 %
6	KPF_6 (0.5 equiv.)	C | Pt	73%
7	-	C | Pt	73% (71%)[b]
8	-	C | Cu or C | SS or C | Ni	73% (71%)[b]
9[c]	-	C | Cu	73% (71%)[b]
10[d]	-	C | Cu	65%
11[e]	-	C | Cu	73% (71%)[b]
12	-	GC | Cu	< 8 %
13	-	RVC | Cu	< 8 %
14	-	Cu | Cu or Pt | Pt or Ni | Ni or SS | SS	n.d.
15[f]	-	C | Cu	73% (71%)[b]
16[g]	-	C | Cu	54%
17[h]	-	C/Cu	25%

n.d., not detected, [a] NMR yield, [b] Isolated yield, [c] 2,2-dimethyl-1-propanol (**2h**, 4.5 equiv.), [d] 2,2-dimethyl-1-propanol (**2h**, 4.0 equiv.), [e] 315 min ($Q_{applied}$ = 280 C, $Q_{Faraday}$ = 4.84 F), [f] The reaction was performed in the setup shown in Figure S8, instead of using an ElectraSyn 2.0, [g] 4 × AA batteries as the power source, [h] a copper wire (d = 1 mm) was used.

Table S4: Electrode and electrolyte screening.

Note 1: Nickel, stainless steel, platinum, and copper can all be used as suitable cathodes for this reaction.

Note 2: The minimum amount of alcohol can be 4.5 equivalents, and the minimum $Q_{applied}$ can be 280 C. Increasing either of these parameters does not improve the reaction, while decreasing them significantly reduces the reaction yield.

Note 3: Decreasing the electrode distance to less than 4 mm significantly reduces the reaction yield.

*Note 4: The lower yields observed with electrolyte (table S4, entries 1-4) arise from alcohol and SO_2 consumption during the side reaction that produces dineopentyl sulfite. A 15% yield of dineopentyl sulfite (**BP2**, see spectra) was obtained from the electrolyte-containing reaction mixture, whereas this by-product was not detected in the absence of electrolyte. Comparable side reaction were also detected with other alcohol substrates, such as 4-phenyl-1-butanol (**BP3**, see spectra).*

Leaving group and additive optimization

An oven-dried 10 mL Electrasyn 2.0 vial equipped with a stir bar, was charged with **alkyne** (0.6 mmol), 2,2-dimethyl-1-propanol (238 mg, 3.0 mmol, 5 equiv.), **additive**, then 6 mL dry DCM, DMIPA (234 µL, 167 mg, 3.2 equiv.) and SO_2 in DMSO (0.8 mL, 6.0 M, 8 equiv.) were added. The vial was then connected to an IKA ElectraSyn 2.0 stir plate, and electrolysis was carried out at 15 mA (27 mm × 8 mm was fully submerged into the solution, 2.16 cm^2, 6.94 mA/cm^2), 0.6 mmol, 315 min, 800 rpm. An ElectraSyn 2.0 cap, fitted with a graphite (SK-50) (2 mm x 8 mm x 52 mm) anode and a copper electrode cathode (2 mm x 8 mm x 52 mm), was used to close the vial. Upon completion of the programmed electrolysis, 26.4 mg of ethylene carbonate was introduced into the reaction mixture as an internal standard. The solution was stirred for 5 min at room temperature and then 200 µL of the mixture was taken for qNMR analysis using a ^{1}H NMR spectrum at 300 MHz.

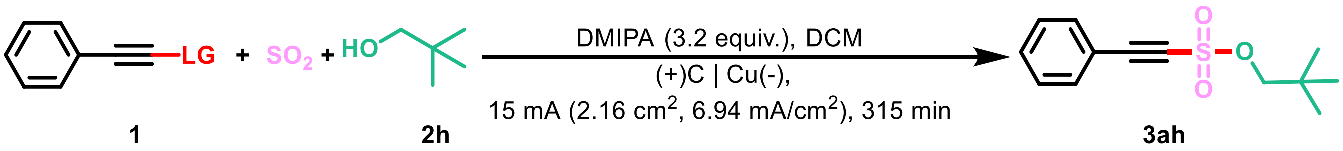

entry	Leaving group	Additive (1 equiv.)	yield (%)[a]
1	CO_2H	-	73%(71%)[b]
2	CO_2Na	-	66%
3	Br	-	69%(97%)[b]
4	H	-	30%
5	H	TFA (1 equiv.)	31%
6	H	AcOH (1 equiv.)	< 8 %
7	H	3,5-Bis(trifluoromethyl)benzoic acid (1 equiv.)	39% (35%)[b]
8	CO_2H	3,5- Bis (trifluoromethyl)benzoic acid (1 equiv.)	70%
9	Br	3,5- Bis (trifluoromethyl)benzoic acid (1 equiv.)	69%
10	CO_2H	2,6-Di-*tert*-butyl-4-methylphenol (BHT, 4 equiv.)	n.d.
11	Br	2,6-Di-*tert*-butyl-4-methylphenol (BHT, 4 equiv.)	n.d.
12	CO_2H	(2,2,6,6-Tetramethylpiperidin-1-yl)oxyl (TEMPO, 4 equiv.)	13%

n.d., not detected, [a] NMR yield, [b] Isolated yield

Table S4: Leaving group and additive screening.

Optimization of copper catalyzed reactions

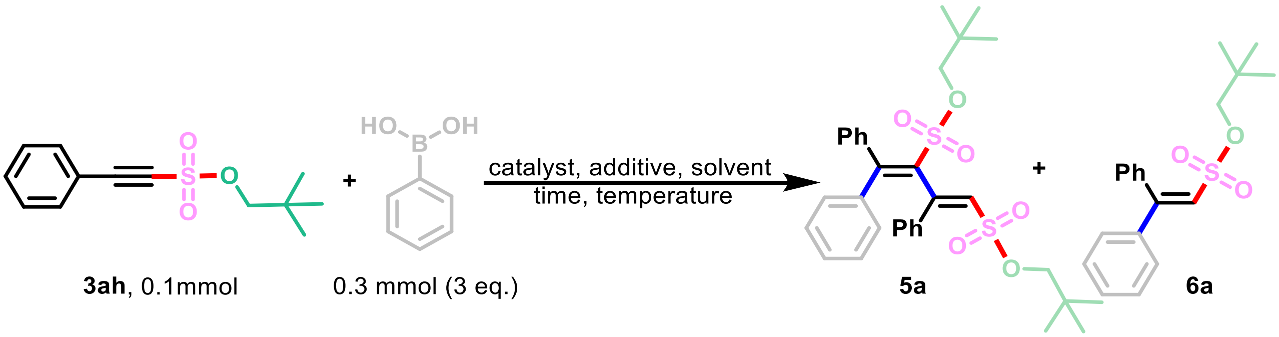

entry	catalyst	additive	solvent	Time/temperature	5a/6a yield (%)[a]
1	$Ni(cod)_2$ (5 mol%)	P(4-MeO–C_6H_4)$_3$ (20 mol%)	MeCN	24 h, 80 °C	n.d. / 5%
2	$Ni(cod)_2$ (3 mol%)	P(i-Pr)$_3$ (12 mol %),	Toluene	24 h, 80 °C	n.d. / n.d.
3	[(Cp*$RhCl_2$)$_2$] (5 mol%)	$Cu(OAc)_2$(0.5 equiv.)	DMF	2 h, 100 °C	n.d./ 37%
4	[(Cp*$RhCl_2$)$_2$] (5 mol%)	-	DMF	2 h, 100 °C	n.d. / n.d.
5	$Cu(OAc)_2$(0.5 equiv.)	-	DMF	2 h, 100 °C	8% / 58%
6	$Cu(OAc)_2$ (10 mol%)	-	DMF	2 h, 100 °C	15%/ 58%
7	$Cu(OAc)_2$ (10 mol%)	-	NMP	2 h, 100 °C	n.d. / 79%

8	$Cu(OAc)_2$ (10 mol%)		DMSO	2 h, 100 °C	n.d. / n.d.
9	$Cu(OAc)_2$ (10 mol%)		MeCN	2 h, 80 °C	n.d. / n.d.
10	$Cu(OAc)_2$ (10 mol%)		Dioxane	2 h, 100 °C	n.d. / n.d.
11	$Cu(OAc)_2$ (10 mol%)		THF	2 h, 60 °C	n.d. / n.d.
12	$Cu(OAc)_2$ (10 mol%)	-	MeOH	2 h, 60 °C	18% / 35%
13	$Cu(OAc)_2$ (10 mol%)	-	NMP	24 h, r.t.	n.d. / n.d.
14	$Cu(OAc)_2$ (10 mol%)	-	MeOH	12 h, r.t.	85% / 10%
15	$Cu(OAc)_2$ (10 mol%)	-	EtOH	12 h, r.t.	49% / 15%

n.d., not detected, [a] Isolated yield

Table S5: optimization of the synthesis of Alkenes & 1,3-Butadienes

Radical Trapping Experiment

An oven-dried 10 mL Electrasyn 2.0 vial equipped with a stir bar, was charged with 3-phenylpropiolic acid (88 mg, 0.6 mmol), 2,2-dimethyl-1-propanol (264 mg, 3.0 mmol, 5 equiv.) and 2,6-di-tert-butyl-4-methylphenol (BHT, 2.4 mmol, 529 mg, 4 equiv.) then 6 mL dry DCM, DMIPA (234 µL, 167 mg, 3.2 equiv.) and SO_2 in DMSO (0.8 mL, 6.0 M, 8 equiv.) were added. The vial was then connected to an IKA ElectraSyn 2.0 stir plate, and electrolysis was carried out at 15 mA (2.7 cm × 0.8 cm × 0.02 cm was fully submerged into the solution, 2.16 cm^2, 6.95 mA/cm^2), 0.6 mmol, 315 min. No product was detected. Instead, **3va** was isolated in 37% yield (*see spectra*).

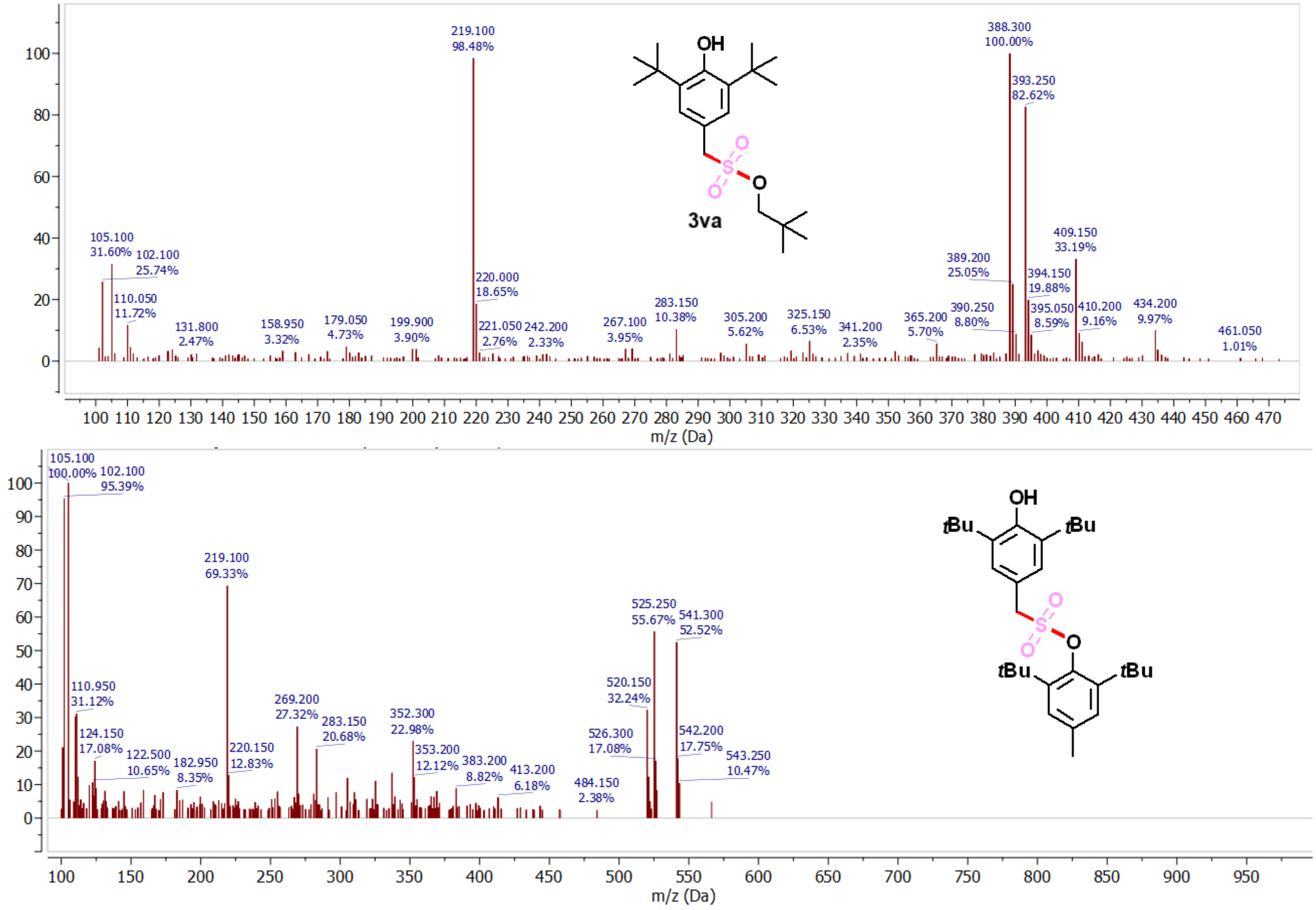

Figure S2: Mass spectrum obtained by LC/MS of a BHT-trapped intermediate

Cyclic Voltammetry Studies

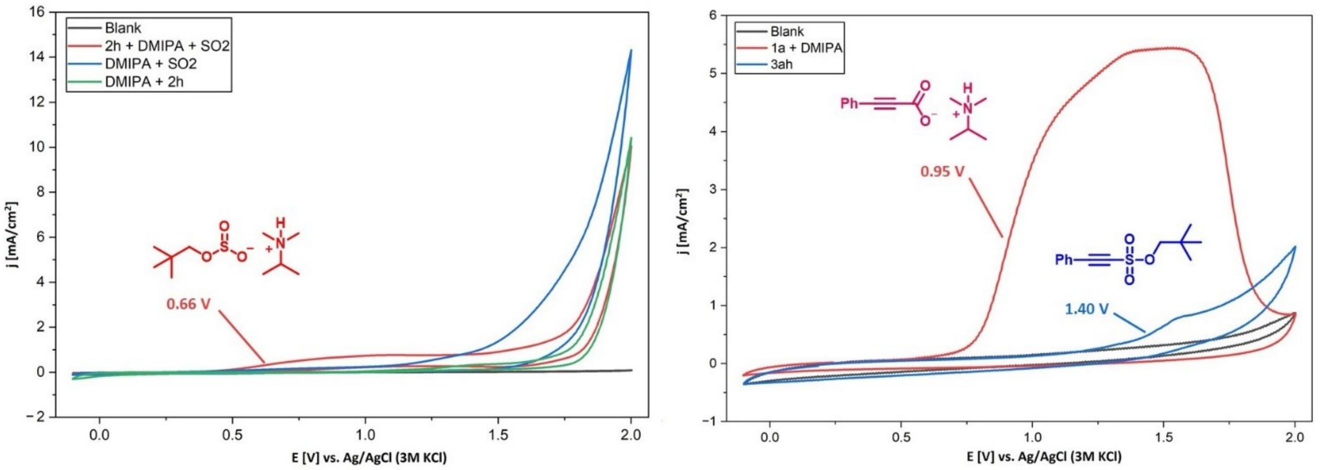

Figure S3: Cyclic voltammograms. Measurement conditions: 0.1 M Bu_4NBF_4 in MeCN; c(substrate) = 10 mmol×L^{-1}; c(SO_2) = 40 mmol×L^{-1}; v = 100 mV×s^{-1}. Solutions were degassed by sparging with a MeCN-saturated N_2 stream for 5 min prior to measuring. Potentials are reported against the Ag/AgCl reference electrode.

As depicted by Figure S3, the oxidation potentials increase in the following order: the intermediate monoalkylsulfite, 3-phenylpropiolate, alkyne Sulfonate **3ah**. It is noteworthy, that **3ah** is not prone to overoxidization due to its significantly higher oxidation potential compared to the intermediate monoalkylsulfite.

Mechanistic Proposal for Electrochemical Reactions

The reaction mechanism proposal is depicted in Scheme S1. Sulfur dioxide and the alcohol form Lewis acid-base adducts, generating the intermediate monoalkylsulfite (**Int I**) in an equilibrium reaction after deprotonation by DMIPA. Initial anodic oxidation of the intermediate monoalkylsulfite forms the radical **Int II**. Subsequently, the attack of the radical **Int II** onto the acetylenic acid anion or the bromoalkyne generated radical **Int III** or **Int IV**, which **Int III** undergoes further oxidation, leading to decarboxylation and formation of the desired product **3**,[2] whereas **Int IV** may undergo cathodic reduction to afford **3**. As cathodic reaction, most likely SO_2 reduction or hydrogen evolution process occurs. [3]

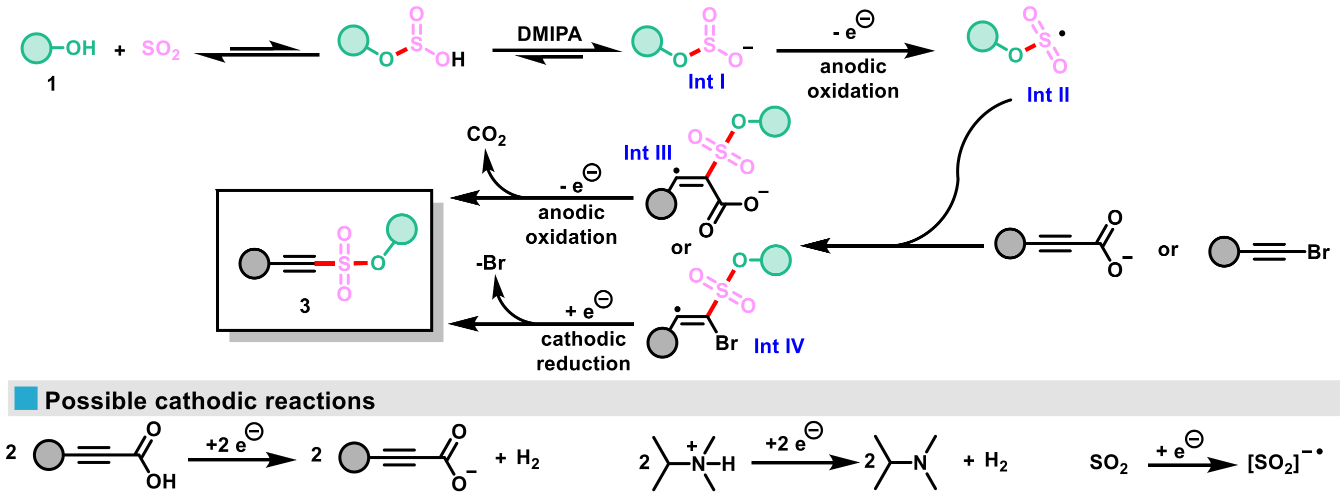

Scheme S1**:** Proposed mechanism for the synthesis of alkyne sulfonates

Deuteration Experiments

(4-Iodophenyl)boronic acid-*d2*

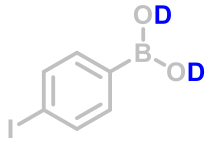

To a 25 mL round bottom flask was added 4-iodophenylboronic acid (620 mg, 2.5mmol,) and $CDCl_3$-D_2O (1:1) 8 mL. Then resulting solution was refluxed at 80 °C for 12 h in oil bath. The reaction mixture was cooled and then After filtration, the colorless solid was dried by using a high vacuum for 6 h. Deuterated 4-iodophenylboronic acid (550 mg, 89%, ≈ 86% D) was obtained.

NMR Spectroscopy (see spectra):
^{1}H NMR (300 MHz, DMSO): δ 8.27 – 8.07 (s, 0.28H), 7.82 – 7.67 (m, 2H), 7.66 – 7.53 (m, 2H).

Benzoxazole-2-thiol-*d*

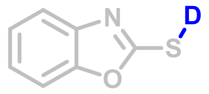

To a 25 mL round bottom flask was added 2-mercapto-benzoxazol (378 mg, 2.5mmol,) and CD_3OD-D_2O (1:1) 8 mL. Then resulting solution was refluxed at 80 °C for 12 h in oil bath. The reaction mixture was cooled and then After filtration, the colorless solid was dried by using a high vacuum for 6 h. Deuterated 2-mercapto-benzoxazol (350 mg, 92%, ≈ 77% D) was obtained.

NMR Spectroscopy (see spectra):
^{1}H NMR (300 MHz, $CDCl_3$): δ 11.05 (s, 0.23H), 7.49 – 7.16 (m, 4H).

4l-D1

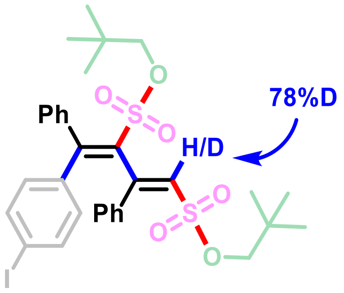

Following the general procedure B. neopentyl 2-phenylethyne-1-sulfonate (**3ah**) (25.2 mg, 0.1 mmol), (4-iodophenyl)boronic acid (74 mg, 0.3 mmol, 3 equiv.), $Cu(OAc)_2$ (1.8 mg), and methanol-d_4 (CD_3OD) (0.5 mL) were used. Purification via column chromatography on silica gel (cyclohexane: ethyl acetate = 94:6, v/v) afforded **4l-D1** as a colorless solid; Yield 70% (24 mg, ≈ 78% D).

NMR Spectroscopy (see spectra):
^{1}H NMR (400 MHz, $CDCl_3$): δ 7.52 (d, J = 8.4 Hz, 2H), 7.41 – 7.34 (m, 3H), 7.32 – 7.25 (m, 5H), 7.24 – 7.19 (m, 2H), 6.92 (s, 0.22H), 6.64 (d, J = 8.4 Hz, 2H), 3.73 (s, 2H), 3.62 (s, 2H), 0.89 (s, 9H), 0.84 (s, 9H).

4l-D2

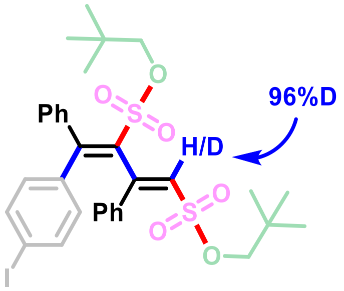

Following the general procedure B. neopentyl 2-phenylethyne-1-sulfonate (**3ah**) (25.2 mg, 0.1 mmol), (4-iodophenyl)boronic acid-d_2 (74 mg, 0.3 mmol, 3 equiv.), $Cu(OAc)_2$ (1.8 mg), and methanol-d_4 (CD_3OD) (0.5 mL) were used. Purification via column chromatography on silica gel (cyclohexane: ethyl acetate = 94:6, v/v) afforded **4l-D2** as a colorless solid; Yield 70% (24 mg, ≈ 96% D).

NMR Spectroscopy (see spectra):
^{1}H NMR (400 MHz, $CDCl_3$): δ 7.52 (d, J = 8.4 Hz, 2H), 7.41 – 7.34 (m, 3H), 7.32 – 7.25 (m, 5H), 7.24 – 7.19 (m, 2H), 6.92 (s, 0.04H), 6.64 (d, J = 8.4 Hz, 2H), 3.73 (s, 2H), 3.62 (s, 2H), 0.89 (s, 9H), 0.84 (s, 9H).

5c-D1

Following the general procedure C. neopentyl 2-phenylethyne-1-sulfonate (**3ah**) (25.2 mg, 0.1 mmol), (3-(trifluoromethyl)phenyl)boronic acid (57 mg, 0.3 mmol, 3 equiv.), (74 mg, 0.3 mmol, 3 equiv.), $Cu(OAc)_2$ (1.8 mg), D_2O (20 mg, 18 µL, 10 equiv) and dry NMP (0.5 mL) were used. Purification via column chromatography on silica gel (cyclohexane: ethyl acetate = 94:6, v/v) afforded **5c-D1** as a colorless solid; Yield 39% (16 mg, ≈ 90% D).

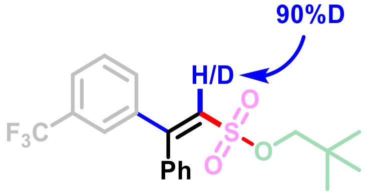

NMR Spectroscopy (see spectra):

^{1}H NMR (400 MHz, $CDCl_3$): δ 7.71 (d, J = 7.6 Hz, 1H), 7.57 – 7.44 (m, 6H), 7.41 – 7.36 (m, 2H), 6.79 (s, 0.1H), 3.77 (s, 2H), 0.93 (s, 9H).

6m-D1

Following the general procedure D. with neopentyl 2-phenylethyne-1-sulfonate (**3ah**) (25.2 mg, 0.1 mmol), dry MeCN (1 mL), benzo[d]oxazole-2-thiol-*d* (15 mg, 0.1 mmol, 1 equiv.) and Et_3N (20 µL, 23 mg, 1.4 mmol, 1.4 equiv.) were used. Purification via column chromatography on silica gel (cyclohexane: ethyl acetate = 80:20, v/v) afforded **6m-D1** as a colorless solid; Yield 99 % (40 mg, ≈ 77% D).

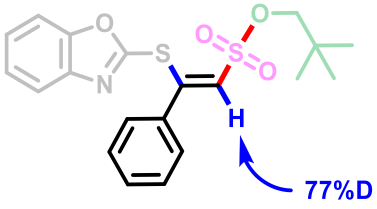

NMR Spectroscopy (see spectra):

^{1}H NMR (300 MHz, $CDCl_3$): δ 7.59 – 7.51 (m, 3H), 7.30 – 7.21 (m, 6H), 6.75 (s, 0.23H), 4.04 (s, 2H), 1.05 (s, 9H).

6m-D2

Following the general procedure D. with neopentyl 2-phenylethyne-1-sulfonate (3ah) (25.2 mg, 0.1 mmol), dry MeCN (1 mL), 2-mercaptobenzoxazole (15 mg, 0.1 mmol, 1 equiv.), D_2O (20 mg, 18 µL, 10 equiv) and Et_3N (20 µL, 23 mg, 1.4 mmol, 1.4 equiv.) were used. Purification via column chromatography on silica gel (cyclohexane: ethyl acetate = 80:20, v/v) afforded **6m-D2** as a colorless solid; Yield 99 % (40 mg, ≈ 98% D).

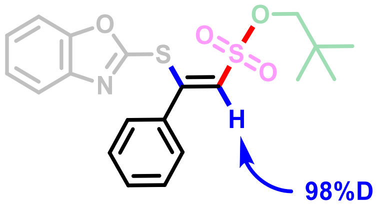

NMR Spectroscopy (see spectra):

^{1}H NMR (300 MHz, $CDCl_3$): δ 7.59 – 7.51 (m, 3H), 7.30 – 7.21 (m, 6H), 6.75 (s, 0.02H), 4.04 (s, 2H), 1.05 (s, 9H).

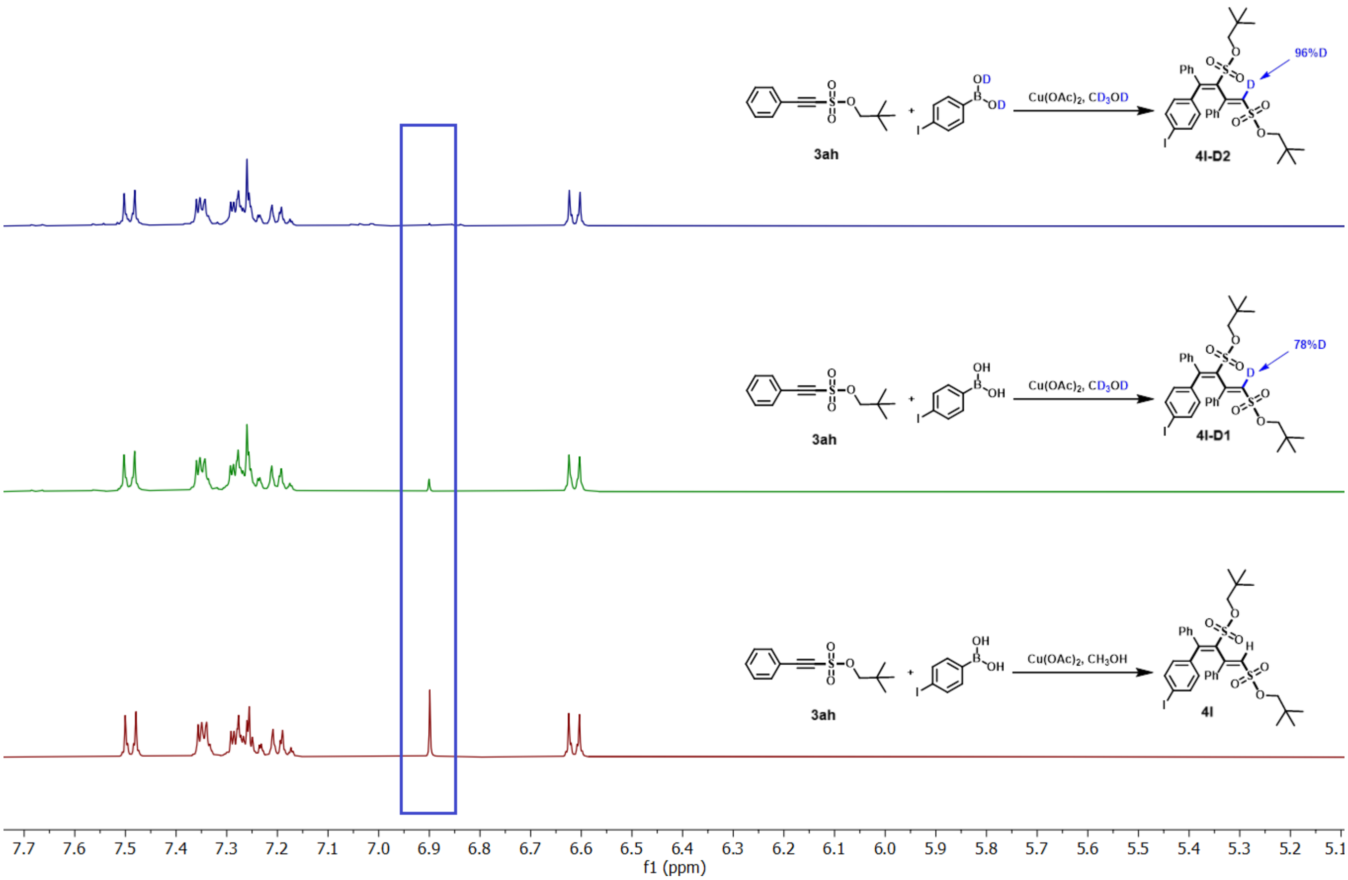

Figure S4: Illustrating the roles of organoboronic acid and methanol as proton sources

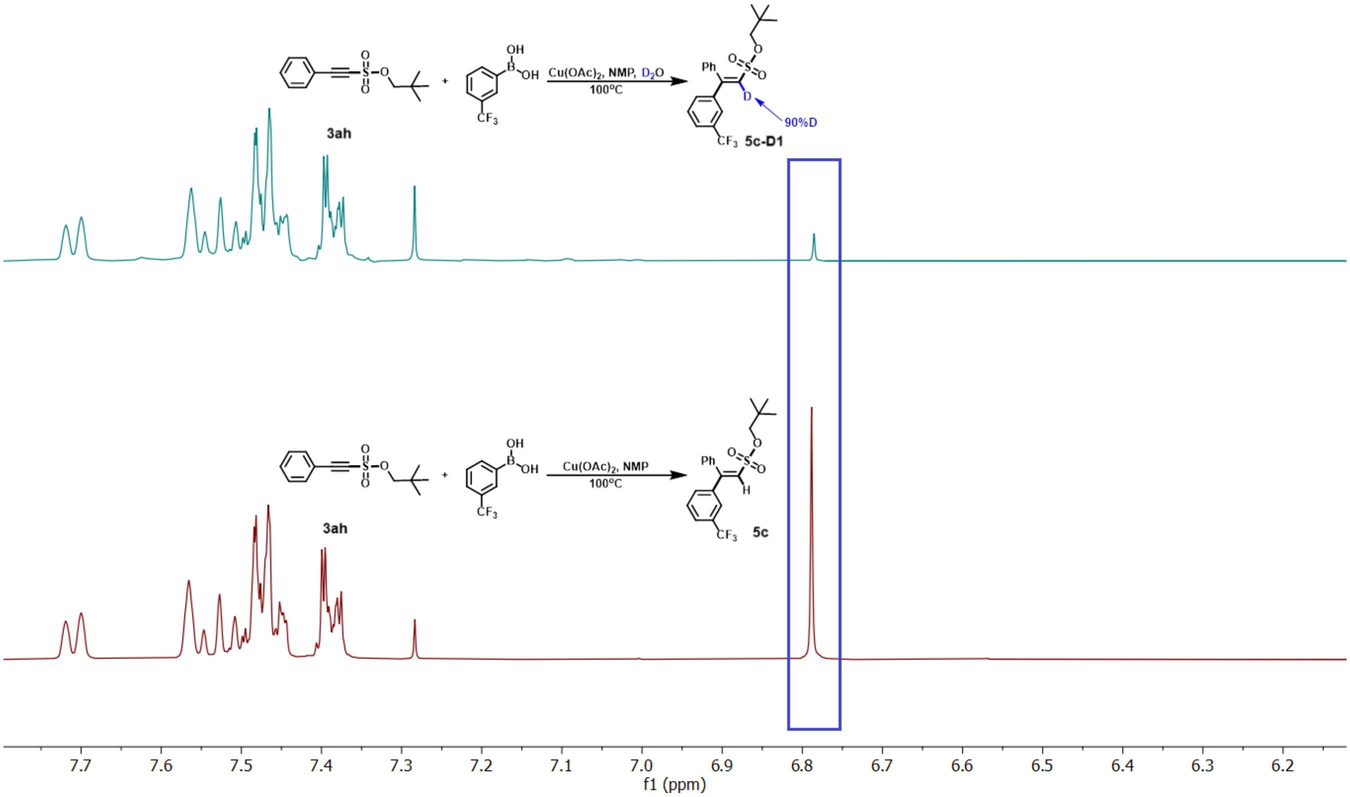

Figure S5: Illustrating the roles of water as a proton source

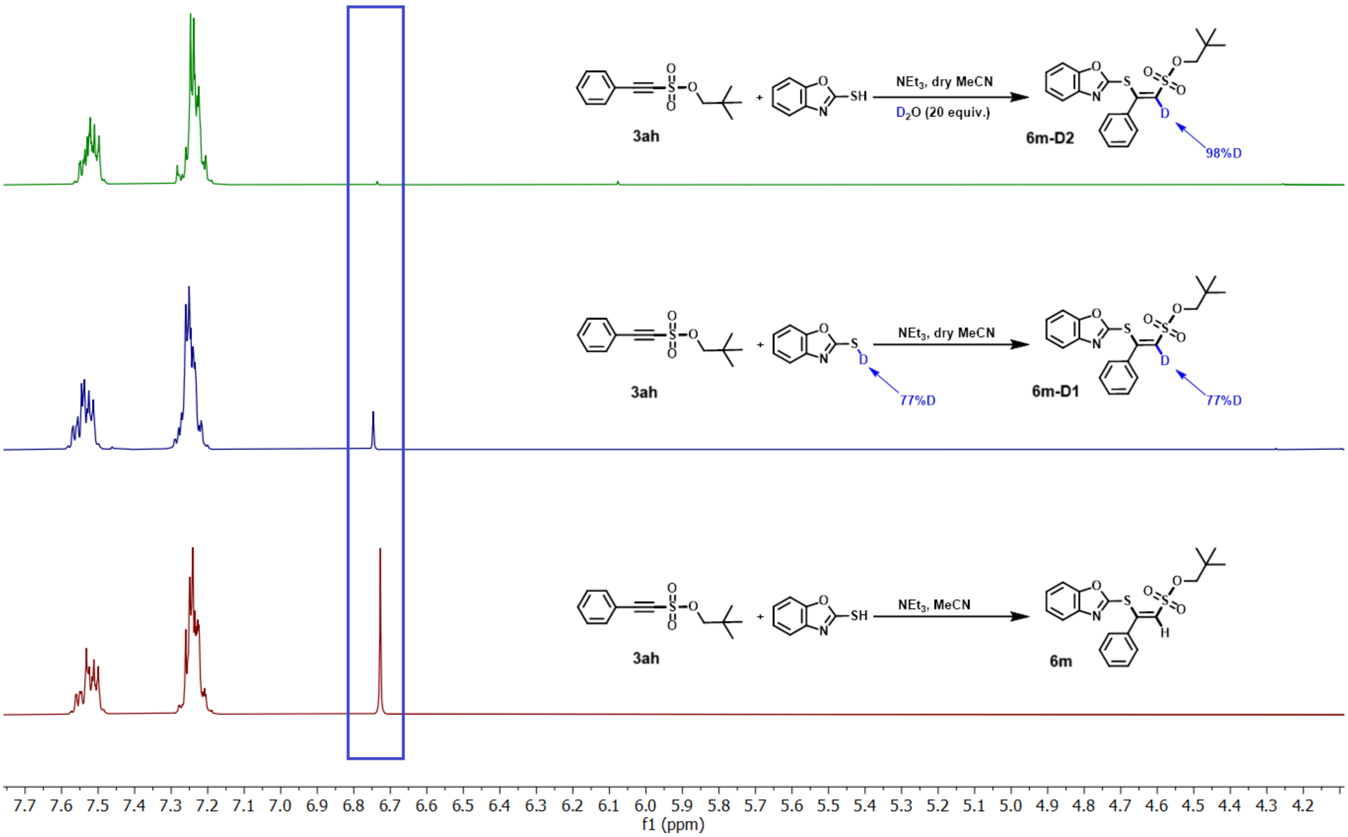

Figure S6: Illustrating the roles of nucleophile as a proton source

In the synthesis of 1,3-butadienes (Figure S4), performing the reaction in CD_3OD led to the formation of the corresponding deuterated product (**4l-D1**) with 78% deuterium incorporation. In a separate experiment using deuterated boronic acid in CD_3OD, 96% deuterium incorporation was observed in the 1,3-diene product (**4l-D2**). Similarly, in the synthesis of highly substituted alkenes (Figure S5), the use of D_2O (10 equiv.) in NMP resulted in 90% deuterium incorporation in the alkene product (**5c-D1**). The lower level of deuterium incorporation in **4l-D1** may be attributed to H–D exchange between MeOD and the organoboronic acid or direct proton transfer from the organoboronic acid to an copper intermediate . In the synthesis of highly substituted alkenes, water serves as the proton (or deuteron) source.

In nuclophilic modification, using benzoxazole-2-thiol-d (≈ 77% D) as nucleophile (Figure S6) led to the same level of deuterium incorporation in the product (**6m-D1**). On the other hand, employing D_2O in the reaction resulted in the formation of a product with 98% deuterium incorporation (**6m-D2**), suggesting that in the absence of water, the nucleophile can act as the proton (or deuteron) source.

Mechanistic Proposal for Copper-Catalyzed Reactions

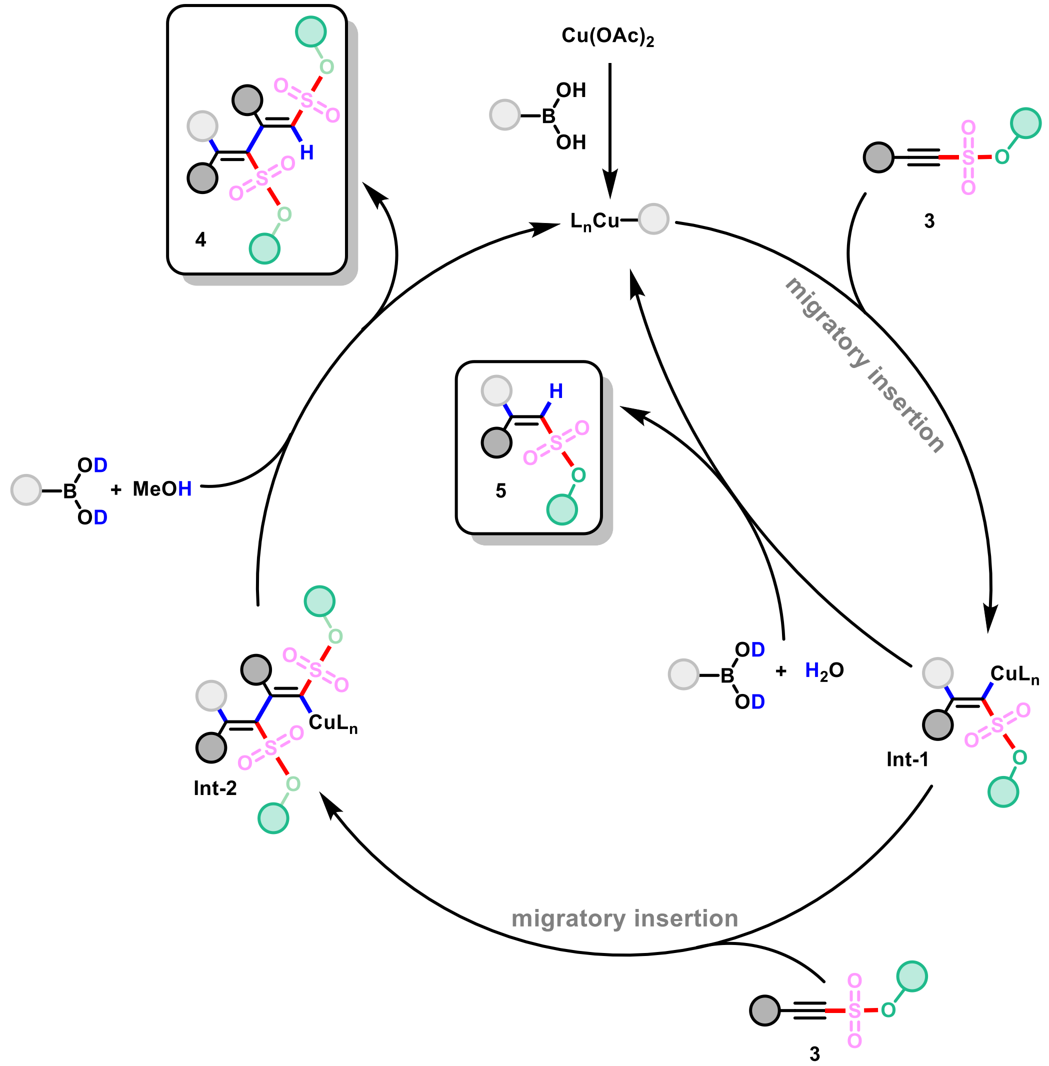

Scheme S2: proposed mechanism for nucleophilic addition to alkyne sulfonate

Scheme S2 presents a plausible mechanism for the Cu-catalyzed addition of arylboronic acids to alkyne sulfonates. Transmetallation of the arylboronic acid with the copper catalyst forms a reactive arylcopper species. [4] This intermediate undergoes *syn* carbocupration of the alkyne (**3**) to give vinylcopper intermediate **Int-1**, [5] which can then undergo protonolysis by methanol to afford the highly substituted alkene (**5**). Alternatively, a second migratory insertion generates vinylcopper intermediate **Int-2**, followed by protonolysis with methanol to produce the 1,3-butadiene (**4**).

*Note 1: Not surprisingly, when two different alkyne sulfonates were combined in a single reaction, a regioisomeric products was obtained (Figure S7, **4w1–4w4** in 73% yield and **4x1–4x4** in 60% yield). The regioisomers were difficult to separate into individual isomers using column chromatography, and they are represented as a mixture of regio-isomers.*

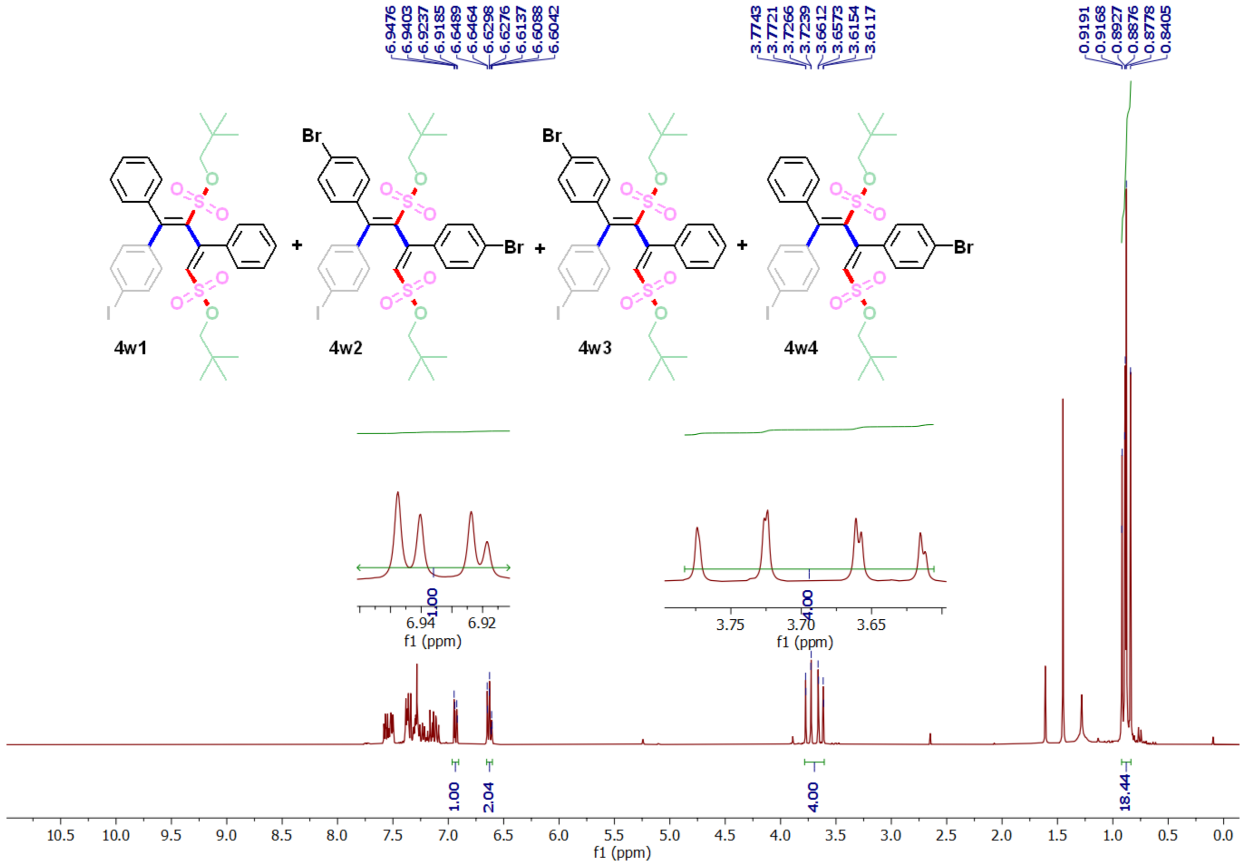

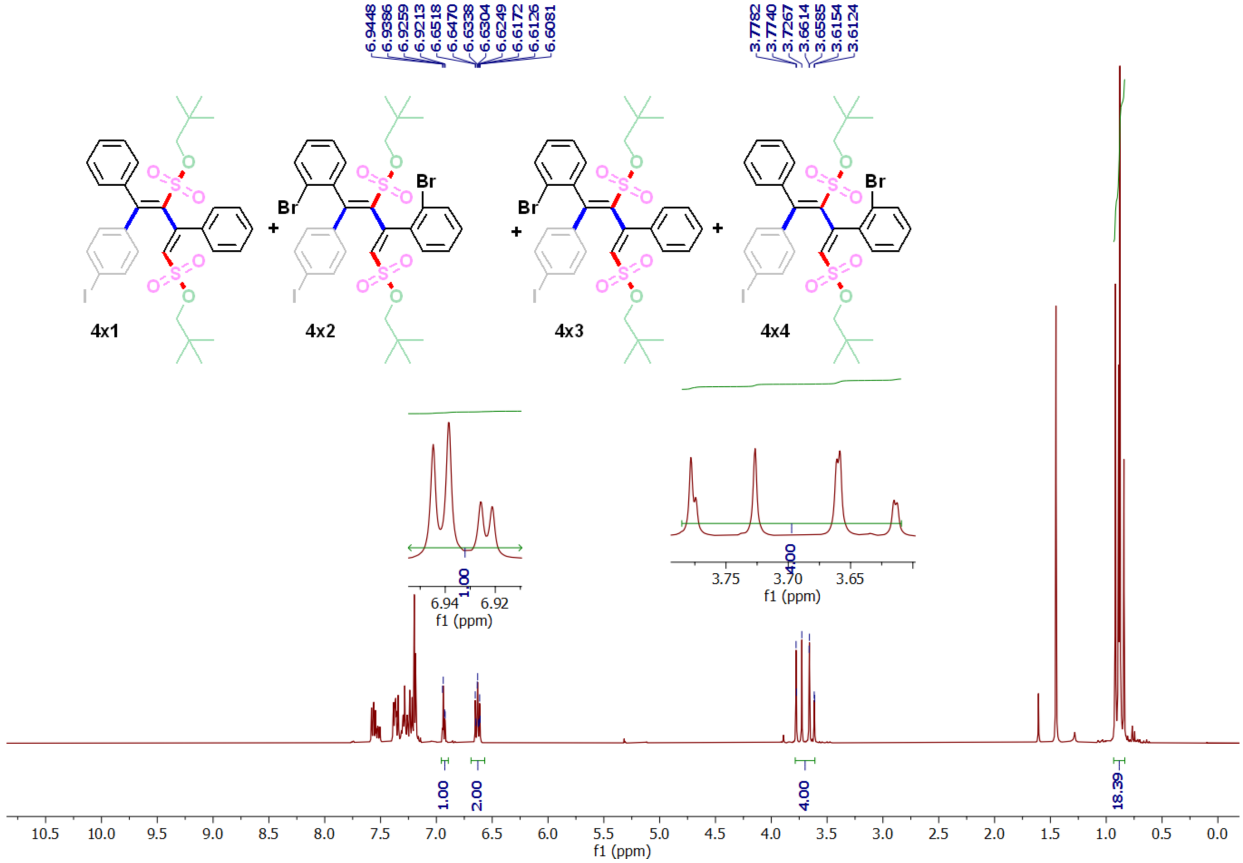

Figure S7: formation of regioisomeric products from mixed alkyne sulfonates

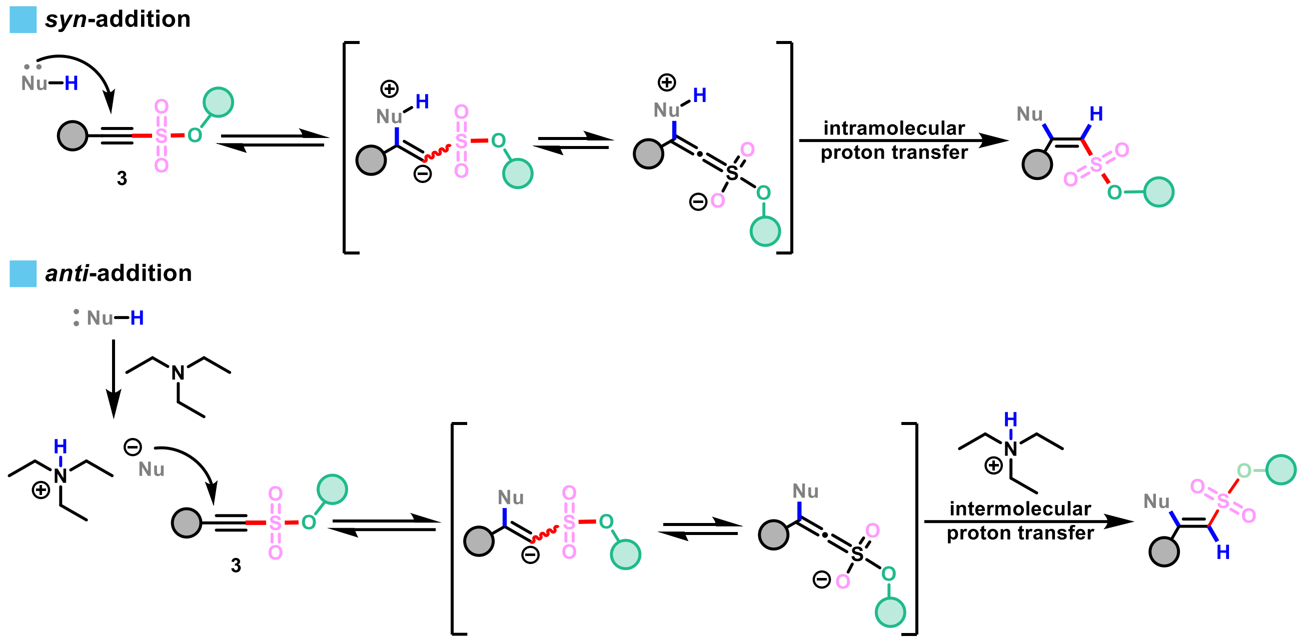

Scheme S3: proposed mechanism for nucleophilic addition to alkyne sulfonate

General Procedures

General Procedure A: electrochemical synthesis of alkyne sulfonates and sulfonamides

An oven-dried 10 mL Electrasyn 2.0 vial equipped with a stir bar, was charged with alkyne (0.6 mmol, 1.0 equiv.) and alcohol (2.7 mmol, 4.5 equiv.) if they was solid, then 6 mL dry DCM and alkyne (0.6 mmol, 1.0 equiv.) and alcohol (2.7 mmol, 4.5 equiv.) or amine (2.7 mmol, 4.5 equiv.) if they was liquid, then DMIPA (234 µL, 167 mg, 3.2 equiv.) and SO_2 in DMSO (0.8 mL, 6.0 M, 8 equiv.) were added. The vial was then connected to an IKA ElectraSyn 2.0 stir plate, and electrolysis was carried out at 15 mA (2.7 cm × 0.8 cm × 0.02 cm was fully submerged into the solution, 2.16 cm^2, 6.95 mA/cm^2), 0.6 mmol, 315 min. Upon completion of the programmed electrolysis, the solution was transferred to a round-bottom flask and electrods were rinsed with DCM (30 ml). The reaction mixture concentrated under reduced pressure. then the crude product was filtered over a short layer of silica (ca. 3-15 cm) and washed with cyclohexane/ethyl acetate 97/2 - 80/20 (based on polarity of the product).

Note 1: The small plastic joint of the Electrasyn 2.0 cap was removed, as the reaction requires an outlet for the hydrogen produced at the cathode to escape. The setup shown in Figure S8 also has an outlet,

Note 2: A quick Work-up with water can be performed for sulfonamides and sulfonates derived from primary alcohols. However, work-up is not recommended for alkyne sulfonates obtained from secondary alcohols.

Note 3: It is recommended not to leave the product on silica gel for extended periods. Chromatographic separation should be performed in <1 h to ensure the best recovery of the product.

Note 4: For column chromatography, wet loading using mixture of ethyl acetate: n-Heptane (based on solubility of crude) is recommended.

Note 5: The observed voltage typically begins in the range of 12–14 V and decreases to approximately 5–7 V by the end of the reaction time. It is important to note that these values are not absolute and may vary depending on the equipment setup and the nature of the precursor materials used. Such fluctuations are expected and should not be interpreted as indicators of reaction failure.

Note 6: For HRMS analysis of this class of compounds, the use of ammonium acetate buffer (5 mM) is recommended.

*Note 7: All compounds were stable at 4 °C except for compounds **3aw**, **3ax**, and **3ay**, for which decomposition was observed.*

Reaction Setups and Graphical Guide for the electrochemical synthesis

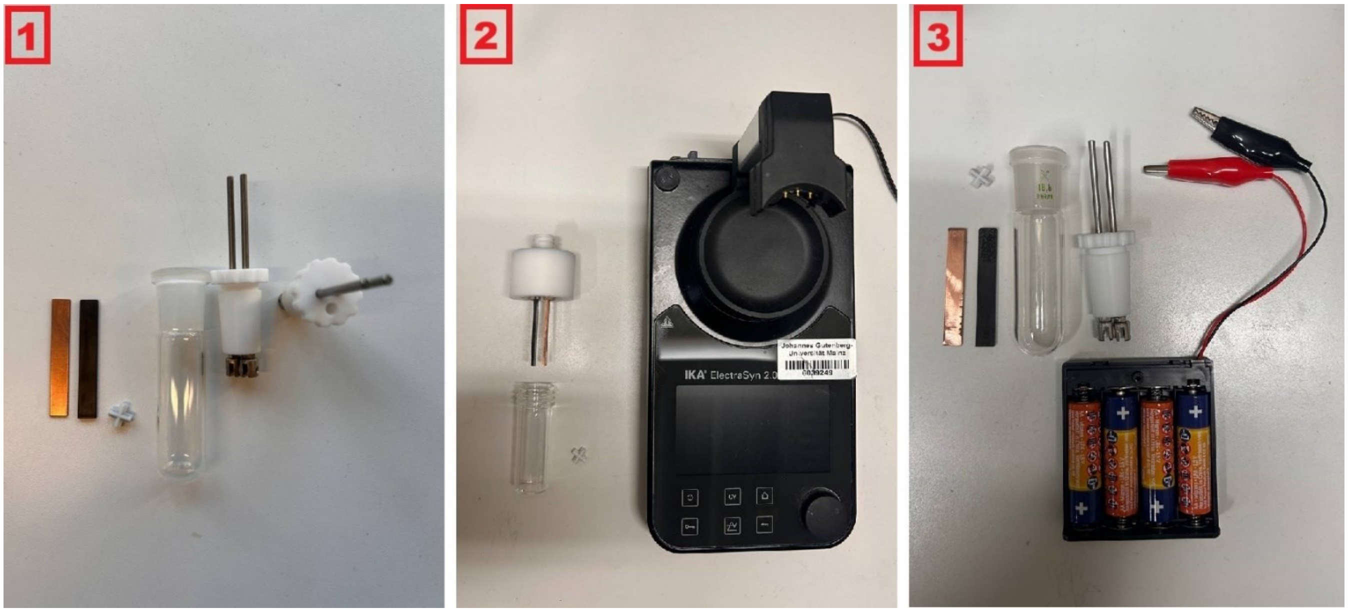

Figure S8: Electrochemical reaction setups.

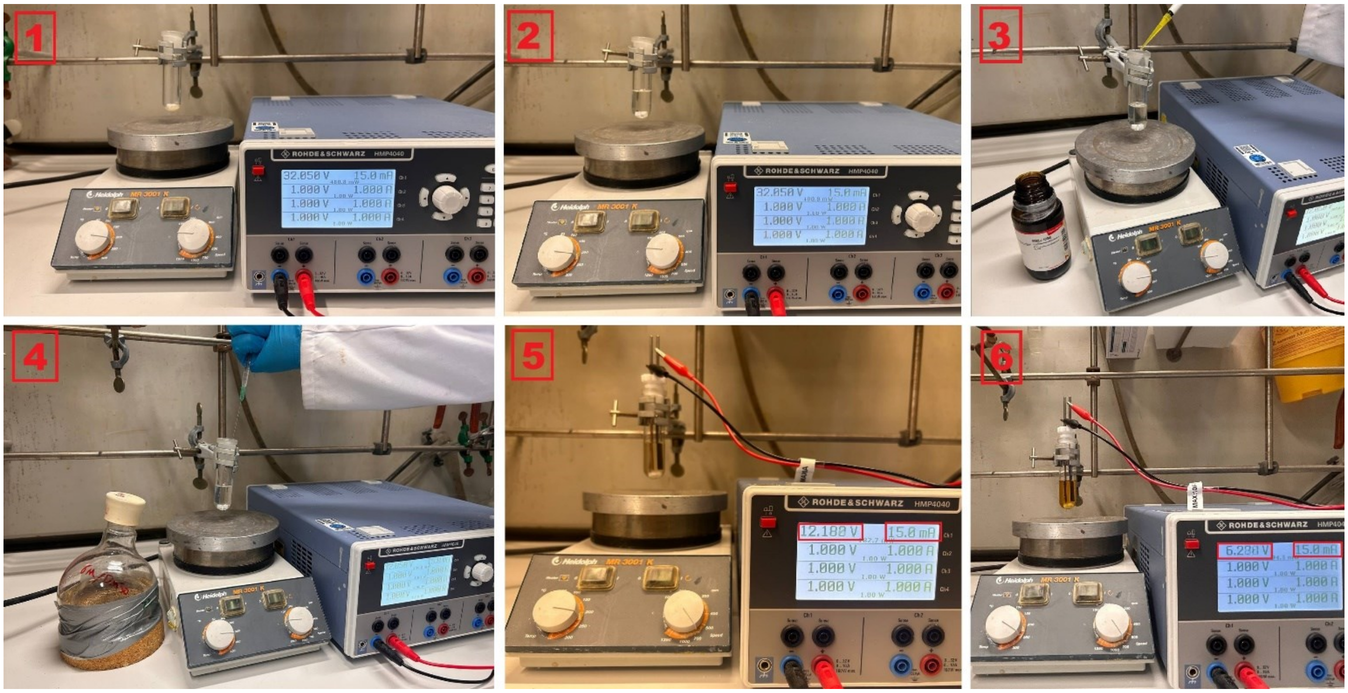

Figure S9: **1)** A test tube was charged with the alkyne and the alcohol (if solid). **2)** 6 mL of DCM was added to the mixture. **3)** DMIPA was added to the reaction mixture. **4)** SO_2 was added to the system. **5)** Electrodes were inserted, the current was adjusted, and the reaction was started. **6)** End of reaction time.

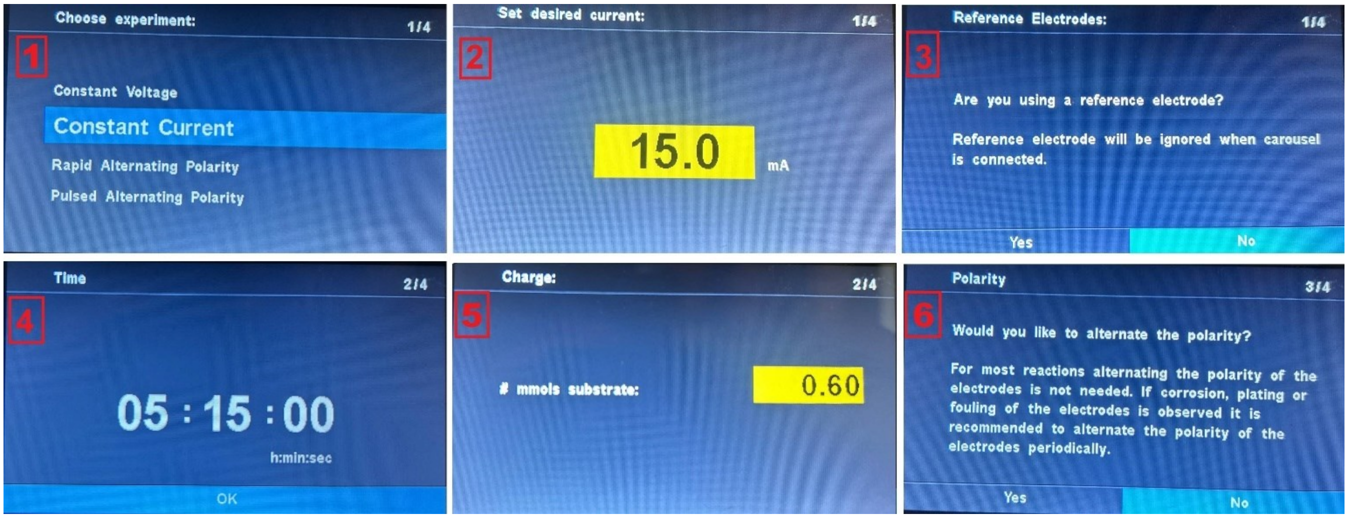

Figure S10: IKA Electrasyn 2.0 settings

Protocol for the Scale-up Reaction

The scale-up experiment was performed in a jacketed undivided glass cell with a volume of ca. 125 mL equipped with a PTFE stopper and sleeve, electrodes, and electrode holders, and a magnetic stir bar embedded in a PTFE ring (The cells are commercially available by Sigma-Aldrich within the SynLectro™ series). The electrolysis was conducted using a TDK-Lambda Z+ series (TDK-Lambda UK Limited, Devon, United Kingdom) as power source. The electrode dimensions were 60 mm × 20 mm × 3 mm, with 50 mm submerged into the reaction mixture, resulting in an active electrode area of 10.0 cm^2. The distance between the electrodes was 12 mm. The graphite employed was Sigrafine™ V2100 by SGL Carbon/Bad Godesberg, Germany. The stainless-steel cathode was made from V2A steel.

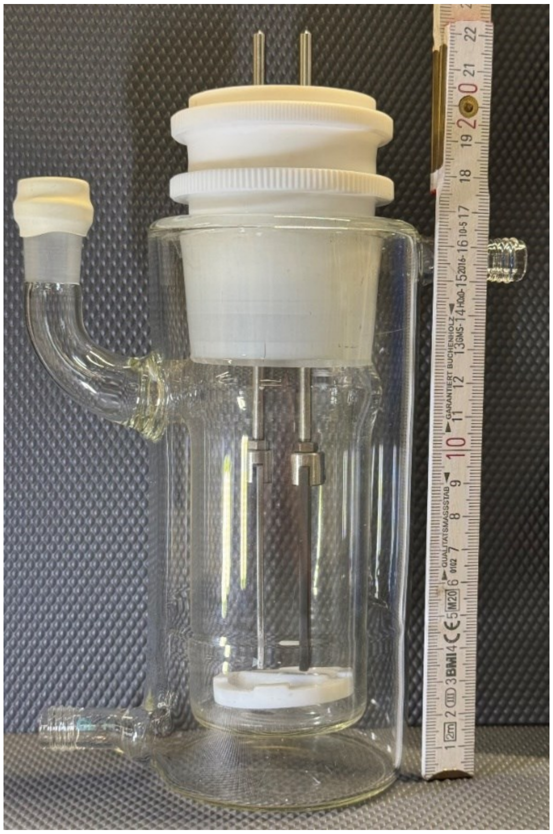
Figure S11: Jacketed electrochemical glass cell with 125 mL used for the scale-up experiment equipped with graphite and stainless-steel electrodes, PTFE plug and electrode holders.

The electrolysis cell was charged with neopentyl alcohol (3.97 g, 45.0 mmol, 4.5 eq.), phenylpropiolic acid (1.46 g, 10.0 mmol, 1.0 eq.), dichloromethane (dry, 100 mL), DMIPA (2.79 g, 3.87 mL, 32.0 mmol, 3.2 equiv.) and SO_2 stock solution in dry DMSO (5.85 M, 13.7 mL, 8.0 equiv), so that a total volume of ca. 120 mL was reached. The cell was equipped with a graphite anode and a stainless-steel cathode and connected to the power source. The current was set accordingly so that a current density of 69.4 mA (5 cm × 2 cm × 0.03 cm was fully submerged into the solution, 10 cm^2, 6.94 mA/cm^2). The electrolysis was conducted under constant stirring (400 rpm) for 18 h 39 min ($Q_{applied}$ = 4666 C, $Q_{Faraday}$ = 4.84 F) at 20 °C with active temperature control through a cryostat. After completion of the electrolysis, the reaction mixture was transferred into a separatory funnel and washed with water (3 x 100 mL). The combined watery phases were extracted with dichloromethane (2 x 100 mL) and the combined organic phases were dried over $MgSO_4$. The crude was subjected to automated column chromatography (cyclohexane/ethyl acetate = 99/1 → 85/15, column: PF-25SiHC-F0120, detection @ 254 nm). The desired product neopentyl 2-phenylethyne-1-sulfonate (**3ah**, 1.41 g, 56%) was obtained as an off-white liquid.

Note 1: Spectroscopic data of the isolated compound matched the one obtained from the small-scale reaction described in *Compounds Characterization Data*.

Note 2: An ^1H NMR yield of 62% was calculated by addition of ethylene carbonate (150.6 mg, 1.71 mmol) as internal standard to the reaction mixture after the electrolysis.

General Procedure B: synthesis of 1,3-butadienes via alkyne dimerization and arylation

A 10 mL reaction tube was charged with alkyne sulfonate (0.1 mmol, 1.0 equiv.), boronic acid (0.3 mmol, 3.0 equiv.), $Cu(OAc)_2$ (1.8 mg), MeOH (0.5 mL) and a magnetic stir bar. Subsequently, the reaction mixture was stirred for 12 h at room temperature. Once the time has passed, the reaction mixture was diluted with EtOAc and washed with H_2O (2 x 25 mL), brine (2 x 25 mL), dried over anhydrous Na_2SO_4 and concentrated under reduced pressure. Purification by flash column chromatography (EtOAc/cyclohexane) provided the desired product.

*Note 1: Due to the insolubility of (E)-(2-((methoxyimino)methyl)phenyl)boronic acid in MeOH, (0.1 mL) DCM was used as the cosolvent for the synthesis of the **4p**.*

Note 2: Hydroarylated side products were consistently formed in 5–20% yield during the synthesis of 1,3-butadiene derivatives.

General Procedure C: synthesis of highly substituted alkenes via hydroarylation

A 10 mL reaction tube was charged with alkyne sulfonate (0.1 mmol, 1.0 equiv.), boronic acid (0.3 mmol, 3.0 equiv.), $Cu(OAc)_2$ (1.8 mg), NMP (0.5 mL) and a magnetic stir bar. Subsequently, the reaction mixture was stirred for 2 h at 100 °C. Once the time has passed, the reaction mixture was diluted with EtOAc and washed with H_2O (2 x 25 mL), brine (2 x 25 mL), dried over anhydrous Na_2SO_4 and concentrated under reduced pressure. Purification by flash column chromatography (EtOAc/cyclohexane) provided the desired product.

Note 1: Under the procedure C, 1,3-butadienes were not observed as side products.

General Procedure D: nucleophilic modification

A 10 mL reaction tube was charged with alkyne sulfonate (0.1 mmol, 1.0 equiv.), nucleophile (0.1 mmol, 1.0 equiv.), MeCN (1 mL), Et_3N (20 µL, 23 mg, 1.4 mmol, 1.4 equiv.) and a magnetic stir bar. Subsequently, the reaction mixture was stirred for 20 min (for hydrothiolation) or 12 h (for hydroamination, hydroalkoxylation, hydrocarboxylation) at room temperature, progress of the reaction controlled by TLC. Once the time has passed, the reaction mixture was diluted with EtOAc and washed with H_2O (2 x 25 mL), brine (2 x 25 mL), dried over anhydrous Na_2SO_4 and concentrated under reduced pressure. Purification by flash column chromatography (EtOAc/cyclohexane) provided the desired product.

*Note 1: Due to the insolubility of D-(+)-biotin in MeCN, DMF was used as the solvent for the synthesis of the **6s**.*

*Note 2: Due to the insolubility of (5α -cholestan-3β-yl) 2-phenylethyne-1-sulfonate (**3azb**) in MeCN, a DCM/MeCN (1/1) mixture was used as the solvent for the synthesis of **6u**, and a DMF/MeCN (1/1) mixture was used for the synthesis of **6v**.*

*Note 3: The hydrothiolation products (**6j**-**6m**) do not require column chromatography; pure compounds were obtained directly after the aqueous work-up, without the need for further purification.*

*Note 4: In the synthesis of compounds **6f**, **6g**, and **6o**, the use of triethylamine as a base is not required.*

X-ray Structure of BP1

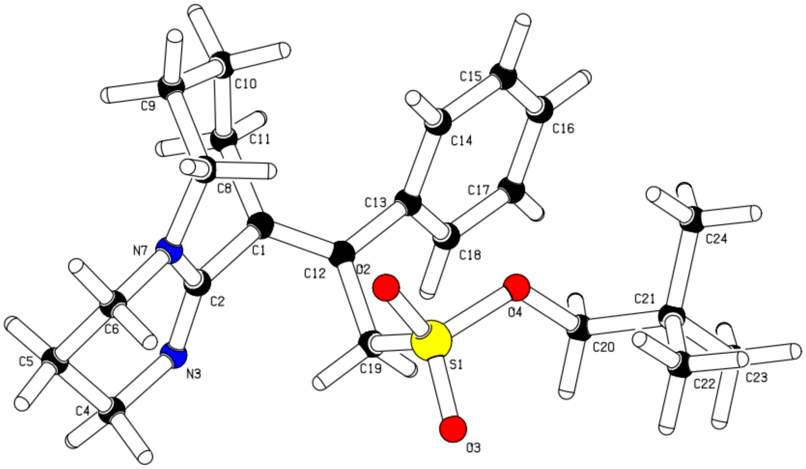

Deposition number (CCDC): 2464885

Empirical formula	$C_{22}H_{32}N_2O_3S$	
Formula weight	404.55	
Temperature	120(2) K	
Wavelength, radiation type	0.71073Å, MoKα	
Crystal system	Monoclinic	
Space group name, number	P 2_1/c, (14)	
Unit cell dimensions	a = 11.7336(4) Å	α = 90°
	b = 6.24360(10)Å	β = 91.691(3)°
	c = 28.5842(10) Å	γ = 90°
Volume	2093.16(11) $Å^3$	
Z	4	
Density (calculated)	1.284 Mg/m^3	
Absorption coefficient	0.180 mm^{-1}	
F(000)	872	
Crystal size	0.090 x 0.230 x 0.520 mm3	
Theta range for data collection	2.715 to 27.922°	
Index ranges	-15<=h<=15, -7<=k<=8, -37<=l<=30	
Number of reflections:		
Collected	11054	

independent	4972 [R(int) = 0.0297]
observed [I>2sigma(I)]	4432
Completeness to theta = 25.2°	99.7 %
Refinement method	Full-matrix least-squares on F^2
Data / restraints / parameters	4972 / 0 / 256
Goodness-of-fit on F^2	1.049
Final R indices [I>2sigma(I)]	R1 = 0.0385, wR2 = 0.1003
R indices (all data)	R1 = 0.0437, wR2 = 0.1058
Largest diff. peak and hole	0.323 and -0.398 eÅ^{-3}

X-ray Structure of 4k

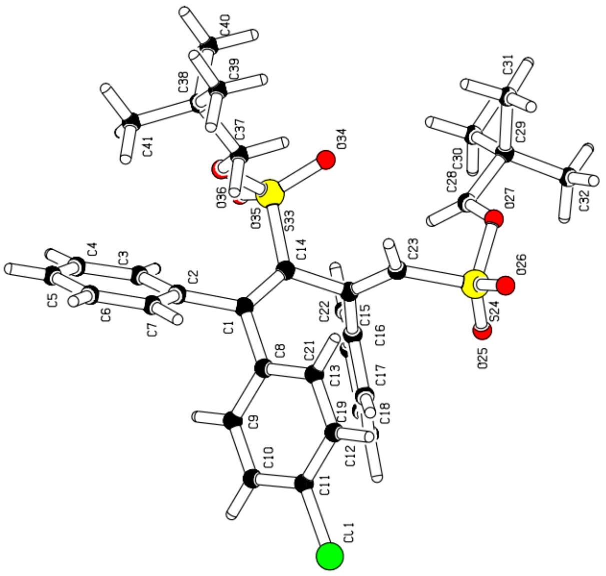

Deposition number (CCDC): 2464884

Empirical formula	$C_{32}H_{37}ClO_6S_2$	
Formula weight	617.18	
Temperature	120(2) K	
Wavelength, radiation type	0.71073Å, MoKα	
Crystal system	Monoclinic	
Space group name, number	P 2_1/c, (14)	
Unit cell dimensions	a = 11.7210(5) Å	α = 90°
	b = 15.2438(5) Å	β = 103.125(3)°
	c = 18.2053(8) Å	γ = 90°

Volume	3167.8(2) $Å^3$
Z	4
Density (calculated)	1.294 Mg/m^3
Absorption coefficient	0.294 mm^{-1}
F(000)	1304
Crystal size	0.210 x 0.400 x 0.500 mm^3
Theta range for data collection	2.658 to 27.916°.
Index ranges	-15<=h<=15, -18<=k<=20, -23<=l<=20
Number of reflections:	
Collected	15665
independent	7520 [R(int) = 0.0221]
observed [I>2sigma(I)]	6332
Completeness to theta = 25.2°	99.8 %
Refinement method	Full-matrix least-squares on F^2
Data / restraints / parameters	7520 / 0 / 433
Goodness-of-fit on F^2	1.050
Final R indices [I>2sigma(I)]	R1 = 0.0413, wR2 = 0.0921
R indices (all data)	R1 = 0.0530, wR2 = 0.0990
Largest diff. peak and hole	0.520 and -0.385 $eÅ^{-3}$

Compounds Characterization Data

Neopentyl (*Z*)-2-(3,4,6,7,8,9-hexahydropyrimido[1,2-a]azepin-10(2*H*)-ylidene)-2-phenylethane-1-sulfonate (BP1)

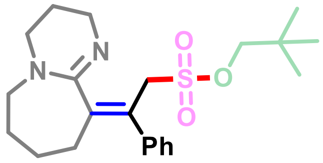

A 10 mL reaction tube was charged with 2-phenylethyne-1-sulfonate (**3ah**) (50.4 mg, 0.2 mmol), DBU (33 µL,335 mg, 0.22 mmol, 1.1 equiv.), DCM (0.5 mL) and a magnetic stir bar. Subsequently, the reaction mixture was stirred for 1 min at room temperature. Once the time has passed, the reaction mixture was diluted with Diethyl ether(1 mL), then the mixture was placed in the freezer. After 12 hours, the formed crystals were washed with a small amount of pentane to afford the final product as a pure colorless crystals. Yield 99% (80 mg).

NMR Spectroscopy (see spectra):
^{1}H NMR (300 MHz, $CDCl_3$): δ 7.40 – 7.25 (m, 5H), 3.84 – 2.84 (m, 9H), 2.49 – 1.42 (m, 9H), 0.79 (s, 9H).
^{13}C NMR (75 MHz, $CDCl_3$): δ 159.06, 144.57, 139.33, 128.51, 128.34, 127.90, 127.50, 78.94, 55.02, 51.61, 47.28, 44.09, 31.82, 31.49, 29.41, 27.04, 25.92, 22.28.

HRMS (ESI^{+}): m/z calc'd for **BP1** $C_{22}H_{33}N_2O_3S$ $[M+H]^+$: 405.2212, found: 405.2219.
mp: 114.5 – 116.7 °C.

Dineopentyl sulfite (BP2)

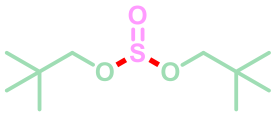

Purification via column chromatography on silica gel (cyclohexane: ethyl acetate = 95:5, v/v) afforded **BP2** as a colorless oil; Yield 15% (90 mg).

NMR Spectroscopy (see spectra):
^{1}H NMR (300 MHz, $CDCl_3$): δ 3.70 (d, J = 9.6 Hz, 2H), 3.53 (d, J = 9.6 Hz, 2H), 0.95 (s, 18H).
^{13}C NMR (75 MHz, $CDCl_3$): δ 71.24, 31.65, 26.34.

bis(4-phenylbutyl) sulfite (BP3)

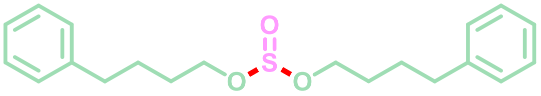

Purification via column chromatography on silica gel (cyclohexane: ethyl acetate = 95:5, v/v) afforded **BP3** as a colorless oil; Yield 13% (122 mg).

NMR Spectroscopy (see spectra):
^{1}H NMR (300 MHz, $CDCl_3$): δ 7.38 – 7.28 (m, 4H), 7.26 – 7.14 (m, 6H), 4.13 – 3.89 (m, 4H), 2.71 – 2.64 (m, 4H), 1.81 – 1.69 (m, 8H).
^{13}C NMR (75 MHz, $CDCl_3$): δ 141.84, 128.42, 128.41, 125.93, 62.09, 35.34, 29.05, 27.55.

Methyl 2-phenylethyne-1-sulfonate (3aa)

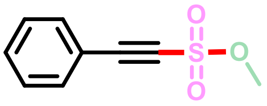

Following the general procedure A. 3-phenylpropiolic acid (88 mg, 0.6 mmol), methanol (109 µL, 87 mg, 2.7 mmol, 4.5 equiv.), SO_2 in DMSO (0.8 mL) and DCM (6 mL) were used. Purification via column chromatography on silica gel (cyclohexane: ethyl acetate = 98:2, v/v) afforded **3aa** as a colorless oil; Yield 38% (45 mg).

NMR Spectroscopy (see spectra):
^{1}H NMR (400 MHz, $CDCl_3$): δ 7.62 – 7.66 (m, 2H), 7.57 (tt, J = 7.5, 2.1 HZ, 1H), 7.43 – 2.48 (m, 2H), 4.09 (s, 3H).
^{13}C NMR (100 MHz, $CDCl_3$): δ 133.01, 131.96, 128.88, 117.24, 91.32, 78.87, 58.07.

HRMS (ESI^{+}): m/z calc'd for **3aa** $C_9H_{12}NO_3S$ $[M+NH_4]^+$: 214.0532, found: 214.0531.

Ethyl 2-phenylethyne-1-sulfonate (3ab)

Following the general procedure A. 3-phenylpropiolic acid (88 mg, 0.6 mmol), Ethanol (158 μL, 124 mg, 2.7 mmol, 4.5 equiv.), SO_2 in DMSO (0.8 mL) and DCM (6 mL) were used. Purification via column chromatography on silica gel (cyclohexane: ethyl acetate = 98:2, v/v) afforded **3ab** as a colorless oil; Yield 59% (74 mg).

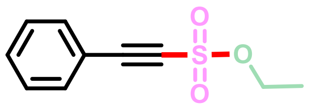

NMR Spectroscopy (see spectra):
^{1}H NMR (400 MHz, $CDCl_3$): δ 7.58 – 7.64 (m, 2H), 7.55 (tt, *J* = 6.7, 1.5 HZ, 1H), 7.41 – 7.47 (m, 2H), 4.48 (q, *J* = 7.1 HZ, 2H), 1.51 (t, *J* = 7.1 HZ, 3H).
^{13}C NMR (100 MHz, $CDCl_3$): δ 132.92, 131.90, 128.89, 117.32, 90.66, 79.88, 69.51, 14.61.

HRMS (ESI^+): m/z calc'd for **3ab** $C_{10}H_{14}NO_3S$ $[M+NH_4]^+$: 228.0678, found: 228.0689.

Propyl 2-phenylethyne-1-sulfonate (3ac)

Following the general procedure A. 3-phenylpropiolic acid (88 mg, 0.6 mmol), 1-propanol (202 μL, 162 mg, 2.7 mmol, 4.5 equiv.), SO_2 in DMSO (0.8 mL), and DCM (6 mL) were used. Purification via column chromatography on silica gel (cyclohexane: ethyl acetate = 98:2, v/v) afforded **3ac** as a colorless oil; Yield 59% (80 mg).

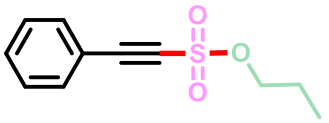

NMR Spectroscopy (see spectra):
^{1}H NMR (400 MHz, $CDCl_3$): δ 7.58 – 7.67 (m, 2H), 7.55 (tt, *J* = 7.5, 1.5 HZ, 1H), 7.41 – 7.48 (m, 2H), 4.37 (t, *J* = 6.5 HZ, 2H), 1.82 – 1.95 (m, 2H), 1.07 (t, *J* = 7.4 HZ, 3H).
^{13}C NMR (75 MHz, $CDCl_3$): δ 132.93, 131.85, 128.87, 117.41, 90.63, 79.84, 74.73, 22.23, 10.07.

HRMS (ESI^+): m/z calc'd for **3ac** $C_{11}H_{16}NO_3S$ $[M+NH_4]^+$: 242.0845, found: 242.0839.

Pentyl 2-phenylethyne-1-sulfonate (3ad)

Following the general procedure A. 3-phenylpropiolic acid (88 mg, 0.6 mmol), 1-pentanol (292 μL, 238 mg, 2.7 mmol, 4.5 equiv.), SO_2 in DMSO (0.8 mL), and DCM (6 mL) were used. Purification via column chromatography on silica gel (cyclohexane: ethyl acetate = 98:2, v/v) afforded **3ad** as a colorless oil; Yield 62% (94 mg).

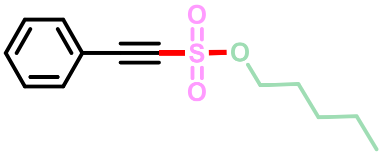

NMR Spectroscopy (see spectra):
^{1}H NMR (400 MHz, $CDCl_3$): δ 7.58 – 7.65 (m, 2H), 7.55 (tt, *J* = 7.3, 1.3 HZ, 1H), 7.41 – 7.48 (m, 2H), 4.41 (t, *J* = 6.5 HZ, 2H), 1.80 – 1.90 (m, 2H), 1.34-1.50 (m, 4H), 0.93 (t, *J* = 7.2 HZ, 3H).
^{13}C NMR (75 MHz, $CDCl_3$): δ 132.91, 131.84, 128.87, 117.41, 90.60, 79.86, 73.32, 28.37, 27.51, 22.08, 13.86.

HRMS (ESI^+): m/z calc'd for **3ad** $C_{13}H_{16}O_3SNa$ $[M+Na]^+$: 275.0712, found: 275.0711.

Pentadecyl 2-phenylethyne-1-sulfonate (3ae)

Following the general procedure A. 3-phenylpropiolic acid (88 mg, 0.6 mmol), pentadecan-1-ol (617 mg, 2.7 mmol, 4.5 equiv.), SO_2 in DMSO (0.8 mL), and DCM (6 mL) were used. Purification via column chromatography on silica gel (cyclohexane: ethyl acetate = 98:2, v/v) afforded **3ae** as a colorless solid; Yield 57% (140 mg).

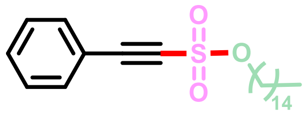

NMR Spectroscopy (see spectra):

^{1}**H NMR** (300 MHz, $CDCl_3$): δ 7.68 – 7.59 (m, 2H), 7.59 – 7.50 (m, 1H), 7.49 – 7.41 (m, 2H), 4.41 (t, *J* = 6.5 Hz, 2H), 1.90 – 1.79 (m, 2H), 1.52 – 1.41 (m, 2H), 1.39 – 1.23 (m, 24H), 0.94 – 0.86 (m, 3H).
^{13}**C NMR** (75 MHz, $CDCl_3$): δ 132.92, 131.82, 128.86, 117.44, 90.57, 79.89, 73.33, 31.95, 29.71, 29.67, 29.63, 29.54, 29.44, 29.38, 29.01, 28.69, 25.43, 22.72, 14.15.

HRMS (ESI^+): m/z calc'd for **3ae** $C_{23}H_{36}O_3S$ $[M+NH_4]^+$: 410.2723, found: 410.2712.
mp: 42.6 – 43.9 °C.

Isobutyl 2-phenylethyne-1-sulfonate (3af)

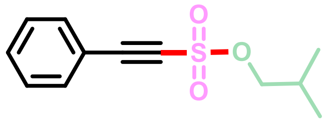

Following the general procedure A. 3-phenylpropiolic acid (88 mg, 0.6 mmol), 2-methyl-1-propanol (250 µL, 200 mg, 2.7 mmol, 4.5 equiv.), SO_2 in DMSO (0.8 mL), and DCM (6 mL) were used. Purification via column chromatography on silica gel (cyclohexane: ethyl acetate = 98:2, v/v) afforded **3af** as a colorless oil; Yield 65% (93 mg).

NMR Spectroscopy (see spectra):
^{1}**H NMR** (300 MHz, $CDCl_3$): δ 7.64 – 7.60 (m, 2H), 7.59 – 7.52 (m, 1H), 7.49 – 7.41 (m, 2H), 4.18 (d, *J* = 6.5 Hz, 2H), 2.16 (hept, *J* = 6.7 Hz, 1H), 1.06 (d, *J* = 6.7 Hz, 6H).
^{13}**C NMR** (75 MHz, $CDCl_3$): δ 132.92, 131.83, 128.87, 117.43, 90.64, 79.79, 78.76, 28.00, 18.66.

HRMS (ESI^+): m/z calc'd for **3af** $C_{12}H_{18}NO_3S$ $[M+Na]^+$: 256.0998, found: 256.1002.

4-Methylpentyl 2-phenylethyne-1-sulfonate (3ag)

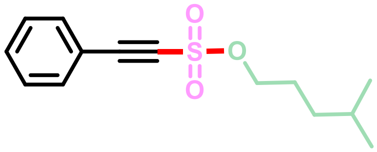

Following the general procedure A. 3-phenylpropiolic acid (88 mg, 0.6 mmol), isohexanol (339 µL, 276 mg, 2.7 mmol, 4.5 equiv.), SO_2 in DMSO (0.8 mL), and DCM (6 mL) were used. Purification via column chromatography on silica gel (cyclohexane: ethyl acetate = 98:2, v/v) afforded **3ag** as a colorless oil; Yield 71% (114 mg).

NMR Spectroscopy (see spectra):
^{1}**H NMR** (300 MHz, $CDCl_3$): δ 7.66 – 7.59 (m, 2H), 7.59 – 7.52 (m, 1H), 7.48 – 7.41 (m, 2H), 4.40 (t, *J* = 6.5 Hz, 2H), 1.91 – 1.79 (m, 2H), 1.69 – 1.54 (m, 1H), 1.40 – 1.30 (m, 2H), 0.92 (d, *J* = 6.6 Hz, 7H).
^{13}**C NMR** (75 MHz, $CDCl_3$): δ 132.92, 131.86, 128.88, 117.40, 90.64, 79.86, 73.64, 34.43, 27.59, 26.65, 22.40.

HRMS (ESI^+): m/z calc'd for **3ag** $C_{14}H_{22}NO_3S$ $[M+NH_4]^+$: 284.1315, found: 284.1310.

Neopentyl 2-phenylethyne-1-sulfonate (3ah)

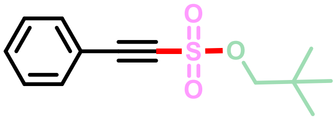

Following the general procedure A. 3-phenylpropiolic acid (88 mg, 0.6 mmol), 2,2-dimethyl-1-propanol (238 mg, 2.7 mmol, 4.5 equiv.), SO_2 in DMSO (0.8 mL), and DCM (6 mL) were used. Purification via column chromatography on silica gel (cyclohexane: ethyl acetate = 98:2, v/v) afforded **3ah** as a colorless oil; Yield 71% (108 mg).

Following the general procedure A. (bromethinyl)benzol (109 mg, 0.6 mmol), 2,2-dimethyl-1-propanol (238 mg, 2.7 mmol, 4.5 equiv.), SO_2 in DMSO (0.8 mL), and DCM (6 mL) were used. Purification via column chromatography on silica gel (cyclohexane: ethyl acetate = 98:2, v/v) afforded **3ah** as a colorless oil; Yield 67% (101 mg).

NMR Spectroscopy (see spectra):
^{1}**H NMR** (300 MHz, $CDCl_3$): δ 7.51 – 7.66 (m, 3H), 7.41 – 7.49 (m, 2H), 4.06 (s, 2H), 1.06 (s, 9H).

^{13}**C NMR** (75 MHz, $CDCl_3$): δ 132.91, 131.84, 128.88, 117.42, 90.69, 81.93, 79.70, 31.79, 26.07.

HRMS (ESI$^+$): m/z calc'd for **3ah** $C_{13}H_{16}O_3SNa$ $[M+Na]^+$: 275.0712, found: 275.0708.

(*S*)-2-Methylbutyl 2-phenylethyne-1-sulfonate (3ai)

Following the general procedure A. 3-phenylpropiolic acid (88 mg, 0.6 mmol), (*S*)-(−)-2-methyl-1-butanol (292 µL, 238 mg, 2.7 mmol, 4.5 equiv.), SO_2 in DMSO (0.8 mL), and DCM (6 mL) were used. Purification via column chromatography on silica gel (cyclohexane: ethyl acetate = 98:2, v/v) afforded **3ai** as a colorless oil; Yield 66% (100 mg).

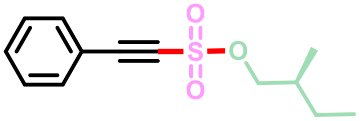

NMR Spectroscopy (see spectra):
^{1}**H NMR** (400 MHz, $CDCl_3$): δ 7.58 – 7.65 (m, 2H), 7.55 (tt, J = 7.6, 1.4 HZ, 1H), 7.41 – 7.49 (m, 2H), 4.19 – 4.30 (m, 2H), 1.85 – 1.98 (m, 1H), 1.49 – 1.61 (m, 1H), 1.25 – 1.37 (m, 1H), 1.05 (d, J = 6.8 HZ, 3H), 0.97 (t, J = 7.5 HZ, 3H).
^{13}**C NMR** (100 MHz, $CDCl_3$): δ 132.90, 131.83, 128.87, 117.42, 90.63, 79.80, 34.30, 25.46, 16.05, 11.05.

HRMS (ESI$^+$): m/z calc'd for **3ai** $C_{13}H_{20}NO_3S$ $[M+NH_4]^+$: 270.1158, found: 270.1155.
Specific rotation $[\alpha]_D^{25}$: +11.2 (c = 0.5, $CHCl_3$).

2-Fluoroethyl 2-phenylethyne-1-sulfonate (3aj)

Following the general procedure A. 3-phenylpropiolic acid (88 mg, 0.6 mmol), 2-fluoroethanol (157 µL, 173 mg, 2.7 mmol, 4.5 equiv.), SO_2 in DMSO (0.8 mL), and DCM (6 mL) were used. Purification via column chromatography on silica gel (cyclohexane: ethyl acetate = 98:3, v/v) afforded **3aj** as a colorless oil; Yield 77% (105 mg).

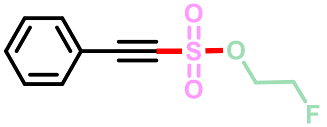

NMR Spectroscopy (see spectra):
^{1}**H NMR** (400 MHz, $CDCl_3$): δ 7.60 – 7.66 (m, 2H), 7.57 (tt, J = 7.6, 1.4 HZ, 1H), 7.42 – 7.49 (m, 2H), 4.81 – 4.86(m, 1H), 4.63 – 4.71 (m, 2H), 4.53 – 4.60 (m, 1H).
^{13}**C NMR** (75 MHz, $CDCl_3$): δ 133.04, 132.08, 128.90, 117.08, 91.67, 81.35, 70.82 (d, J = 173.6 HZ), 79.22, 70.82 (d, J = 20.8 HZ).

HRMS (ESI$^+$): m/z calc'd for **3aj** $C_{10}H_{13}FNO_3S$ $[M+NH_4]^+$: 246.0595, found: 246.0591.

2-Chloroethyl 2-phenylethyne-1-sulfonate (3ak)

Following the general procedure A. 3-phenylpropiolic acid (88 mg, 0.6 mmol), 2-chlorethanol (181 µL, 217 mg, 2.7 mmol, 4.5 equiv.), SO_2 in DMSO (0.8 mL), and DCM (6 mL) were used. Purification via column chromatography on silica gel (cyclohexane: ethyl acetate = 98:3, v/v) afforded **3ak** as a colorless oil; Yield 67% (99 mg).

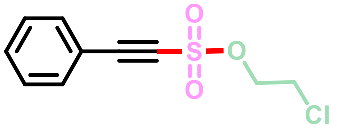

NMR Spectroscopy (see spectra):
^{1}**H NMR** (400 MHz, $CDCl_3$): δ 7.61 – 7.67 (m, 2H), 7.57 (tt, J = 7.5, 1.4 HZ, 1H), 7.42 – 7.49 (m, 2H), 4.60 (t, J = 5.8 HZ, 3H), 3.85 (t, J = 5.8 HZ, 3H).
^{13}**C NMR** (75 MHz, $CDCl_3$): δ 133.05, 132.13, 128.93, 117.03, 91.78, 79.28, 71.23, 40.42.

HRMS (ESI$^+$): m/z calc'd for **3ak** $C_{10}H_{13}ClNO_3S$ $[M+NH_4]^+$: 262.0299 (^{35}Cl), found: 262.0299.

2-Methoxyethyl 2-phenylethyne-1-sulfonate (3al)

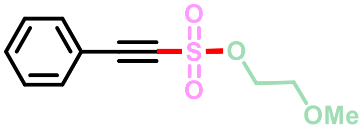

Following the general procedure A. 3-phenylpropiolic acid (88 mg, 0.6 mmol), 2-methoxyethanol (213 µL, 205 mg, 2.7 mmol, 4.5 equiv.), SO_2 in DMSO (0.8 mL), and DCM (6 mL) were used. Purification via column chromatography on silica gel (cyclohexane: ethyl acetate = 70:30, v/v) afforded **3al** as a colorless oil; Yield 56% (81 mg).

NMR Spectroscopy (see spectra):
^{1}H NMR (300 MHz, $CDCl_3$): δ 7.68 – 7.59 (m, 2H), 7.58 – 7.51 (m, 1H), 7.49 – 7.42 (m, 2H), 4.55 – 4.49 (m, 2H), 3.79 – 3.74 (m, 2H), 3.44 (s, 3H).
^{13}C NMR (75 MHz, $CDCl_3$): δ 132.98, 131.89, 128.85, 117.34, 91.12, 79.59, 71.47, 69.61, 59.21.

HRMS (ESI$^+$): m/z calc'd for **3al** $C_{11}H_{12}O_4SNa$ $[M+Na]^+$: 263.0349, found: 263.0350.

2-(2-Butoxyethoxy)ethyl 2-phenylethyne-1-sulfonate (3am)

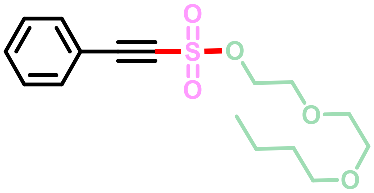

Following the general procedure A. 3-phenylpropiolic acid (88 mg, 0.6 mmol), 2-(2-butoxyethoxy)ethanol (456 µL, 438 mg, 2.7 mmol, 4.5 equiv.), SO_2 in DMSO (0.8 mL), and DCM (6 mL) were used. Purification via column chromatography on silica gel (cyclohexane: ethyl acetate = 50:50, v/v) afforded **3am** as a colorless oil; Yield 58% (115 mg).

NMR Spectroscopy (see spectra):
^{1}H NMR (400 MHz, $CDCl_3$): δ 0.91 (t, J = 7.3 HZ, 3H), 1.30 – 1.41 (m, 2H), 1.51 – 1.61 (m, 2H), 3.45 (t, J = 6.7HZ, 2H), 3.56 – 3.61 (m, 2H), 3.67 – 3.72 (m, 2H), 3.85 – 3.90 (m, 2H), 4.48 – 4.55 (m, 2H), 7.40 – 7.47 (m, 2H), 7.55 (tt, J = 7.6, 1.4 HZ, 1H), 7.59 – 7.64 (m, 2H).
^{13}C NMR (101 MHz, $CDCl_3$): δ 132.97, 131.90, 128.85, 117.31, 91.09, 79.59, 71.68, 71.27, 70.99, 70.08, 68.41, 31.68, 19.26, 13.91.

HRMS (ESI$^+$): m/z calc'd for **3am** $C_{16}H_{26}NO_5S$ $[M+NH_4]^+$: 344.1526, found: 344.1526.

2-(2-(2-Chloroethoxy)ethoxy)ethyl 2-phenylethyne-1-sulfonate (3an)

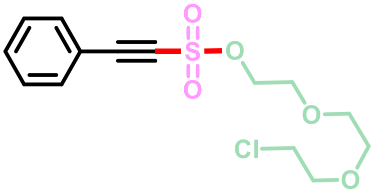

Following the general procedure A. 3-phenylpropiolic acid (88 mg, 0.6 mmol), 2-[2-(2-chloroethoxy)ethoxy]ethanol (392 µL, 455 mg, 2.7 mmol, 4.5 equiv.), SO_2 in DMSO (0.8 mL), and DCM (6 mL) were used. Purification via column chromatography on silica gel (cyclohexane: ethyl acetate = 50:50, v/v) afforded **3an** as a colorless oil; Yield 45% (89 mg).

NMR Spectroscopy (see spectra):
^{1}H NMR (400 MHz, $CDCl_3$): δ 7.60 – 7.66 (m, 2H), 7.56 (tt, J = 7.6, 1.4 HZ, 1H), 7.41 – 7.48 (m, 2H), 4.50 – 4.55 (m, 2H), 3.86 – 3.91(m, 2H), 3.68-3.79 (m, 6H), 3.60 – 3.65 (m, 2H).
^{13}C NMR (75 MHz, $CDCl_3$): δ 133.00, 131.96, 128.89, 117.27, 91.16, 79.57, 71.66, 71.41, 70.91, 70.67, 68.50, 42.81.

HRMS (ESI$^+$): m/z calc'd for **3an** $C_{14}H_{17}ClO_5SNa$ $[M+Na]^+$: 355.0377 (^{35}Cl), found: 355.0372.

(*S*)-(2,2-Dimethyl-1,3-dioxolan-4-yl)methyl 2-phenylethyne-1-sulfonate (3ao)

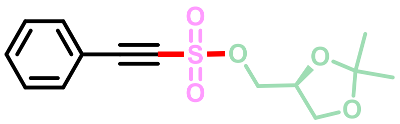

Following the general procedure A. 3-phenylpropiolic acid (88 mg, 0.6 mmol) (109 mg, 0.6 mmol), (*S*)-(+)-2,2-dimethyl-1,3-dioxolan-4-methanol (335 µL, 357 mg, 2.7 mmol, 4.5 equiv.), SO_2 in DMSO (0.8 mL), and DCM (6 mL) were used. Purification via column chromatography on silica gel (cyclohexane: ethyl acetate = 50:50, v/v) afforded **3ao** as a colorless oil; Yield 55% (98 mg).

Following the general procedure A. (Bromethinyl)benzol (109 mg, 0.6 mmol), (*S*)-(+)-2,2-dimethyl-1,3-dioxolan-4-methanol (335 µL, 357 mg, 2.7 mmol, 4.5 equiv.), SO_2 in DMSO (0.8 mL), and DCM (6 mL) were used. Purification via column chromatography on silica gel (cyclohexane: ethyl acetate = 50:50, v/v) afforded **3ao** as a colorless oil; Yield 48% (85 mg).

NMR Spectroscopy (see spectra):
^{1}H NMR (400 MHz, $CDCl_3$): δ 7.60 – 7.66 (m, 2H), 7.56 (tt, *J* = 7.6, 1.4 HZ, 1H), 7.42 – 7.48 (m, 2H), 4.42 – 4.50 (m, 1H), 4.29 – 4.40 (m, 2H), 4.13 – 4.18 (m, 1H), 3.91 – 3.96 (m, 1H), 1.46 (s, 3H), 1.38 (s, 3H).
^{13}C NMR (101 MHz, $CDCl_3$): δ 133.01, 132.03, 128.90, 117.13, 110.37, 91.54, 79.31, 72.67, 71.57, 66.11, 26.76, 25.19.

HRMS (ESI$^+$): m/z calc'd for **3ao** $C_{14}H_{20}NO_5S$ $[M+NH_4]^+$: 314.1057, found: 314.1049.
Specific rotation $[\alpha]_D^{25}$: -7.1 (c = 0.5, $CHCl_3$).

Phenethyl 2-phenylethyne-1-sulfonate (3ap)

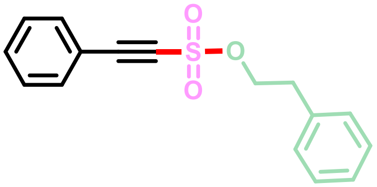

Following the general procedure A. 3-phenylpropiolic acid (88 mg, 0.6 mmol), 2-phenylethanol (323 µL, 330 mg, 2.7 mmol, 4.5 equiv.), SO_2 in DMSO (0.8 mL), and DCM (6 mL) were used. Purification via column chromatography on silica gel (cyclohexane: ethyl acetate = 96:4, v/v) afforded **3ap** as a colorless oil; Yield 51% (88 mg).

NMR Spectroscopy (see spectra):
^{1}H NMR (400 MHz, $CDCl_3$): δ 7.61 – 7.53 (m, 3H), 7.47 – 7.41 (m, 2H), 7.36 – 7.31 (m, 2H), 7.31 – 7.26 (m, 3H), 4.59 (t, *J* = 7.1 Hz, 2H), 3.17 (t, *J* = 7.1 Hz, 2H).
^{13}C NMR (75 MHz, $CDCl_3$): δ 135.90, 132.99, 131.90, 129.04, 128.84, 128.79, 127.18, 117.29, 90.92, 79.66, 73.03, 35.20.

HRMS (ESI$^+$): m/z calc'd for **3ap** $C_{16}H_{18}NO_3S$ $[M+NH_4]^+$: 304.1002, found: 304.0996.

4-Phenylbutyl 2-phenylethyne-1-sulfonate (3aq)

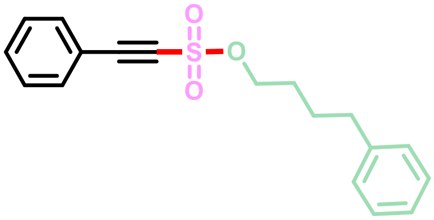

Following the general procedure A. 3-phenylpropiolic acid (88 mg, 0.6 mmol), 4-phenylbutan-1-ol (412 µL, 406 mg, 2.7 mmol, 4.5 equiv.), SO_2 in DMSO (0.8 mL), and DCM (6 mL) were used. Purification via column chromatography on silica gel (cyclohexane: ethyl acetate = 96:4, v/v) afforded **3aq** as a colorless oil; Yield 71% (134 mg).

NMR Spectroscopy (see spectra):
^{1}H NMR (400 MHz, $CDCl_3$): δ 7.66 – 7.51 (m, 3H), 7.48 – 7.41 (m, 2H), 7.33 – 7.28 (m, 2H), 7.25 – 7.18 (m, 3H), 4.43 (t, *J* = 6.0 Hz, 2H), 2.71 (t, *J* = 7.2 Hz, 2H), 1.94 – 1.79 (m, 4H).
^{13}C NMR (101 MHz, $CDCl_3$): δ 141.45, 132.94, 131.85, 128.87, 128.46, 128.40, 126.03, 117.36, 90.74, 79.83, 73.03, 35.15, 28.22, 27.25.

HRMS (ESI$^+$): m/z calc'd for **3aq** $C_{18}H_{18}O_3SNa$ $[M+Na]^+$: 337.0869, found: 337.0867.

Isopropyl 2-phenylethyne-1-sulfonate (3ar)

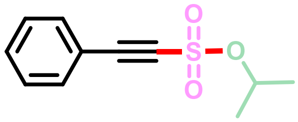

Following the general procedure A. 3-phenylpropiolic acid (88 mg, 0.6 mmol), isopropanol (206 µL, 162 mg, 2.7 mmol, 4.5 equiv.), SO_2 in DMSO (0.8 mL), and DCM (6 mL) were used. Purification via column chromatography on silica gel (cyclohexane: ethyl acetate = 4:96, v/v) afforded **3ar** as a colorless oil; Yield 61% (82 mg).

Following the general procedure A. (bromethinyl)benzol (109 mg, 0.6 mmol), isopropanol (206 µL, 162 mg, 2.7 mmol, 4.5 equiv.), SO_2 in DMSO (0.8 mL), and DCM (6 mL) were used. Purification via column chromatography on silica gel (cyclohexane: ethyl acetate = 4:96, v/v) afforded **3ar** as a colorless oil; Yield 56% (75 mg).

NMR Spectroscopy (see spectra):
^{1}**H NMR** (400 MHz, $CDCl_3$): δ 7.63 – 7.51 (m, 3H), 7.48 – 7.41 (m, 2H), 5.11 (hept, J = 6.3 Hz, 1H), 1.53 (d, J = 6.3 Hz, 6H).
^{13}**C NMR** (101 MHz, $CDCl_3$): δ 132.81, 131.76, 128.87, 117.53, 89.81, 81.01, 80.74, 22.66.

HRMS (ESI^+): m/z calc'd for **3ar** $C_{11}H_{16}NO_3S$ $[M+Na]^+$: 242.0845, found: 242.0843.

1-Cyanoethyl 2-phenylethyne-1-sulfonate (3as)

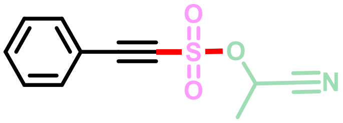

Following the general procedure A. 3-phenylpropiolic acid (88 mg, 0.6 mmol), lactonitrile (194 µL, 192 mg, 2.7 mmol, 4.5 equiv.), SO_2 in DMSO (0.8 mL), and DCM (6 mL) were used. Purification via column chromatography on silica gel (cyclohexane: ethyl acetate = 4:96, v/v) afforded **3as** as a colorless oil; Yield 52% (73 mg).

NMR Spectroscopy (see spectra):
^{1}**H NMR** (300 MHz, $CDCl_3$): δ 7.72 – 7.66 (m, 2H), 7.62 – 7.55 (m, 1H), 7.51 – 7.43 (m, 2H), 5.39 (q, J = 6.9 Hz, 1H), 1.88 (d, J = 6.9 Hz, 3H).
^{13}**C NMR** (101 MHz, $CDCl_3$): δ 133.20, 132.45, 128.96, 116.57, 115.31, 93.70, 64.87, 26.93, 20.10.

HRMS (ESI^+): m/z calc'd for **3as** $C_{11}H_{13}N_2O_3S$ $[M+Na]^+$: 253.0641, found: 253.0640.

1-Methoxypropan-2-yl 2-phenylethyne-1-sulfonate (3at)

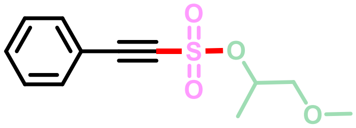

Following the general procedure A. 3-phenylpropiolic acid (88 mg, 0.6 mmol), propylene glycol methyl ether (264 µL, 243 mg, 2.7 mmol, 4.5 equiv.), SO_2 in DMSO (0.8 mL), and DCM (6 mL) were used. Purification via column chromatography on silica gel (cyclohexane: ethyl acetate = 4:96, v/v) afforded **3at** as a colorless oil; Yield 73% (111 mg).

NMR Spectroscopy (see spectra):
^{1}**H NMR** (300 MHz, $CDCl_3$): δ 7.64 – 7.57 (m, 2H), 7.57 – 7.51 (m, 1H), 7.46 – 7.41 (m, 2H), 5.10 – 4.98 (m, 1H), 3.64 – 3.54 (m, 2H), 3.41 (s, 3H), 1.52 (d, J = 6.5 Hz, 3H).7.72 – 7.66 (m, 2H), 7.62 – 7.55 (m, 1H), 7.51 – 7.43 (m, 2H), 5.39 (q, J = 6.9 Hz, 1H), 1.88 (d, J = 6.9 Hz, 3H).
^{13}**C NMR** (101 MHz, $CDCl_3$): δ 132.81, 131.78, 128.87, 117.52, 90.25, 81.35, 80.76, 74.51, 59.34, 17.35.

HRMS (ESI^+): m/z calc'd for **3at** $C_{12}H_{18}NO_4S$ $[M+NH_4]^+$: 272.0951, found: 272.0946.

Methyl (*R*)-3-(((phenylethynyl)sulfonyl)oxy)butanoate (3au)

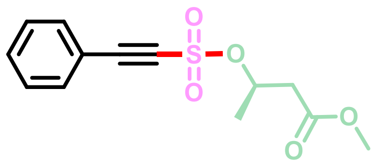

Following the general procedure A. 3-phenylpropiolic acid (88 mg, 0.6 mmol), methyl (*R*)-3-hydroxybutanoate (302 µL, 319 mg, 2.7 mmol, 4.5 equiv.), SO_2 in DMSO (0.8 mL), and DCM (6 mL) were used. Purification via column chromatography on silica gel (cyclohexane: ethyl acetate = 25:75, v/v) afforded **3au** as a colorless oil; Yield 62% (105 mg).

NMR Spectroscopy (see spectra):
^{1}**H NMR** (300 MHz, $CDCl_3$): δ 7.69 – 7.59 (m, 2H), 7.58 – 7.50 (m, 1H), 7.47 – 7.40 (m, 2H), 5.32 (h, *J* = 6.4 Hz, 1H), 3.69 (s, 3H), 2.94 (dd, *J* = 16.1, 6.7 Hz, 1H), 2.69 (dd, *J* = 16.1, 6.4 Hz, 1H), 1.60 (d, *J* = 6.3 Hz, 3H).
^{13}**C NMR** (75 MHz, $CDCl_3$): δ 169.53, 132.92, 131.87, 128.87, 117.37, 90.63, 80.40, 78.99, 52.08, 41.09, 20.81.

HRMS (ESI^+): m/z calc'd for **3au** $C_{13}H_{14}O_5SNa$ $[M+Na]^+$: 305.0454, found: 305.0457.
mp: 78.6 – 80.9 °C.
Specific rotation $[\alpha]_D^{25}$: +14.7 (c = 0.5, $CHCl_3$).

Tetrahydro-2H-pyran-4-yl 2-phenylethyne-1-sulfonate (3av)

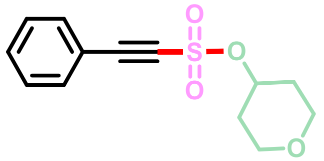

Following the general procedure A. 3-phenylpropiolic acid (88 mg, 0.6 mmol), tetrahydro-4-pyranol (257 µL, 275 mg, 2.7 mmol, 4.5 equiv.), SO_2 in DMSO (0.8 mL), and DCM (6 mL) were used. Purification via column chromatography on silica gel (cyclohexane: ethyl acetate = 25:75, v/v) afforded **3av** as a colorless oil; Yield 57% (91 mg).

NMR Spectroscopy (see spectra):
^{1}**H NMR** (400 MHz, $CDCl_3$): δ 7.66 – 7.59 (m, 2H), 7.56 (ddt, *J* = 8.6, 6.9, 1.4 Hz, 1H), 7.45 (td, *J* = 7.2, 1.2 Hz, 2H), 5.07 (tt, *J* = 8.1, 4.0 Hz, 1H), 3.99 (ddd, *J* = 11.9, 6.2, 3.9 Hz, 2H), 3.62 (ddd, *J* = 11.7, 8.0, 3.4 Hz, 2H), 2.19 – 2.11 (m, 2H), 2.07 – 1.97 (m, 2H).
^{13}**C NMR** (101 MHz, $CDCl_3$): δ 132.88, 131.92, 128.92, 117.32, 90.29, 80.91, 80.56, 64.65, 32.21.

HRMS (ESI^+): m/z calc'd for **3av** $C_{13}H_{18}NO_4S$ $[M+NH_4]^+$: 284.0951, found: 284.0947.

Cyclohexyl 2-phenylethyne-1-sulfonate (3aw)

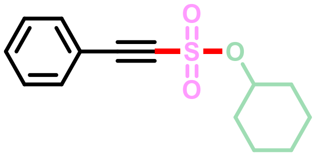

Following the general procedure A. 3-phenylpropiolic acid (88 mg, 0.6 mmol), cyclohexanol (281 µL, 270 mg, 2.7 mmol, 4.5 equiv.), SO_2 in DMSO (0.8 mL), and DCM (6 mL) were used. Purification via column chromatography on silica gel (cyclohexane: ethyl acetate = 95:2, v/v) afforded **3aw** as a colorless oil; Yield 38% (61 mg).

NMR Spectroscopy (see spectra):
^{1}**H NMR** (400 MHz, $CDCl_3$): δ 7.66 – 7.57 (m, 2H), 7.57 – 7.52 (m, 1H), 7.48 – 7.42 (m, 2H), 4.89 (tt, *J* = 8.1, 3.9 Hz, 1H), 2.12 – 2.04 (m, 2H), 1.88 – 1.76 (m, 4H), 1.59 – 1.51 (m, 1H), 1.50 – 1.33 (m, 4H).
^{13}**C NMR** (101 MHz, $CDCl_3$): δ 132.81, 131.67, 128.84, 117.64, 89.61, 85.10, 81.25, 32.13, 24.82, 23.28.

HRMS (ESI^+): m/z calc'd for **3aw** $C_{14}H_{20}O_3NS$ $[M+NH_4]^+$: 282.1159, found: 282.1167.

1,7,7-Trimethylbicyclo[2.2.1]heptan-2-yl 2-phenylethyne-1-sulfonate (3ax)

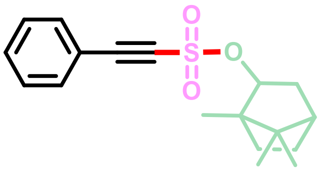

Following the general procedure A. 3-phenylpropiolic acid (88 mg, 0.6 mmol), (+)-borneol (416 mg, 2.7 mmol, 4.5 equiv.), SO_2 in DMSO (0.8 mL), and DCM (6 mL) were used. Purification via column chromatography on silica gel (cyclohexane: ethyl acetate = 95:5, v/v) afforded **3ax** as a colorless oil; Yield 59% (113 mg).

NMR Spectroscopy (see spectra):
^{1}H NMR (300 MHz, $CDCl_3$): δ 7.64 – 7.50 (m, 3H), 7.48 – 7.42 (m, 2H), 4.96 (ddd, *J* = 9.9, 3.3, 2.1 Hz, 1H), 2.50 – 2.41 (m, 1H), 2.05 – 1.93 (m, 1H), 1.85 – 1.76 (m, 2H), 1.54 (dd, *J* = 14.2, 3.3 Hz, 1H), 1.43 – 1.33 (m, 2H), 1.00 (s, 3H), 0.93 (d, *J* = 2.8 Hz, 6H).
^{13}C NMR (101 MHz, $CDCl_3$): δ 132.74, 131.71, 128.87, 117.59, 91.38, 90.12, 80.82, 49.79, 47.82, 44.70, 35.99, 27.76, 26.92, 26.64, 19.69, 18.82, 13.13.

HRMS (ESI$^+$): m/z calc'd for **3ax** $C_{18}H_{26}NO_3S$ $[M+NH_4]^+$: 336.1629, found: 336.1637.
Specific rotation $[\alpha]_D^{25}$: -18 (c = 0.5, $CHCl_3$).

(1*R*,2*S*,5*R*)-2-Isopropyl-5-methylcyclohexyl 2-phenylethyne-1-sulfonate (3ay)

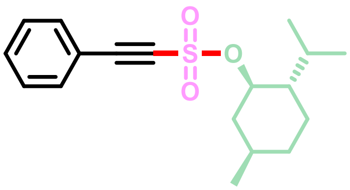

Following the general procedure A. 3-phenylpropiolic acid (88 mg, 0.6 mmol), L-menthol (422 mg, 2.7 mmol, 4.5 equiv.), SO_2 in DMSO (0.8 mL), and DCM (6 mL) were used. Purification via column chromatography on silica gel (cyclohexane: ethyl acetate = 95:2, v/v) afforded **3ay** as a colorless oil; Yield 54% (104 mg).

NMR Spectroscopy (see spectra):
^{1}H NMR (300 MHz, $CDCl_3$): δ 7.65 – 7.57 (m, 2H), 7.57 – 7.50 (m, 1H), 7.47 – 7.41 (m, 2H), 4.68 (td, *J* = 10.8, 4.6 Hz, 1H), 2.49 (dddd, *J* = 12.1, 5.0, 3.2, 1.9 Hz, 1H), 2.24 (pd, *J* = 7.0, 2.4 Hz, 1H), 1.74 (dddd, *J* = 18.9, 13.0, 6.3, 3.5 Hz, 2H), 1.58 – 1.46 (m, 2H), 1.43 – 1.33 (m, 1H), 1.31 – 1.25 (m, 1H), 1.10 (qd, *J* = 12.8, 3.0 Hz, 1H), 0.97 (dd, *J* = 6.7, 5.3 Hz, 6H), 0.94 – 0.90 (m, 1H), 0.85 (d, *J* = 6.9 Hz, 3H).
^{13}C NMR (101 MHz, $CDCl_3$): δ 132.74, 131.65, 128.85, 117.71, 89.86, 87.44, 81.44, 47.46, 41.65, 33.65, 31.74, 25.61, 23.11, 21.89, 20.92, 16.01.

HRMS (ESI$^+$): m/z calc'd for **3ay** $C_{18}H_{28}NO_3S$ $[M+NH_4]^+$: 338.1785, found: 338.1785.
Specific rotation $[\alpha]_D^{25}$: -67.1 (c = 0.5, $CHCl_3$).

((1*R*,3*R*,5*R*,7*R*)-Adamantan-2-yl)methyl 2-phenylethyne-1-sulfonate (3az)

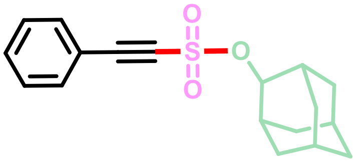

Following the general procedure A. 3-phenylpropiolic acid (88 mg, 0.6 mmol), 2-adamantanol (411 mg, 2.7 mmol, 4.5 equiv.), SO_2 in DMSO (0.8 mL), and DCM (6 mL) were used. Purification via column chromatography on silica gel (cyclohexane: ethyl acetate = 97:3, v/v) afforded **3az** as a colorless solid; Yield 49% (93 mg).

NMR Spectroscopy (see spectra):
^{1}H NMR (400 MHz, $CDCl_3$): δ 7.64 – 7.57 (m, 2H), 7.57 – 7.51 (m, 1H), 7.47 – 7.41 (m, 2H), 5.05 (t, *J* = 3.7 Hz, 1H), 2.35 (s, 2H), 2.23 – 2.15 (m, 2H), 1.99 – 1.87 (m, 4H), 1.85 – 1.75 (m, 4H), 1.69 – 1.61 (m, 2H).
^{13}C NMR (101 MHz, $CDCl_3$): δ 132.79, 131.66, 128.85, 117.66, 89.98, 89.49, 81.38, 37.06, 36.46, 32.65, 31.21, 26.82, 26.58.

HRMS (ESI$^+$): m/z calc'd for **3az** $C_{18}H_{24}NO_3S$ $[M+NH_4]^+$: 334.1471, found: 334.1470.
mp: 68.6 – 69.9 °C.

(5α-Cholestan-3β-yl) 2-phenylethyne-1-sulfonate (3aza)

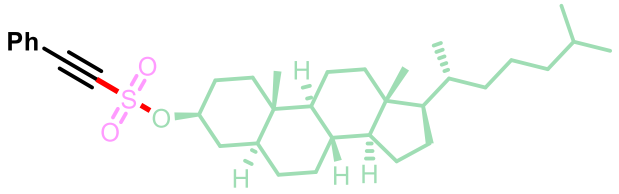

Following the general procedure A. 3-phenylpropiolic acid (14.5 mg, 0.1 mmol), 5α-cholestan-3β-ol (175 mg, 2.7 mmol, 4.5 equiv.), SO_2 in DMSO (130 μL), and DCM (2 mL) were used. Purification via column chromatography on silica gel (cyclohexane: ethyl acetate = 99:1, v/v) afforded **3aza** as a colorless solid; the yields were 15% (49 mg) and 27% (90 mg), corresponding to $Q_{Faraday}$ values of 4.84 F and 6.45 F, respectively.

NMR Spectroscopy (see spectra):
^{1}H NMR (400 MHz, $CDCl_3$): δ 7.69 – 7.58 (m, 2H), 7.57 – 7.48 (m, 1H), 7.48 – 7.40 (m, 2H), 2.19 – 2.10 (m, 1H), 1.99 (dt, J = 12.6, 3.5 Hz, 1H), 1.95 – 1.88 (m, 1H), 1.87 – 1.78 (m, 3H), 1.72 – 1.65 (m, 2H), 1.56 – 1.43 (m, 3H), 1.39 – 1.27 (m, 9H), 1.17 – 1.06 (m, 7H), 1.04 – 0.97 (m, 3H), 0.92 (d, J = 6.4 Hz, 4H), 0.89 (d, J = 1.8 Hz, 4H), 0.88 (d, J = 1.8 Hz, 3H), 0.86 (s, 3H), 0.67 (s, 3H).
^{13}C NMR (101 MHz, $CDCl_3$): δ 132.82, 131.67, 128.84, 117.67, 89.64, 85.95, 81.29, 56.37, 56.26, 54.09, 53.46, 44.88, 42.59, 39.92, 39.52, 36.84, 36.17, 35.79, 35.40, 35.30, 34.67, 31.91, 28.52, 28.27, 28.24, 28.03, 26.93, 24.20, 23.84, 22.84, 22.58, 21.23, 18.68, 12.19, 12.08.

HRMS (ESI$^+$): m/z calc'd for **3aza** $C_{35}H_{56}NO_3S$ $[M+NH_4]^+$: 570.3975, found: 570.3976.
Specific rotation $[\alpha]_D^{25}$: +24.5 (c = 0.5, $CHCl_3$).
mp: 80.3 – 89.9 °C.

1-((Phenylethynyl)sulfonyl)piperidine (3azb)

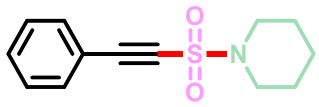

Following the general procedure A. 3-phenylpropiolic acid (88 mg, 0.6 mmol), piperidine (237 μL, 204 mg, 2.4 mmol, 4 equiv.), SO_2 in DMSO (0.8 mL), and DCM (6 mL) were used. Purification via column chromatography on silica gel (cyclohexane: ethyl acetate = 90:10, v/v) afforded **3azb** as a colorless solid; Yield 29% (44 mg).

NMR Spectroscopy (see spectra):
^{1}H NMR (300 MHz, $CDCl_3$): δ 7.61, 7.61, 7.61, 7.61, 7.60, 7.59, 7.59, 7.54, 7.53, 7.53, 7.52, 7.51, 7.51, 7.50, 7.49, 7.49, 7.45, 7.44, 7.43, 7.41, 7.41, 7.41, 3.27, 3.25, 3.24, 1.80, 1.79, 1.77, 1.76, 1.75, 1.66, 1.64, 1.63, 1.62, 1.61, 1.61, 1.59, 1.58, 1.56.
^{13}C NMR (101 MHz, $CDCl_3$): δ 132.78, 131.17, 128.75, 118.30, 90.32, 80.33, 47.37, 24.77, 23.38.

HRMS (ESI$^+$): m/z calc'd for **3azb** $C_{13}H_{15}NO_2SNa$ $[M+Na]^+$: 272.0716, found: 272.0716.
mp: 44.1 – 47.2 °C.

4-((Phenylethynyl)sulfonyl)morpholine (3azc)

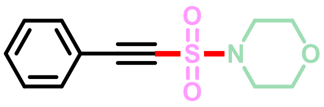

Following the general procedure A. 3-phenylpropiolic acid (88 mg, 0.6 mmol), morpholine (186 μL, 187 mg, 2.4 mmol, 4 equiv.), SO_2 in DMSO (0.8 mL), and DCM (6 mL) were used. Purification via column chromatography on silica gel (cyclohexane: ethyl acetate = 80:20, v/v) afforded **3azc** as a colorless solid; Yield 47% (71 mg).

Following the general procedure A. (bromethinyl)benzol (109 mg, 0.6 mmol), morpholine (186 μL, 187 mg, 2.4 mmol, 4 equiv.), SO_2 in DMSO (0.8 mL), and DCM (6 mL) were used. Purification via column chromatography on silica gel (cyclohexane: ethyl acetate = 80:20, v/v) afforded **3azc** as a colorless solid; Yield 39% (59 mg).

NMR Spectroscopy (see spectra):

^{1}**H NMR** (400 MHz, $CDCl_3$): δ 7.64 – 7.57 (m, 2H), 7.56 – 7.49 (m, 1H), 7.46 – 7.40 (m, 2H), 3.88 – 3.84 (m, 4H), 3.28 – 3.24 (m, 4H).
^{13}**C NMR** (101 MHz, $CDCl_3$): δ 132.89, 131.51, 128.84, 117.80, 91.43, 79.22, 65.74, 46.36.

HRMS (ESI^+): m/z calc'd for **3azc** $C_{12}H_{13}NO_3SNa$ $[M+Na]^+$: 274.0508, found: 274.0501.
mp: 103.6 – 105.1 °C.

4-Methoxy-1-((phenylethynyl)sulfonyl)piperidine (3azd)

Following the general procedure A. 3-phenylpropiolic acid (88 mg, 0.6 mmol), 4-methoxypiperidine (276 mg, 2.4 mmol, 4 equiv.), SO_2 in DMSO (0.8 mL), and DCM (6 mL) were used. Purification via column chromatography on silica gel (cyclohexane: ethyl acetate = 80:20, v/v) afforded **3azd** as a colorless oil; Yield 53% (89 mg).

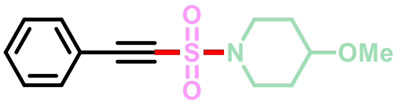

NMR Spectroscopy (see spectra):
^{1}**H NMR** (300 MHz, $CDCl_3$): δ 7.66 – 7.55 (m, 2H), 7.55 – 7.47 (m, 1H), 7.46 – 7.38 (m, 2H), 3.48 – 3.36 (m, 6H), 3.29 – 3.20 (m, 2H), 2.05 – 1.82 (m, 4H).
^{13}**C NMR** (101 MHz, $CDCl_3$): δ 132.83, 131.24, 128.76, 118.20, 90.54, 80.27, 73.53, 55.90, 43.38, 29.26.

HRMS (ESI^+): m/z calc'd for **3azd** $C_{14}H_{17}NO_3SNa$ $[M+Na]^+$: 302.0821, found: 302.0819.

***Cis*-2,6-Dimethyl-4-((phenylethynyl)sulfonyl)morpholine (3aze)**

Following the general procedure A. 3-phenylpropiolic acid (88 mg, 0.6 mmol), Cis-2,6-dimethylmorpholin (296 µL, 276 mg, 2.4 mmol, 4 equiv.), SO_2 in DMSO (0.8 mL), and DCM (6 mL) were used. Purification via column chromatography on silica gel (cyclohexane: ethyl acetate = 70:30, v/v) afforded **3aze** as a colorless solid; Yield 51% (85mg).

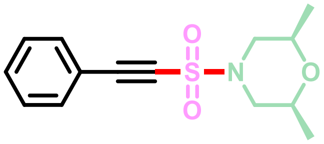

Following the general procedure A. (bromethinyl)benzol (109 mg, 0.6 mmol), cis-2,6-dimethylmorpholin (296 µL, 276 mg, 2.4 mmol, 4 equiv.), SO_2 in DMSO (0.8 mL), and DCM (6 mL) were used. Purification via column chromatography on silica gel (cyclohexane: ethyl acetate = 70:30, v/v) afforded **3aze** as a colorless solid; Yield 44% (73 mg).

NMR Spectroscopy (see spectra):
^{1}**H NMR** (300 MHz, $CDCl_3$): δ 7.64 – 7.57 (m, 2H), 7.56 – 7.48 (m, 1H), 7.46 – 7.40 (m, 2H), 3.86 – 3.73 (m, 2H), 3.62 – 3.57 (m, 2H), 2.44 (dd, J = 11.7, 10.3 Hz, 2H), 1.25 (d, J = 6.3 Hz, 6H).
^{13}**C NMR** (101 MHz, $CDCl_3$): δ 132.88, 131.45, 128.82, 117.90, 91.10, 79.47, 71.06, 51.10, 18.74.

HRMS (ESI^+): m/z calc'd for **3aze** $C_{14}H_{17}NO_3SNa$ $[M+Na]^+$: 302.0821, found: 302.0810.
mp: 103.2 – 105.7 °C.

4-(((4-Chlorophenyl)ethynyl)sulfonyl)-cis-2,6-dimethylmorpholine (3azf)

Following the general procedure A. 3-(4-chlorophenyl)propiolic acid (108 mg, 0.6 mmol), cis-2,6-dimethylmorpholin (296 µL, 276 mg, 2.4 mmol, 4 equiv.), SO_2 in DMSO (0.8 mL), and DCM (6 mL) were used. Purification via column chromatography on silica gel (cyclohexane: ethyl acetate = 70:30, v/v) afforded **3azf** as a colorless solid; Yield 57% (107 mg).

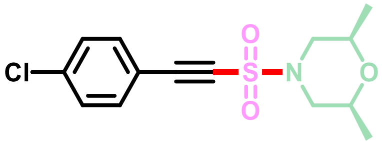

NMR Spectroscopy (see spectra):

^{1}**H NMR** (300 MHz, $CDCl_3$): δ 7.59 – 7.49 (m, 2H), 7.47 – 7.38 (m, 2H), 3.86 – 3.73 (m, 2H), 3.63 – 3.58 (m, 2H), 2.47 – 2.40 (m, 2H), 1.26 (d, *J* = 6.2 Hz, 6H).
^{13}**C NMR** (101 MHz, $CDCl_3$): δ 137.94, 134.06, 129.31, 116.38, 89.71, 80.44, 71.08, 51.05, 18.73.

HRMS (ESI^+): m/z calc'd for **3azf** $C_{14}H_{16}ClNO_3SNa$ $[M+Na]^+$: 336.0432(^{35}Cl), found: 336.0429.
mp: 110.1 – 112.7 °C.

***N*,*N*-Bis(2-methoxyethyl)-2-phenylethyne-1-sulfonamide (3azg)**

Following the general procedure A. 3-phenylpropiolic acid (88 mg, 0.6 mmol), bis(2-methoxyethyl)amine (354 µL, 319 mg, 2.4 mmol, 4 equiv.), SO_2 in DMSO (0.8 mL), and DCM (6 mL) were used. Purification via column chromatography on silica gel (cyclohexane: ethyl acetate = 50:50, v/v) afforded **3azg** as a colorless oil; Yield 31% (55 mg).

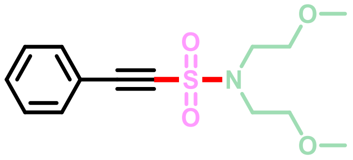

NMR Spectroscopy (see spectra):
^{1}**H NMR** (400 MHz, $CDCl_3$): δ 7.61 – 7.47 (m, 3H), 7.45 – 7.37 (m, 2H), 3.68 (t, *J* = 5.4 Hz, 4H), 3.59 (t, *J* = 5.8 Hz, 4H), 3.38 (s, 6H).
^{13}**C NMR** (101 MHz, $CDCl_3$): δ 132.58, 131.04, 128.73, 118.52, 88.32, 83.24, 71.09, 58.90, 49.44.

HRMS (ESI^+): m/z calc'd for **3azg** $C_{14}H_{19}NO_4SNa$ $[M+Na]^+$: 320.0929, found: 320.0937.

Neopentyl 2-(4-chlorophenyl)ethyne-1-sulfonate (3ba)

Following the general procedure A. 3-(4-chlorophenyl)propiolic acid (108 mg, 0.6 mmol), 2,2-dimethyl-1-propanol (238 mg, 2.7 mmol, 4.5 equiv.), SO_2 in DMSO (0.8 mL), and DCM (6 mL) were used. Purification via column chromatography on silica gel (cyclohexane: ethyl acetate = 98:2, v/v) afforded **3ba** as a colorless solid; Yield 68% (118 mg).

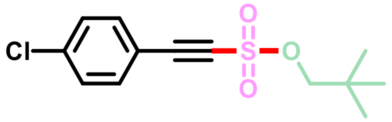

NMR Spectroscopy (see spectra):
^{1}**H NMR** (400 MHz, $CDCl_3$): δ 7.61 – 7.49 (m, 2H), 7.49 – 7.38 (m, 2H), 4.05 (s, 2H), 1.06 (s, 9H).
^{13}**C NMR** (101 MHz, $CDCl_3$): δ 138.41, 134.10, 129.41, 115.88, 89.30, 82.05, 80.60, 31.81, 26.06.

HRMS (ESI^+): m/z calc'd for **3ba** $C_{13}H_{19}ClNO_3S$ $[M+NH_4]^+$: 304.0769 (^{35}Cl), found: 304.0762.
mp: 93.2 – 95.6 °C.

Neopentyl 2-(4-bromophenyl)ethyne-1-sulfonate (3ca)

Following the general procedure A. 1-bromo-4-(bromoethynyl)benzene (156 mg, 0.6 mmol), 2,2-dimethyl-1-propanol (238 mg, 2.7 mmol, 4.5 equiv.), SO_2 in DMSO (0.8 mL), and DCM (6 mL) were used. Purification via column chromatography on silica gel (cyclohexane: ethyl acetate = 98:2, v/v) afforded **3ca** as a colorless solid; Yield 72% (143 mg).

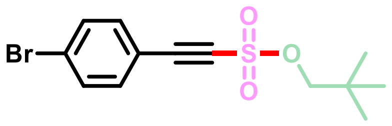

NMR Spectroscopy (see spectra):
^{1}**H NMR** (400 MHz, $CDCl_3$): δ 7.63 – 7.57 (m, 2H), 7.52 – 7.43 (m, 2H), 4.05 (s, 2H), 1.05 (s, 9H).
^{13}**C NMR** (101 MHz, $CDCl_3$): δ 134.14, 132.33, 126.85, 116.34, 89.34, 82.06, 80.71, 31.80, 26.05.

HRMS (ESI^+): m/z calc'd for **3ca** $C_{13}H_{19}BrNO_3S$ $[M+NH_4]^+$: 348.0264 (^{79}Br), found: 348.0261.
mp: 95.1 – 96.8 °C.

Neopentyl 2-(4-fluorophenyl)ethyne-1-sulfonate (3da)

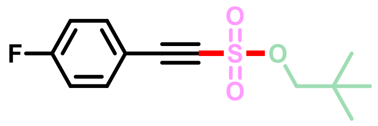

Following the general procedure A. 1-(bromoethynyl)-4-fluorobenzene (119 mg, 0.6 mmol), 2,2-dimethyl-1-propanol (238 mg, 2.7 mmol, 4.5 equiv.), SO_2 in DMSO (0.8 mL), and DCM (6 mL) were used. Purification via column chromatography on silica gel (cyclohexane: ethyl acetate = 98:2, v/v) afforded **3da** as a colorless solid; Yield 69% (112 mg).

NMR Spectroscopy (see spectra):
^{1}H NMR (400 MHz, $CDCl_3$): δ 7.68 – 7.59 (m, 2H), 7.19 – 7.12 (m, 2H), 4.05 (s, 2H), 1.06 (s, 9H).
^{13}C NMR (101 MHz, $CDCl_3$): δ 159.55 (d, J = 256.4 Hz), 135.31 (d, J = 9.1 Hz), 116.55 (d, J = 22.6 Hz), 113.57, 89.59, 81.96, 79.74, 79.72, 31.79, 26.06.
^{19}F NMR (282 MHz, $CDCl_3$): δ -103.96 (tt, J = 8.2, 5.1 Hz, 1F).

HRMS (ESI$^+$): m/z calc'd for **3da** $C_{13}H_{19}FNO_3S$ $[M+NH_4]^+$: 288.1064, found: 288.1056.
mp: 47.6 – 49.1 °C.

Neopentyl 2-(p-tolyl)ethyne-1-sulfonate (3ea)

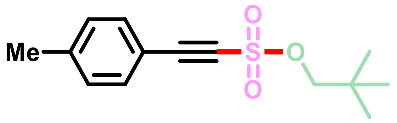

Following the general procedure A. 1-(bromoethynyl)-4-methylbenzene (117 mg, 0.6 mmol), 2,2-dimethyl-1-propanol (238 mg, 2.7 mmol, 4.5 equiv.), SO_2 in DMSO (0.8 mL), and DCM (6 mL) were used. Purification via column chromatography on silica gel (cyclohexane: ethyl acetate = 98:2, v/v) afforded **3ea** as a colorless solid; Yield 31% (49 mg).

NMR Spectroscopy (see spectra):
^{1}H NMR (400 MHz, $CDCl_3$): δ 7.51 (d, J = 8.2 Hz, 2H), 7.25 (d, J = 7.8 Hz, 2H), 4.04 (s, 2H), 2.43 (s, 3H), 1.06 (s, 9H).
^{13}C NMR (101 MHz, $CDCl_3$): δ 142.70, 132.87, 129.63, 114.29, 91.37, 81.78, 31.77, 26.08, 21.84.

HRMS (ESI$^+$): m/z calc'd for **3ea** $C_{14}H_{22}NO_3S$ $[M+NH_4]^+$: 284.1315, found: 284.1318.
mp: 44.7 – 45.9 °C.

Neopentyl 2-(4-(trifluoromethyl)phenyl)ethyne-1-sulfonate (3fa)

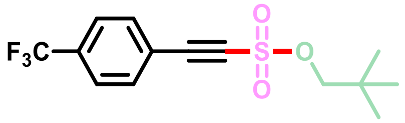

Following the general procedure A. 1-(bromoethynyl)-4-(trifluoromethyl)benzene (149 mg, 0.6 mmol), 2,2-dimethyl-1-propanol (238 mg, 2.7 mmol, 4.5 equiv.), SO_2 in DMSO (0.8 mL), and DCM (6 mL) were used. Purification via column chromatography on silica gel (cyclohexane: ethyl acetate = 98:2, v/v) afforded **3fa** as a colorless solid; Yield 67% (129 mg).

NMR Spectroscopy (see spectra):
^{1}H NMR (400 MHz, $CDCl_3$): δ 7.79 – 7.69 (m, 4H), 4.07 (s, 2H), 1.07 (s, 9H).
^{13}C NMR (101 MHz, $CDCl_3$): δ 133.49, 133.23, 133.16, 125.84 (q, J = 3.9 Hz), 124.66, 121.94, 121.29, 88.12, 82.27, 81.51, 31.82, 26.02.
^{19}F NMR (282 MHz, $CDCl_3$): δ -63.33 (s, 3F, CF_3).

HRMS (ESI$^+$): m/z calc'd for **3fa** $C_{14}H_{15}F_3O_3S$ $[M+NH_4]^+$: 338.1032, found: 338.1032.
mp: 53.3 – 55.1 °C.

Neopentyl 2-(4-cyanophenyl)ethyne-1-sulfonate (3ga)

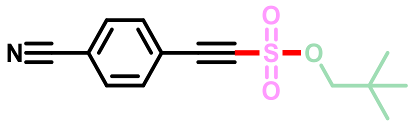

Following the general procedure A. 4-(bromoethynyl)benzonitrile (124 mg, 0.6 mmol), 2,2-dimethyl-1-propanol (238 mg, 2.7 mmol, 4.5 equiv.), SO_2 in DMSO (0.8 mL), and DCM (6 mL) were used. Purification via column chromatography on silica gel (cyclohexane: ethyl acetate = 95:5, v/v) afforded **3ga** as a colorless oil; Yield 74% (124 mg).

NMR Spectroscopy (see spectra):
^{1}H NMR (400 MHz, $CDCl_3$): δ 7.78 – 7.70 (m, 4H), 4.07 (s, 2H), 1.06 (s, 9H).
^{13}C NMR (101 MHz, $CDCl_3$): δ 133.33, 132.46, 122.19, 117.47, 115.27, 87.32, 82.89, 82.41, 31.84, 26.03.

HRMS (ESI$^+$): m/z calc'd for **3ga** $C_{14}H_{19}N_2O_3S$ $[M+NH_4]^+$: 295.1111, found: 295.1112.

Neopentyl 2-(2-bromophenyl)ethyne-1-sulfonate (3ha)

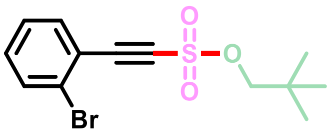

Following the general procedure A. 1-bromo-2-(bromoethynyl)benzene (156 mg, 0.6 mmol), 2,2-dimethyl-1-propanol (238 mg, 2.7 mmol, 4.5 equiv.), SO_2 in DMSO (0.8 mL), and DCM (6 mL) were used. Purification via column chromatography on silica gel (cyclohexane: ethyl acetate = 98:2, v/v) afforded **3ha** as a colorless oil; Yield 71% (141 mg).

NMR Spectroscopy (see spectra):
^{1}H NMR (400 MHz, $CDCl_3$): δ 7.72 – 7.57 (m, 2H), 7.43 – 7.36 (m, 2H), 4.10 (s, 2H), 1.06 (s, 9H).
^{13}C NMR (101 MHz, $CDCl_3$): δ 134.83, 133.03, 132.90, 127.57, 126.36, 120.22, 88.38, 83.19, 82.16, 31.78, 26.09.

HRMS (ESI$^+$): m/z calc'd for **3ha** $C_{13}H_{19}BrNO_3S$ $[M+NH_4]^+$: 348.0264 (^{79}Br), found: 348.0266.

Neopentyl 2-(4'-ethyl-[1,1'-biphenyl]-4-yl)ethyne-1-sulfonate (3ia)

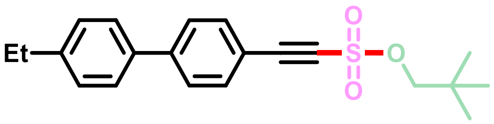

Following the general procedure A. 4-(bromoethynyl)-4'-ethyl-1,1'-biphenyl (171 mg, 0.6 mmol), 2,2-dimethyl-1-propanol (238 mg, 2.7 mmol, 4.5 equiv.), SO_2 in DMSO (0.8 mL), and DCM (6 mL) were used. Purification via column chromatography on silica gel (cyclohexane: ethyl acetate = 98:2, v/v) afforded **3ia** as a colorless solid; Yield 64% (137 mg).

NMR Spectroscopy (see spectra):
^{1}H NMR (400 MHz, $CDCl_3$): δ 7.71 – 7.65 (m, 4H), 7.56 (d, J = 8.3 Hz, 2H), 7.34 (d, J = 8.4 Hz, 2H), 4.09 (s, 2H), 2.74 (q, J = 7.6 Hz, 2H), 1.32 (t, J = 7.6 Hz, 3H), 1.09 (s, 9H).
^{13}C NMR (101 MHz, $CDCl_3$): δ 144.90, 144.63, 136.75, 133.38, 128.64, 127.26, 127.12, 115.63, 91.05, 81.90, 80.16, 31.81, 28.59, 26.10, 15.54.

HRMS (ESI$^+$): m/z calc'd for **3ia** $C_{21}H_{24}O_3SNa$ $[M+Na]^+$: 379.1338, found: 379.1336.
mp: 123.2 – 125.1 °C.

Neopentyl hept-1-yne-1-sulfonate (3ja)

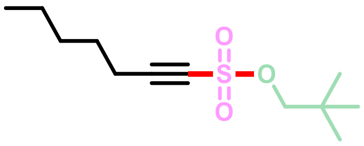

Following the general procedure A. 1-bromohept-1-yne (105 mg, 0.6 mmol), 2,2-dimethyl-1-propanol (238 mg, 2.7 mmol, 4.5 equiv.), SO_2 in DMSO (0.8 mL), and DCM (6 mL) were used. Purification via column chromatography on silica gel (cyclohexane: ethyl acetate = 99:1, v/v) afforded **3ja** as a colorless oil; Yield 66% (97 mg).

NMR Spectroscopy (see spectra):
^{1}H NMR (400 MHz, $CDCl_3$): δ 3.94 (s, 2H), 2.43 (t, J = 7.1 Hz, 2H), 1.68 – 1.59 (m, 2H), 1.45 – 1.31 (m, 4H), 1.03 (s, 9H), 0.93 (t, J = 7.1 Hz, 3H).
^{13}C NMR (101 MHz, $CDCl_3$): δ 94.79, 81.49, 72.37, 31.68, 30.90, 26.67, 26.04, 22.00, 18.61, 13.82.

HRMS (ESI$^+$): m/z calc'd for **3ja** $C_{12}H_{22}O_3S$ $[M+NH_4]^+$: 264.1628, found: 264.1620.

Neopentyl dec-1-yne-1-sulfonate (3ka)

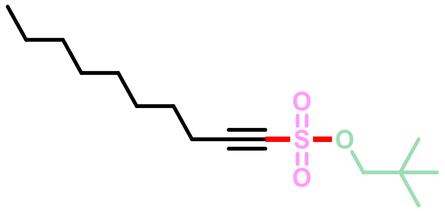

Following the general procedure A. 1-bromodec-1-yne (130 mg, 0.6 mmol), 2,2-dimethyl-1-propanol (238 mg, 2.7 mmol, 4.5 equiv.), SO_2 in DMSO (0.8 mL), and DCM (6 mL) were used. Purification via column chromatography on silica gel (cyclohexane: ethyl acetate = 99:1, v/v) afforded **3ka** as a colorless oil; Yield 52% (90 mg).

NMR Spectroscopy (see spectra):
^{1}H NMR (400 MHz, $CDCl_3$): δ 3.95 (s, 2H), 2.44 (t, J = 7.1 Hz, 2H), 1.69 – 1.58 (m, 2H), 1.52 – 1.18 (m, 12H), 1.03 (s, 9H), 0.94 – 0.86 (m, 3H).
^{13}C NMR (101 MHz, $CDCl_3$): δ 94.81, 81.50, 72.37, 31.75, 31.69, 29.06, 28.89, 28.81, 27.00, 26.06, 22.62, 18.66, 14.08.

HRMS (ESI$^+$): m/z calc'd for **3ka** $C_{15}H_{32}NO_3S$ $[M+NH_4]^+$: 306.2097, found: 306.2098.

Neopentyl 5-chloropent-1-yne-1-sulfonate (3la)

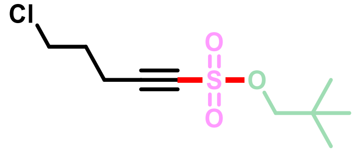

Following the general procedure A. 1-bromo-5-chloropent-1-yne (109 mg, 0.6 mmol), 2,2-dimethyl-1-propanol (238 mg, 2.7 mmol, 4.5 equiv.), SO_2 in DMSO (0.8 mL), and DCM (6 mL) were used. Purification via column chromatography on silica gel (cyclohexane: ethyl acetate = 99:1, v/v) afforded **3ka** as a colorless oil; Yield 32% (49 mg).

NMR Spectroscopy (see spectra):
^{1}H NMR (300 MHz, $CDCl_3$): δ 3.96 (s, 2H), 3.66 (t, J = 6.0 Hz, 2H), 2.67 (t, J = 7.0 Hz, 2H), 2.17 – 2.03 (m, 2H), 1.03 (s, 9H).
^{13}C NMR (75 MHz, $CDCl_3$): δ 92.4, 81.8, 73.3, 42.9, 31.7, 29.6, 26.0, 16.1.

HRMS (ESI$^+$): m/z calc'd for **3la** $C_{10}H_{21}ClNO_3S$ $[M+NH_4]^+$: 270.0930, found: 270.0928.

Neopentyl 6-cyanohex-1-yne-1-sulfonate (3ma)

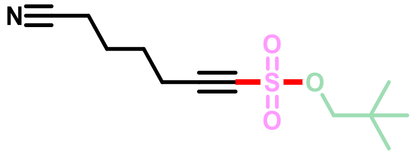

Following the general procedure A. 7-bromohept-6-ynenitrile (112 mg, 0.6 mmol), 2,2-dimethyl-1-propanol (238 mg, 2.7 mmol, 4.5 equiv.), SO_2 in DMSO (0.8 mL), and DCM (6 mL) were used. Purification via column chromatography on silica gel (cyclohexane: ethyl acetate = 95:5, v/v) afforded **3ma** as a colorless oil; Yield 62% (96 mg).

NMR Spectroscopy (see spectra):
^{1}H NMR (300 MHz, $CDCl_3$): δ 3.92 (s, 2H), 2.54 – 2.36 (m, 4H), 1.83 – 1.73 (m, 4H), 0.99 (s, 9H).
^{13}C NMR (75 MHz, $CDCl_3$): δ 119.0, 92.8, 81.8, 73.2, 31.7, 26.0, 25.9, 24.4, 18.1, 16.8.

HRMS (ESI$^+$): m/z calc'd for **3ma** $C_{12}H_{23}N_2O_3S$ $[M+NH_4]^+$: 275.1424, found: 275.1422.

Ethyl 6-((neopentyloxy)sulfonyl)hex-5-ynoate (3na)

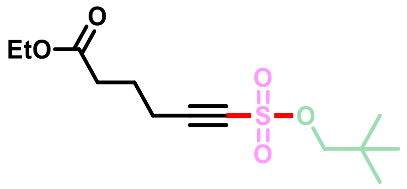

Following the general procedure A. Ethyl 6-bromohex-5-ynoate (131 mg, 0.6 mmol), 2,2-dimethyl-1-propanol (238 mg, 2.7 mmol, 4.5 equiv.), SO_2 in DMSO (0.8 mL), and DCM (6 mL) were used. Purification via column chromatography on silica gel (cyclohexane: ethyl acetate = 92:8, v/v) afforded **3na** as a colorless oil; Yield 63% (110 mg).

NMR Spectroscopy (see spectra):
^{1}H NMR (400 MHz, $CDCl_3$): δ 4.11 (q, J = 7.1 Hz, 2H), 3.90 (s, 2H), 2.50 (t, J = 7.1 Hz, 2H), 2.40 (t, J = 7.2 Hz, 2H), 1.90 (p, J = 7.1 Hz, 2H), 1.23 (t, J = 7.1 Hz, 3H), 0.97 (s, 9H).
^{13}C NMR (101 MHz, $CDCl_3$): δ 172.2, 93.2, 81.6, 73.0, 60.6, 32.6, 31.7, 26.0, 22.2, 18.0, 14.2.

HRMS (ESI$^+$): m/z calc'd for **3na** $C_{13}H_{26}NO_5S$ $[M+NH_4]^+$: 308.1526, found: 308.1525.

6-((neopentyloxy)sulfonyl)hex-5-yn-1-yl 4-methylbenzenesulfonate (3oa)

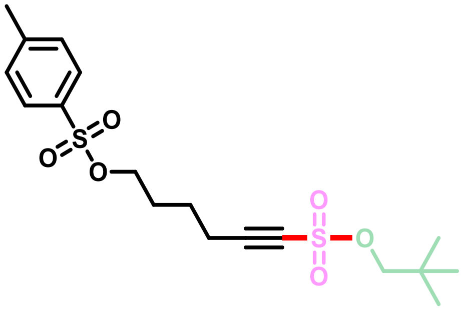

Following the general procedure A. 6-bromohex-5-yn-1-yl 4-methylbenzenesulfonate (199 mg, 0.6 mmol), 2,2-dimethyl-1-propanol (238 mg, 2.7 mmol, 4.5 equiv.), SO_2 in DMSO (0.8 mL), and DCM (6 mL) were used. Purification via column chromatography on silica gel (cyclohexane: ethyl acetate = 90:10, v/v) afforded **3oa** as a colorless oil; Yield 69% (164 mg).

NMR Spectroscopy (see spectra):
^{1}H NMR (400 MHz, $CDCl_3$): δ 7.76 (d, J = 8.4 Hz, 2H), 7.35 (d, J = 7.7 Hz, 2H), 4.02 (t, J = 5.9 Hz, 2H), 3.90 (s, 2H), 2.44 (s, 3H), 2.41 (t, J = 6.9 Hz, 2H), 1.79 – 1.61 (m, 4H), 0.98 (s, 9H).
^{13}C NMR (101 MHz, $CDCl_3$): δ 145.1, 132.8, 130.0, 127.8, 93.3, 81.7, 73.0, 69.3, 31.7, 27.8, 26.0, 23.2, 21.7, 18.0.

HRMS (ESI$^+$): m/z calc'd for **3oa** $C_{18}H_{30}NO_6S_2$ $[M+NH_4]^+$: 420.1511, found: 420.1509.

Neopentyl 6-phenethoxyhex-1-yne-1-sulfonate (3pa)

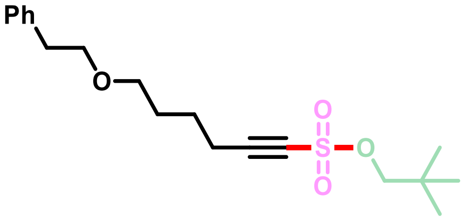

Following the general procedure A. (2-((6-bromohex-5-yn-1-yl)oxy)ethyl)benzene (169 mg, 0.6 mmol), 2,2-dimethyl-1-propanol (238 mg, 2.7 mmol, 4.5 equiv.), SO_2 in DMSO (0.8 mL), and DCM (6 mL) were used. Purification via column chromatography on silica gel (cyclohexane: ethyl acetate = 95:5, v/v) afforded **3pa** as a colorless oil; Yield 63 % (129 mg).

NMR Spectroscopy (see spectra):
^{1}H NMR (300 MHz, $CDCl_3$): δ 7.42 – 7.28 (m, 5H), 4.52 (s, 2H), 3.95 (s, 2H), 3.56 – 3.49 (m, 2H), 2.53 – 2.43 (m, 2H), 1.80 – 1.71 (m, 4H), 1.03 (s, 9H).
^{13}C NMR (75 MHz, $CDCl_3$): δ 138.3, 128.4, 127.7, 127.6, 94.4, 81.5, 73.0, 72.6, 69.2, 31.7, 28.8, 26.9, 26.1, 24.1, 18.5.

HRMS (ESI$^+$): m/z calc'd for **3pa** $C_{18}H_{30}NO_4S$ $[M+NH_4]^+$: 356.1890, found: 356.1881.

Neopentyl 6-(2-oxo-2-phenylethoxy)hex-1-yne-1-sulfonate (3qa)

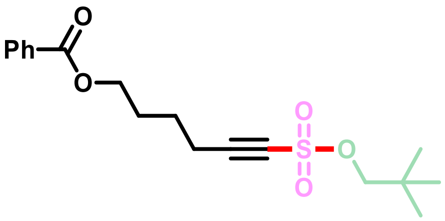

Following the general procedure A. 2-((6-bromohex-5-yn-1-yl)oxy)-1-phenylethan-1-one (177 mg, 0.6 mmol), 2,2-dimethyl-1-propanol (238 mg, 2.7 mmol, 4.5 equiv.), SO_2 in DMSO (0.8 mL), and DCM (6 mL) were used. Purification via column chromatography on silica gel (cyclohexane: ethyl acetate = 95:5, v/v) afforded **3qa** as a colorless oil; Yield 67% (141 mg).

NMR Spectroscopy (see spectra):
^{1}**H NMR** (300 MHz, $CDCl_3$): δ 8.07 – 7.97 (m, 2H), 7.61 – 7.50 (m, 1H), 7.47 – 7.39 (m, 2H), 4.34 (t, J = 6.1 Hz, 2H), 3.92 (s, 2H), 2.51 (t, J = 6.9 Hz, 2H), 1.95 – 1.72 (m, 4H), 0.98 (s, 9H).
^{13}**C NMR** (75 MHz, $CDCl_3$): δ 166.5, 133.1, 130.1, 129.6, 128.4, 93.8, 81.6, 72.9, 63.9, 31.7, 27.9, 26.0, 23.9, 18.4.

HRMS (ESI$^+$): m/z calc'd for **3qa** $C_{18}H_{28}NO_5S$ [M+NH$_4$]$^+$: 370.1683, found: 370.1676.

6-((Neopentyloxy)sulfonyl)hex-5-yn-1-yl 2-(11-oxo-6,11-dihydrodibenzo[*b*,*e*]oxepin-2-yl)acetate (3ra)

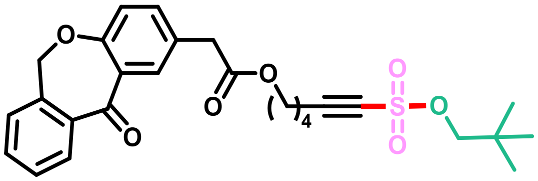

Following the general procedure A. 6-bromohex-5-yn-1-yl 2-(11-oxo-6,11-dihydrodibenzo[*b*,*e*]oxepin-2-yl)acetate (256 mg, 0.6 mmol),, 2,2-dimethyl-1-propanol (238 mg, 2.7 mmol, 4.5 equiv.), SO_2 in DMSO (0.8 mL), and DCM (6 mL) were used. Purification via column chromatography on silica gel (cyclohexane: ethyl acetate = 90:10, v/v) afforded **3ra** as a colorless oil; Yield 66% (198 mg).

NMR Spectroscopy (see spectra):
^{1}**H NMR** (400 MHz, $CDCl_3$): δ 8.08 (d, J = 2.4 Hz, 1H), 7.84 (dd, J = 7.7, 1.4 Hz, 1H), 7.52 (td, J = 7.5, 1.5 Hz, 1H), 7.43 (td, J = 7.6, 1.3 Hz, 1H), 7.39 (dd, J = 8.4, 2.4 Hz, 1H), 7.32 (dd, J = 7.4, 1.4 Hz, 1H), 7.00 (d, J = 8.4 Hz, 1H), 5.14 (s, 2H), 4.09 (t, J = 6.1 Hz, 2H), 3.90 (s, 2H), 3.61 (s, 2H), 2.42 (t, J = 6.9 Hz, 2H), 1.77 – 1.60 (m, 4H), 0.97 (s, 9H).
^{13}**C NMR** (101 MHz, $CDCl_3$): δ 190.7, 171.3, 160.5, 140.4, 136.3, 135.6, 132.8, 132.3, 129.4, 129.2, 127.9, 127.7, 125.1, 121.1, 93.8, 81.6, 73.6, 72.8, 63.9, 40.2, 31.7, 27.6, 26.0, 23.7, 18.3.

HRMS (ESI$^+$): m/z calc'd for **3ra** $C_{27}H_{34}NO_7S$ [M+NH$_4$]$^+$: 516.2050, found: 516.2052.

(*R*)-2,5,7,8-Tetramethyl-2-((4*R*,8*R*)-4,8,12-trimethyltridecyl)chroman-6-yl 6-((neopentyloxy)sulfonyl)hex-5-ynoate (3sa)

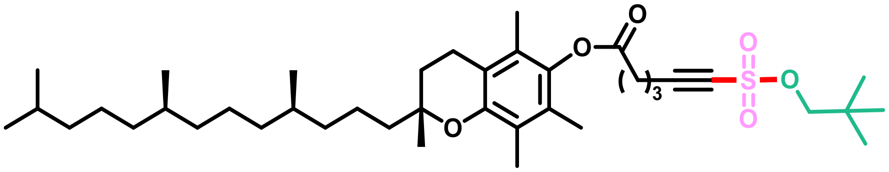

Following the general procedure A. (*R*)-2,5,7,8-tetramethyl-2-((4*R*,8*R*)-4,8,12-trimethyltridecyl)chroman-6-yl 6-bromohex-5-ynoate (362 mg, 0.6 mmol), 2,2-dimethyl-1-propanol (238 mg, 2.7 mmol, 4.5 equiv.), SO_2 in DMSO (0.8 mL), and DCM (6 mL) were used. Purification via column chromatography on silica gel (cyclohexane: ethyl acetate = 90:10, v/v) afforded **3sa** as a colorless oil; Yield 57% (230 mg).

NMR Spectroscopy (see spectra):
^{1}**H NMR** (300 MHz, $CDCl_3$): δ 3.96 (s, 2H), 2.76 (t, J = 7.2 Hz, 2H), 2.66 – 2.57 (m, 4H), 2.14 – 2.06 (m, 5H), 2.02 (s, 3H), 1.98 (s, 3H), 1.88 – 1.70 (m, 2H), 1.62 – 1.35 (m, 8H), 1.32 – 1.23 (m, 10H), 1.19 – 1.05 (m, 7H), 1.03 (s, 9H), 0.90 – 0.84 (m, 12H).

^{13}C NMR (75 MHz, $CDCl_3$): δ 170.9, 149.5, 140.3, 126.5, 124.8, 123.1, 117.5, 92.9, 81.7, 75.1, 73.3, 39.4, 37.6, 37.5, 37.4, 37.4, 37.3, 32.8, 32.7, 32.3, 31.7, 31.1, 28.0, 26.1, 24.8, 24.5, 22.8, 22.7, 22.4, 21.1, 20.6, 19.8, 19.7, 19.7, 19.6, 18.0, 13.1, 12.2, 11.9.

HRMS (ESI^+): m/z calc'd for **3sa** $C_{40}H_{70}NO_6S$ $[M+NH_4]^+$: 692.4920, found: 692.4901.

Neopentyl 2-(4-(bromoethynyl)phenyl)ethyne-1-sulfonate (3ta)

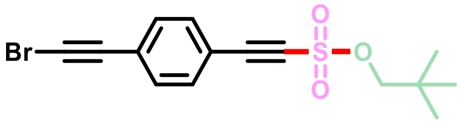

Following the general procedure A.1,4-bis(bromoethynyl)benzene (170 mg, 0.6 mmol), 2,2-dimethyl-1-propanol (238 mg, 2.7 mmol, 4.5 equiv.), SO_2 in DMSO (0.8 mL), and DCM (6 mL) were used. Purification via column chromatography on silica gel (cyclohexane: ethyl acetate = 96:4, v/v) afforded **3ta** as a colorless solid; Yield 23% (50 mg) and 41% (89 mg), corresponding to $Q_{Faraday}$ values of 4.84 F and 6.45 F, respectively.

NMR Spectroscopy (see spectra):
^{1}H NMR (400 MHz, $CDCl_3$): δ 7.61 – 7.44 (m, 4H), 4.05 (s, 2H), 1.06 (s, 9H).
^{13}C NMR (101 MHz, $CDCl_3$): 132.77, 132.32, 126.36, 117.40, 89.68, 82.05, 81.26, 79.01, 54.82, 31.81, 26.07.

HRMS (ESI^+): m/z calc'd for **3ta** $C_{15}H_{19}BrNO_3S$ $[M+NH_4]^+$: 372.0264 (^{79}Br), found: 372.0254.
mp: 102.5 – 104.1 °C.

(*S*)-2-Methylbutyl 2-(4-(((neopentyloxy)sulfonyl)ethynyl)phenyl)ethyne-1-sulfonate (3ua)

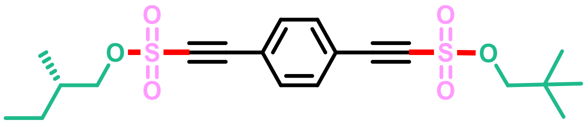

Following the general procedure A. neopentyl 2-(4-(bromoethynyl)phenyl)ethyne-1-sulfonate (212 mg, 0.6 mmol), (*S*)-(−)-2-methyl-1-butanol (292 µL, 238 mg, 2.7 mmol, 4.5 equiv.), SO_2 in DMSO (0.8 mL), and DCM (6 mL) were used. Purification via column chromatography on silica gel (cyclohexane: ethyl acetate = 95:5, v/v) afforded **3ua** as a colorless solid; Yield 70% (179 mg).

NMR Spectroscopy (see spectra):
^{1}H NMR (400 MHz, $CDCl_3$): δ 7.65 (s, 4H), 4.28 – 4.17 (m, 2H), 4.04 (s, 2H), 1.90 (dh, J = 13.0, 6.6 Hz, 1H), 1.52 (dqd, J = 13.2, 7.5, 5.7 Hz, 1H), 1.34 – 1.23 (m, 1H), 1.02 (d, J = 8.4 Hz, 12H), 0.94 (t, J = 7.5 Hz, 3H).
^{13}C NMR (101 MHz, $CDCl_3$): δ 133.06, 120.74, 88.13, 88.08, 82.61, 82.52, 82.31, 77.74, 34.30, 31.83, 26.03, 25.43, 16.02, 11.04.

HRMS (ESI^+): m/z calc'd for **3ua** $C_{20}H_{30}NO_6S_2$ $[M+NH_4]^+$: 444.1509, found: 444.1487.
mp: 87.2 – 89.1 °C.

Neopentyl (3,5-di-tert-butyl-4-hydroxyphenyl)methanesulfonate (3va)

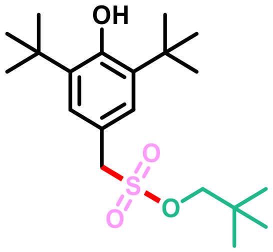

(see the procedure)Purification via column chromatography on silica gel (cyclohexane: ethyl acetate = 95:5, v/v) afforded **3ta** as a colorless oil; Yield 37% (55 mg).

NMR Spectroscopy (see spectra):
^{1}H NMR (300 MHz, $CDCl_3$): δ 7.18 (s, 2H), 5.33 (s, 1H), 4.28 (s, 2H), 3.70 (s, 2H), 1.44 (s, 18H), 0.89 (s, 9H).
^{13}C NMR (101 MHz, $CDCl_3$): δ 154.47, 136.34, 127.50, 118.57, 79.14, 56.82, 34.32, 31.74, 30.19, 25.99.

HRMS (ESI^+): m/z calc'd for **3va** $C_{20}H_{38}NO_4S$ $[M+NH_4]^+$: 388.2516, found: 388.2511.

Neopentyl (*E*)-benzofuran-3(2*H*)-ylidenebromomethanesulfonate (3wa)

Following the general procedure A. ((3-bromoprop-2-yn-1-yl)oxy)benzene (127 mg, 0.6 mmol), 2,2-dimethyl-1-propanol (238 mg, 2.7 mmol, 4.5 equiv.), SO_2 in DMSO (0.8 mL), and DCM (6 mL) were used. Purification via column chromatography on silica gel (cyclohexane: ethyl acetate = 90:10, v/v) afforded **3wa** as a colorless oil; Yield 45% (98 mg).

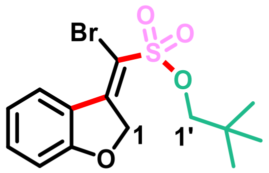

NMR Spectroscopy (see spectra):
^{1}H NMR (400 MHz, $CDCl_3$): δ 7.74 (dd, J = 7.9, 1.6 Hz, 1H), 7.39 (ddd, J = 8.2, 7.4, 1.6 Hz, 1H), 7.12 (td, J = 7.7, 1.2 Hz, 1H), 6.95 (dd, J = 8.2, 1.2 Hz, 1H), 5.00 (s, 2H), 3.90 (s, 2H), 1.03 (s, 9H).
^{13}C NMR (101 MHz, $CDCl_3$): δ 155.0, 133.7, 129.7, 128.0, 126.6, 122.8, 122.0, 116.5, 80.4, 66.4, 31.8, 26.2.
E/*Z*-isomerism was determined via a *^{1}H,^{1}H-NOESY*-contact between 1-H and 1'-H, which is only possible in the (*E*)-configured molecule.

HRMS (ESI$^+$): m/z calc'd for **3wa** $C_{14}H_{21}BrNO_4S$ $[M+NH_4]^+$: 378.0369, found: 378.0357.

Dineopentyl (*E*)-2,4,4-triphenylbuta-1,3-diene-1,3-disulfonate (4a)

Following the general procedure B. neopentyl 2-phenylethyne-1-sulfonate (**3ah**) (15.2 mg, 0.1 mmol), phenylboronic acid (36.6 mg, 0.3 mmol, 3 equiv.), $Cu(OAc)_2$ (1.8 mg), and MeOH (0.5 mL) were used. Purification via column chromatography on silica gel (cyclohexane: ethyl acetate = 96:4, v/v) afforded **4a** as a colorless solid; Yield 85% (25 mg).

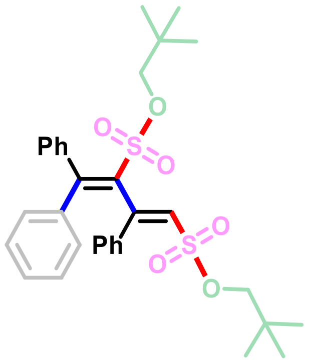

NMR Spectroscopy (see spectra):
^{1}H NMR (300 MHz, $CDCl_3$): δ 7.40 – 7.27 (m, 8H), 7.26 – 7.15 (m, 6H), 6.97 – 6.90 (m, 3H), 3.74 (s, 2H), 3.57 (s, 2H), 0.91 (s, 9H), 0.83 (s, 9H).
^{13}C NMR (75 MHz, $CDCl_3$): δ 157.62, 147.79, 139.79, 138.83, 136.05, 135.61, 129.94, 129.41, 129.33, 129.19, 129.13, 128.81, 128.71, 128.38, 128.03, 127.59, 79.90, 79.67, 31.75, 31.49, 26.06, 25.88.

HRMS (ESI$^+$): m/z calc'd for **4a** $C_{32}H_{38}O_6S_2Na$ $[M+Na]^+$: 605.2002, found: 605.2009.
mp: 150.5 – 152.7 °C.

Dineopentyl (1*E*,3*E*)-4-(3-methoxyphenyl)-2,4-diphenylbuta-1,3-diene-1,3-disulfonate (4b)

Following the general procedure B. neopentyl 2-phenylethyne-1-sulfonate (**3ah**) (25.2 mg, 0.1 mmol), (4-methoxyphenyl)boronic acid (36.6 mg, 0.3 mmol, 3 equiv.), $Cu(OAc)_2$ (1.8 mg), and MeOH (0.5 mL) were used. Purification via column chromatography on silica gel (cyclohexane: ethyl acetate = 96:4, v/v) afforded **4b** as a yellow oil; Yield 88% (27 mg).

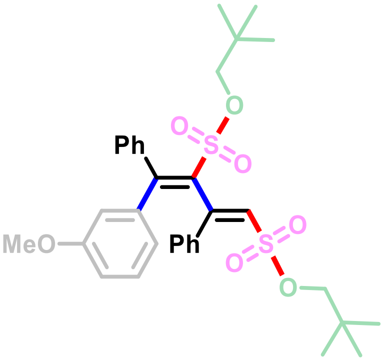

NMR Spectroscopy (see spectra):
^{1}H NMR (400 MHz, $CDCl_3$): δ 7.39 – 7.31 (m, 7H), 7.28 – 7.18 (m, 3H), 7.13 – 7.08 (m, 1H), 6.90 (s, 1H), 6.78 (ddd, J = 8.4, 2.6, 0.9 Hz, 1H), 6.54 (ddd, J = 7.6, 1.6, 1.0 Hz, 1H), 6.41 (dd, J = 2.6, 1.5 Hz, 1H), 3.73 (s, 2H), 3.66 (s, 3H), 3.58 (s, 2H), 0.90 (s, 9H), 0.83 (s, 9H).
^{13}C NMR (101 MHz, $CDCl_3$): δ 159.35, 157.36, 147.77, 140.91, 138.62, 136.11, 135.59, 129.68, 129.47, 129.40, 129.19, 128.60, 128.02, 127.59, 121.31, 115.31, 113.72, 79.90, 79.66, 55.26, 31.73, 31.47, 26.04, 25.85.

HRMS (ESI$^+$): m/z calc'd for **4b** $C_{33}H_{40}O_7S_2Na$ $[M+Na]^+$: 635.2108, found: 635.2128.

Dineopentyl (1*E*,3*E*)-4-(4-methoxyphenyl)-2,4-diphenylbuta-1,3-diene-1,3-disulfonate (4c)

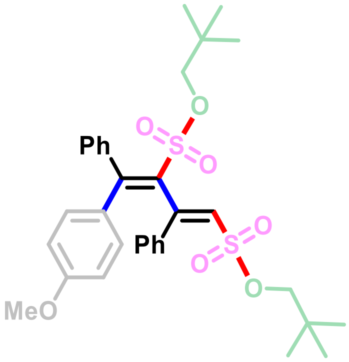

Following the general procedure B. neopentyl 2-phenylethyne-1-sulfonate (**3ah**) (25.2 mg, 0.1 mmol), (4-methoxyphenyl)boronic acid (36.6 mg, 0.3 mmol, 3 equiv.), $Cu(OAc)_2$ (1.8 mg), and MeOH (0.5 mL) were used. Purification via column chromatography on silica gel (cyclohexane: ethyl acetate = 90:10, v/v) afforded **4c** as a colorless solid; Yield 88% (27 mg).

NMR Spectroscopy (see spectra):
^{1}**H NMR** (300 MHz, $CDCl_3$): δ 7.43 – 7.29 (m, 7H), 7.24 – 7.15 (m, 3H), 6.96 (s, 1H), 6.90 – 6.84 (m, 2H), 6.73 – 6.67 (m, 2H), 3.79 (s, 3H), 3.73 (s, 2H), 3.63 (s, 2H), 0.91 (s, 9H), 0.85 (s, 9H).
^{13}**C NMR** (75 MHz, $CDCl_3$): δ 160.67, 158.05, 148.29, 139.30, 136.02, 134.46, 132.28, 131.10, 129.88, 129.31, 129.23, 129.11, 127.89, 127.48, 113.74, 79.91, 79.41, 55.37, 31.74, 31.49, 26.09, 25.88.

HRMS (ESI$^+$): m/z calc'd for **4c** $C_{33}H_{40}O_7S_2Na$ $[M+Na]^+$: 635.2108, found: 635.2108.
mp: 119.2 – 121.4.

Dineopentyl (1*E*,3*E*)-4-(3,4-dimethoxyphenyl)-2,4-diphenylbuta-1,3-diene-1,3-disulfonate (4d)

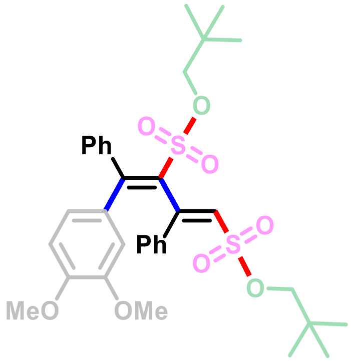

Following the general procedure B. neopentyl 2-phenylethyne-1-sulfonate (**3ah**) (25.2 mg, 0.1 mmol), (3,4-dimethoxyphenyl)boronic acid (54.6 mg, 0.3 mmol, 3 equiv.), $Cu(OAc)_2$ (1.8 mg), and MeOH (0.5 mL) were used. Purification via column chromatography on silica gel (cyclohexane: ethyl acetate = 90:10, v/v) afforded **4d** as a yellow oil; Yield 93% (30 mg).

NMR Spectroscopy (see spectra):
^{1}**H NMR** (400 MHz, $CDCl_3$): δ 7.41 – 7.30 (m, 7H), 7.26 – 7.17 (m, 3H), 6.98 (s, 1H), 6.67 (d, J = 8.4 Hz, 1H), 6.51 (dd, J = 8.4, 2.1 Hz, 1H), 6.35 (d, J = 2.1 Hz, 1H), 3.85 (s, 3H), 3.74 (s, 2H), 3.65 (s, 3H), 3.64 (s, 2H), 0.91 (s, 9H), 0.84 (s, 9H).
^{13}**C NMR** (101 MHz, $CDCl_3$): δ 158.09, 150.24, 148.62, 148.28, 139.09, 136.15, 134.56, 132.44, 129.63, 129.29, 129.11, 127.89, 127.49, 123.13, 112.25, 110.53, 79.94, 79.42, 55.95, 55.81, 31.73, 31.47, 26.08, 25.85.

HRMS (ESI$^+$): m/z calc'd for **4d** $C_{34}H_{42}O_8S_2Na$ $[M+Na]^+$: 665.2213, found: 665.2218.

Dineopentyl (1*E*,3*E*)-4-(3,4-dimethylphenyl)-2,4-diphenylbuta-1,3-diene-1,3-disulfonate (4e)

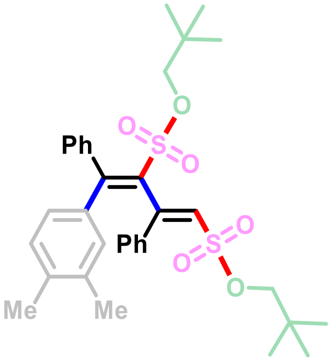

Following the general procedure B. neopentyl 2-phenylethyne-1-sulfonate (**3ah**) (25.2 mg, 0.1 mmol), (3,4-dimethylphenyl)boronic acid (45 mg, 0.3 mmol, 3 equiv.), $Cu(OAc)_2$ (1.8 mg), and MeOH (0.5 mL) were used. Purification via column chromatography on silica gel (cyclohexane: ethyl acetate = 94:6, v/v) afforded **4e** as a colorless solid; Yield 88% (27 mg).

NMR Spectroscopy (see spectra):
^{1}**H NMR** (400 MHz, $CDCl_3$): δ 7.42 – 7.29 (m, 6H), 7.30 – 7.21 (m, 4H), 7.21 – 7.17 (m, 2H), 6.94 – 6.83 (m, 3H), 6.54 – 6.45 (m, 2H), 3.72 (s, 2H), 3.55 (s, 2H), 2.17 (s, 6H), 0.90 (s, 9H), 0.81 (s, 9H).
^{13}**C NMR** (101 MHz, $CDCl_3$): δ 157.85, 147.85, 139.55, 138.72, 138.00, 137.55, 135.69, 130.57, 129.79, 129.46, 129.33, 129.17, 129.07, 128.96, 128.60, 128.54, 127.96, 127.47, 126.49, 79.84, 79.53, 31.72, 31.43, 26.93, 21.09, 21.06.

HRMS (ESI$^+$): m/z calc'd for **4e** $C_{34}H_{42}O_6S_2Na$ $[M+Na]^+$: 633.2315, found: 633.2318.
mp: 100.0 – 102.2 °C.

Dineopentyl (1*E*,3*E*)-4-([1,1'-biphenyl]-4-yl)-2,4-diphenylbuta-1,3-diene-1,3-disulfonate (4f)

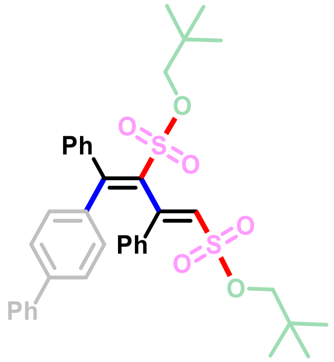

Following the general procedure B. neopentyl 2-phenylethyne-1-sulfonate (**3ah**) (25.2 mg, 0.1 mmol), [1,1'-biphenyl]-4-ylboronic acid (59 mg, 0.3 mmol, 3 equiv.), $Cu(OAc)_2$ (1.8 mg), and MeOH (0.5 mL) were used. Purification via column chromatography on silica gel (cyclohexane: ethyl acetate = 94:6, v/v) afforded **4f** as a colorless solid; Yield 70% (22 mg).

NMR Spectroscopy (see spectra):
^{1}H NMR (400 MHz, $CDCl_3$): δ 7.56 – 7.52 (m, 2H), 7.49 – 7.44 (m, 2H), 7.43 – 7.37 (m, 8H), 7.35 – 7.31 (m, 2H), 7.27 – 7.22 (m, 1H), 7.20 – 7.15 (m, 2H), 7.04 – 6.96 (m, 3H), 3.78 (s, 2H), 3.64 (s, 2H), 0.93 (s, 9H), 0.83 (s, 9H).
^{13}C NMR (101 MHz, $CDCl_3$): δ 157.62, 147.81, 142.16, 139.89, 138.83, 138.71, 135.88, 129.96, 129.46, 129.37, 129.28, 128.92, 128.88, 128.05, 127.95, 127.53, 127.04, 126.92, 79.94, 79.66, 31.76, 31.47, 26.07, 25.85.

HRMS (ESI$^+$): m/z calc'd for **4f** $C_{38}H_{42}O_6S_2Na$ $[M+Na]^+$: 681.2315, found: 681.2325.
mp: 128.3– 129.7 °C.

Dineopentyl (1*E*,3*E*)-2,4-diphenyl-4-(4-vinylphenyl)buta-1,3-diene-1,3-disulfonate (4g)

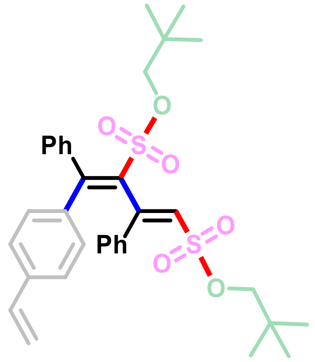

Following the general procedure B. neopentyl 2-phenylethyne-1-sulfonate (**3ah**) (25.2 mg, 0.1 mmol), (4-vinylphenyl)boronic acid (44 mg, 0.3 mmol, 3 equiv.), $Cu(OAc)_2$ (1.8 mg), and MeOH (0.5 mL) were used. Purification via column chromatography on silica gel (cyclohexane: ethyl acetate = 94:6, v/v) afforded **4g** as a colorless oil; Yield 52% (16 mg).

NMR Spectroscopy (see spectra):
^{1}H NMR (400 MHz, $CDCl_3$): δ 7.39 – 7.30 (m, 7H), 7.28 – 7.16 (m, 5H), 6.94 – 6.87 (m, 3H), 6.63 (dd, J = 17.6, 10.9 Hz, 1H), 5.74 (d, J = 17.6 Hz, 1H), 5.31 (d, J = 11.2 Hz, 1H), 3.72 (s, 2H), 3.56 (s, 2H), 0.90 (s, 9H), 0.82 (s, 9H).
^{13}C NMR (101 MHz, $CDCl_3$): δ 157.37, 147.79, 139.09, 138.78, 138.47, 135.83, 135.77, 135.64, 129.95, 129.43, 129.39, 129.27, 129.23, 128.83, 128.01, 127.57, 126.06, 115.64, 79.91, 79.62, 31.73, 31.45, 26.04, 25.85.

HRMS (ESI$^+$): m/z calc'd for **4g** $C_{34}H_{40}O_6S_2Na$ $[M+Na]^+$: 631.2159, found: 631.2163.
mp: 133.8 – 135 °C.

Dineopentyl (1*E*,3*E*)-2,4-diphenyl-4-(3-(trifluoromethyl)phenyl)buta-1,3-diene-1,3-disulfonate (4h)

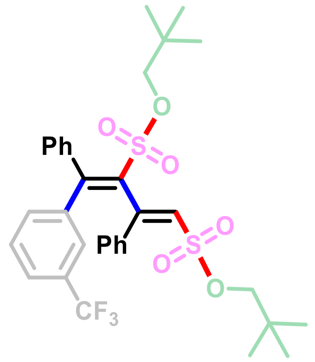

Following the general procedure B. neopentyl 2-phenylethyne-1-sulfonate (**3ah**) (25.2 mg, 0.1 mmol), (3-(trifluoromethyl)phenyl)boronic acid (57 mg, 0.3 mmol, 3 equiv.), $Cu(OAc)_2$ (1.8 mg), and MeOH (0.5 mL) were used. Purification via column chromatography on silica gel (cyclohexane: ethyl acetate = 94:6, v/v) afforded **4h** as a yellow oil; Yield 21% (7 mg).

NMR Spectroscopy (see spectra):
^{1}H NMR (400 MHz, $CDCl_3$): δ 7.49 (d, J = 7.7 Hz, 1H), 7.41 – 7.32 (m, 6H), 7.27 – 7.14 (m, 6H), 6.97 (s, 1H), 6.96 – 6.90 (m, 1H), 3.78 (s, 2H), 3.67 (s, 2H), 0.91 (s, 9H), 0.84 (s, 9H).

^{13}C NMR (101 MHz, $CDCl_3$): δ 155.77, 146.99, 140.48, 137.94, 137.50, 135.66, 131.76, 131.10, 130.77, 130.16, 129.66, 129.57, 129.11, 128.98, 128.61, 128.30, 127.75, 125.63, 125.17, 124.68, 121.97, 80.03, 80.00, 31.78, 31.51, 26.01, 25.84.
^{19}F NMR (282 MHz, $CDCl_3$): δ -62.80 (s, 3F, CF_3).

HRMS (ESI$^+$): m/z calc'd for **4h** $C_{33}H_{37}F_3O_6S_2Na$ $[M+Na]^+$: 673.1876, found: 673.1878.

Dineopentyl (1*E*,3*E*)-4-(3-bromophenyl)-2,4-diphenylbuta-1,3-diene-1,3-disulfonate (4i)

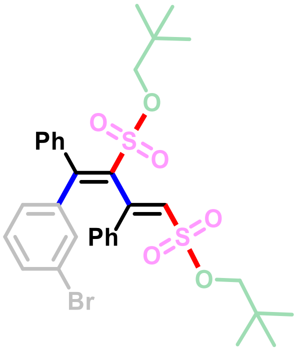

Following the general procedure B. neopentyl 2-phenylethyne-1-sulfonate (**3ah**) (25.2 mg, 0.1 mmol), (3-bromophenyl)boronic acid (60 mg, 0.3 mmol, 3 equiv.), $Cu(OAc)_2$ (1.8 mg), and MeOH (0.5 mL) were used. Purification via column chromatography on silica gel (cyclohexane: ethyl acetate = 94:6, v/v) afforded **4i** as a colorless solid; Yield 75% (25 mg).

NMR Spectroscopy (see spectra):
^{1}H NMR (400 MHz, $CDCl_3$): δ 7.44 – 7.36 (m, 4H), 7.36 – 7.27 (m, 6H), 7.28 – 7.23 (m, 2H), 6.94 (s, 1H), 6.69 (tt, J = 8.7, 2.3 Hz, 1H), 6.45 – 6.38 (m, 2H), 3.75 (s, 2H), 3.69 (s, 2H), 0.90 (s, 9H), 0.86 (s, 9H).
^{13}C NMR (101 MHz, $CDCl_3$): δ 155.69, 147.23, 141.56, 138.04, 137.13, 135.57, 131.96, 131.30, 129.92, 129.64, 129.46, 129.29, 128.60, 128.23, 127.77, 127.20, 122.45, 80.00, 79.92, 31.77, 31.52, 26.02, 25.88.

HRMS (ESI$^+$): m/z calc'd for **4i** $C_{32}H_{37}BrO_6S_2Na$ $[M+Na]^+$: 683.1107 (^{79}Br), found: 683.1127.
mp: 110.9 – 112.1 °C.

Dineopentyl (1*E*,3*E*)-4-(4-fluorophenyl)-2,4-diphenylbuta-1,3-diene-1,3-disulfonate (4j)

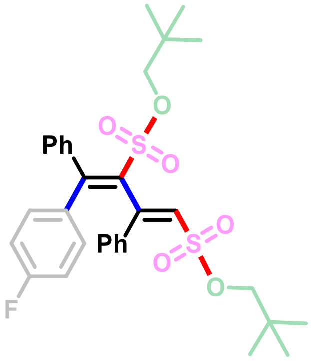

Following the general procedure B. neopentyl 2-phenylethyne-1-sulfonate (**3ah**) (25.2 mg, 0.1 mmol), (4-fluorophenyl)boronic acid (42 mg, 0.3 mmol, 3 equiv.), $Cu(OAc)_2$ (1.8 mg), and MeOH (0.5 mL) were used. Purification via column chromatography on silica gel (cyclohexane: ethyl acetate = 94:6, v/v) afforded **4j** as a colorless solid; Yield 65% (19 mg).

NMR Spectroscopy (see spectra):
^{1}H NMR (300 MHz, $CDCl_3$): δ 7.42 – 7.34 (m, 3H), 7.33 – 7.17 (m, 7H), 6.95 (s, 1H), 6.93 – 6.81 (m, 4H), 3.74 (s, 2H), 3.64 (s, 2H), 0.91 (s, 9H), 0.84 (s, 9H).
^{13}C NMR (75 MHz, $CDCl_3$): δ 156.73, 147.57, 138.73, 136.15, 135.79, 131.05, 130.94, 130.14, 129.51, 129.40, 129.24, 128.83, 128.09, 127.66, 115.67, 115.38, 79.99, 79.71, 31.76, 31.50, 26.05, 25.86.
^{19}F NMR (282 MHz, $CDCl_3$): δ -110.57 (ddd, J = 13.7, 7.6, 5.9 Hz, 1F).

HRMS (ESI$^+$): m/z calc'd for **4j** $C_{32}H_{37}FO_6S_2Na$ $[M+Na]^+$: 623.1908, found: 623.1908.
mp: 139.5 – 140.7 °C.

Dineopentyl (1*E*,3*E*)-4-(4-chlorophenyl)-2,4-diphenylbuta-1,3-diene-1,3-disulfonate (4k)

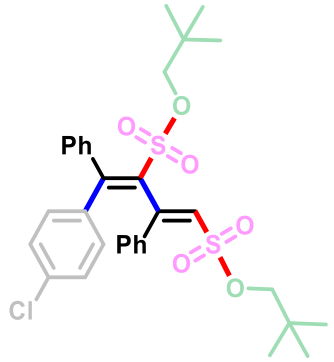

Following the general procedure B. neopentyl 2-phenylethyne-1-sulfonate (**3ah**) (25.2 mg, 0.1 mmol), (4-chlorophenyl)boronic acid (47 mg, 0.3 mmol, 3 equiv.), $Cu(OAc)_2$ (1.8 mg), and MeOH (0.5 mL) were used. Purification via column chromatography on silica gel (cyclohexane: ethyl acetate = 94:6, v/v) afforded **4k** as a colorless solid; Yield 96% (29 mg).

NMR Spectroscopy (see spectra):
^{1}H NMR (400 MHz, $CDCl_3$): δ 7.41 – 7.35 (m, 3H), 7.33 – 7.19 (m, 8H), 7.18 – 7.12 (m, 2H), 6.93 (s, 1H), 6.87 – 6.82 (m, 2H), 3.73 (s, 2H), 3.62 (s, 2H), 0.90 (s, 9H), 0.84 (s, 9H).
^{13}C NMR (101 MHz, $CDCl_3$): δ 156.32, 147.35, 138.42, 138.18, 136.52, 135.63, 135.48, 130.13, 129.59, 129.41, 129.34, 128.74, 128.61, 128.13, 127.70, 79.99, 79.78, 31.74, 31.47, 26.02, 25.84.

HRMS (ESI^{+}): m/z calc'd for **4k** $C_{32}H_{37}ClO_6S_2Na$ $[M+Na]^+$: 639.1612 (^{35}Cl), found: 639.1620.
mp: 148.1 – 150.7 °C.

Dineopentyl (1*E*,3*E*)-4-(4-iodophenyl)-2,4-diphenylbuta-1,3-diene-1,3-disulfonate (4l)

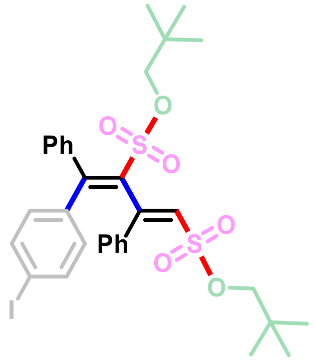

Following the general procedure B. neopentyl 2-phenylethyne-1-sulfonate (**3ah**) (25.2 mg, 0.1 mmol), (4-iodophenyl)boronic acid (74 mg, 0.3 mmol, 3 equiv.), $Cu(OAc)_2$ (1.8 mg), and MeOH (0.5 mL) were used. Purification via column chromatography on silica gel (cyclohexane: ethyl acetate = 94:6, v/v) afforded **4l** as a colorless solid; Yield 72% (25 mg).

NMR Spectroscopy (see spectra):
^{1}H NMR (400 MHz, $CDCl_3$): δ 7.52 (d, *J* = 8.4 Hz, 2H), 7.40 – 7.35 (m, 3H), 7.32 – 7.19 (m, 7H), 6.93 (s, 1H), 6.64 (d, *J* = 8.4 Hz, 2H), 3.73 (s, 2H), 3.62 (s, 2H), 0.90 (s, 9H), 0.84 (s, 9H).
^{13}C NMR (101 MHz, $CDCl_3$): δ 156.40, 147.34, 139.27, 138.27, 137.52, 136.48, 135.61, 130.36, 130.08, 129.60, 129.42, 129.36, 128.72, 128.15, 127.71, 95.51, 79.99, 79.80, 31.75, 31.49, 26.03, 25.89.

HRMS (ESI^{+}): m/z calc'd for **4l** $C_{32}H_{37}IO_6S_2Na$ $[M+Na]^+$: 731.0968, found: 731.0989.
mp: 115.6 – 117.3 °C.

Dineopentyl (1*E*,3*E*)-4-(3,5-difluorophenyl)-2,4-diphenylbuta-1,3-diene-1,3-disulfonate (4m)

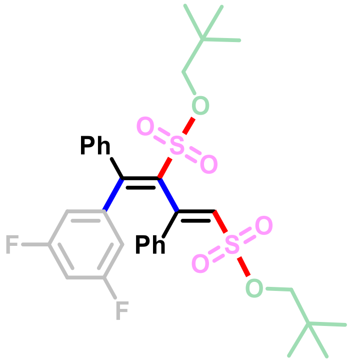

Following the general procedure B. neopentyl 2-phenylethyne-1-sulfonate (**3ah**) (25.2 mg, 0.1 mmol), (3,5-difluorophenyl)boronic acid (47.5 mg, 0.3 mmol, 3 equiv.), $Cu(OAc)_2$ (1.8 mg), and MeOH (0.5 mL) were used. Purification via column chromatography on silica gel (cyclohexane: ethyl acetate = 94:6, v/v) afforded **4m** as a colorless oil; Yield 72% (22 mg).

NMR Spectroscopy (see spectra):
^{1}H NMR (400 MHz, $CDCl_3$): δ 7.44 – 7.36 (m, 4H), 7.36 – 7.27 (m, 6H), 7.28 – 7.23 (m, 2H), 6.94 (s, 1H), 6.69 (tt, *J* = 8.7, 2.3 Hz, 1H), 6.45 – 6.38 (m, 2H), 3.75 (s, 2H), 3.69 (s, 2H), 0.90 (s, 9H), 0.86 (s, 9H).
^{13}C NMR (101 MHz, $CDCl_3$): δ 162.61 (dd, *J* = 239.8, 12.6 Hz), 154.48, 146.85, 142.42 (t, *J* = 9.1 Hz), 137.75, 137.60, 135.44, 129.98, 129.86, 129.60, 129.34, 128.42, 128.34, 127.89, 111.89, 111.82, 111.70, 111.63, 104.33 (t, *J* = 25.4 Hz), 80.13, 80.04, 31.77, 31.52, 25.98, 25.83.
^{19}F NMR (282 MHz, $CDCl_3$): δ -108.07 – -108.21 (m, 2F).

HRMS (ESI$^+$): m/z calc'd for **4m** $C_{32}H_{36}F_2O_6S_2Na$ [M+Na]$^+$: 639.2277, found: 639.2281.

Methyl 4-((1*E*,3*E*)-2,4-bis((neopentyloxy)sulfonyl)-1,3-diphenylbuta-1,3-dien-1-yl)benzoate (4n)

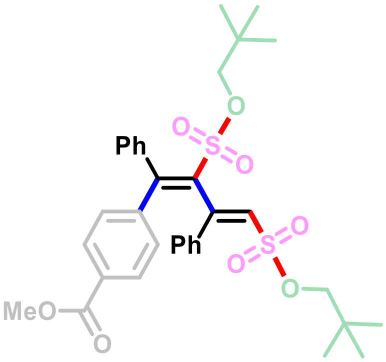

Following the general procedure B. neopentyl 2-phenylethyne-1-sulfonate (**3ah**) (25.2 mg, 0.1 mmol), (4-(methoxycarbonyl)phenyl)boronic acid (54 mg, 0.3 mmol, 3 equiv.), $Cu(OAc)_2$ (1.8 mg), and MeOH (0.5 mL) were used. Purification via column chromatography on silica gel (cyclohexane: ethyl acetate = 94:6, v/v) afforded **4n** as a colorless oil; Yield 76% (24 mg).

NMR Spectroscopy (see spectra):
^{1}H NMR (400 MHz, $CDCl_3$): δ 7.84 (d, *J* = 8.4 Hz, 2H), 7.40 – 7.31 (m, 5H), 7.29 – 7.24 (m, 3H), 7.21 – 7.15 (m, 2H), 7.00 (d, *J* = 8.4 Hz, 2H), 6.92 (s, 1H), 3.92 (s, 3H), 3.74 (s, 2H), 3.58 (s, 2H), 0.89 (s, 9H), 0.82 (s, 9H).
^{13}C NMR (101 MHz, $CDCl_3$): δ 166.14, 156.16, 147.16, 144.02, 138.09, 137.11, 135.41, 131.82, 130.32, 130.11, 129.67, 129.54, 129.42, 129.38, 128.60, 128.56, 128.21, 127.75, 115.20, 79.98, 79.91, 52.40, 31.75, 31.46, 26.01, 25.81.

HRMS (ESI$^+$): m/z calc'd for **4n** $C_{34}H_{40}O_8S_2Na$ [M+Na]$^+$: 663.2057, found: 663.2067.

Dineopentyl (1*E*,3*E*)-4-(3-formylphenyl)-2,4-diphenylbuta-1,3-diene-1,3-disulfonate (4o)

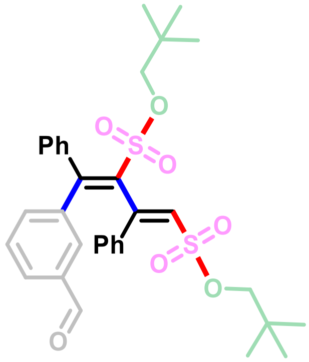

Following the general procedure B. neopentyl 2-phenylethyne-1-sulfonate (**3ah**) (25.2 mg, 0.1 mmol), (3-formylphenyl)boronic acid (45 mg, 0.3 mmol, 3 equiv.), $Cu(OAc)_2$ (1.8 mg), and MeOH (0.5 mL) were used. Purification via column chromatography on silica gel (cyclohexane: ethyl acetate = 94:6, v/v) afforded **4o** as a colorless oil; Yield 74% (22 mg).

NMR Spectroscopy (see spectra):
^{1}H NMR (400 MHz, $CDCl_3$): δ 9.79 (s, 1H), 7.76 (dt, *J* = 7.7, 1.4 Hz, 1H), 7.43 – 7.31 (m, 7H), 7.27 – 7.20 (m, 5H), 7.18 – 7.13 (m, 2H), 6.96 (s, 1H), 3.77 (s, 2H), 3.66 (s, 2H), 0.90 (s, 9H), 0.83 (s, 9H).
^{13}C NMR (101 MHz, $CDCl_3$): δ 191.01, 155.80, 147.03, 140.71, 137.99, 137.31, 136.25, 135.70, 134.07, 130.15, 129.71, 129.66, 129.62, 129.55, 129.22, 128.64, 128.30, 127.77, 80.06, 79.98, 31.78, 31.51, 26.01, 25.85.

HRMS (ESI$^+$): m/z calc'd for **4o** $C_{33}H_{38}O_7S_2Na$ [M+Na]$^+$: 633.1951, found: 633.1962.

Dineopentyl (1*E*,3*E*)-4-(2-((*E*)-(methoxyimino)methyl)phenyl)-2,4-diphenylbuta-1,3-diene-1,3-disulfonate (4p)

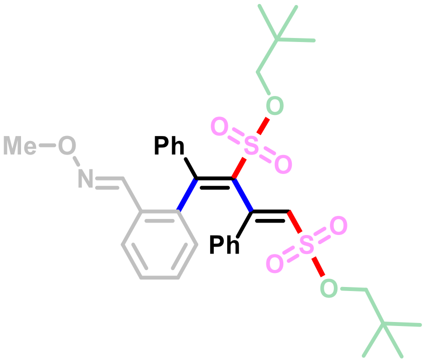

Following the general procedure B. neopentyl 2-phenylethyne-1-sulfonate (**3ah**) (25.2 mg, 0.1 mmol), (*E*)-(2-((methoxyimino)methyl)phenyl)boronic acid (54 mg, 0.3 mmol, 3 equiv.), $Cu(OAc)_2$ (1.8 mg), DCM (0.1 mL) and MeOH (0.5 mL) were used. Purification via column chromatography on silica gel (cyclohexane: ethyl acetate = 80:20, v/v) afforded **4p** as a colorless oil; Yield 46% (15 mg).

NMR Spectroscopy (see spectra):
^{1}H NMR (400 MHz, $CDCl_3$): δ 7.85 (s, 1H), 7.58 (d, *J* = 7.9 Hz, 1H), 7.47 – 7.42 (m, 2H), 7.37 – 7.32 (m, 3H), 7.27 – 7.21 (m, 2H), 7.16 (d, *J* = 4.3 Hz, 4H), 7.07 (t, *J* = 7.6 Hz, 1H), 6.80 (s, 1H), 6.66 (d, *J* = 7.8 Hz, 1H), 3.98 (s, 3H), 3.85 – 3.72 (m, 2H), 3.65 (s, 2H), 0.89 (s, 9H), 0.84 (s, 9H).
^{13}C NMR (101 MHz, $CDCl_3$): δ 154.97, 147.49, 145.79, 138.07, 137.65, 136.87, 135.46, 129.48, 129.44, 129.23, 129.20, 129.05, 128.99, 128.96, 128.90, 128.01, 127.62, 127.49, 79.95, 79.88, 62.17, 31.76, 31.51, 25.98, 25.87.

HRMS (ESI+): m/z calc'd for **4p** $C_{34}H_{41}NO_7S_2Na$ $[M+Na]^+$: 662.2217, found: 662.2217.

Dineopentyl (1*E*,3*E*)-4-(1-methyl-1*H*-indol-5-yl)-2,4-diphenylbuta-1,3-diene-1,3-disulfonate (4q)

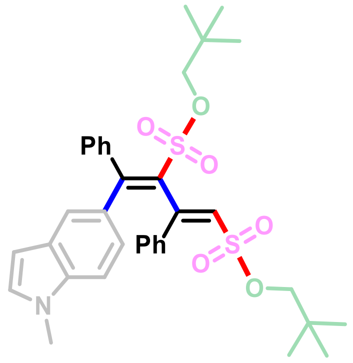

Following the general procedure B. neopentyl 2-phenylethyne-1-sulfonate (**3ah**) (25.2 mg, 0.1 mmol), (1-methyl-1*H*-indol-5-yl)boronic acid (52 mg, 0.3 mmol, 3 equiv.), $Cu(OAc)_2$ (1.8 mg), and MeOH (0.5 mL) were used. Purification via column chromatography on silica gel (cyclohexane: ethyl acetate = 80:20, v/v) afforded **4q** as a brown solid; Yield 84% (26 mg).

NMR Spectroscopy (see spectra):
^{1}H NMR (400 MHz, $CDCl_3$): δ 7.36 (d, J = 2.0 Hz, 5H), 7.28 – 7.24 (m, 3H), 7.20 – 7.15 (m, 1H), 7.13 – 7.06 (m, 3H), 7.05 (d, J = 3.1 Hz, 1H), 6.99 (s, 1H), 6.79 (dd, J = 8.6, 1.7 Hz, 1H), 6.41 (dd, J = 3.1, 0.9 Hz, 1H), 3.76 (s, 3H), 3.74 (s, 2H), 3.46 (s, 2H), 0.93 (s, 9H), 0.76 (s, 9H).
^{13}C NMR (101 MHz, $CDCl_3$): δ 159.84, 148.76, 139.98, 136.85, 135.94, 134.05, 131.14, 130.13, 129.77, 129.24, 129.18, 129.13, 129.03, 128.08, 127.75, 127.29, 123.12, 109.02, 102.06, 79.74, 79.29, 32.92, 31.73, 31.35, 26.12, 25.76.

HRMS (ESI+): m/z calc'd for **4q** $C_{35}H_{41}NO_6S_2Na$ $[M+Na]^+$: 658.2268, found: 658.2260.
mp: 145.2 – 146.3 °C.

***Tert*-Butyl 3-((1*E*,3*E*)-2,4-bis((neopentyloxy)sulfonyl)-1,3-diphenylbuta-1,3-dien-1-yl)-1*H*-indole-1-carboxylate (4r)**

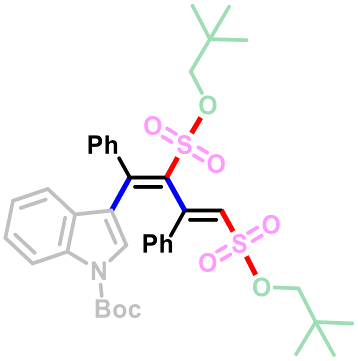

Following the general procedure B. neopentyl 2-phenylethyne-1-sulfonate (**3ah**) (25.2 mg, 0.1 mmol), (1-(tert-butoxycarbonyl)-1*H*-indol-3-yl)boronic acid (78 mg, 0.3 mmol, 3 equiv.), $Cu(OAc)_2$ (1.8 mg), and MeOH (0.5 mL) were used. Purification via column chromatography on silica gel (cyclohexane: ethyl acetate = 85:15, v/v) afforded **4r** as a yellow oil; Yield 61% (22 mg).

NMR Spectroscopy (see spectra):
^{1}H NMR (400 MHz, $CDCl_3$): δ 8.07 (d, J = 8.4 Hz, 1H), 7.56 (s, 1H), 7.51 (dd, J = 7.7, 1.8 Hz, 2H), 7.43 – 7.33 (m, 5H), 7.25 – 7.16 (m, 1H), 7.14 – 7.08 (m, 3H), 7.00 (s, 1H), 6.97 – 6.92 (m, 1H), 6.55 (d, J = 8.0 Hz, 1H), 3.75 (s, 2H), 3.54 (s, 2H), 1.70 (s, 9H), 0.90 (s, 9H), 0.78 (s, 9H).
^{13}C NMR (101 MHz, $CDCl_3$): δ 150.54, 148.85, 148.12, 137.76, 135.68, 135.52, 135.03, 129.81, 129.37, 129.11, 128.19, 128.08, 127.67, 127.50, 124.94, 123.16, 119.98, 119.69, 115.11, 85.03, 79.87, 79.57, 31.89, 31.72, 31.40, 28.03, 26.04, 25.80.

HRMS (ESI+): m/z calc'd for **4r** $C_{39}H_{47}NO_8S_2Na[M+Na]^+$: 744.2635, found: 744.2631.

Dineopentyl (1*E*,3*E*)-2,4-diphenyl-4-(9-phenyl-9*H*-carbazol-3-yl)buta-1,3-diene-1,3-disulfonate (4s)

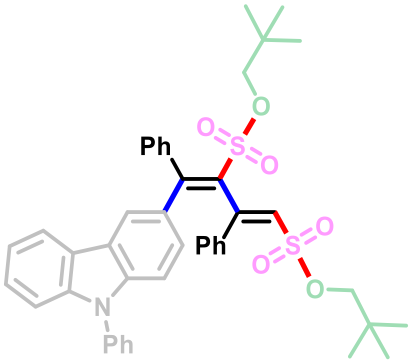

Following the general procedure B. neopentyl 2-phenylethyne-1-sulfonate (**3ah**) (25.2 mg, 0.1 mmol), (9-phenyl-9*H*-carbazol-3-yl)boronic acid (86 mg, 0.3 mmol, 3 equiv.), $Cu(OAc)_2$ (1.8 mg), and MeOH (0.5 mL) were used. Purification via column chromatography on silica gel (cyclohexane: ethyl acetate = 85:15, v/v) afforded **4s** as a brown oil; Yield 64% (24 mg).

NMR Spectroscopy (see spectra):

^{1}**H NMR** (400 MHz, $CDCl_3$): δ 8.03 (d, J = 7.7 Hz, 1H), 7.78 (d, J = 1.3 Hz, 1H), 7.65 – 7.60 (m, 2H), 7.53 – 7.48 (m, 3H), 7.47 – 7.38 (m, 7H), 7.33 – 7.29 (m, 3H), 7.19 – 7.12 (m, 2H), 7.12 – 7.04 (m, 3H), 6.97 – 6.94 (m, 1H), 3.79 (s, 2H), 3.56 (s, 2H), 0.95 (s, 9H), 0.74 (s, 9H).
^{13}**C NMR** (101 MHz, $CDCl_3$): δ 158.93, 148.48, 141.40, 141.09, 139.78, 136.98, 136.05, 134.38, 131.57, 130.04, 129.99, 129.34, 129.28, 129.23, 127.94, 127.88, 127.70, 127.37, 126.90, 126.69, 123.11, 122.88, 122.28, 120.74, 120.62, 110.05, 109.57, 79.82, 79.38, 31.78, 31.37, 26.13, 25.75.

HRMS (ESI$^+$): m/z calc'd for **4s** $C_{44}H_{45}NO_6S_2Na[M+Na]^+$: 770.2581, found: 770.2578.

Dineopentyl (1*E*,3*E*)-2,4,5-triphenylhexa-1,3,5-triene-1,3-disulfonate (4t)

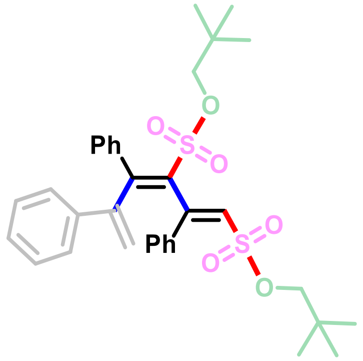

Following the general procedure B. neopentyl 2-phenylethyne-1-sulfonate (**3ah**) (25.2 mg, 0.1 mmol), 1-phenylvinylboronic acid (44 mg, 0.3 mmol, 3 equiv.), $Cu(OAc)_2$ (1.8 mg), and MeOH (0.5 mL) were used. Purification via column chromatography on silica gel (cyclohexane: ethyl acetate = 95:5, v/v) afforded **4t** as a colorless solid; Yield 68% (20 mg).

NMR Spectroscopy (see spectra):
^{1}**H NMR** (300 MHz, $CDCl_3$): δ 7.68 – 7.61 (m, 2H), 7.44 – 7.36 (m, 3H), 7.33 – 7.29 (m, 3H), 7.27 (d, J = 3.2 Hz, 2H), 7.16 – 7.03 (m, 3H), 6.87 – 6.70 (m, 3H), 5.71 (s, 1H), 5.33 (s, 1H), 3.75 – 3.63 (m, 4H), 0.88 (s, 9H), 0.84 (s, 9H).
^{13}**C NMR** (75 MHz, $CDCl_3$): δ 156.55, 148.06, 147.24, 137.11, 136.50, 136.19, 135.55, 130.33, 129.73, 129.29, 129.19, 128.70, 128.21, 128.16, 127.90, 127.80, 126.65, 119.07, 79.96, 79.80, 31.73, 31.51, 26.01, 25.88.

HRMS (ESI$^+$): m/z calc'd for **4t** $C_{34}H_{40}O_6S_2Na[M+Na]^+$: 631.2159, found: 631.2148.
mp: 113.0 – 116.1 °C.

Dineopentyl (1*E*,3*Z*)-2,4-bis(4-fluorophenyl)-4-(4-methoxyphenyl)buta-1,3-diene-1,3-disulfonate (4u)

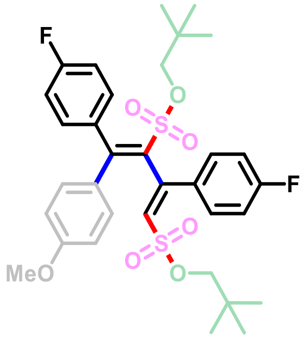

Following the general procedure B. neopentyl 2-(4-fluorophenyl)ethyne-1-sulfonate (**3ag**) (27 mg, 0.1 mmol), (4-methoxyphenyl)boronic acid (36.6 mg, 0.3 mmol, 3 equiv.), $Cu(OAc)_2$ (1.8 mg), and MeOH (0.5 mL) were used. Purification via column chromatography on silica gel (cyclohexane: ethyl acetate = 85:15, v/v) afforded **4u** as a colorless oil; Yield 45% (15 mg).

NMR Spectroscopy (see spectra):
^{1}**H NMR** (300 MHz, $CDCl_3$): δ 7.31 – 7.24 (m, 4H), 7.13 – 7.00 (m, 2H), 6.90 – 6.81 (m, 4H), 6.72 (d, J = 8.9 Hz, 2H), 3.80 (s, 3H), 3.77 (s, 2H), 3.67 (s, 2H), 0.93 (s, 9H), 0.87 (s, 9H).
^{13}**C NMR** (151 MHz, $CDCl_3$): δ 164.20, 163.90, 162.54, 162.23, 160.97, 157.30, 147.11, 135.06, 135.03, 134.16, 133.06, 132.17, 132.15, 132.07, 131.44, 131.38, 131.26, 131.19, 131.14, 130.19, 115.22, 115.07, 114.76, 114.62, 113.86, 80.05, 79.54, 55.44, 31.79, 31.54, 26.07, 25.88.
^{19}**F NMR** (282 MHz, $CDCl_3$): δ -110.65 (tt, J = 8.6, 5.3 Hz, 1F), -110.80 (tt, J = 8.6, 5.2 Hz, 1F).

HRMS (ESI$^+$): m/z calc'd for **4u** $C_{33}H_{38}F_2O_7S_2Na[M+Na]^+$: 671.1919, found: 671.1912.
mp: 116.9 – 118.4 °C.

Dineopentyl (1*E*,3E)-4-(3-bromophenyl)-2,4-bis(4-bromophenyl)buta-1,3-diene-1,3-disulfonate (4v)

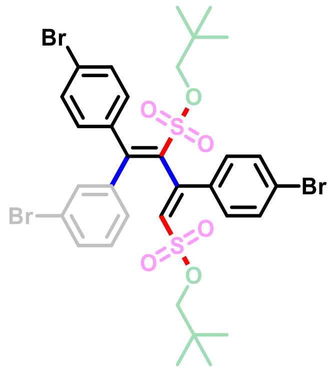

Following the general procedure B. neopentyl 2-(4-bromophenyl)ethyne-1-sulfonate (**3ca**) (33.1 mg, 0.1 mmol), (3-bromophenyl)boronic acid (60 mg, 0.3 mmol, 3 equiv.), $Cu(OAc)_2$ (1.8 mg), and MeOH (0.5 mL) were used. Purification via column chromatography on silica gel (cyclohexane: ethyl acetate = 85:15, v/v) afforded **4v** as a colorless oil; Yield 75% (31 mg).

NMR Spectroscopy (see spectra):
^{1}H NMR (300 MHz, $CDCl_3$): δ 7.52 (d, J = 8.5 Hz, 2H), 7.42 (dt, J = 8.1, 1.3 Hz, 1H), 7.35 (d, J = 8.6 Hz, 2H), 7.21 – 7.12 (m, 3H), 7.11 – 7.06 (m, 3H), 6.94 (s, 1H), 6.91 – 6.83 (m, 2H), 3.79 (s, 2H), 3.68 (s, 2H), 0.92 (s, 9H), 0.88 (s, 9H).
^{13}C NMR (75 MHz, $CDCl_3$): δ 154.85, 145.88, 140.92, 136.95, 136.64, 134.46, 132.38, 131.56, 131.34, 131.10, 130.61, 130.26, 130.17, 127.17, 124.44, 124.28, 123.87, 122.75, 118.80, 114.25, 80.20, 80.14, 31.82, 31.59, 25.99, 25.87.

HRMS (ESI$^+$): m/z calc'd for **4v** $C_{34}H_{40}O_6S_2K[M+K]^+$: 854.90570 (^{79}Br), found: 854.9043.
mp: 136.7 – 138.0 °C.

Neopentyl 2,2-diphenylethene-1-sulfonate (5a)

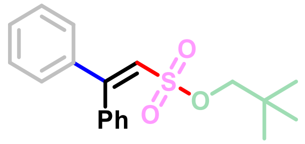

Following the general procedure C. neopentyl 2-phenylethyne-1-sulfonate (**3ah**) (25.2 mg, 0.1 mmol), phenylboronic acid (36.6 mg, 0.3 mmol, 3 equiv.), $Cu(OAc)_2$ (1.8 mg), and NMP (1 mL) were used. Purification via column chromatography on silica gel (cyclohexane: ethyl acetate = 95:5, v/v) afforded **5a** as a colorless solid; Yield 79% (26 mg).

NMR Spectroscopy (see spectra):
^{1}H NMR (400 MHz, $CDCl_3$): δ 7.48 – 7.36 (m, 8H), 7.32 – 7.28 (m, 2H), 6.76 (s, 1H), 3.75 (s, 2H), 0.94 (s, 9H).
^{13}C NMR (101 MHz, $CDCl_3$): δ 156.39, 139.36, 135.99, 130.42, 129.70, 129.45, 128.69, 128.53, 128.08, 121.58, 79.16, 31.64, 26.07.

HRMS (ESI$^+$): m/z calc'd for **5a** $C_{19}H_{22}O_3SNa[M+Na]^+$: 353.1182, found: 353.1179.
mp: 109.1 – 111.0 °C.

Neopentyl (*E*)-2-(3-cyanophenyl)-2-phenylethene-1-sulfonate (5b)

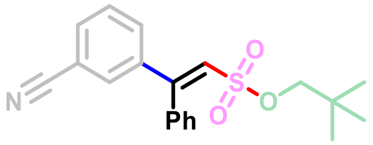

Following the general procedure C. neopentyl 2-phenylethyne-1-sulfonate (**3ah**) (25.2 mg, 0.1 mmol), (3-cyanophenyl)boronic acid (44 mg, 0.3 mmol, 3 equiv.), $Cu(OAc)_2$ (1.8 mg), and NMP (0.5 mL) were used. Purification via column chromatography on silica gel (cyclohexane: ethyl acetate = 95:5, v/v) afforded **5b** as a colorless solid; Yield 76% (27 mg).

NMR Spectroscopy (see spectra):
^{1}H NMR (400 MHz, $CDCl_3$): δ 7.73 (dt, J = 6.9, 1.8 Hz, 1H), 7.62 – 7.42 (m, 6H), 7.40 – 7.30 (m, 2H), 6.78 (s, 1H), 3.76 (s, 2H), 0.93 (s, 9H).
^{13}C NMR (101 MHz, $CDCl_3$): δ 153.69, 140.74, 134.85, 133.48, 132.42, 132.04, 130.08, 130.05, 129.70, 129.59, 128.49, 123.87, 117.97, 79.48, 31.67, 26.02.

HRMS (ESI$^+$): m/z calc'd for **5b** $C_{20}H_{21}NO_3SNa[M+Na]^+$: 378.1135, found: 378.1142.
mp: 85.8 – 87.7 °C.

Neopentyl (*E*)-2-phenyl-2-(3-(trifluoromethyl)phenyl)ethene-1-sulfonate (5c)

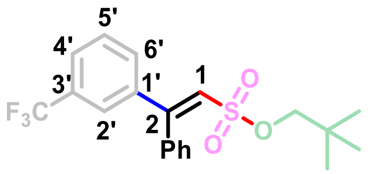

Following the general procedure C. neopentyl 2-phenylethyne-1-sulfonate (**3ah**) (25.2 mg, 0.1 mmol), (3-(trifluoromethyl)phenyl)boronic acid (57 mg, 0.3 mmol, 3 equiv.), $Cu(OAc)_2$ (1.8 mg), and NMP (0.5 mL) were used. Purification via column chromatography on silica gel (cyclohexane: ethyl acetate = 95:5, v/v) afforded **5c** as a colorless solid; Yield 55% (22 mg).

NMR Spectroscopy (see spectra):
^{1}**H NMR** (400 MHz, $CDCl_3$): δ 7.71 (d, *J* = 7.6 Hz, 1H), 7.57 – 7.44 (m, 6H), 7.41 – 7.36 (m, 2H), 6.79 (s, 1H), 3.77 (s, 2H), 0.93 (s, 9H).
^{13}**C NMR** (101 MHz, $CDCl_3$): δ 154.67, 140.33, 135.21, 131.86, 131.47, 131.14, 129.90, 129.66, 129.33, 128.35, 126.95 (q, *J* = 3.8 Hz), 125.01 (q, *J* = 3.8 Hz), 123.31, 122.29, 79.41, 31.66, 26.02.
^{19}**F NMR** (282 MHz, $CDCl_3$): δ -62.74 (s, 3F, CF_3).
E/*Z*-isomerism was determined via a *^{1}H,^{1}H-NOESY*-contact between 1-H and 2'-H, 6'-H, which is only possible in the (*E*)-configured molecule.

HRMS (ESI+): m/z calc'd for **5c** $C_{20}H_{21}F_3O_3SNa$ $[M+Na]^+$: 421.1056, found: 421.1051.
mp: 84.8 – 86.6 °C.

Neopentyl (*E*)-2-(4-methoxyphenyl)-2-phenylethene-1-sulfonate (5d)

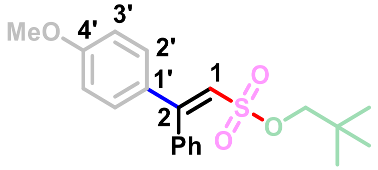

Following the general procedure C. neopentyl 2-phenylethyne-1-sulfonate (**3ah**) (25.2 mg, 0.1 mmol), (4-methoxyphenyl)boronic acid (36.6 mg, 0.3 mmol, 3 equiv.), $Cu(OAc)_2$ (1.8 mg), and NMP (0.5 mL) were used. Purification via column chromatography on silica gel (cyclohexane: ethyl acetate = 95:5, v/v) afforded **5d** as a colorless solid; Yield 69% (25 mg).

NMR Spectroscopy (see spectra):
^{1}**H NMR** (400 MHz, $CDCl_3$): δ 7.47 – 7.41 (m, 3H), 7.37 (dq, *J* = 6.8, 2.5 Hz, 2H), 7.26 – 7.21 (m, 2H), 6.89 (d, *J* = 8.9 Hz, 2H), 6.70 (s, 1H), 3.85 (s, 3H), 3.73 (s, 2H), 0.94 (s, 9H).
^{13}**C NMR** (101 MHz, $CDCl_3$): δ 161.57, 156.02, 136.18, 131.45, 130.09, 129.66, 129.31, 128.02, 119.35, 114.08, 78.95, 55.44, 31.63, 26.09.
E/*Z*-isomerism was determined via a *^{1}H,^{1}H-NOESY*-contact between 1-H and 2'-H, which is only possible in the (*E*)-configured molecule.

HRMS (ESI+): m/z calc'd for **5d** $C_{20}H_{24}O_4SNa$ $[M+Na]^+$: 383.1288, found: 383.1286.
mp: 109.5 – 111.1 °C.

Neopentyl (*E*)-2-phenyl-2-(4-vinylphenyl)ethene-1-sulfonate (5e)

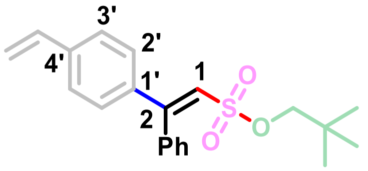

Following the general procedure C. neopentyl 2-phenylethyne-1-sulfonate (**3ah**) (25.2 mg, 0.1 mmol), (4-vinylphenyl)boronic acid (44 mg, 0.3 mmol, 3 equiv.), $Cu(OAc)_2$ (1.8 mg), and NMP (0.5 mL) were used. Purification via column chromatography on silica gel (cyclohexane: ethyl acetate = 95:5, v/v) afforded **5e** as a colorless solid; Yield 56% (20 mg).

NMR Spectroscopy (see spectra):
^{1}**H NMR** (400 MHz, $CDCl_3$): δ 7.48 – 7.37 (m, 7H), 7.28 – 7.22 (m, 2H), 6.87 – 6.61 (m, 2H), 5.84 (d, *J* = 17.6 Hz, 1H), 5.37 (d, *J* = 11.5 Hz, 1H), 3.75 (s, 2H), 0.94 (s, 9H).
^{13}**C NMR** (101 MHz, $CDCl_3$): δ 155.90, 139.71, 138.49, 135.90, 135.81, 129.68, 129.46, 128.80, 128.10, 126.44, 121.13, 115.87, 79.15, 31.65, 26.08.

E/*Z*-isomerism was determined via a *¹H,¹H-NOESY*-contact between 1-H and 2'-H, which is only possible in the (*E*)-configured molecule.

HRMS (ESI+): m/z calc'd for **5e** $C_{21}H_{24}O_3SNa$ $[M+Na]^+$: 379.1338, found: 379.1333.
mp: 110.8– 112.1 °C.

Neopentyl (*E*)-2-(2-((*E*)-(methoxyimino)methyl)phenyl)-2-phenylethene-1-sulfonate (5f)

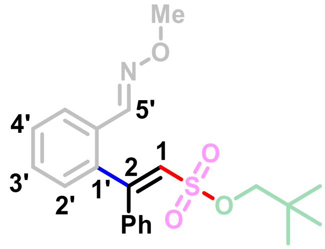

Following the general procedure C. neopentyl 2-phenylethyne-1-sulfonate (**3ah**) (25.2 mg, 0.1 mmol), (*E*)-(2-((methoxyimino)methyl)phenyl)boronic acid (54 mg, 0.3 mmol, 3 equiv.), Cu(OAc)₂ (1.8 mg), and NMP (0.5 mL) were used. Purification via column chromatography on silica gel (cyclohexane: ethyl acetate = 95:5, v/v) afforded **5f** as a colorless oil; Yield 67% (26 mg).

NMR Spectroscopy (see spectra):
¹H NMR (400 MHz, $CDCl_3$): δ 8.15 (s, 1H), 7.89 (dd, *J* = 7.2, 2.0 Hz, 1H), 7.48 – 7.33 (m, 8H), 7.21 (dd, *J* = 6.2, 1.5 Hz, 1H), 6.42 (s, 1H), 3.94 (s, 3H), 3.82 (s, 2H), 0.94 (s, 9H).
¹³C NMR (101 MHz, $CDCl_3$): δ 154.48, 146.33, 139.84, 136.22, 130.45, 130.15, 130.13, 129.81, 129.64, 129.54, 128.23, 127.20, 125.41, 79.64, 62.09, 31.67, 25.97.
E/*Z*-isomerism was determined via a *¹H,¹H-NOESY*-contact between 1-H and 5'-H, which is only possible in the (*E*)-configured molecule.

HRMS (ESI+): m/z calc'd for **5f** $C_{21}H_{25}NO_4SNa$ $[M+Na]^+$: 410.1397, found: 410.1390

Neopentyl (*E*)-2-phenyl-2-(9-phenyl-9*H*-carbazol-3-yl)ethene-1-sulfonate (5g)

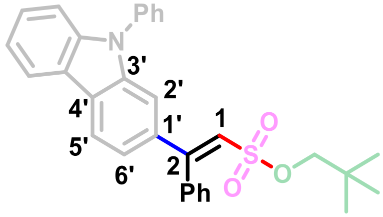

Following the general procedure C. neopentyl 2-phenylethyne-1-sulfonate (**3ah**) (25.2 mg, 0.1 mmol), (9-phenyl-9*H*-carbazol-3-yl)boronic acid (86 mg, 0.3 mmol, 3 equiv.), (44 mg, 0.3 mmol, 3 equiv.), Cu(OAc)₂ (1.8 mg), and NMP (0.5 mL) were used. Purification via column chromatography on silica gel (cyclohexane: ethyl acetate = 95:5, v/v) afforded **5g** as a colorless solid; Yield 83% (41 mg).

NMR Spectroscopy (see spectra):
¹H NMR (400 MHz, $CDCl_3$): δ 8.10 (d, *J* = 7.8 Hz, 1H), 8.07 (s, 1H), 7.68 – 7.63 (m, 2H), 7.59 – 7.41 (m, 10H), 7.39 – 7.31 (m, 3H), 6.87 (s, 1H), 3.80 (s, 2H), 0.97 (s, 9H).
¹³C NMR (101 MHz, $CDCl_3$): δ 157.41, 142.07, 141.59, 137.05, 136.71, 131.13, 130.07, 129.93, 129.41, 128.04, 127.05, 126.75, 126.59, 123.54, 123.01, 121.18, 120.69, 120.53, 119.43, 110.20, 109.89, 78.98, 31.68, 26.14.
E/*Z*-isomerism was determined via a *¹H,¹H-NOESY*-contact between 2'-H and 6'-H, which is only possible in the (*E*)-configured molecule.

HRMS (ESI+): m/z calc'd for **5g** $C_{31}H_{29}NO_3SNa$ $[M+Na]^+$: 518.1760, found: 518.1759.
mp: 173.5 – 175.0 °C.

Neopentyl (*E*)-2-(1-methyl-1*H*-indol-5-yl)-2-phenylethene-1-sulfonate (5h)

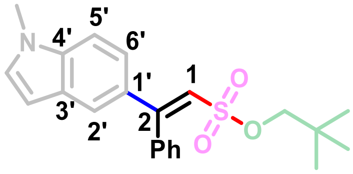

Following the general procedure C. neopentyl 2-phenylethyne-1-sulfonate (**3ah**) (25.2 mg, 0.1 mmol), (1-methyl-1*H*-indol-5-yl)boronic acid (52 mg, 0.3 mmol, 3 equiv.), (44 mg, 0.3 mmol, 3 equiv.), Cu(OAc)₂ (1.8 mg), and NMP (0.5 mL) were used. Purification via column chromatography on silica gel (cyclohexane: ethyl acetate = 95:5, v/v) afforded **5h** as a yellow solid; Yield 91% (35 mg).

NMR Spectroscopy (see spectra):

^{1}**H NMR** (400 MHz, $CDCl_3$): δ 7.52 (d, *J* = 1.8 Hz, 1H), 7.50 – 7.41 (m, 5H), 7.32 (d, *J* = 8.6 Hz, 1H), 7.21 (dd, *J* = 8.7, 1.8 Hz, 1H), 7.11 (d, *J* = 3.1 Hz, 1H), 6.78 (s, 1H), 6.50 (d, *J* = 3.0 Hz, 1H), 3.83 (s, 3H), 3.76 (s, 2H), 0.96 (s, 9H).
^{13}**C NMR** (101 MHz, $CDCl_3$): δ 158.07, 137.75, 136.95, 130.60, 130.32, 129.85, 129.16, 128.41, 127.89, 122.45, 121.92, 118.99, 109.41, 102.14, 78.88, 33.04, 31.64, 26.14.
E/*Z*-isomerism was determined via a *^{1}H,^{1}H-NOESY*-contact between 1-H and 2'-H, 6'-H which is only possible in the (*E*)-configured molecule.

HRMS (ESI+): m/z calc'd for **5h** $C_{22}H_{25}NO_3SNa$ $[M+Na]^+$: 406.1447, found: 406.1440.
mp: 134.6 – 136.6 °C.

Neopentyl (*E*)-2-(4-bromophenyl)-2-(1-methyl-1*H*-indol-5-yl)ethene-1-sulfonate (5i)

Following the general procedure C. neopentyl 2-(4-bromophenyl)ethyne-1-sulfonate (**3ca**) (33.1 mg, 0.1 mmol), (1-methyl-1*H*-indol-5-yl)boronic acid (52 mg, 0.3 mmol, 3 equiv.), $Cu(OAc)_2$ (1.8 mg), and NMP (0.5 mL) were used. Purification via column chromatography on silica gel (cyclohexane: ethyl acetate = 95:5, v/v) afforded **5i** as a colorless solid; Yield 80% (37 mg).

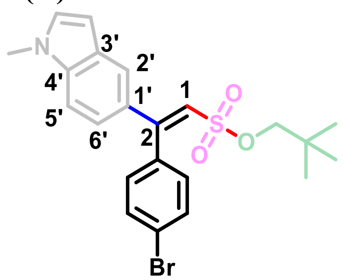

NMR Spectroscopy (see spectra):
^{1}**H NMR** (300 MHz, $CDCl_3$): δ 7.58 (d, *J* = 8.5 Hz, 2H), 7.49 (d, *J* = 1.2 Hz, 1H), 7.35 – 7.27 (m, 3H), 7.18 (dd, *J* = 8.7, 1.9 Hz, 1H), 7.13 (d, *J* = 3.2 Hz, 1H), 6.77 (s, 1H), 6.50 (dd, *J* = 3.2, 0.8 Hz, 1H), 3.84 (s, 3H), 3.79 (s, 2H), 0.97 (s, 9H).
^{13}**C NMR** (75 MHz, $CDCl_3$): δ 156.85, 137.81, 135.86, 131.50, 131.19, 130.48, 130.05, 128.45, 123.66, 122.47, 121.79, 119.28, 109.55, 102.19, 78.98, 33.08, 31.69, 26.13.
E/*Z*-isomerism was determined via a *^{1}H,^{1}H-NOESY*-contact between 1-H and 2'-H, 6'-H which is only possible in the (*E*)-configured molecule.

HRMS (ESI+): m/z calc'd for **5i** $C_{22}H_{24}BrNO_3SNa$ $[M+Na]^+$: 484.0552 (^{79}Br), found: 484.0553.
mp: 168.8 – 169.9 °C.

Ethyl (*E*)-2-(4-methoxyphenyl)-2-phenylethene-1-sulfonate (5j)

Following the general procedure C. ethyl 2-phenylethyne-1-sulfonate (**3ab**) (21 mg, 0.1 mmol), (4-methoxyphenyl)boronic acid (36.6 mg, 0.3 mmol, 3 equiv.), $Cu(OAc)_2$ (1.8 mg), and MeOH (0.5 mL) were used. Purification via column chromatography on silica gel (cyclohexane: ethyl acetate = 95:5, v/v) afforded **5j** as a colorless solid; Yield 64% (20 mg).

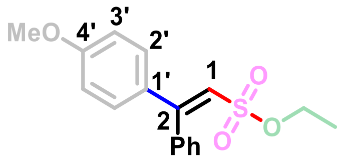

NMR Spectroscopy (see spectra):
^{1}**H NMR** (400 MHz, $CDCl_3$): δ 7.50 – 7.41 (m, 3H), 7.40 – 7.33 (m, 2H), 7.24 (d, *J* = 8.9 Hz, 2H), 6.89 (d, *J* = 8.9 Hz, 2H), 6.70 (s, 1H), 4.12 (q, *J* = 7.1 Hz, 2H), 3.85 (s, 3H), 1.27 (t, *J* = 7.1 Hz, 3H).
^{13}**C NMR** (101 MHz, $CDCl_3$): δ 161.58, 155.84, 136.15, 131.34, 130.08, 129.56, 129.30, 128.01, 119.43, 114.07, 66.23, 55.44, 14.82.
E/*Z*-isomerism was determined via a *^{1}H,^{1}H-NOESY*-contact between 1-H and 2'-H which is only possible in the (*E*)-configured molecule.

HRMS (ESI+): m/z calc'd for **5j** $C_{17}H_{18}O_4SNa$ $[M+Na]^+$: 341.0818, found: 341.0825.
mp: 95.3 – 97.7 °C.

Neopentyl (*E*)-2-phenyl-2-(pyridin-3-yl)ethene-1-sulfonate (5k)

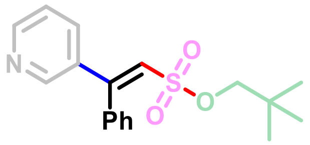

Following the general procedure B. neopentyl 2-phenylethyne-1-sulfonate (**3ah**) (25.2 mg, 0.1 mmol), 3-pyridinylboronic acid (37 mg, 0.3 mmol, 3 equiv.), $Cu(OAc)_2$ (1.8 mg), and MeOH (0.5 mL) were used. Purification via column chromatography on silica gel (cyclohexane: ethyl acetate = 95:5, v/v) afforded **5k** as a yellow solid; Yield 78 % (26 mg).

Following the general procedure C. neopentyl 2-phenylethyne-1-sulfonate (3ah) (25.2 mg, 0.1 mmol), 3-pyridinylboronic acid (37 mg, 0.3 mmol, 3 equiv.), $Cu(OAc)_2$ (1.8 mg), and NMP (0.5 mL) were used. Purification via column chromatography on silica gel (cyclohexane: ethyl acetate = 95:5, v/v) afforded **5k** as a yellow solid; Yield 81% (27 mg).

NMR Spectroscopy (see spectra):
^{1}H NMR (400 MHz, $CDCl_3$): δ 8.64 (d, *J* = 29.3 Hz, 2H), 7.54 (dt, *J* = 8.0, 2.0 Hz, 1H), 7.49 – 7.43 (m, 3H), 7.40 – 7.36 (m, 2H), 7.36 – 7.29 (m, 1H), 6.78 (s, 1H), 3.76 (s, 2H), 0.93 (s, 9H).
^{13}C NMR (101 MHz, $CDCl_3$): δ 153.12, 151.22, 148.92, 135.87, 135.02, 129.92, 129.61, 128.39, 123.45, 123.01, 79.46, 31.66, 26.03.

HRMS (ESI+): m/z calc'd for **5k** $C_{18}H_{21}NO_3S$ $[M+H]^+$: 332.1315, found: 332.1317.
mp: 105.6 – 107.7 °C.

Neopentyl (*E*)-2-(2-methoxypyrimidin-5-yl)-2-phenylethene-1-sulfonate (5l)

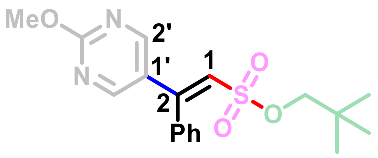

Following the general procedure B. neopentyl 2-phenylethyne-1-sulfonate (**3ah**) (25.2 mg, 0.1 mmol), 2-methoxypyrimidine-5-boronic acid (46 mg, 0.3 mmol, 3 equiv.), $Cu(OAc)_2$ (1.8 mg), and MeOH (0.5 mL) were used. Purification via column chromatography on silica gel (cyclohexane: ethyl acetate = 95:5, v/v) afforded **5l** as a colorless solid; Yield 77 % (28 mg).

Following the general procedure C. neopentyl 2-phenylethyne-1-sulfonate (3ah) (25.2 mg, 0.1 mmol), 2-methoxypyrimidine-5-boronic acid (46 mg, 0.3 mmol, 3 equiv.), $Cu(OAc)_2$ (1.8 mg), and NMP (0.5 mL) were used. Purification via column chromatography on silica gel (cyclohexane: ethyl acetate = 95:5, v/v) afforded **5l** as a colorless solid; Yield 83% (30 mg).

NMR Spectroscopy (see spectra):
^{1}H NMR (400 MHz, $CDCl_3$): δ 8.43 (s, 2H), 7.52 – 7.44 (m, 3H), 7.41 – 7.34 (m, 2H), 6.75 (s, 1H), 4.07 (s, 3H), 3.75 (s, 2H), 0.93 (s, 9H).
^{13}C NMR (101 MHz, $CDCl_3$): δ 166.41, 158.78, 150.02, 134.32, 130.18, 129.47, 128.59, 126.96, 121.53, 79.45, 55.53, 31.66, 26.02.
E/*Z*-isomerism was determined via a *^{1}H,^{1}H-NOESY*-contact between 1-H and 2'-H, which is only possible in the (*E*)-configured molecule.

HRMS (ESI+): m/z calc'd for **5l** $C_{18}H_{23}N_2O_4S$ $[M+H]^+$: 363.1373, found: 363.1364.
mp: 162.7 – 165.4 °C.

Neopentyl (*Z*)-2-(cyclopent-1-en-1-yl)-2-phenylethene-1-sulfonate (5m)

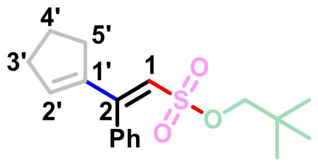

Following the general procedure B. neopentyl 2-phenylethyne-1-sulfonate (**3ah**) (25.2 mg, 0.1 mmol), cyclopenten-1-ylboronic acid (33 mg, 0.3 mmol, 3 equiv.), $Cu(OAc)_2$ (1.8 mg), and MeOH (0.5 mL) were used. Purification via column chromatography on silica gel (cyclohexane: ethyl acetate = 95:5, v/v) afforded **5m** as a yellow oil; Yield 73% (23 mg).

Following the general procedure C. neopentyl 2-phenylethyne-1-sulfonate (**3ah**) (25.2 mg, 0.1 mmol), cyclopenten-1-ylboronic acid (33 mg, 0.3 mmol, 3 equiv.), $Cu(OAc)_2$ (1.8 mg), and NMP (0.5 mL) were used. Purification via column chromatography on silica gel (cyclohexane: ethyl acetate = 95:5, v/v) afforded **5m** as a colorless solid; Yield 70% (22 mg).

NMR Spectroscopy (see spectra):
^{1}H NMR (400 MHz, $CDCl_3$): δ 7.45 – 7.35 (m, 3H), 7.29 – 7.24 (m, 2H), 6.26 (d, *J* = 1.0 Hz, 1H), 5.81 – 5.70 (m, 1H), 3.65 (s, 2H), 2.63 – 2.57 (m, 2H), 2.52 – 2.45 (m, 2H), 2.06 (p, *J* = 7.6 Hz, 2H), 0.92 (s, 9H).
^{13}C NMR (101 MHz, $CDCl_3$): δ 152.13, 143.70, 143.10, 135.54, 128.59, 128.41, 127.68, 120.42, 78.79, 33.93, 32.12, 31.60, 26.08, 23.22.
E/*Z*-isomerism was determined via a *^{1}H,^{1}H-NOESY*-contact between 1-H and 5'-H, which is only possible in the (*Z*)-configured molecule.

HRMS (ESI+): m/z calc'd for **5m** $C_{18}H_{24}O_3SNa$ $[M+Na]^+$: 343.1338, found: 343.1333.

Neopentyl (1*Z*,3*E*)-2,4-diphenylbuta-1,3-diene-1-sulfonate (5n)

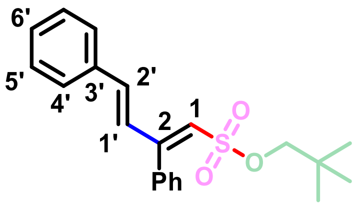

Following the general procedure B. neopentyl 2-phenylethyne-1-sulfonate (**3ah**) (25.2 mg, 0.1 mmol), trans-2-phenylvinylboronic acid (44.5 mg, 0.3 mmol, 3 equiv.), $Cu(OAc)_2$ (1.8 mg), and MeOH (0.5 mL) or NMP (0.5 mL) were used. Purification via column chromatography on silica gel (cyclohexane: ethyl acetate = 95:5, v/v) afforded **5n** as a colorless oil; Yield 36 % (13 mg).

Following the general procedure C. neopentyl 2-phenylethyne-1-sulfonate (**3ah**) (25.2 mg, 0.1 mmol), trans-2-phenylvinylboronic acid (44.5 mg, 0.3 mmol, 3 equiv.), $Cu(OAc)_2$ (1.8 mg), and NMP (0.5 mL) were used. Purification via column chromatography on silica gel (cyclohexane: ethyl acetate = 95:5, v/v) afforded **5n** as a colorless oil; Yield 70% (25 mg).

NMR Spectroscopy (see spectra):
^{1}H NMR (400 MHz, $CDCl_3$): δ 7.52 – 7.46 (m, 3H), 7.43 – 7.33 (m, 7H), 7.01 (d, *J* = 15.9 Hz, 1H), 6.53 (s, 1H), 6.48 (d, *J* = 15.8 Hz, 1H), 3.71 (s, 2H), 0.95 (s, 9H).
^{13}C NMR (101 MHz, $CDCl_3$): δ 154.25, 140.79, 135.45, 133.67, 129.49, 129.15, 128.92, 128.90, 128.87, 128.12, 127.44, 123.45, 78.98, 31.64, 26.11.
E/*Z*-isomerism was determined via a *^{1}H,^{1}H-NOESY*-contact between 1-H and 2'-H, which is only possible in the (1*Z*)-configured molecule.

HRMS (ESI+): m/z calc'd for **5n** $C_{21}H_{24}O_3SNa$ $[M+Na]^+$: 379.1338, found: 379.1341.

Neopentyl (*Z*)-2-phenylpenta-1,4-diene-1-sulfonate (5o)

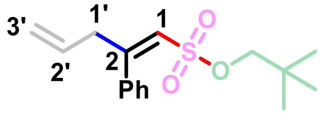

Following the general procedure B. neopentyl 2-phenylethyne-1-sulfonate (**3ah**) (25.2 mg, 0.1 mmol), allylboronic acid pinacol ester (50 mg, 0.3 mmol, 3 equiv.), $Cu(OAc)_2$ (1.8 mg), and MeOH (0.5 mL) were used. Purification via column chromatography on silica gel (cyclohexane: ethyl acetate = 95:5, v/v) afforded **5o** as a colorless oil; Yield 85% (25 mg).

Following the general procedure C. neopentyl 2-phenylethyne-1-sulfonate (**3ah**) (25.2 mg, 0.1 mmol), Allylboronic acid pinacol ester (50 mg, 0.3 mmol, 3 equiv.), $Cu(OAc)_2$ (1.8 mg), and NMP (0.5 mL) were used. Purification via column chromatography on silica gel (cyclohexane: ethyl acetate = 95:5, v/v) afforded **5o** as a colorless solid; Yield 85% (25 mg).

NMR Spectroscopy (see spectra):

^{1}H NMR (400 MHz, $CDCl_3$): δ 7.44 – 7.38 (m, 3H), 7.37 – 7.33 (m, 2H), 6.34 (t, J = 1.5 Hz, 1H), 5.79 (ddt, J = 17.0, 10.1, 6.9 Hz, 1H), 5.25 – 5.16 (m, 2H), 3.66 (s, 2H), 3.26 (dq, J = 6.9, 1.4 Hz, 2H), 0.90 (s, 9H).
^{13}C NMR (101 MHz, $CDCl_3$): δ 157.20, 136.94, 132.34, 128.96, 128.16, 127.43, 122.46, 119.67, 79.00, 44.21, 31.58, 26.03.
E/*Z*-isomerism was determined via a *^{1}H,^{1}H-NOESY*-contact between 1-H and 1'-H, which is only possible in the (*Z*)-configured molecule.

HRMS (ESI+): m/z calc'd for **5o** $C_{16}H_{22}O_3SNa$ $[M+Na]^+$: 317.1182, found: 317.1177.

Neopentyl (*Z*)-2-phenylpenta-1,3,4-triene-1-sulfonate (5p)

Following the general procedure B. neopentyl 2-phenylethyne-1-sulfonate (**3ah**) (25.2 mg, 0.1 mmol), allenylboronic acid pinacol ester (50 mg, 0.3 mmol, 3 equiv.), $Cu(OAc)_2$ (1.8 mg), and MeOH (0.5 mL) were used. Purification via column chromatography on silica gel (cyclohexane: ethyl acetate = 95:5, v/v) afforded **5p** as a colorless oil; Yield 75% (22 mg).

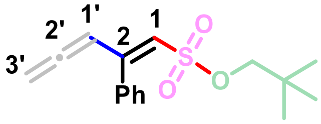

NMR Spectroscopy (see spectra):
^{1}H NMR (400 MHz, $CDCl_3$): δ 7.39 (q, J = 3.6 Hz, 3H), 7.35 – 7.30 (m, 2H), 6.38 (s, 1H), 6.08 (t, J = 6.5 Hz, 1H), 4.97 (dd, J = 6.5, 1.5 Hz, 2H), 3.66 (s, 2H), 0.91 (s, 9H).
^{13}C NMR (101 MHz, $CDCl_3$): δ 214.18, 151.71, 134.16, 129.04, 128.46, 127.72, 121.24, 96.85, 79.63, 79.03, 31.58, 26.04.
E/*Z*-isomerism was determined via a *^{1}H,^{1}H-NOESY*-contact between 1-H and 1'-H, which is only possible in the (*Z*)-configured molecule.

HRMS (ESI+): m/z calc'd for **5p** $C_{16}H_{20}O_3SNa$ $[M+Na]^+$: 315.1025, found: 315.1024.

Neopentyl (*E*)-2-ferrocenyl-2-phenylethene-1-sulfonate (5q)

Following the general procedure B. neopentyl 2-phenylethyne-1-sulfonate (3ah) (25.2 mg, 0.1 mmol), ferroceneboronic acid (72 mg, 0.3 mmol, 3 equiv.), $Cu(OAc)_2$ (1.8 mg), and MeOH (0.5 mL) were used. Purification via column chromatography on silica gel (cyclohexane: ethyl acetate = 95:5, v/v) afforded **5q** as a red oil; Yield 17% (8 mg).

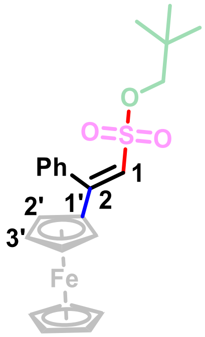

NMR Spectroscopy (see spectra):
^{1}H NMR (400 MHz, $CDCl_3$): δ 7.48 – 7.43 (m, 3H), 7.43 – 7.36 (m, 2H), 6.62 (s, 1H), 4.46 (t, J = 1.9 Hz, 2H), 4.31 (t, J = 1.9 Hz, 2H), 4.21 (s, 4H), 3.65 (s, 2H), 0.93 (s, 9H).
^{13}C NMR (101 MHz, $CDCl_3$): δ 158.46, 135.54, 128.65, 128.35, 127.71, 116.08, 81.83, 78.63, 71.31, 70.17, 68.72, 31.57, 26.12, 26.08.
E/*Z*-isomerism was determined via a *^{1}H,^{1}H-NOESY*-contact between 1-H and 2'-H, which is only possible in the (*E*)-configured molecule.

HRMS (ESI+): m/z calc'd for **5q** $C_{23}H_{26}FeO_3SNa$ $[M+Na]^+$: 461.0837, found: 459.0891.

Neopentyl (*E*)-2-(1-methyl-1*H*-indol-5-yl)dec-1-ene-1-sulfonate (5r)

Following the general procedure C. neopentyl dec-1-yne-1-sulfonate (**3la**) (28.8 mg, 0.1 mmol), (1-methyl-1*H*-indol-5-yl)boronic acid (52 mg, 0.3 mmol, 3 equiv.), $Cu(OAc)_2$ (1.8 mg), and NMP (0.5 mL) were used. Purification via column chromatography on silica gel (cyclohexane: ethyl acetate = 95:5, v/v) afforded **5r** as a yellow oil; Yield 36% (15 mg).

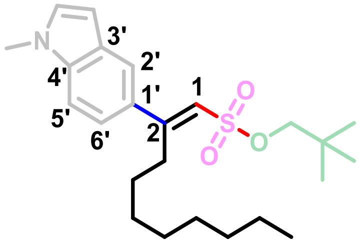

NMR Spectroscopy (see spectra):

^{1}**H NMR** (300 MHz, $CDCl_3$): δ 7.73 (dd, J = 1.8, 0.7 Hz, 1H), 7.38 – 7.28 (m, 2H), 7.13 (d, J = 3.1 Hz, 1H), 6.56 (dd, J = 3.2, 0.9 Hz, 1H), 6.41 (s, 1H), 3.86 (s, 2H), 3.84 (s, 3H), 3.19 – 3.01 (m, 2H), 1.50 – 1.35 (m, 4H), 1.23 (d, J = 2.5 Hz, 8H), 1.02 (s, 9H), 0.89 – 0.84 (m, 4H).
^{13}**C NMR** (75 MHz, $CDCl_3$): δ 162.00, 137.45, 130.18, 129.95, 128.53, 120.42, 119.71, 119.30, 109.58, 101.83, 78.89, 77.24, 33.03, 31.81, 31.32, 30.96, 29.71, 29.30, 29.17, 28.94, 26.21, 22.64, 14.10.
E/*Z*-isomerism was determined via a *^{1}H,^{1}H-NOESY*-contact between 1-H and 2'-H, 6'-H which is only possible in the (*E*)-configured molecule.

HRMS (ESI+): m/z calc'd for **5r** $C_{24}H_{37}NO_3SNa$ $[M+Na]^+$: 442.2386, found: 442.2382.

Neopentyl (*Z*)-2-phenylpenta-1,4-diene-1-sulfonate (5s)

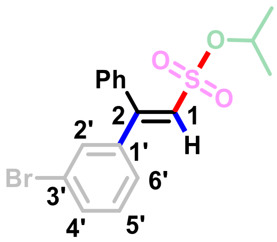

Following the general procedure B. isopropyl 2-phenylethyne-1-sulfonate (**3at**) (22.5 mg, 0.1 mmol), (3-bromophenyl)boronic acid (60 mg, 0.3 mmol, 3 equiv.), $Cu(OAc)_2$ (1.8 mg), and MeOH (0.5 mL) were used. Purification via column chromatography on silica gel (cyclohexane: ethyl acetate = 95:5, v/v) afforded **5s** as a colorless oil; Yield 30% (11 mg).

Following the general procedure C. isopropyl 2-phenylethyne-1-sulfonate (3at) (22.5 mg, 0.1 mmol), (3-bromophenyl)boronic acid (60 mg, 0.3 mmol, 3 equiv.), $Cu(OAc)_2$ (1.8 mg), and NMP (0.5 mL) were used. Purification via column chromatography on silica gel (cyclohexane: ethyl acetate = 95:5, v/v) afforded **5s** as a colorless solid; Yield 43% (16 mg).

NMR Spectroscopy (see spectra):
^{1}**H NMR** (400 MHz, $CDCl_3$): δ 7.57 (ddd, J = 7.8, 1.9, 1.2 Hz, 1H), 7.51 – 7.41 (m, 4H), 7.36 (dd, J = 7.8, 1.8 Hz, 2H), 7.26 (t, J = 7.8 Hz, 1H), 7.20 (dt, J = 7.9, 1.4 Hz, 1H), 6.74 (s, 1H), 4.84 (hept, J = 6.3 Hz, 1H), 1.35 (d, J = 6.3 Hz, 6H).
^{13}**C NMR** (101 MHz, $CDCl_3$): δ 154.04, 141.59, 135.42, 133.19, 131.28, 130.17, 129.68, 129.64, 128.20, 127.17, 124.16, 122.87, 77.16, 23.03.
E/*Z*-isomerism was determined via a *^{1}H,^{1}H-NOESY*-contact between 1-H and 6'-H, which is only possible in the (*Z*)-configured molecule.

HRMS (ESI+): m/z calc'd for **5s** $C_{17}H_{17}BrNO_3S$ $[M+NH_4]^+$: 398.0420 (^{79}Br), found: 398.0411.

4-((2,2-Diphenylvinyl)sulfonyl)morpholine (5t)

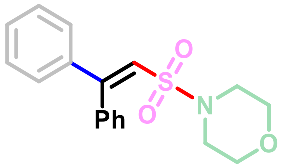

Following the general procedure B. 4-((phenylethynyl)sulfonyl)morpholine (**3azf**) (25.2 mg, 0.1 mmol), phenylboronic acid (36.6 mg, 0.3 mmol, 3 equiv.), $Cu(OAc)_2$ (1.8 mg), and MeOH (0.5 mL) were used. Purification via column chromatography on silica gel (cyclohexane: ethyl acetate = 95:5, v/v) afforded **5t** as a colorless solid; Yield 68% (22 mg).

NMR Spectroscopy (see spectra):
^{1}**H NMR** (400 MHz, $CDCl_3$): 1H NMR (400 MHz, CDCl3) δ 7.48 – 7.33 (m, 8H), 7.31 – 7.25 (m, 2H), 6.66 (s, 1H), 3.69 – 3.60 (m, 4H), 3.16 – 3.07 (m, 4H).
^{13}**C NMR** (101 MHz, $CDCl_3$): δ 155.58, 139.79, 136.45, 130.24, 129.84, 129.14, 128.69, 128.38, 127.98, 121.82, 66.39, 45.55.

HRMS (ESI+): m/z calc'd for **5t** $C_{18}H_{19}NO_3SNa$ $[M+Na]^+$: 352.0978, found: 352.0978.
mp: 117.5 – 119.2

Neopentyl 5-methyl-7-phenyl-2,3-dihydro-1*H*-pyrrolizine-6-sulfonate (6a)

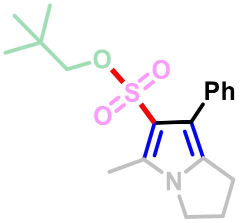

A 5 mL crimp reaction vial was charged with L-proline (11.5 mg, 0.1 mmol), Ac_2O (400 µL), neopentyl 2-phenylethyne-1-sulfonate (**3ah**) (30 mg, 0.12 mmol, and a magnetic stir bar. Then heated to 120 °C for 2 h. The Ac_2O was removed under a stream of nitrogen. Purification via column chromatography on silica gel (cyclohexane: ethyl acetate = 80:20, v/v) afforded **6a** as a pale brown solid; Yield 72% (25 mg).

NMR Spectroscopy (see spectra):
^{1}H NMR (400 MHz, $CDCl_3$): δ 7.52 (d, *J* = 6.9 Hz, 2H), 7.38 – 7.33 (m, 2H), 7.28 – 7.21 (m, 1H), 3.96 (t, *J* = 7.2 Hz, 2H), 3.53 (s, 2H), 2.94 – 2.88 (m, 2H), 2.58 – 2.48 (m, 5H), 0.78 (s, 9H).
^{13}C NMR (101 MHz, $CDCl_3$): δ 134.12, 134.03, 130.38, 129.95, 129.44, 127.91, 126.43, 115.23, 114.20, 78.22, 45.20, 31.31, 26.74, 26.02, 24.29.

HRMS (ESI+): m/z calc'd for **6a** $C_{19}H_{25}NO_3SNa$ $[M+Na]^+$: 370.1447, found: 370.1438.
mp: 110.7 – 111.9 °C.

Neopentyl 3a-hydroxy-8-oxo-2-phenyl-3a,8-dihydroisoxazolo[3,2-a]isoindole-3-sulfonate (6b)

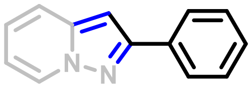

A 10 mL reaction flask was charged with neopentyl 2-phenylethyne-1-sulfonate (**3ah**) (25.2 mg, 0.1 mmol), MeCN (1 mL), potassium carbonate (27.6 mg, 0.2 mmol, 2 equiv.), 1-aminopyridinium iodide (25 mg, 0.11 mmol, 1.1 equiv.) and a magnetic stir bar. The resulting mixture was stirred at room temperature For 12 h. Then the reaction mixture was diluted with ethyl acetate, which was washed with water and brine, dried over $MgSO_4$, filtered, and concentrated in vacuo. Purification via column chromatography on silica gel (cyclohexane: ethyl acetate = 90:10, v/v) afforded **6b** as a colorless solid; Yield 77 % (15 mg).

NMR Spectroscopy (see spectra):
^{1}H NMR (300 MHz, $CDCl_3$): δ 8.50 (dd, *J* = 7.0, 1.0 Hz, 1H), 8.03 – 7.97 (m, 2H), 7.55 – 7.45 (m, 3H), 7.43 – 7.36 (m, 1H), 7.14 – 7.08 (m, 1H), 6.82 (s, 1H), 6.75 (td, *J* = 6.9, 1.4 Hz, 1H).
^{13}C NMR (101 MHz, $CDCl_3$): δ 153.55, 141.65, 133.22, 128.76, 128.53, 128.44, 126.51, 123.46, 117.94, 111.74, 93.73.

HRMS (ESI+): m/z calc'd for **6b** $C_{13}H_{11}N_2$ $[M+H]^+$: 195.0917, found: 195.0909.
mp: 110.2-111.5 °C.

Neopentyl 3a-hydroxy-8-oxo-2-phenyl-3a,8-dihydroisoxazolo[3,2-a]isoindole-3-sulfonate (6c)

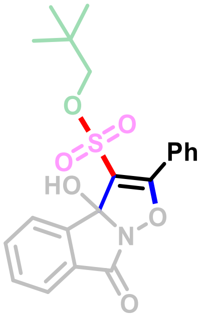

A 10 mL Schlenk tube was charged with *N*-hydroxyphthalimide (16.3 mg, 0.1 mmol), neopentyl 2-phenylethyne-1-sulfonate (**3ah**) (30 mg, 0.12 mmol), Ph_3P (5.3 mg, 0.02 mmol), DMF (0.5 mL), and a magnetic stir bar. The resulting mixture was stirred at room temperature under nitrogen atmosphere For 2 h. After completion of the reaction as monitored by TLC, the reaction mixture was diluted with CH_2Cl_2, which was washed with water and brine, dried over $MgSO_4$, filtered, and concentrated in vacuo. Purification via column chromatography on silica gel (cyclohexane: ethyl acetate = 70:30, v/v) afforded **6c** as a colorless solid; Yield 84% (35 mg).

NMR Spectroscopy (see spectra):
^{1}H NMR (300 MHz, $CDCl_3$): δ 8.08 (d, *J* = 7.8 Hz, 1H), 7.96 – 7.83 (m, 3H), 7.80 (td, *J* = 7.6, 1.2 Hz, 1H), 7.65 – 7.54 (m, 2H), 7.53 – 7.40 (m, 2H), 4.41 (s, 1H), 3.54 (d, *J* = 8.8 Hz, 1H), 3.40 (d, *J* = 8.8 Hz, 1H), 0.66 (s, 9H).
^{13}C NMR (75 MHz, $CDCl_3$): δ 173.19, 168.25, 145.10, 135.91, 132.82, 131.09, 129.96, 128.45, 127.93, 126.41, 125.06, 124.49, 110.01, 98.05, 80.12, 31.26, 25.73.

HRMS (ESI+): m/z calc'd for **6c** $C_{21}H_{21}NO_6SNa$ $[M+Na]^+$: 438.0982, found: 438.0972.

mp: 126.7 – 128.3 °C.

Neopentyl 2-oxo-2-phenylethane-1-sulfonate (6d)

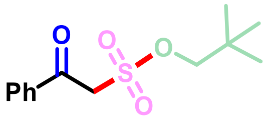

A 5 mL crimp reaction vial was charged with neopentyl 2-phenylethyne-1-sulfonate (**3ah**) (25.2 mg, 0.1 mmol), acetone (0.5mL), H_2O (0.5 mL), and a magnetic stir bar. The resulting mixture was stirred at room temperature For 48 h. After completion of the reaction as monitored by TLC, the reaction mixture was diluted with ethyl acetate, which was washed with water and brine, dried over $MgSO_4$, filtered, and concentrated in vacuo. Purification via column chromatography on silica gel (cyclohexane: ethyl acetate = 85:15, v/v) afforded **6d** as a brown oil; Yield 87 % (23 mg).

NMR Spectroscopy (see spectra):
^{1}H NMR (300 MHz, $CDCl_3$): δ 8.09 – 7.97 (m, 2H), 7.71 – 7.63 (m, 1H), 7.58 – 7.51 (m, 2H), 4.74 (s, 2H), 4.02 (s, 2H), 0.98 (s, 9H).
^{13}C NMR (75 MHz, $CDCl_3$): δ 187.28, 135.34, 134.60, 129.29, 129.01, 80.93, 57.14, 31.90, 25.97.

HRMS (ESI+): m/z calc'd for **6d** $C_{13}H_{18}O_4SNa$ $[M+Na]^+$: 293.0818, found: 293.0813.
mp: 57.7 – 58.9 °C.

Dineopentyl-3,4-diphenyl-2,5-dihydrothiophene-2,5-disulfonate (6e)

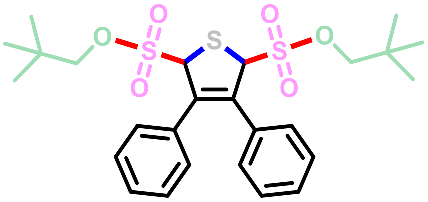

A 5 mL crimp reaction vial was charged with neopentyl 2-phenylethyne-1-sulfonate (**3ah**) (25.2 mg, 0.1 mmol), sulfur (S_8, 9.6 mg, 0.033 mmol), KOH (5.6 mg, 0.1 mmol), toluene (0.5 mL), and a magnetic stir bar. The resulting mixture was stirred at 120 °C For 3 h. After completion of the reaction as monitored by TLC, the reaction mixture was diluted with ethyl acetate, which was washed with water and brine, dried over $MgSO_4$, filtered, and concentrated in vacuo. Purification via column chromatography on silica gel (cyclohexane: ethyl acetate = 80:20, v/v) afforded **6e** as a yellow oil; Yield 70 % (19 mg).

NMR Spectroscopy (see spectra):
^{1}H NMR (300 MHz, $CDCl_3$): δ 7.32 – 7.25 (m, 3H), 7.14 (t, J = 7.8 Hz, 4H), 7.03 – 6.97 (m, 4H), 6.44 (s, 2H), 3.95 (s, 4H), 1.07 (s, 18H).
^{13}C NMR (75 MHz, $CDCl_3$): δ 153.48, 137.36, 130.32, 128.55, 127.96, 124.51, 79.94, 31.85, 26.21.

HRMS (ESI+): m/z calc'd for **6e** $C_{26}H_{34}O_6S_3Na$ $[M+Na]^+$: 561.1410, found: 561.1403.

Diphenyl(phenylethynyl)phosphine oxide (6f)

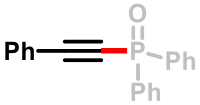

A 10 mL reaction flask was charged with neopentyl 2-phenylethyne-1-sulfonate (**3ah**) (25.2 mg, 0.1 mmol), MeCN (1 mL), cesium carbonate (65 mg, 0.2 mmol, 2 equiv.), diphenylphosphine oxide (40 mg, 0.2 mmol, 2 equiv.) and a magnetic stir bar. The resulting mixture was stirred at room temperature For 24 h under air. Then the reaction mixture was diluted with ethyl acetate, which was washed with water and brine, dried over $MgSO_4$, filtered, and concentrated in vacuo. Purification via column chromatography on silica gel (cyclohexane: ethyl acetate = 50:50, v/v) afforded **6f** as a colorless solid; Yield 50 % (15 mg).

NMR Spectroscopy (see spectra):
^{1}H NMR (300 MHz, $CDCl_3$): δ 8.00 – 7.85 (m, 4H), 7.65 – 7.45 (m, 10H), 7.44 – 7.37 (m, 2H).
^{13}C NMR (75 MHz, $CDCl_3$): δ 133.9, 132.6 (d, $^4J_{C-P}$ = 2.7 Hz), 132.3 (d, $^4J_{C-P}$ = 3.8 Hz), 131.0 (d, $^3J_{C-P}$ = 15.2 Hz), 130.7, 128.8, 128.6, 120.0 (d, $^3J_{C-P}$ = 5.4.0 Hz), 105.5 (d, $^2J_{C-P}$ = 40.3 Hz), 82.8 (d, $^1J_{C-P}$ = 227.0.7 Hz).

HRMS (ESI+): m/z calc'd for **6f** $C_{20}H_{15}OPNa$ $[M+Na]^+$: 325.0753, found: 325.0744.
mp:101.4-103.1 °C.

Neopentyl (*E*)-2-morpholino-2-phenylethene-1-sulfonate (6g)

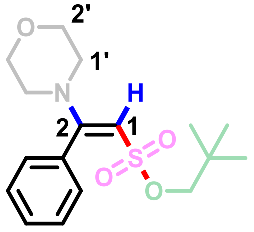

Following the general procedure D without Et_3N. with neopentyl 2-phenylethyne-1-sulfonate (**3ah**) (25.2 mg, 0.1 mmol), MeCN (1 mL) and morpholin (9 µL, 9 mg, 1 mmol, 1 equiv.) were used. Purification via column chromatography on silica gel (cyclohexane: ethyl acetate = 75:25, v/v) afforded **6g** as a yellow oil; Yield 79% (27 mg).

NMR Spectroscopy (see spectra):
^{1}**H NMR** (400 MHz, $CDCl_3$): δ 7.46 – 7.40 (m, 3H), 7.39 – 7.32 (m, 2H), 5.31 (s, 1H), 3.73 – 3.68 (m, 4H), 3.59 (s, 2H), 3.12 – 3.03 (m, 4H), 0.93 (s, 9H).
^{13}**C NMR** (101 MHz, $CDCl_3$): δ 162.04, 132.85, 129.96, 129.16, 128.35, 94.28, 77.56, 66.27, 47.94, 31.50, 26.25.
E/*Z*-isomerism was determined via a *^{1}H,^{1}H-NOESY*-contact between 1-H and 1'-H, which is only possible in the (*E*)-configured molecule.

HRMS (ESI+): m/z calc'd for **6g** $C_{17}H_{25}NO_4S$ $[M+Na]^+$: 362.1397, found: 362.1385.

Neopentyl (E)-2-(cis-2,6-dimethylmorpholino)-2-phenylethene-1-sulfonate (6h)

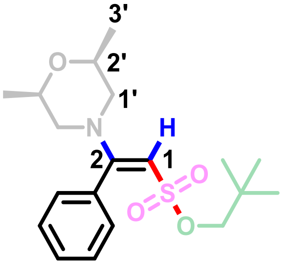

Following the general procedure D without Et_3N. with neopentyl 2-phenylethyne-1-sulfonate (3ah) (25.2 mg, 0.1 mmol), MeCN (1 mL) and cis-2,6-dimethylmorpholin (12 µL, 11.5 mg, 0.1 mmol, 1 equiv.) were used. Purification via column chromatography on silica gel (cyclohexane: ethyl acetate = 75:25, v/v) afforded **6h** as a yellow oil; Yield 62% (21 mg).

NMR Spectroscopy (see spectra):
^{1}**H NMR** (300 MHz, $CDCl_3$): δ 7.48 – 7.40 (m, 3H), 7.39 – 7.30 (m, 2H), 5.29 (s, 1H), 3.72 – 3.57 (m, 4H), 3.19 (d, J = 12.7 Hz, 2H), 2.51 (dd, J = 12.8, 10.6 Hz, 2H), 1.16 (d, J = 6.2 Hz, 6H), 0.93 (s, 9H).
^{13}**C NMR** (75 MHz, $CDCl_3$): δ 161.63, 132.98, 129.96, 129.15, 128.35, 93.86, 77.48, 71.28, 53.12, 31.51, 26.26, 18.72.
E/*Z*-isomerism was determined via a *^{1}H,^{1}H-NOESY*-contact between 1-H and 1'-H, which is only possible in the (*E*)-configured molecule.

HRMS (ESI+): m/z calc'd for **6h** $C_{19}H_{29}NO_4SNa$ $[M+Na]^+$: 390.1710, found: 390.1708.

Neopentyl (*Z*)-2-(1*H*-benzo[d][1,2,3]triazol-1-yl)-2-phenylethene-1-sulfonate (6i)

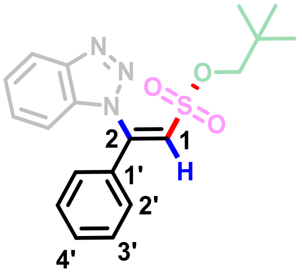

Following the general procedure D. with neopentyl 2-phenylethyne-1-sulfonate (**3ah**) (25.2 mg, 0.1 mmol), MeCN (1 mL), benzotriazole (12 mg, 0.1 mmol, 1 equiv.), Et_3N (20 µL, 23 mg, 1.4 mmol, 1.4 equiv.) were used. Purification via column chromatography on silica gel (cyclohexane: ethyl acetate = 80:20, v/v) afforded **6i** as a pale yellow solid; Yield 67% (25 mg).

NMR Spectroscopy (see spectra):
^{1}**H NMR** (300 MHz, $CDCl_3$): δ 8.21 – 8.10 (m, 1H), 7.61 – 7.54 (m, 1H), 7.49 – 7.39 (m, 4H), 7.30 – 7.23 (m, 2H), 7.11 – 7.04 (m, 1H), 7.00 (s, 1H), 4.01 (s, 2H), 1.00 (s, 9H).
^{13}**C NMR** (75 MHz, $CDCl_3$): δ 145.97, 144.80, 133.67, 132.57, 132.49, 129.54, 128.74, 127.75, 124.73, 120.43, 120.11, 110.62, 80.86, 31.83, 26.08.
E/*Z*-isomerism was determined via a *^{1}H,^{1}H-NOESY*-contact between 1-H and 2'-H, which is only possible in the (*Z*)-configured molecule.

HRMS (ESI+): m/z calc'd for **6i** $C_{19}H_{21}N_3O_3SNa$ $[M+Na]^+$: 394.1196, found: 394.1188.
mp: 128.7 – 130.4 °C.

Neopentyl (*Z*)-2-(1*H*-benzo[d]imidazol-1-yl)-2-phenylethene-1-sulfonate (6j)

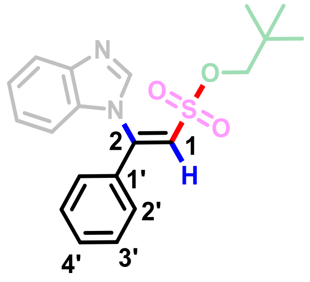

Following the general procedure D. with neopentyl 2-phenylethyne-1-sulfonate (**3ah**) (25.2 mg, 0.1 mmol), MeCN (1 mL), benzimidazole (12 mg, 0.1 mmol, 1 equiv.), Et_3N (20 µL, 23 mg, 1.4 mmol, 1.4 equiv.) were used. Purification via column chromatography on silica gel (cyclohexane: ethyl acetate = 80:20, v/v) afforded **6j** as a colorless solid; Yield 89% (33 mg).

NMR Spectroscopy (see spectra):
^{1}H NMR (300 MHz, $CDCl_3$): δ 8.37 (s, 1H), 7.86 (d, *J* = 8.2 Hz, 1H), 7.60 – 7.54 (m, 1H), 7.50 – 7.39 (m, 2H), 7.33 – 7.26 (m, 3H), 7.17 – 7.10 (m, 1H), 6.83 (s, 1H), 6.72 (dt, *J* = 8.1, 0.9 Hz, 1H), 3.73 (s, 2H), 0.81 (s, 9H).
^{13}C NMR (75 MHz, $CDCl_3$): δ 145.55, 144.05, 143.77, 133.59, 132.89, 132.53, 129.51, 127.85, 124.08, 123.46, 120.84, 117.92, 111.84, 80.17, 31.58, 25.81.
E/*Z*-isomerism was determined via a *^{1}H,^{1}H-NOESY*-contact between 1-H and 2'-H, which is only possible in the (*Z*)-configured molecule.

HRMS (ESI+): m/z calc'd for **6j** $C_{20}H_{22}N_2O_3SNa$ $[M+Na]^+$: 393.1243, found: 393.1235.
mp: 141.9 – 143.4 °C.

(*Z*)-2-((2-((Neopentyloxy)sulfonyl)-1-phenylvinyl)thio)pyridine 1-oxide (6k)

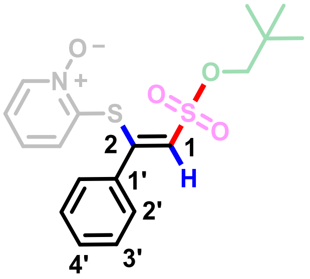

Following the general procedure D. with neopentyl 2-phenylethyne-1-sulfonate (**3ah**) (25.2 mg, 0.1 mmol), MeCN (1 mL), omadine (13 mg, 0.1 mmol, 1 equiv.), Et_3N (20 µL, 23 mg, 1.4 mmol, 1.4 equiv.) were used. After 20 min, the reaction mixture was concentrated under reduced pressure. Then, the crude mixture was diluted with EtOAc and washed with H_2O (2 x 20 mL), brine (2 x 20 mL), dried over anhydrous Na_2SO_4 and concentrated under reduced pressure; Yield 79% (30 mg).

NMR Spectroscopy (see spectra):
^{1}H NMR (400 MHz, $CDCl_3$): δ 8.03 (s, 1H), 7.56 (d, *J* = 7.3 Hz, 2H), 7.39 – 7.26 (m, 3H), 6.88 (s, 4H), 4.07 (s, 2H), 1.03 (s, 9H).
^{1}H NMR (600 MHz, pyridine-*d5*): δ 8.31 (d, *J* = 6.3 Hz, 1H), 7.75 (d, *J* = 7.5 Hz, 2H), 7.62 (s, 1H), 7.35 – 7.28 (m, 4H), 6.95 (td, *J* = 8.0, 2.0 Hz, 1H), 6.90 (t, *J* = 7.8 Hz, 1H), 4.37 (s, 2H), 1.00 (s, 9H).
^{13}C NMR (101 MHz, pyridine-*d5*): δ 152.73, 146.73, 139.79, 137.20, 131.62, 129.47, 129.29, 125.34, 125.04, 81.04, 32.38, 26.56.
E/*Z*-isomerism was determined via a *^{1}H,^{1}H-NOESY*-contact between 1-H and 2'-H, which is only possible in the (*Z*)-configured molecule.

HRMS (ESI+): m/z calc'd for **6k** $C_{18}H_{21}NO_4S_2Na$ $[M+Na]^+$: 402.0804, found: 402.0797.
mp: 137.0-139.1 °C.

Neopentyl (*Z*)-2-(benzo[d]thiazol-2-ylthio)-2-phenylethene-1-sulfonate (6l)

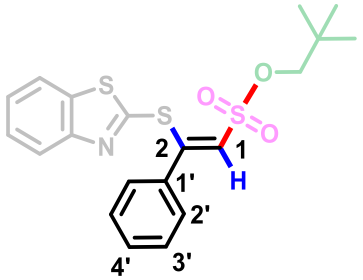

Following the general procedure D. with neopentyl 2-phenylethyne-1-sulfonate (**3ah**) (25.2 mg, 0.1 mmol), MeCN (1 mL), 2-mercaptobenzothiazole (17 mg, 0.1 mmol, 1 equiv.), Et_3N (20 µL, 23 mg, 1.4 mmol, 1.4 equiv.) were used. After 20 min, the reaction mixture was concentrated under reduced pressure. Then, the crude mixture was diluted with EtOAc and washed with H_2O (2 x 20 mL), brine

(2 x 20 mL), dried over anhydrous Na_2SO_4 and concentrated under reduced pressure. **6l** as a colorless solid was obtained; Yield 98% (41 mg).

NMR Spectroscopy (see spectra):
^{1}H NMR (300 MHz, $CDCl_3$): δ 7.89 (d, J = 8.1 Hz, 1H), 7.67 (d, J = 7.8 Hz, 1H), 7.62 – 7.51 (m, 2H), 7.42 (td, J = 7.8, 1.3 Hz, 1H), 7.35 – 7.24 (m, 4H), 6.76 (s, 1H), 4.04 (s, 2H), 1.05 (s, 9H).
^{13}C NMR (75 MHz, $CDCl_3$): δ 159.98, 152.65, 151.72, 137.10, 136.08, 130.87, 128.82, 128.68, 126.40, 125.79, 125.49, 122.98, 121.04, 80.31, 31.89, 26.18.
E/*Z*-isomerism was determined via a *^{1}H,^{1}H-NOESY*-contact between 1-H and 2'-H, which is only possible in the (*Z*)-configured molecule.

HRMS (ESI+): m/z calc'd for **6l** $C_{20}H_{21}NO_3S_3Na$ $[M+Na]^+$: 442.0576, found: 442.0569.
mp: 138.2 – 139.4 °C.

Neopentyl (*Z*)-2-(benzo[d]oxazol-2-ylthio)-2-phenylethene-1-sulfonate (6m)

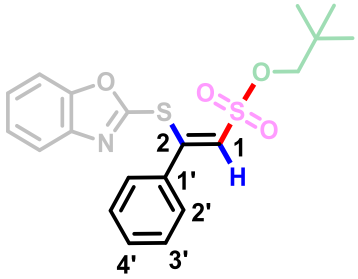

Following the general procedure D. with neopentyl 2-phenylethyne-1-sulfonate (**3ah**) (25.2 mg, 0.1 mmol), MeCN (1 mL), 2-mercaptobenzoxazole (15 mg, 0.1 mmol, 1 equiv.), Et_3N (20 µL, 23 mg, 1.4 mmol, 1.4 equiv.) were used. After 20 min, the reaction mixture was concentrated under reduced pressure. Then, the crude mixture was diluted with EtOAc and washed with H_2O (2 x 20 mL), brine (2 x 20 mL), dried over anhydrous Na_2SO_4 and concentrated under reduced pressure. **6m** as a colorless solid was obtained; Yield 99 % (40 mg).

2 mmol-scale : neopentyl 2-phenylethyne-1-sulfonate (**3ah**) (0.502 mg, 2.0 mmol), MeCN (15 mL), 2-mercaptobenzoxazole (302 mg, 2 mmol, 1 equiv.), Et_3N (20 µL, 23 mg, 1.4 mmol, 1.4 equiv.) were used. After 20 min, the reaction mixture was concentrated under reduced pressure. Then, the crude mixture was diluted with EtOAc and washed with H_2O (2 x 100 mL), brine (2 x 100 mL), dried over anhydrous Na_2SO_4 and concentrated under reduced pressure. **6m** as a colorless solid was obtained; Yield 99 % (0.800 g).

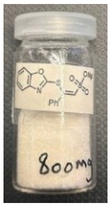

NMR Spectroscopy (see spectra):
^{1}H NMR (300 MHz, $CDCl_3$): δ 7.59 – 7.51 (m, 3H), 7.30 – 7.21 (m, 6H), 6.75 (s, 1H), 4.04 (s, 2H), 1.05 (s, 9H).
^{13}C NMR (75 MHz, $CDCl_3$): δ 157.69, 151.96, 150.34, 141.31, 136.64, 130.75, 128.75, 127.90, 125.49, 125.34, 124.70, 119.80, 110.08, 80.39, 31.88, 26.14.
E/*Z*-isomerism was determined via a *^{1}H,^{1}H-NOESY*-contact between 1-H and 2'-H, which is only possible in the (*Z*)-configured molecule.

HRMS (ESI+): m/z calc'd for **6m** $C_{20}H_{21}NO_4S_2Na$ $[M+Na]^+$: 426.0804, found: 426.0791.
mp: 93.2 – 95.3 °C.

2-Fluoroethyl (*Z*)-2-((1H-benzo[d]imidazol-2-yl)thio)-2-phenylethene-1-sulfonate (6n)

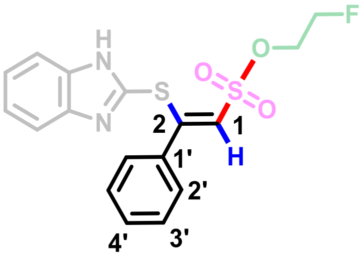

Following the general procedure D. with neopentyl 2-fluoroethyl 2-phenylethyne-1-sulfonate (**3aj**) (22.8 mg, 0.1 mmol), MeCN (1 mL), 2-mercaptobenzothiazole (17 mg, 0.1 mmol, 1 equiv.), Et_3N (20 µL, 23 mg, 1.4 mmol, 1.4 equiv.) were used. After 20 min, the reaction mixture was concentrated under reduced pressure. Then, the crude mixture was diluted with EtOAc and washed with H_2O (2 x 20 mL), brine (2 x 20 mL), dried over anhydrous Na_2SO_4 and concentrated under reduced pressure. **6n** as a colorless solid was obtained; Yield 84% (32 mg).

NMR Spectroscopy (see spectra):
^{1}H NMR (400 MHz, DMSO-d_6): δ 12.84 (s, 1H), 7.50 – 7.43 (m, 3H), 7.28 (d, J = 7.8 Hz, 1H), 7.24 – 7.17 (m, 3H), 7.16 – 7.02 (m, 3H), 4.86 – 4.81 (m, 1H), 4.74 – 4.69 (m, 1H), 4.68 – 4.64 (m, 1H), 4.61 – 4.56 (m, 1H).
^{13}C NMR (101 MHz, DMSO-d_6): δ 153.19, 143.83, 142.56, 136.27, 135.52, 130.63, 128.70, 128.57, 123.51, 123.43, 122.21, 119.11, 111.51, 82.67, 80.99, 70.80, 70.61.
E/*Z*-isomerism was determined via a *^{1}H,^{1}H-NOESY*-contact between 1-H and 2'-H, which is only possible in the (*Z*)-configured molecule.

HRMS (ESI+): m/z calc'd for **6n** $C_{17}H_{15}FN_2O_3S_2Na$ $[M+Na]^+$: 401.0402, found: 401.0396.
mp: 174.2 – 175.7 °C.

Neopentyl (*Z*)-2-((1H-benzo[d]imidazol-2-yl)thio)-2-phenylethene-1-sulfonate (6o)

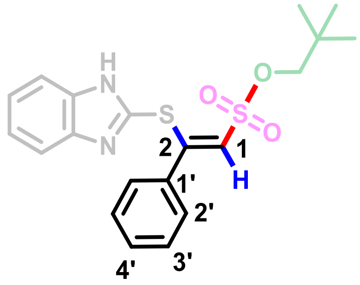

Following the general procedure D. with neopentyl 2-phenylethyne-1-sulfonate (**3ah**) (25.2 mg, 0.1 mmol), MeCN (1 mL), 2-mercaptobenzimidazole (15 mg, 0.1 mmol, 1 equiv.), Et_3N (20 µL, 23 mg, 1.4 mmol, 1.4 equiv.) were used. After 20 min, the reaction mixture was concentrated under reduced pressure. Then, the crude mixture was diluted with EtOAc and washed with H_2O (2 x 20 mL), brine (2 x 20 mL), dried over anhydrous Na_2SO_4 and concentrated under reduced pressure. **6n** as a colorless solid was obtained; Yield 94% (38 mg).

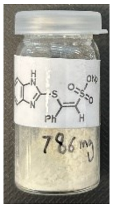

2 mmol-scale : Neopentyl 2-phenylethyne-1-sulfonate (**3ah**) (502 mg, 2.0 mmol), MeCN (15 mL), 2-mercaptobenzimidazole (300 mg, 2 mmol, 1 equiv.), Et_3N (20 µL, 23 mg, 1.4 mmol, 1.4 equiv.) were used. After 20 min, the reaction mixture was concentrated under reduced pressure. Then, the crude mixture was diluted with EtOAc and washed with H_2O (2 x 100 mL), brine (2 x 100 mL), dried over anhydrous Na_2SO_4 and concentrated under reduced pressure. **6o** as a colorless solid was obtained; Yield 98 % (786 mg).

NMR Spectroscopy (see spectra):
^{1}H NMR (300 MHz, $CDCl_3$): δ 7.59 – 7.53 (m, 2H), 7.49 – 7.40 (m, 2H), 7.25 – 7.16 (m, 5H), 6.73 (s, 1H), 4.04 (s, 2H), 1.06 (s, 9H).
^{13}C NMR (75 MHz, $CDCl_3$): δ 145.55, 144.05, 143.77, 133.59, 132.89, 132.53, 129.51, 127.85, 124.08, 123.46, 120.84, 117.92, 111.84, 80.17, 31.58, 25.81.
E/*Z*-isomerism was determined via a *^{1}H,^{1}H-NOESY*-contact between 1-H and 2'-H, which is only possible in the (*Z*)-configured molecule.

HRMS (ESI+): m/z calc'd for **6o** $C_{20}H_{22}N_2O_3S_2Na$ $[M+Na]^+$: 425.0964, found: 425.0957.
mp: 163.0 – 165.9 °C.

2-Phenylbenzo[4,5]imidazo[2,1-b]thiazole (7a)

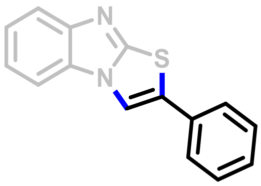

A 10 mL reaction flask was charged with neopentyl (*Z*)-2-((1*H*-benzo[d]imidazol-2-yl)thio)-2-phenylethene-1-sulfonate (**6o**) (40.2 mg, 0.1 mmol), cesium carbonate (98 mg, 0.3 mmol, 3 equiv.), DMF (1 mL) and a magnetic stir bar. The resulting mixture was stirred at 75 °C For 24 h. Then the reaction mixture was diluted with ethyl acetate, which was washed with water and brine, dried over $MgSO_4$, filtered, and concentrated in vacuo. Purification via column chromatography on silica gel (cyclohexane: ethyl acetate = 70:30, v/v) afforded **7a** as a colorless solid; Yield 80% (20 mg)

NMR Spectroscopy (see spectra):

^{1}H NMR (400 MHz, $CDCl_3$): δ 7.92 (s, 1H), 7.82 (dt, J = 8.2, 0.9 Hz, 1H), 7.69 (dt, J = 7.9, 1.0 Hz, 1H), 7.63 – 7.56 (m, 2H), 7.52 – 7.45 (m, 2H), 7.44 – 7.36 (m, 2H), 7.31 (ddd, J = 8.2, 7.2, 1.1 Hz, 1H).

^{13}C NMR (101 MHz, $CDCl_3$): δ 155.79, 147.74, 131.28, 129.68, 129.54, 129.29, 128.87, 125.88, 123.61, 121.20, 119.38, 112.35, 110.21.

HRMS (ESI+): m/z calc'd for **7a** $C_{15}H_{11}N_2S$ $[M+H]^+$: 251.0637, found: 251.0636.
mp: 65.4 – 67.2 °C.

Neopentyl (*Z*)-2-((2-oxo-2*H*-chromen-4-yl)oxy)-2-phenylethene-1-sulfonate (6p)

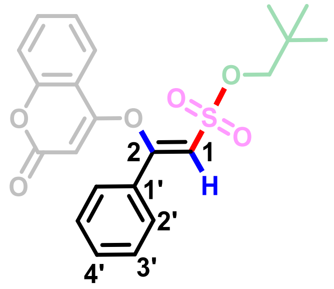

Following the general procedure D. with neopentyl 2-phenylethyne-1-sulfonate (**3ah**) (25.2 mg, 0.1 mmol), MeCN (1 mL), 4-hydroxycoumarin (16.2 mg, 0.1 mmol, 1 equiv.), Et_3N (20 µL, 23 mg, 1.4 mmol, 1.4 equiv.) were used. Purification via column chromatography on silica gel (cyclohexane: ethyl acetate = 70:30, v/v) afforded **6p** as a colorless solid; Yield 75% (31 mg).

NMR Spectroscopy (see spectra):
^{1}H NMR (300 MHz, $CDCl_3$): δ 8.09 (dd, *J* = 7.9, 1.6 Hz, 1H), 7.66 (ddd, *J* = 8.3, 7.4, 1.6 Hz, 1H), 7.59 – 7.52 (m, 3H), 7.51 – 7.34 (m, 4H), 6.77 (s, 1H), 5.50 (s, 1H), 3.93 (s, 2H), 1.00 (s, 9H).
^{13}C NMR (75 MHz, $CDCl_3$): δ 163.23, 161.54, 158.83, 153.60, 133.22, 132.79, 129.74, 129.68, 126.41, 124.58, 123.00, 117.02, 114.60, 113.63, 96.15, 80.13, 31.79, 26.03.
E/*Z*-isomerism was determined via a *^{1}H,^{1}H-NOESY*-contact between 1-H and 2'-H, which is only possible in the (*Z*)-configured molecule.

HRMS (ESI+): m/z calc'd for **6p** $C_{22}H_{22}O_6SNa$ $[M+Na]^+$: 437.1029, found: 437.1021.
mp: 164.9 – 165.7 °C.

Neopentyl (*E*)-2-((1*H*-benzo[d][1,2,3]triazol-1-yl)oxy)-2-phenylethene-1-sulfonate (6q)

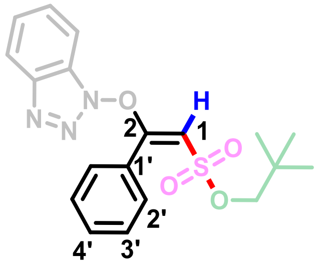

Following the general procedure D. with neopentyl 2-phenylethyne-1-sulfonate (**3ah**) (25.2 mg, 0.1 mmol), MeCN (1 mL), 1-hydroxybenzotriazole monohydrate (14 mg, 0.1 mmol, 1 equiv.), Et_3N (20 µL, 23 mg, 1.4 mmol, 1.4 equiv.) were used. Purification via column chromatography on silica gel (cyclohexane: ethyl acetate = 70:30, v/v) afforded **6q** as a colorless solid; Yield 90% (35 mg).

NMR Spectroscopy (see spectra):
^{1}H NMR (400 MHz, $CDCl_3$): δ 8.12 (d, *J* = 8.4 Hz, 1H), 7.87 (d, *J* = 7.0 Hz, 2H), 7.67 – 7.47 (m, 6H), 5.55 (s, 1H), 3.62 (s, 2H), 0.80 (s, 9H).
^{13}C NMR (101 MHz, $CDCl_3$): δ 168.65, 144.03, 133.22, 130.62, 130.34, 129.24, 128.23, 128.12, 126.33, 121.62, 108.91, 105.70, 80.81, 32.22, 26.54.
No *^{1}H,^{1}H-NOESY*-contact was observed between 1-H and any other proton, especially not with 2'-H, which suggests the (*E*)-configuration.

HRMS (ESI+): m/z calc'd for **6q** $C_{19}H_{21}N_3O_4SNa$ $[M+Na]^+$: 410.1145, found: 410.1143.

mp: 74.5 – 76.3 °C.

(*Z*)-2-((Neopentyloxy)sulfonyl)-1-phenylvinyl 3-(1*H*-indol-3-yl)propanoate (6r)

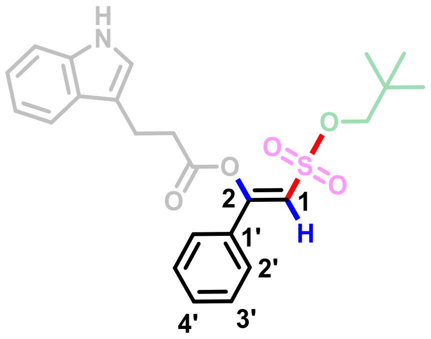

Following the general procedure D. with neopentyl 2-phenylethyne-1-sulfonate (**3ah**) (25.2 mg, 0.1 mmol), MeCN (1 mL), 3-indolepropionic acid (19 mg, 0.1 mmol, 1 equiv.), Et_3N (20 µL, 23 mg, 1.4 mmol, 1.4 equiv.) were used. Purification via column chromatography on silica gel (cyclohexane: ethyl acetate = 70:30, v/v) afforded **6r** as a a colorless solid; Yield 84% (37 mg).

NMR Spectroscopy (see spectra):
^{1}H NMR (300 MHz, $CDCl_3$): δ 8.05 (s, 1H), 7.70 – 7.63 (m, 1H), 7.49 – 7.44 (m, 1H), 7.42 (dt, *J* = 8.1, 1.0 Hz, 1H), 7.37 – 7.30 (m, 4H), 7.25 (ddd, *J* = 8.1, 7.0, 1.2 Hz, 1H), 7.17 (ddd, *J* = 7.9, 7.0, 1.0 Hz, 1H), 7.07 – 7.03 (m, 1H), 6.58 (s, 1H), 3.90 (s, 2H), 3.29 – 3.23 (m, 2H), 3.14 – 3.09 (m, 2H), 1.02 (s, 9H).
^{13}C NMR (75 MHz, $CDCl_3$): δ 170.25, 158.35, 136.33, 131.80, 131.75, 129.03, 127.10, 126.12, 122.18, 121.90, 119.49, 118.71, 114.18, 111.34, 111.25, 80.10, 34.72, 31.76, 26.95, 26.11.
E/*Z*-isomerism was determined via a *^{1}H,^{1}H-NOESY*-contact between 1-H and 2'-H, which is only possible in the (*Z*)-configured molecule.

HRMS (ESI+): m/z calc'd for **6r** $C_{24}H_{27}NO_5SNa$ $[M+Na]^+$: 464.1502, found: 464.1487.
mp: 104.2 – 106.4 °C.

(*Z*)-2-((Neopentyloxy)sulfonyl)-1-phenylvinyl 2-(1,8-diethyl-1,3,4,9-tetrahydropyrano[3,4-b]indol-1-yl)acetate (6s)

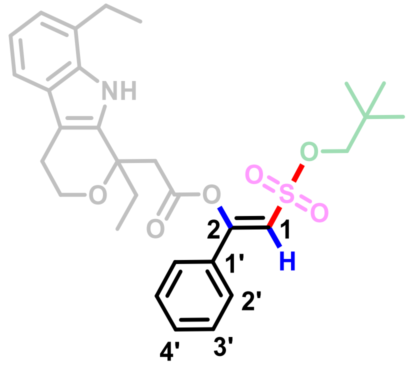

Following the general procedure D. with neopentyl 2-phenylethyne-1-sulfonate (**3ah)** (25.2 mg, 0.1 mmol), MeCN (1 mL), etodolac (29 mg, 0.1 mmol, 1 equiv.), Et_3N (20 µL, 23 mg, 1.4 mmol, 1.4 equiv.) were used. Purification via column chromatography on silica gel (cyclohexane: ethyl acetate = 70:30, v/v) afforded **6s** as a yellow solid; Yield 83% (45 mg).

NMR Spectroscopy (see spectra):
^{1}H NMR (300 MHz, $CDCl_3$): δ 8.54 (s, 1H), 7.51 – 7.39 (m, 4H), 7.37 – 7.30 (m, 2H), 7.10 (t, *J* = 7.5 Hz, 1H), 7.02 (d, *J* = 6.9 Hz, 1H), 6.63 (s, 1H), 4.18 – 4.01 (m, 2H), 3.88 (s, 2H), 3.36 (d, *J* = 1.6 Hz, 2H), 2.96 – 2.69 (m, 4H), 2.30 – 2.07 (m, 2H), 1.24 (t, *J* = 7.6 Hz, 3H), 1.01 (s, 9H), 0.93 (t, *J* = 7.3 Hz, 3H).
^{13}C NMR (75 MHz, $CDCl_3$): δ 169.31, 157.87, 135.00, 134.62, 131.98, 131.50, 129.17, 126.76, 126.10, 120.56, 119.74, 115.99, 111.54, 108.92, 80.10, 74.79, 60.75, 43.05, 31.75, 31.08, 26.08, 24.04, 22.35, 13.67, 7.62.
E/*Z*-isomerism was determined via a *^{1}H,^{1}H-NOESY*-contact between 1-H and 2'-H, which is only possible in the (*Z*)-configured molecule.

HRMS (ESI+): m/z calc'd for **6s** $C_{30}H_{37}NO_6SNa$ $[M+Na]^+$: 562.2234, found: 562.2227.
mp: 144.5 – 146.7 °C.

(*Z*)-2-((Neopentyloxy)sulfonyl)-1-phenylvinyl 4-(*N,N*-dipropylsulfamoyl)benzoate (6t)

Following the general procedure D. with neopentyl 2-phenylethyne-1-sulfonate (**3ah**) (25.2 mg, 0.1 mmol), MeCN (1 mL), probenecid (28.5 mg, 0.1 mmol, 1 equiv.), Et_3N (20 µL, 23 mg, 1.4 mmol, 1.4 equiv.) were used. Purification via column chromatography on silica gel (cyclohexane: ethyl acetate = 70:30, v/v) afforded **6t** as a colorless solid; Yield 45% (24 mg).

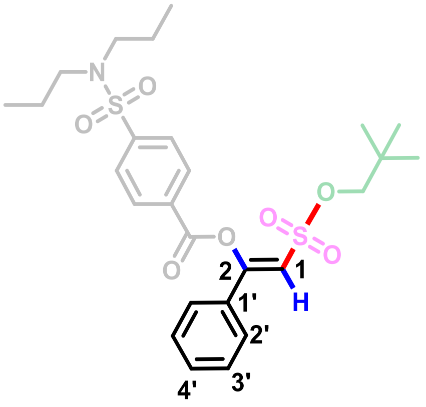

NMR Spectroscopy (see spectra):
^{1}H NMR (300 MHz, $CDCl_3$): δ 8.33 (d, *J* = 8.5 Hz, 2H), 7.97 (d, *J* = 8.6 Hz, 2H), 7.62 – 7.43 (m, 5H), 6.73 (s, 1H), 3.91 (s, 2H), 3.19 – 3.10 (m, 4H), 1.70 – 1.52 (m, 5H), 0.99 (s, 9H), 0.92 (t, *J* = 7.4 Hz, 6H).
^{13}C NMR (75 MHz, $CDCl_3$): δ 162.45, 158.16, 145.38, 132.14, 131.47, 131.19, 129.30, 127.36, 126.14, 112.27, 80.24, 50.19, 31.76, 26.05, 22.15, 11.19.
E/*Z*-isomerism was determined via a *^{1}H,^{1}H-NOESY*-contact between 1-H and 2'-H, which is only possible in the (*Z*)-configured molecule.

HRMS (ESI+): m/z calc'd for **6t** $C_{26}H_{35}NO_7S_2Na$ $[M+Na]^+$: 560.1747, found: 560.1742.
mp: 130.9 – 132.6 °C.

(*Z*)-2-((Neopentyloxy)sulfonyl)-1-phenylvinyl 5-((3aS,4S,6aR)-2-oxohexahydro-1*H*-thieno[3,4-d]imidazol-4-yl)pentanoate (6u)

Following the general procedure D. with neopentyl 2-phenylethyne-1-sulfonate (**3ah**) (25.2 mg, 0.1 mmol), DMF (1 mL), D-(+)-biotin (24.5 mg, 0.1 mmol, 1 equiv.), Et_3N (20 µL, 23 mg, 1.4 mmol, 1.4 equiv.) were used. The reaction mixture was diluted with EtOAc and washed with H_2O (2 x 25 mL), brine (2 x 25 mL), dried over anhydrous Na_2SO_4 and concentrated under reduced pressure. **6u** as a colorless solid was obtained; Yield 82% (41 mg).

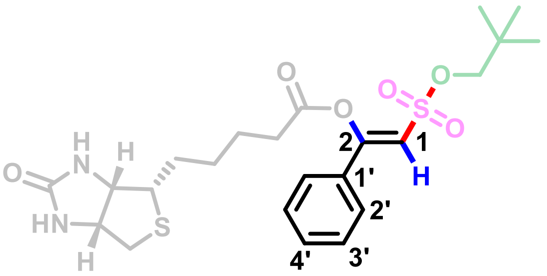

2 mmol-scale : neopentyl 2-phenylethyne-1-sulfonate (**3ah**) (0.502 mg, 2.0 mmol), MeCN (15 mL), D-(+)-biotin (244 mg, 2 mmol, 1 equiv.), Et_3N (20 µL, 23 mg, 1.4 mmol, 1.4 equiv.) were used. Once the time has passed, the reaction mixture was diluted with EtOAc and washed with H_2O (2 x 100 mL), brine (2 x 100 mL), dried over anhydrous Na_2SO_4 and concentrated under reduced pressure. **6u** as a colorless solid was obtained; Yield 93 % (920 mg).

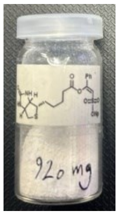

NMR Spectroscopy (see spectra):
^{1}H NMR (300 MHz, $CDCl_3$): δ 7.56 – 7.43 (m, 5H), 6.57 (s, 1H), 6.29 (s, 1H), 5.28 (s, 1H), 4.51 (dd, *J* = 7.9, 4.7 Hz, 1H), 4.33 (dd, *J* = 7.6, 4.5 Hz, 1H), 3.89 (s, 2H), 3.17 (td, *J* = 7.2, 4.5 Hz, 1H), 2.98 – 2.86 (m, 1H), 2.81 – 2.69 (m, 3H), 1.89 – 1.66 (m, 5H), 1.56 (q, *J* = 7.2 Hz, 2H), 1.01 (s, 9H).
^{13}C NMR (75 MHz, $CDCl_3$): δ 170.53, 163.71, 158.40, 131.98, 131.92, 129.20, 126.13, 111.42, 80.15, 61.91, 60.13, 55.66, 40.56, 33.62, 31.76, 28.22, 26.10, 24.36.
E/*Z*-isomerism was determined via a *^{1}H,^{1}H-NOESY*-contact between 1-H and 2'-H, which is only possible in the (*Z*)-configured molecule.

HRMS (ESI+): m/z calc'd for **6u** $C_{23}H_{32}N_2O_6S_2Na$ $[M+Na]^+$: 519.1594, found: 519.1588.
Specific rotation $[\alpha]_D^{25}$: +47.8 (c = 0.5, $CHCl_3$).
mp: 137.1 – 139.3 °C.

(5α-Cholestan-3β-yl) (Z)-2-(benzo[d]thiazol-2-ylthio)-2-phenylethene-1-sulfonate (6v)

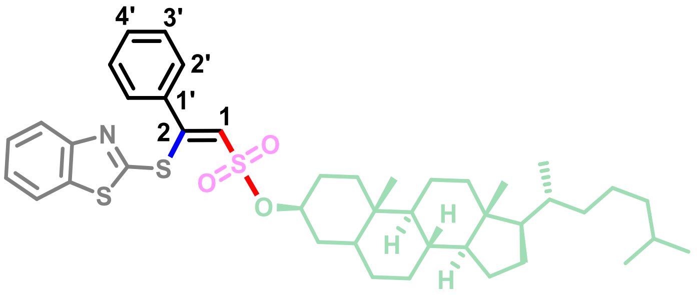

Following the general procedure D. with neopentyl 2-phenylethyne-1-sulfonate (**3azb**) (55.3 mg, 0.1 mmol), MeCN (1 mL), DCM (1mL), 2-mercaptobenzimidazole (15 mg, 0.1 mmol, 1 equiv.), Et_3N (20 µL, 23 mg, 1.4 mmol, 1.4 equiv.) were used. After 20 min, the reaction mixture was concentrated under reduced pressure. Then, the crude mixture was diluted with EtOAc and washed with H_2O (2 x 20 mL), brine (2 x 20 mL), dried over anhydrous Na_2SO4 and concentrated under reduced pressure. Purification via column chromatography on silica gel (cyclohexane: ethyl acetate = 70:30, v/v) afforded **6v** as a colorless oil; Yield 95 % (68 mg).

NMR Spectroscopy (see spectra):
^{1}H NMR (300 MHz, $CDCl_3$): δ 7.88 (d, J = 8.1 Hz, 1H), 7.70 – 7.62 (m, 1H), 7.60 – 7.53 (m, 2H), 7.44 – 7.38 (m, 1H), 7.35 – 7.25 (m, 4H), 6.80 (s, 1H), 4.78 (tt, J = 10.8, 5.1 Hz, 1H), 2.16 – 2.05 (m, 1H), 1.98 (dt, J = 12.4, 3.2 Hz, 1H), 1.91 – 1.76 (m, 4H), 1.72 – 1.61 (m, 3H), 1.59 – 1.48 (m, 2H), 1.41 – 1.22 (m, 9H), 1.20 – 0.96 (m, 10H), 0.94 – 0.86 (m, 10H), 0.84 (s, 3H), 0.66 (s, 3H).
^{13}C NMR (101 MHz, $CDCl_3$): δ 160.21, 152.68, 150.39, 137.09, 136.18, 130.76, 128.81, 128.66, 127.74, 126.35, 125.41, 122.95, 121.00, 83.81, 56.36, 56.26, 54.06, 44.90, 42.58, 39.92, 39.52, 36.89, 36.17, 35.79, 35.40, 35.30, 35.18, 31.90, 28.77, 28.53, 28.24, 28.03, 26.93, 24.20, 23.84, 22.84, 22.58, 21.22, 18.68, 12.20, 12.08.
E/*Z*-isomerism was determined via a *^{1}H,^{1}H-NOESY*-contact between 1-H and 2'-H, which is only possible in the (*Z*)-configured molecule.

HRMS (ESI+): m/z calc'd for **6v** $C_{42}H_{57}NO_3S_3Na$ $[M+Na]^+$: 742.3393, found: 742.3377.
Specific rotation $[\alpha]_D^{25}$: +5 (c = 0.5, $CHCl_3$).

(Z) (5α-Cholestan-3β-oxy)sulfonyl -1-phenylvinyl 2-(1,8-diethyl-1,3,4,9-tetrahydropyrano[3,4-b]indol-1-yl)acetate (6w)

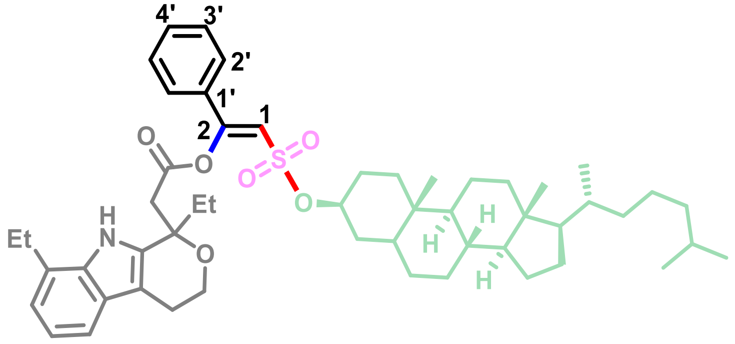

Following the general procedure D. with neopentyl 2-phenylethyne-1-sulfonate (**3azb**) (55.3 mg, 0.1 mmol), MeCN (1 mL), DMF (1mL), etodolac (29 mg, 0.1 mmol, 1 equiv.), Et_3N (20 µL, 23 mg, 1.4 mmol, 1.4 equiv.) were used. After 48 h, the reaction mixture was concentrated under reduced pressure. Then, the crude mixture was diluted with EtOAc and washed with H_2O (2 x 20 mL), brine (2 x 20 mL), dried over anhydrous Na_2SO4 and concentrated under reduced pressure. Purification via column chromatography on silica gel (cyclohexane: ethyl acetate = 70:30, v/v) afforded **6w** as a colorless oil; Yield 47 % (39 mg).

NMR Spectroscopy (see spectra):
^{1}H NMR (300 MHz, $CDCl_3$): δ 8.57 (d, J = 3.6 Hz, 1H), 7.53 – 7.29 (m, 6H), 7.09 (t, J = 7.5 Hz, 1H), 7.01 (d, J = 6.1 Hz, 1H), 6.62 (s, 1H), 4.69 – 4.42 (m, 1H), 4.19 – 3.97 (m, 2H), 3.44 – 3.24 (m, 2H), 2.97 – 2.64 (m, 4H), 2.26 – 2.08 (m, 2H), 1.98 (dt, J = 12.2, 3.2 Hz, 2H), 1.84 – 1.53 (m, 10H), 1.39 – 1.20 (m, 15H), 1.17 – 0.97 (m, 10H), 0.93 – 0.87 (m, 10H), 0.82 (d, J = 2.2 Hz, 3H), 0.66 (s, 3H).
^{13}C NMR (75 MHz, $CDCl_3$): δ 169.37, 156.80, 135.05, 134.60, 131.81, 131.65, 129.14, 126.74, 126.05, 120.54, 119.72, 115.98, 113.16, 108.89, 83.51, 74.75, 60.74, 56.37, 56.26, 54.08, 44.89, 43.04, 42.59, 39.93, 39.53, 36.86,

36.83, 36.17, 35.80, 35.40, 35.24, 35.08, 31.91, 31.06, 28.52, 28.25, 28.04, 26.94, 24.20, 24.07, 23.84, 22.85, 22.59, 22.36, 21.22, 18.68, 13.69, 12.15, 12.08, 7.61.
E/*Z*-isomerism was determined via a *^{1}H,^{1}H-NOESY*-contact between 1-H and 2'-H, which is only possible in the (*Z*)-configured molecule.

HRMS (ESI+): m/z calc'd for **6w** $C_{52}H_{73}NO_6SNa$ $[M+Na]^+$: 862.5051, found: 862.5034.
Specific rotation $[\alpha]_D^{25}$: +8 (c = 0.5, $CHCl_3$).

6-Bromohex-5-yn-1-yl 2-(11-oxo-6,11-dihydrodibenzo[*b*,*e*]oxepin-2-yl)acetate (2r)

Step 1: To a stirred solution of DCC (1.11 g, 5.55 mmol, 1.5 equiv.) in DCM (40 mL) was added isoxepac (992 mg, 3.7 mmol, 1 equiv) at room temperature. Then, a solution of 5-hexyn-1-ol (410µL, 362 mg, 3.7 mmol, 1 equiv) and DMAP (68 mg, 0.555 mmol, 0.15 equiv.) in DCM (40 mL) was added dropwise. The resultant mixture was allowed to stired 12 h at room temperature and then passed through as short pad of celite. The filtrate was concentrated; purification via column chromatography on silica gel (cyclohexane: ethyl acetate = 85:15, v/v) afforded hex-5-yn-1-yl 2-(11-oxo-6,11-dihydrodibenzo[*b*,*e*]oxepin-2-yl)acetate as a colorless oil; Yield 86 % (1.112 g).

NMR Spectroscopy (see spectra):
^{1}H NMR (300 MHz, $CDCl_3$): δ 8.11 (d, *J* = 2.4 Hz, 1H), 7.89 (dd, *J* = 7.6, 1.5 Hz, 1H), 7.56 (td, *J* = 7.4, 1.5 Hz, 1H), 7.51 – 7.34 (m, 3H), 7.03 (d, *J* = 8.4 Hz, 1H), 5.19 (s, 2H), 4.13 (t, *J* = 6.4 Hz, 2H), 3.64 (s, 2H), 2.21 (td, *J* = 7.0, 2.7 Hz, 2H), 1.94 (t, *J* = 2.7 Hz, 1H), 1.82 – 1.71 (m, 2H), 1.61 (t, *J* = 3.8 Hz, 1H), 1.58 – 1.52 (m, 1H).
^{13}C NMR (75 MHz, $CDCl_3$): δ 190.9, 171.5, 160.5, 140.5, 136.4, 135.6, 132.8, 132.5, 129.5, 129.3, 127.9, 127.8, 125.1, 121.1, 83.9, 73.7, 68.8, 64.5, 40.3, 27.6, 24.9, 18.0.

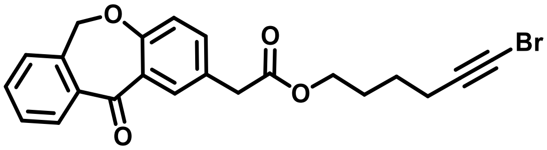

Step 2: $AgNO_3$ (51 mg, 0.3 mmol, 0.1 equiv.) was added to a solution of hex-5-yn-1-yl 2-(11-oxo-6,11-dihydrodibenzo[b,e]oxepin-2-yl)acetate (1.045 g, 3 mmol) in acetone (80 mL). Then NBS (597 mg, 3.36 mmol, 1.12 equiv) was added in portion. The mixture was stirred for 3 h at room temperature, and then concentrated in vacuo. The residue was dissolved in (cyclohexane: ethyl acetate = 90:10, v/v) and filtered through a short column of silica gel. Solvent was removed in vacuo to afford a pale-yellow oil of **2r** (1.260 g, 99%).

NMR Spectroscopy (see spectra):
^{1}H NMR (400 MHz, $CDCl_3$): δ 8.11 (d, *J* = 2.4 Hz, 1H), 7.88 (dd, *J* = 7.7, 1.4 Hz, 1H), 7.54 (td, *J* = 7.5, 1.5 Hz, 1H), 7.46 (td, *J* = 7.6, 1.3 Hz, 1H), 7.42 (dd, *J* = 8.4, 2.4 Hz, 1H), 7.35 (dd, *J* = 7.4, 1.3 Hz, 1H), 7.02 (d, *J* = 8.4 Hz, 1H), 5.17 (s, 2H), 4.11 (t, *J* = 6.4 Hz, 2H), 3.63 (s, 2H), 2.22 (t, *J* = 7.0 Hz, 2H), 1.77 – 1.69 (m, 2H), 1.59 – 1.51 (m, 2H).
^{13}C NMR (101 MHz, $CDCl_3$): δ 190.8, 171.4, 160.5, 140.4, 136.3, 135.6, 132.8, 132.4, 129.5, 129.3, 127.9, 127.8, 125.1, 121.1, 79.7, 73.6, 64.4, 40.3, 38.4, 27.6, 24.8, 19.3.

HRMS (ESI+): m/z calc'd for **2r** $C_{22}H_{19}BrO_4Na$ $[M+Na]^+$: 449.0352 (^{79}Br), found: 449.0359.

(*R*)-2,5,7,8-tetramethyl-2-((4*R*,8*R*)-4,8,12-trimethyltridecyl)chroman-6-yl 6-bromohex-5-ynoate (2s)

Step 1: To a stirred solution of DCC (1.55 g, 7.5 mmol, 1.5 equiv.) in DCM (50 mL) was added 5-Hexynoic acid (552 µL , 560 mg, 5 mmol, 1 equiv) at room temperature. Then, a solution of (+)-α-tocopherol (2.15 g, 5 mmol, 1 equiv) and DMAP (92 mg, 0.75 mmol, 0.15 equiv.) in DCM (50 mL) was added dropwise. The resultant mixture was allowed to stired 12 h at room temperature and then passed through as short pad of celite. The filtrate was concentrated; purification via column chromatography on silica gel (cyclohexane: ethyl acetate = 85:15, v/v) afforded (*R*)-2,5,7,8-tetramethyl-2-((4*R*,8*R*)-4,8,12-trimethyltridecyl)chroman-6-yl hex-5-ynoate as a colorless oil; Yield 94 % (2.463 g).

NMR Spectroscopy (see spectra):
^{1}H NMR (300 MHz, $CDCl_3$): δ 2.76 (t, *J* = 7.5 Hz, 2H), 2.59 (t, *J* = 6.8 Hz, 2H), 2.37 (td, *J* = 6.9, 2.6 Hz, 2H), 2.09 (s, 3H), 2.06 – 1.94 (m, 9H), 1.87 – 1.68 (m, 2H), 1.55 – 1.44 (m, 3H), 1.43 – 1.21 (m, 15H), 1.18 – 1.04 (m, 6H), 0.90 – 0.82 (m, 12H).
^{13}C NMR (75 MHz, $CDCl_3$): δ 171.7, 149.4, 140.4, 126.6, 124.9, 123.1, 117.4, 83.1, 75.1, 69.4, 39.4, 37.5, 37.4, 37.3, 32.8, 32.7, 32.6, 31.1, 28.0, 26.9, 24.8, 24.5, 23.7, 22.7, 22.7, 21.1, 20.6, 19.8, 19.7, 17.9, 13.0, 12.2, 11.9.

HRMS (ESI+): m/z calc'd for $C_{35}H_{56}NO_3$ $[M+NH_4]^+$: 542.4550, found: 542.4568.

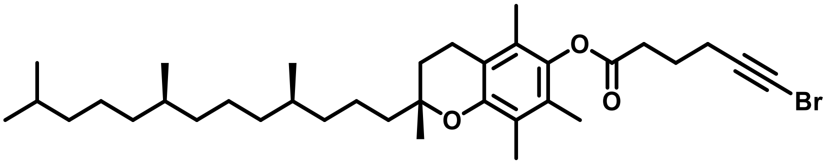

Step 2: $AgNO_3$ (68 mg, 0.4 mmol, 0.1 equiv.) was added to a solution of (R)-2,5,7,8 tetramethyl-2-((4R,8R)-4,8,12 trimethyltridecyl)chroman-6-yl hex-5 ynoate (2.099 g, 4 mmol) in acetone (100 mL). Then NBS (0.8 g, 4.5 mmol, 1.12 equiv) was added in portion. The mixture was stirred for 3 h at room temperature, and then concentrated in vacuo. The residue was dissolved in (cyclohexane: ethyl acetate = 90:10, v/v) and filtered through a short column of silica gel. Solvent was removed in vacuo to afford a pale-yellow oil of **2s** (2.385 g, 99%).

NMR Spectroscopy (see spectra):
^{1}H NMR (300 MHz, $CDCl_3$): δ 2.74 (t, *J* = 7.4 Hz, 2H), 2.59 (t, *J* = 6.8 Hz, 2H), 2.40 (t, *J* = 6.9 Hz, 2H), 2.09 (s, 3H), 2.05 – 1.95 (m, 8H), 1.87 – 1.69 (m, 2H), 1.61 – 1.47 (m, 4H), 1.41 – 1.19 (m, 14H), 1.18 – 1.01 (m, 7H), 0.89 – 0.83 (m, 12H).
^{13}C NMR (75 MHz, $CDCl_3$): δ 171.6, 149.4, 140.4, 126.6, 124.9, 123.1, 117.4, 79.0, 75.1, 39.4, 39.1, 37.5, 37.4, 37.3, 32.8, 32.7, 32.6, 31.1, 28.0, 26.9, 24.8, 24.5, 23.6, 22.8, 22.7, 21.1, 20.6, 19.8, 19.7, 19.6, 19.2, 13.0, 12.2, 11.9.

HRMS (ESI+): m/z calc'd for **2s** $C_{35}H_{55}BrO_3Na$ $[M+Na]^+$: 625.3227 (^{79}Br), found: 625.3210.

^{1}H NMR (300 MHz, $CDCl_3$) of **BP1** (see procedure)

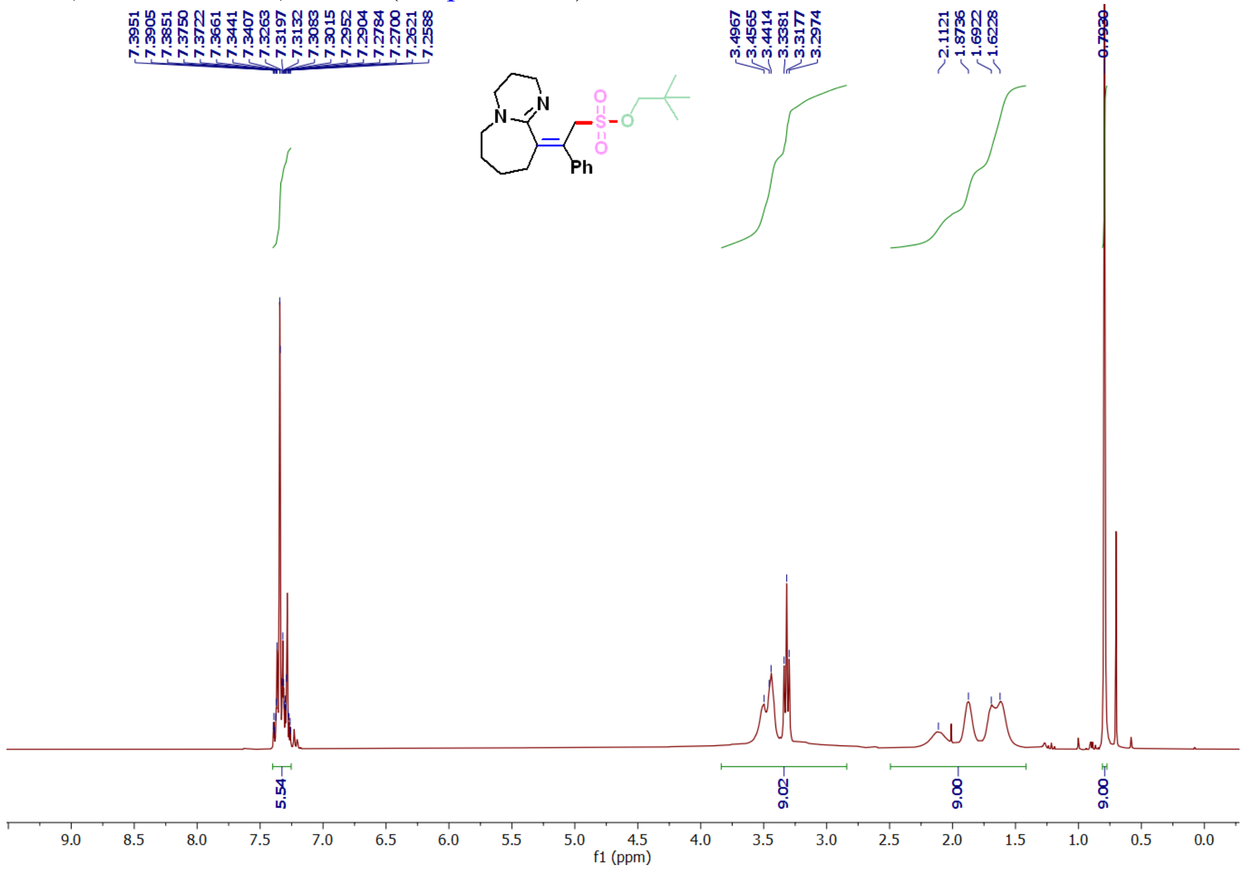

^{13}C NMR (101MHz, $CDCl_3$) of **BP1**

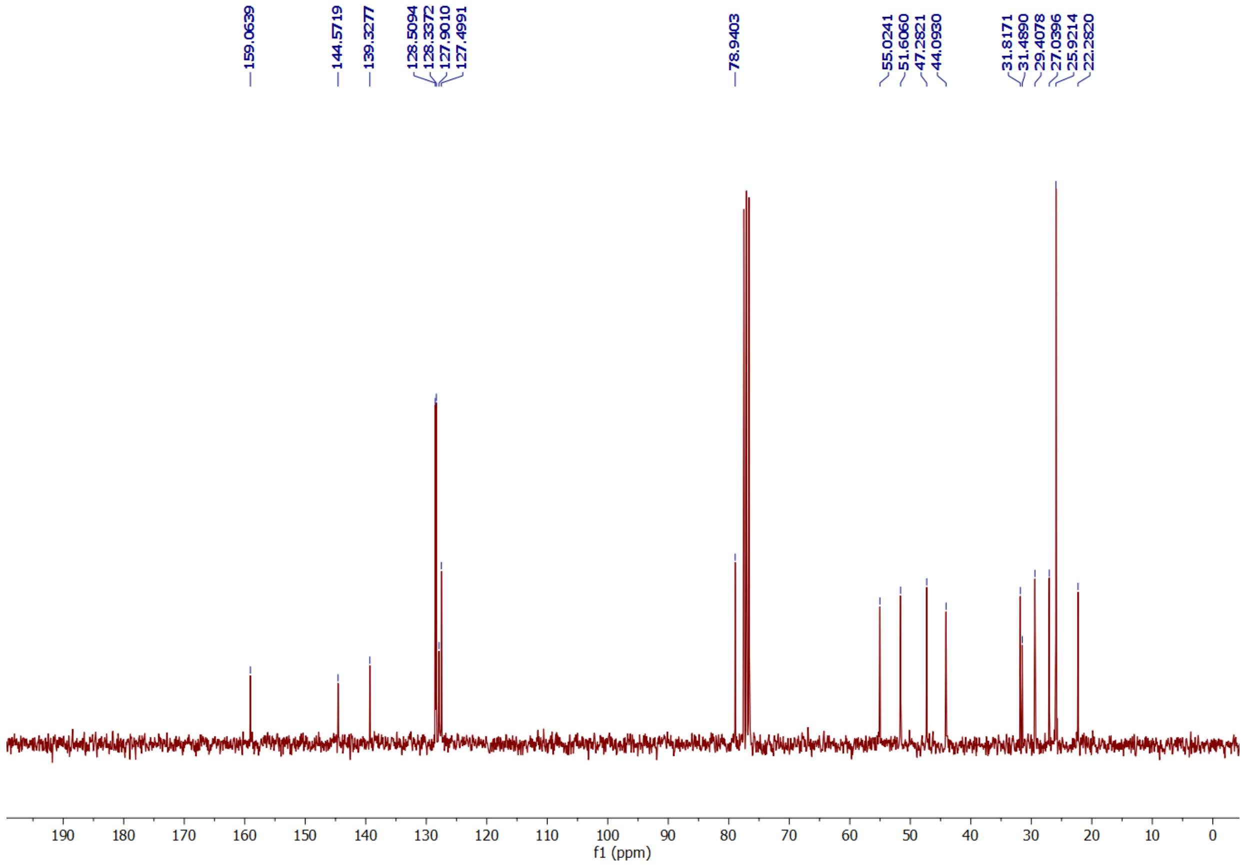

^{1}H NMR (300 MHz, $CDCl_3$) of **BP2** (see procedure)

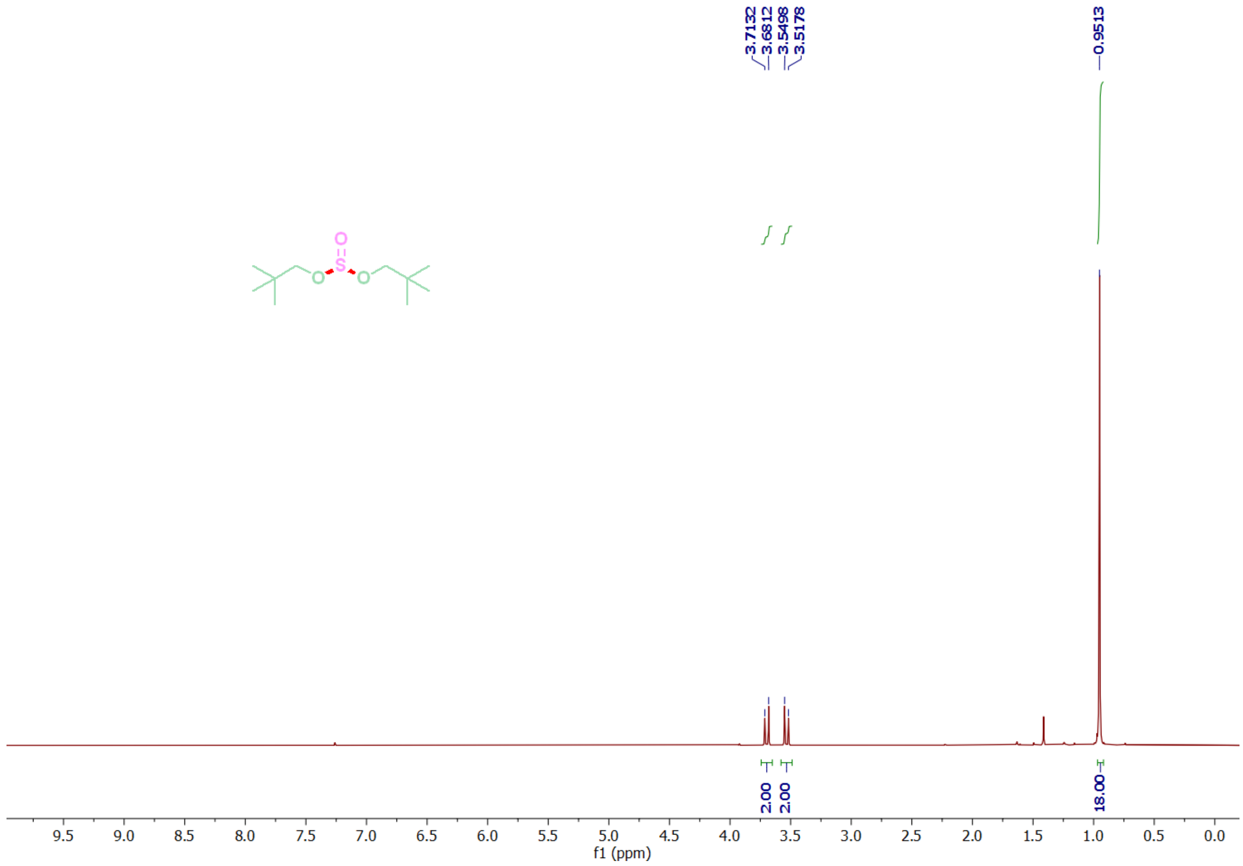

^{13}C NMR (75 MHz, $CDCl_3$) of **BP2**

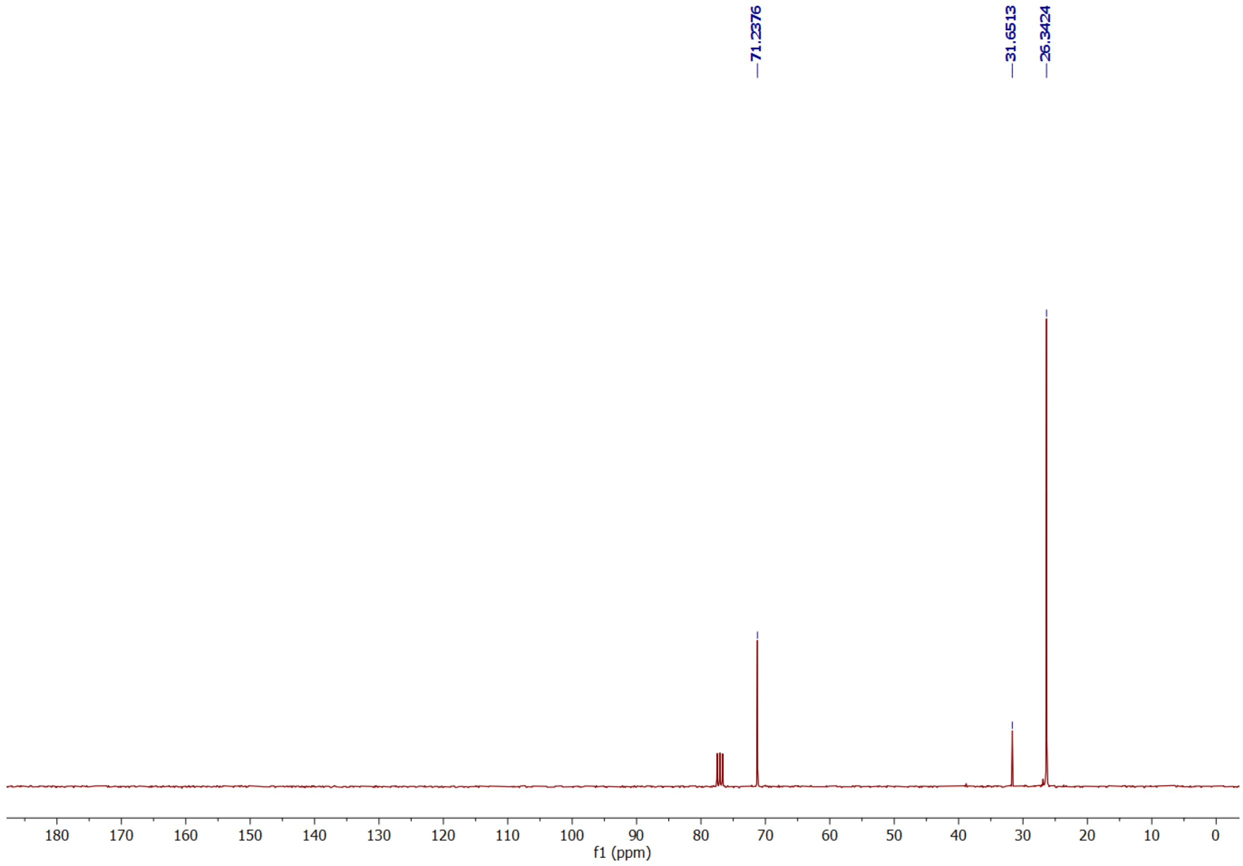

^{1}H NMR (300 MHz, $CDCl_3$) of **BP3** (see procedure)

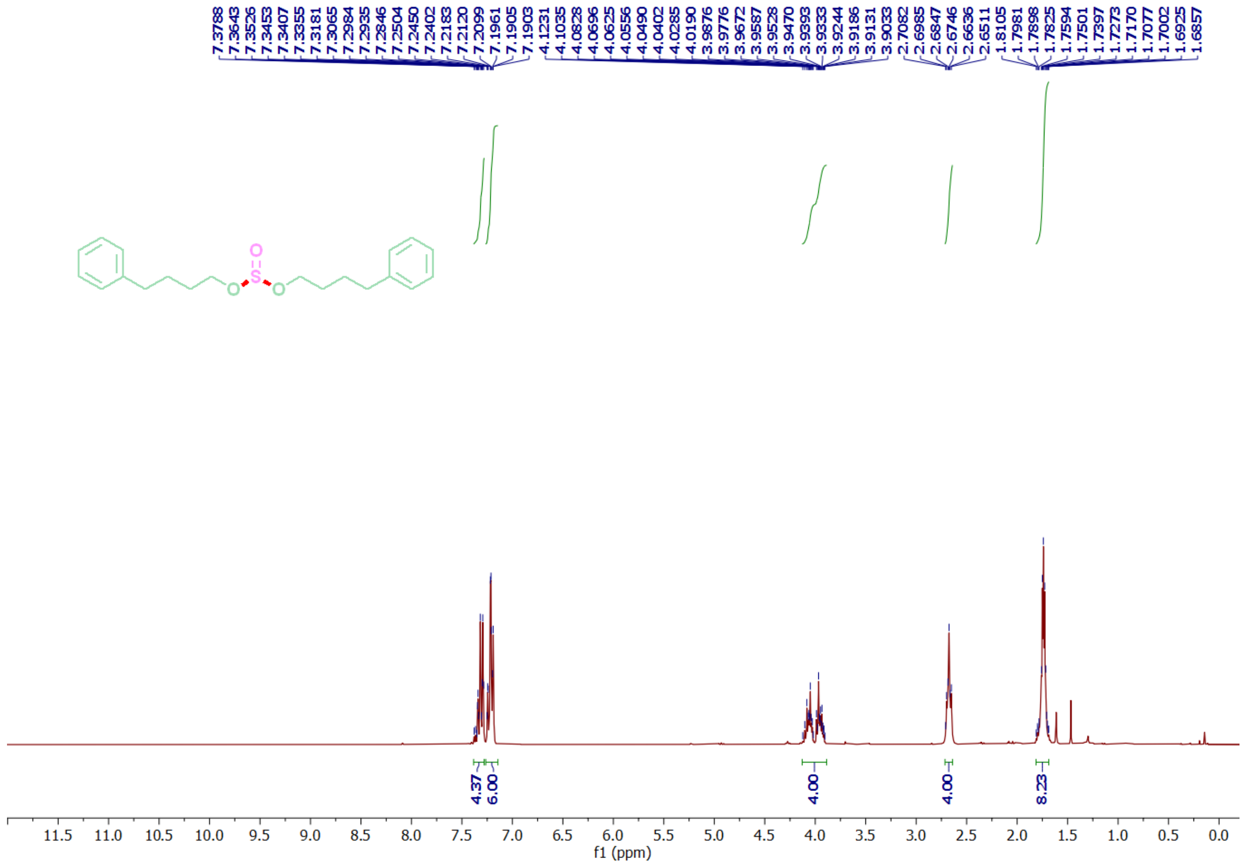

^{13}C NMR (75 MHz, $CDCl_3$) of **BP2**

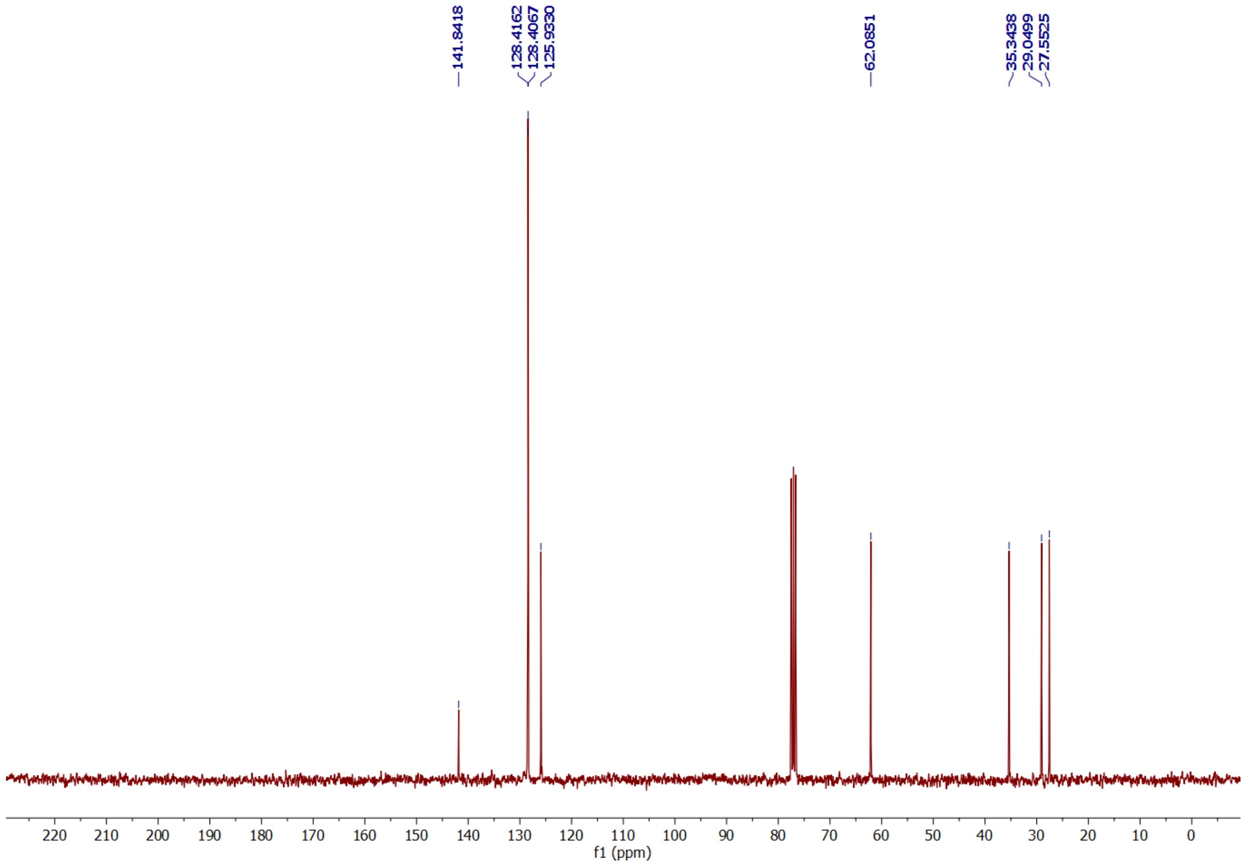

^{1}H NMR (300 MHz, $CDCl_3$) of **(4-iodophenyl)boronic acid-*d2*** (see procedure)

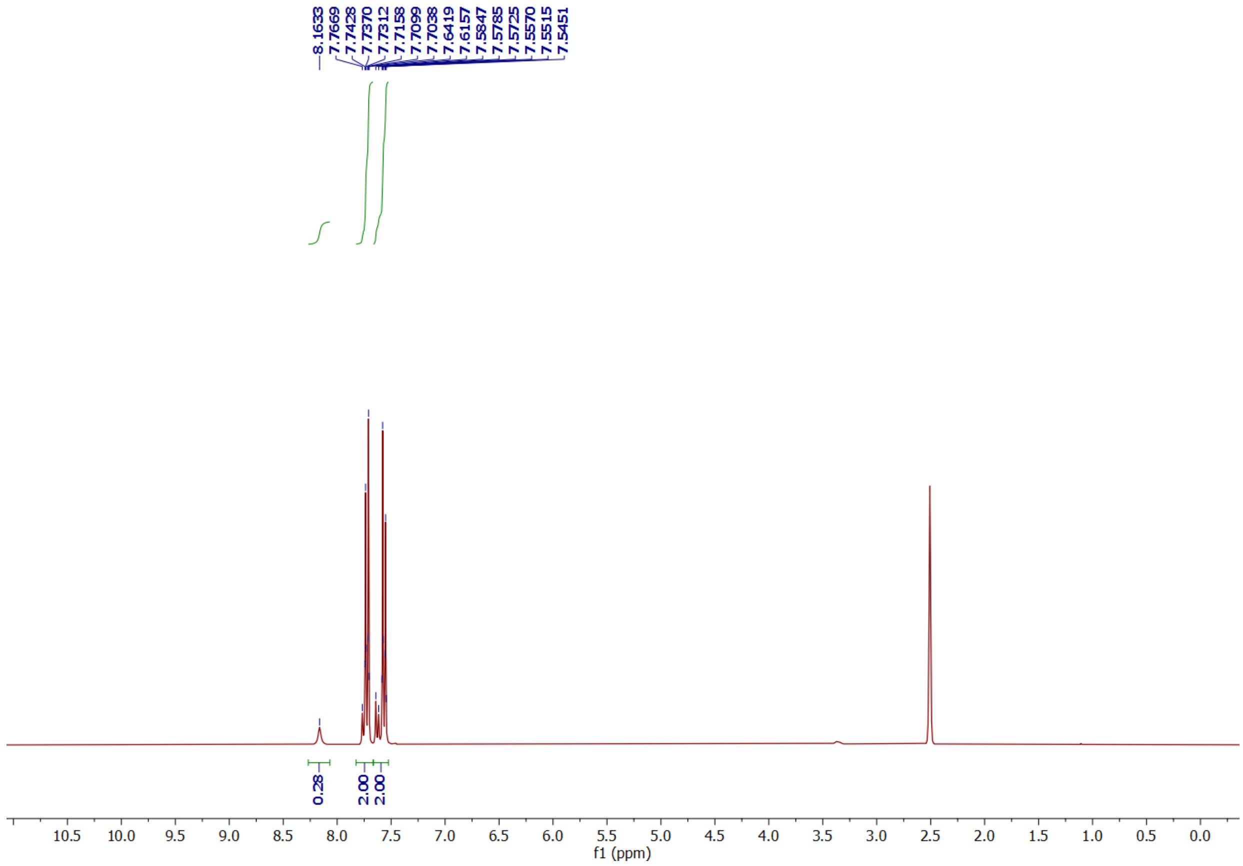

^{1}H NMR (300 MHz, $CDCl_3$) of **benzo[d]oxazole-2-thiol-*d*** (see procedure)

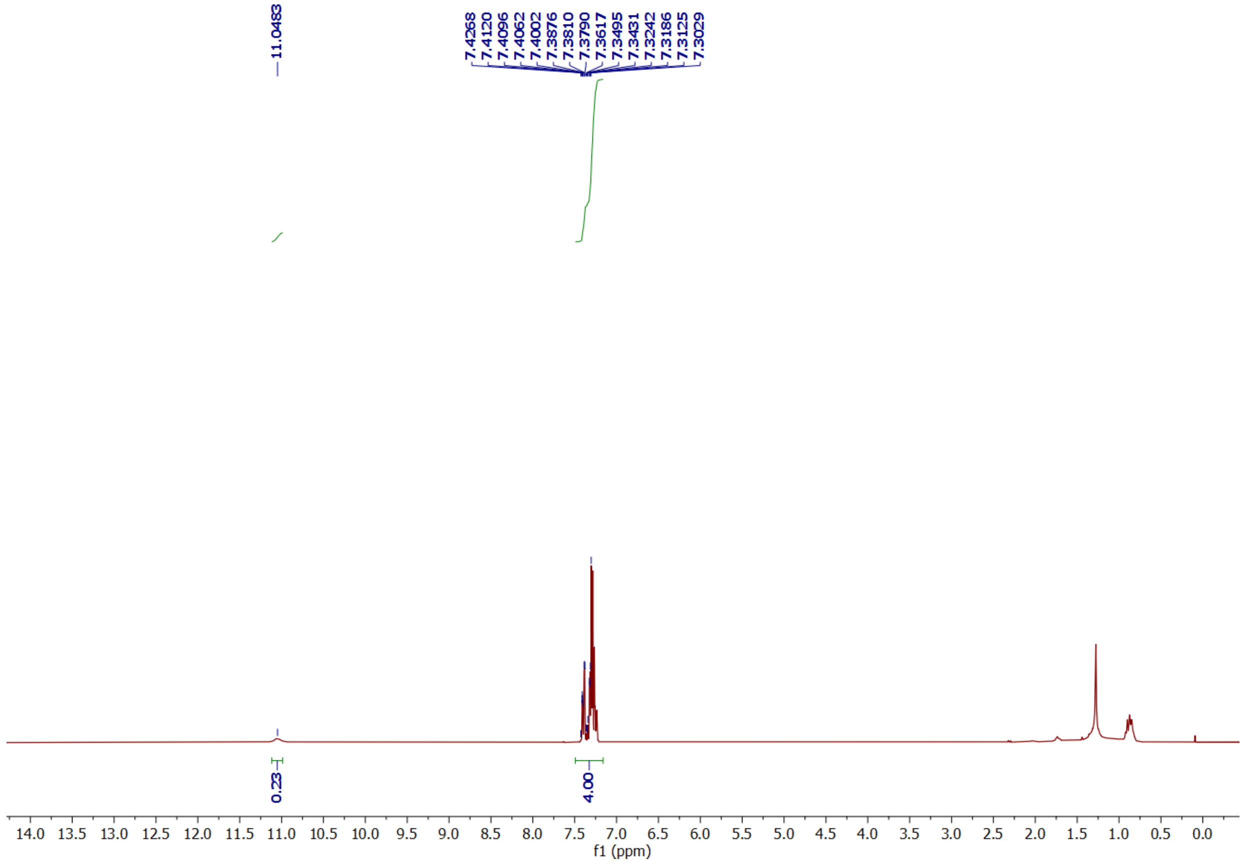

^{1}H NMR (400 MHz, $CDCl_3$) of **3aa** (see procedure)

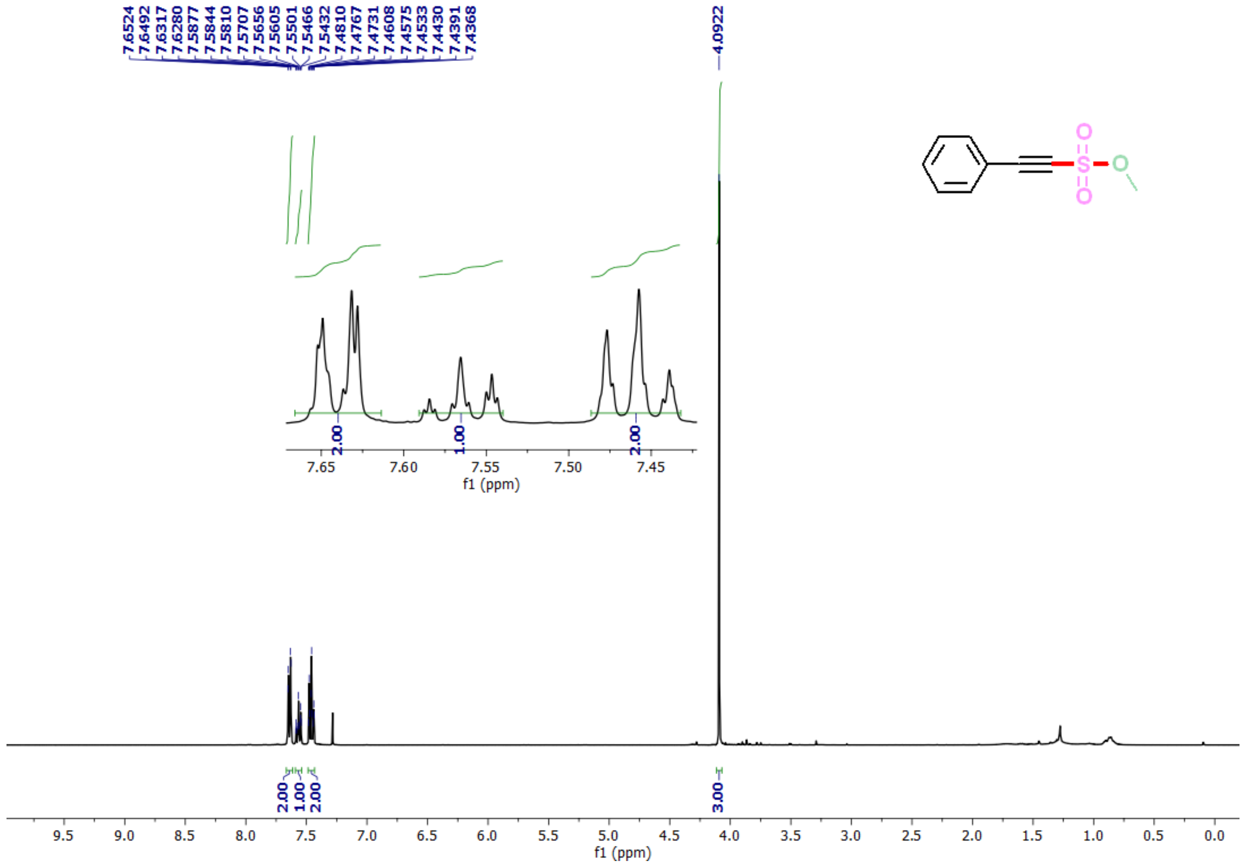

^{13}C NMR (101MHz, $CDCl_3$) of **3aa**

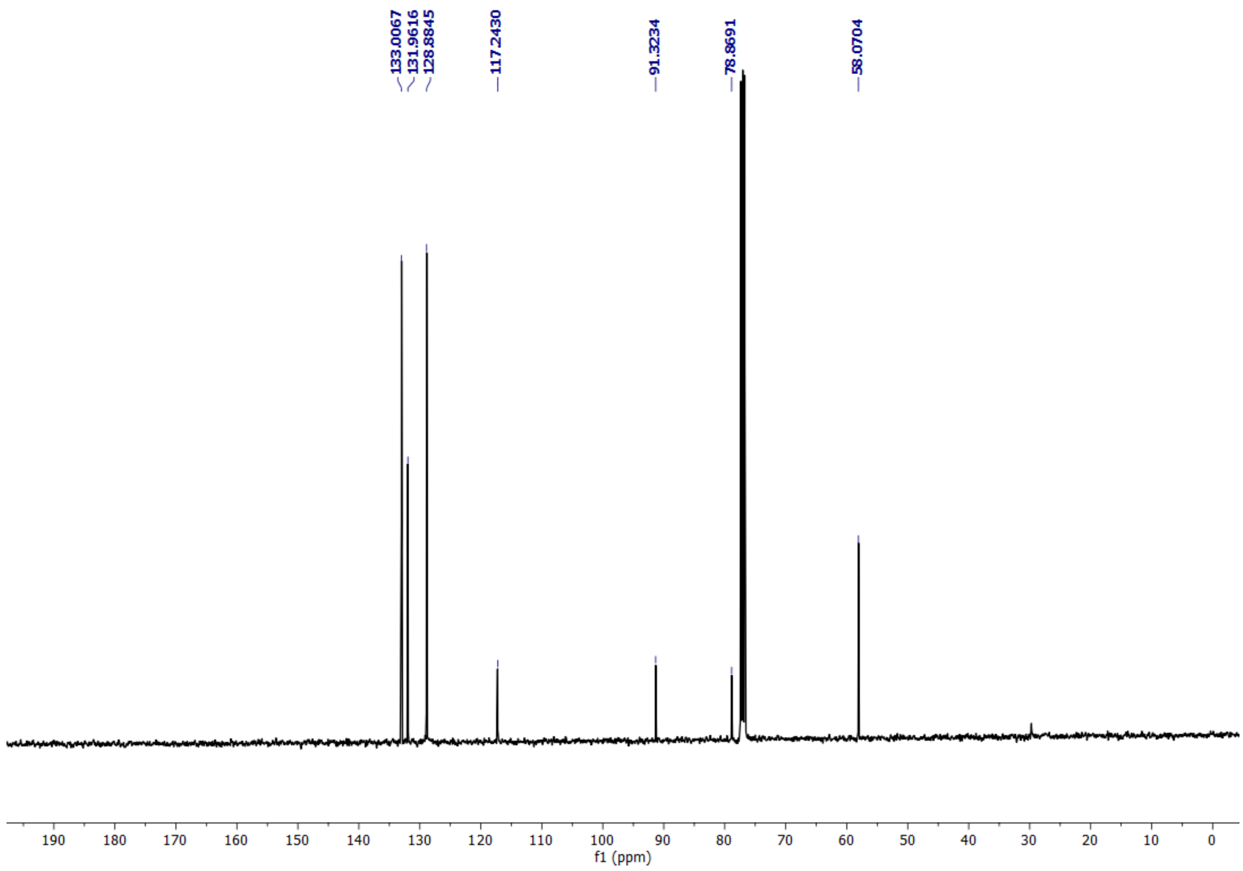

^{1}H NMR (400 MHz, $CDCl_3$) of **3ab** (see procedure)

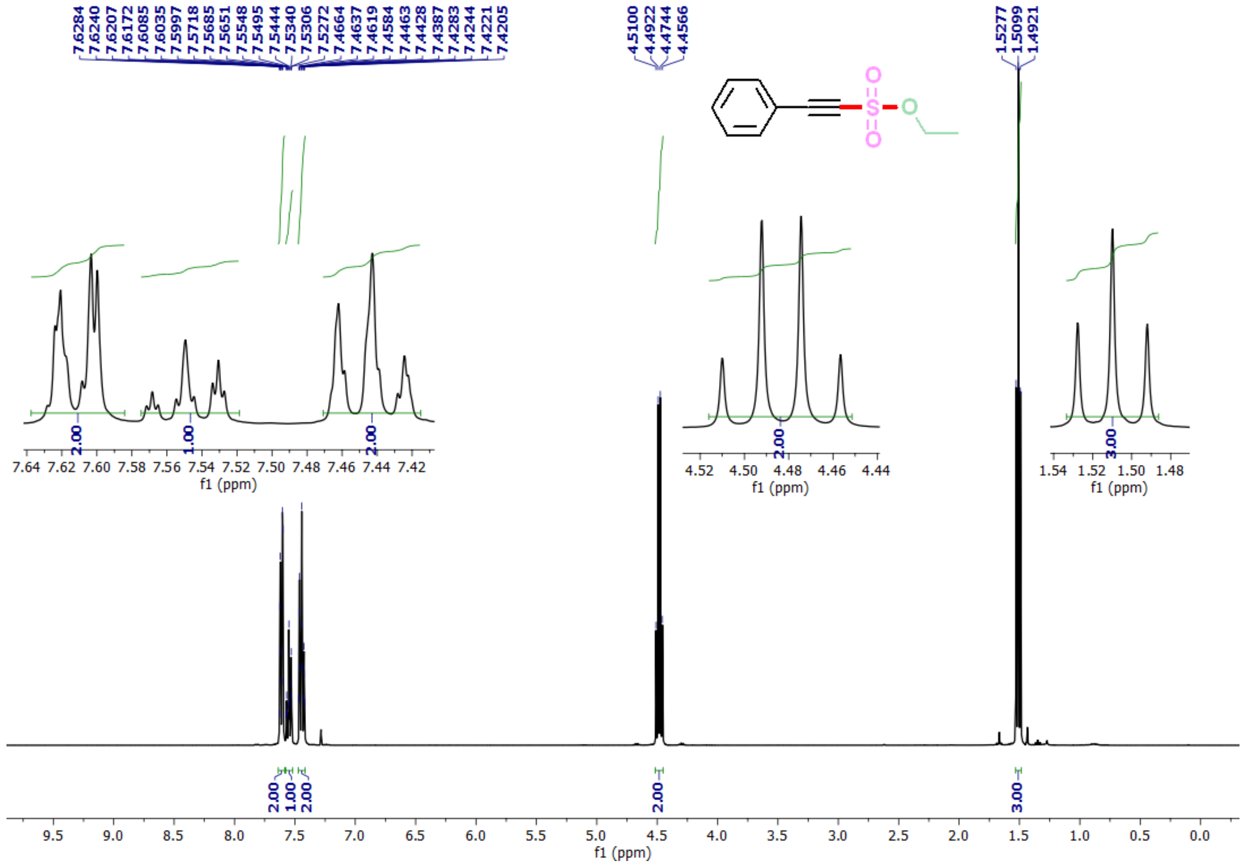

^{13}C NMR (101MHz, $CDCl_3$) of **3ab**

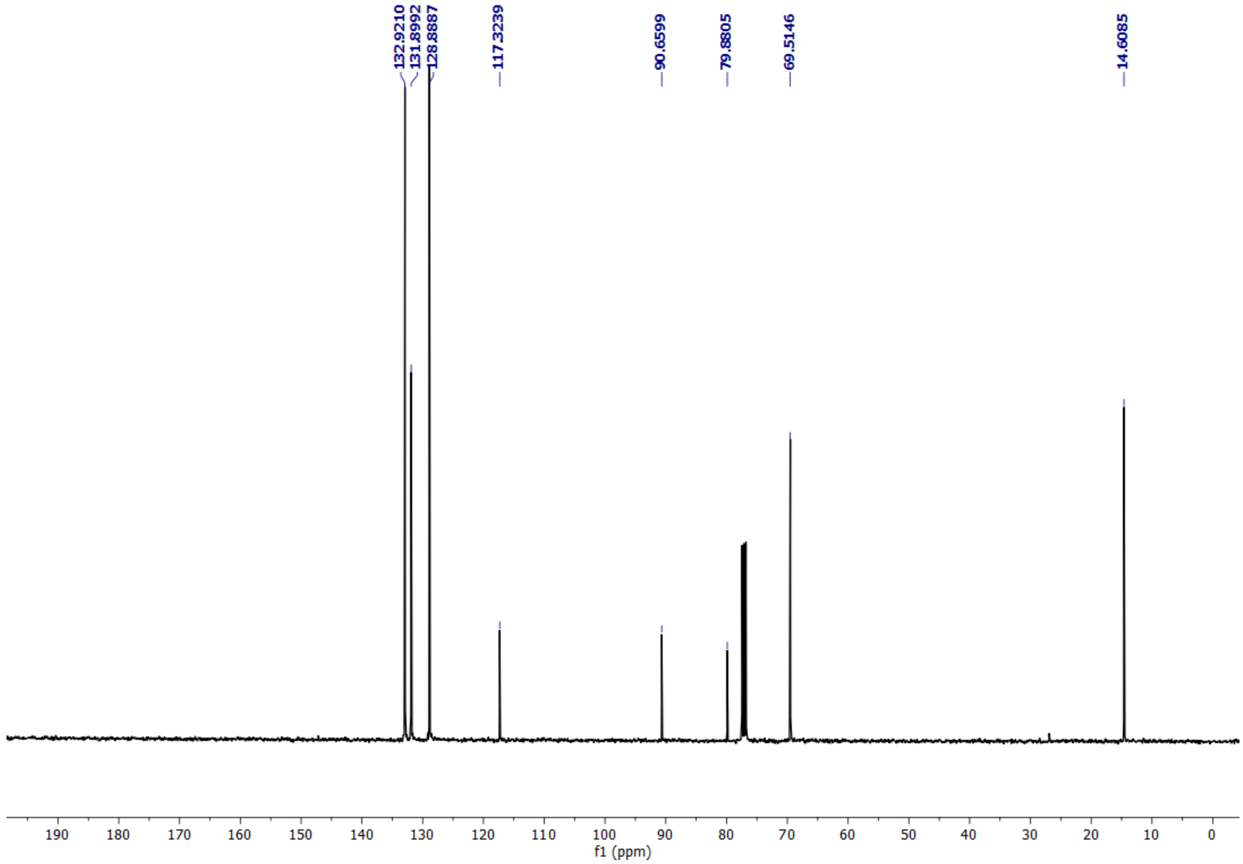

^{1}H NMR (400 MHz, $CDCl_3$) of **3ac** (see procedure)

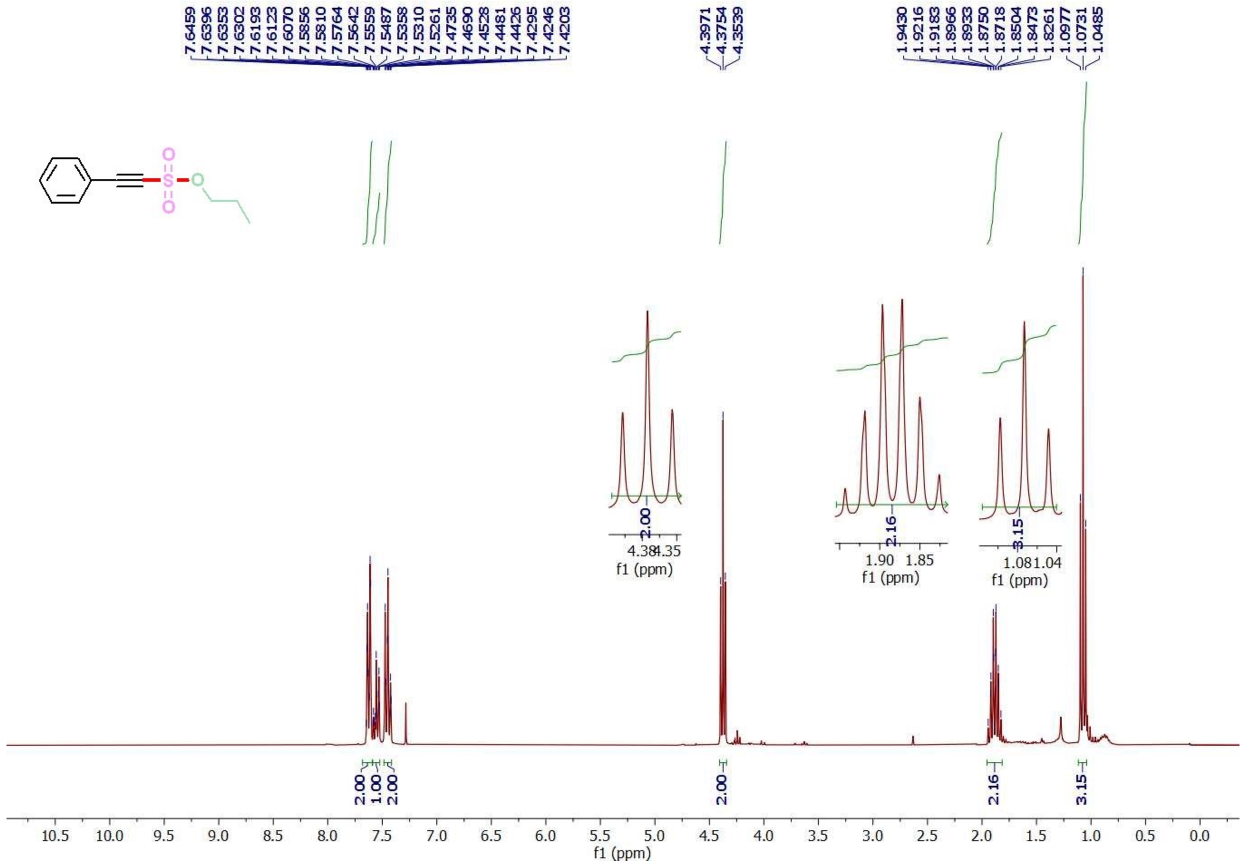

^{13}C NMR (101MHz, $CDCl_3$) of **3ac**

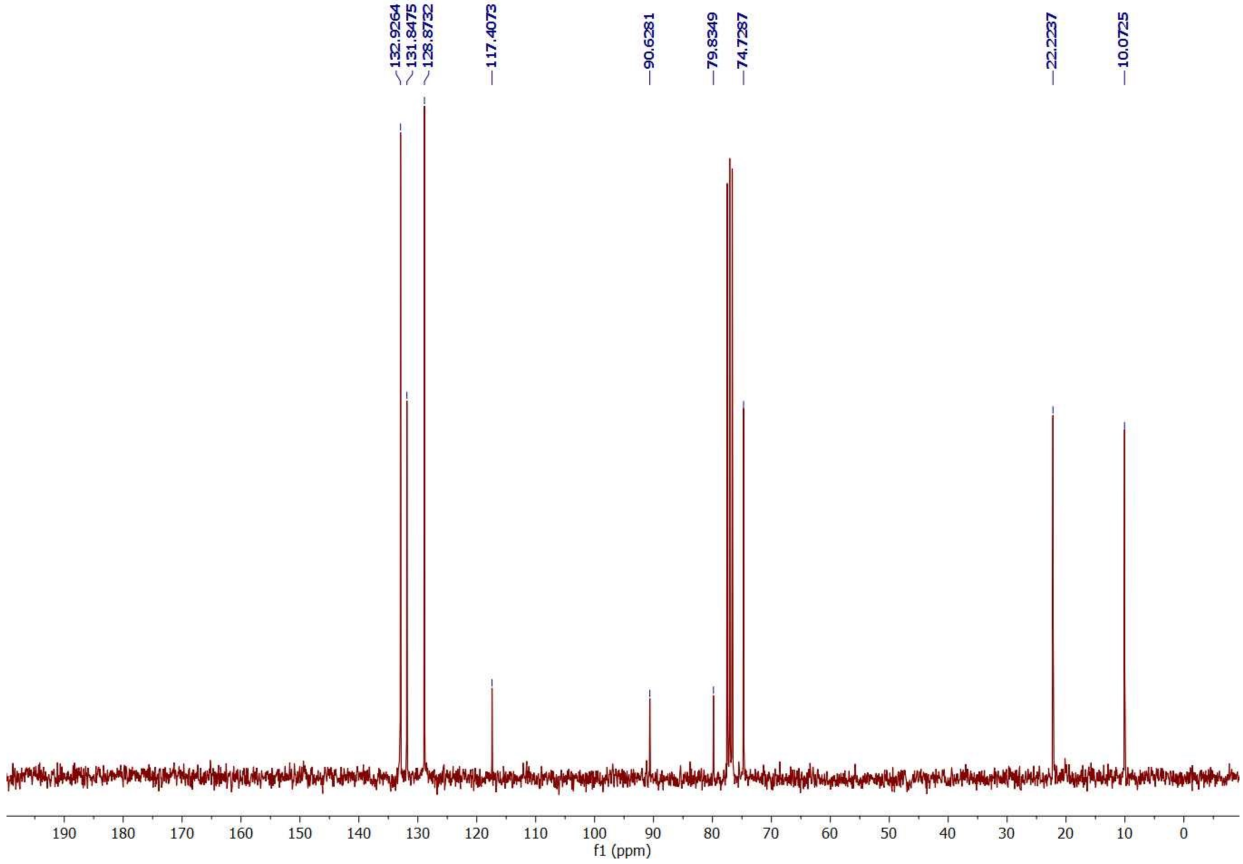

^{1}H NMR (400 MHz, $CDCl_3$) of **3ad** (see procedure)

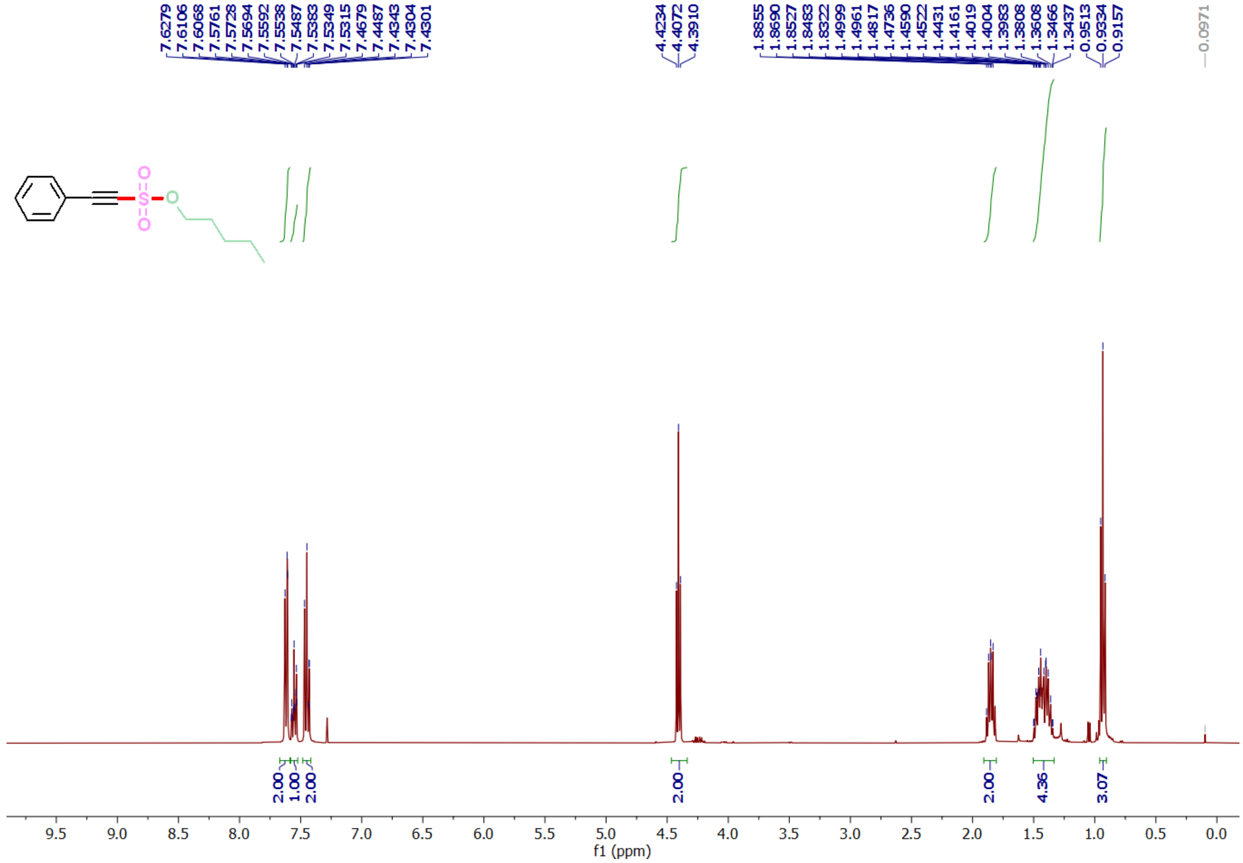

^{13}C NMR (101MHz, $CDCl_3$) of **3ad**

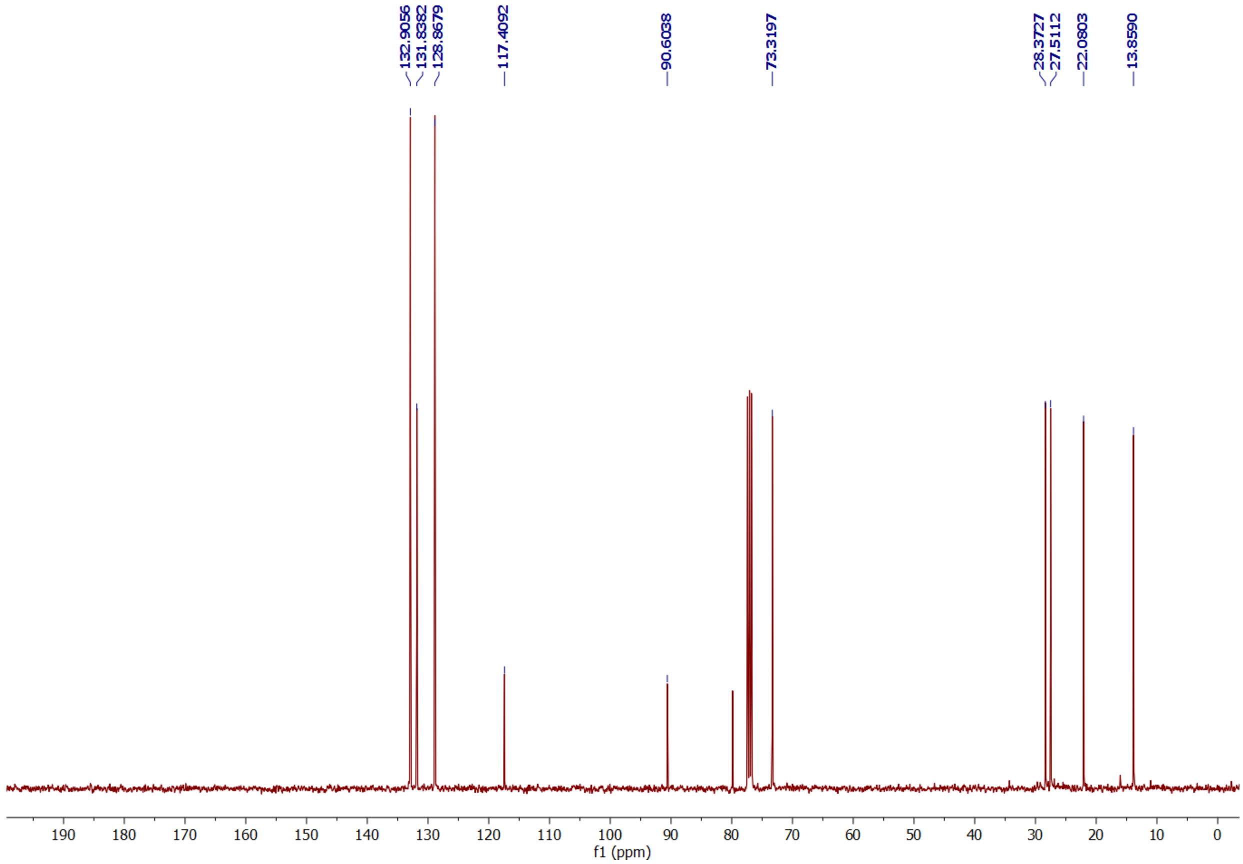

^{1}H NMR (400 MHz, $CDCl_3$) of **3ae** (see procedure)

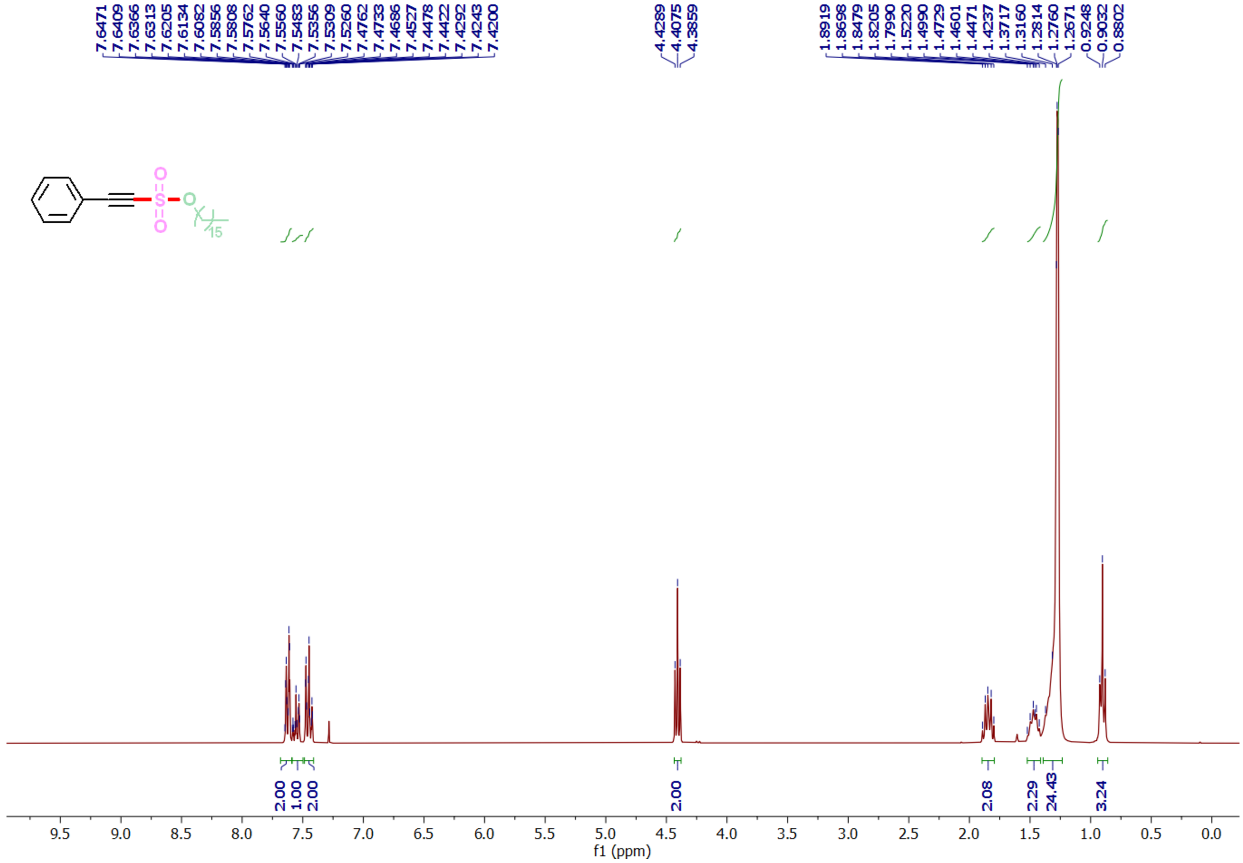

^{13}C NMR (101MHz, $CDCl_3$) of **3ae**

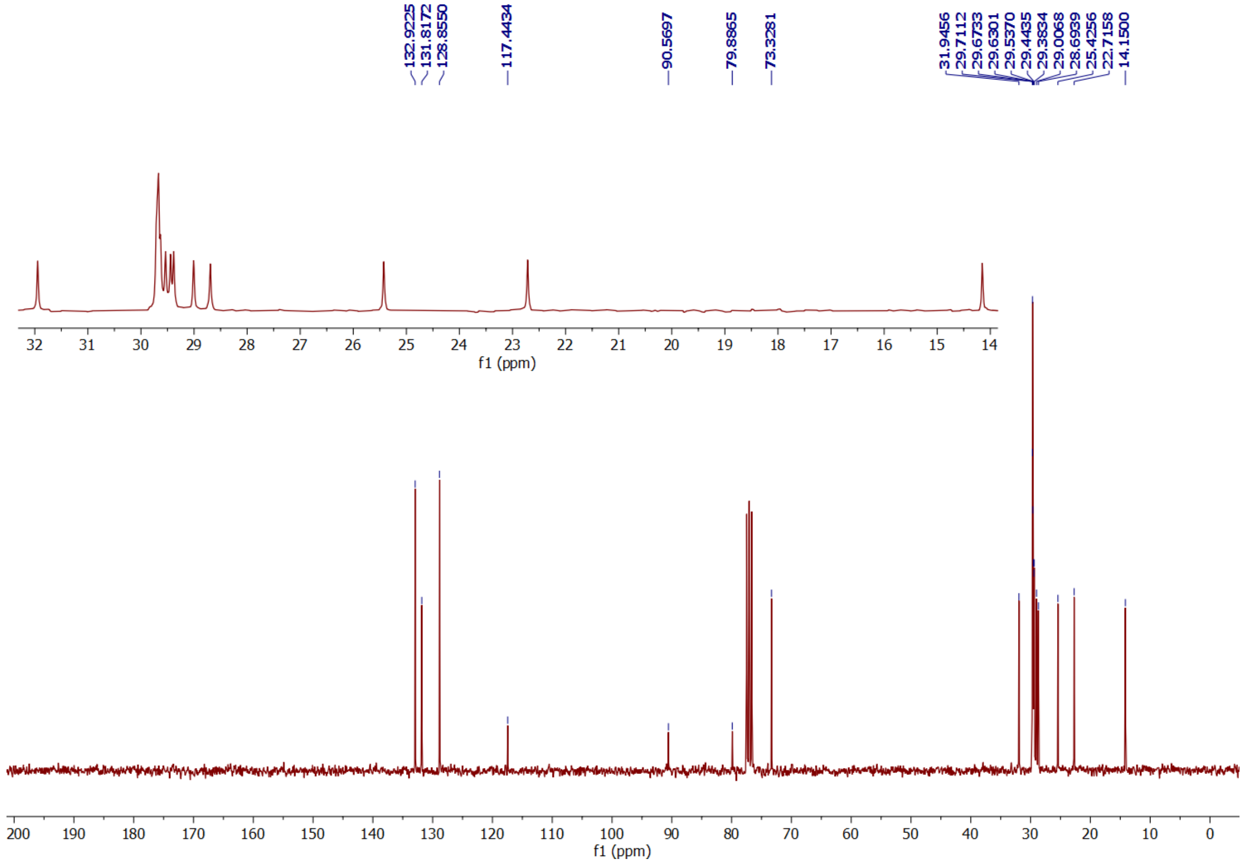

^{1}H NMR (400 MHz, $CDCl_3$) of **3af** (see procedure)

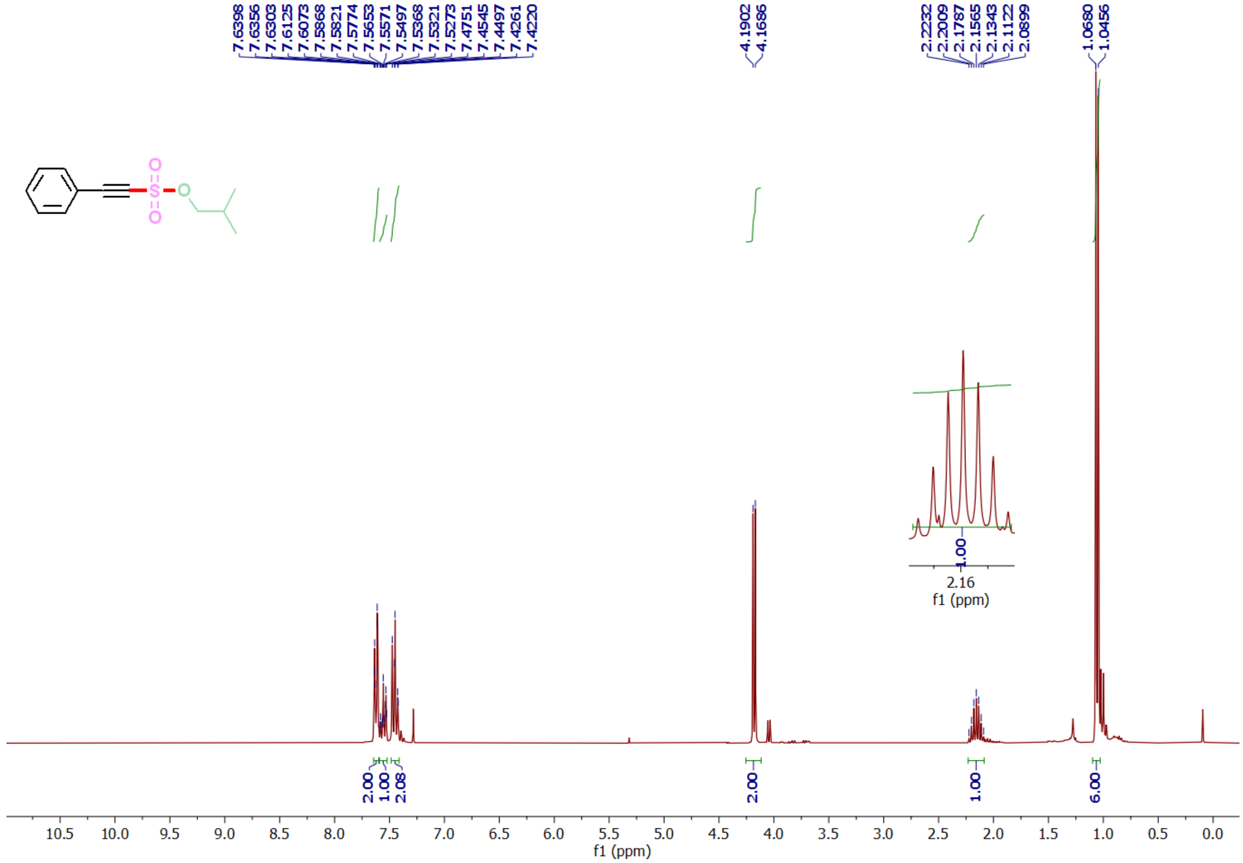

^{13}C NMR (101MHz, $CDCl_3$) of **3af**

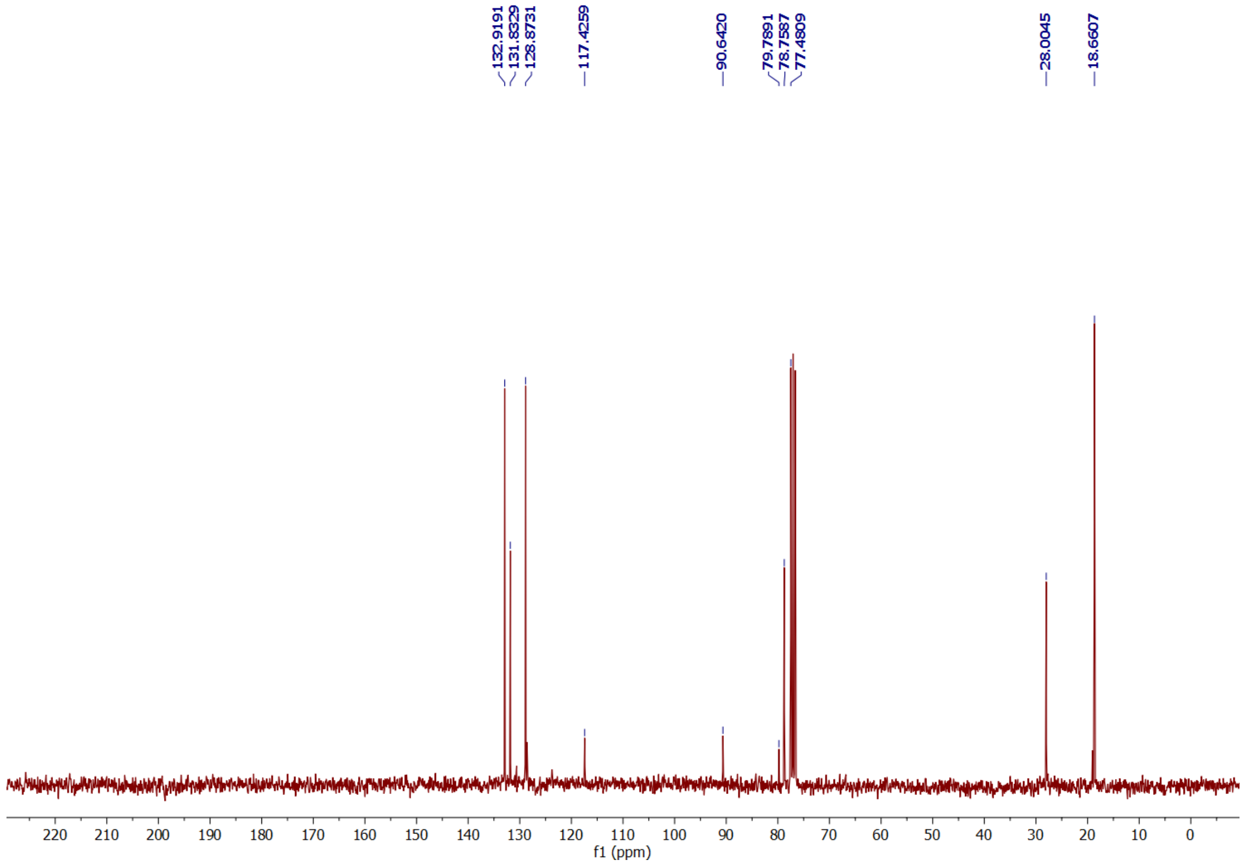

^{1}H NMR (400 MHz, $CDCl_3$) of **3ag** (see procedure)

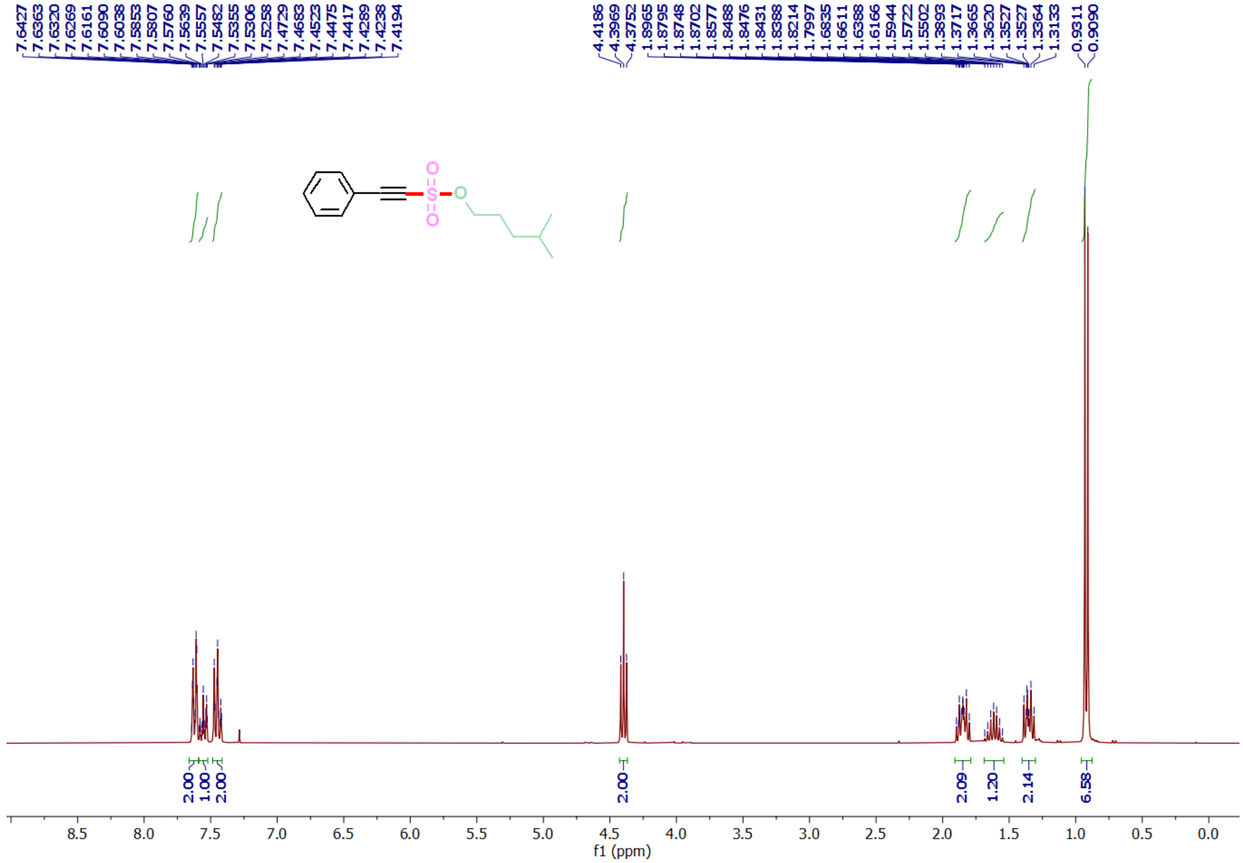

^{13}C NMR (101MHz, $CDCl_3$) of **3ag**

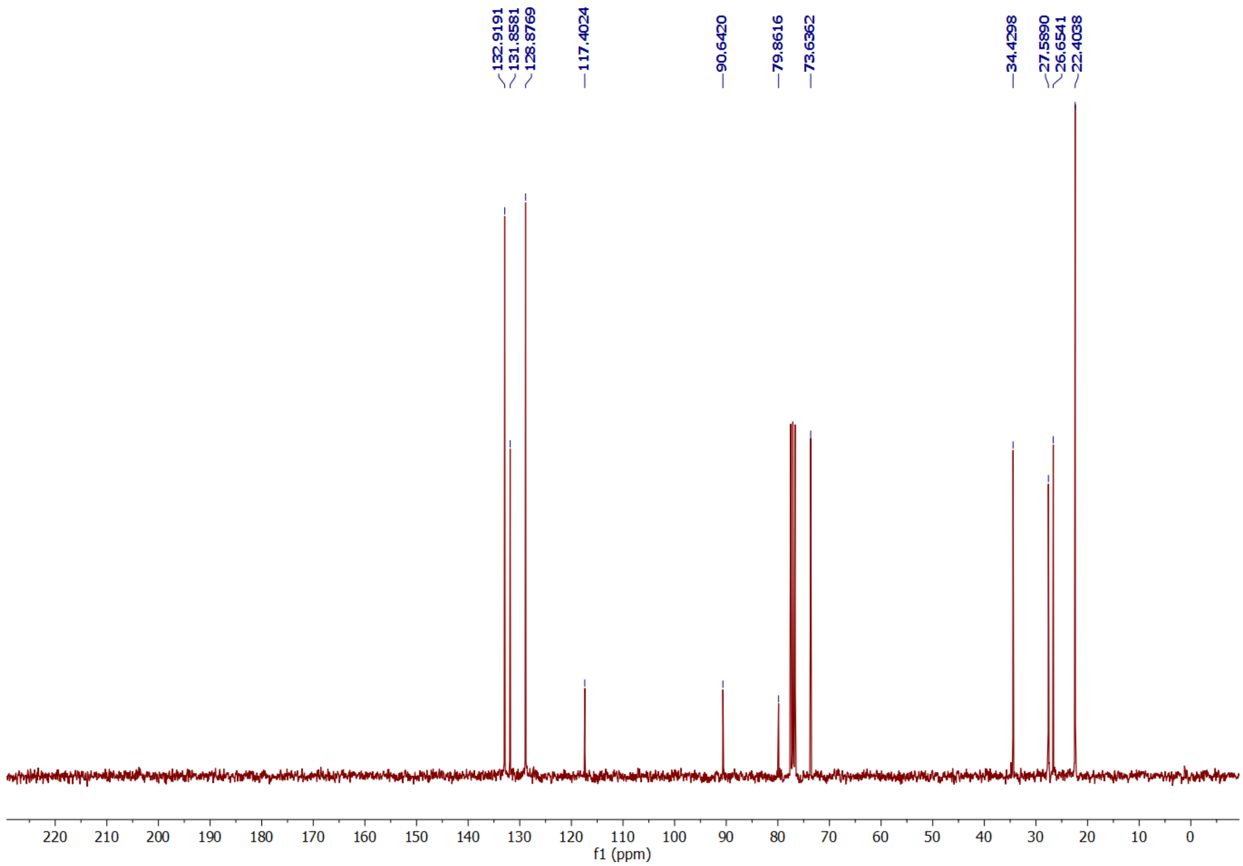

^{1}H NMR (400 MHz, $CDCl_3$) of **3ah** (see procedure)

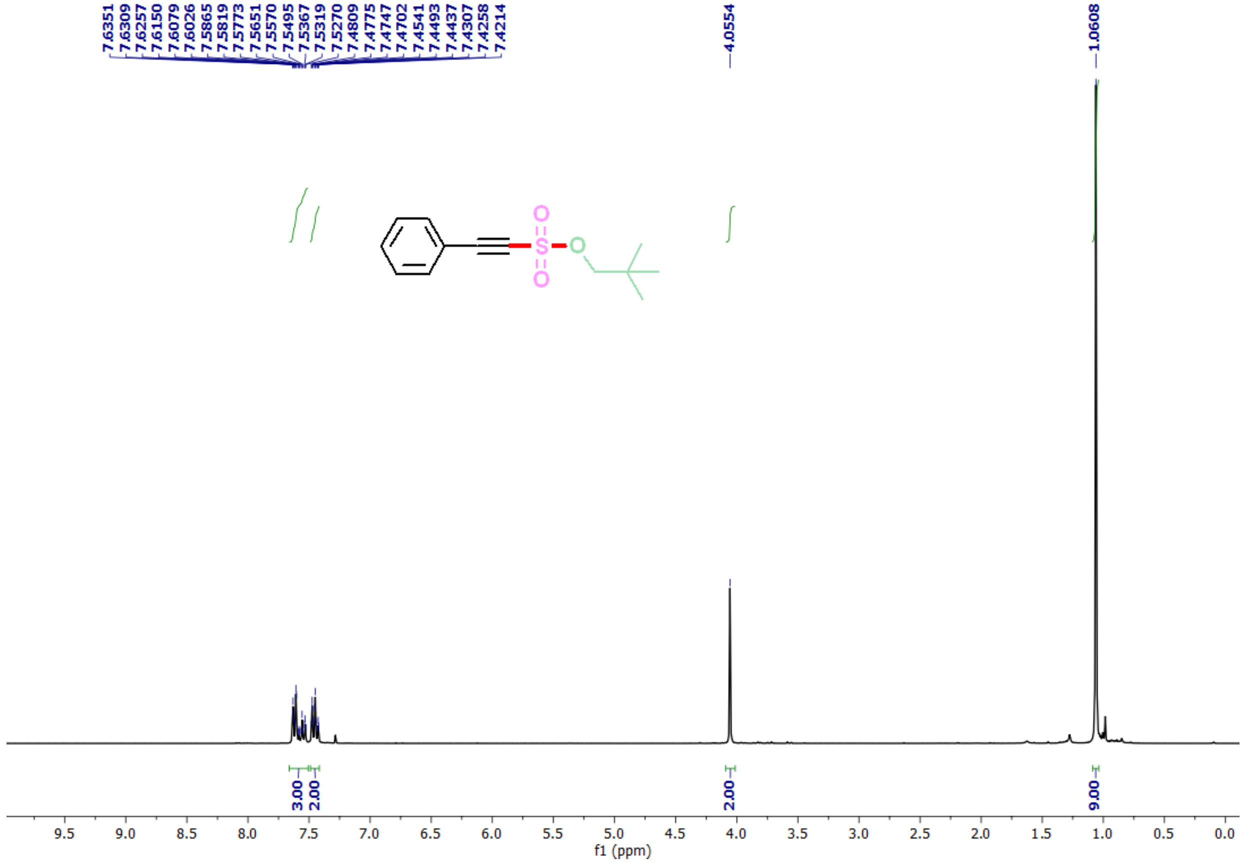

^{13}C NMR (101MHz, $CDCl_3$) of **3ah**

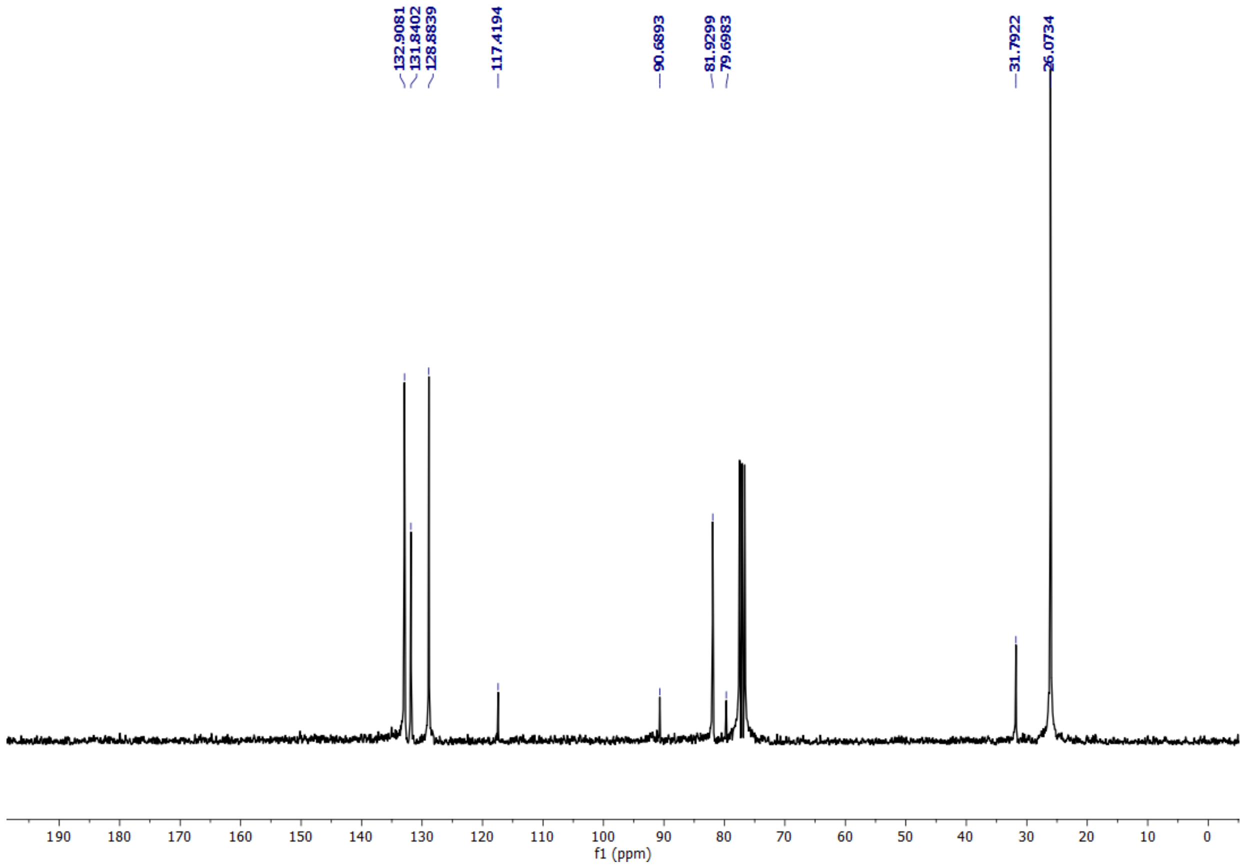

^{1}H NMR (400 MHz, $CDCl_3$) of **3ai** (see procedure)

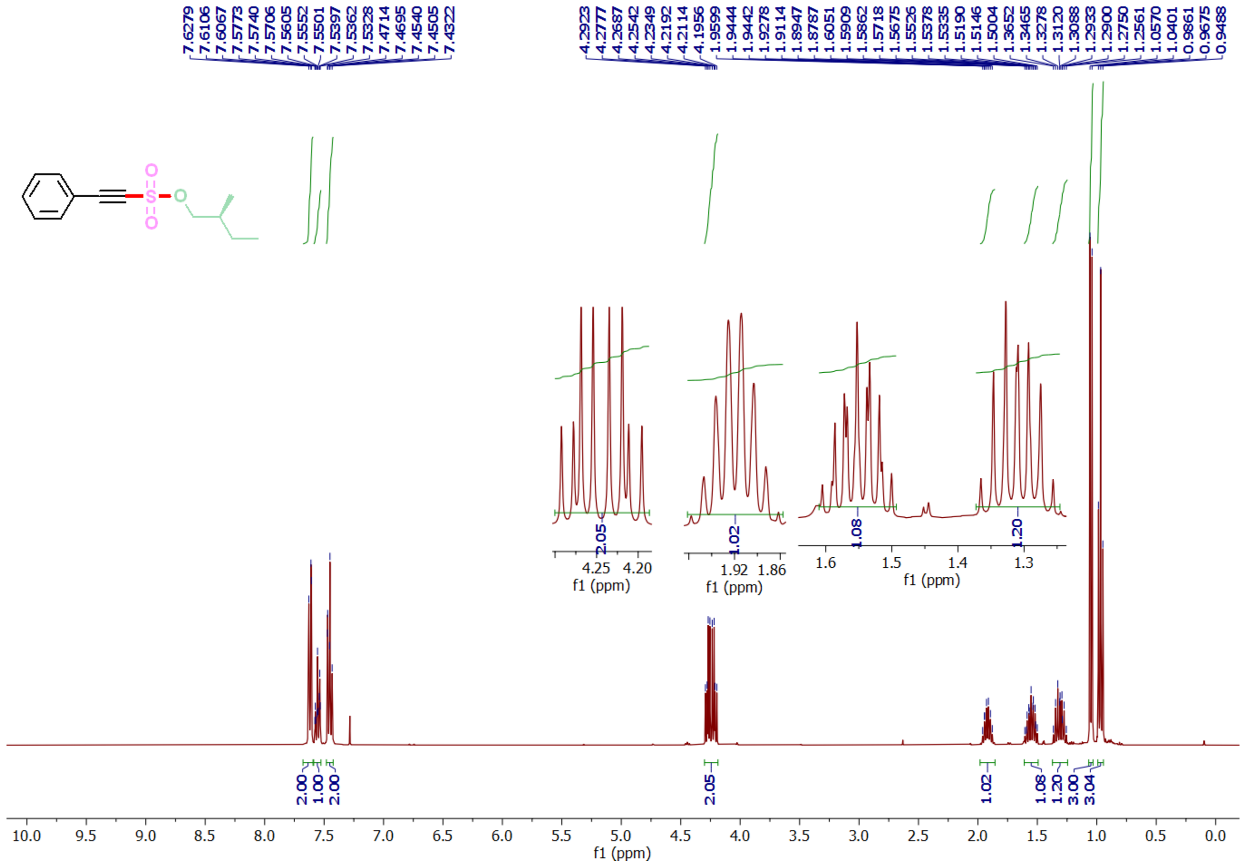

^{13}C NMR (101MHz, $CDCl_3$) of **3ai**

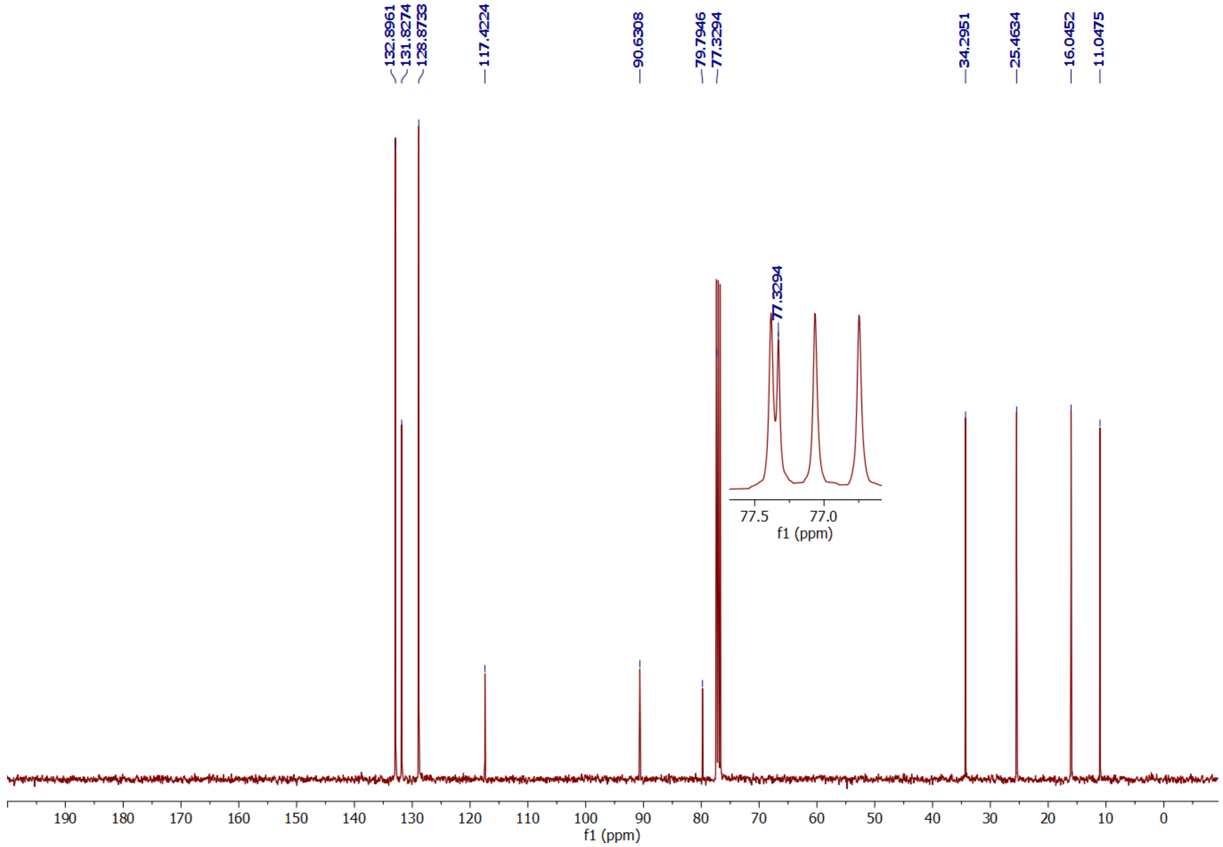

^{1}H NMR (400 MHz, $CDCl_3$) of **3aj** (see procedure)

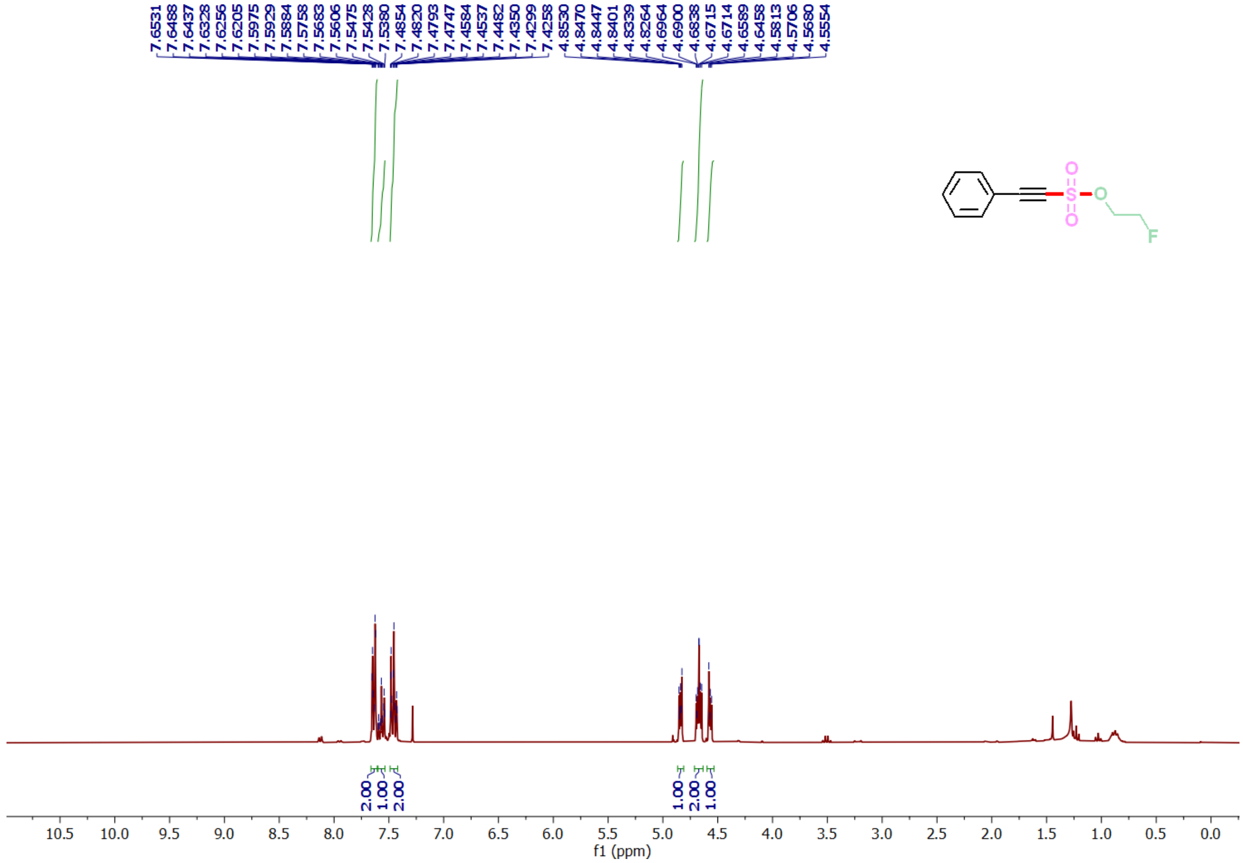

^{13}C NMR (101MHz, $CDCl_3$) of **3aj**

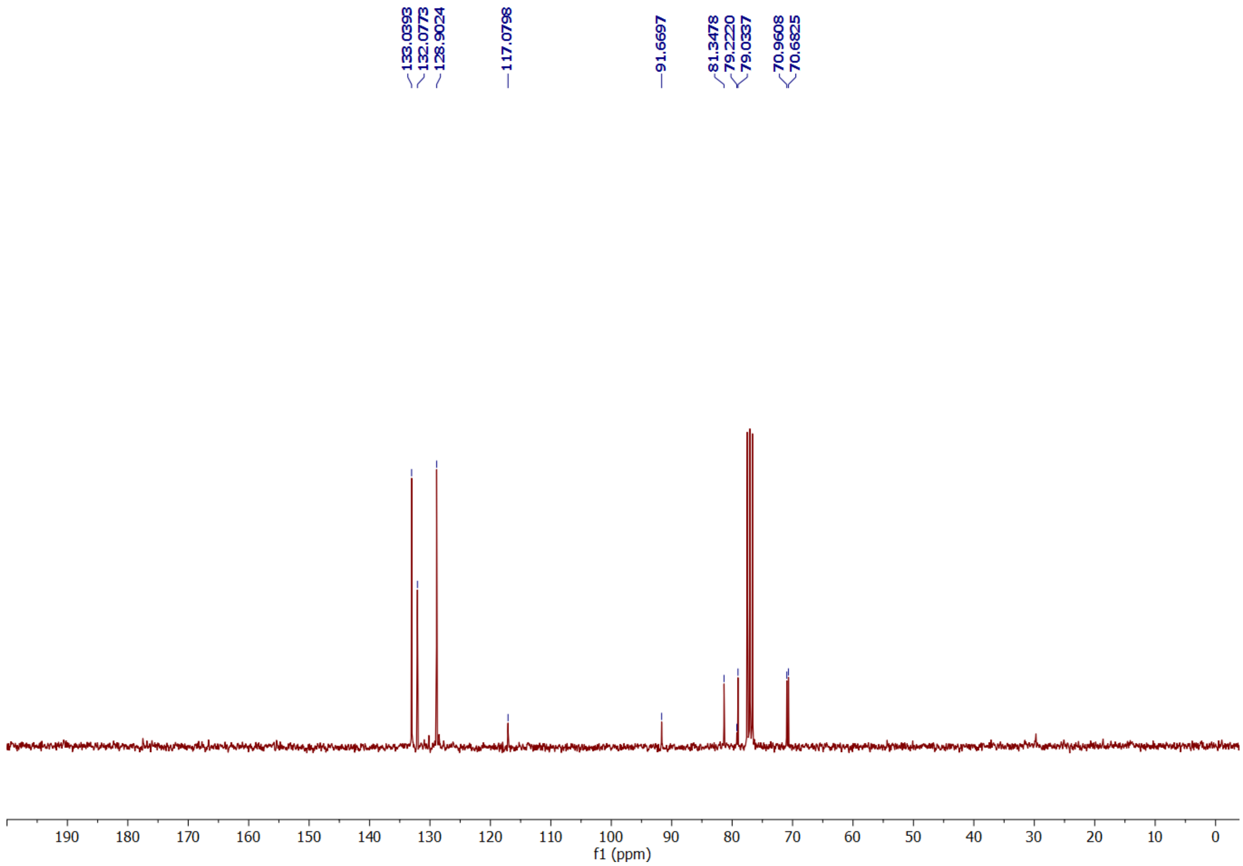

^{1}H NMR (400 MHz, $CDCl_3$) of **3ak** (see procedure)

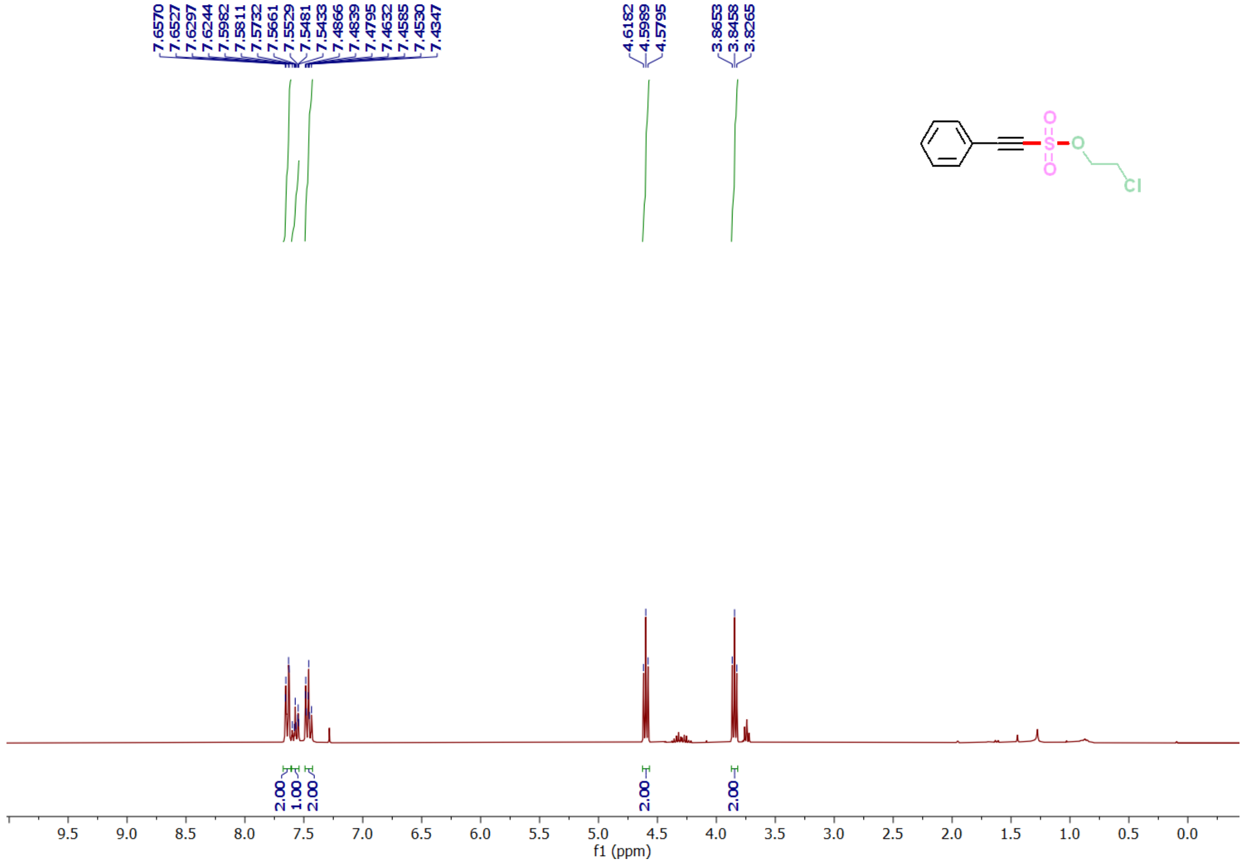

^{13}C NMR (101MHz, $CDCl_3$) of **3ak**

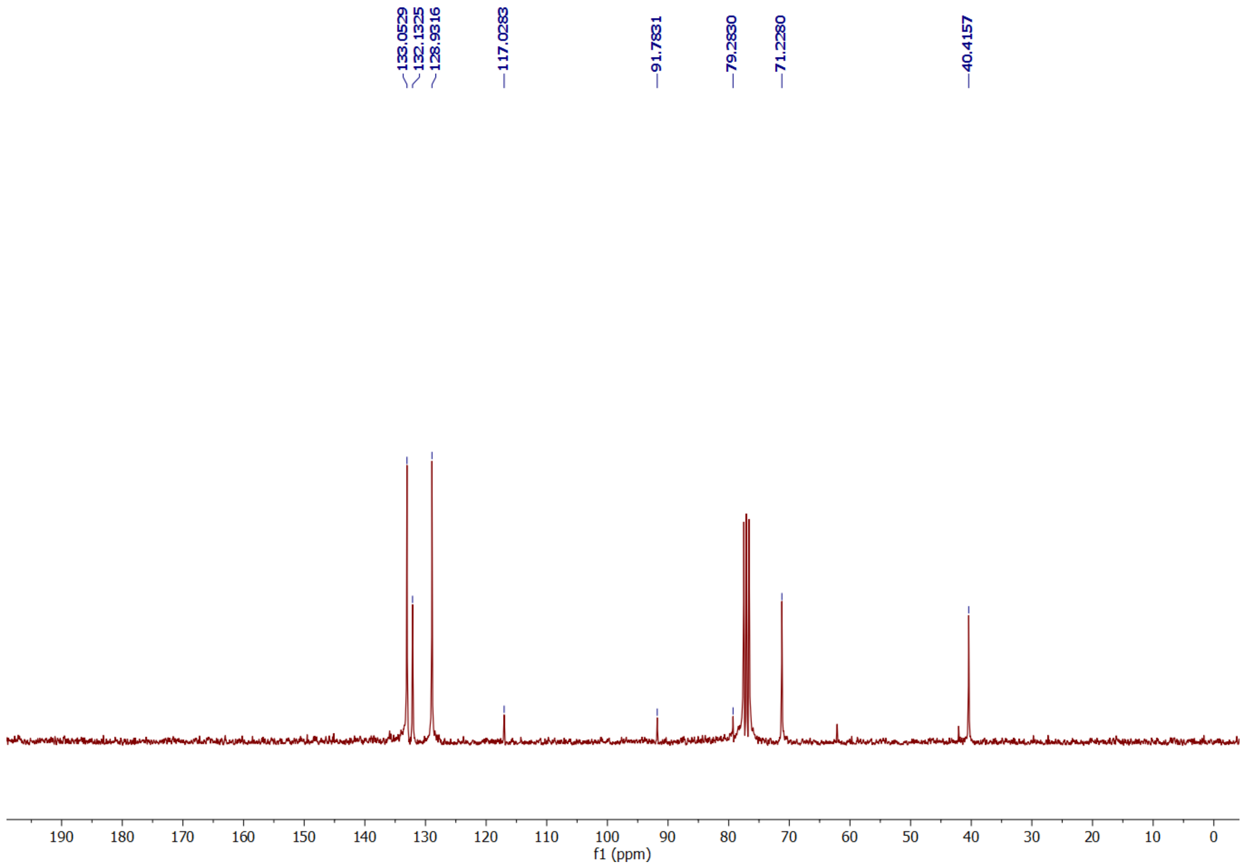

^{1}H NMR (400 MHz, $CDCl_3$) of **3al** (see procedure)

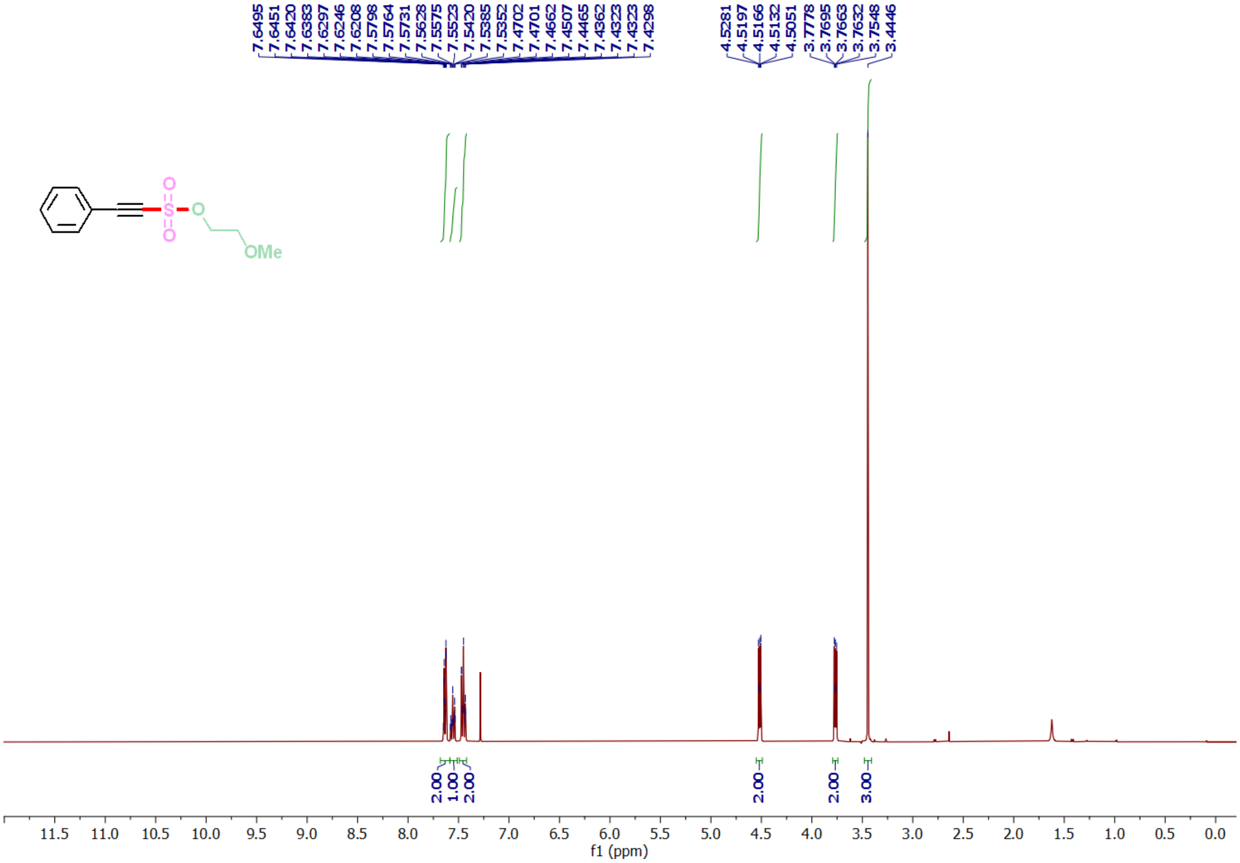

^{13}C NMR (101MHz, $CDCl_3$) of **3al**

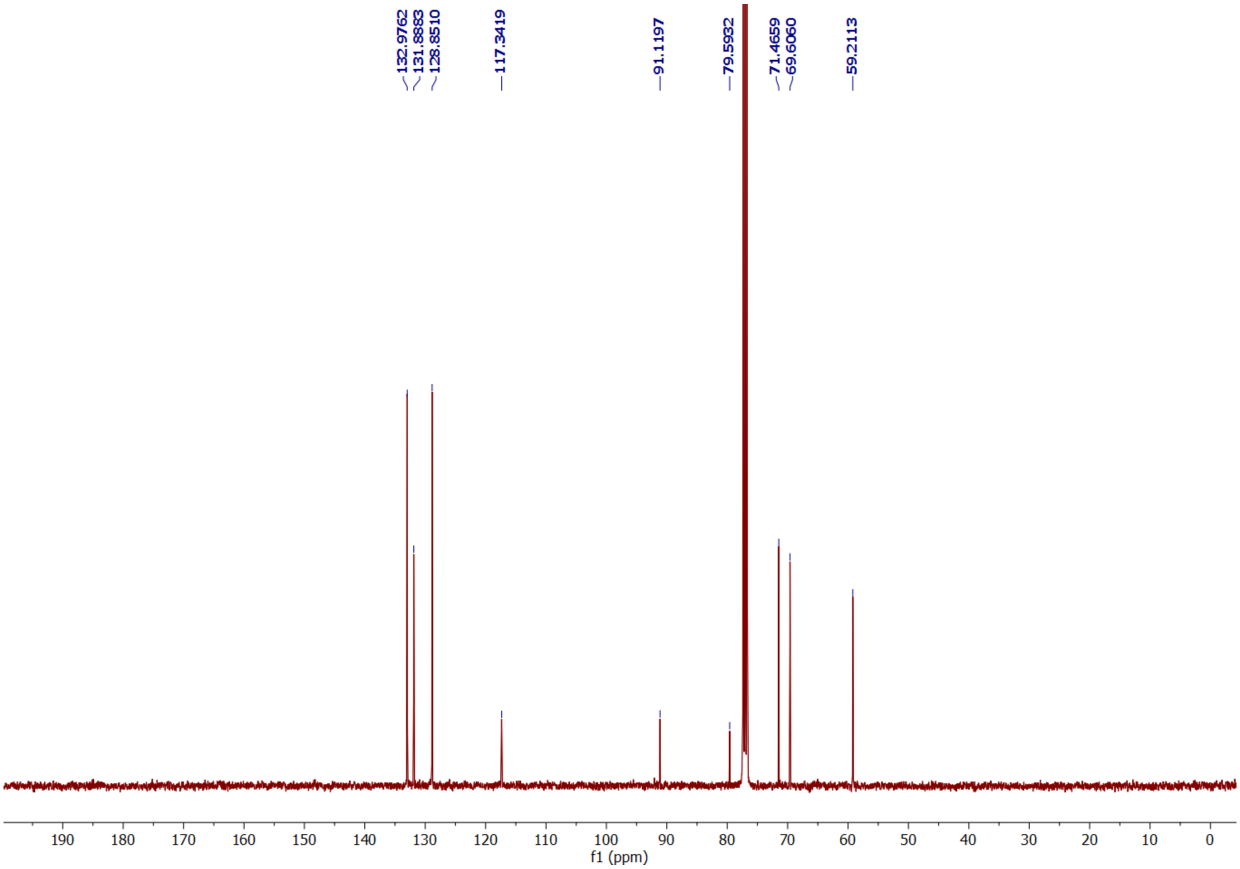

^{1}H NMR (400 MHz, $CDCl_3$) of **3am** (see procedure)

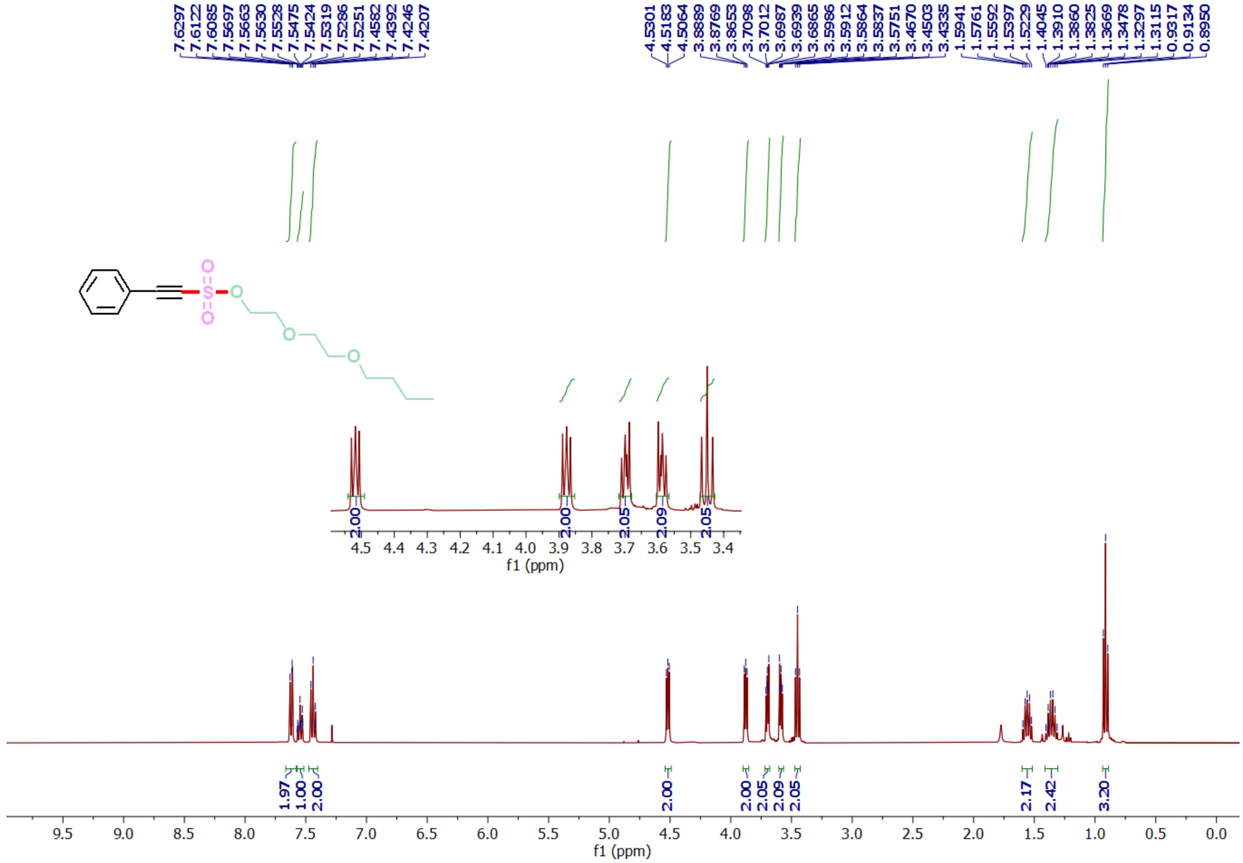

^{13}C NMR (101MHz, $CDCl_3$) of **3am**

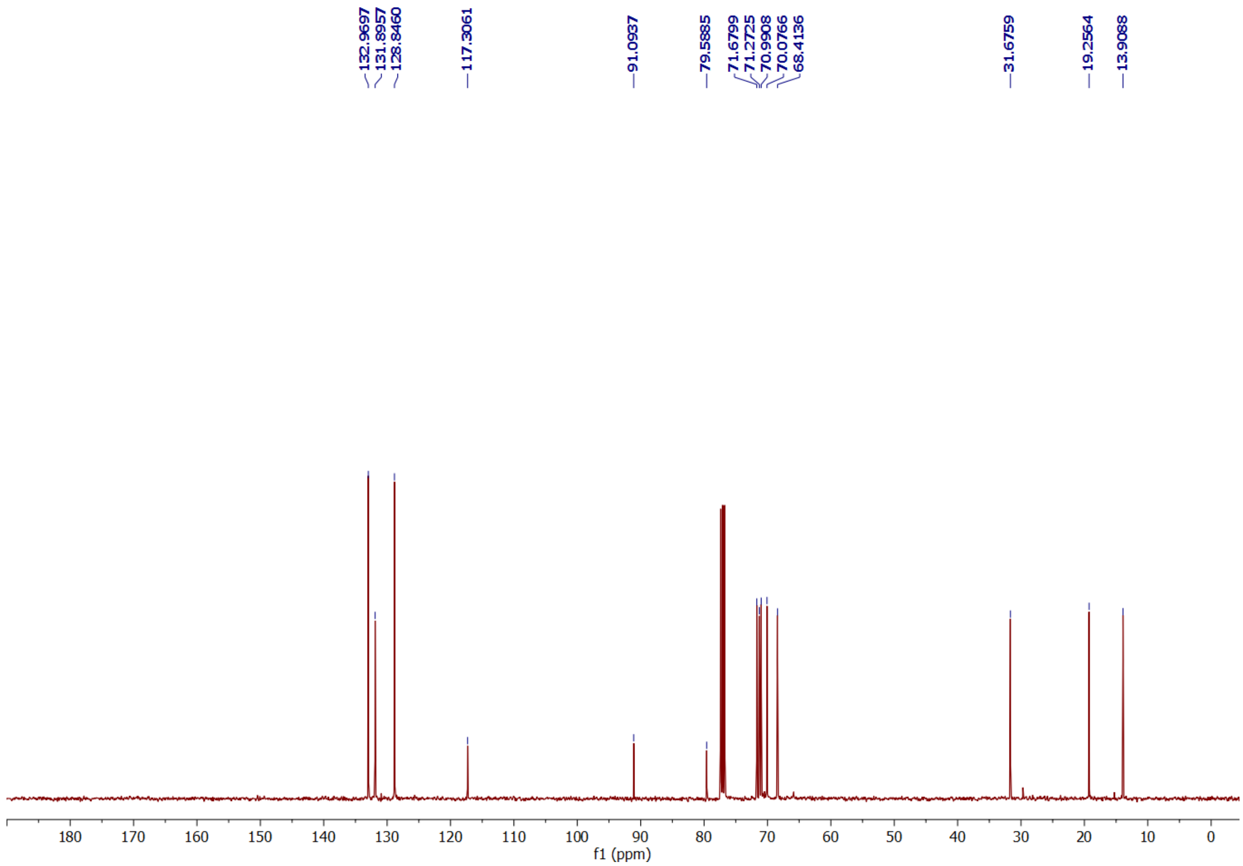

^{1}H NMR (400 MHz, $CDCl_3$) of **3an** (see procedure)

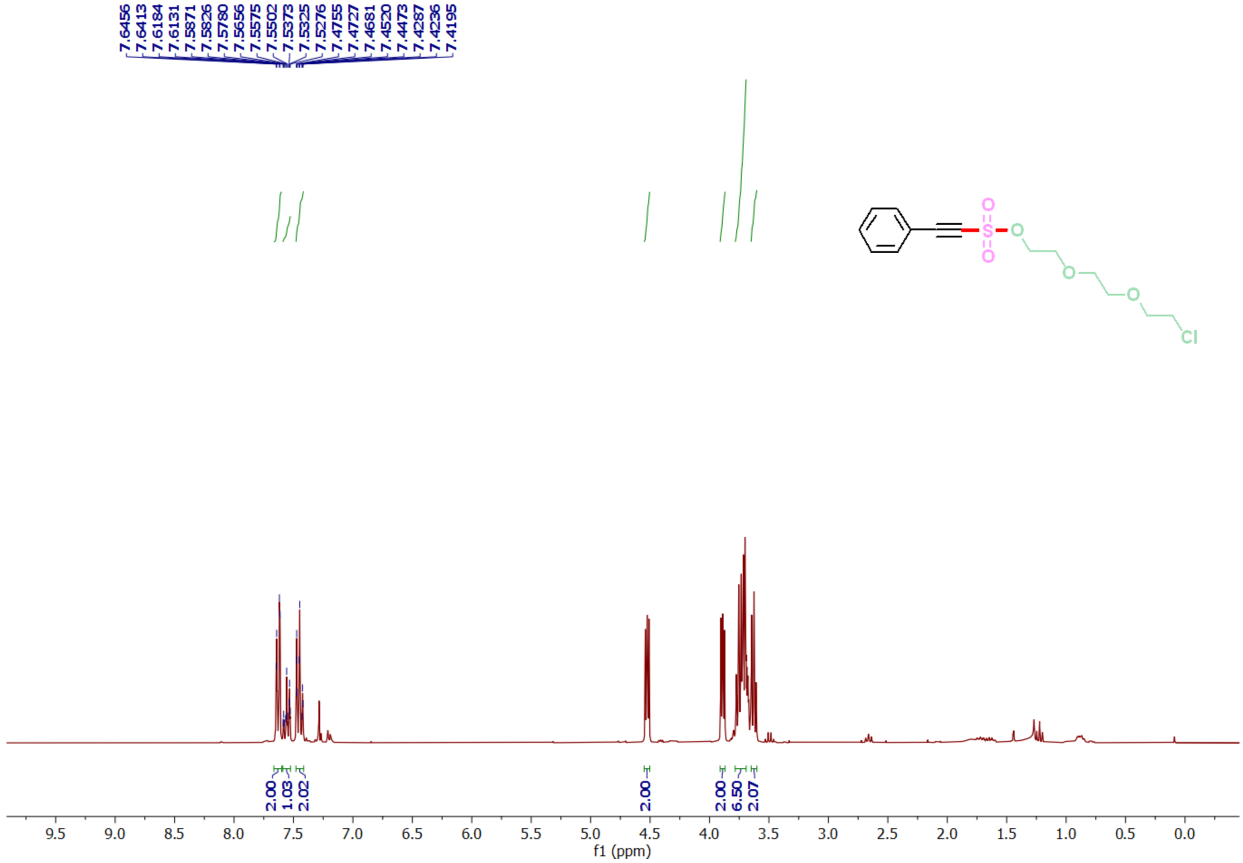

^{13}C NMR (101MHz, $CDCl_3$) of **3an**

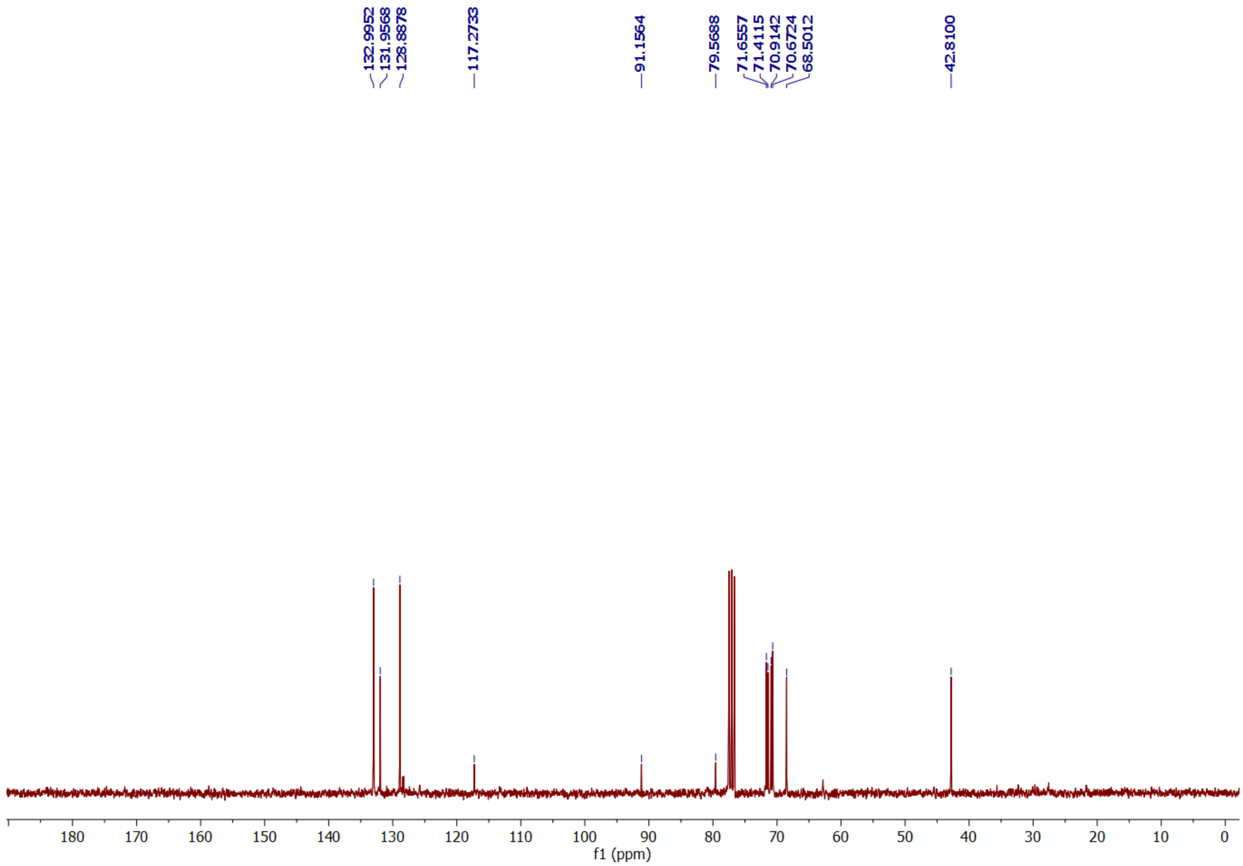

^{1}H NMR (400 MHz, $CDCl_3$) of **3ao** (see procedure)

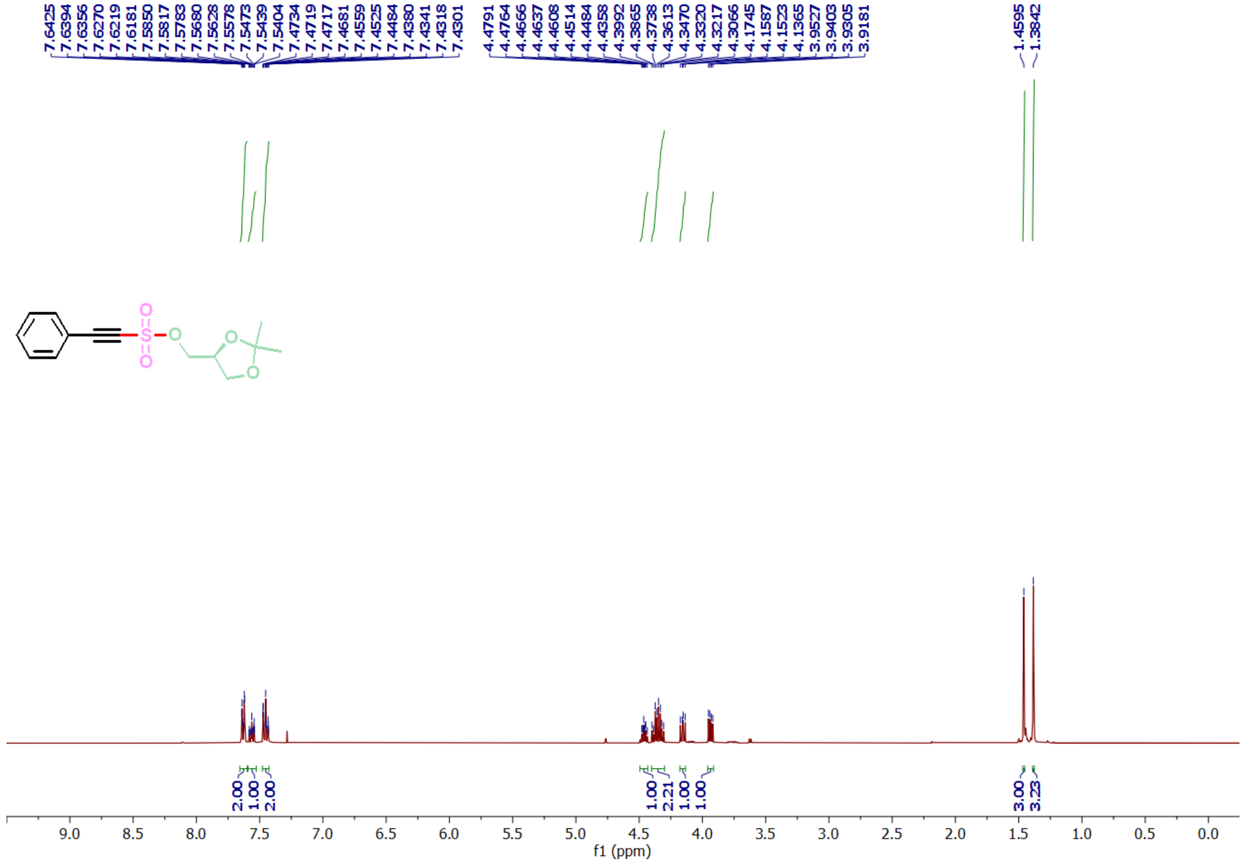

^{13}C NMR (101MHz, $CDCl_3$) of **3ao**

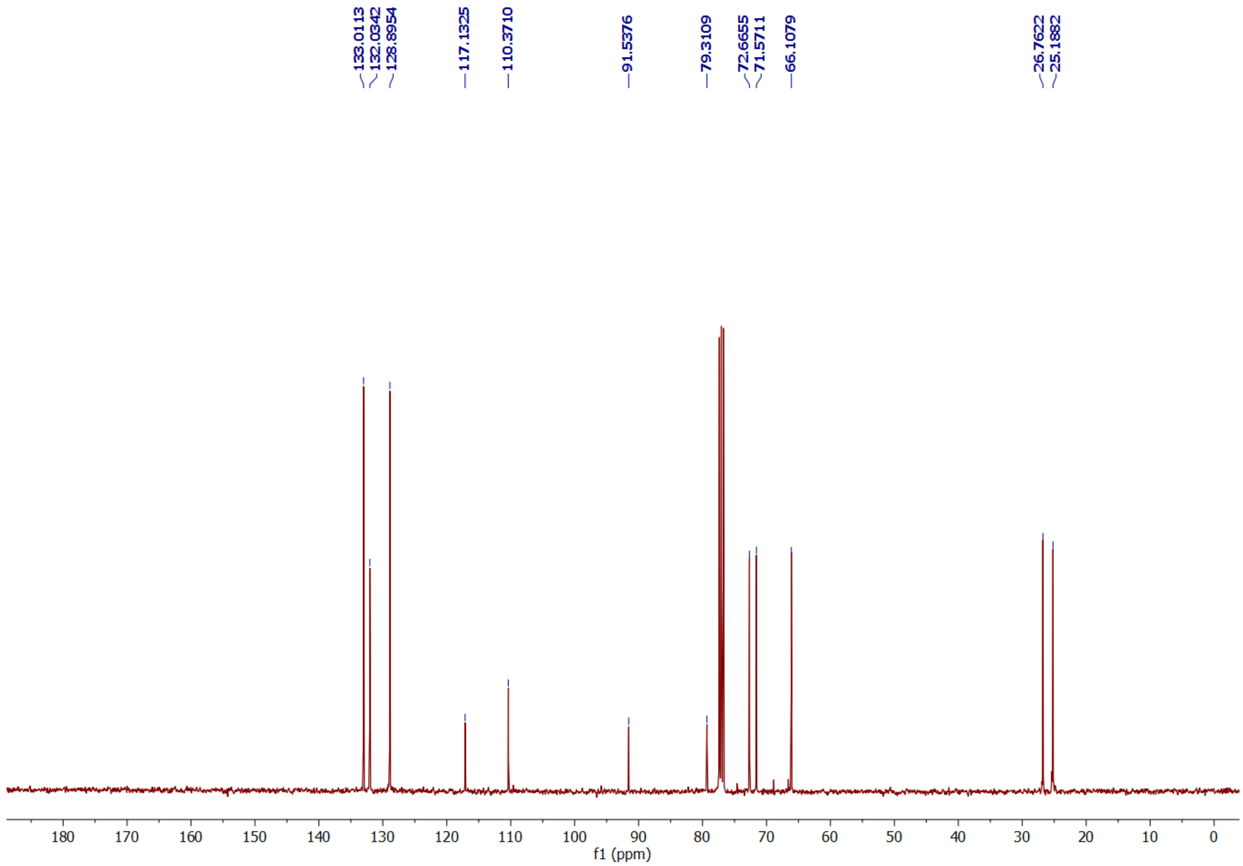

^{1}H NMR (400 MHz, $CDCl_3$) of **3ap** (see procedure)

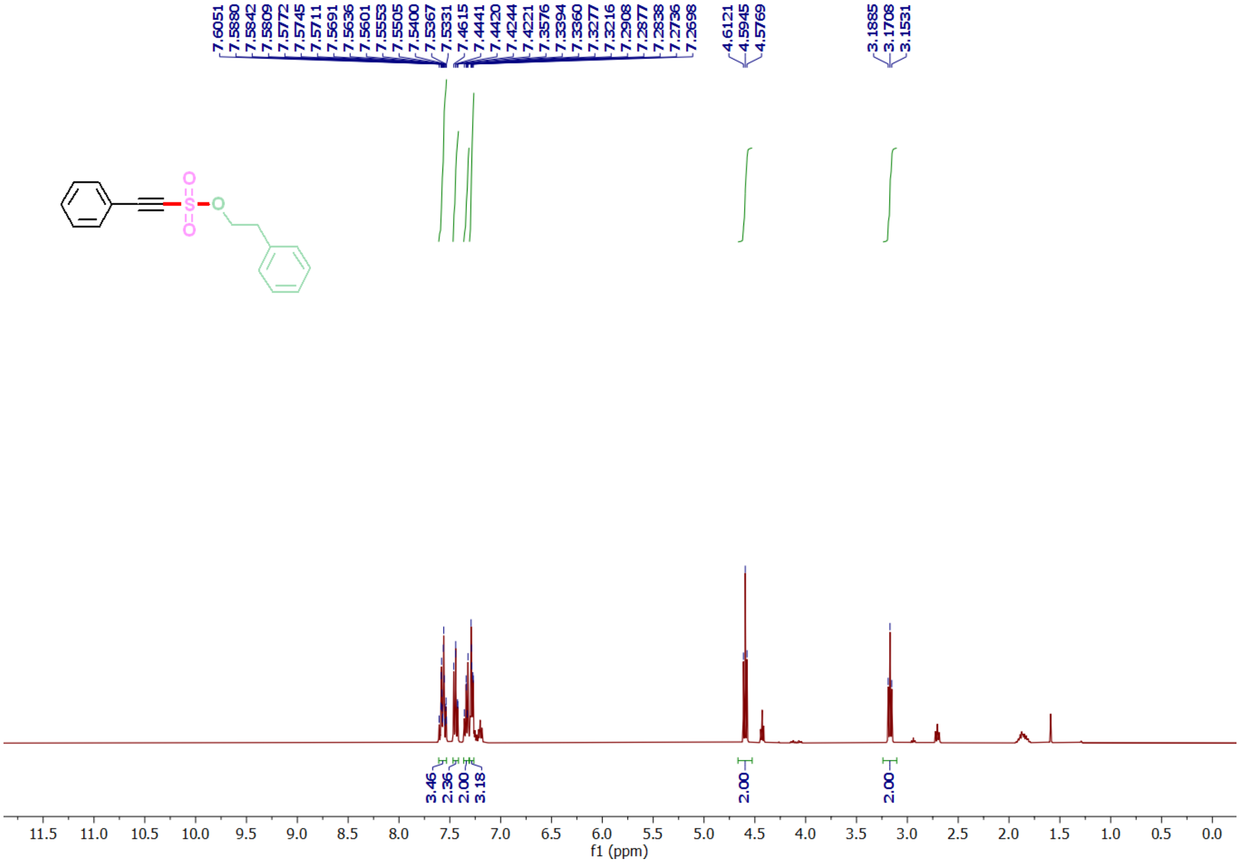

^{13}C NMR (101MHz, $CDCl_3$) of **3ap**

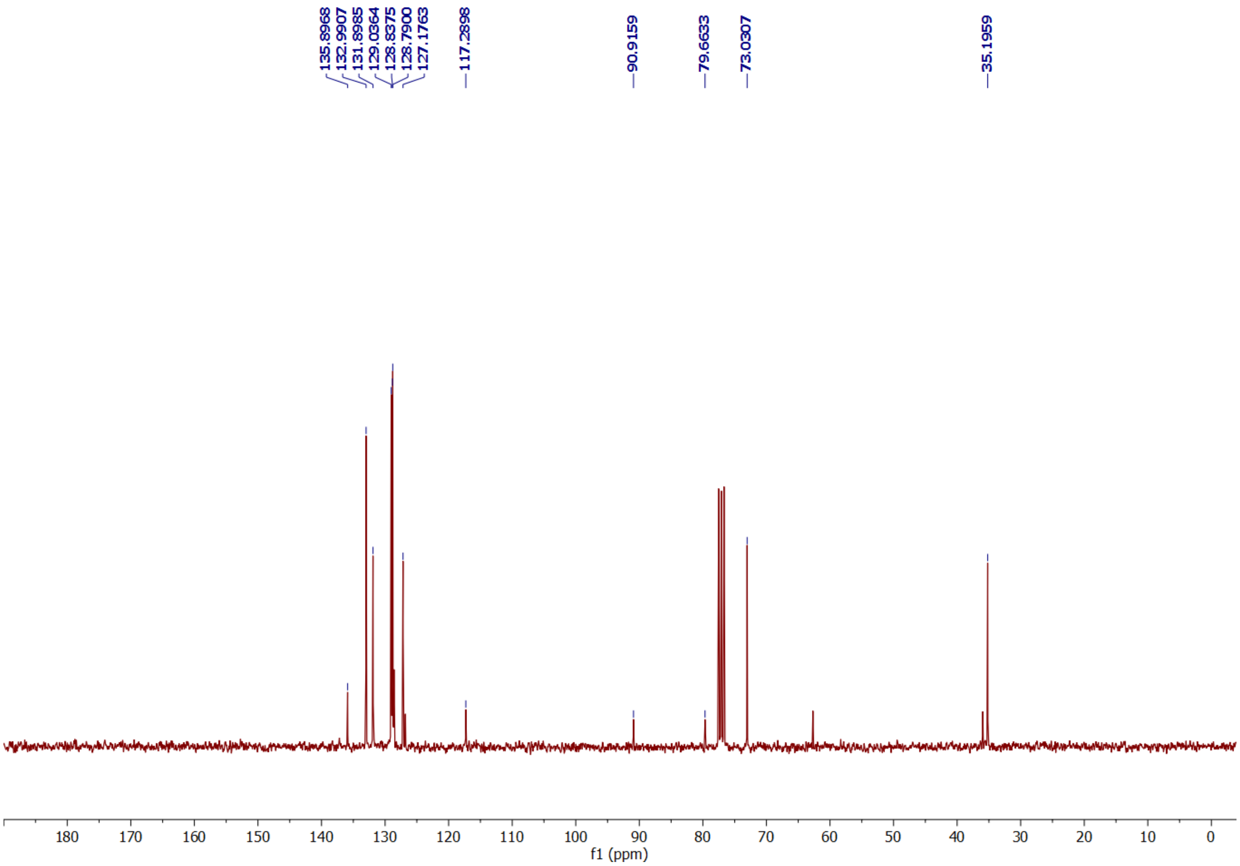

^{1}H NMR (400 MHz, $CDCl_3$) of **3aq** (see procedure)

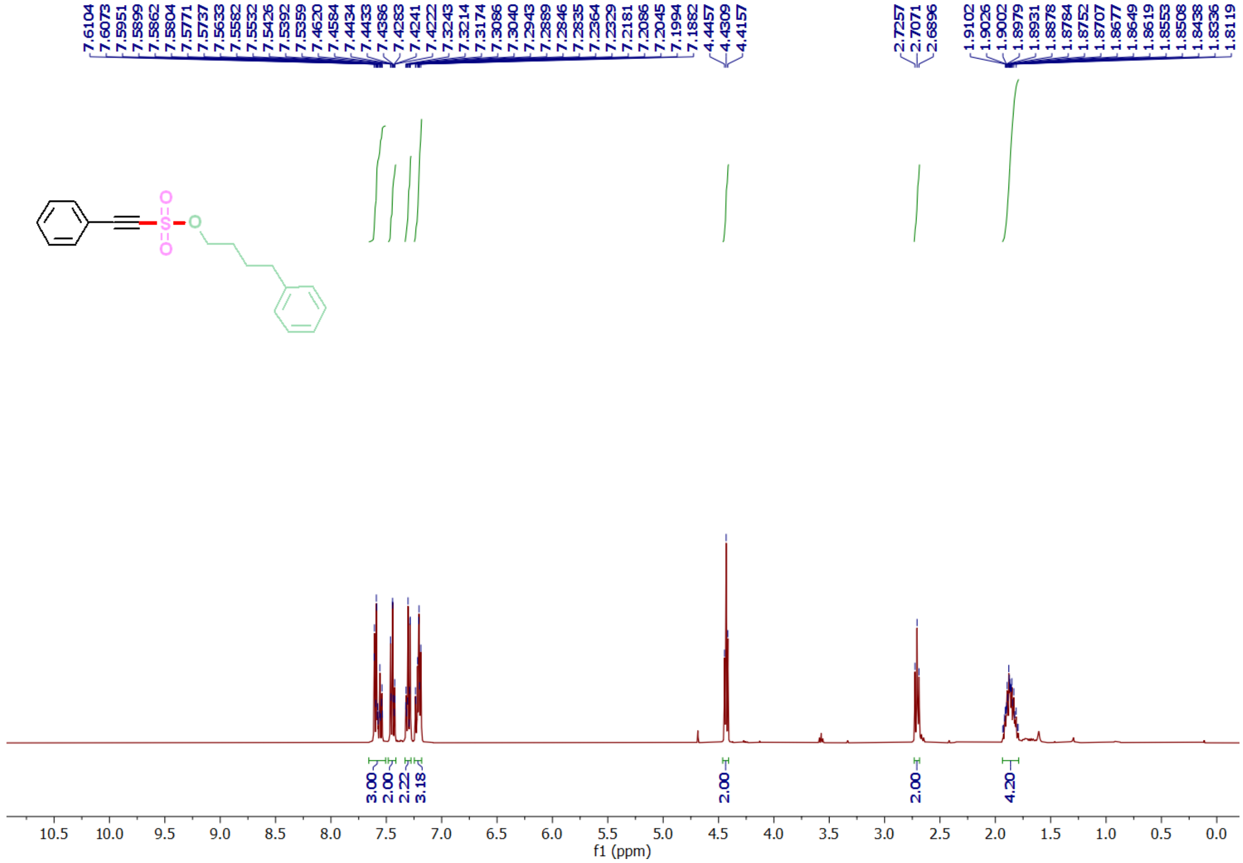

^{13}C NMR (101MHz, $CDCl_3$) of **3aq**

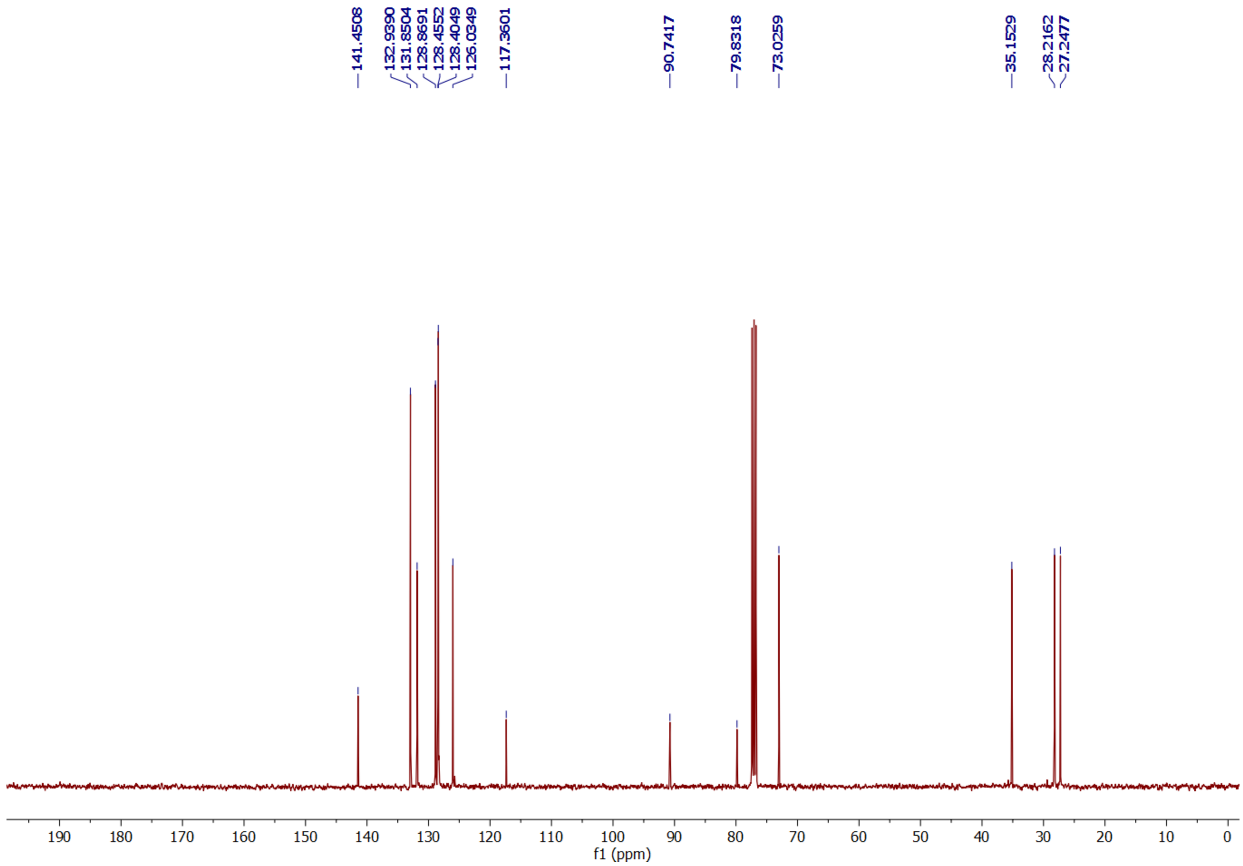

^{1}H NMR (400 MHz, $CDCl_3$) of **3ar** (see procedure)

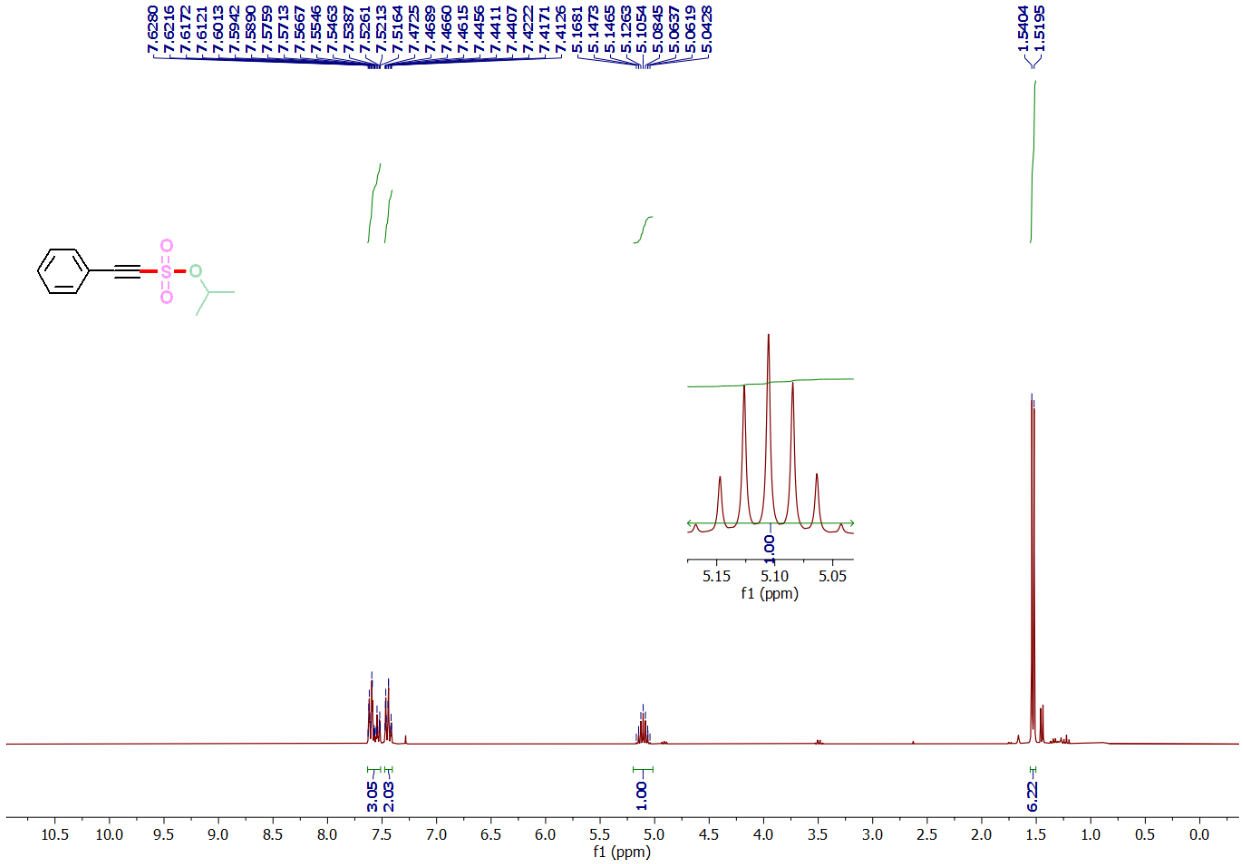

^{13}C NMR (101MHz, $CDCl_3$) of **3ar**

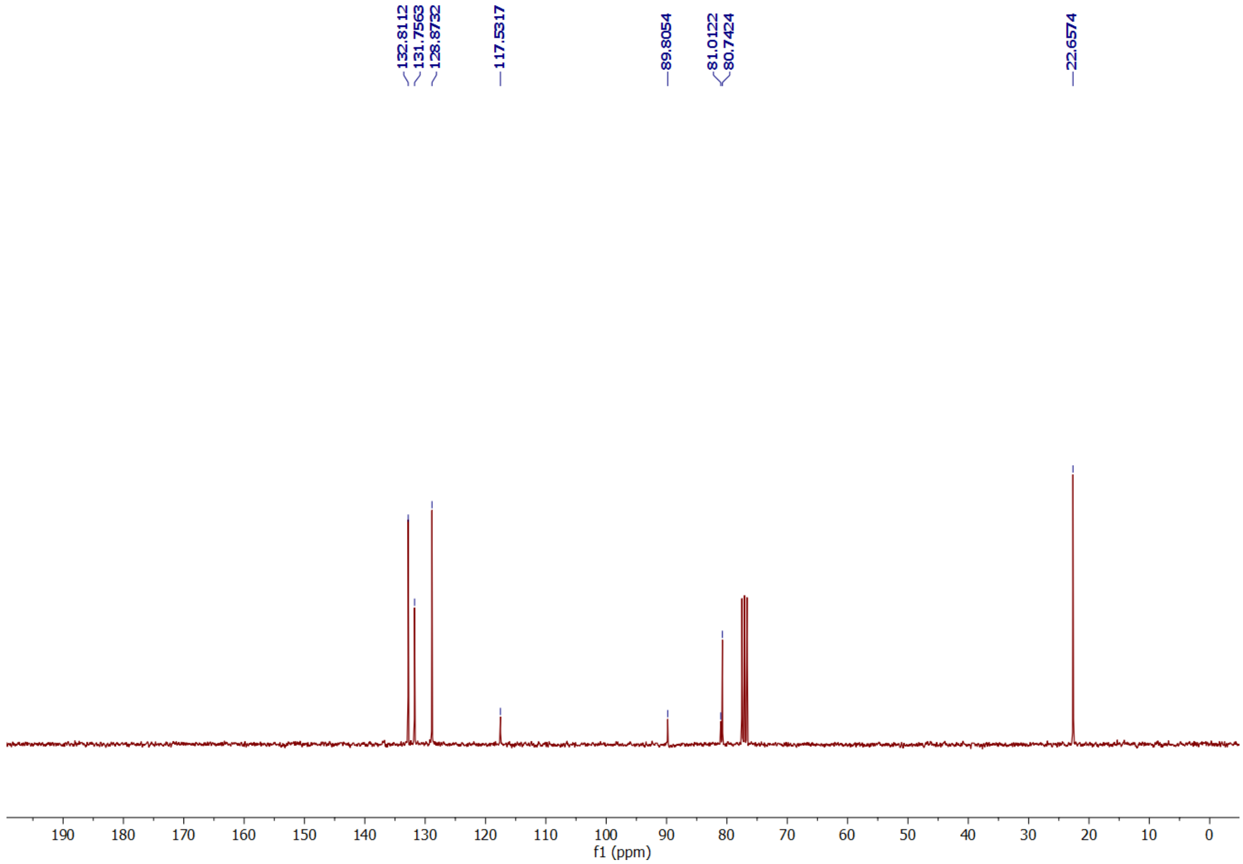

^{1}H NMR (400 MHz, $CDCl_3$) of **3as** (see procedure)

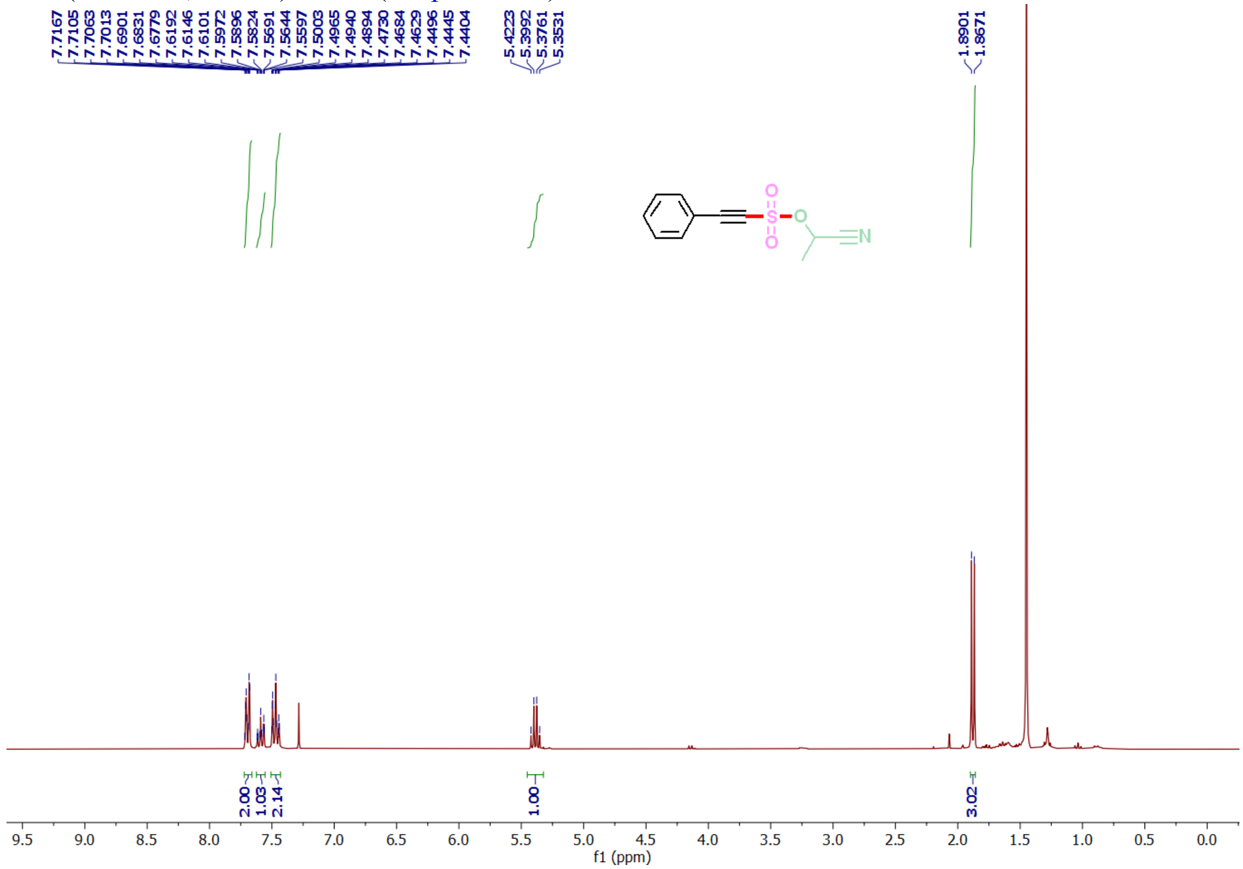

^{13}C NMR (101MHz, $CDCl_3$) of **3as**

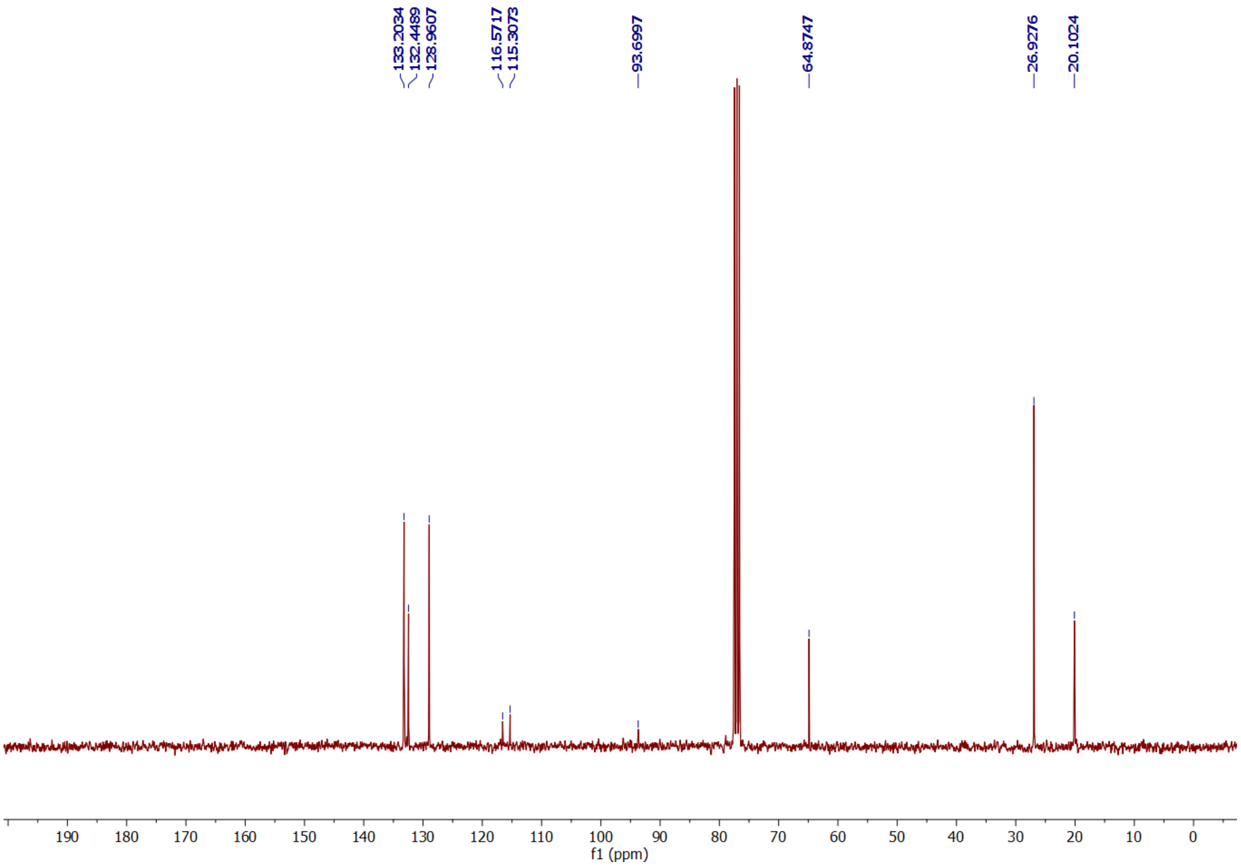

^{1}H NMR (400 MHz, $CDCl_3$) of **3at** (see procedure)

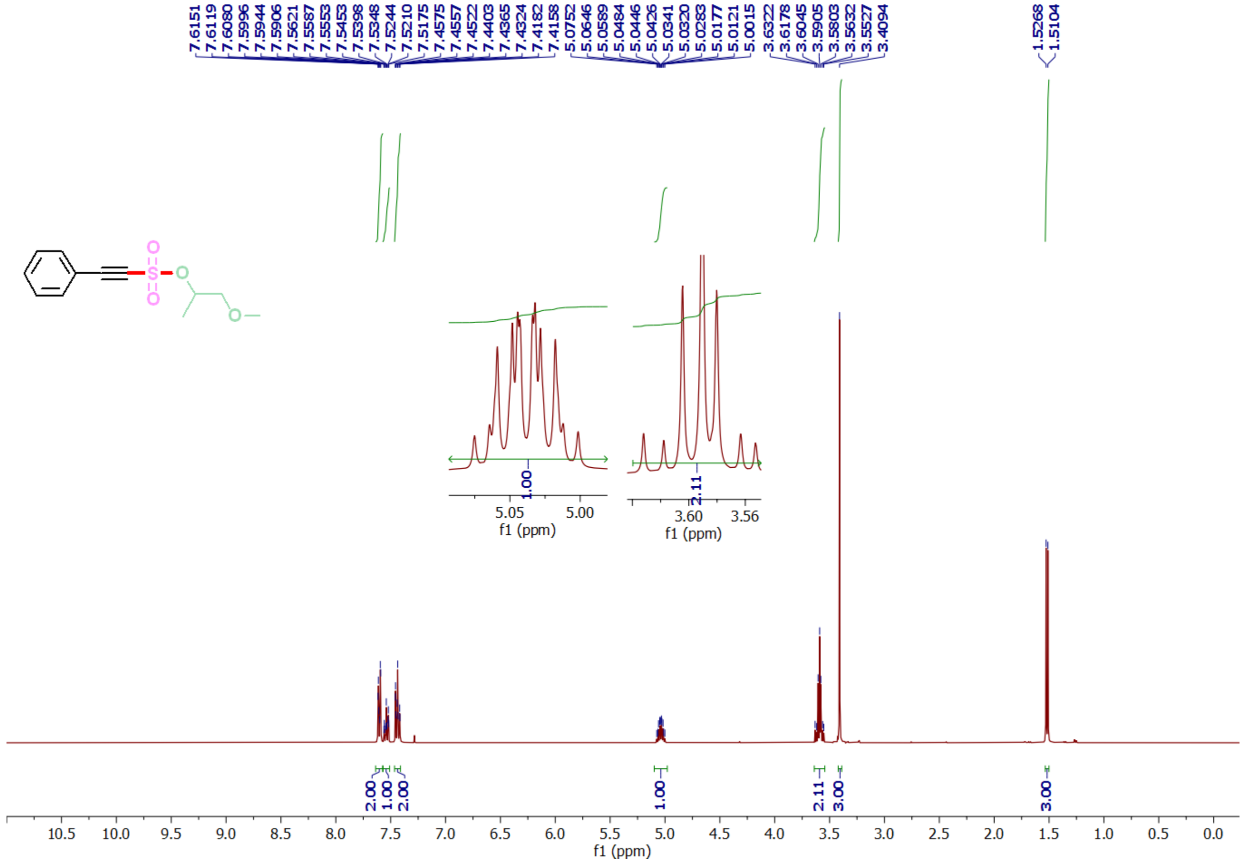

^{13}C NMR (101MHz, $CDCl_3$) of **3at**

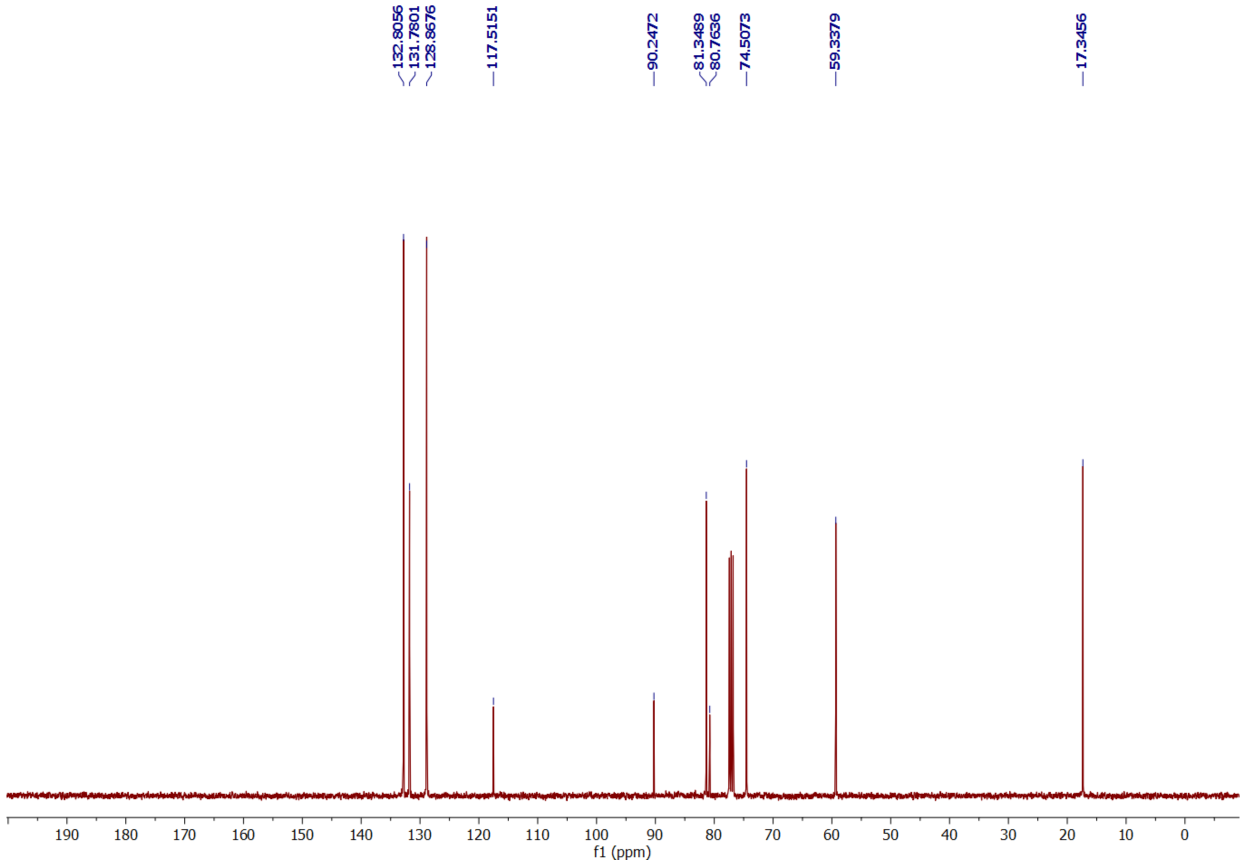

^{1}H NMR (400 MHz, $CDCl_3$) of **3au** (see procedure)

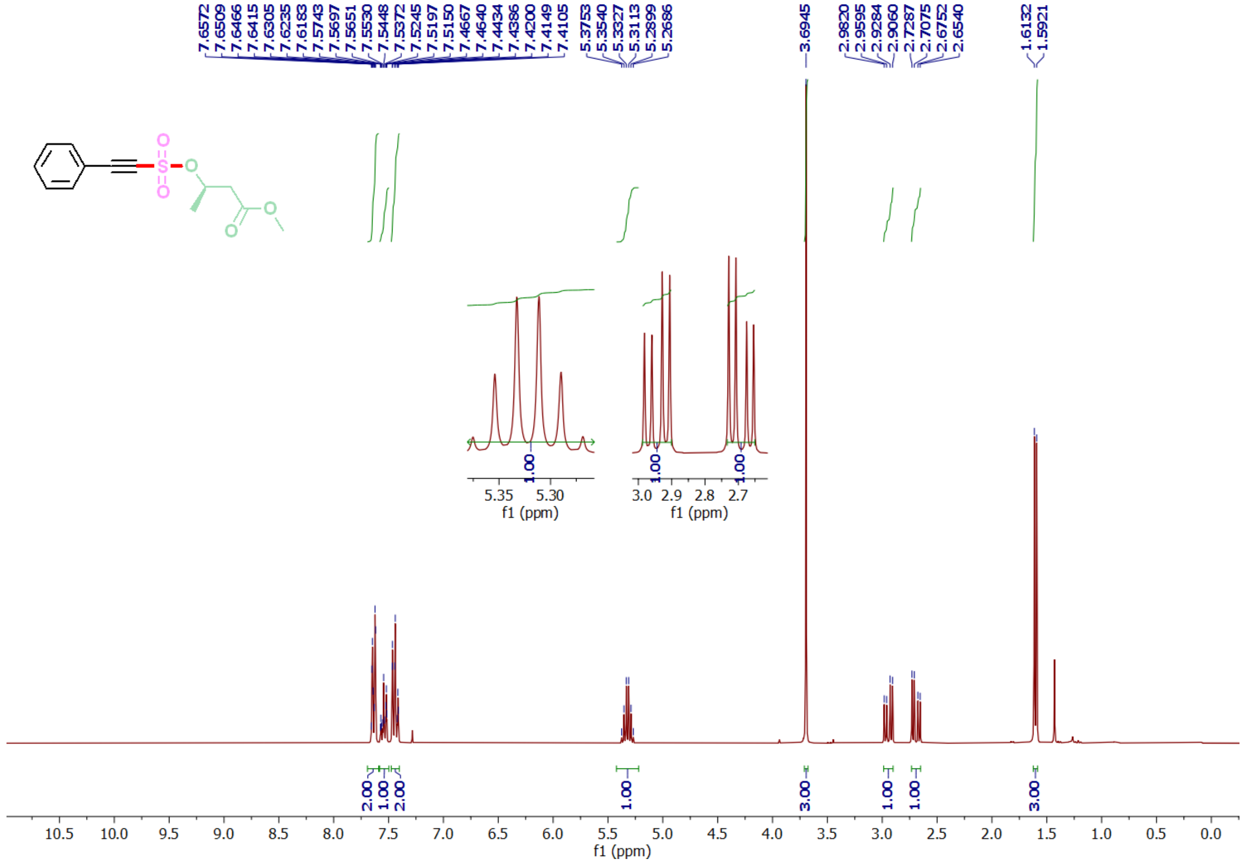

^{13}C NMR (300MHz, $CDCl_3$) of **3au**

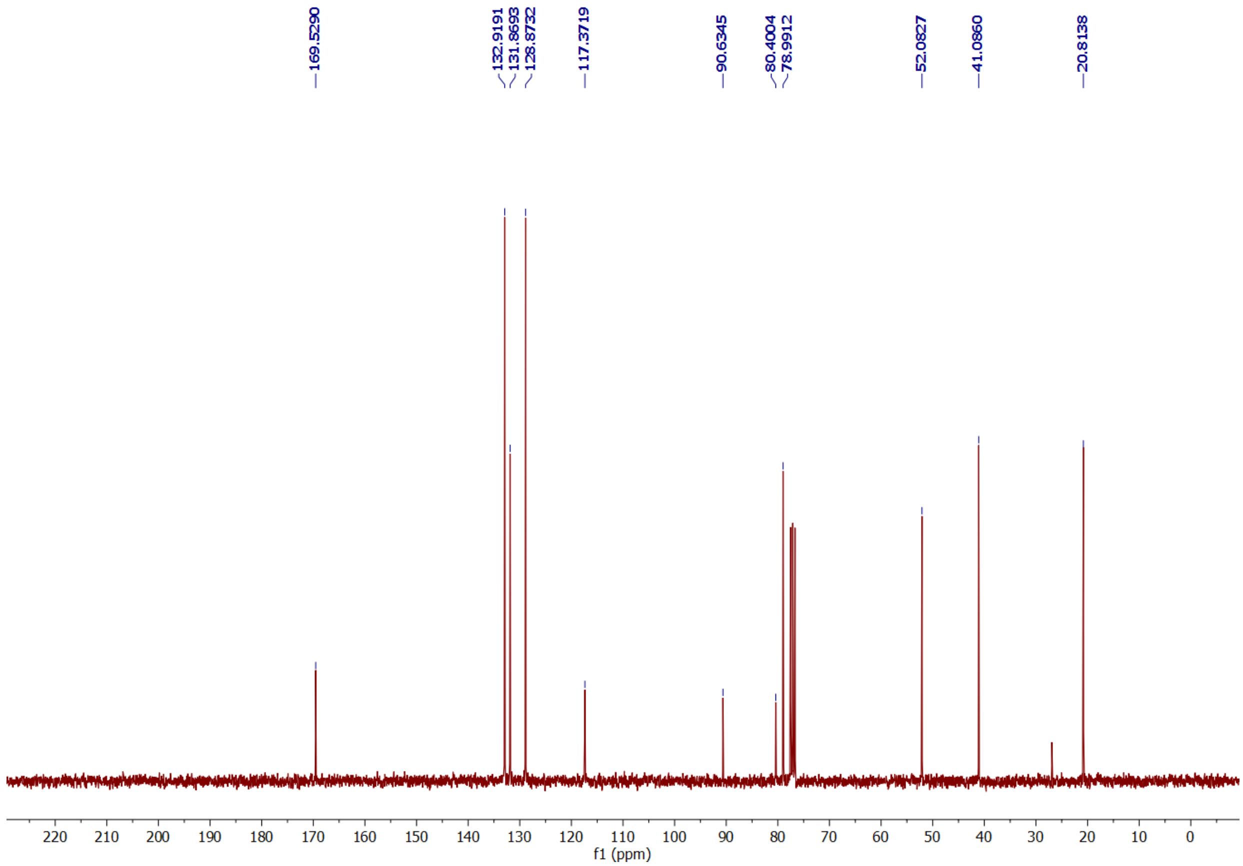

^{1}H NMR (400 MHz, $CDCl_3$) of **3av** (see procedure)

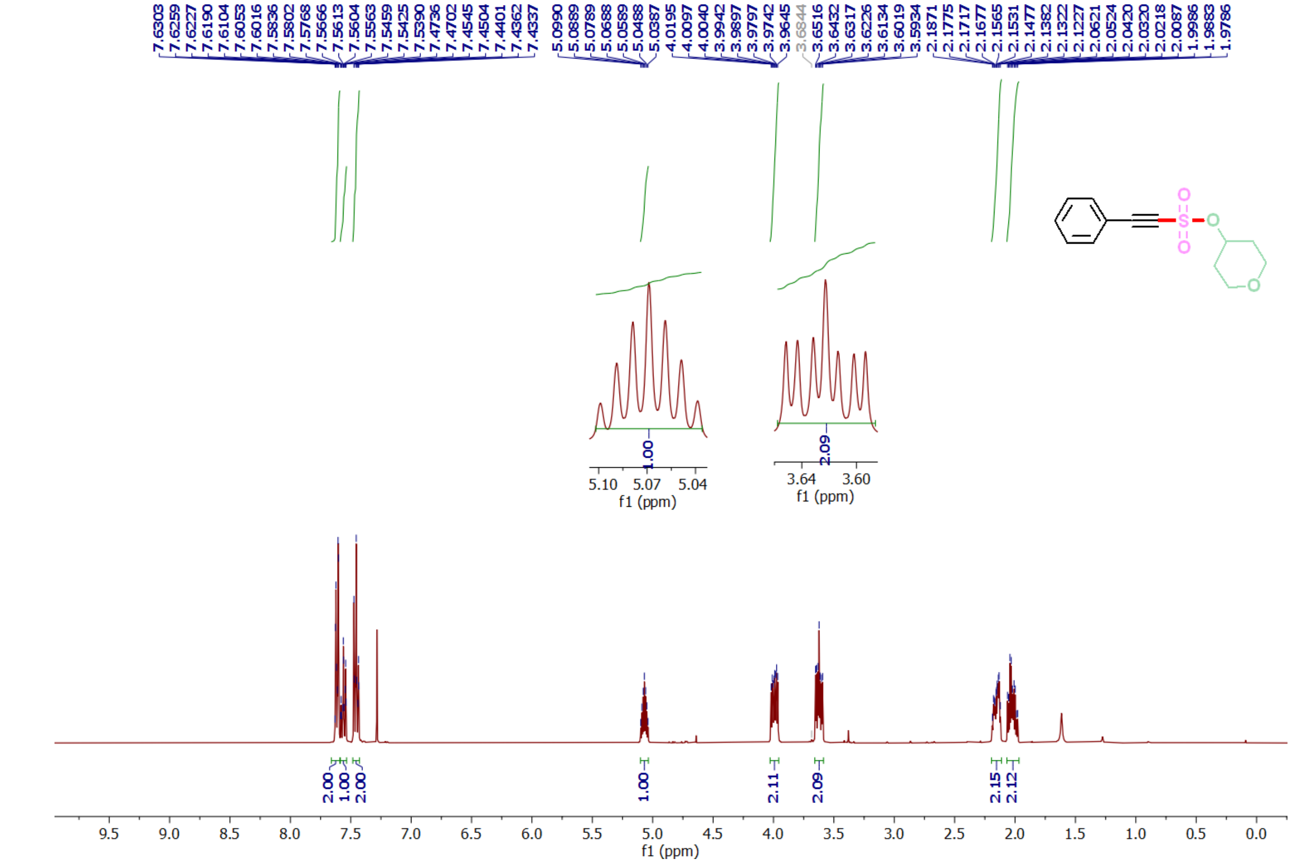

^{13}C NMR (101MHz, $CDCl_3$) of **3av**

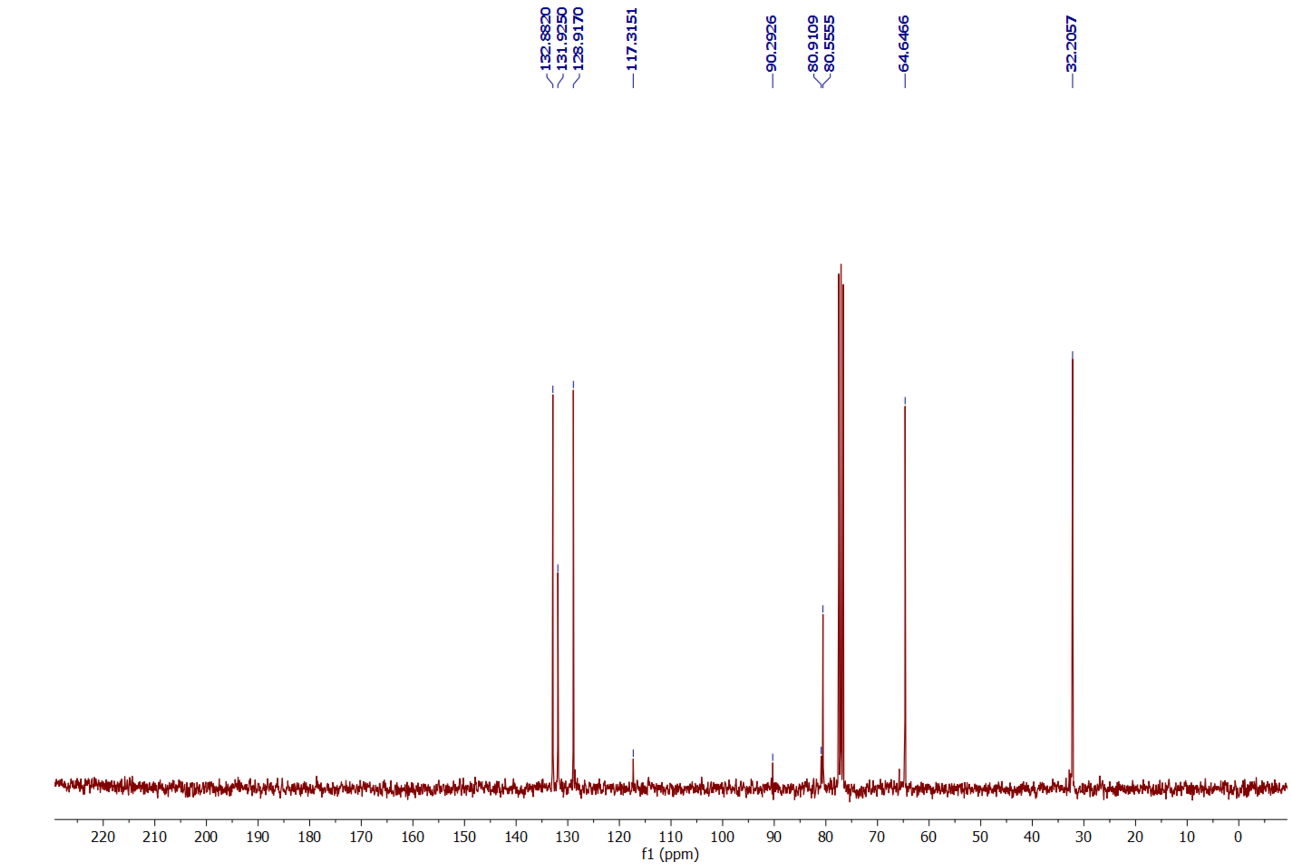

^{1}H NMR (400 MHz, $CDCl_3$) of **3aw** (see procedure)

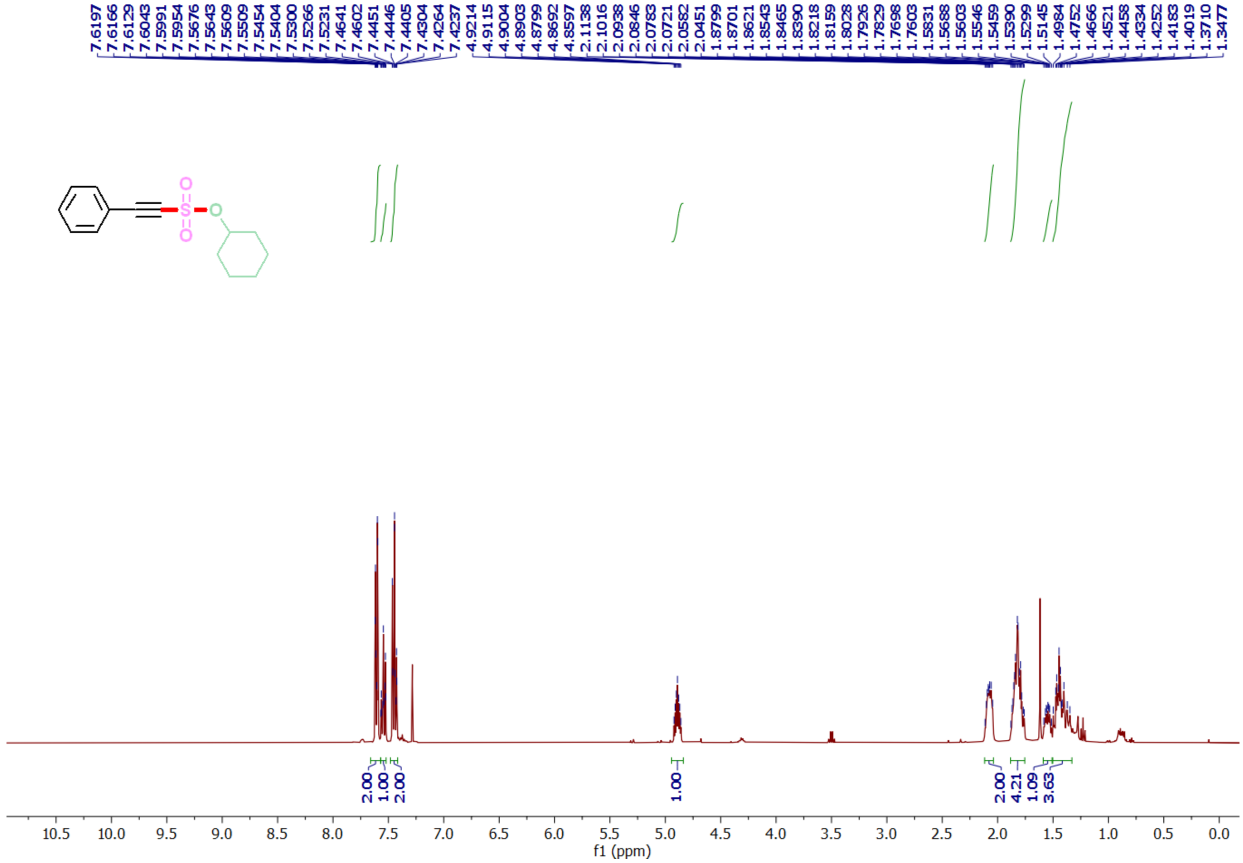

^{13}C NMR (101MHz, $CDCl_3$) of **3aw**

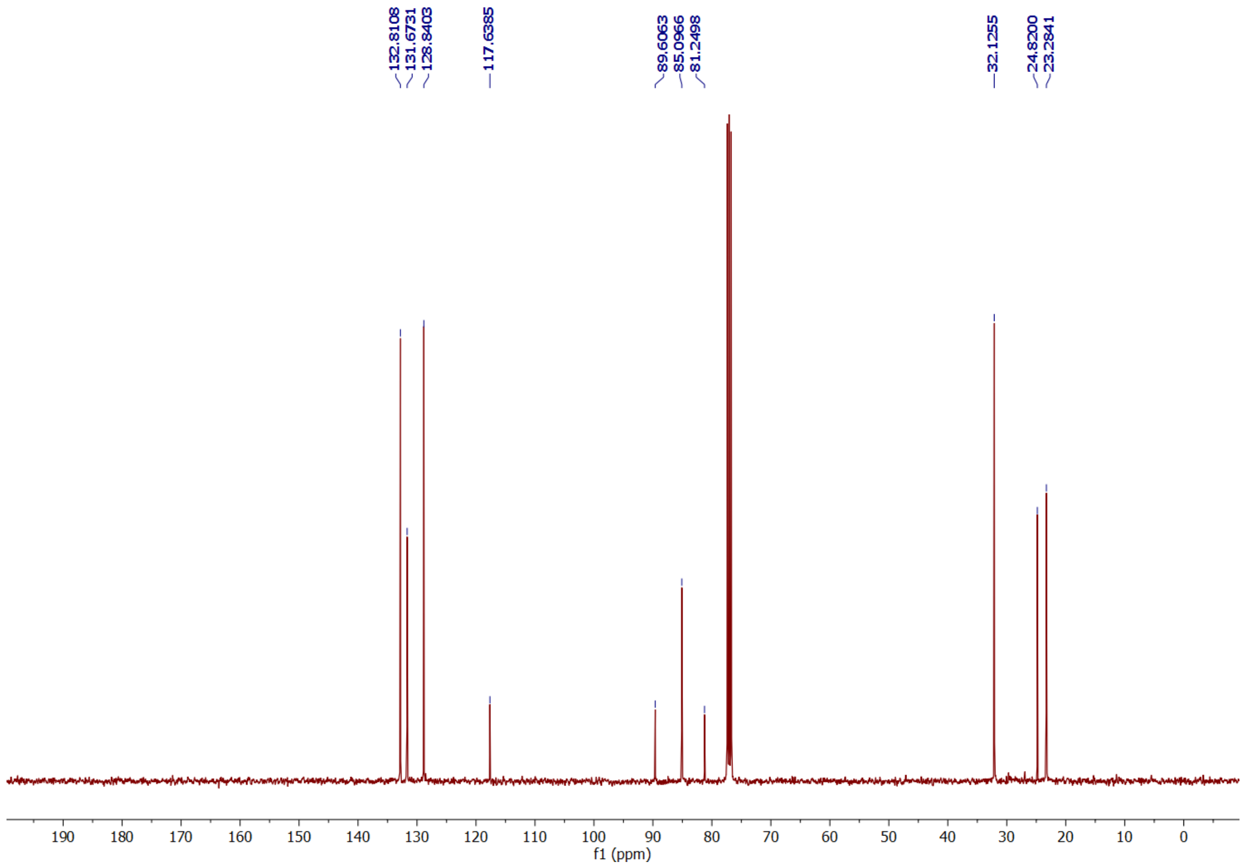

^{1}H NMR (400 MHz, $CDCl_3$) of **3ax** (see procedure)

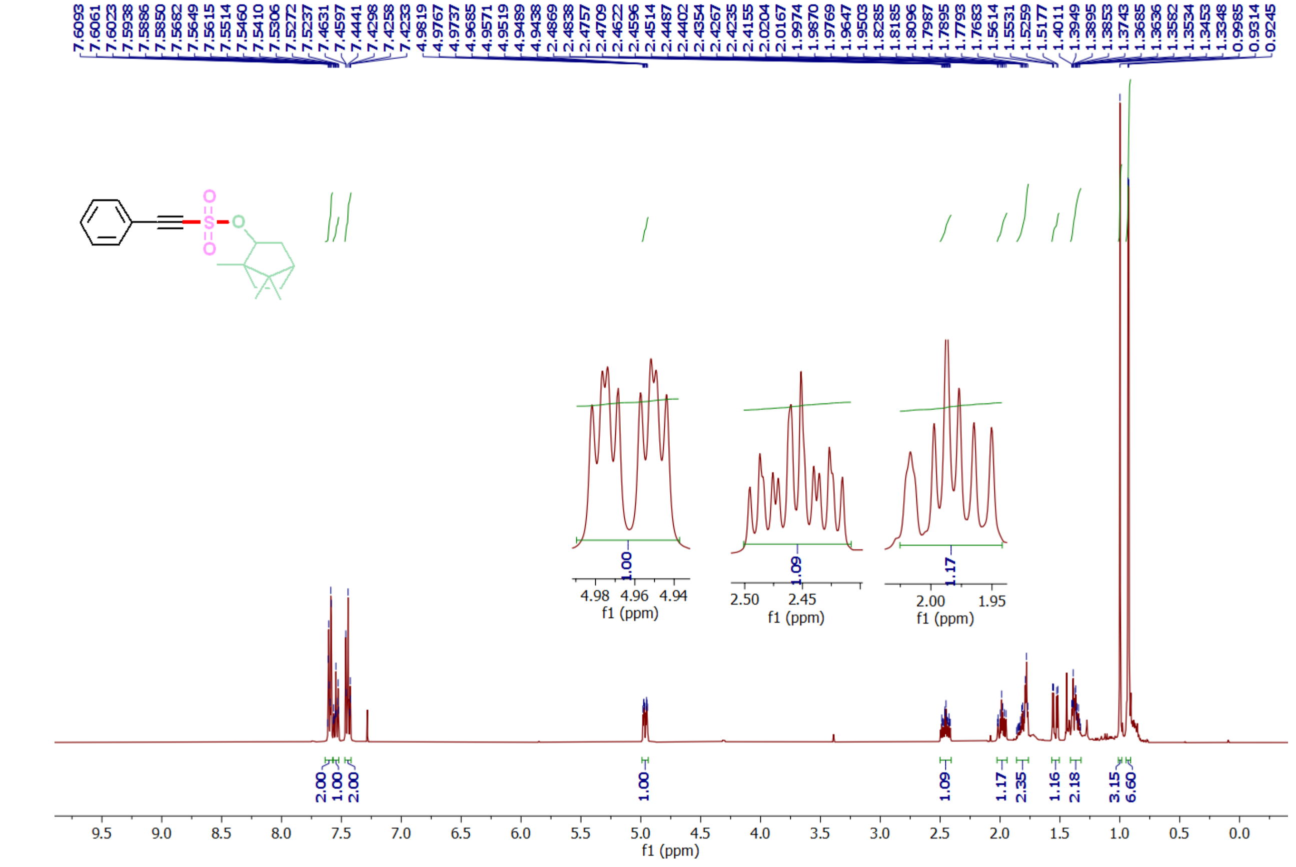

^{13}C NMR (101MHz, $CDCl_3$) of **3ax**

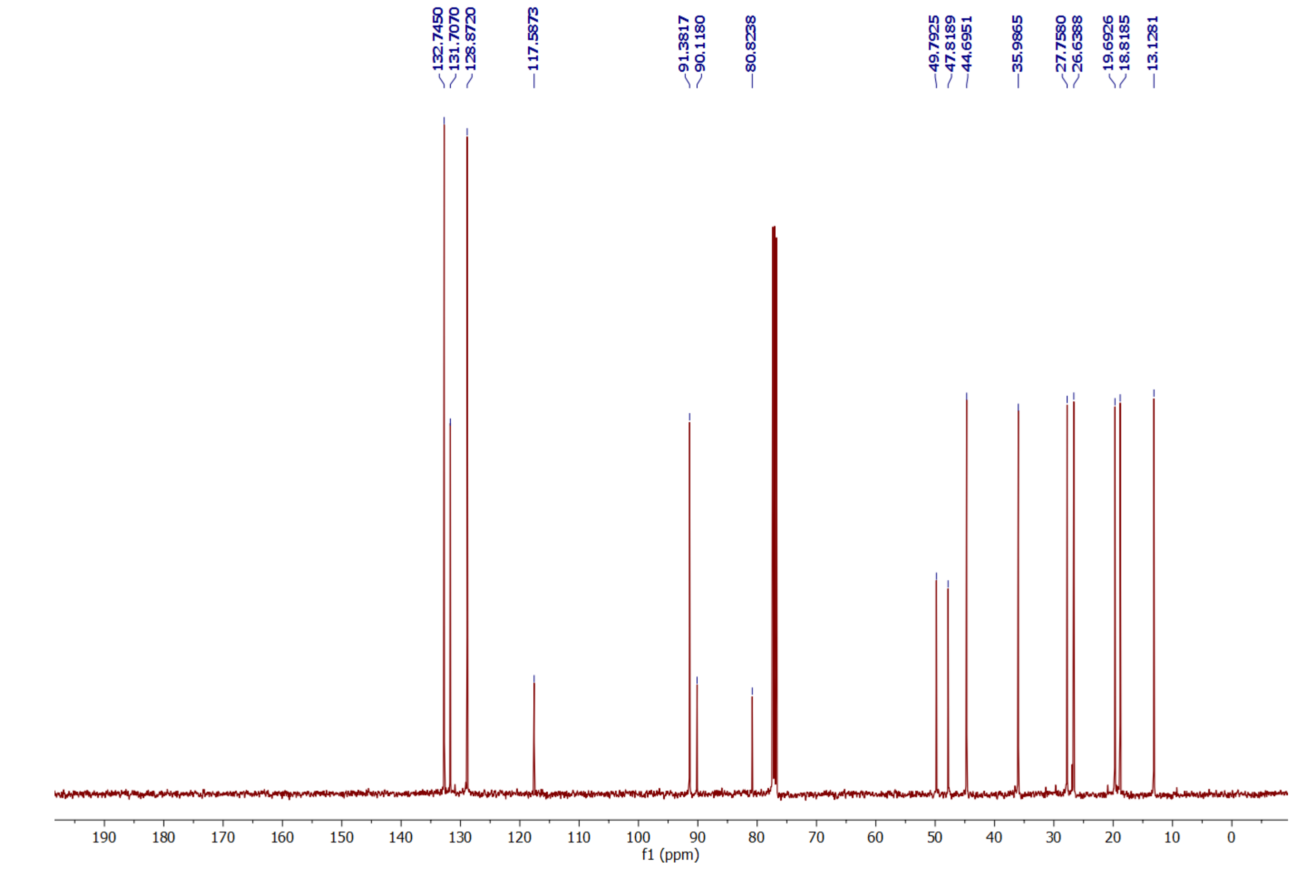

^{1}H NMR (400 MHz, $CDCl_3$) of **3ay** (see procedure)

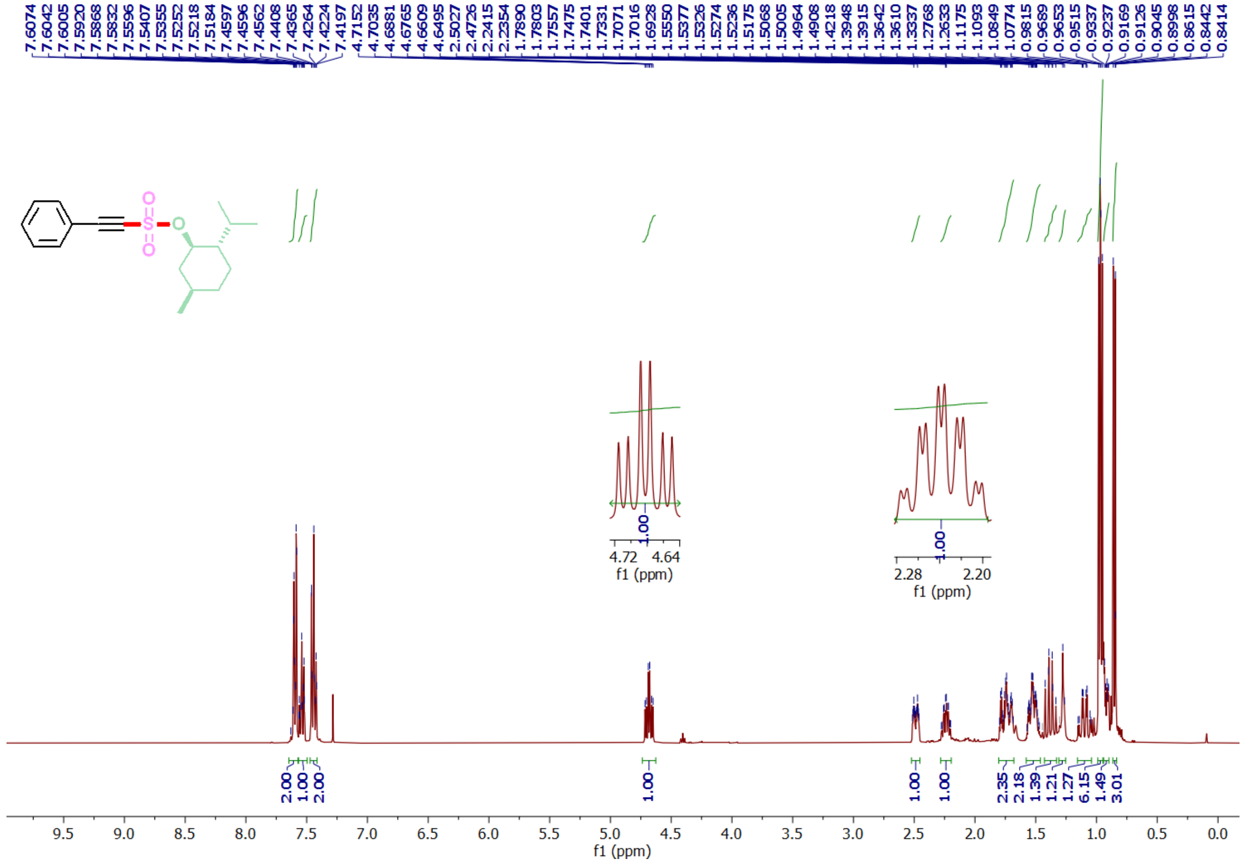

^{13}C NMR (101MHz, $CDCl_3$) of **3ay**

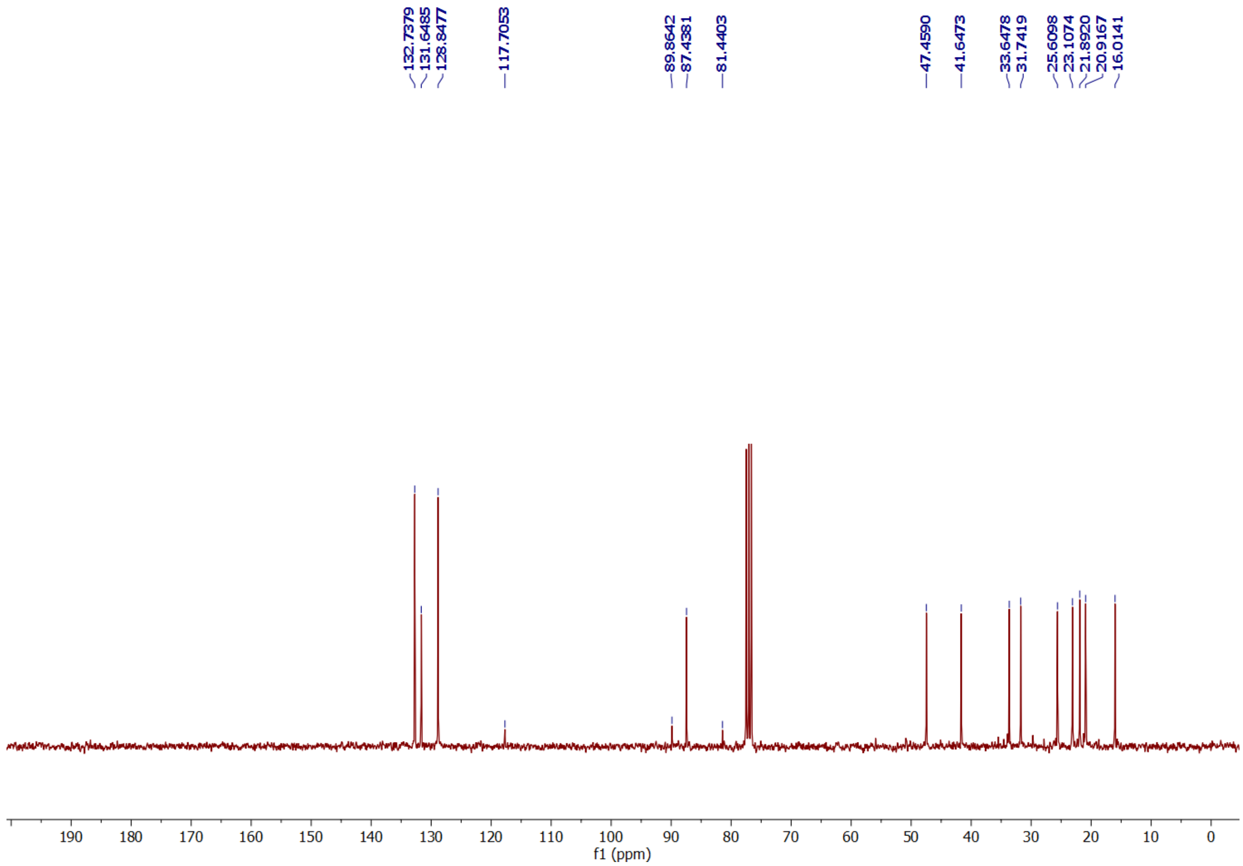

^{1}H NMR (400 MHz, $CDCl_3$) of **3az** (see procedure)

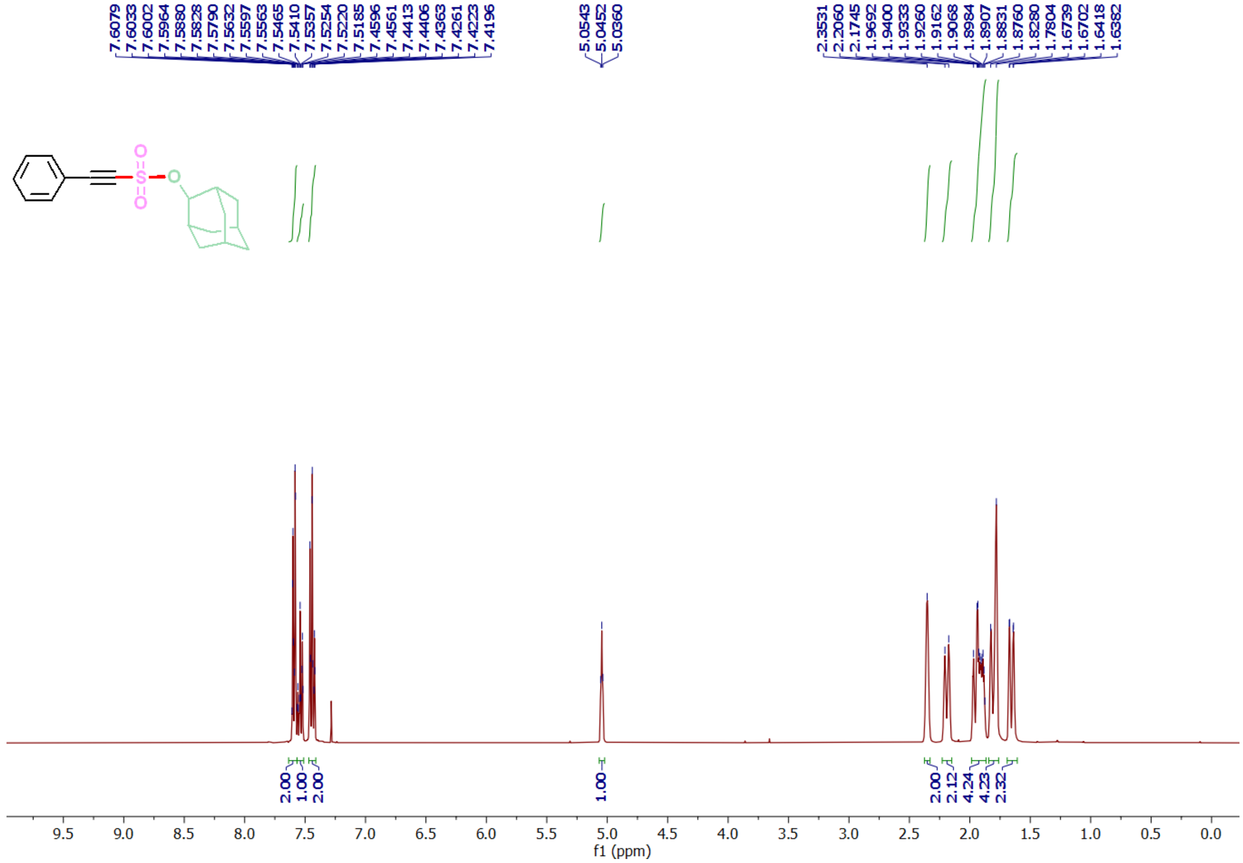

^{13}C NMR (101MHz, $CDCl_3$) of **3az**

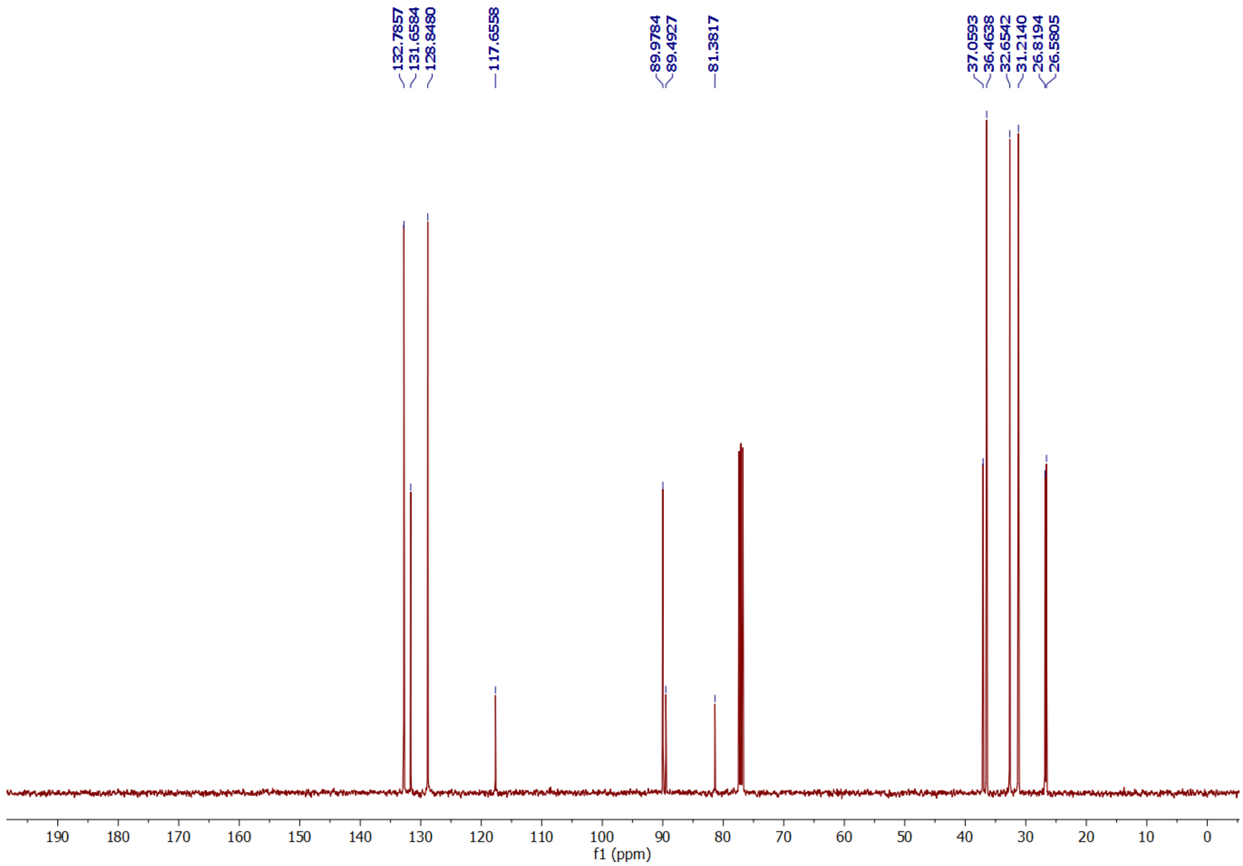

^{1}H NMR (400 MHz, $CDCl_3$) of **3aza** (see procedure)

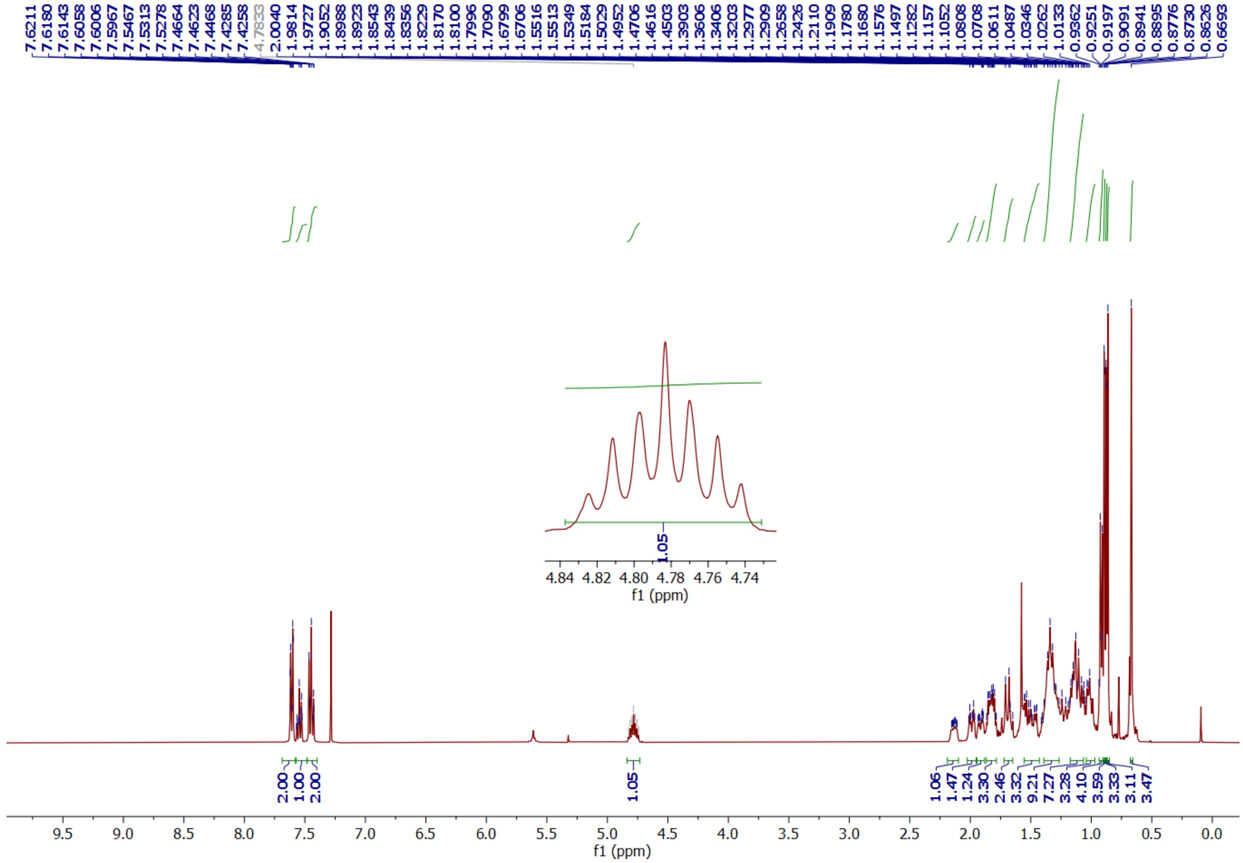

^{13}C NMR (101MHz, $CDCl_3$) of **3aza**

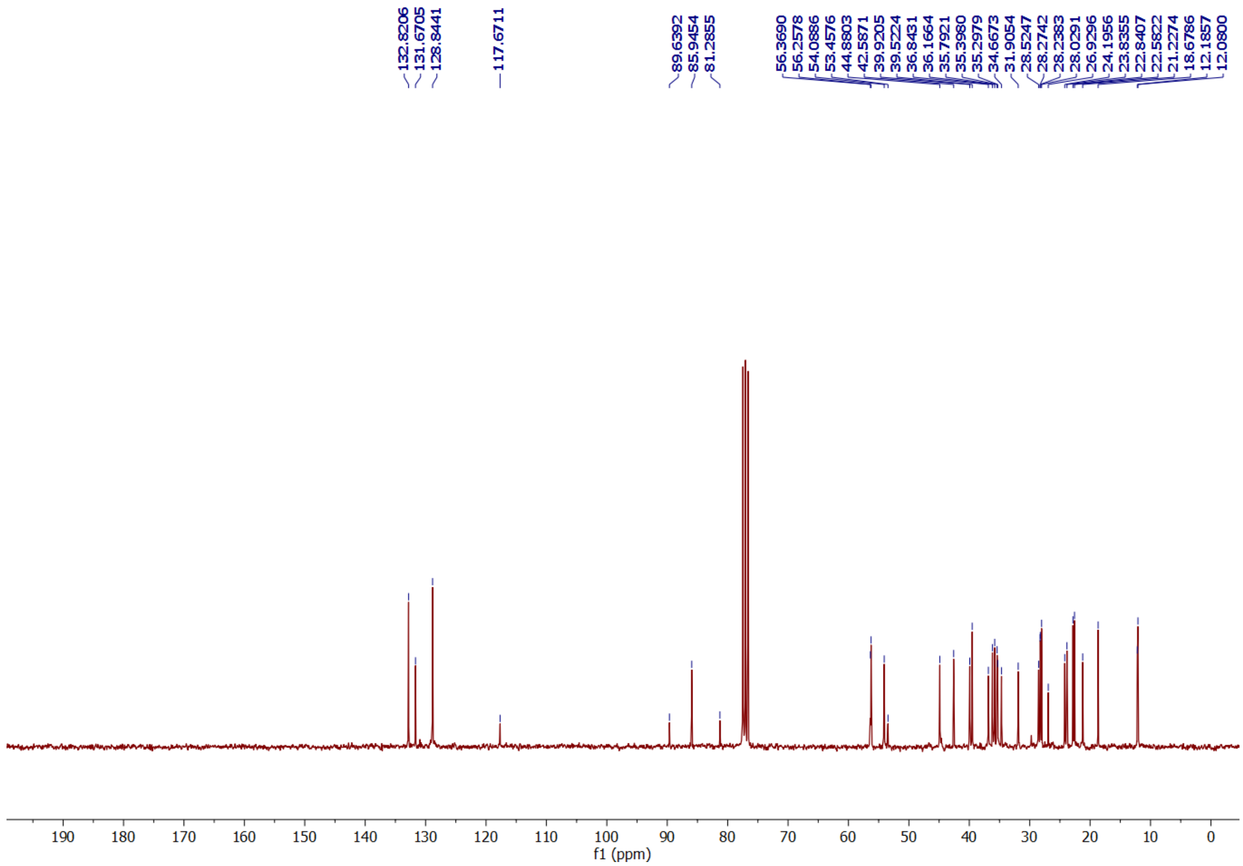

^{1}H NMR (400 MHz, $CDCl_3$) of **3azb** (see procedure)

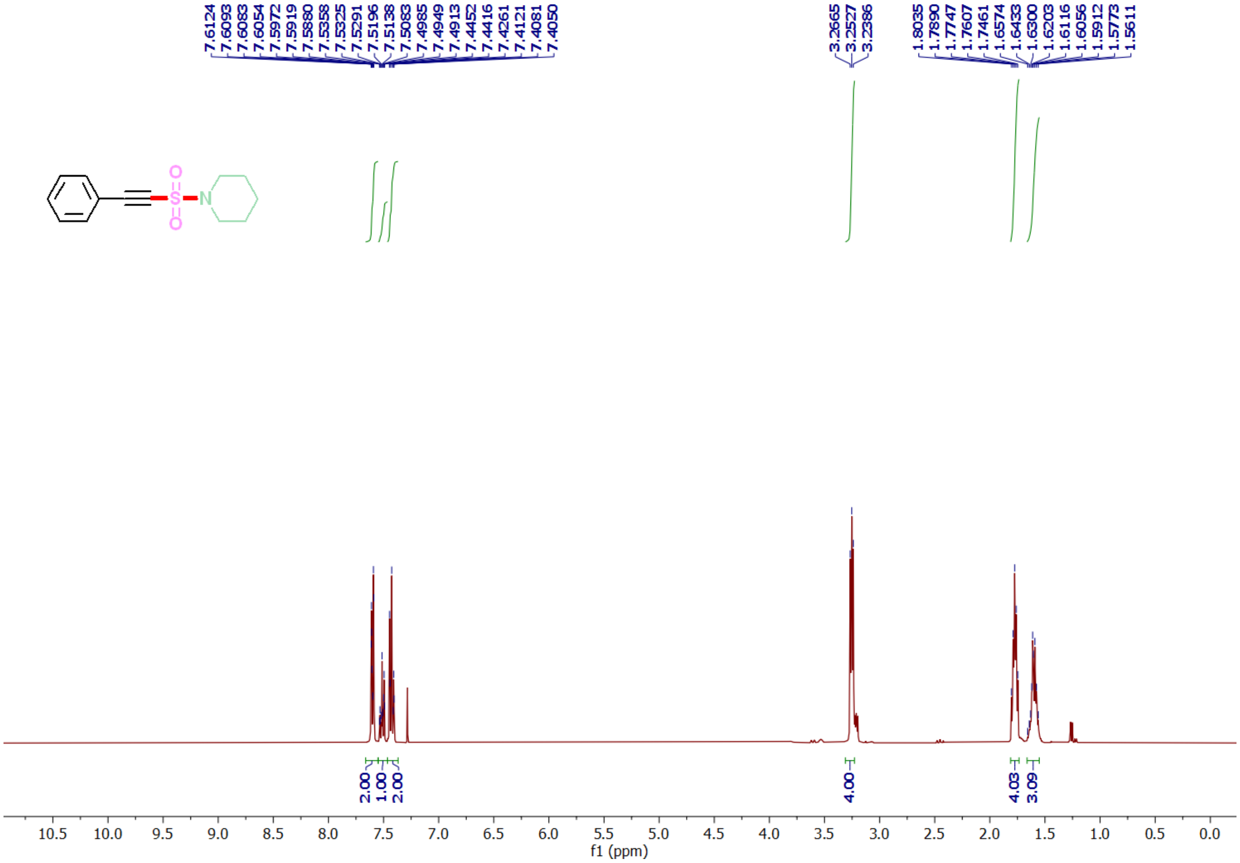

^{13}C NMR (101MHz, $CDCl_3$) of **3azb**

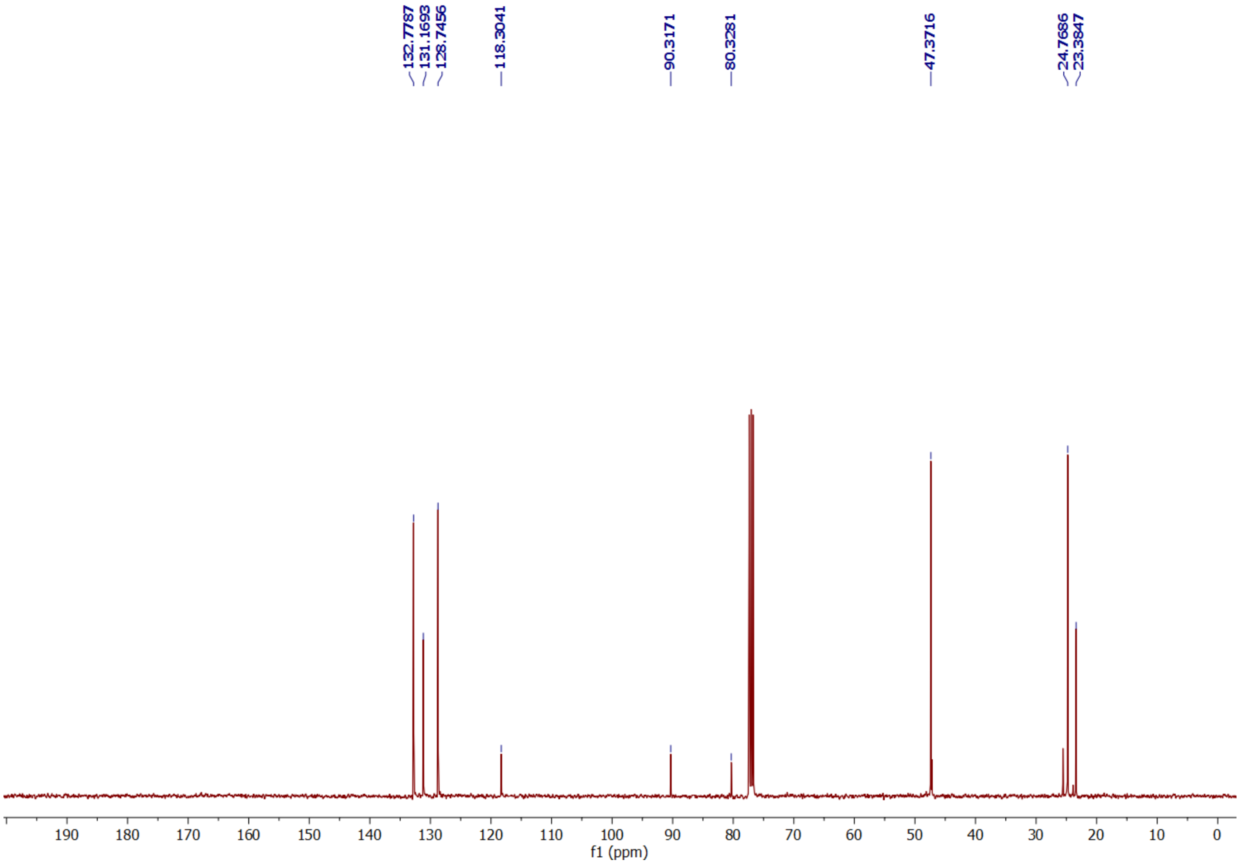

^{1}H NMR (400 MHz, $CDCl_3$) of **3azc** (see procedure)

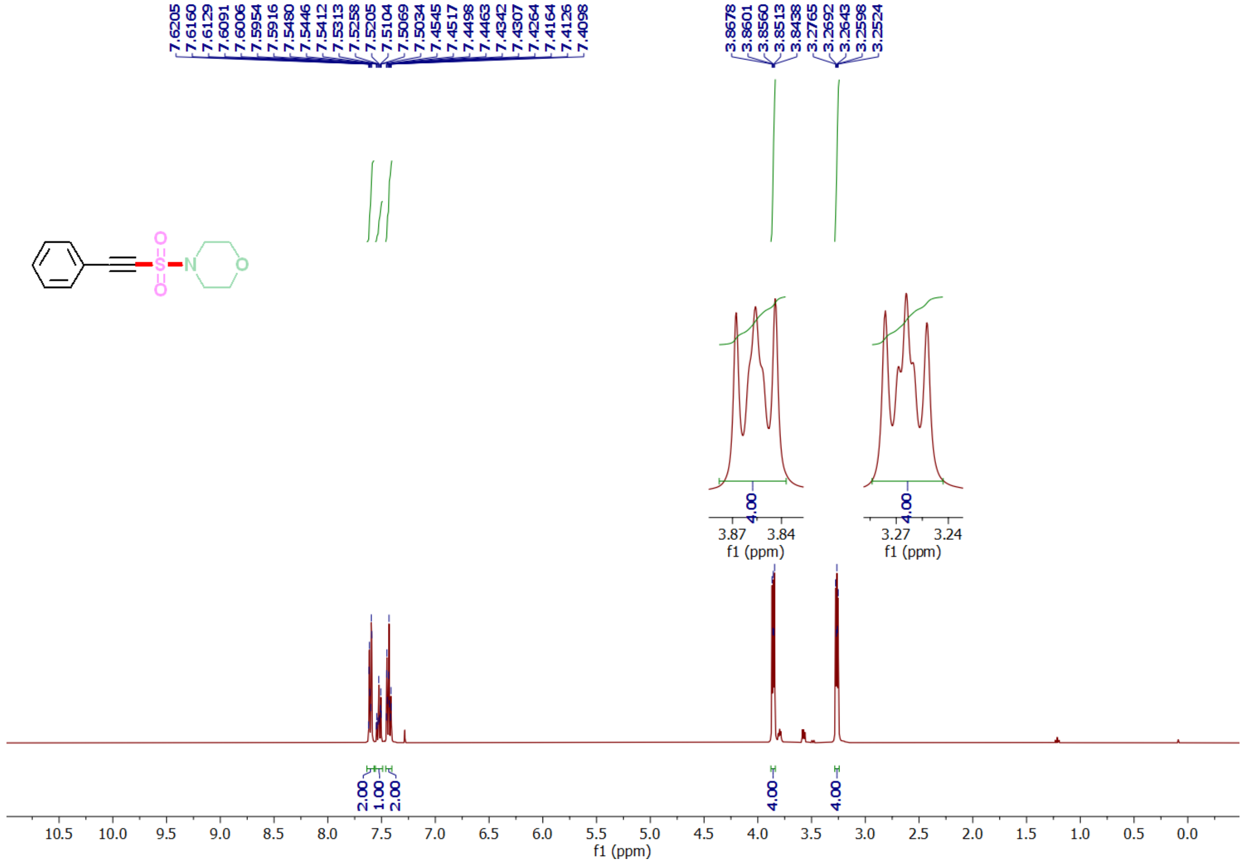

^{13}C NMR (101MHz, $CDCl_3$) of **3azc**

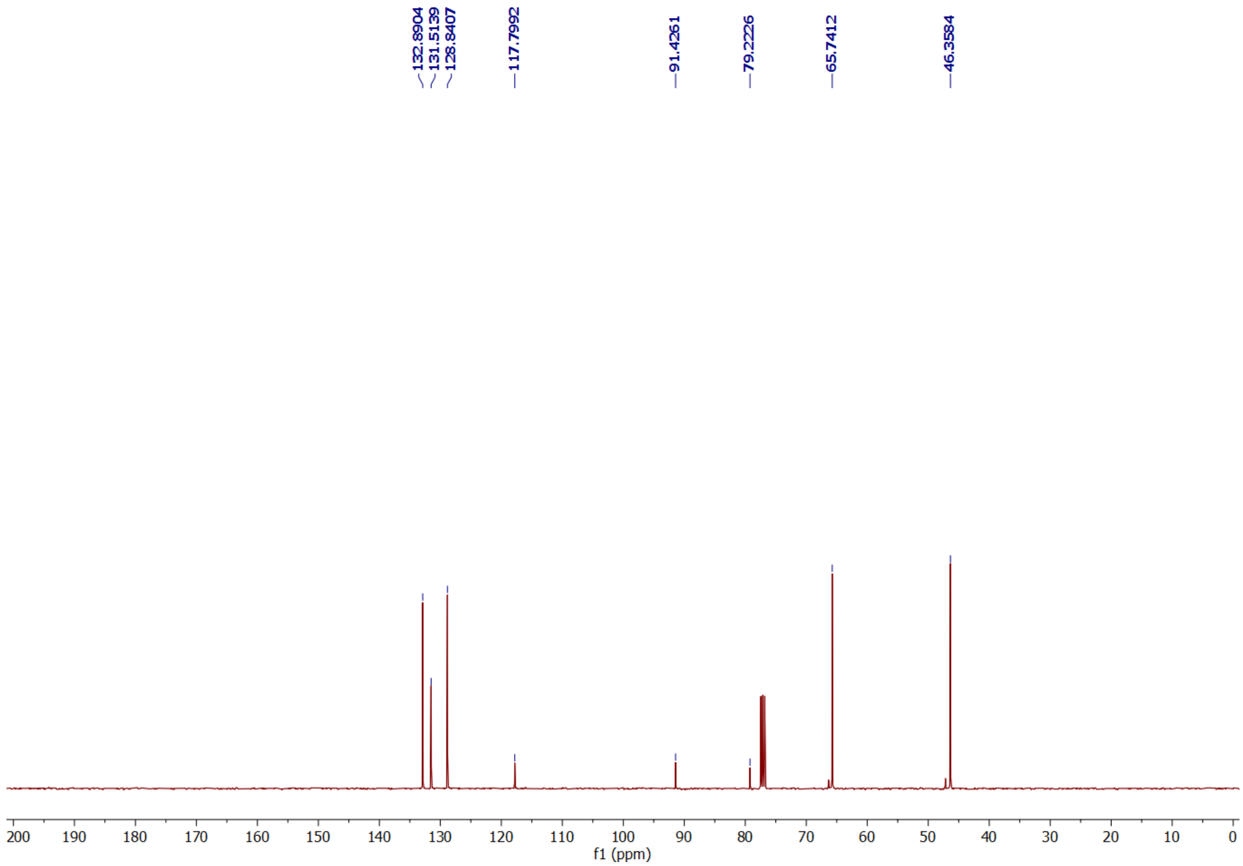

^{1}H NMR (400 MHz, $CDCl_3$) of **3azd** (see procedure)

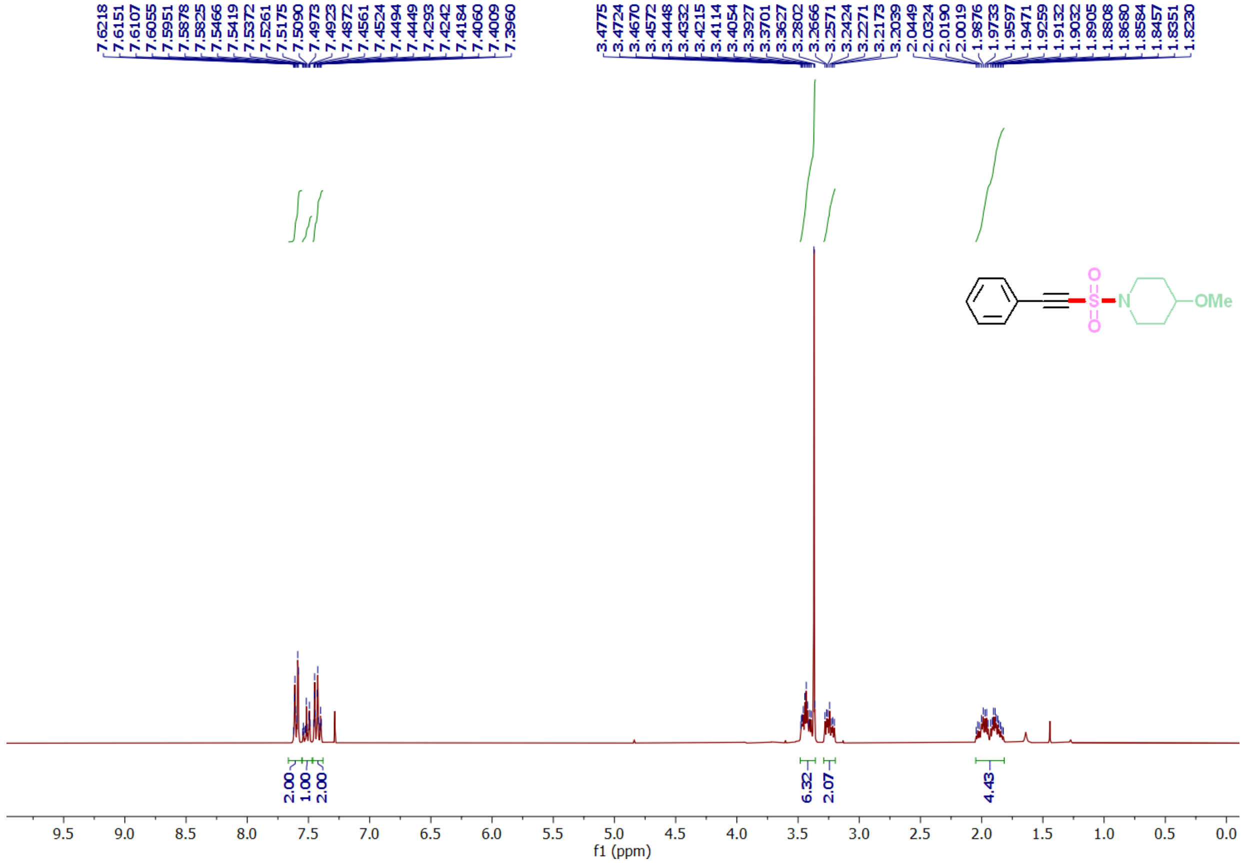

^{13}C NMR (101MHz, $CDCl_3$) of **3azd**

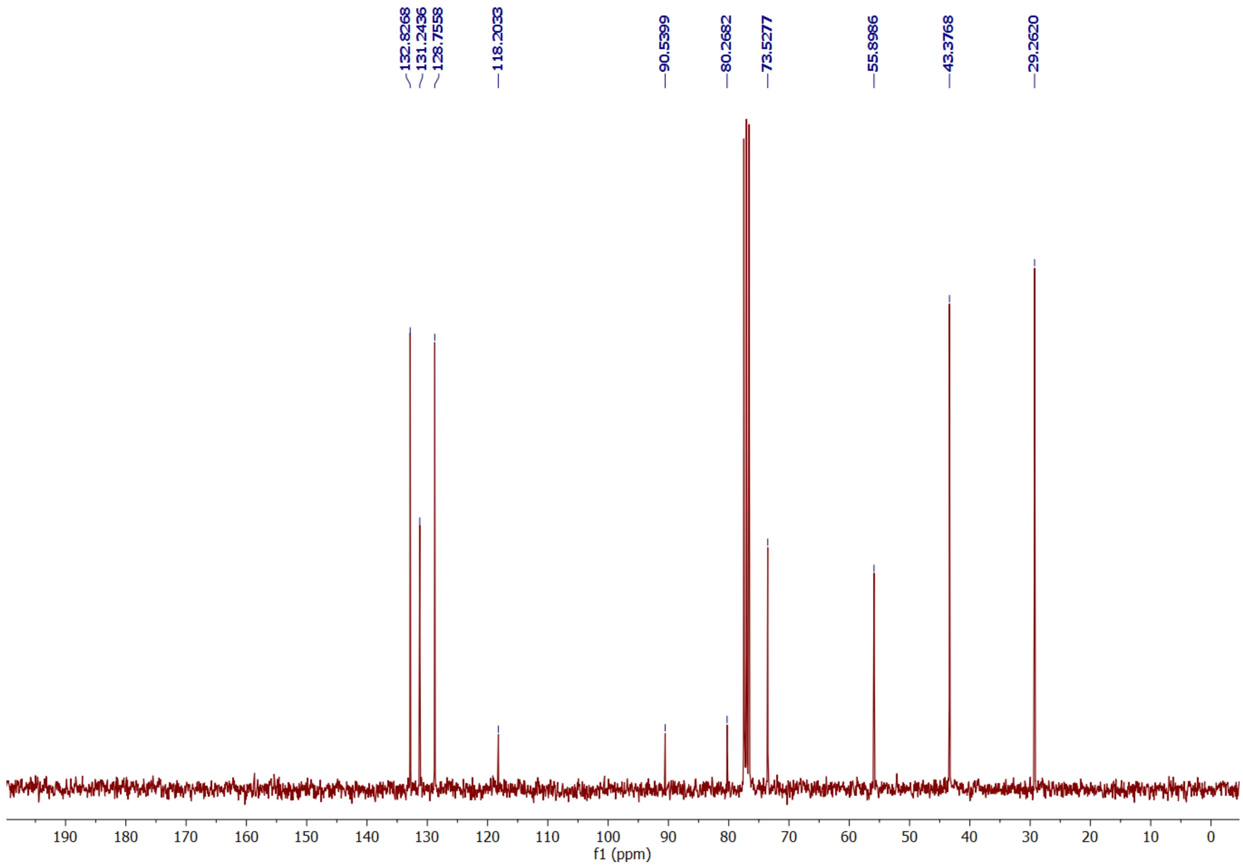

^{1}H NMR (400 MHz, $CDCl_3$) of **3aze** (see procedure)

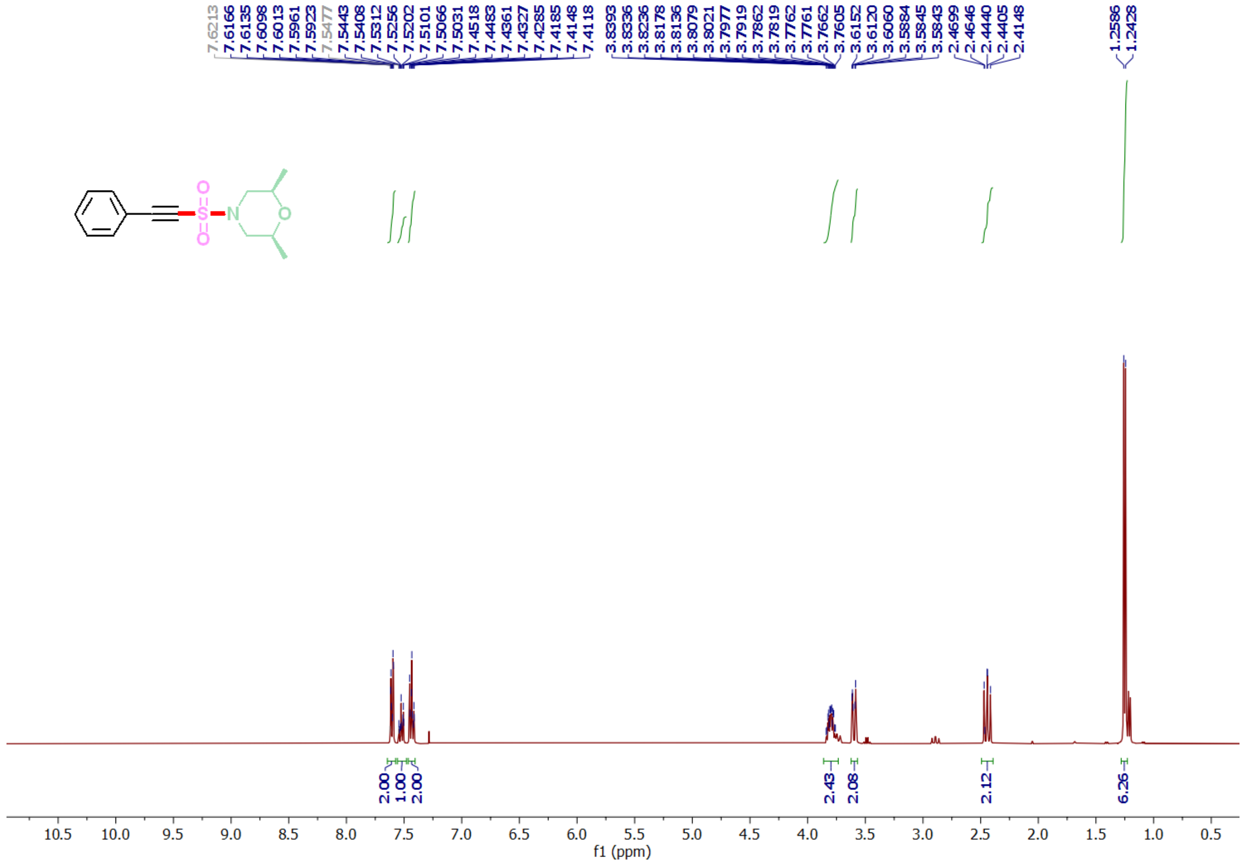

^{13}C NMR (101MHz, $CDCl_3$) of **3aze**

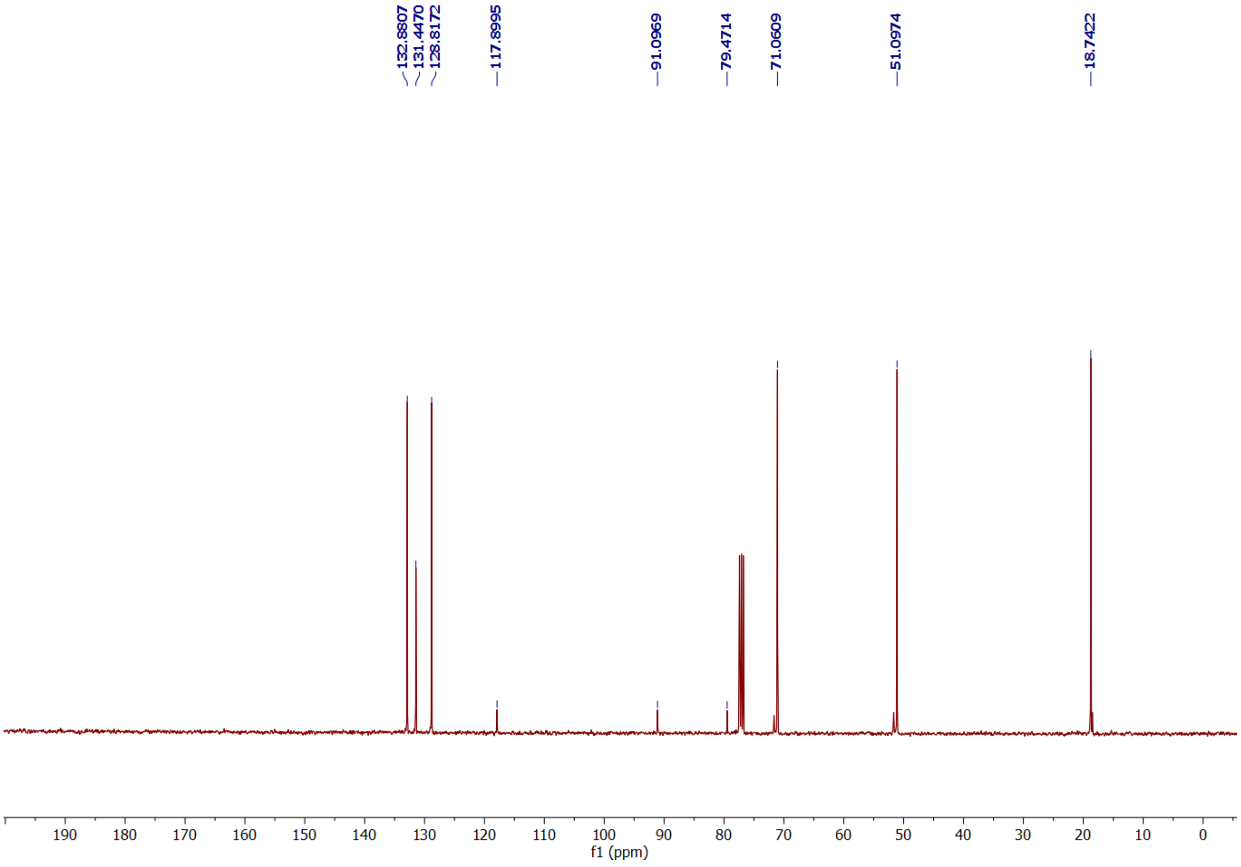

^{1}H NMR (400 MHz, $CDCl_3$) of **3azf**(see procedure)

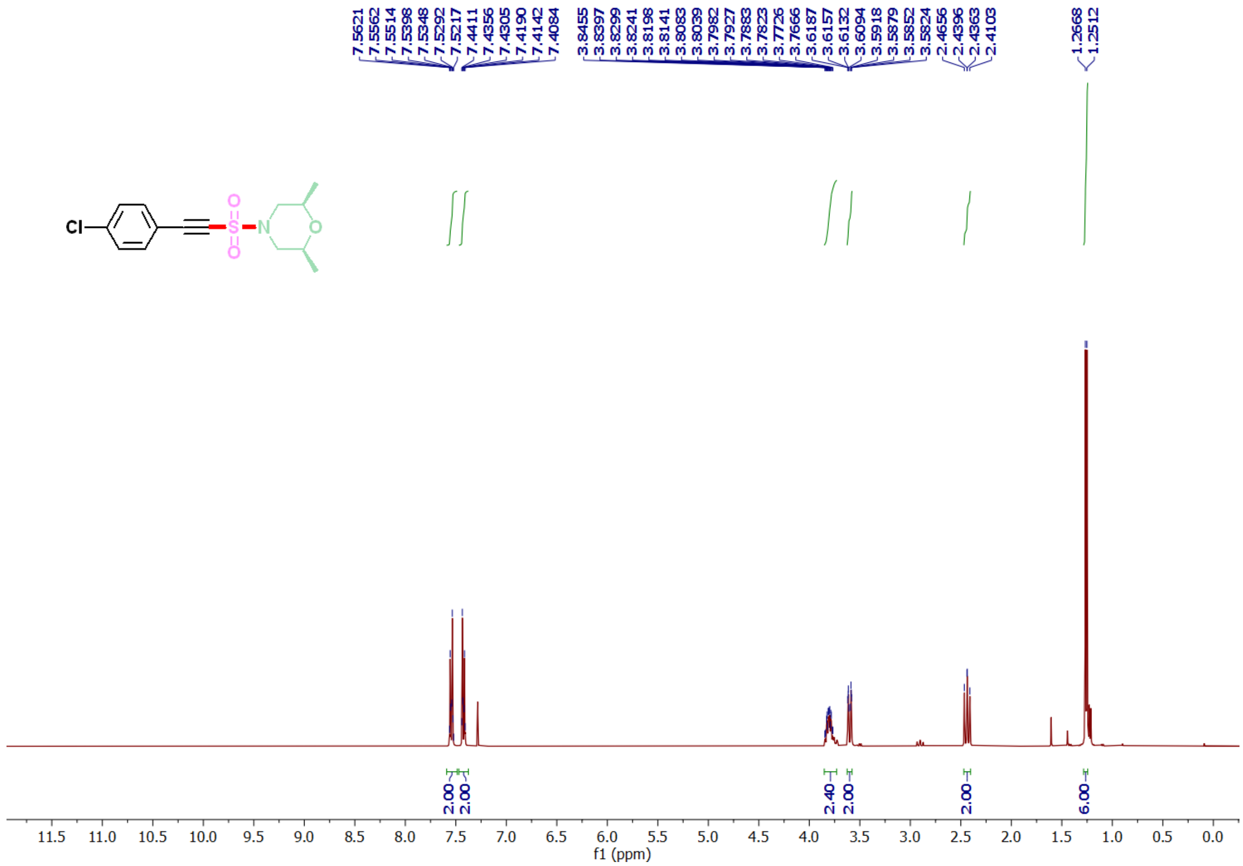

^{13}C NMR (101MHz, $CDCl_3$) of **3azf**

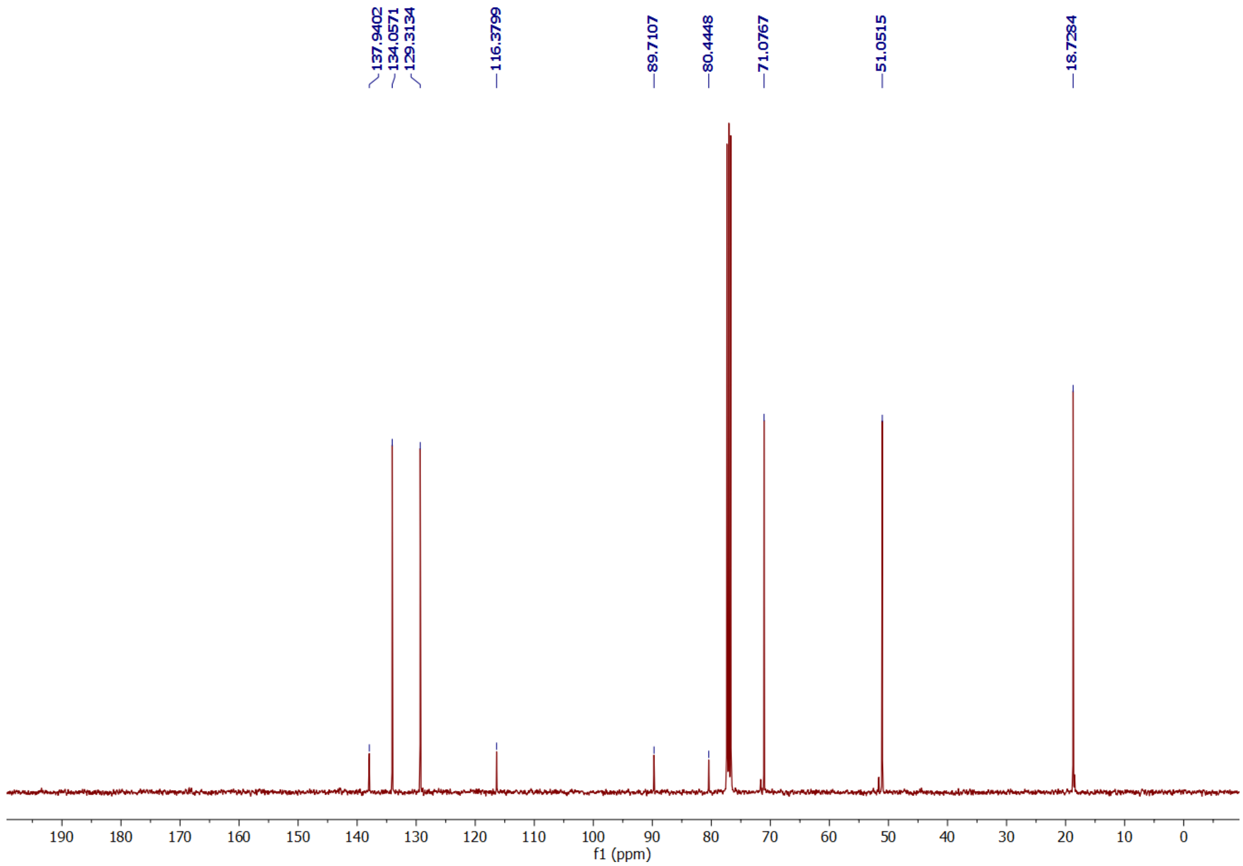

^{1}H NMR (400 MHz, $CDCl_3$) of **3azg** (see procedure)

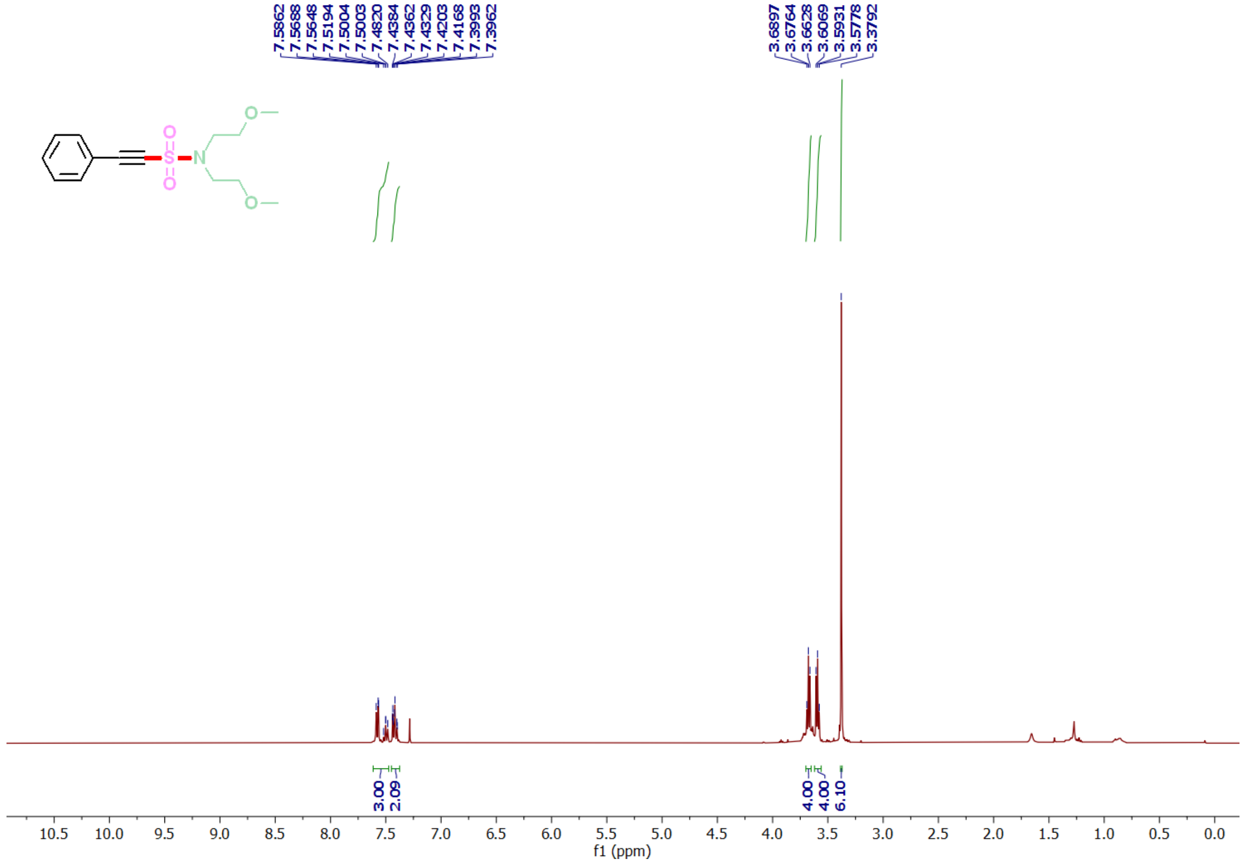

^{13}C NMR (101MHz, $CDCl_3$) of **3azg**

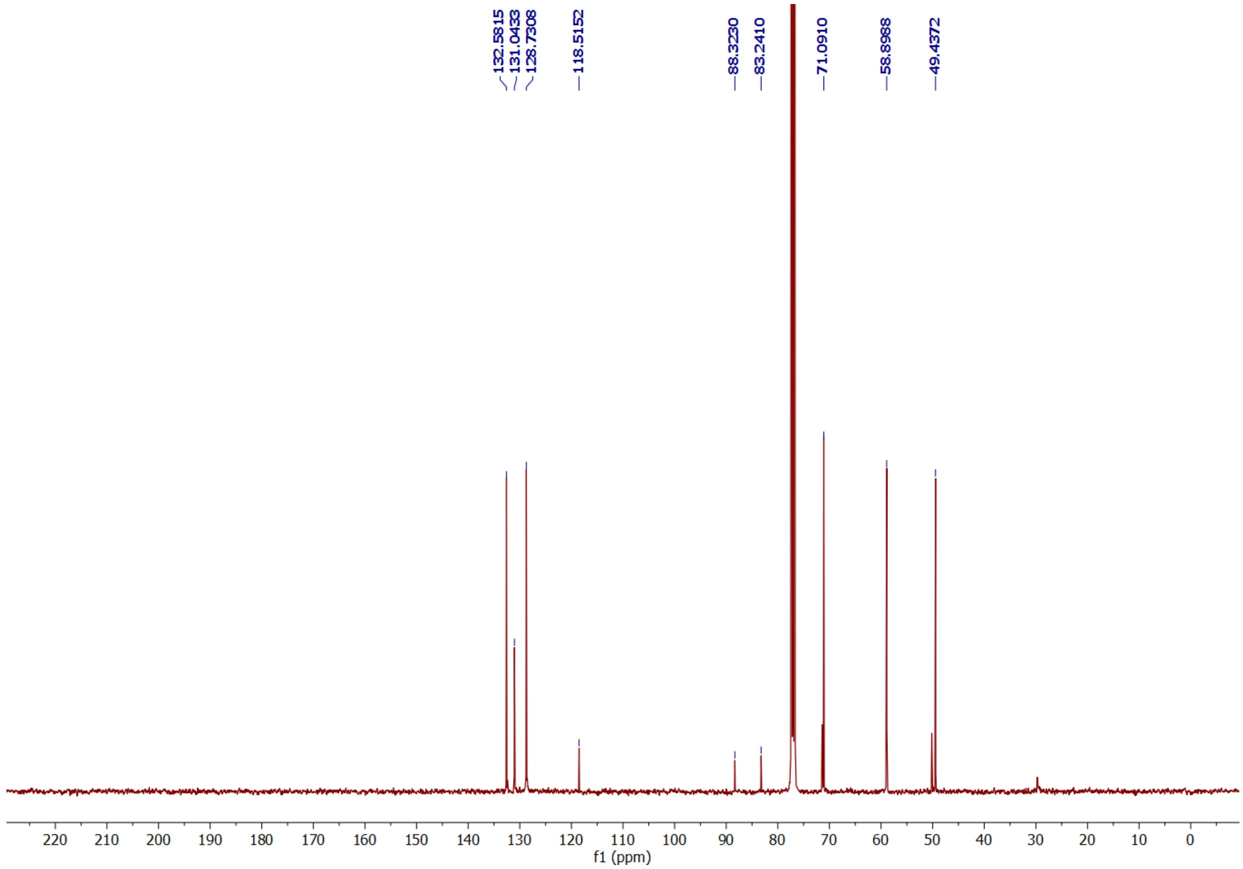

^{1}H NMR (400 MHz, $CDCl_3$) of **3ba** (see procedure)

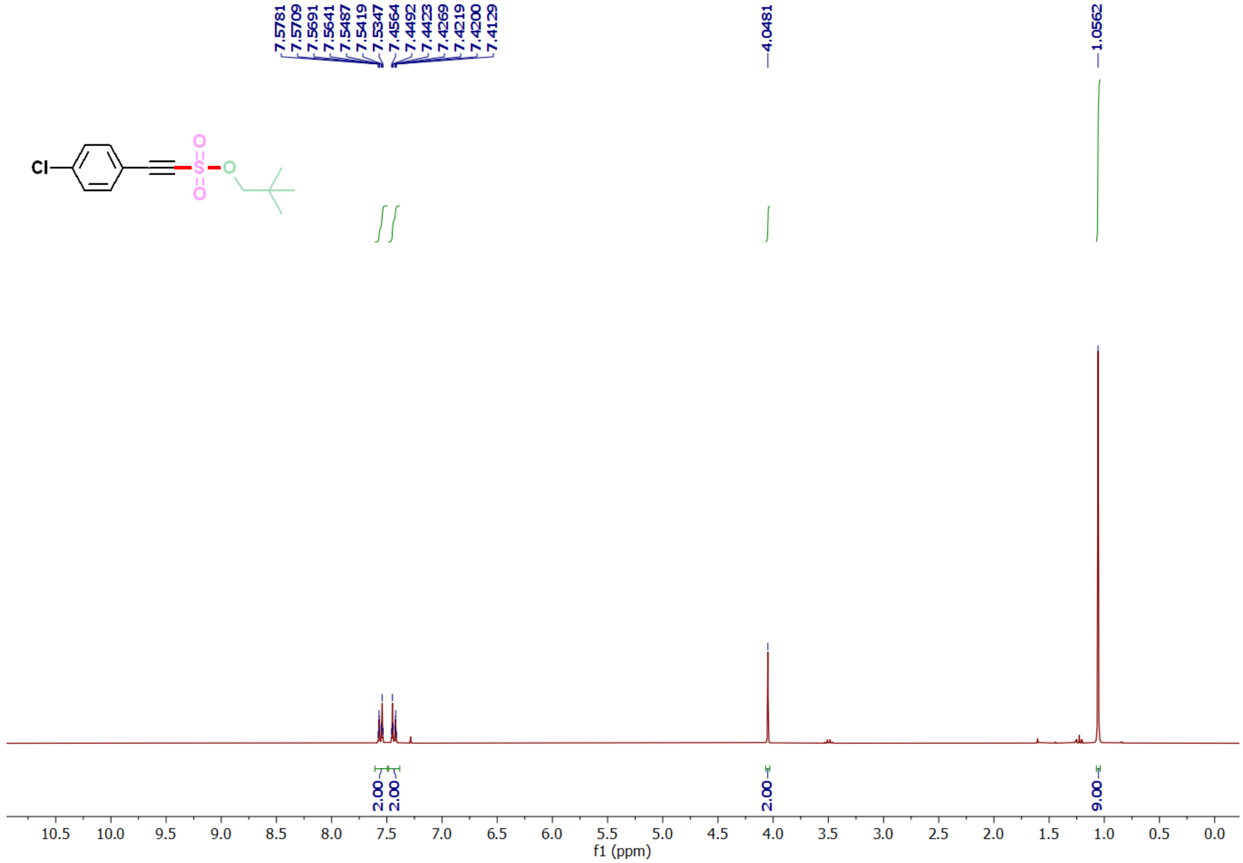

^{13}C NMR (101MHz, $CDCl_3$) of **3ba**

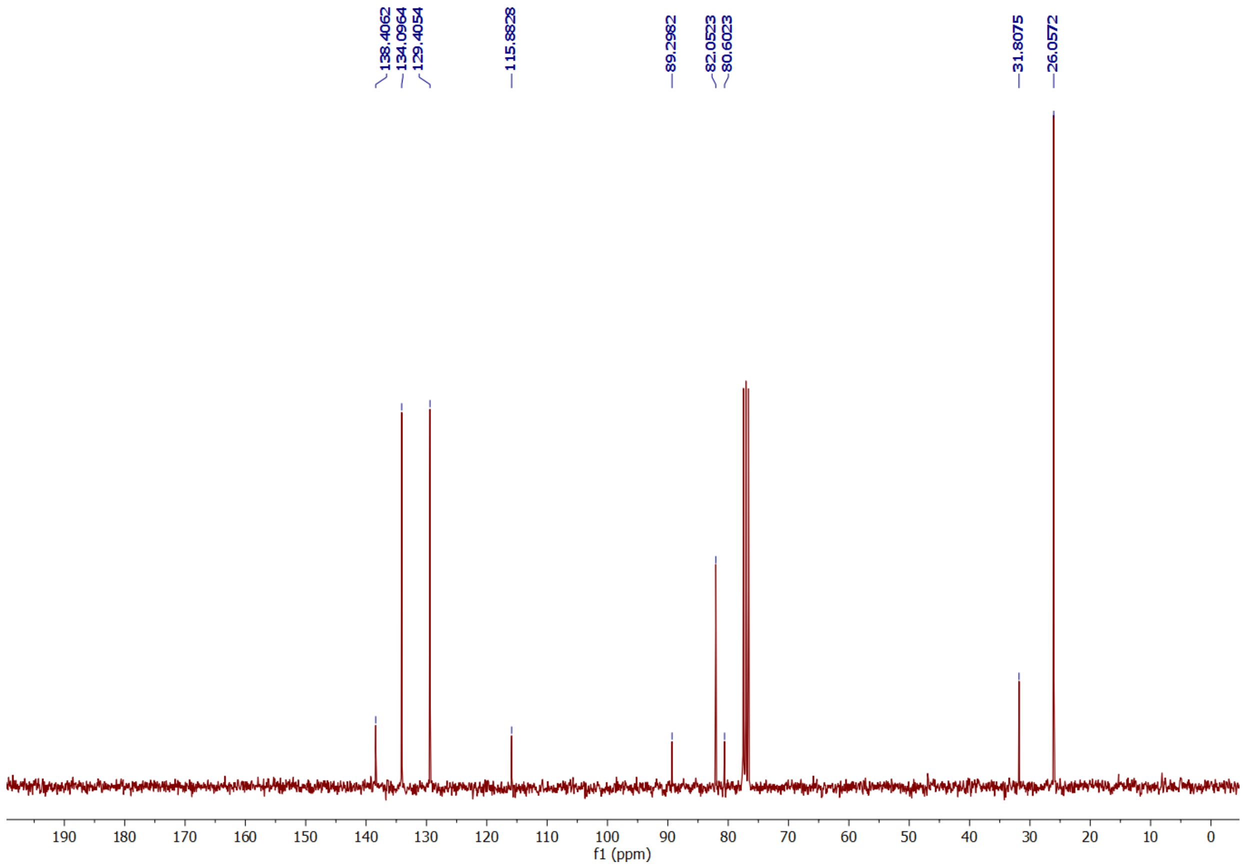

^{1}H NMR (400 MHz, $CDCl_3$) of **3ca** (see procedure)

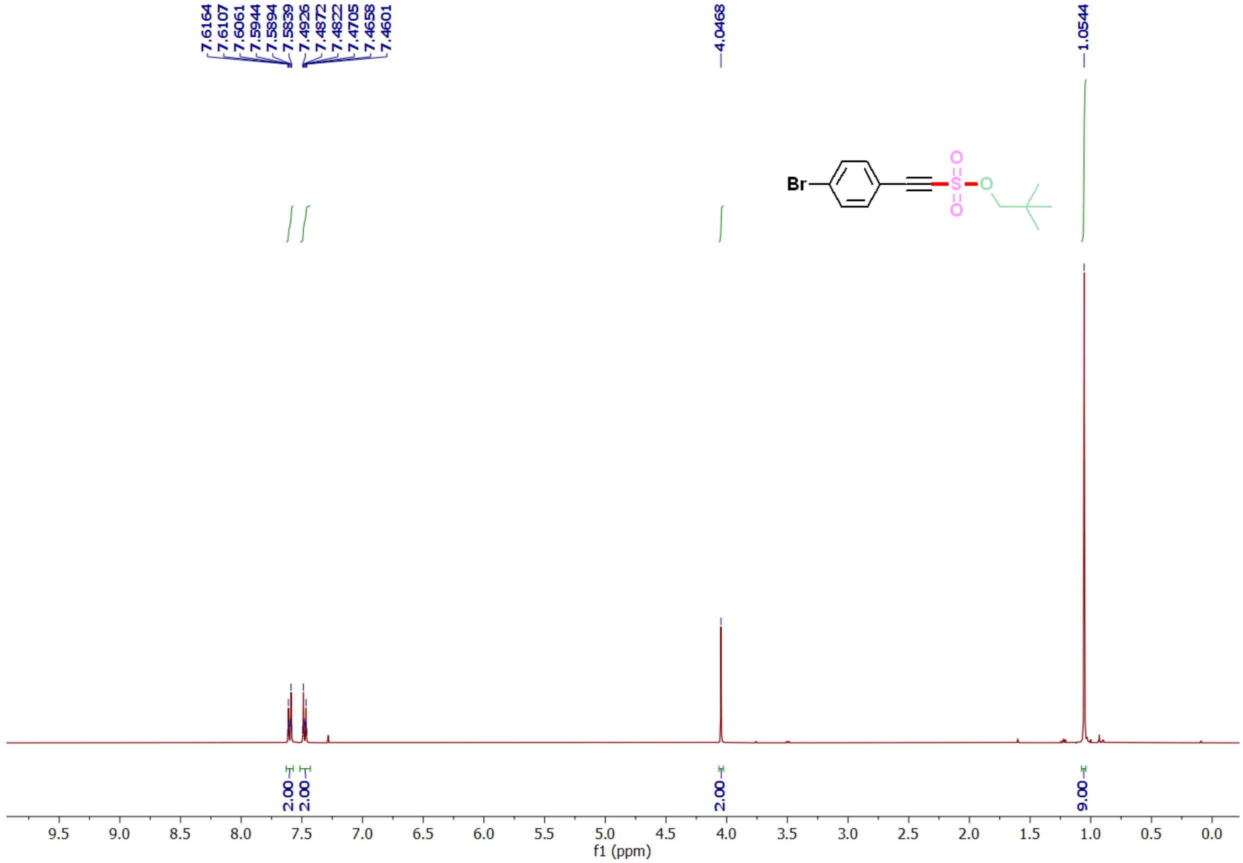

^{13}C NMR (101MHz, $CDCl_3$) of **3ca**

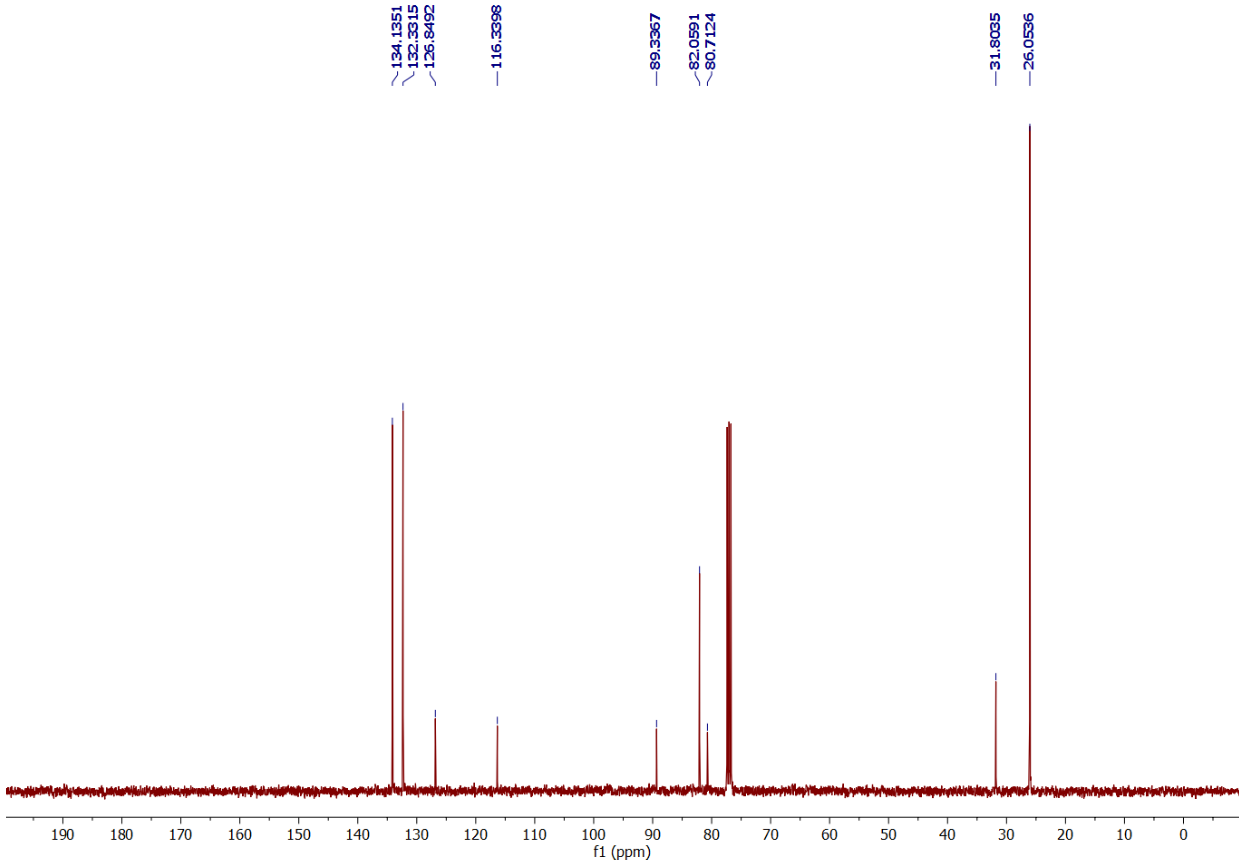

^{1}H NMR (400 MHz, $CDCl_3$) of **3da** (see procedure)

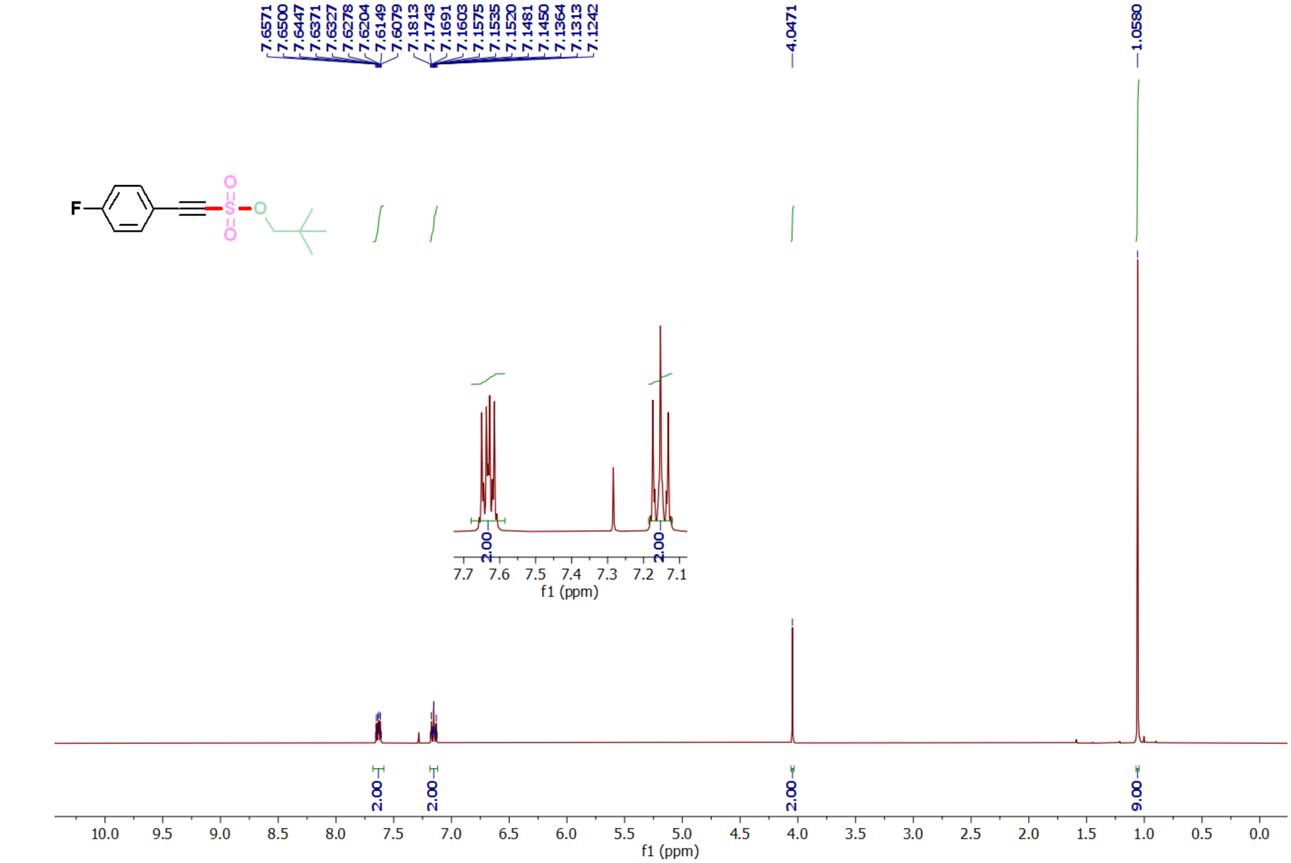

^{13}C NMR (101MHz, $CDCl_3$) of **3da**

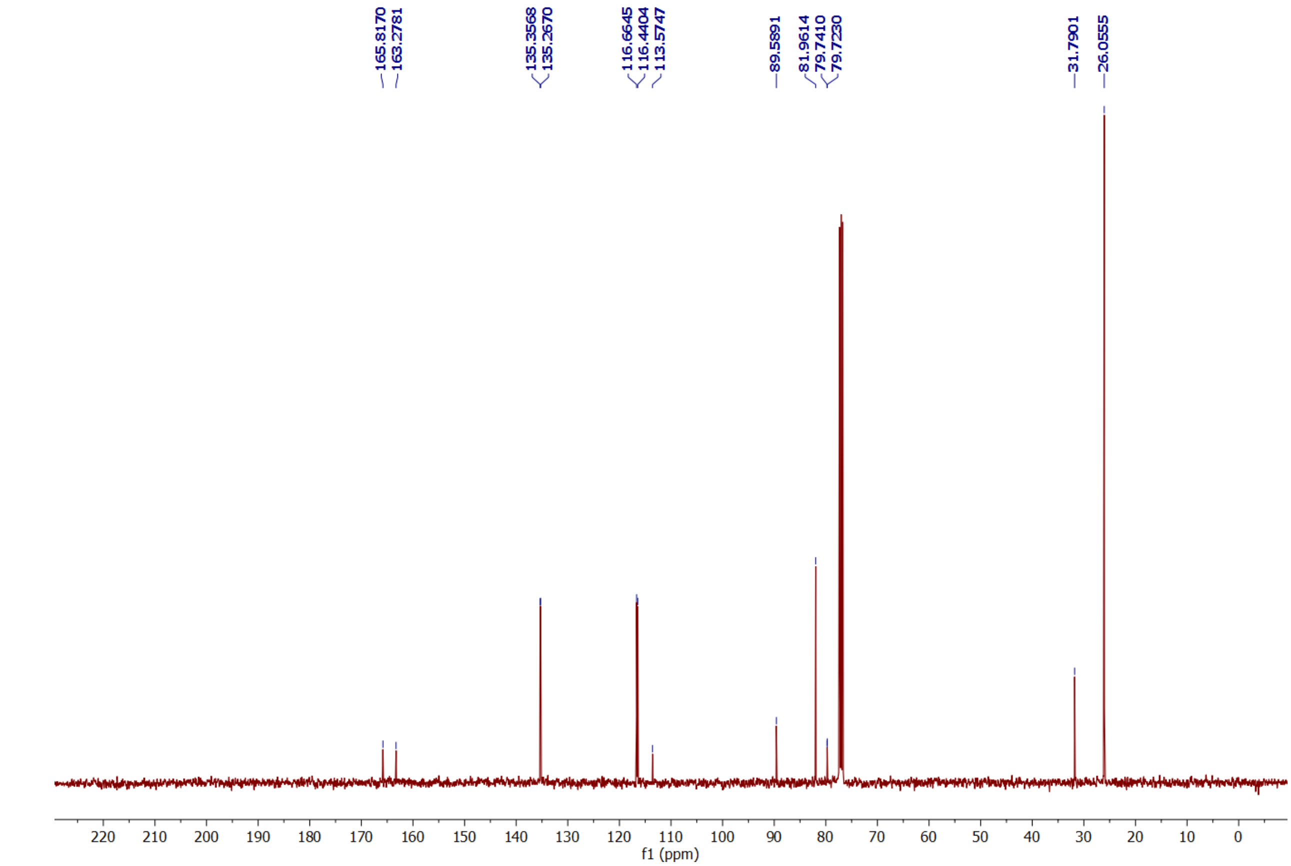

^{19}F NMR (282 MHz, $CDCl_3$) of **3da**

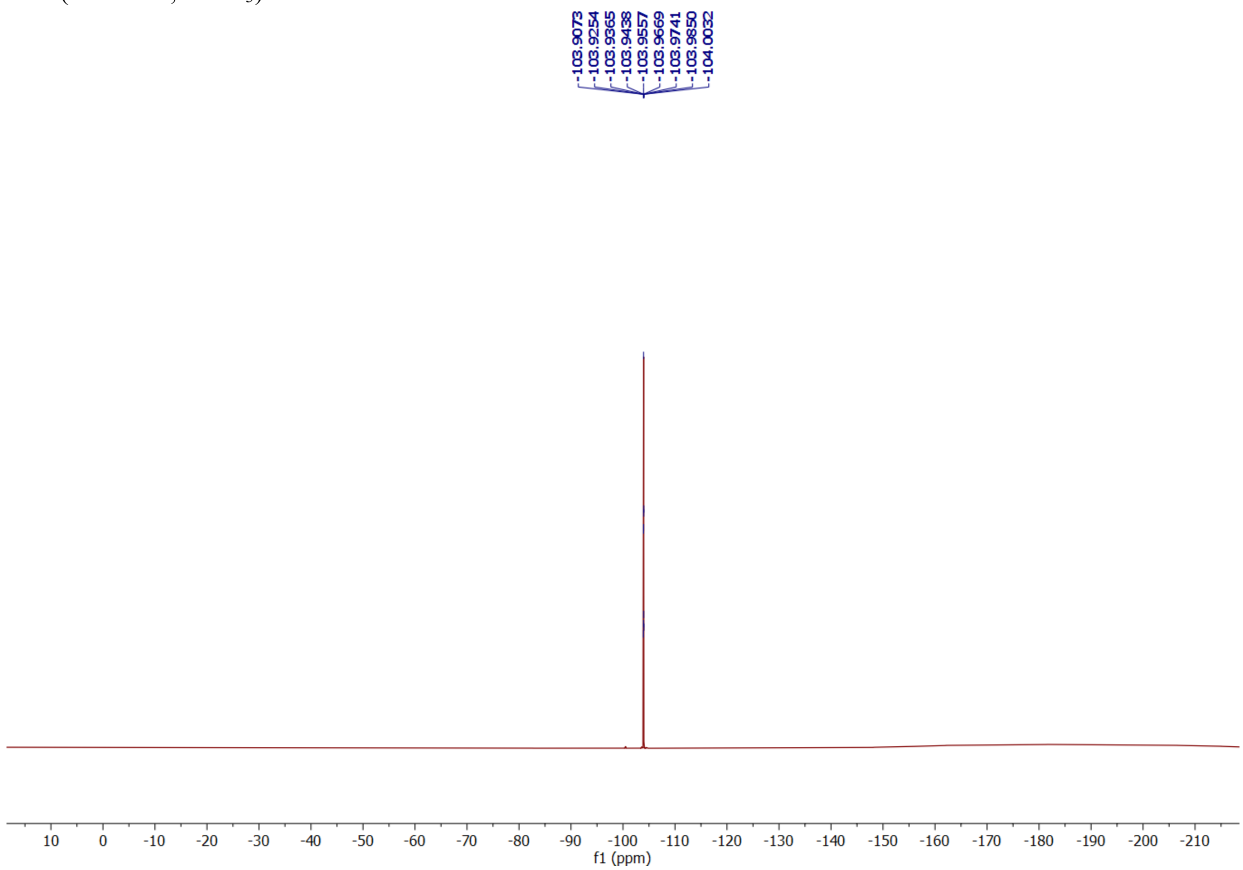

^{1}H NMR (400 MHz, $CDCl_3$) of **3ea** (see procedure)

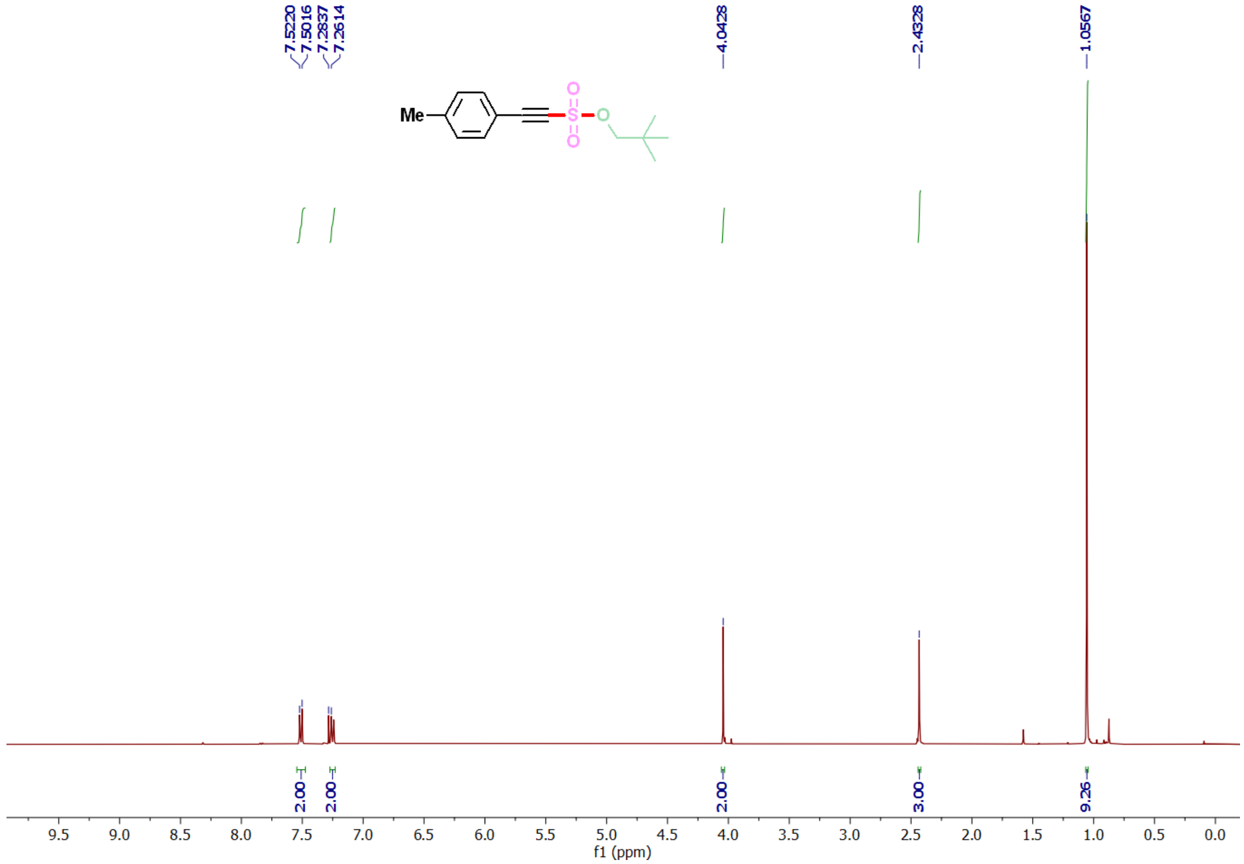

^{13}C NMR (101MHz, $CDCl_3$) of **3ea**

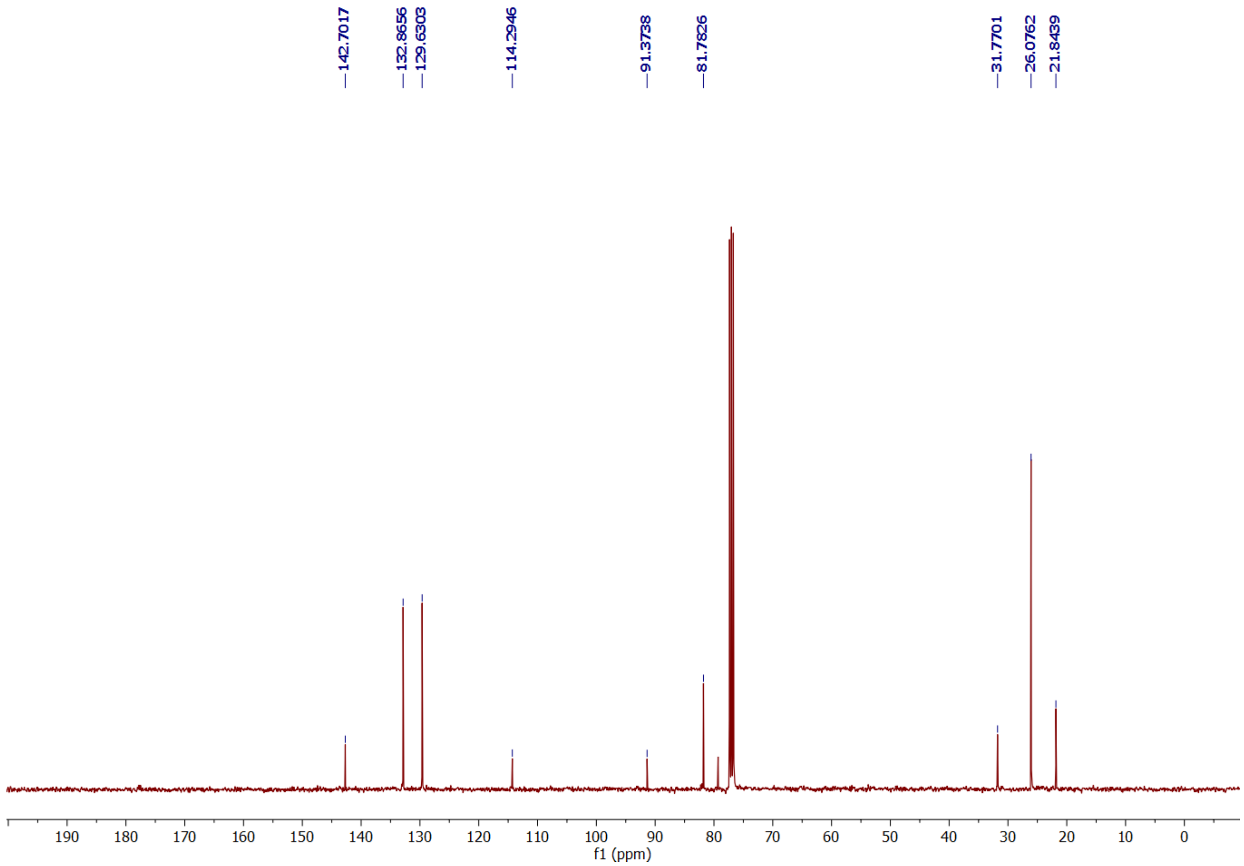

^{1}H NMR (400 MHz, $CDCl_3$) of **3fa** (see procedure)

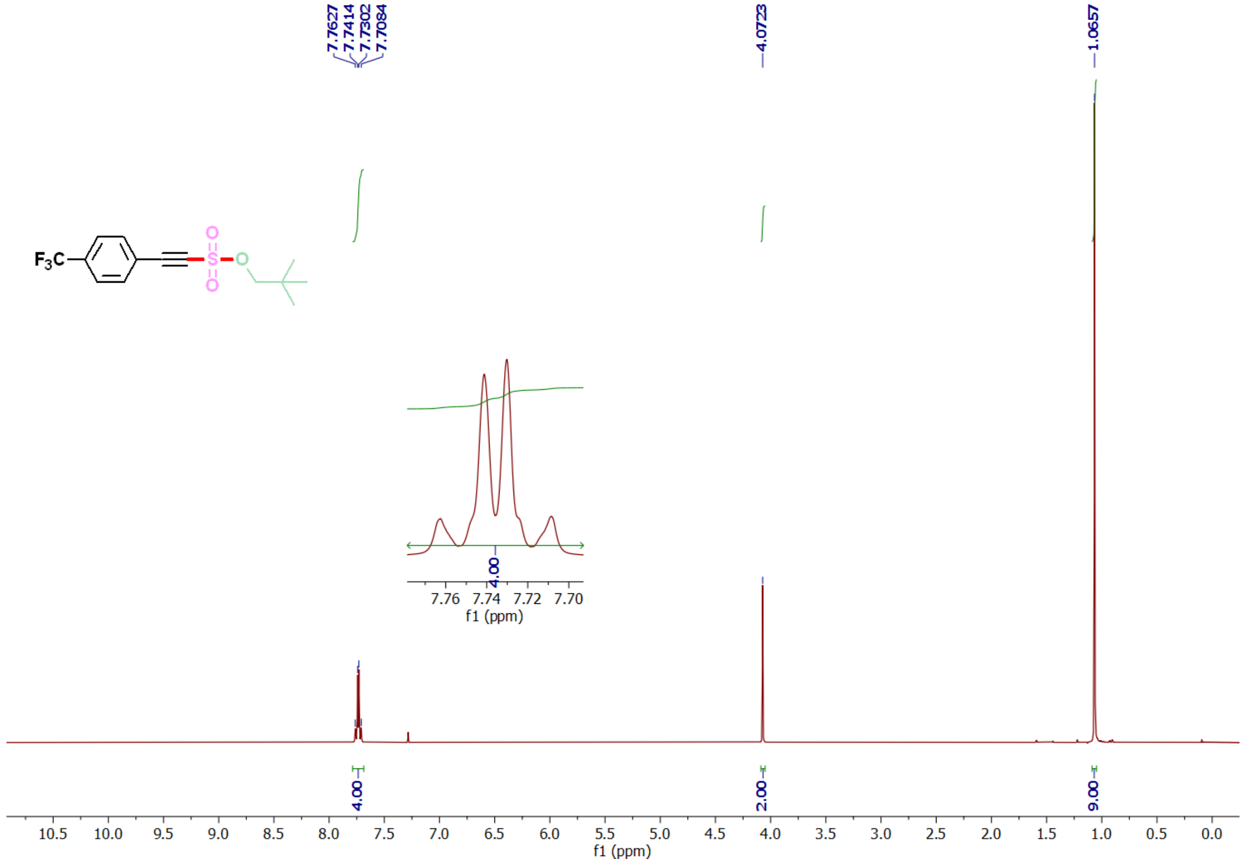

^{13}C NMR (101MHz, $CDCl_3$) of **3fa**

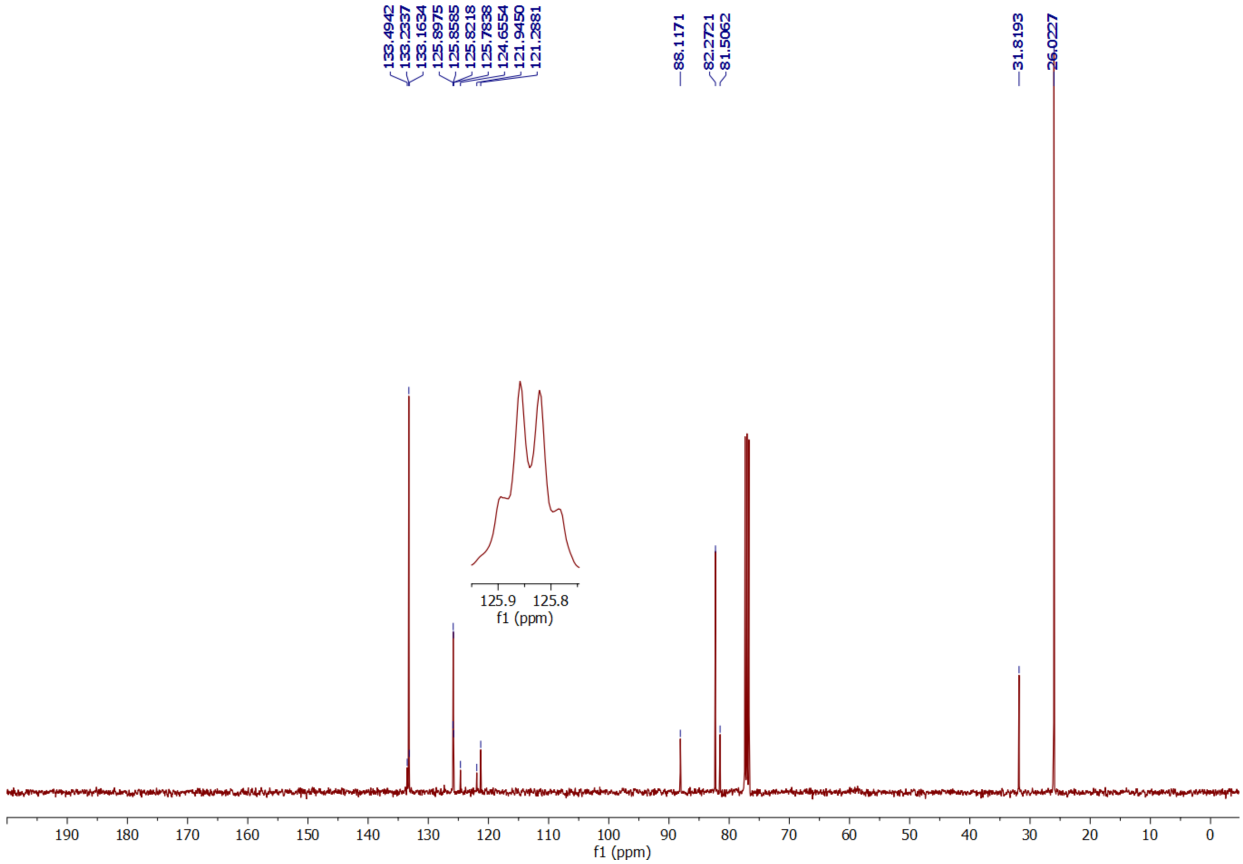

^{19}F NMR (282 MHz, $CDCl_3$) of **3fa**

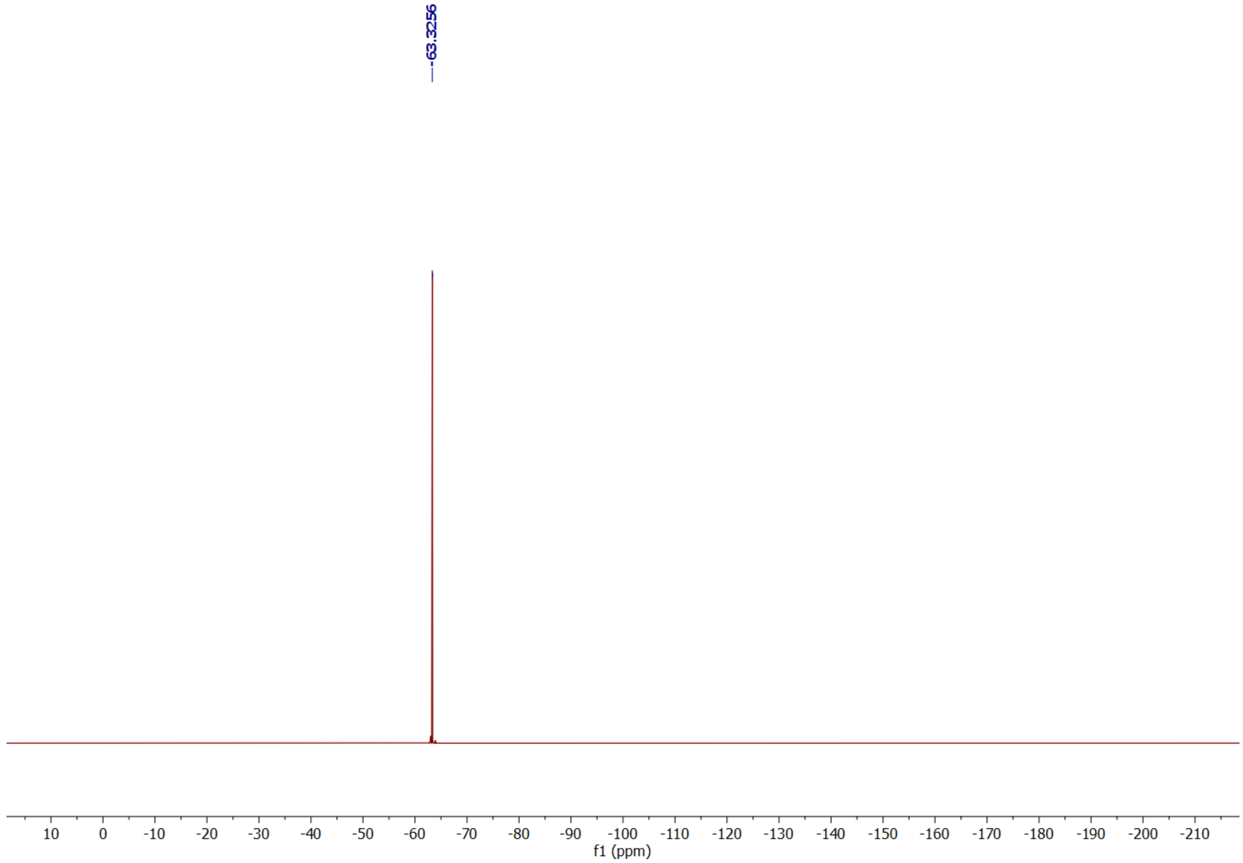

^{1}H NMR (400 MHz, $CDCl_3$) of **3ga** (see procedure)

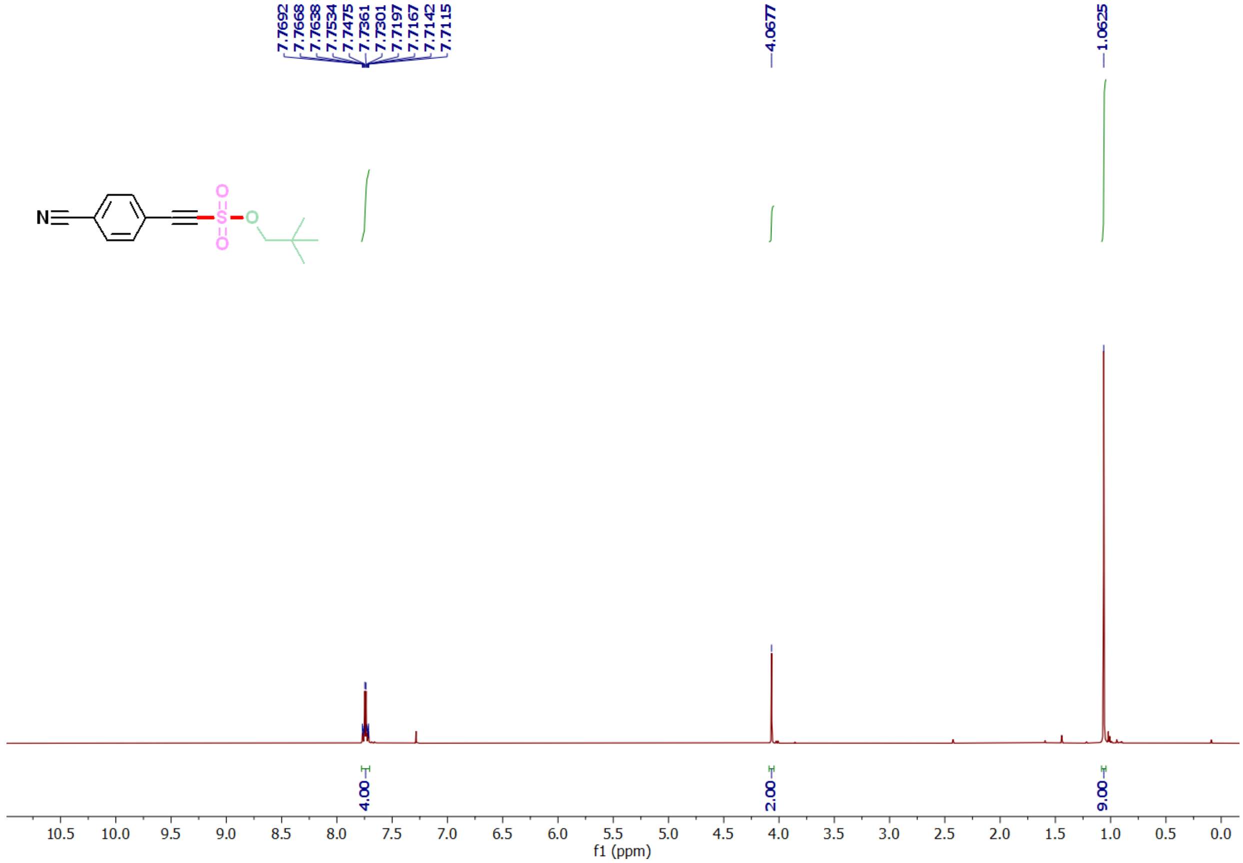

^{13}C NMR (101MHz, $CDCl_3$) of **3ga**

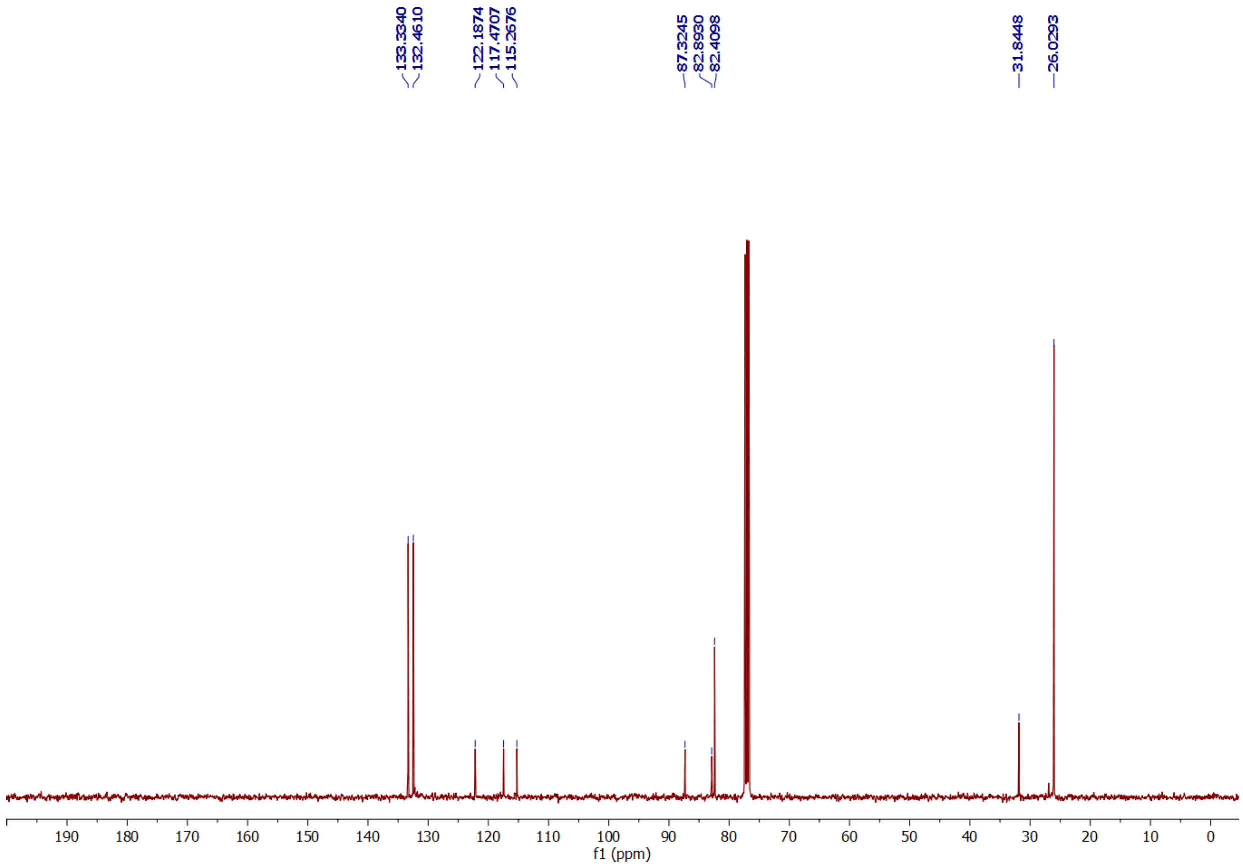

^{1}H NMR (400 MHz, $CDCl_3$) of **3ha** (see procedure)

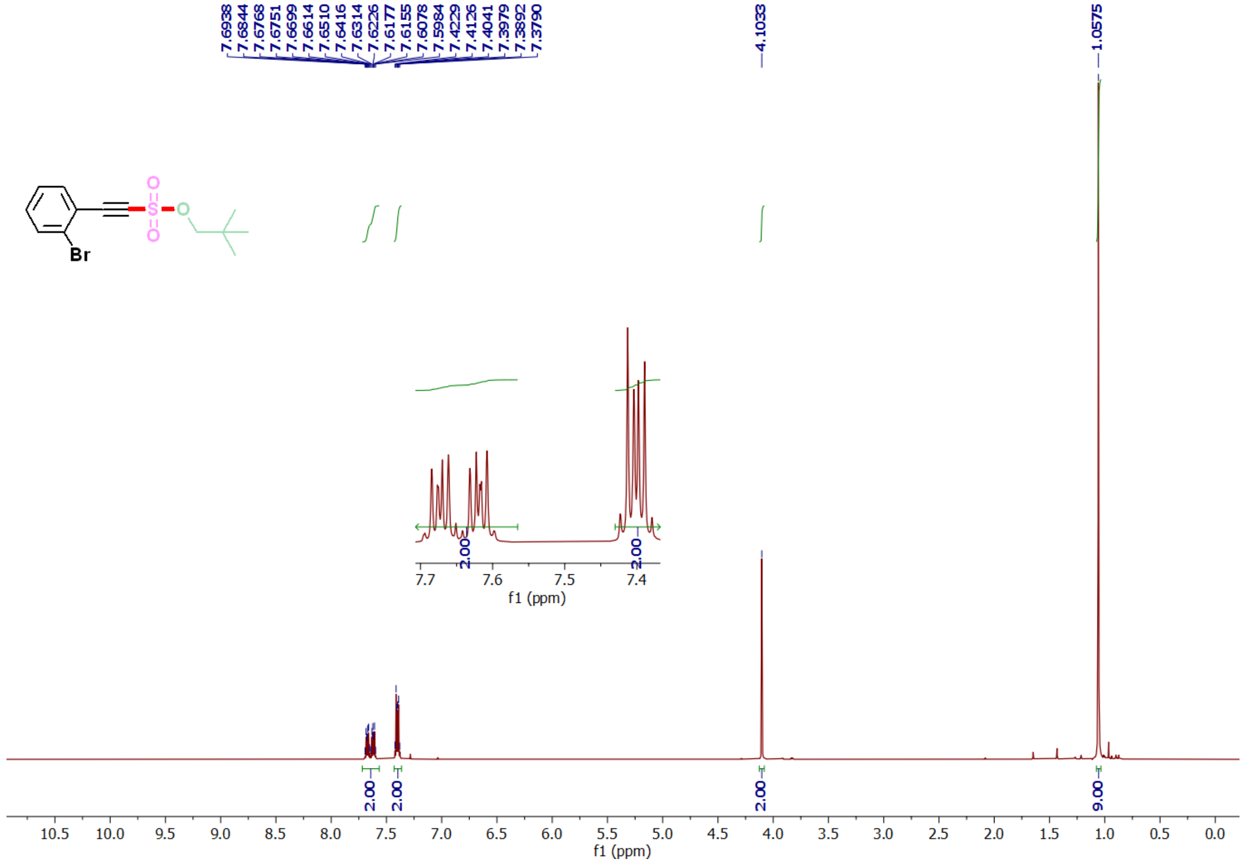

^{13}C NMR (101MHz, $CDCl_3$) of **3ha**

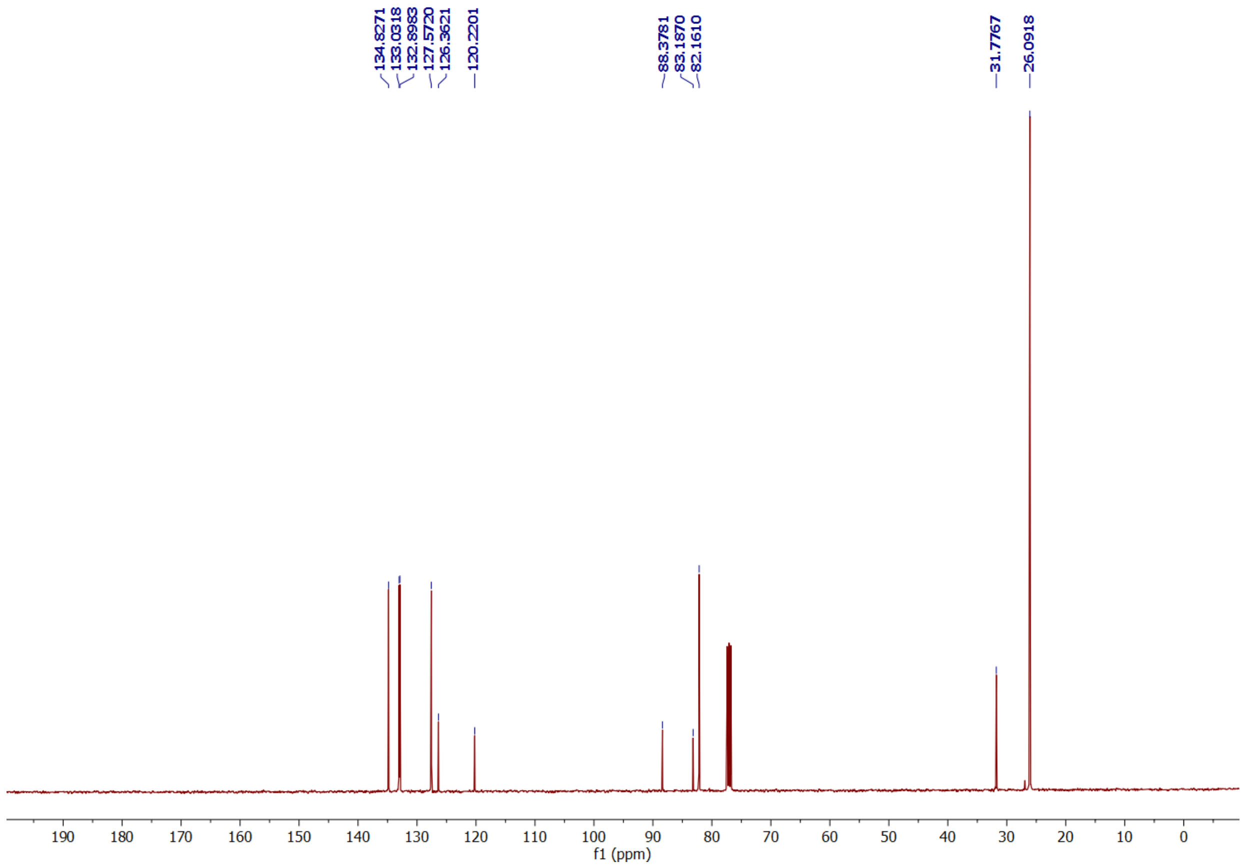

^{1}H NMR (400 MHz, $CDCl_3$) of **3ia** (see procedure)

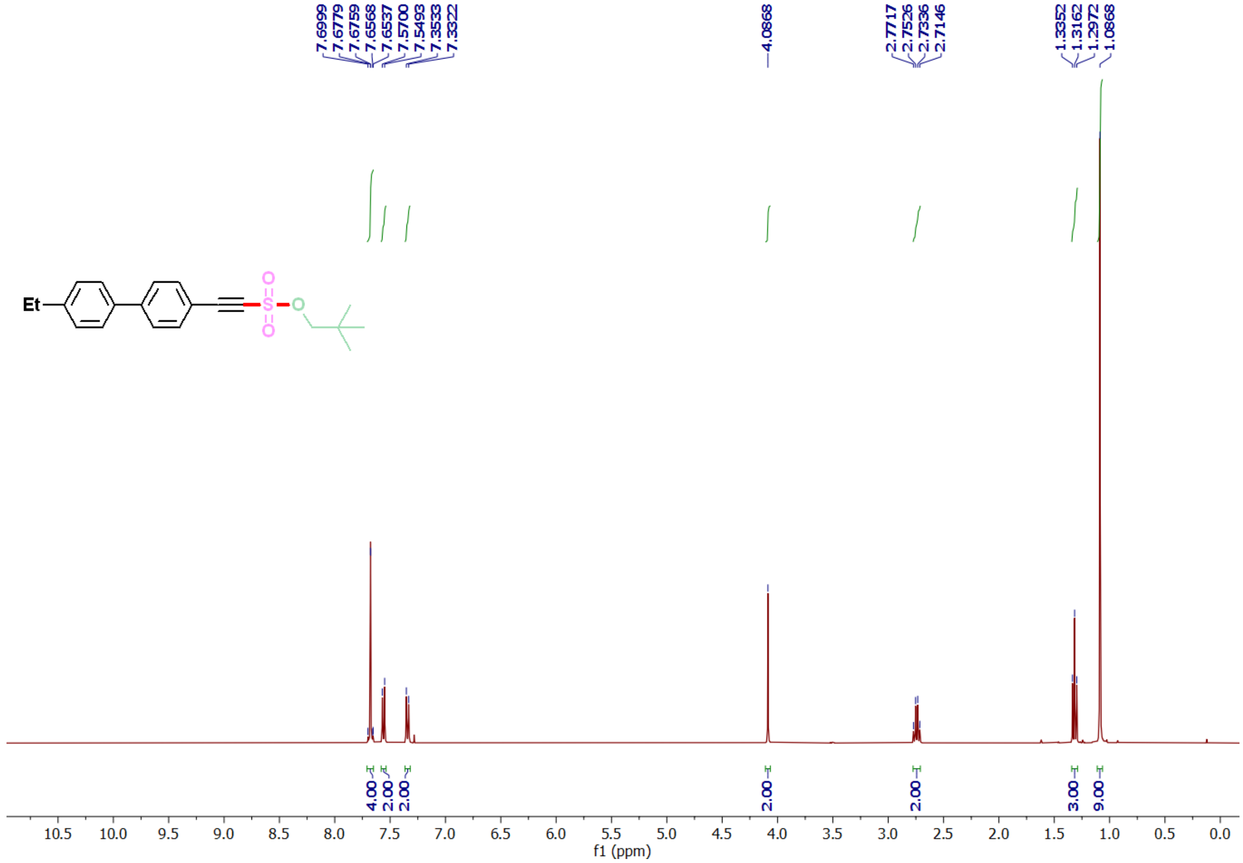

^{13}C NMR (101MHz, $CDCl_3$) of **3ia**

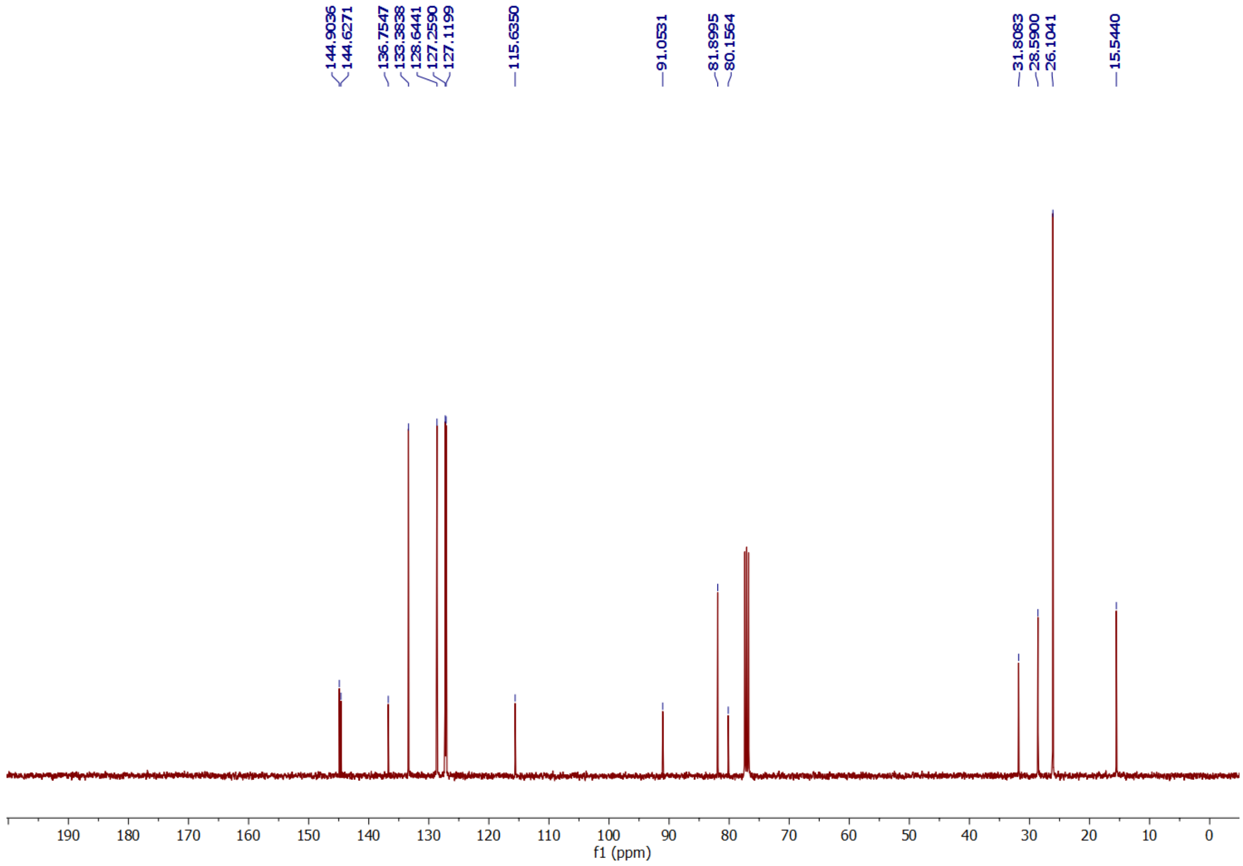

^{1}H NMR (400 MHz, $CDCl_3$) of **3ja** (see procedure)

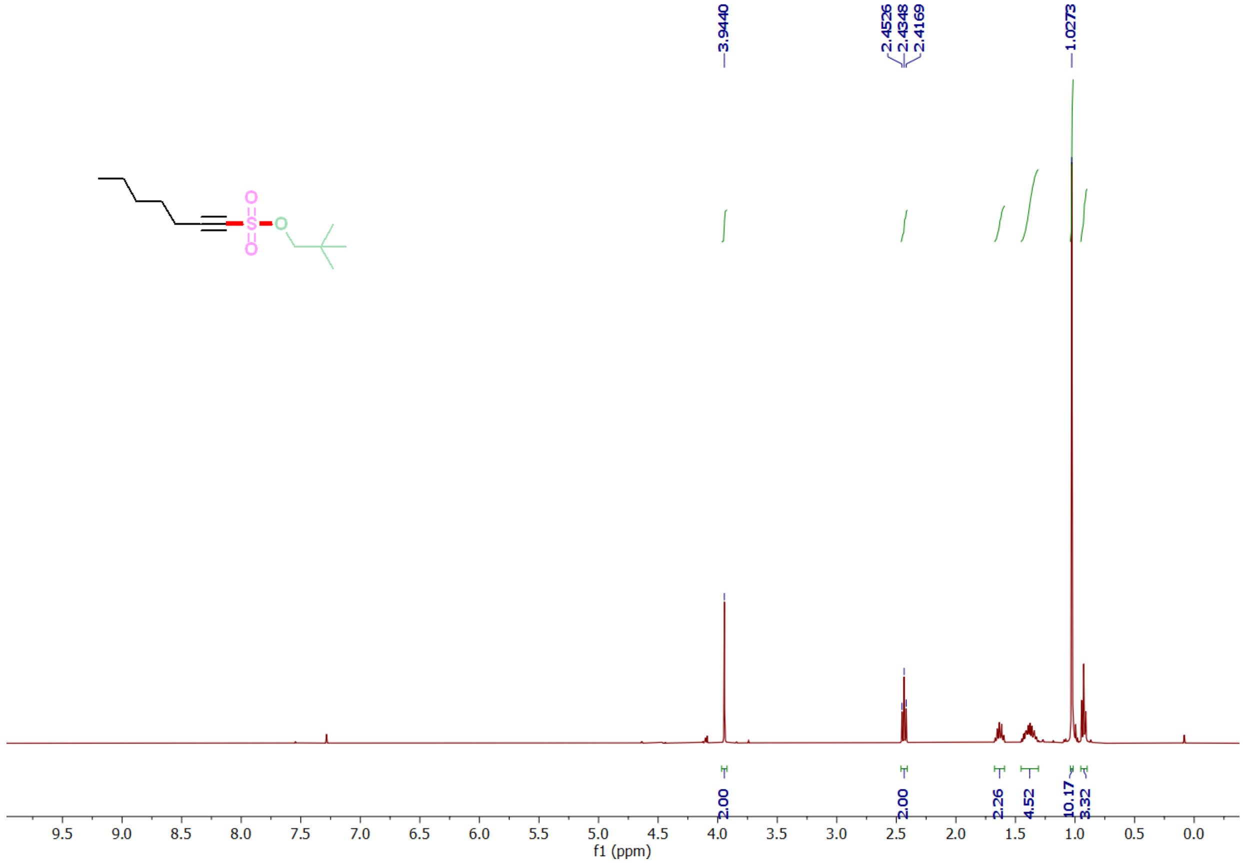

^{13}C NMR (101MHz, $CDCl_3$) of **3ja**

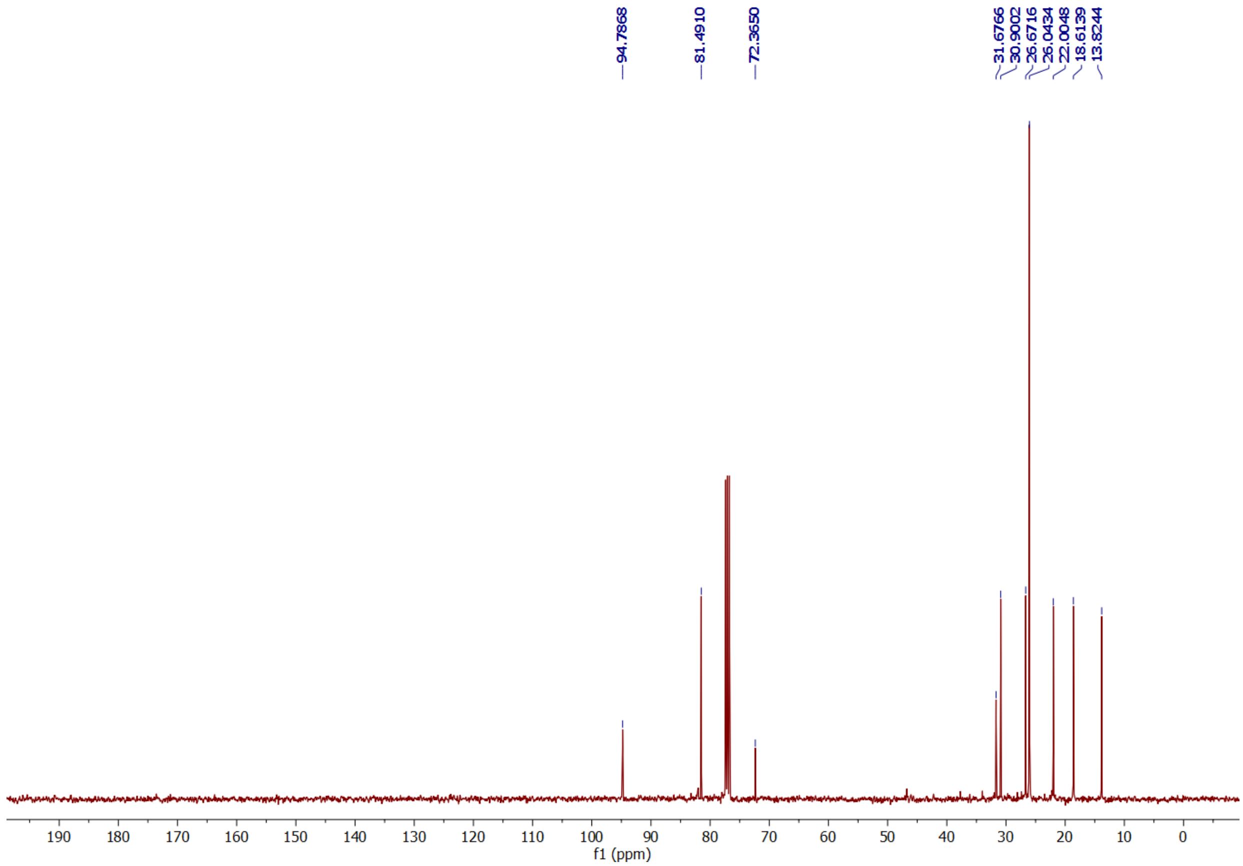

^{1}H NMR (400 MHz, $CDCl_3$) of **3ka** (see procedure)

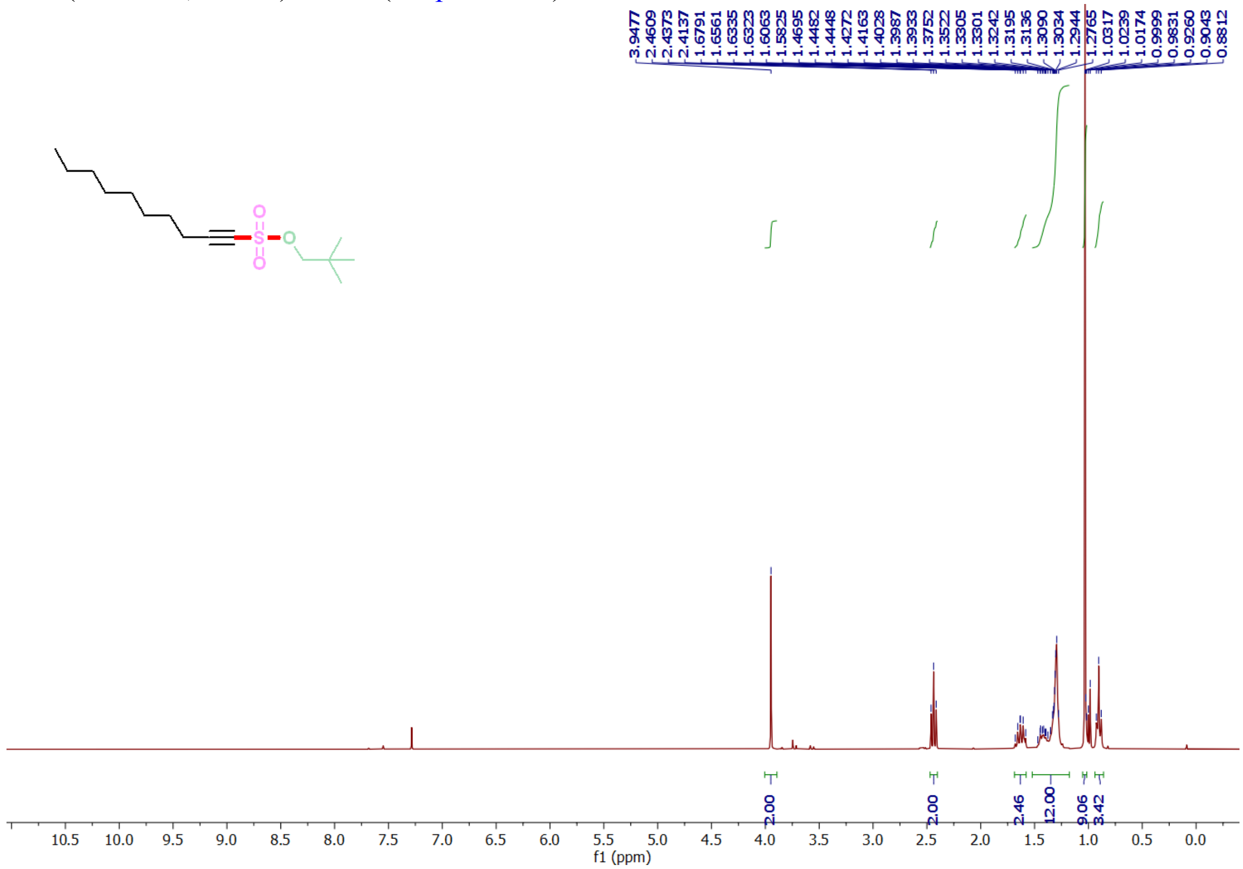

^{13}C NMR (101MHz, $CDCl_3$) of **3ka**

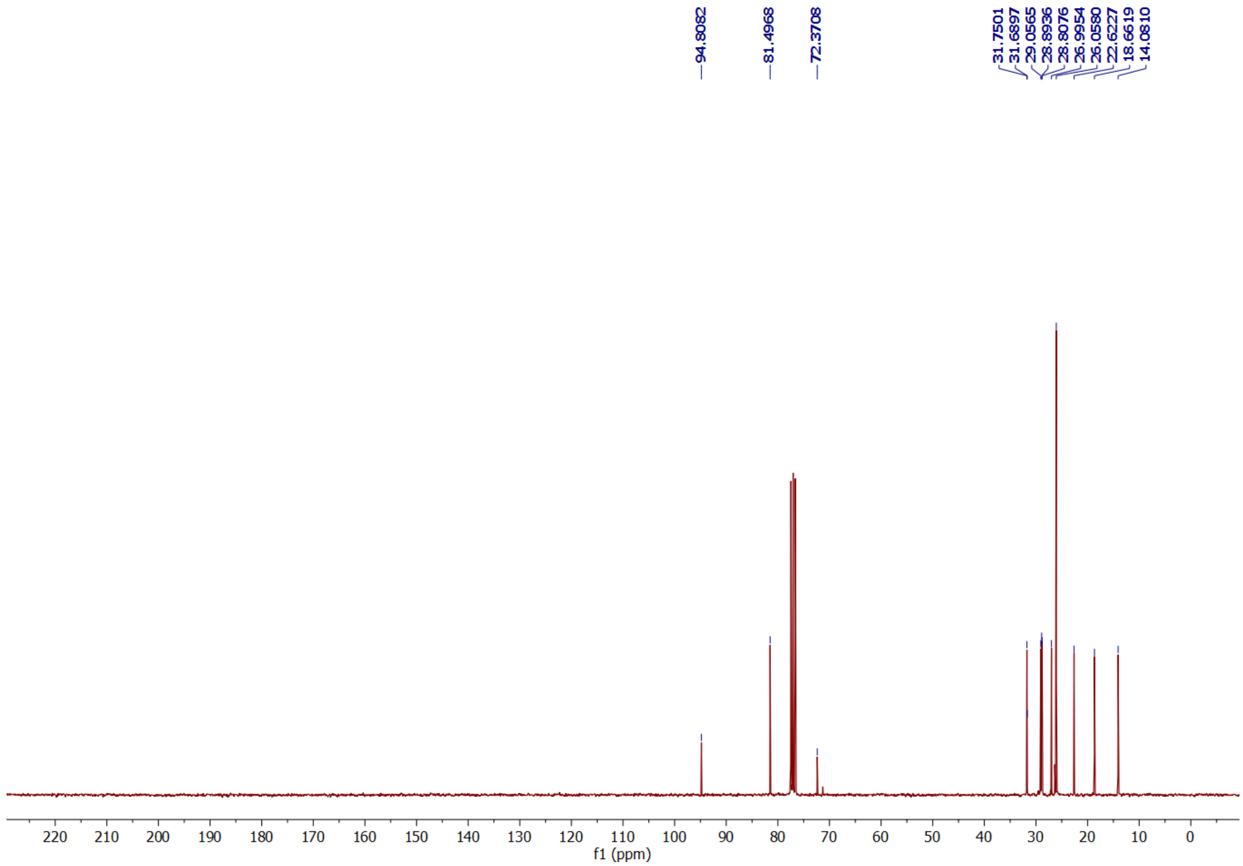

^{1}H NMR (300 MHz, $CDCl_3$) of **3la** (see procedure)

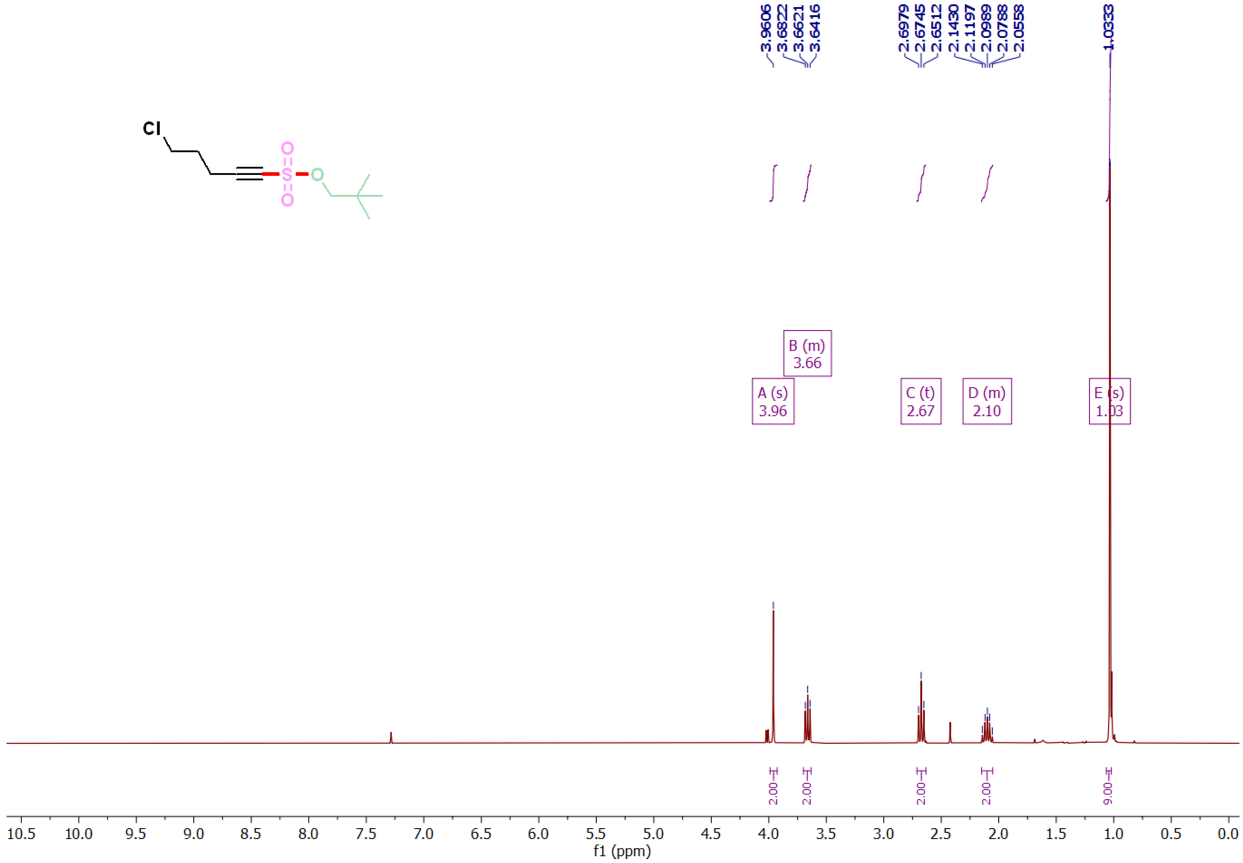

^{13}C NMR (75MHz, $CDCl_3$) of **3la**

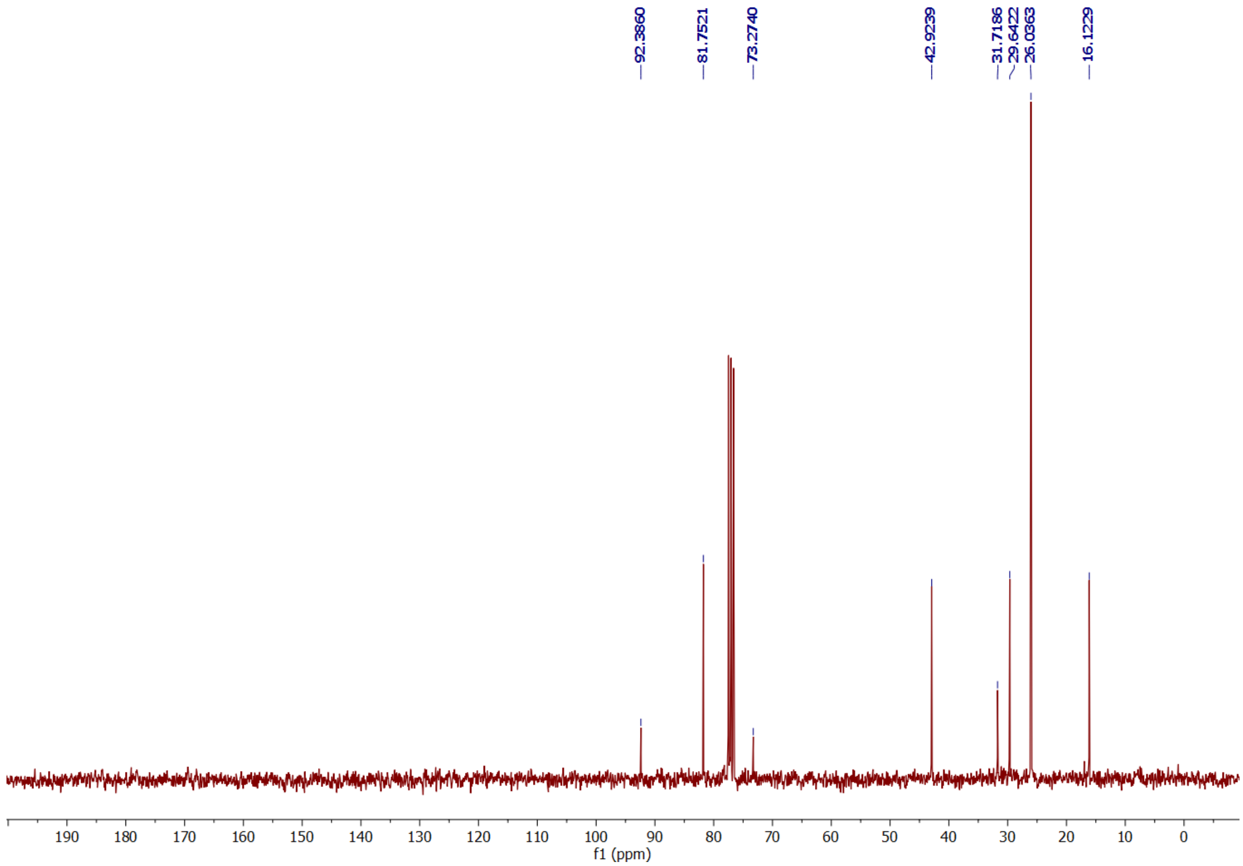

^{1}H NMR (300 MHz, $CDCl_3$) of **3ma** (see procedure)

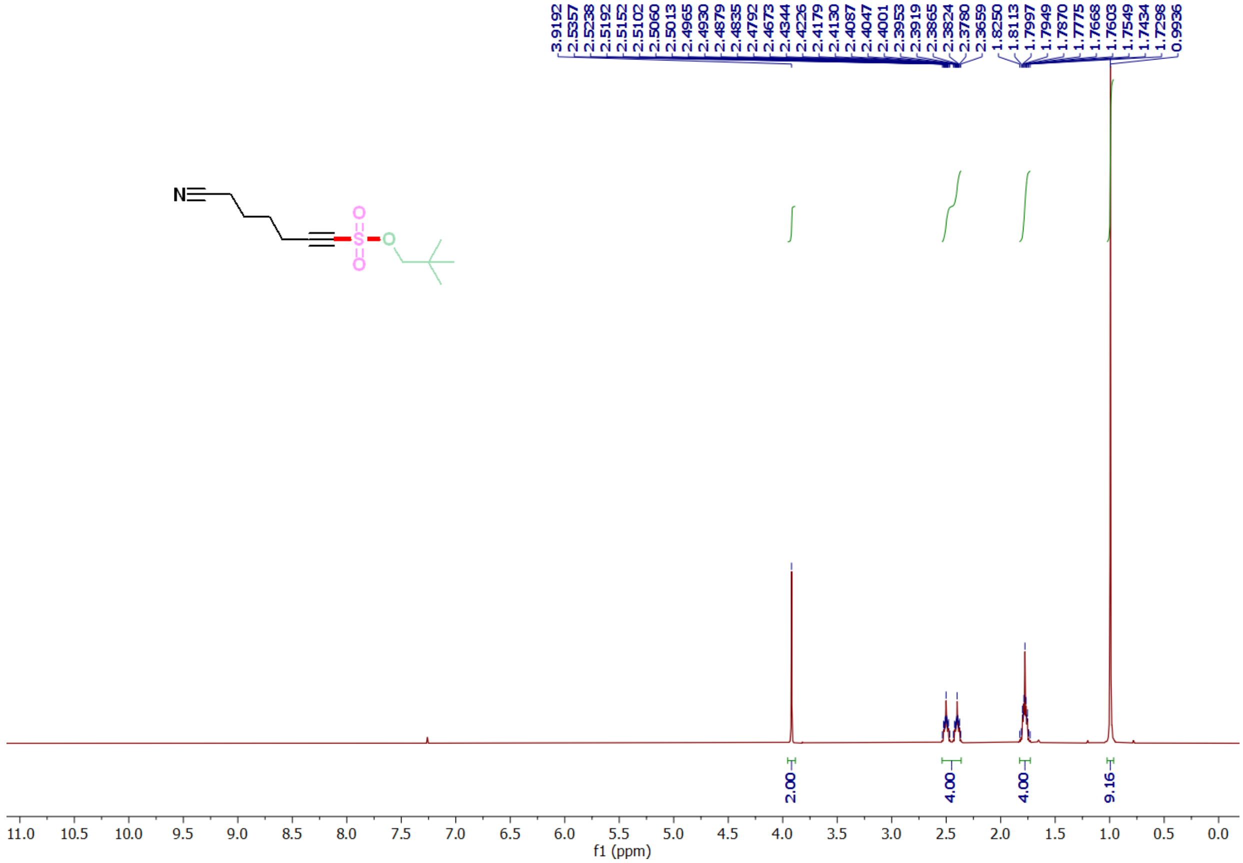

^{13}C NMR (75MHz, $CDCl_3$) of **3ma**

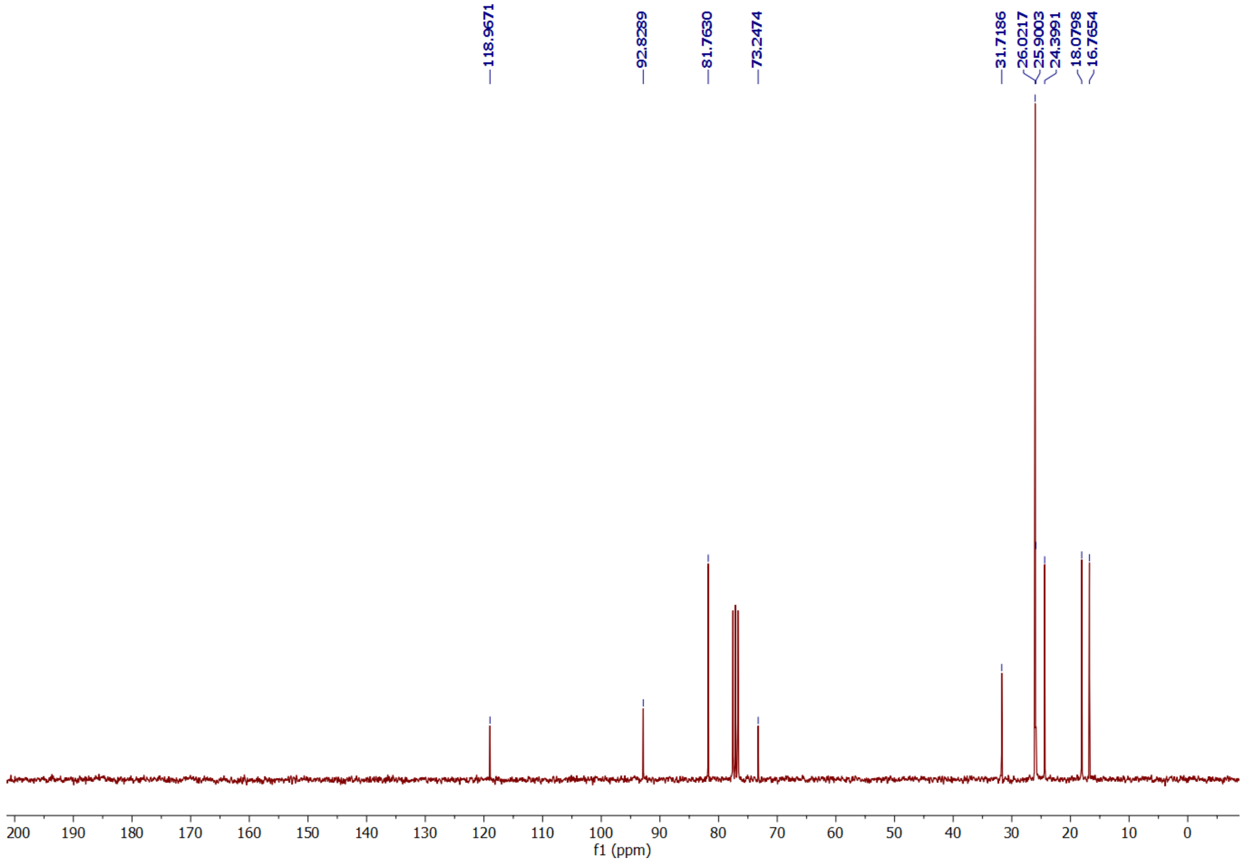

^{1}H NMR (400 MHz, $CDCl_3$) of **3na** (see procedure)

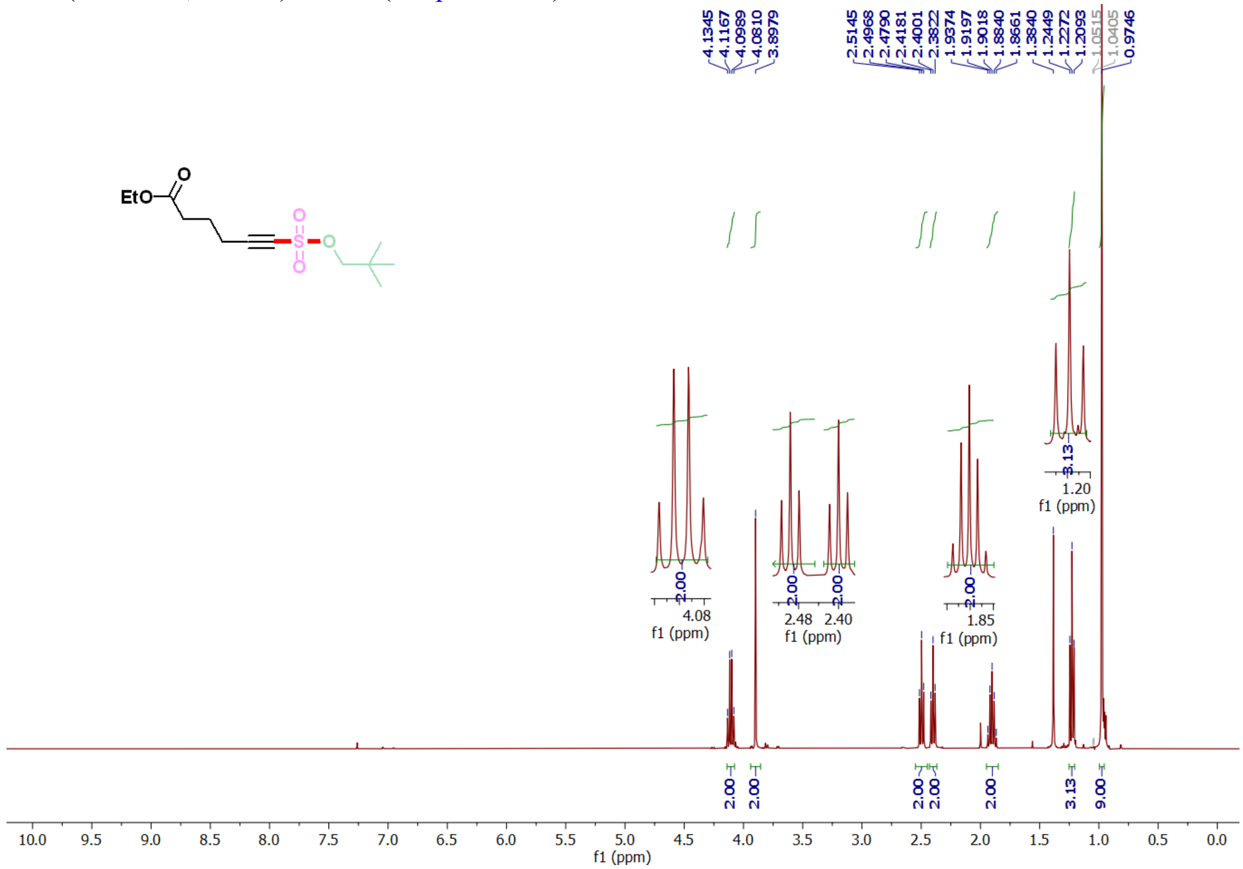

^{13}C NMR (101MHz, $CDCl_3$) of **3na**

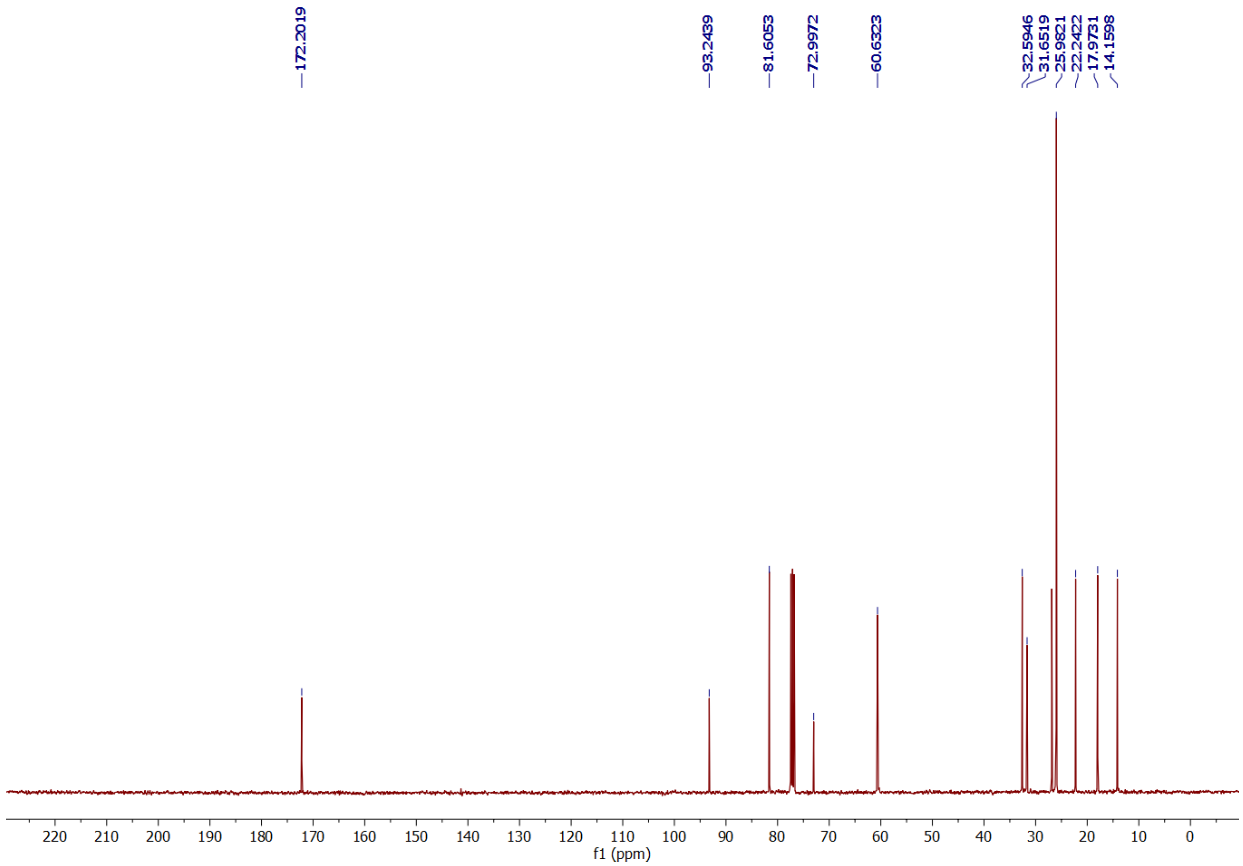

^{1}H NMR (400 MHz, $CDCl_3$) of **3oa** (see procedure)

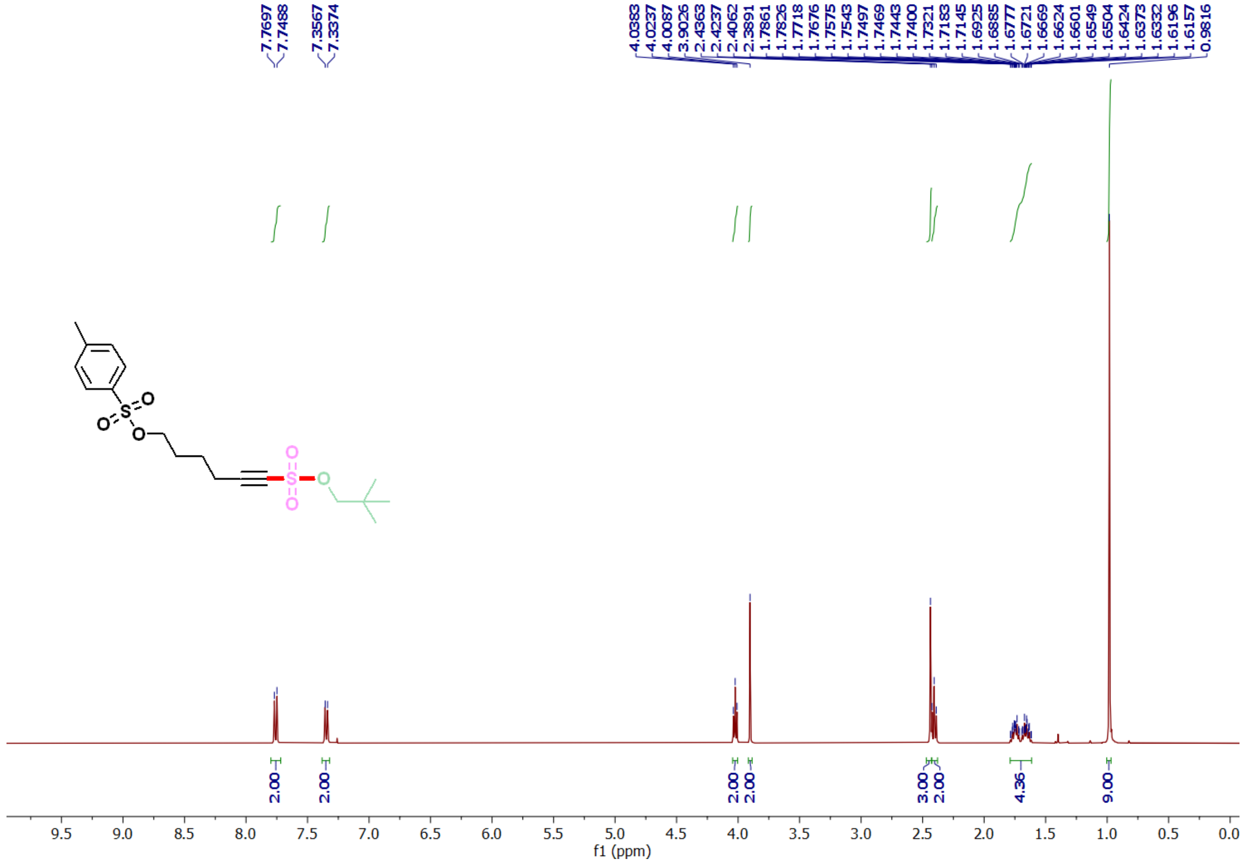

^{13}C NMR (75MHz, $CDCl_3$) of **3oa**

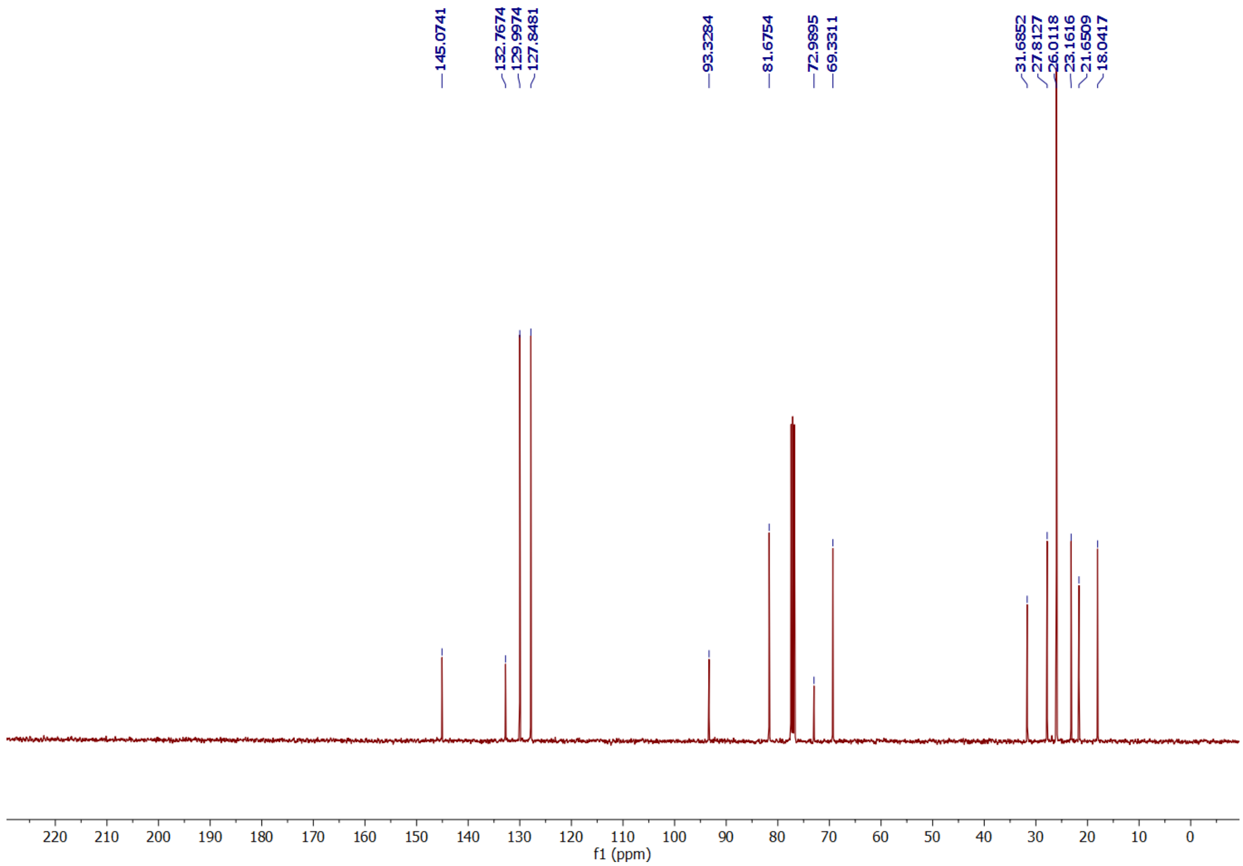

^{1}H NMR (300 MHz, $CDCl_3$) of **3pa** (see procedure)

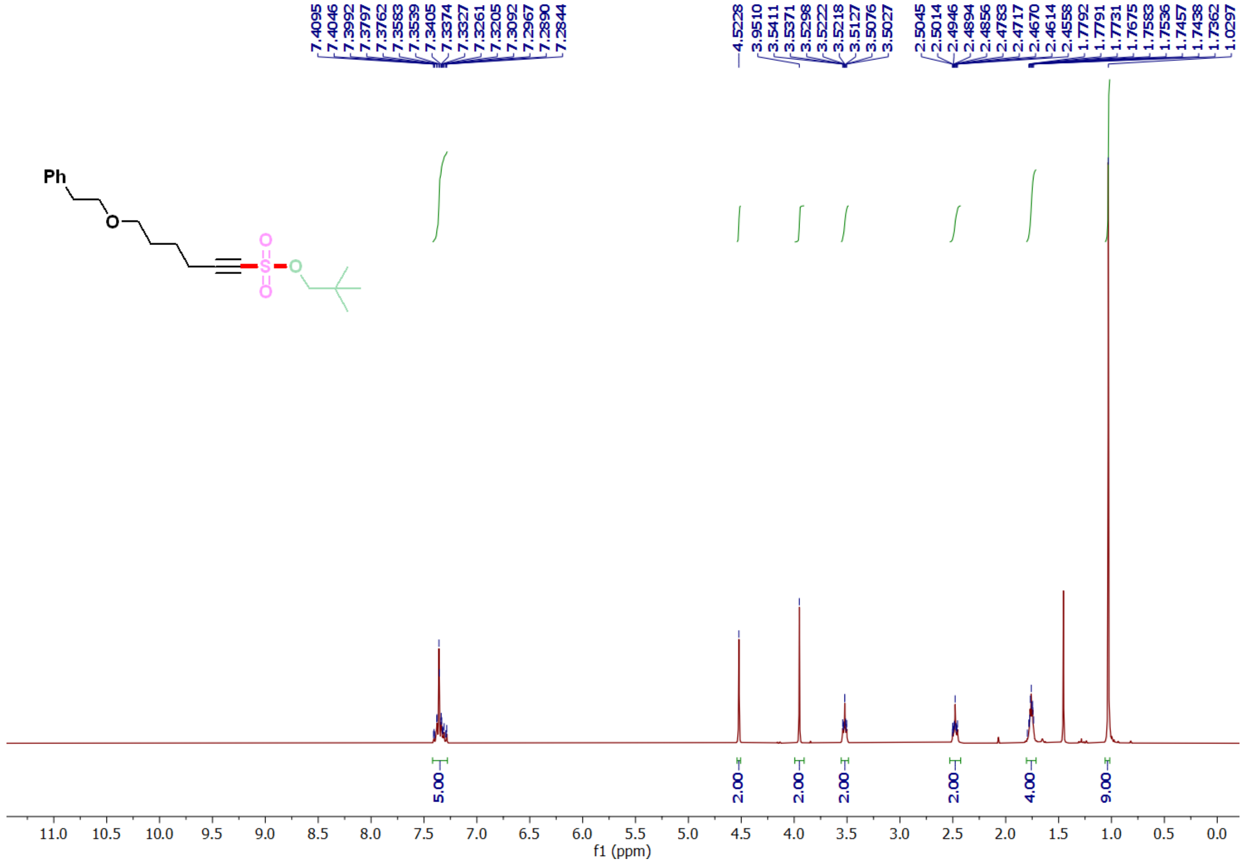

^{13}C NMR (75MHz, $CDCl_3$) of **3pa**

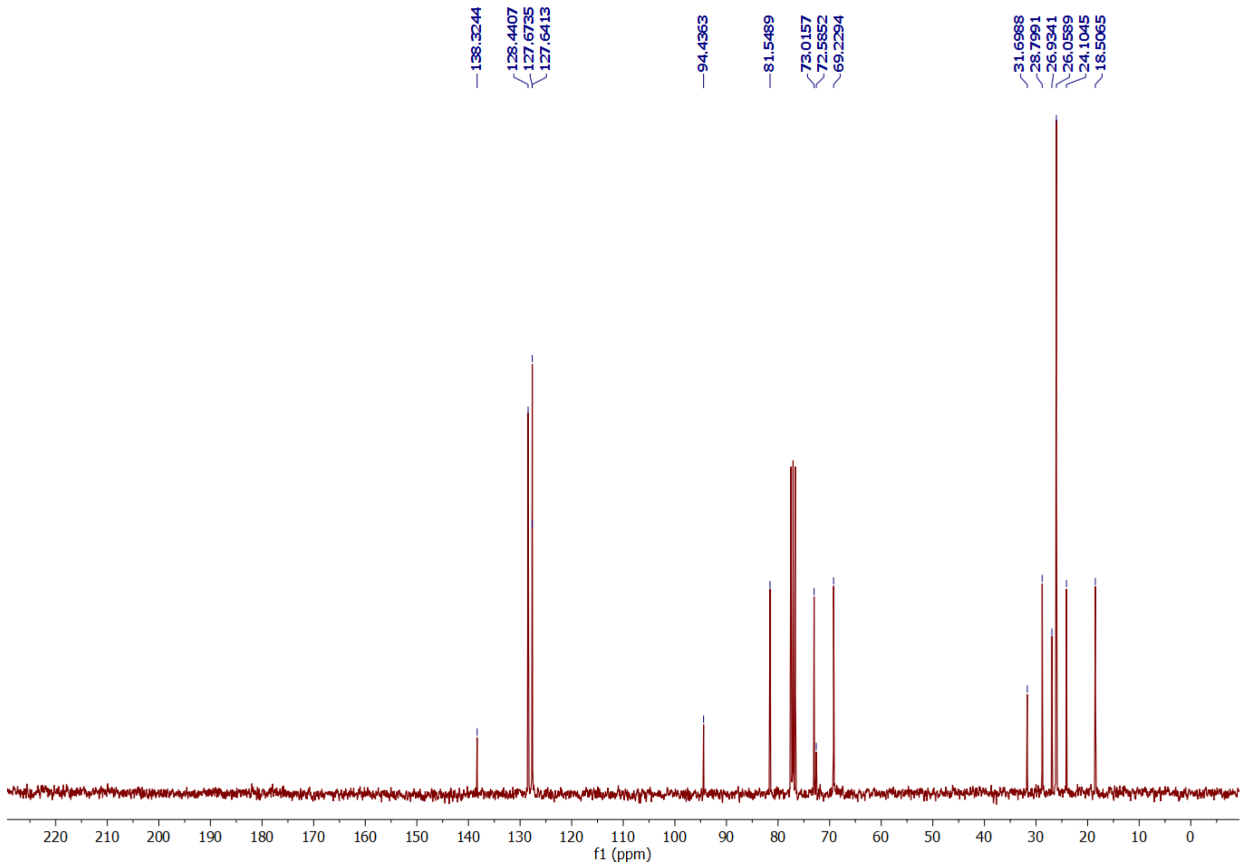

^{1}H NMR (300 MHz, $CDCl_3$) of **3qa** (see procedure)

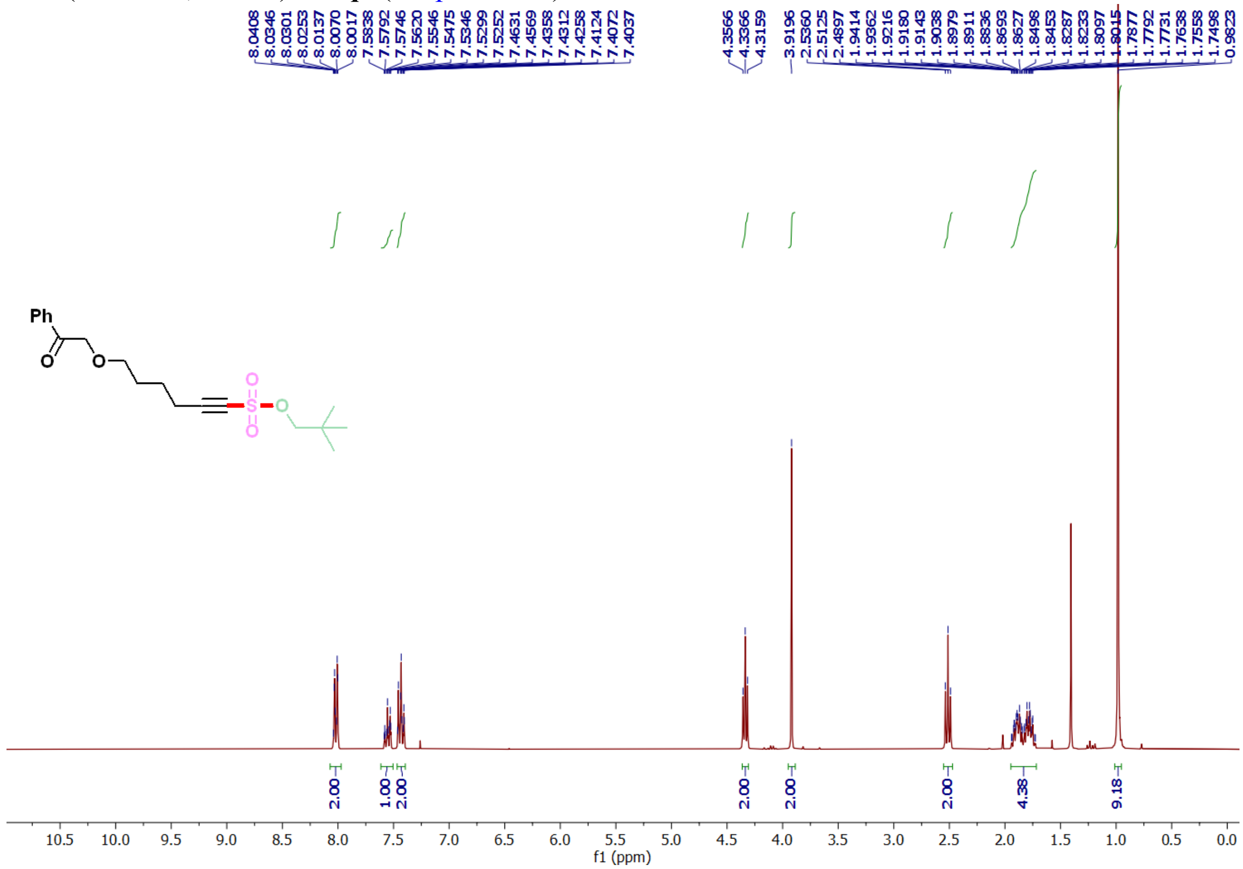

^{13}C NMR (75MHz, $CDCl_3$) of **3qa**

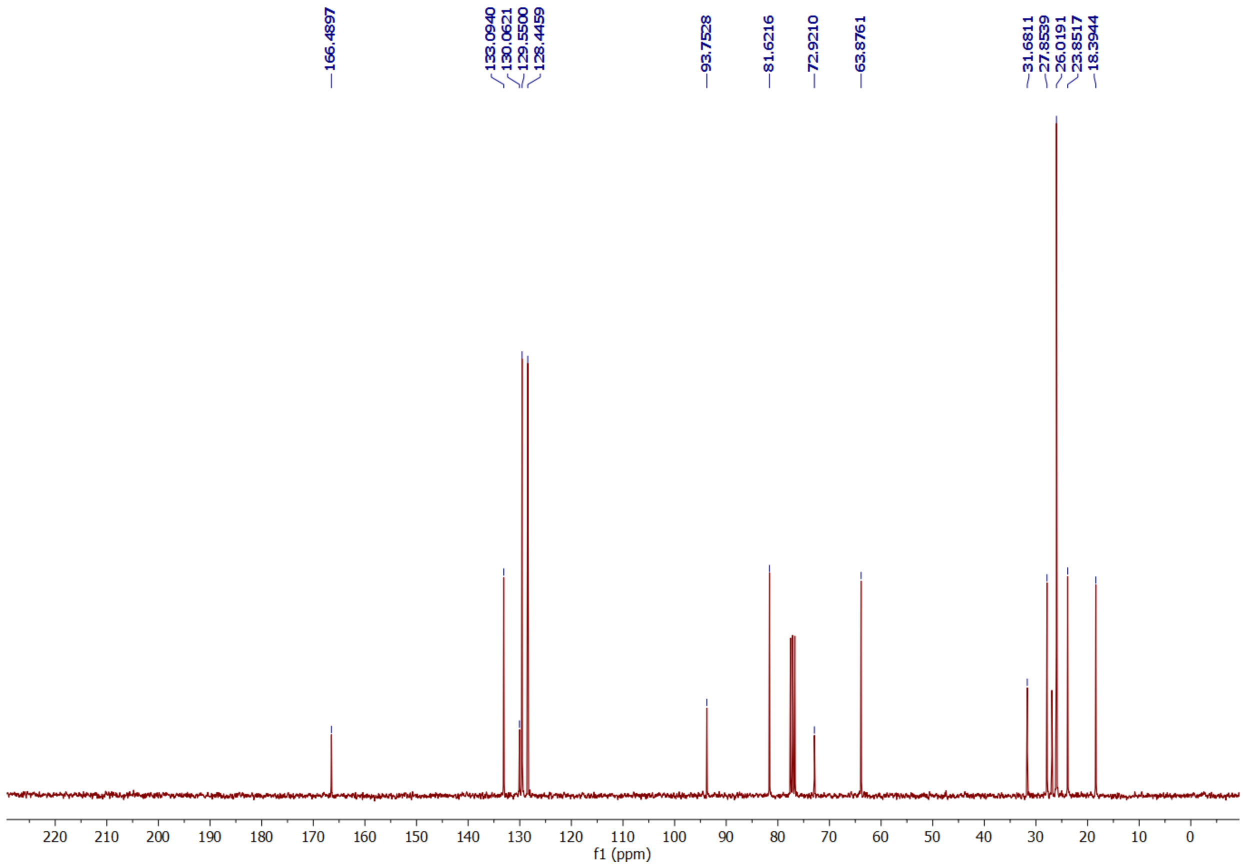

^{1}H NMR (400 MHz, $CDCl_3$) of **3ra** (see procedure)

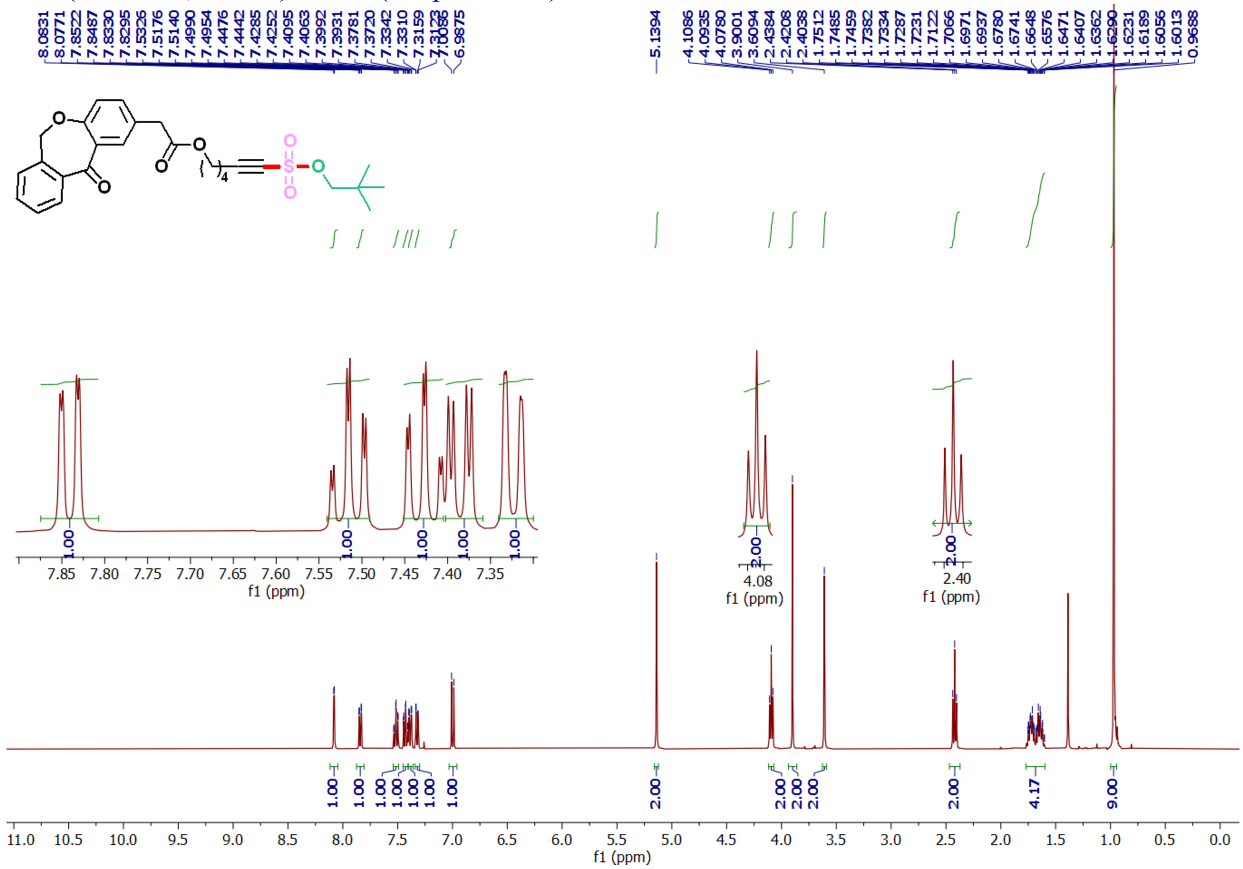

^{13}C NMR (101MHz, $CDCl_3$) of **3ra**

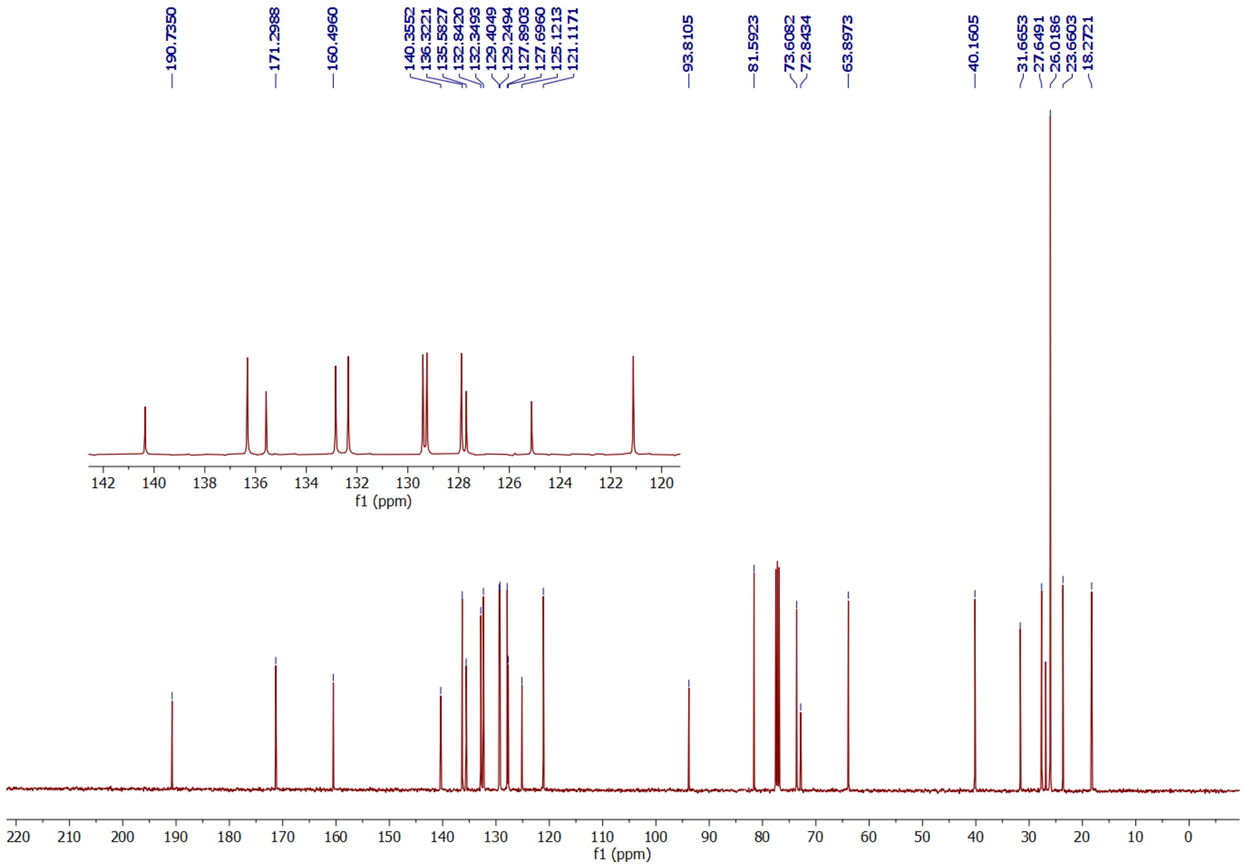

^{1}H NMR (300 MHz, $CDCl_3$) of **3sa** (see procedure)

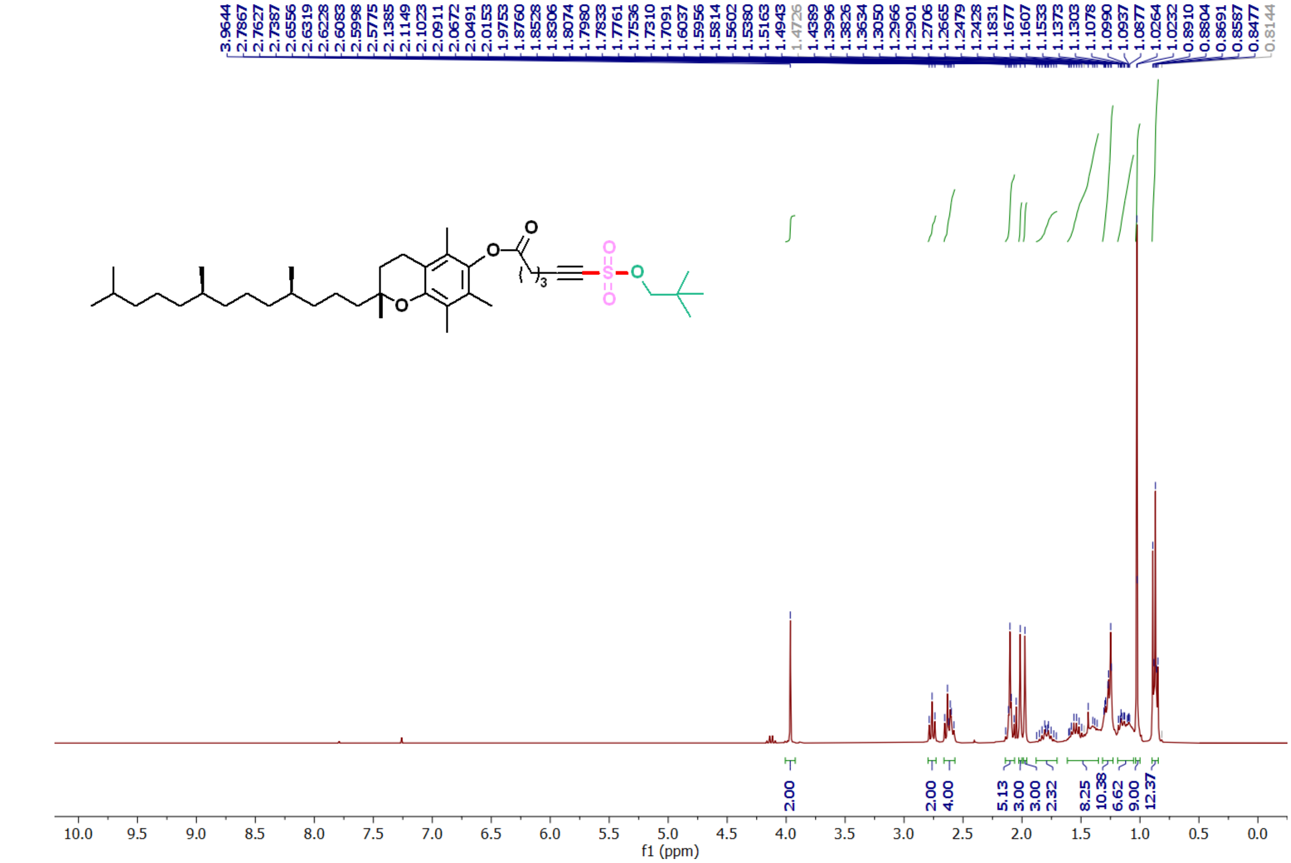

^{13}C NMR (75 MHz, $CDCl_3$) of **3sa**

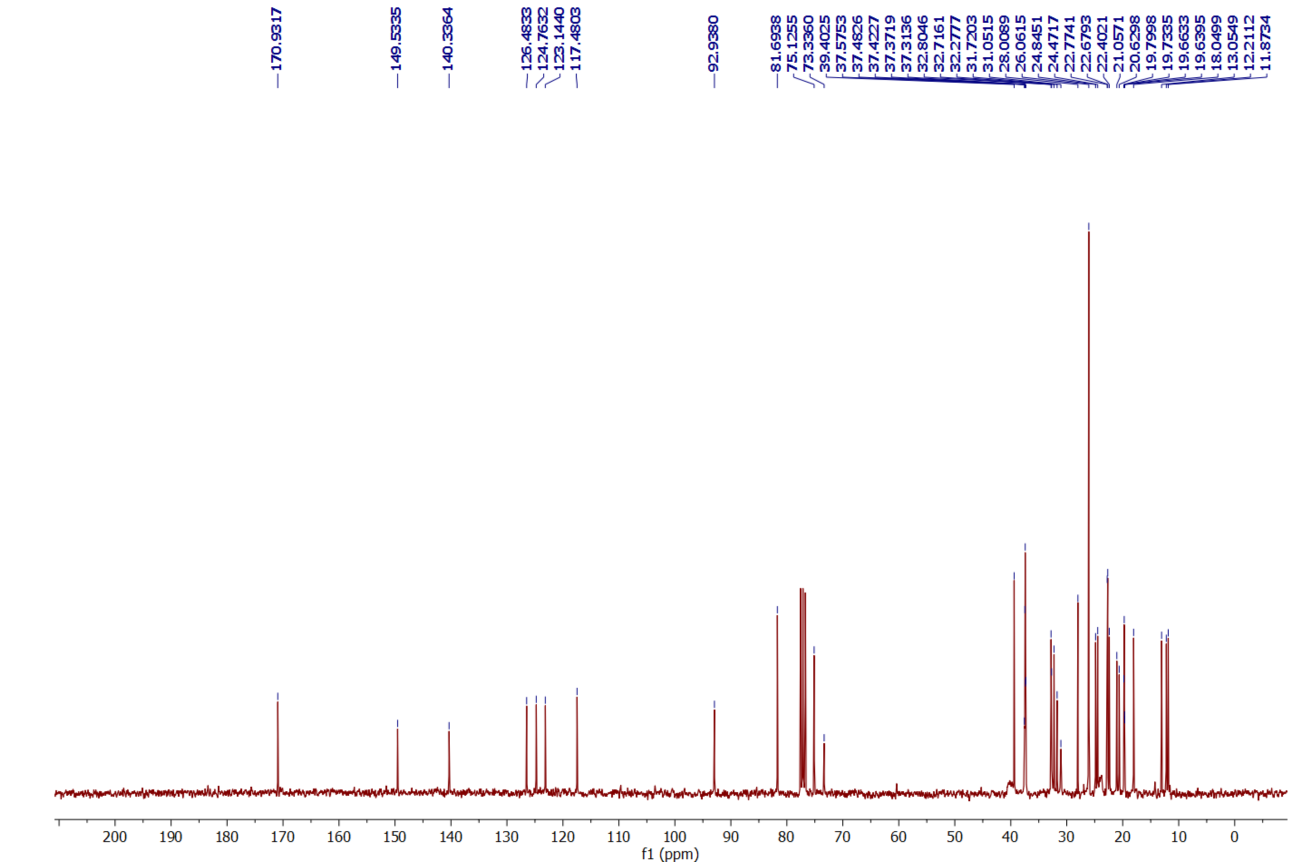

^{1}H NMR (400 MHz, $CDCl_3$) of **3ta** (see procedure)

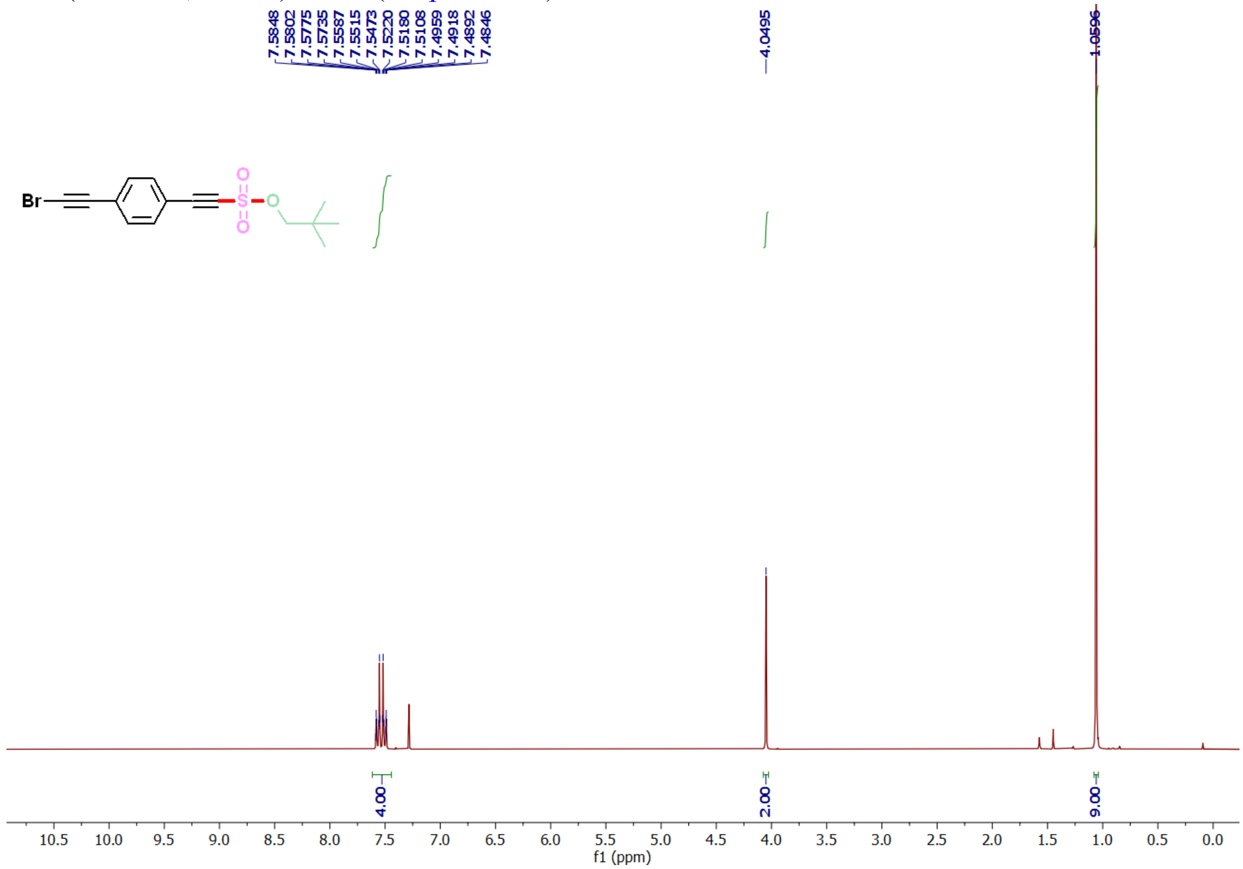

^{13}C NMR (101MHz, $CDCl_3$) of **3ta**

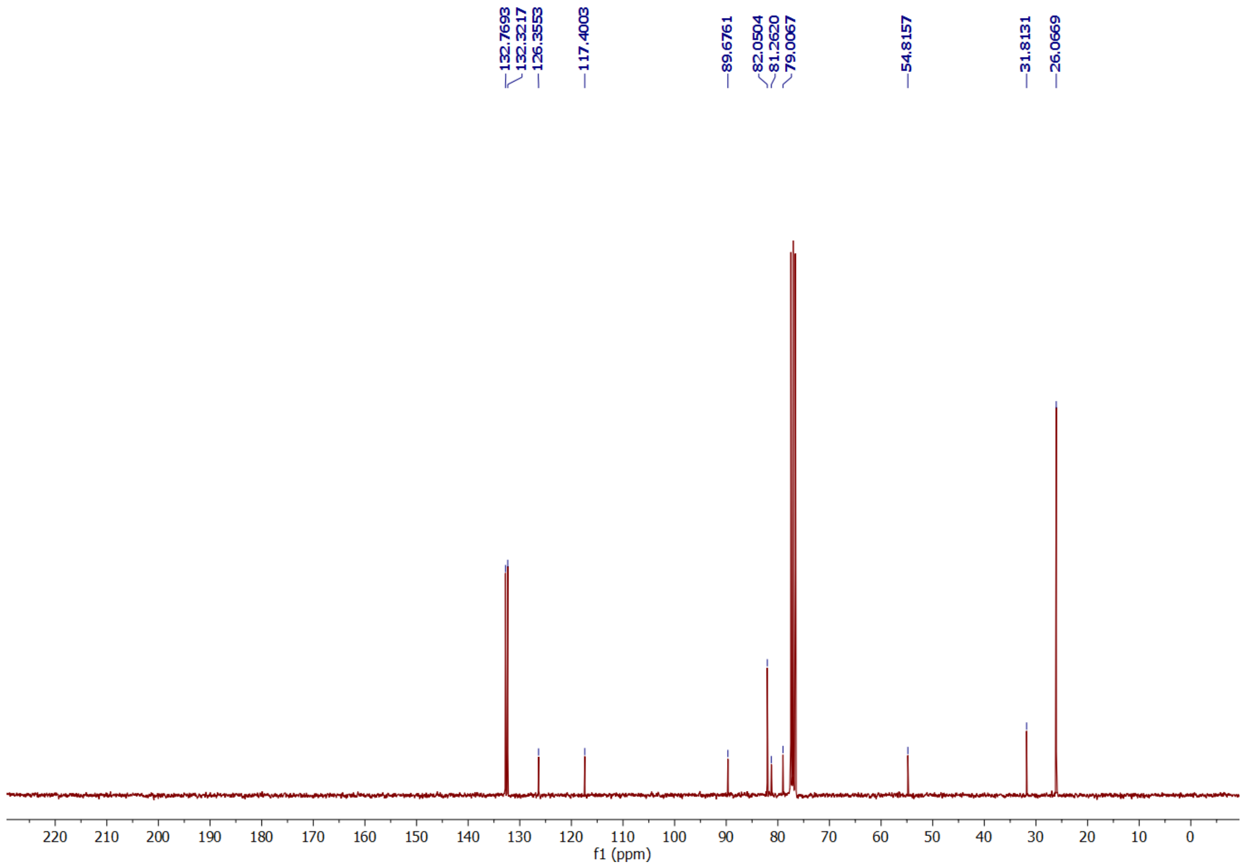

^{1}H NMR (400 MHz, $CDCl_3$) of **3ua** (see procedure)

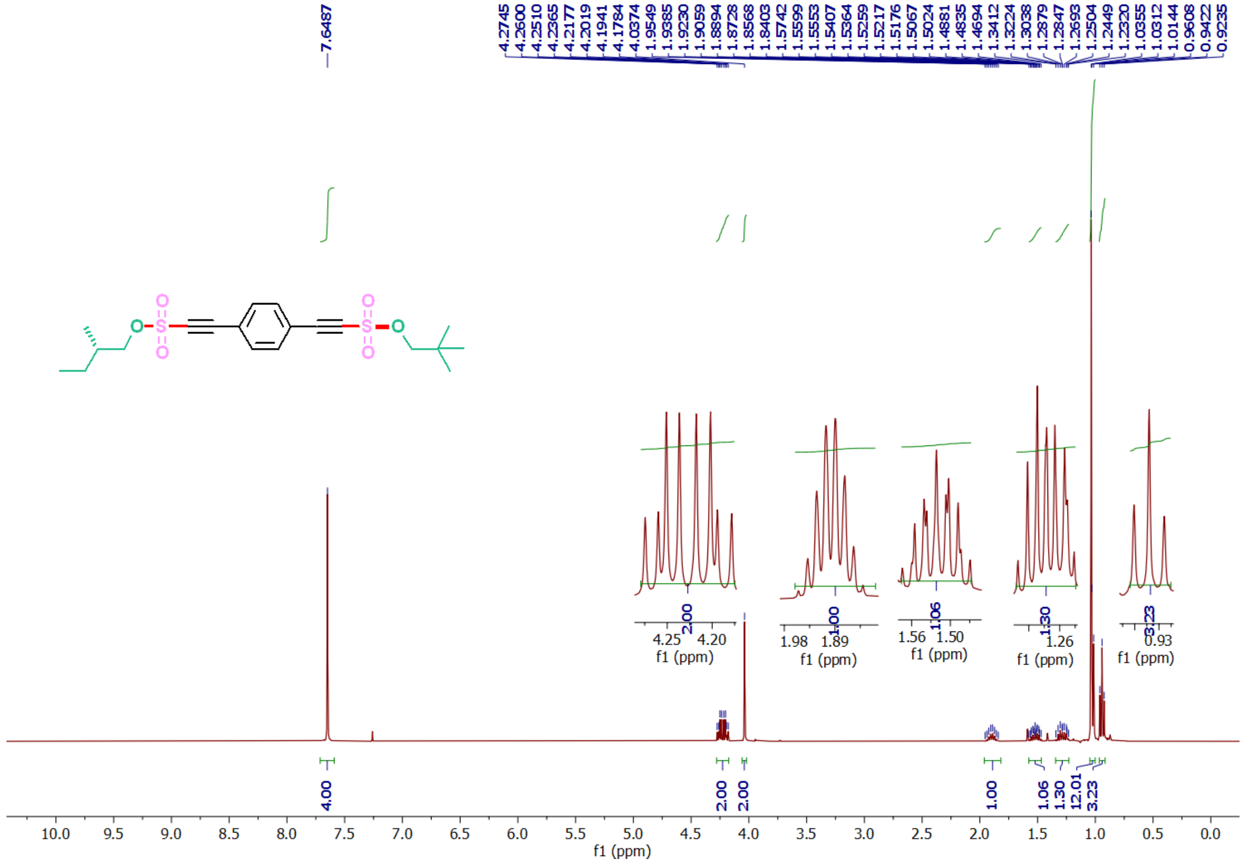

^{13}C NMR (101MHz, $CDCl_3$) of **3ua**

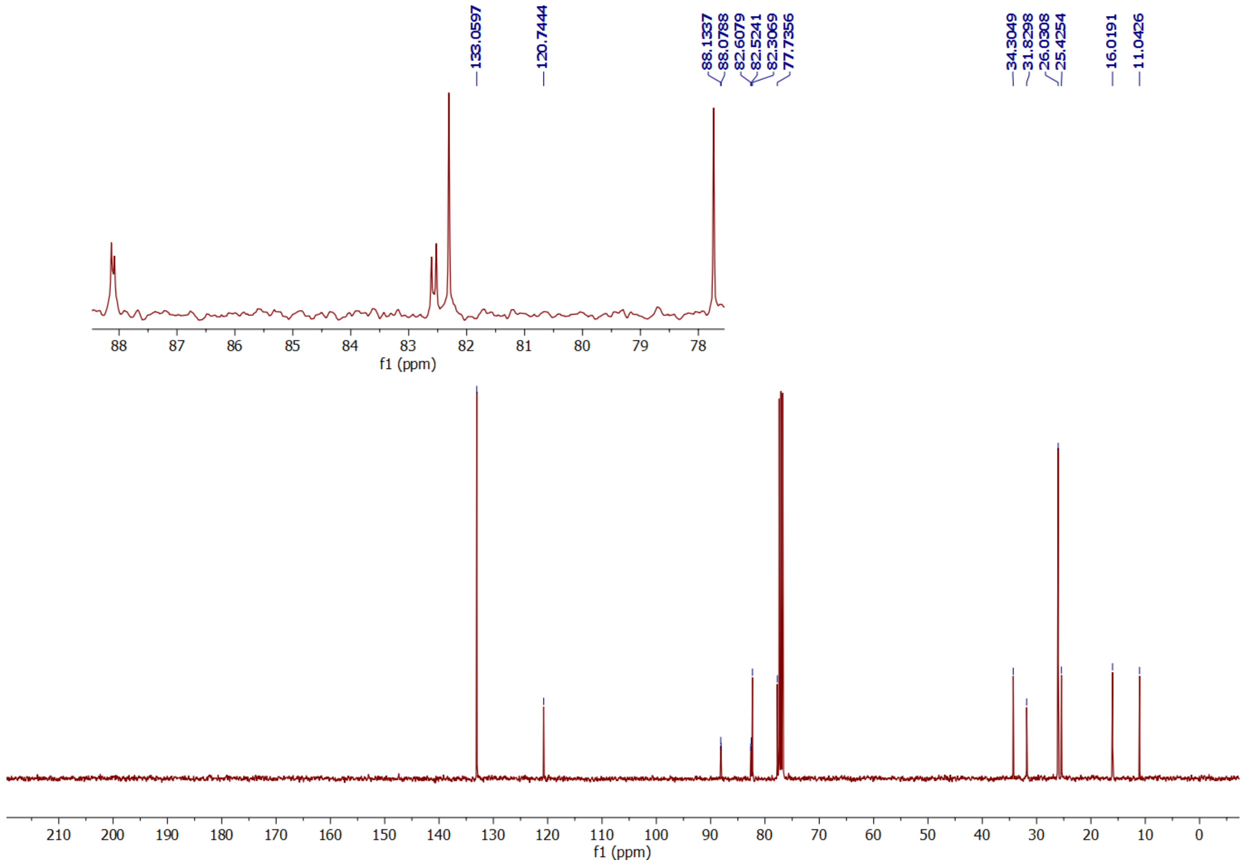

^{1}H NMR (300 MHz, $CDCl_3$) of **3va** (see procedure)

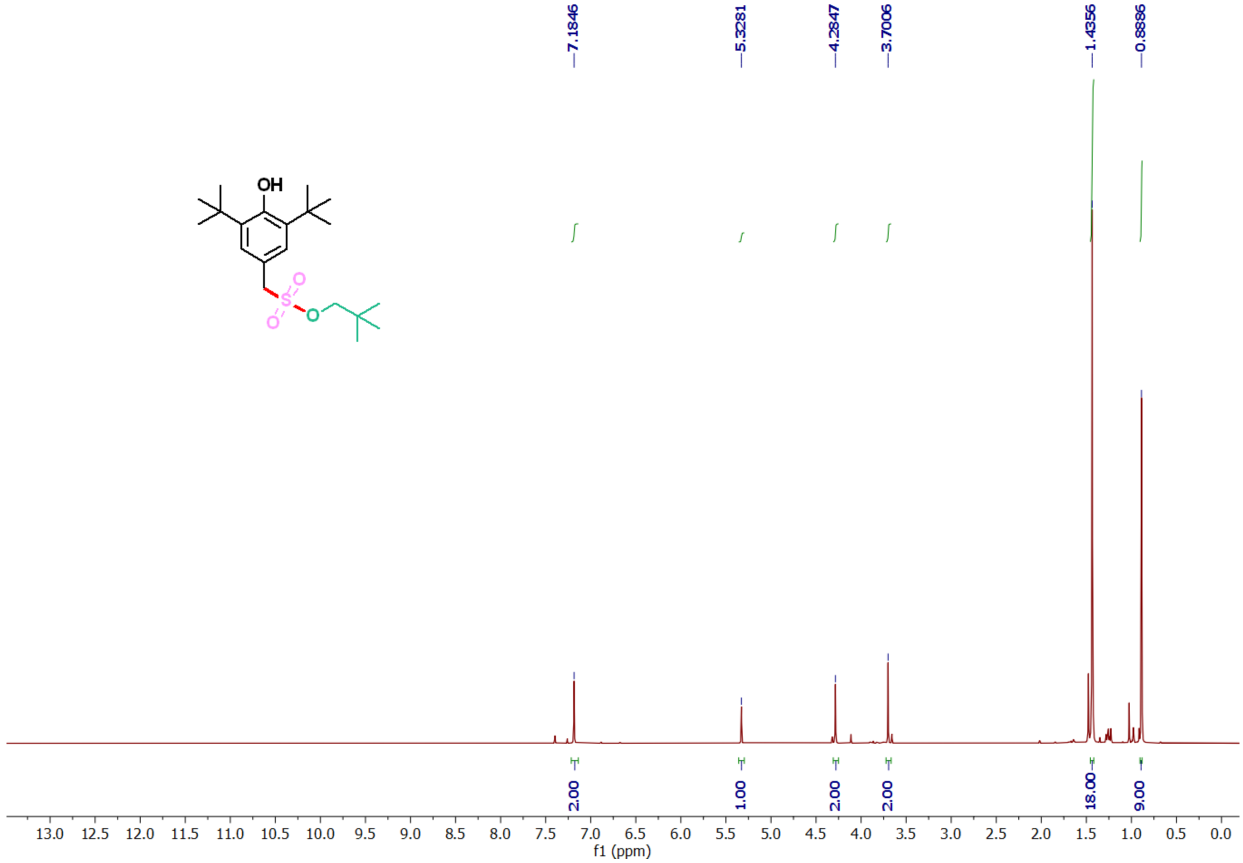

^{13}C NMR (101MHz, $CDCl_3$) of **3va**

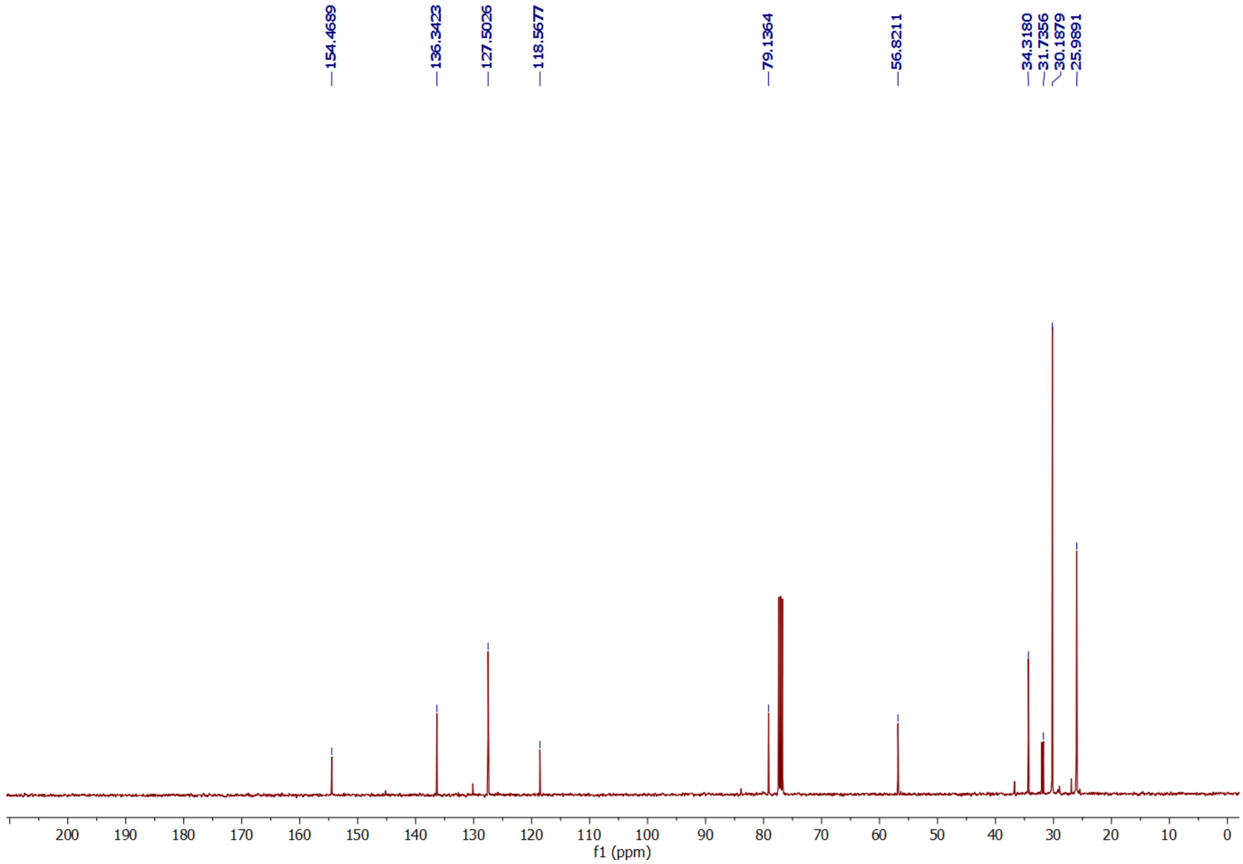

^{1}H NMR (400 MHz, $CDCl_3$) of **3wa** (see procedure)

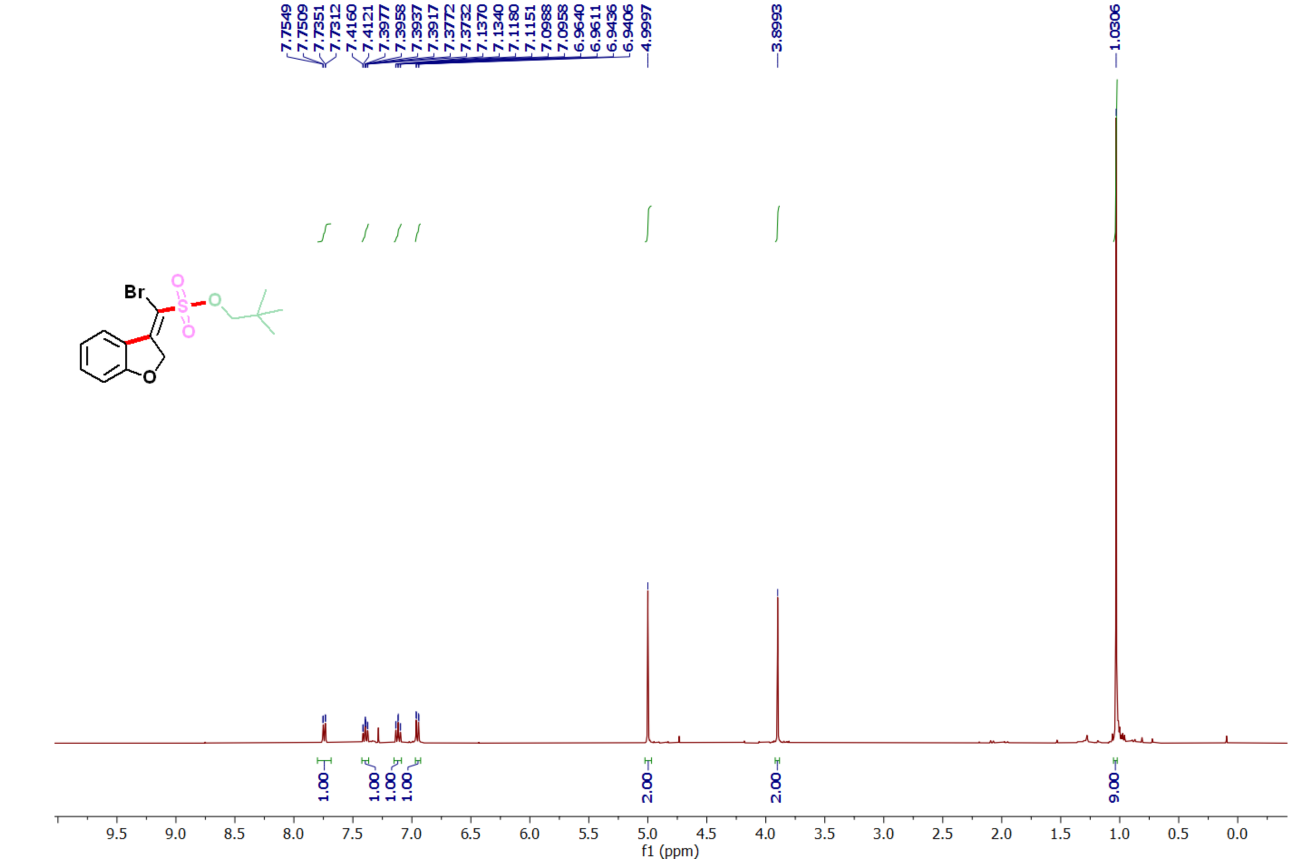

^{13}C NMR (101MHz, $CDCl_3$) of **3wa**

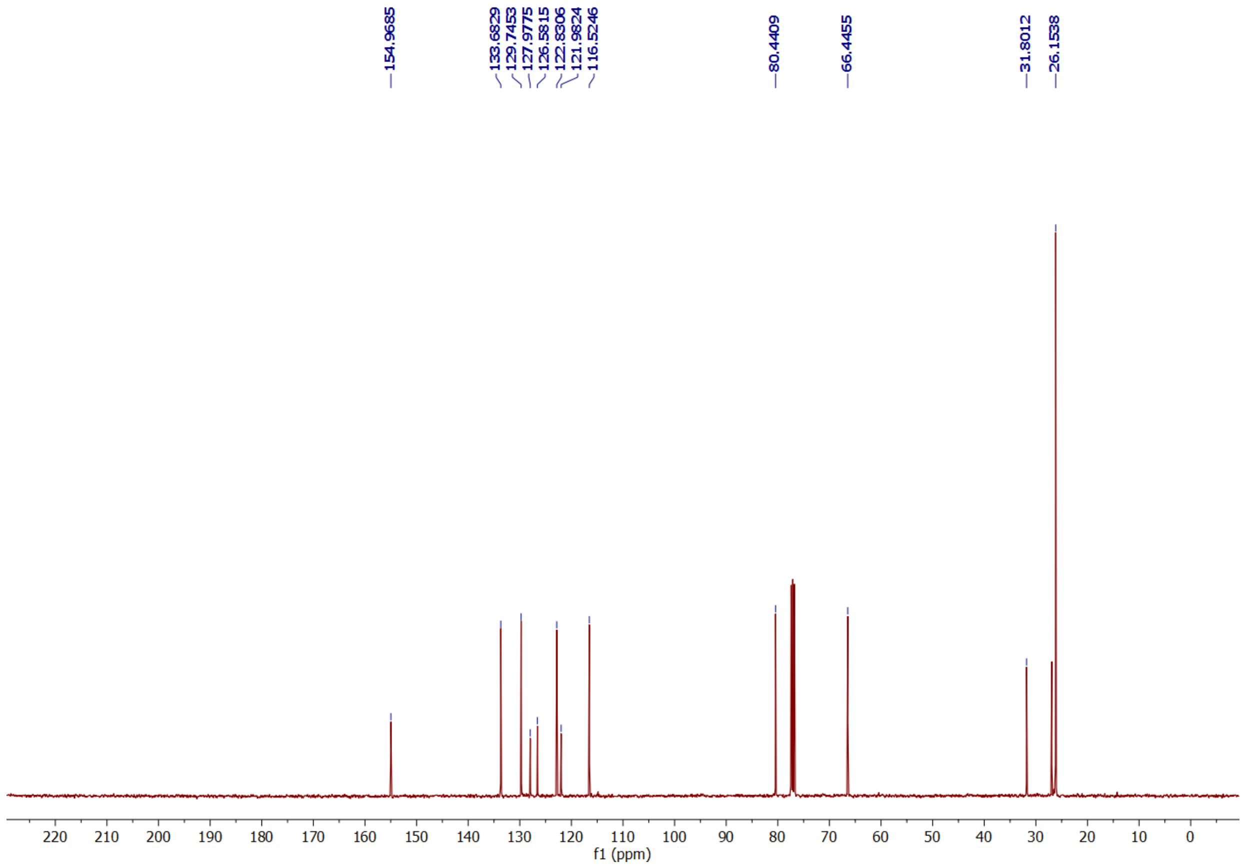

NOE Spectrum for **3wa**

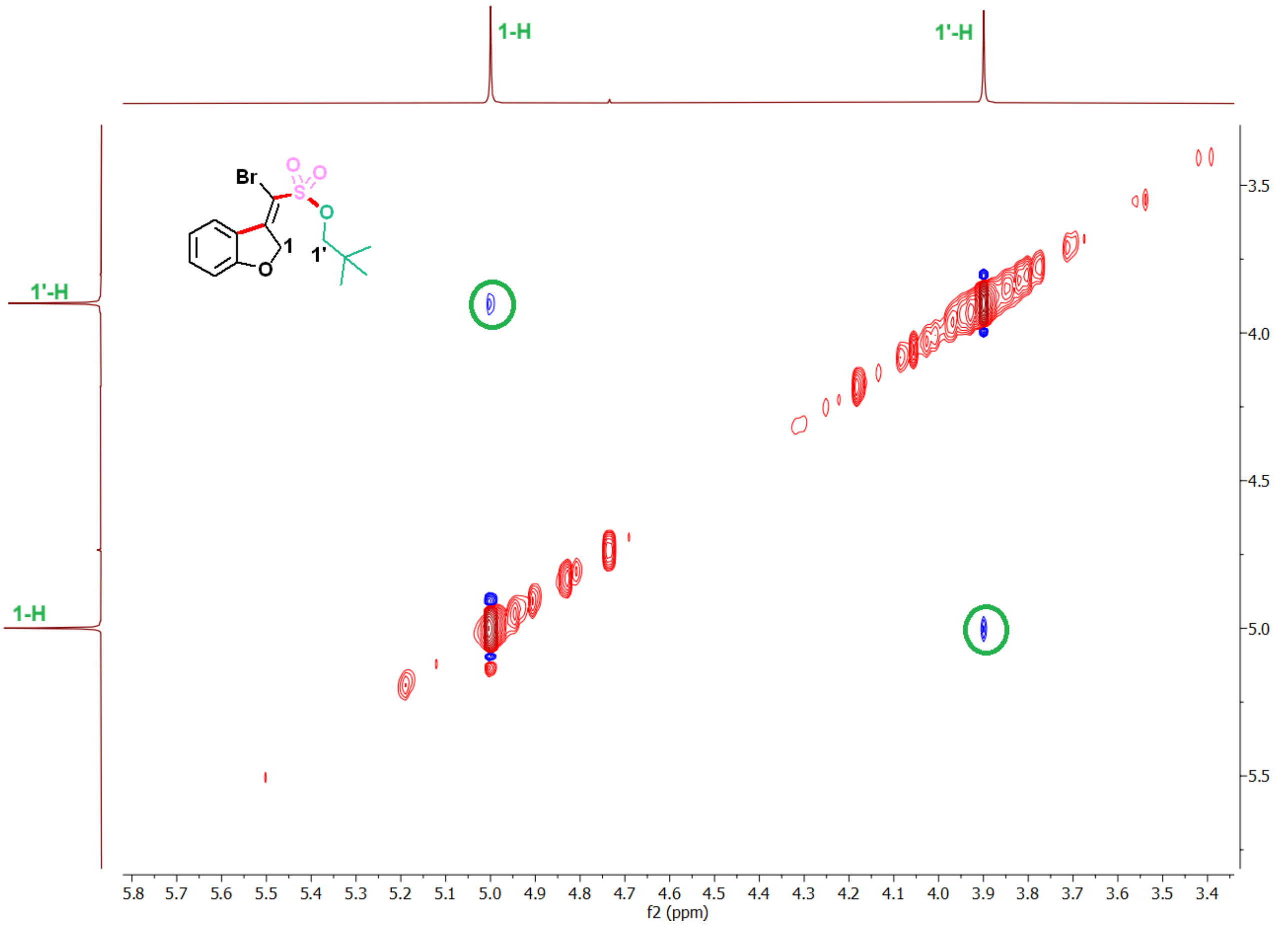

^{1}H NMR (400 MHz, $CDCl_3$) of **4a** (see procedure)

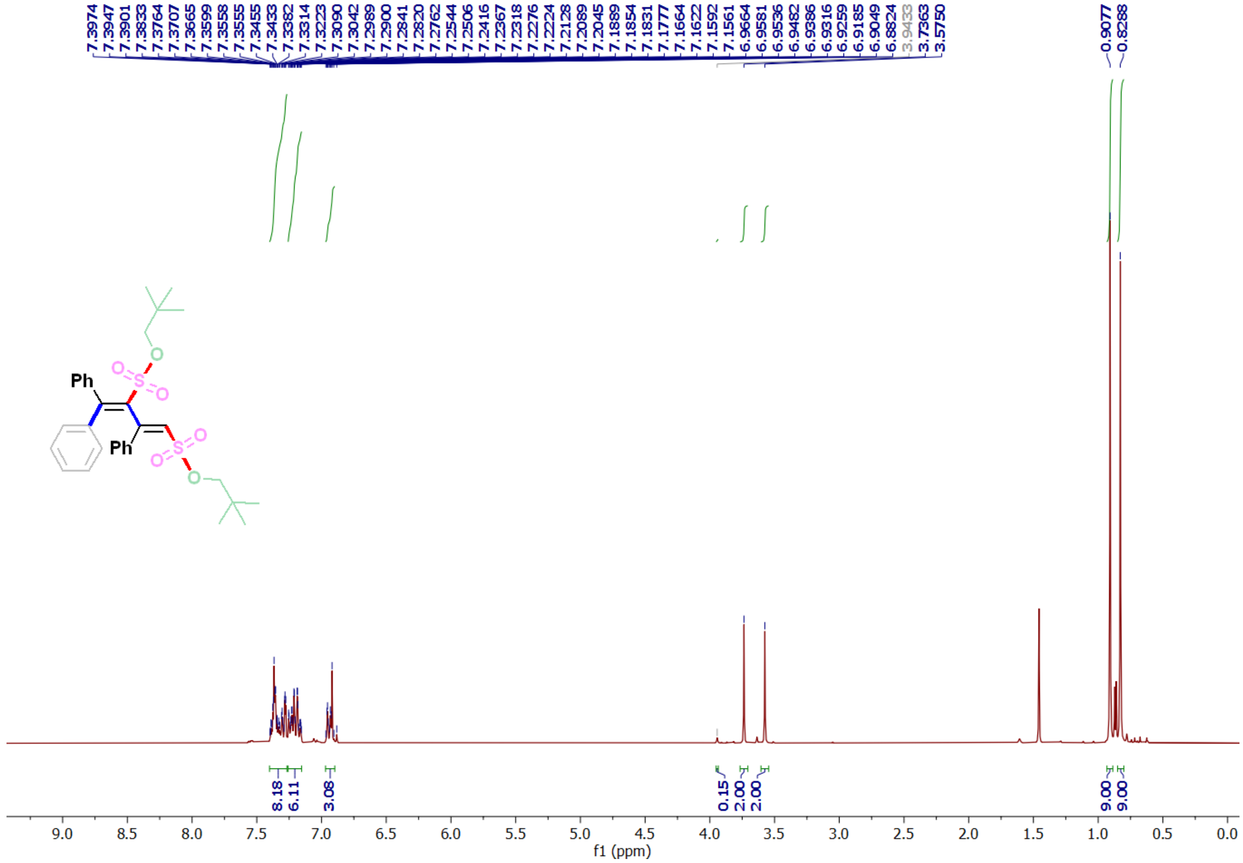

^{13}C NMR (101MHz, $CDCl_3$) of **4a**

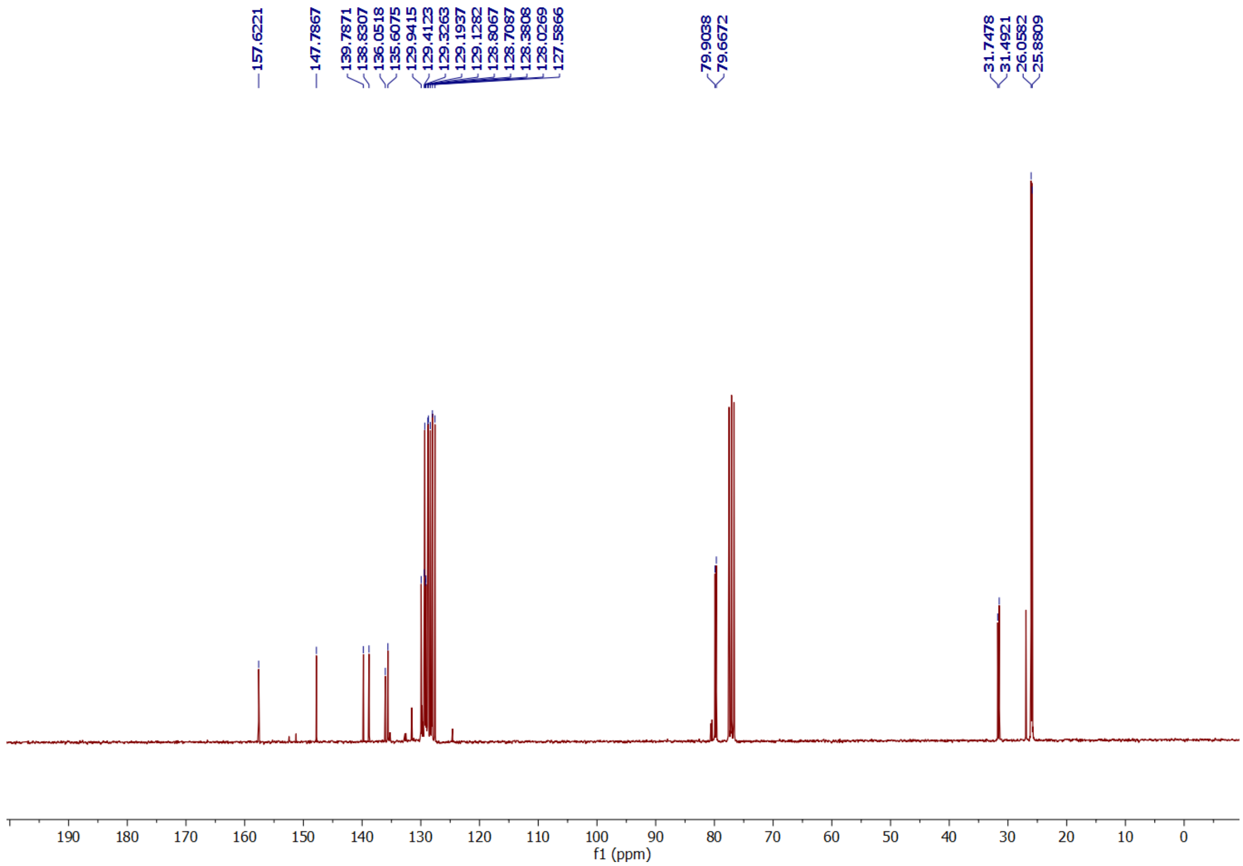

^{1}H NMR (400 MHz, $CDCl_3$) of **4b** (see procedure)

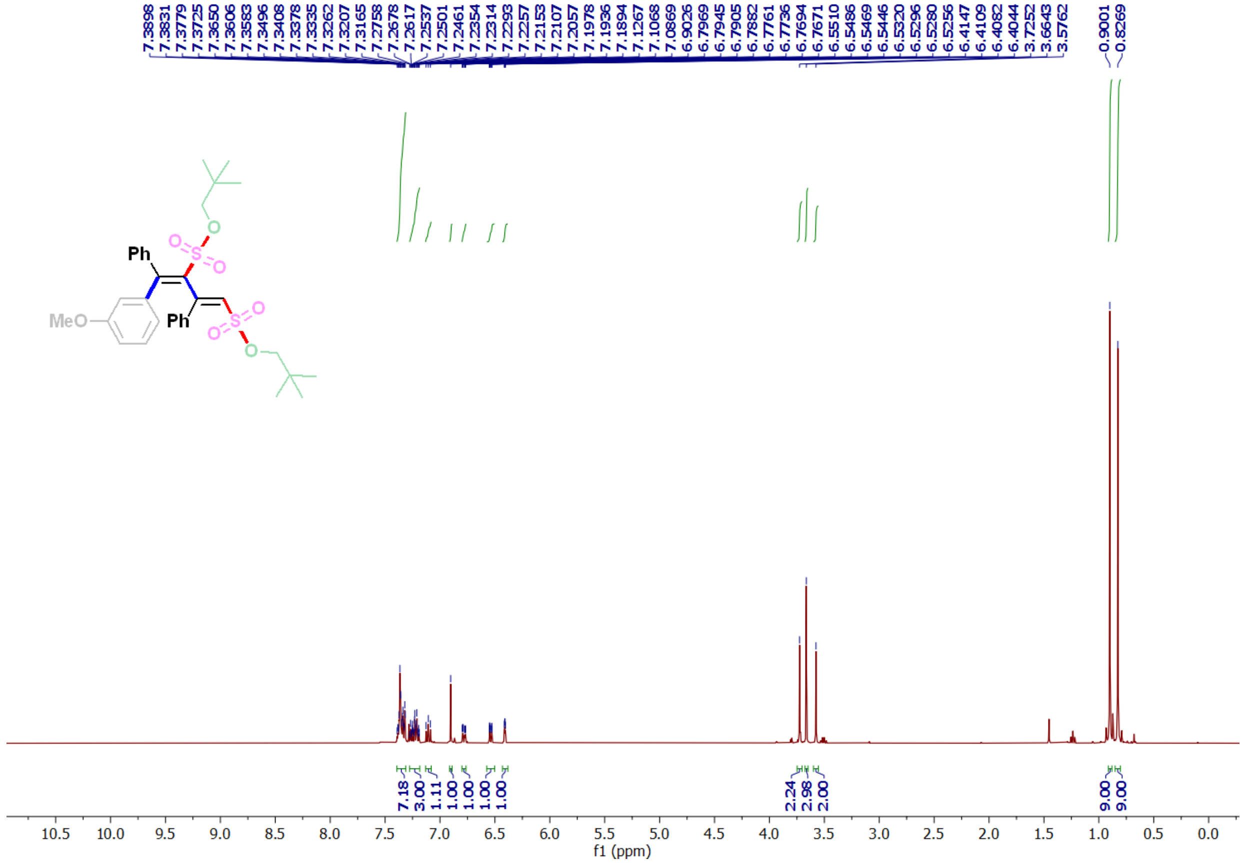

^{13}C NMR (101MHz, $CDCl_3$) of **4b**

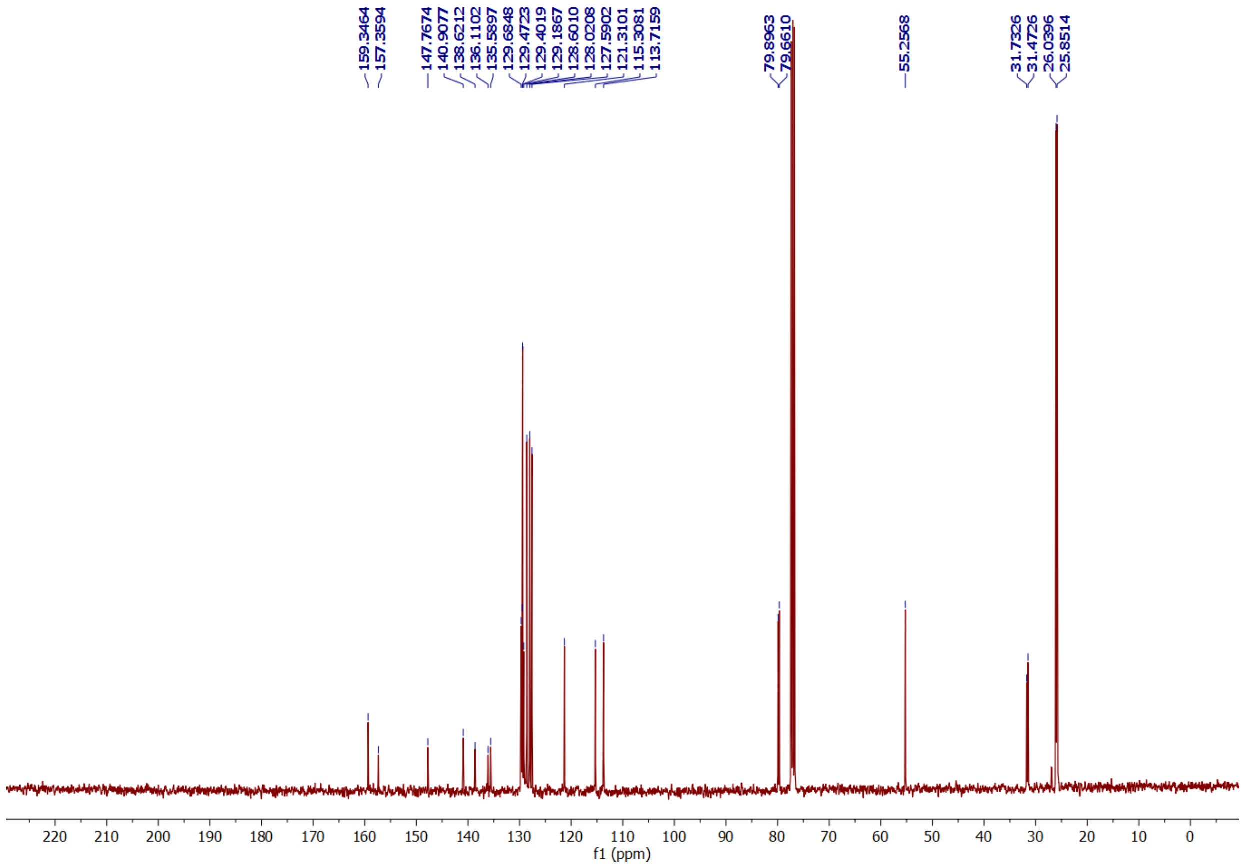

^{1}H NMR (300 MHz, $CDCl_3$) of **4c** (see procedure)

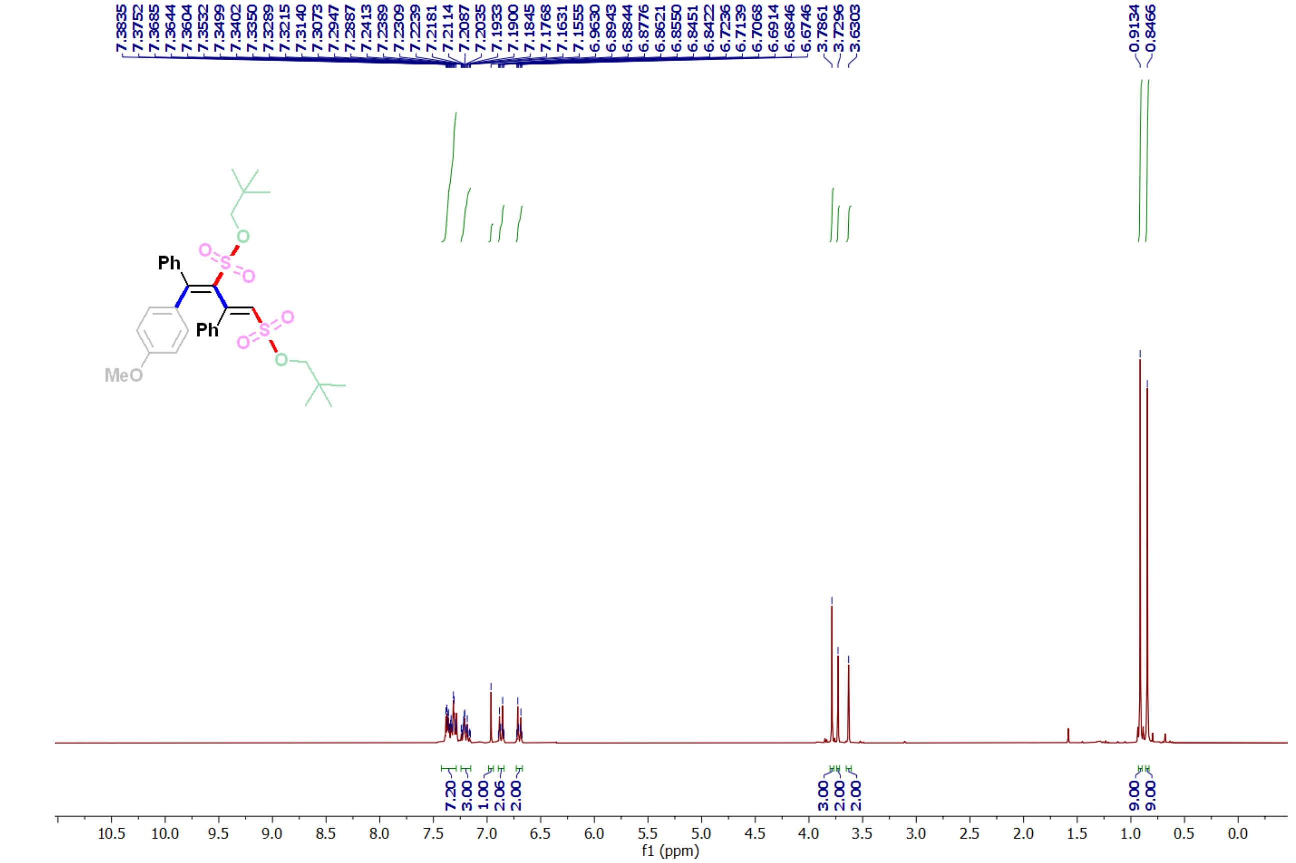

^{13}C NMR (75 MHz, $CDCl_3$) of **4c**

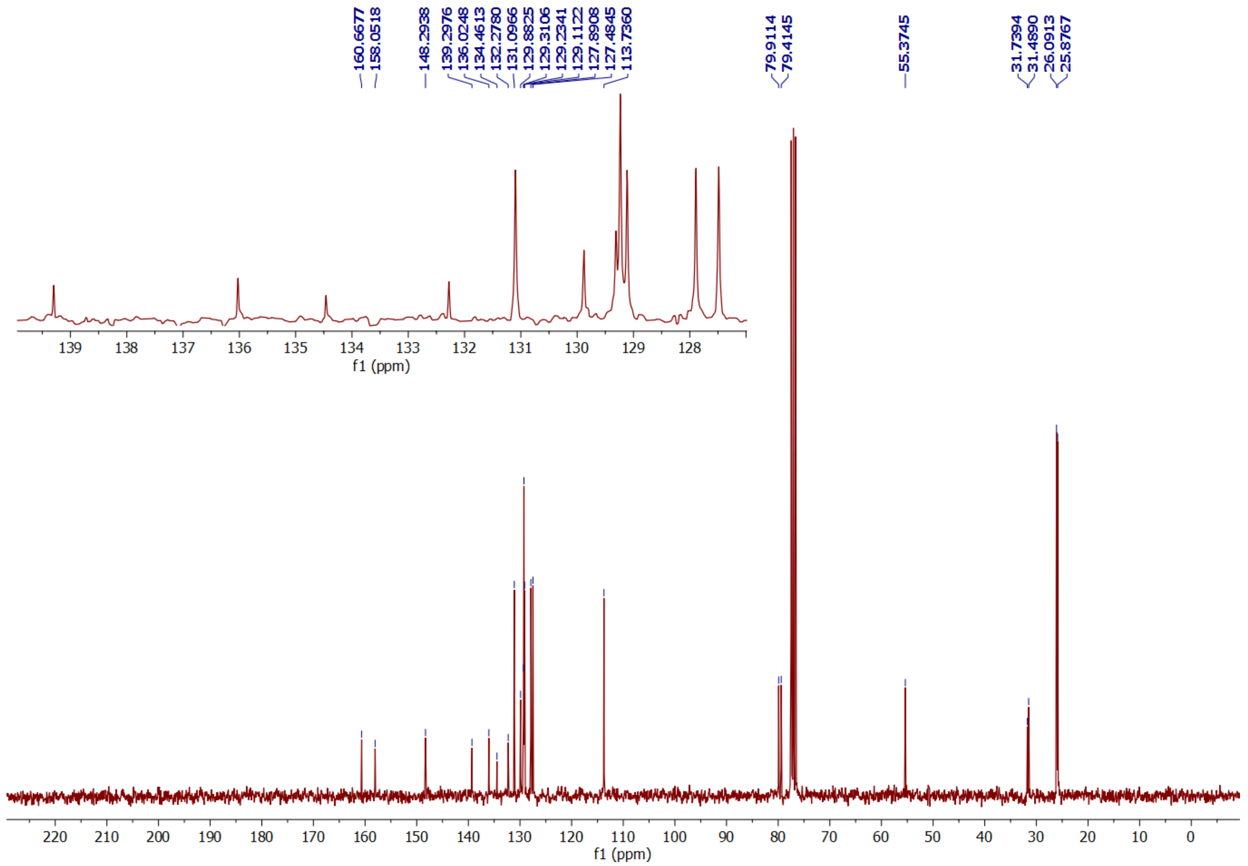

^{1}H NMR (400 MHz, $CDCl_3$) of **4d** (see procedure)

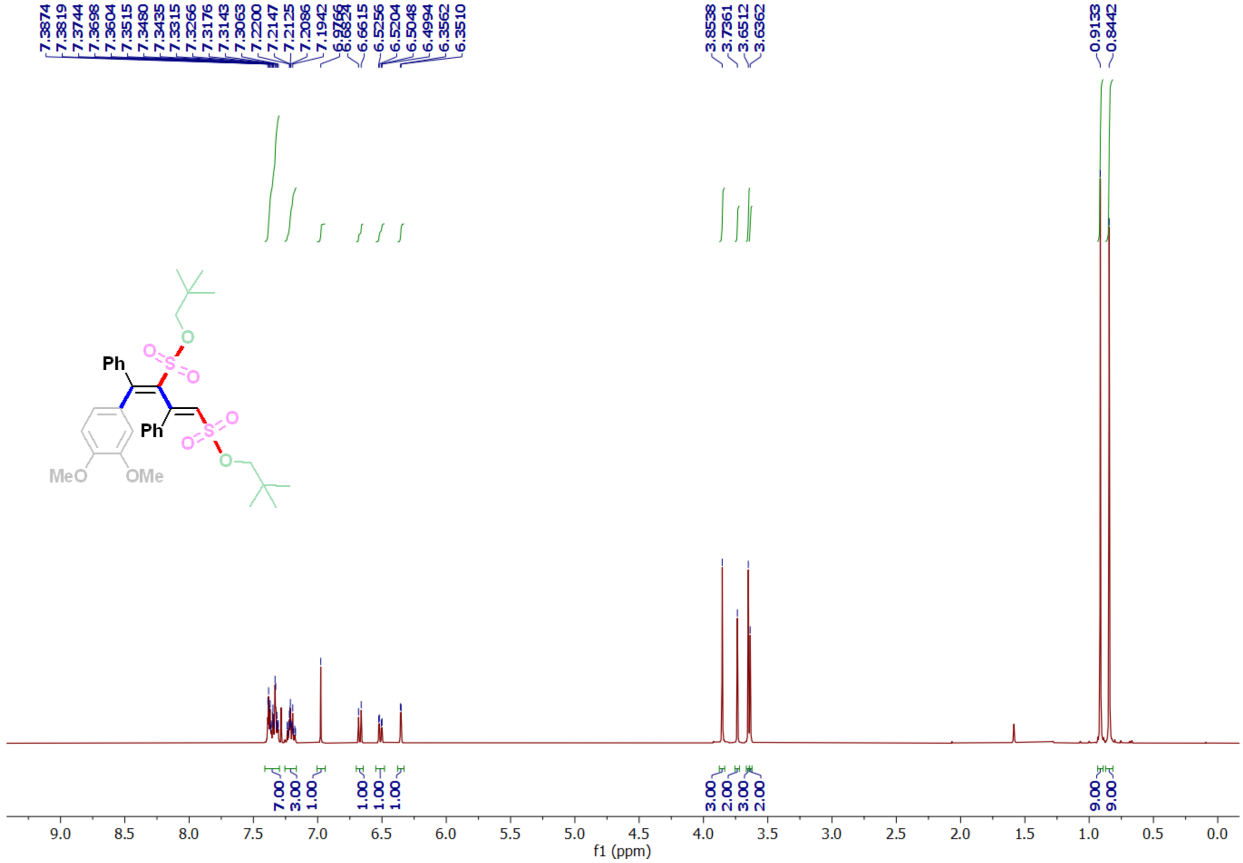

^{13}C NMR (101MHz, $CDCl_3$) of **4d**

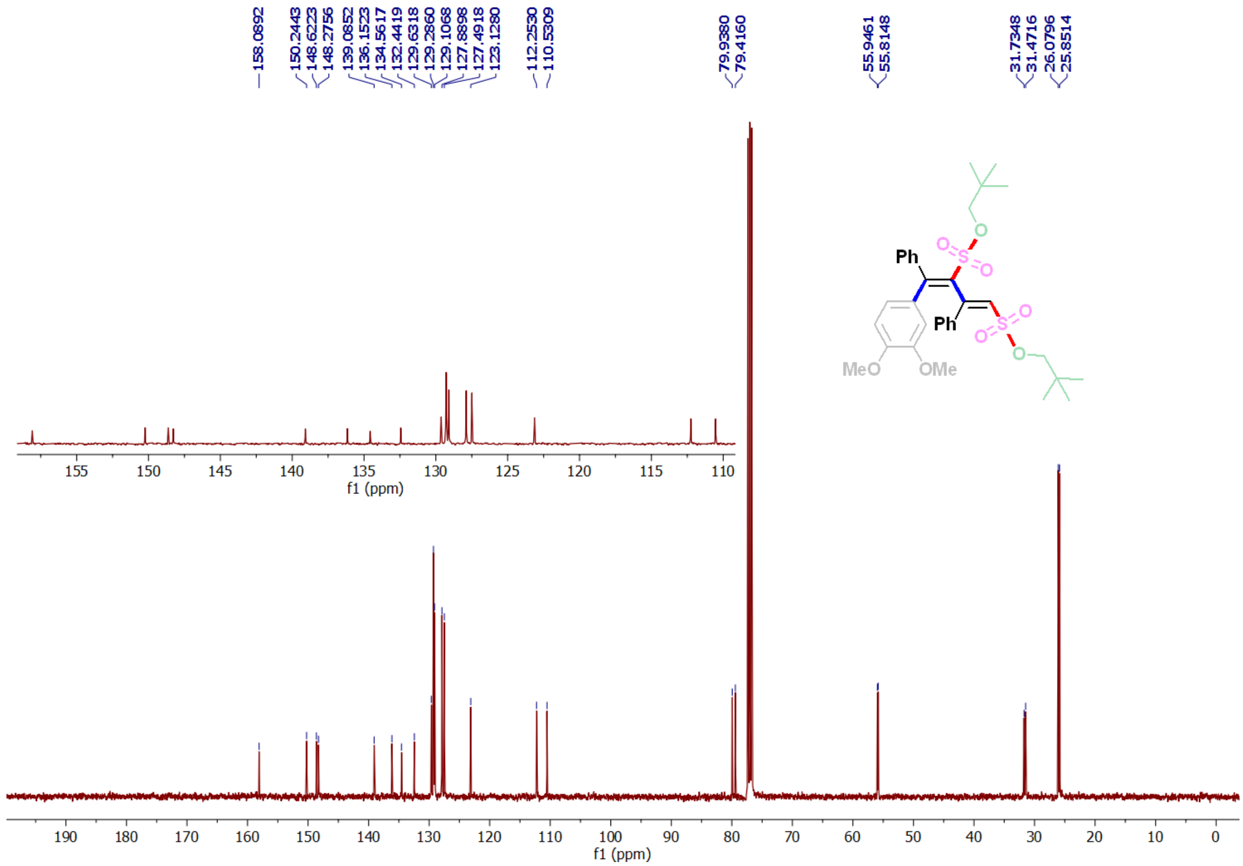

^{1}H NMR (400 MHz, $CDCl_3$) of **4e** (see procedure)

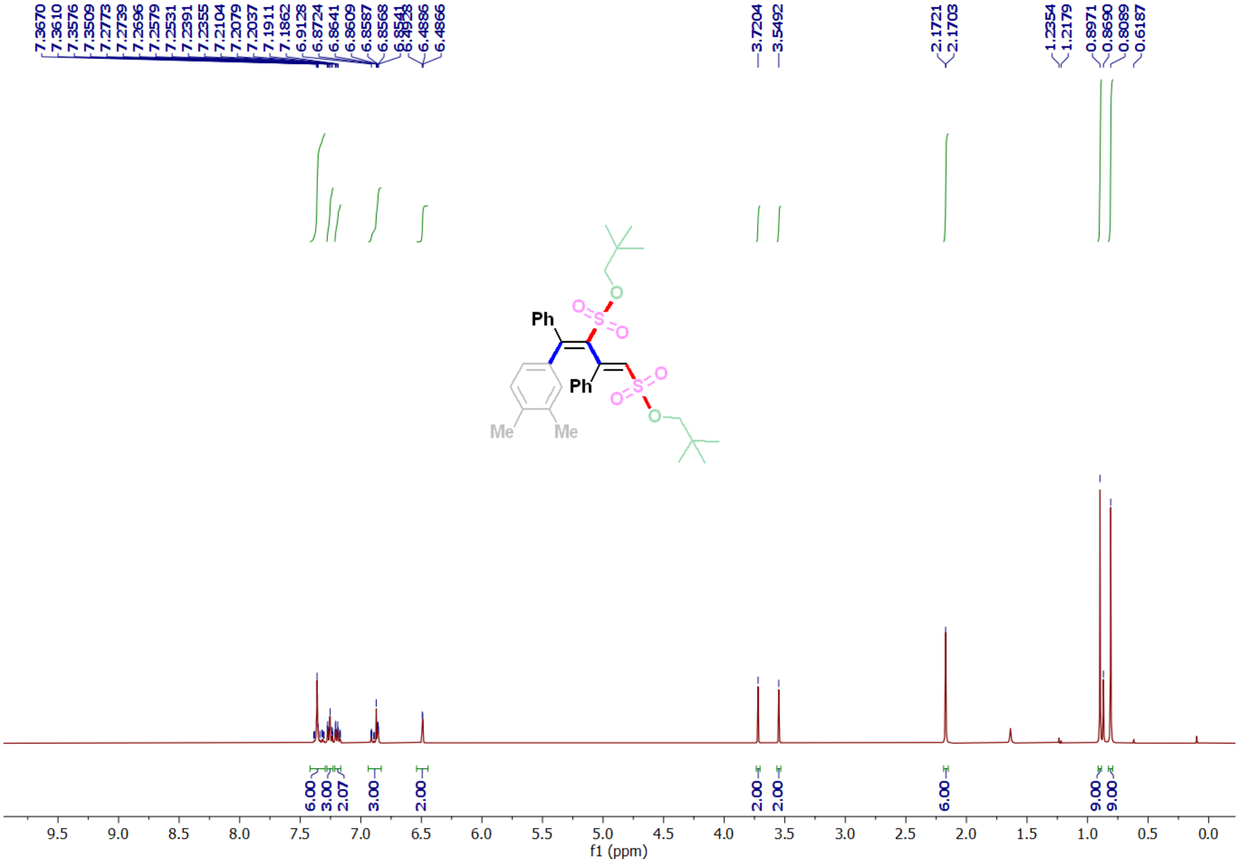

^{13}C NMR (101MHz, $CDCl_3$) of **4e**

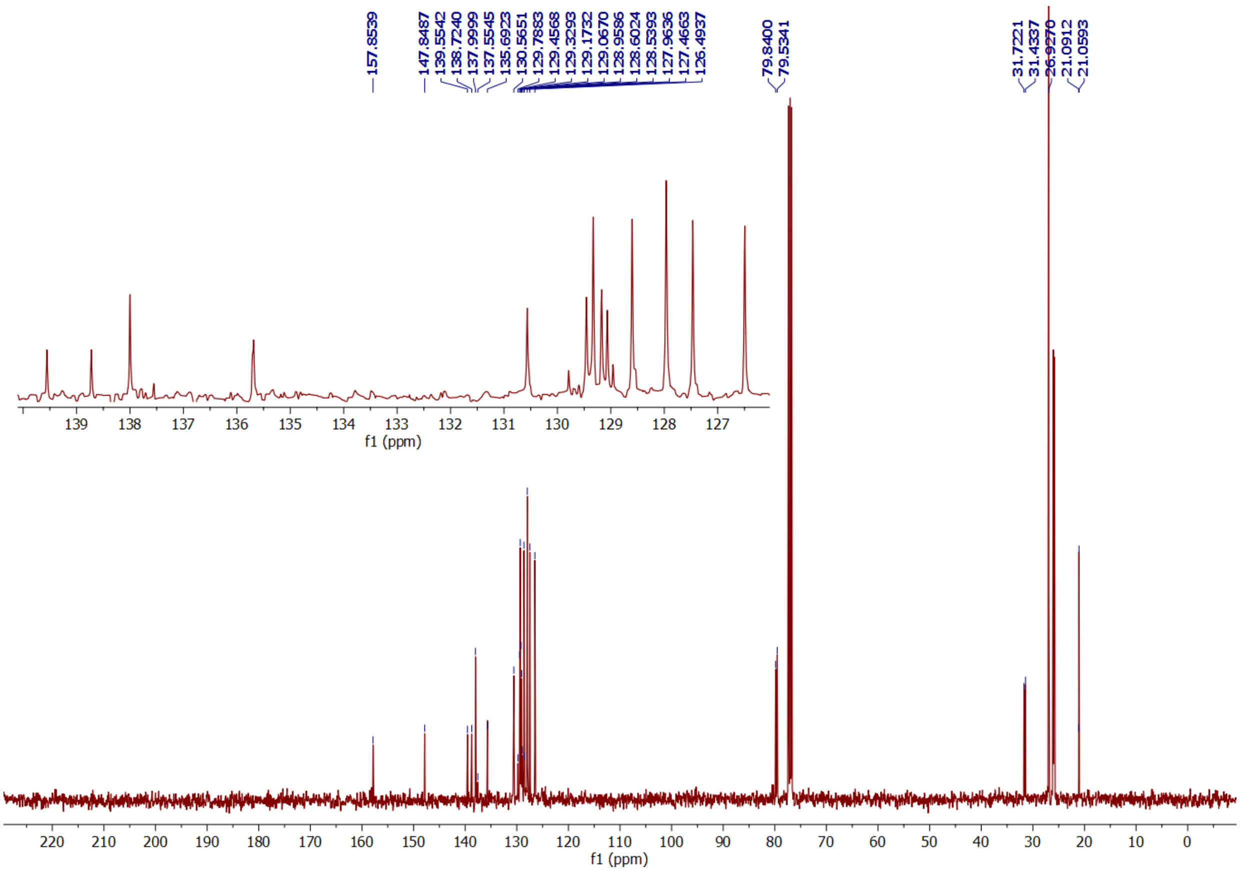

^{1}H NMR (400 MHz, $CDCl_3$) of **4f** (see procedure)

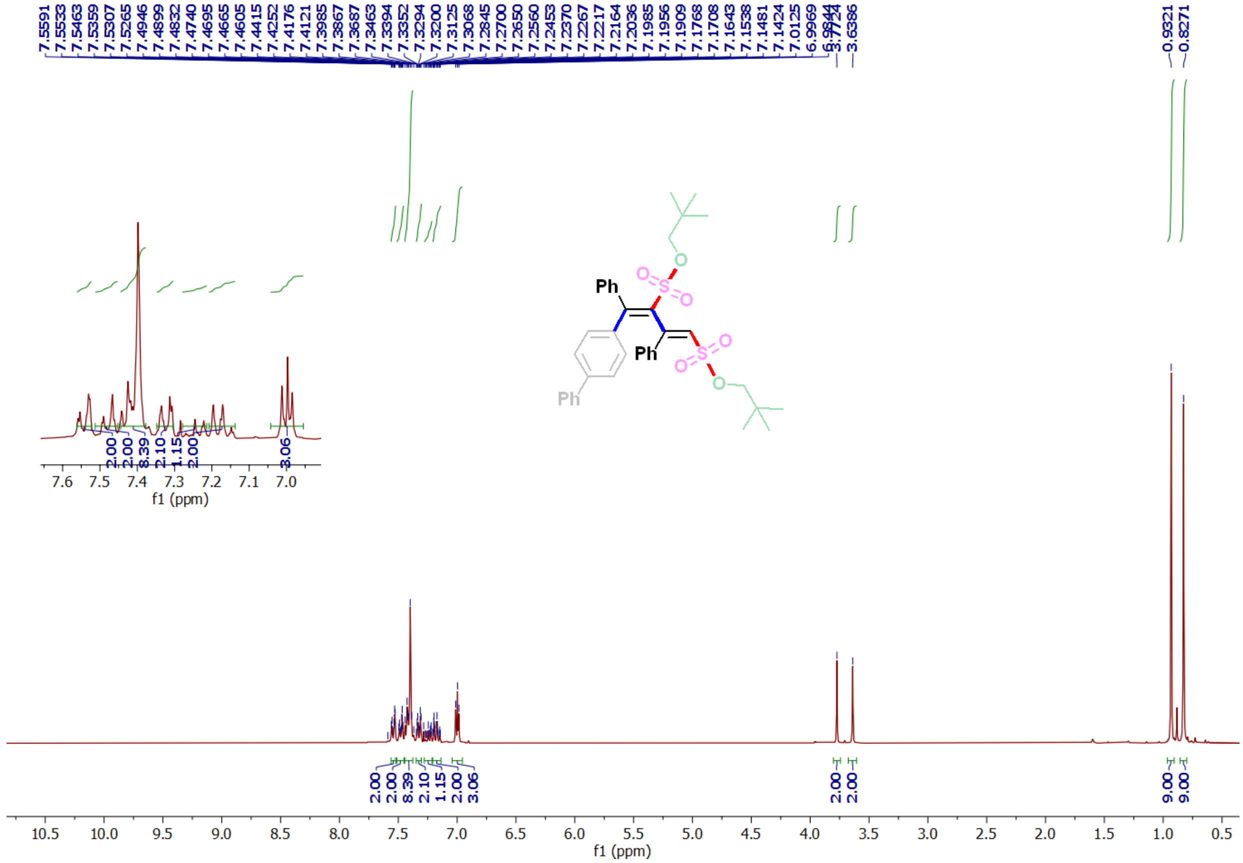

^{13}C NMR (101MHz, $CDCl_3$) of **4f**

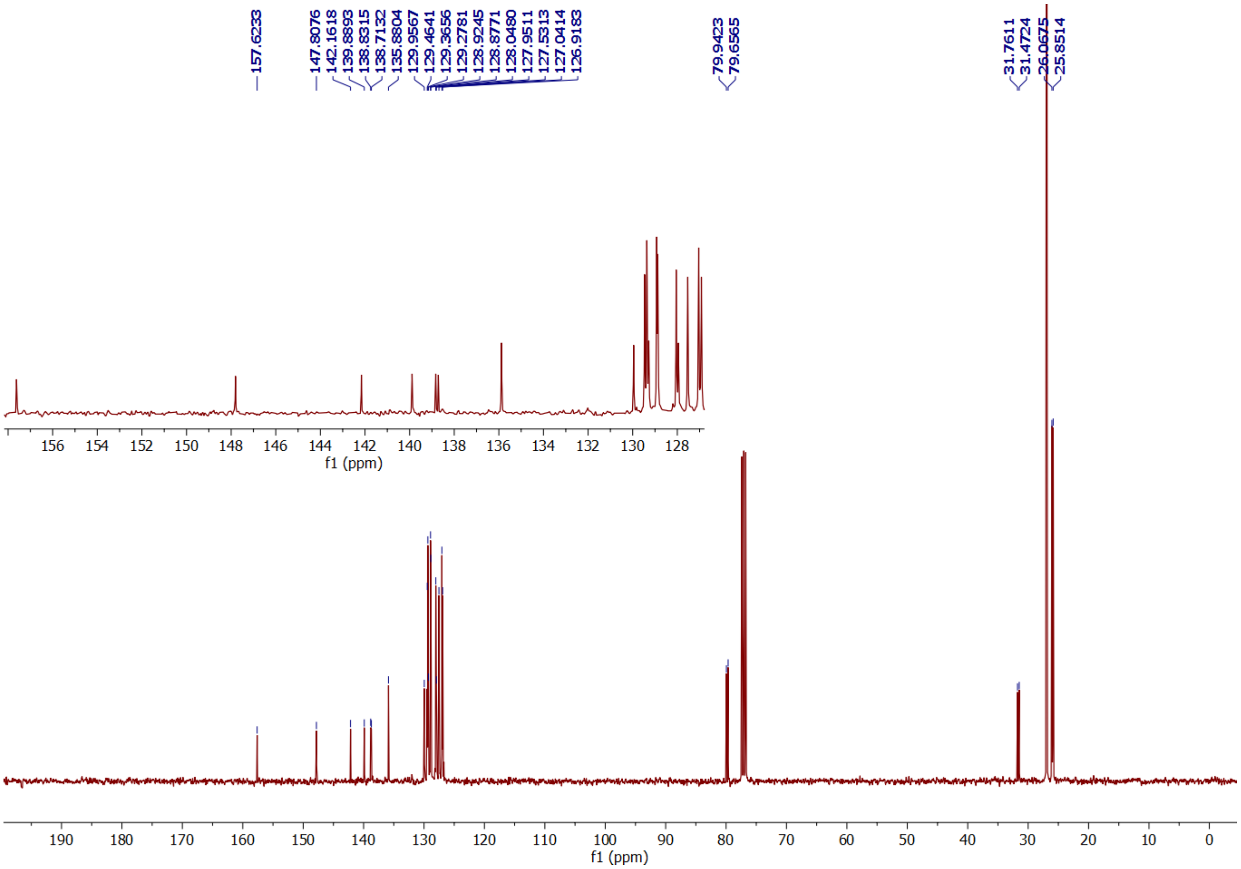

^{1}H NMR (400 MHz, $CDCl_3$) of **4g** (see procedure)

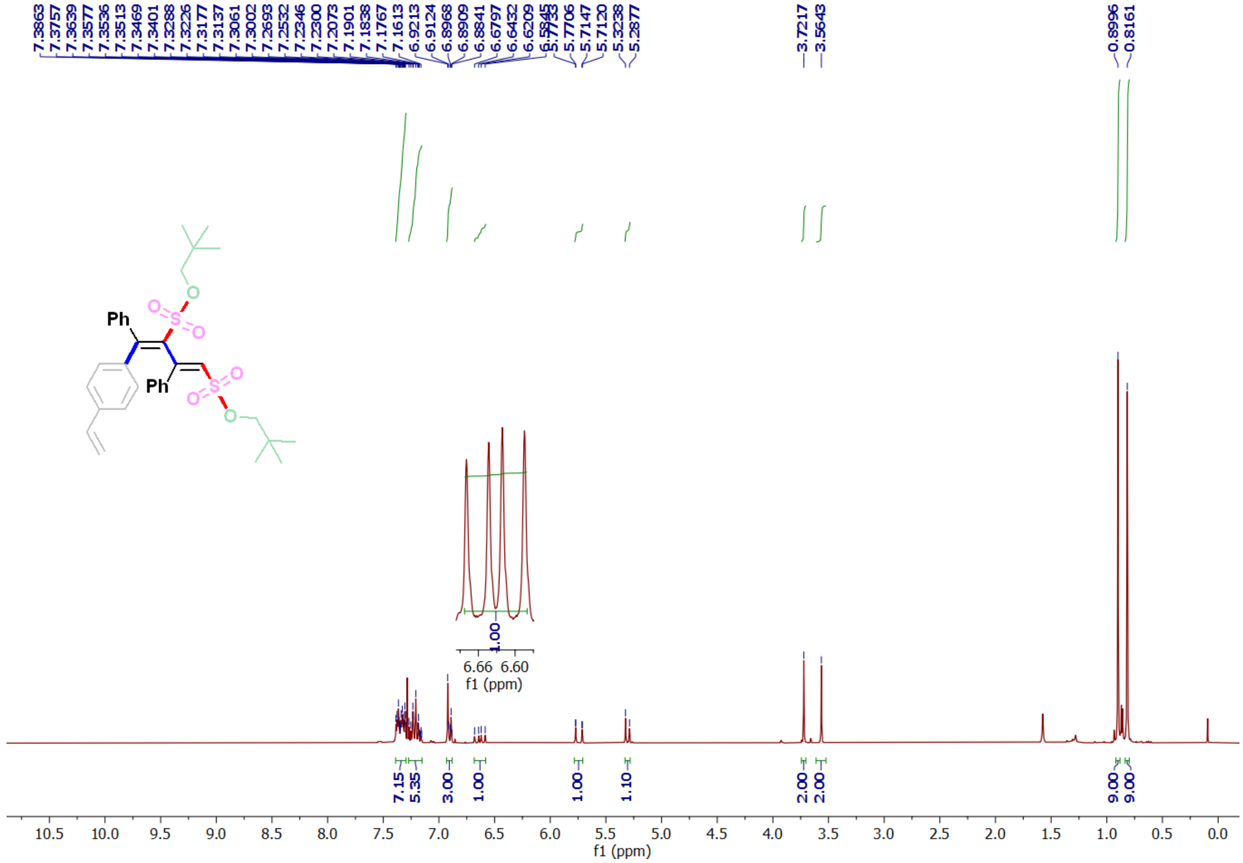

^{13}C NMR (101MHz, $CDCl_3$) of **4g**

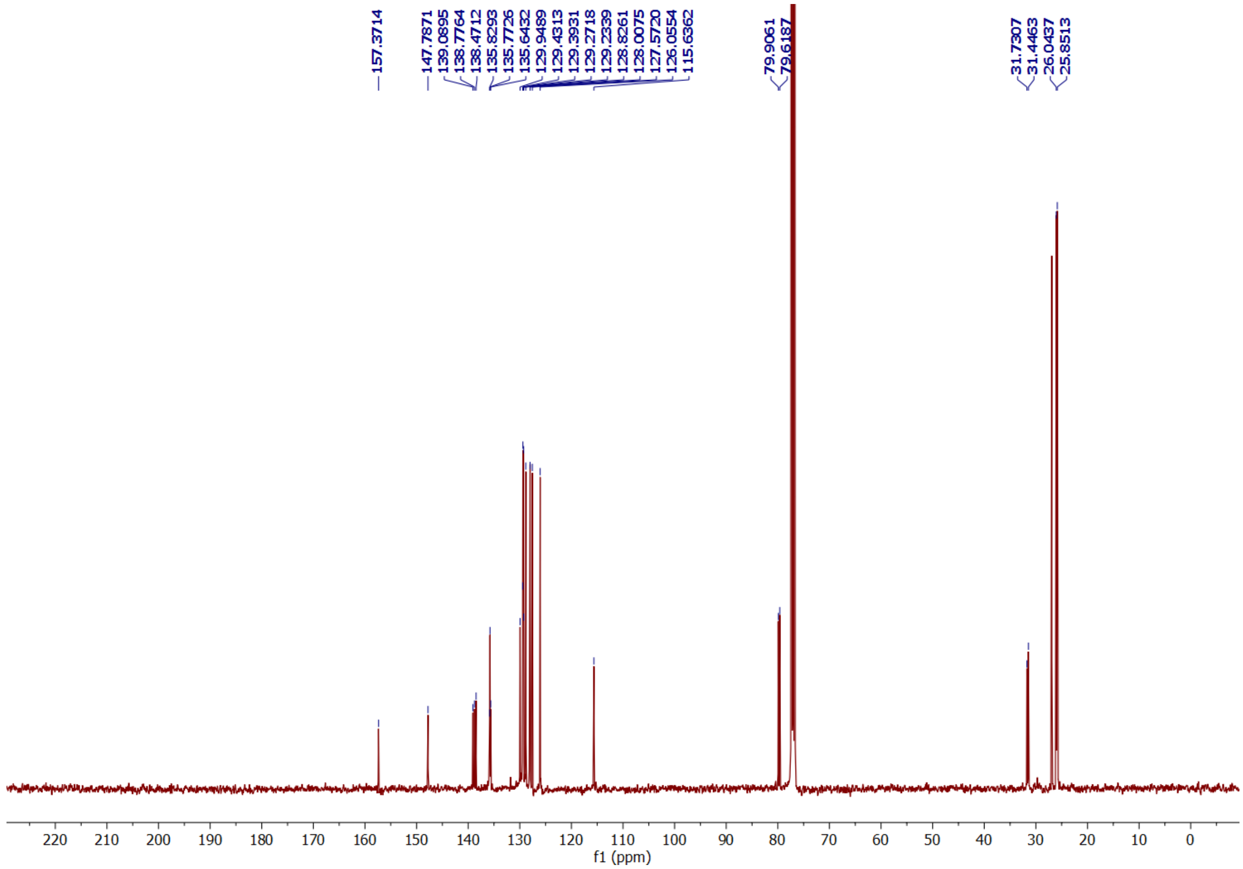

^{1}H NMR (400 MHz, $CDCl_3$) of **4h** (see procedure)

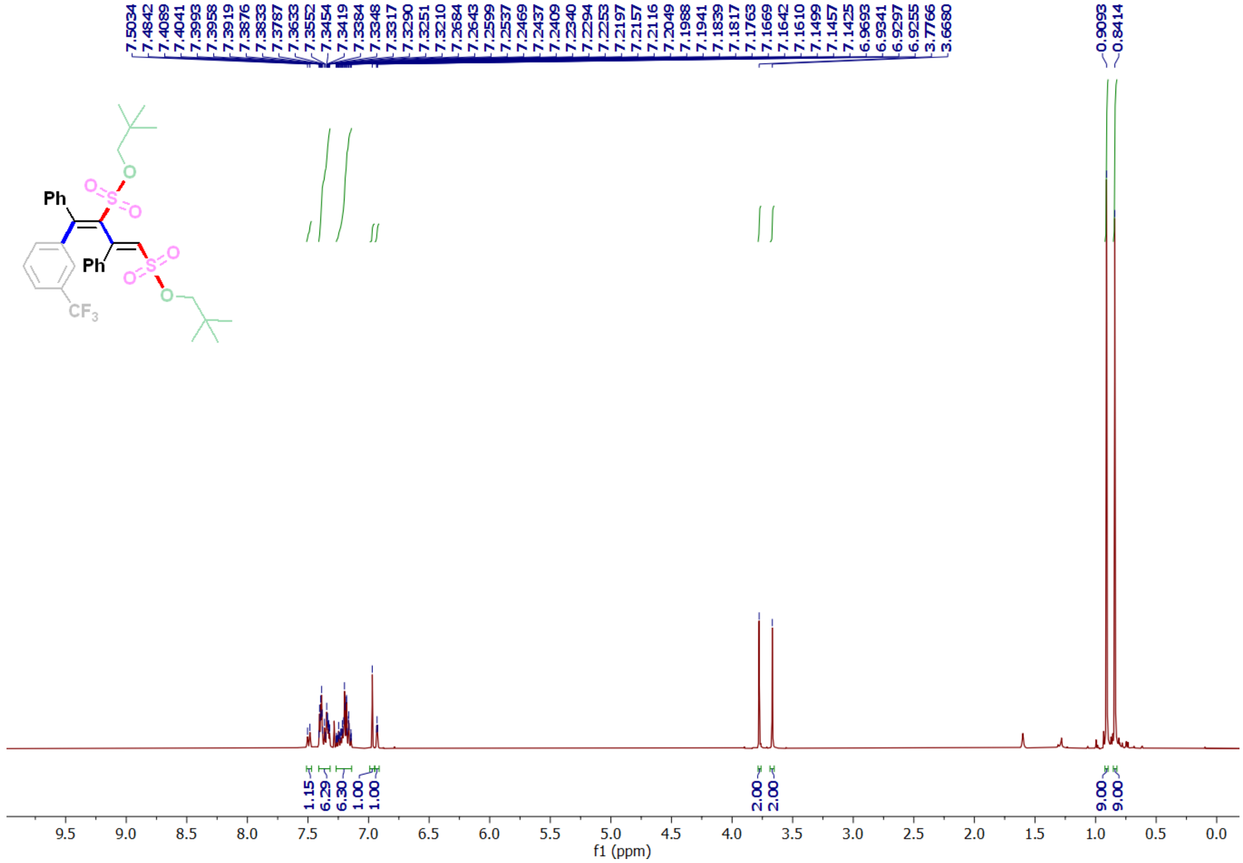

^{13}C NMR (101MHz, $CDCl_3$) of **4h**

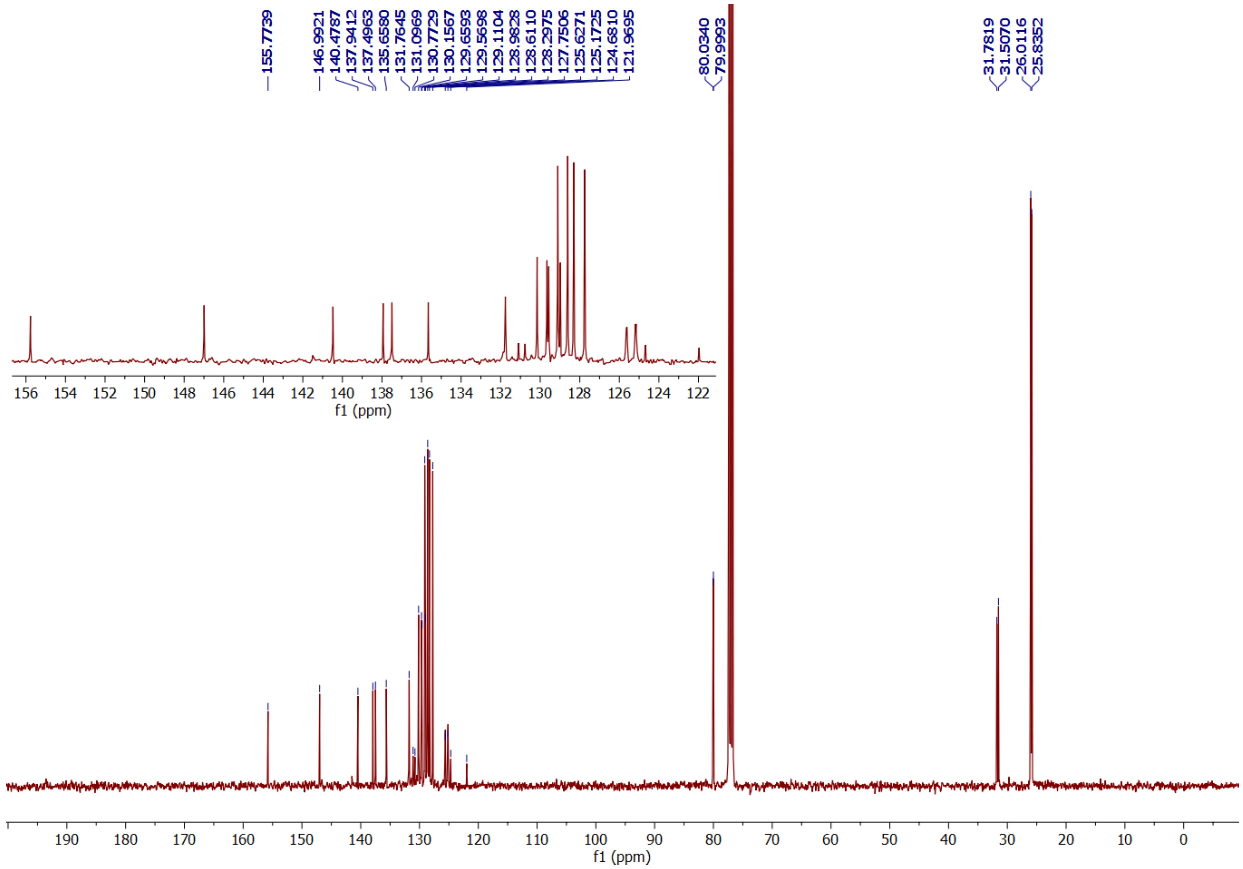

^{19}F NMR (282 MHz, $CDCl_3$) of **4h**

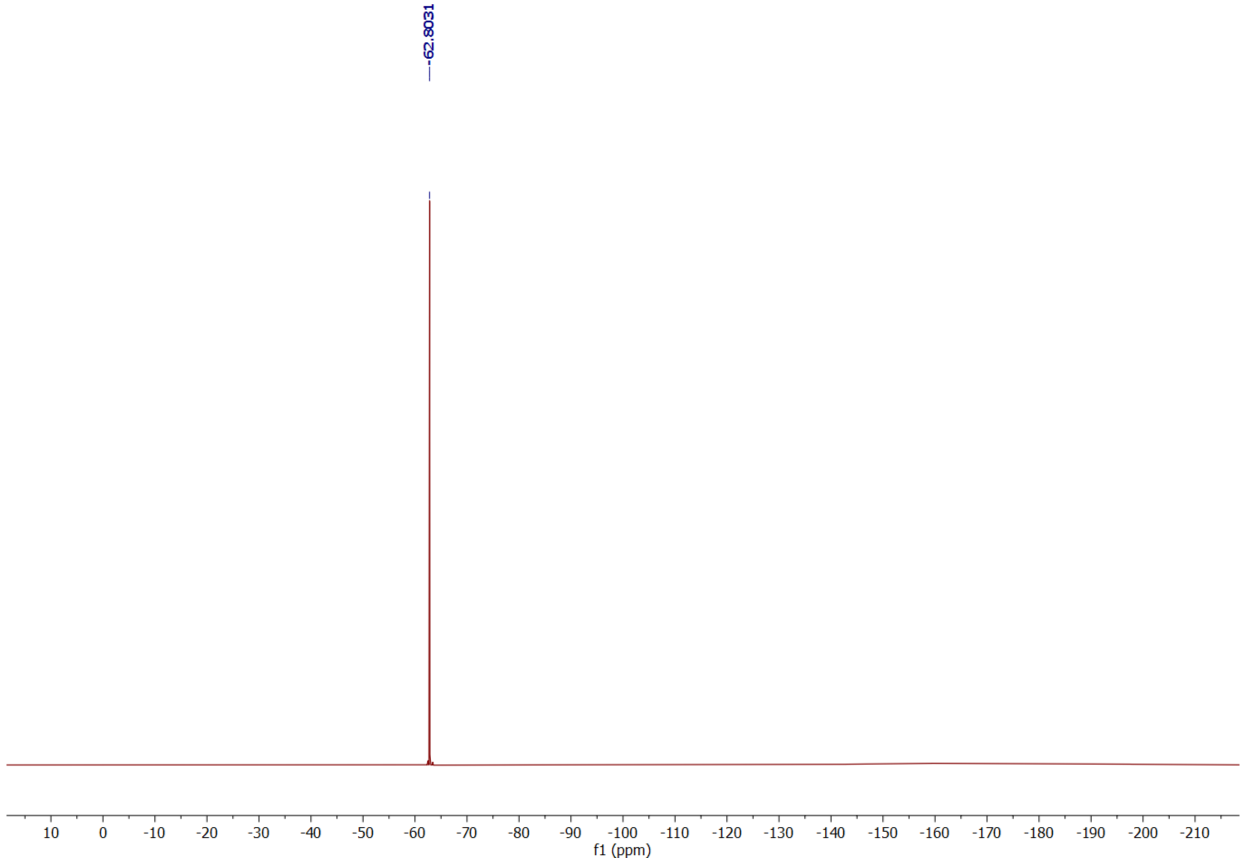

^{1}H NMR (400 MHz, $CDCl_3$) of **4i** (see procedure)

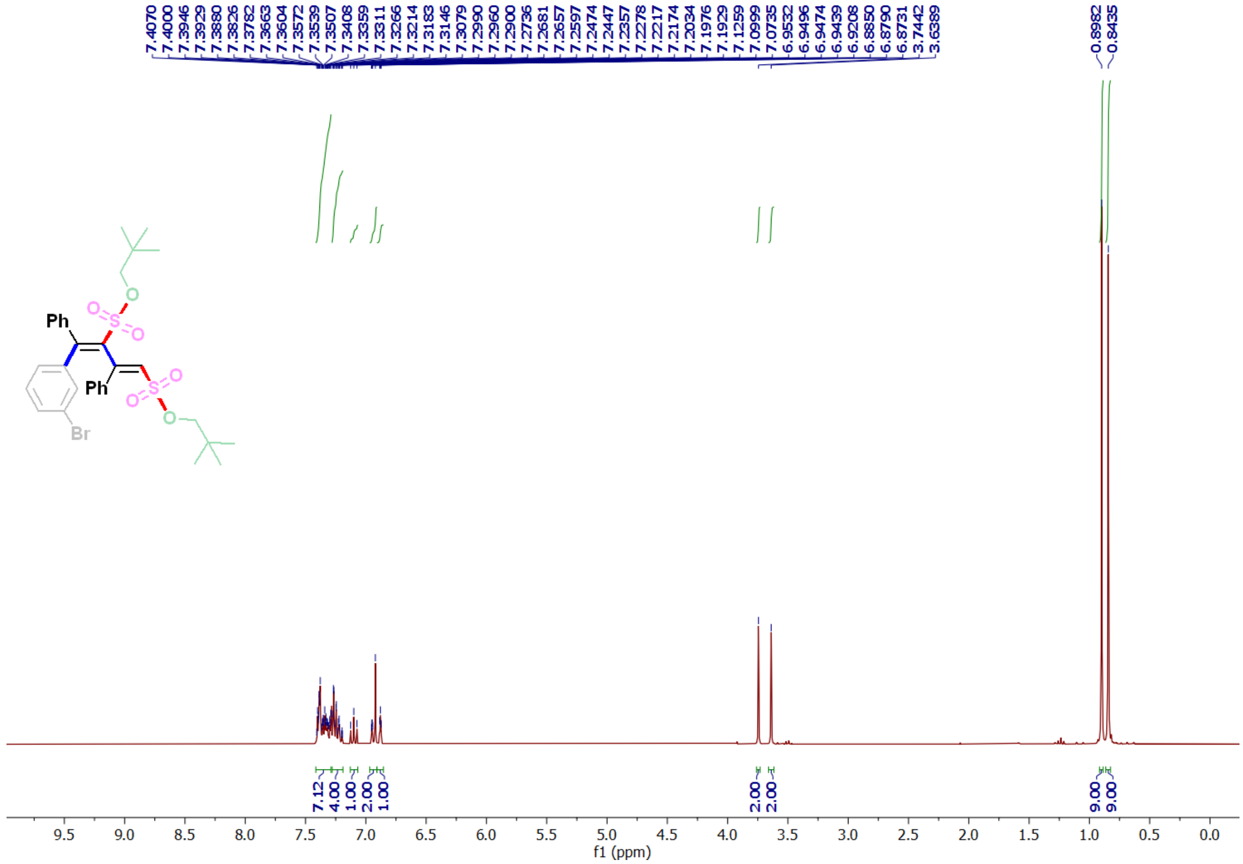

^{13}C NMR (101MHz, $CDCl_3$) of **4i**

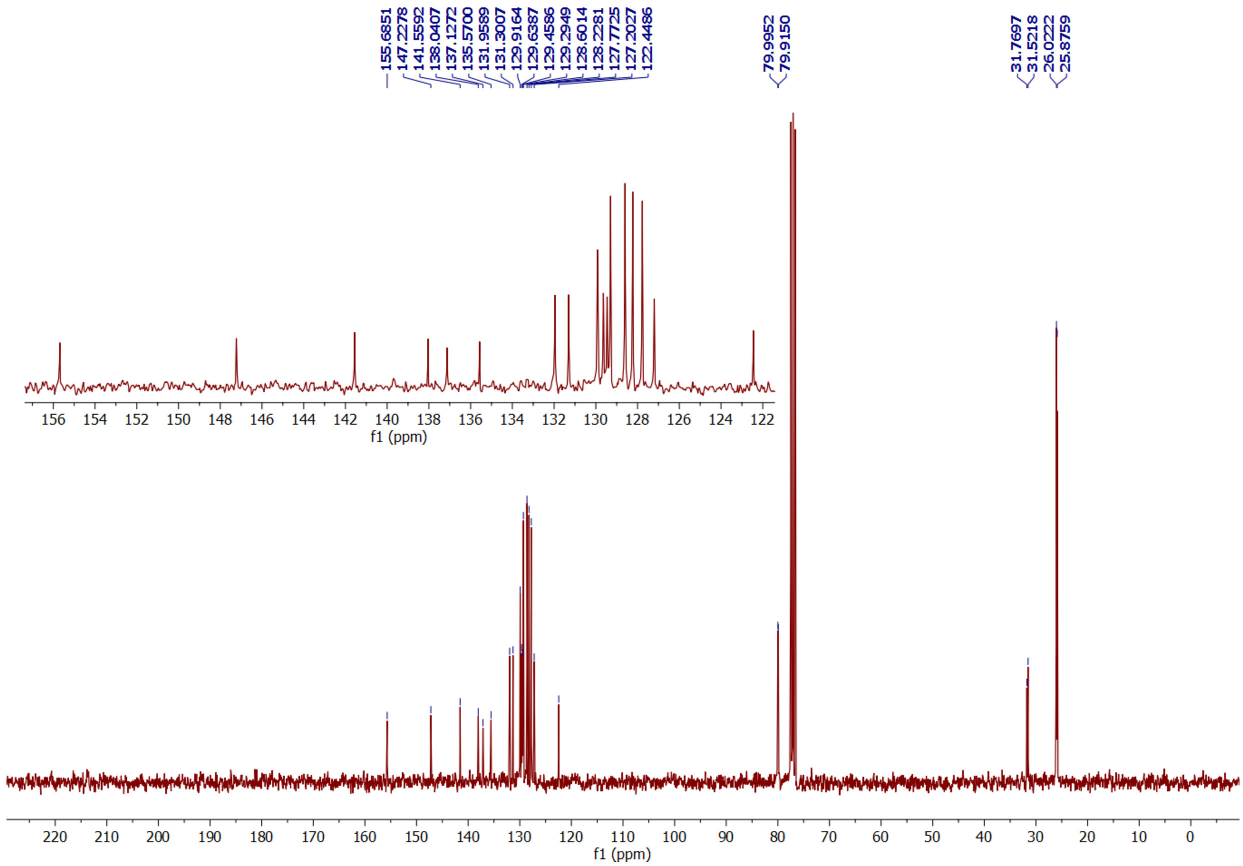

^{1}H NMR (300 MHz, $CDCl_3$) of **4j** (see procedure)

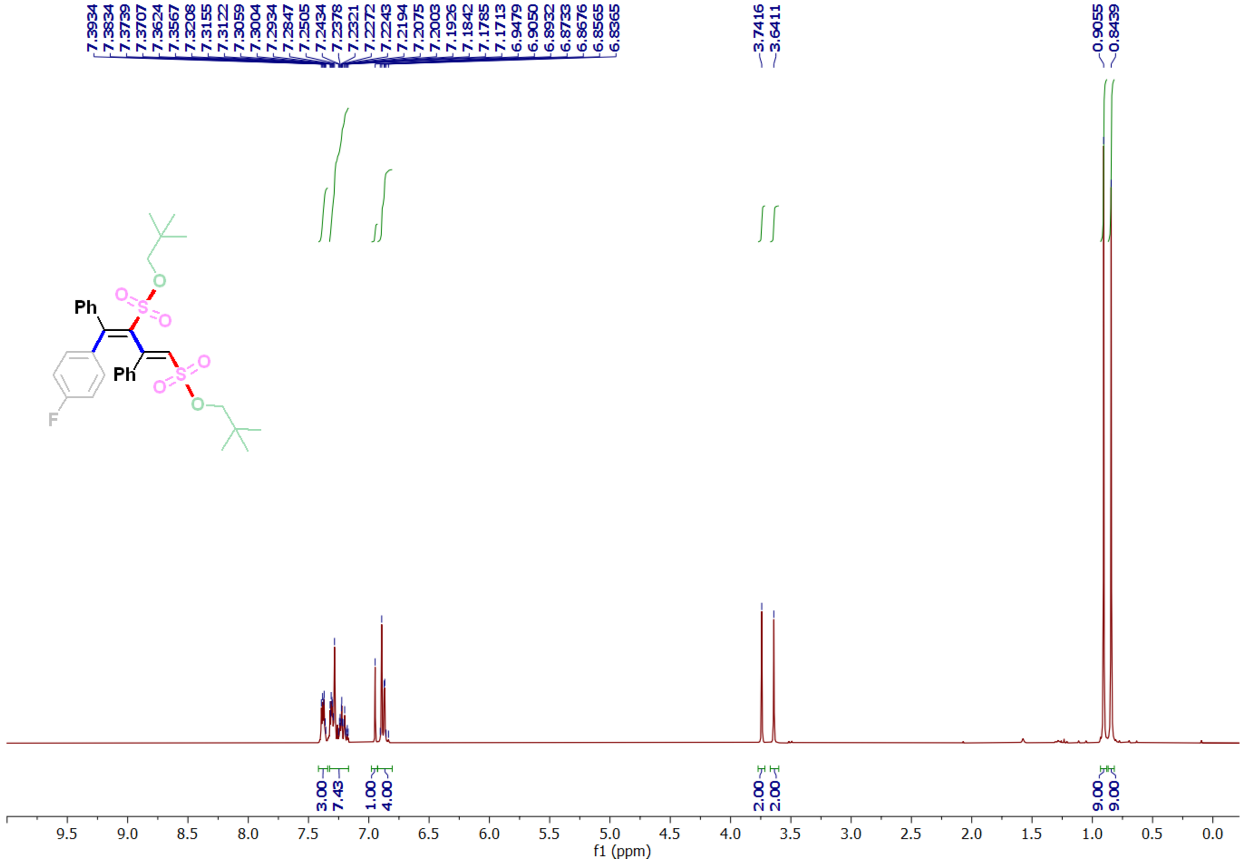

^{13}C NMR (75 MHz, $CDCl_3$) of **4j**

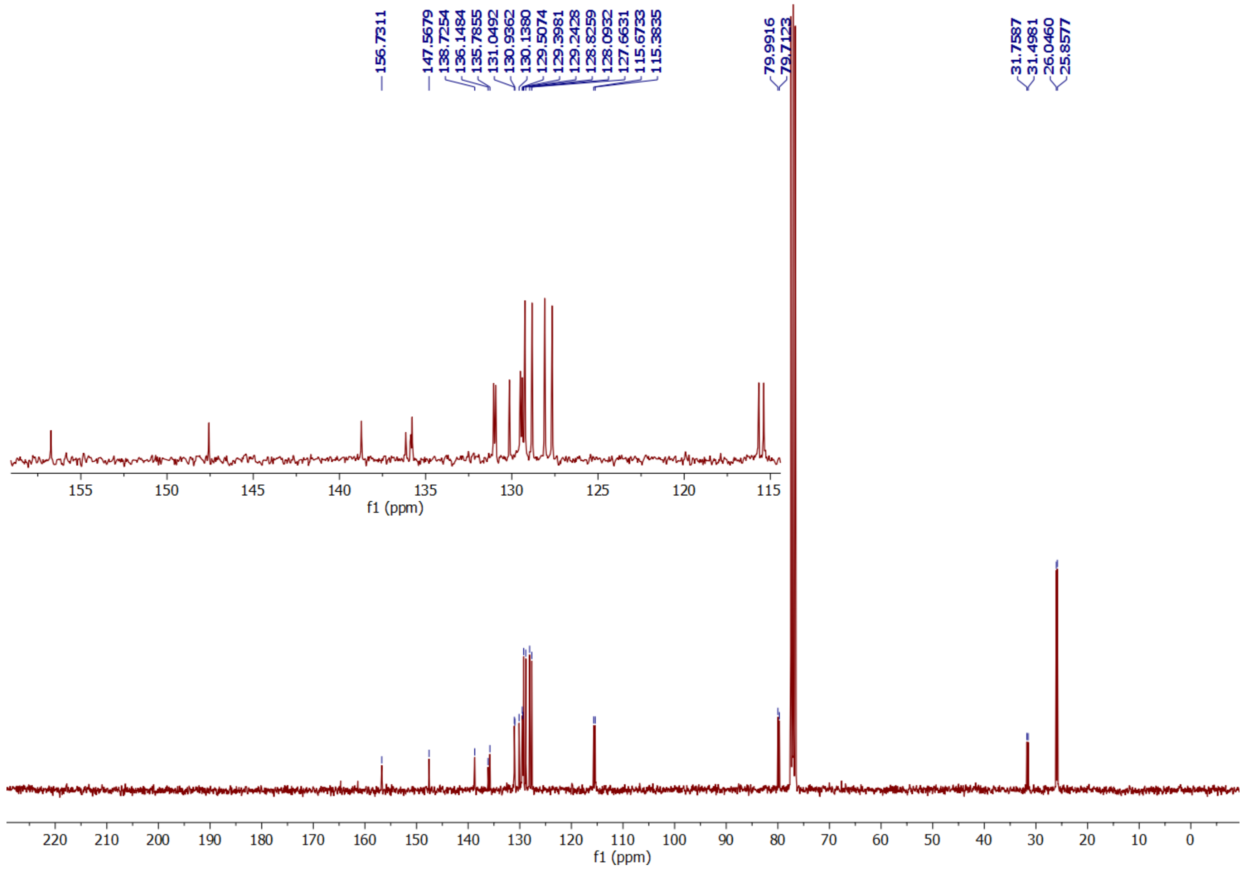

^{19}F NMR (282 MHz, $CDCl_3$) of **4j**

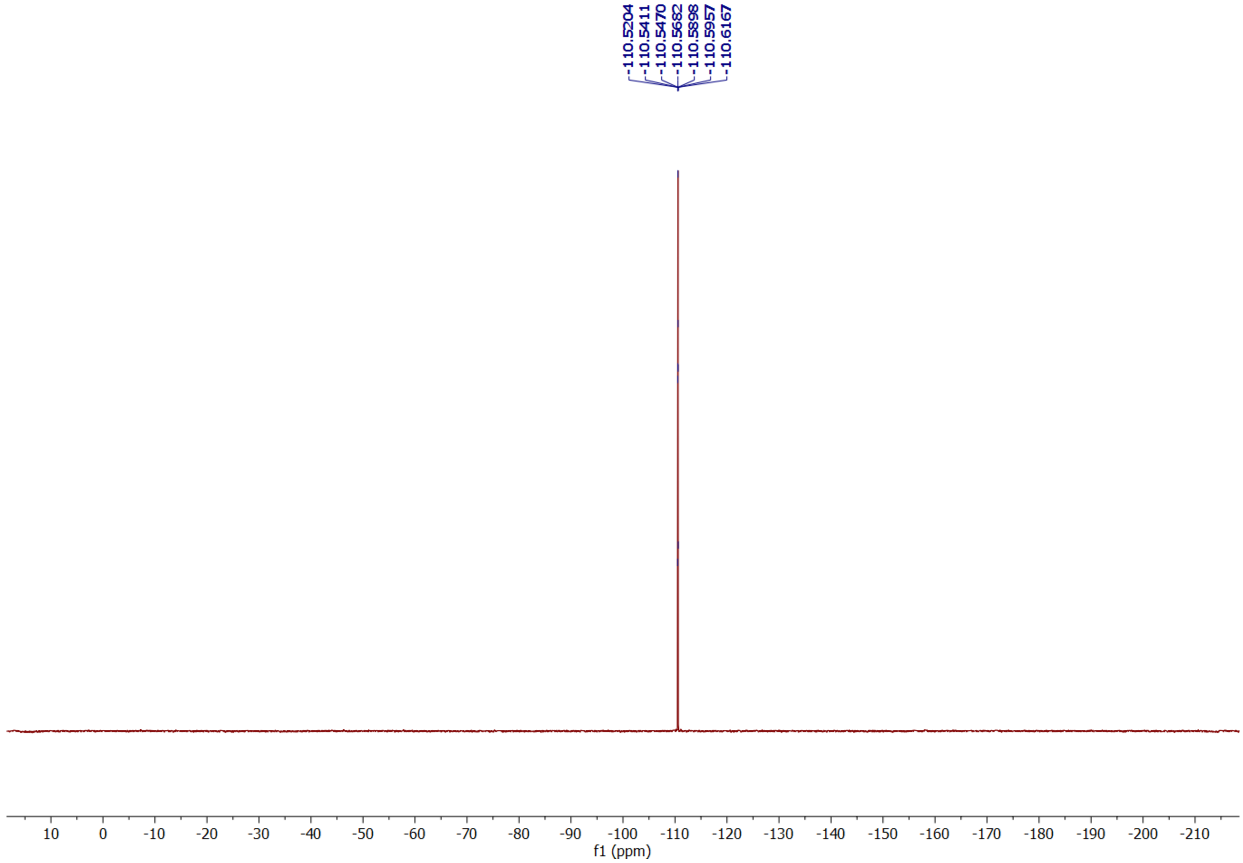

^{1}H NMR (400 MHz, $CDCl_3$) of **4k** (see procedure)

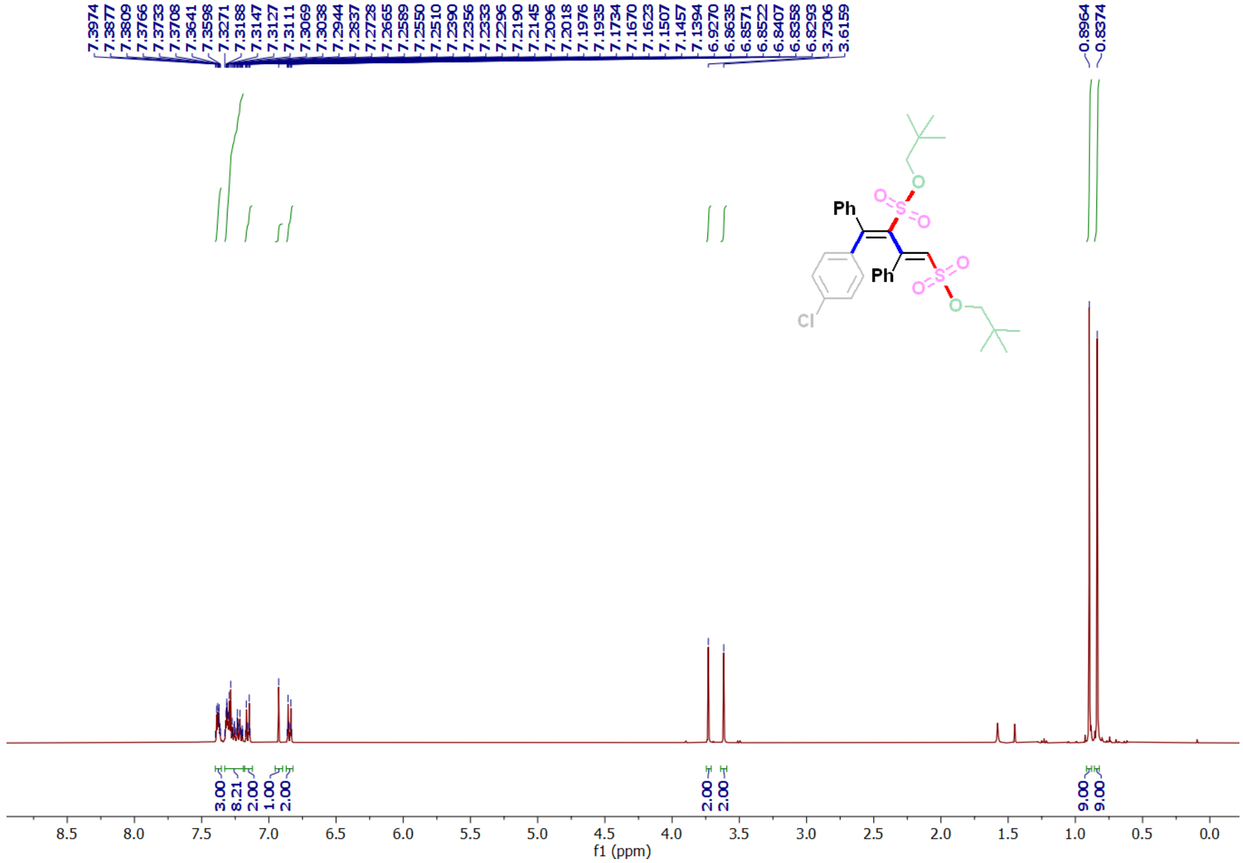

^{13}C NMR (101MHz, $CDCl_3$) of **4k**

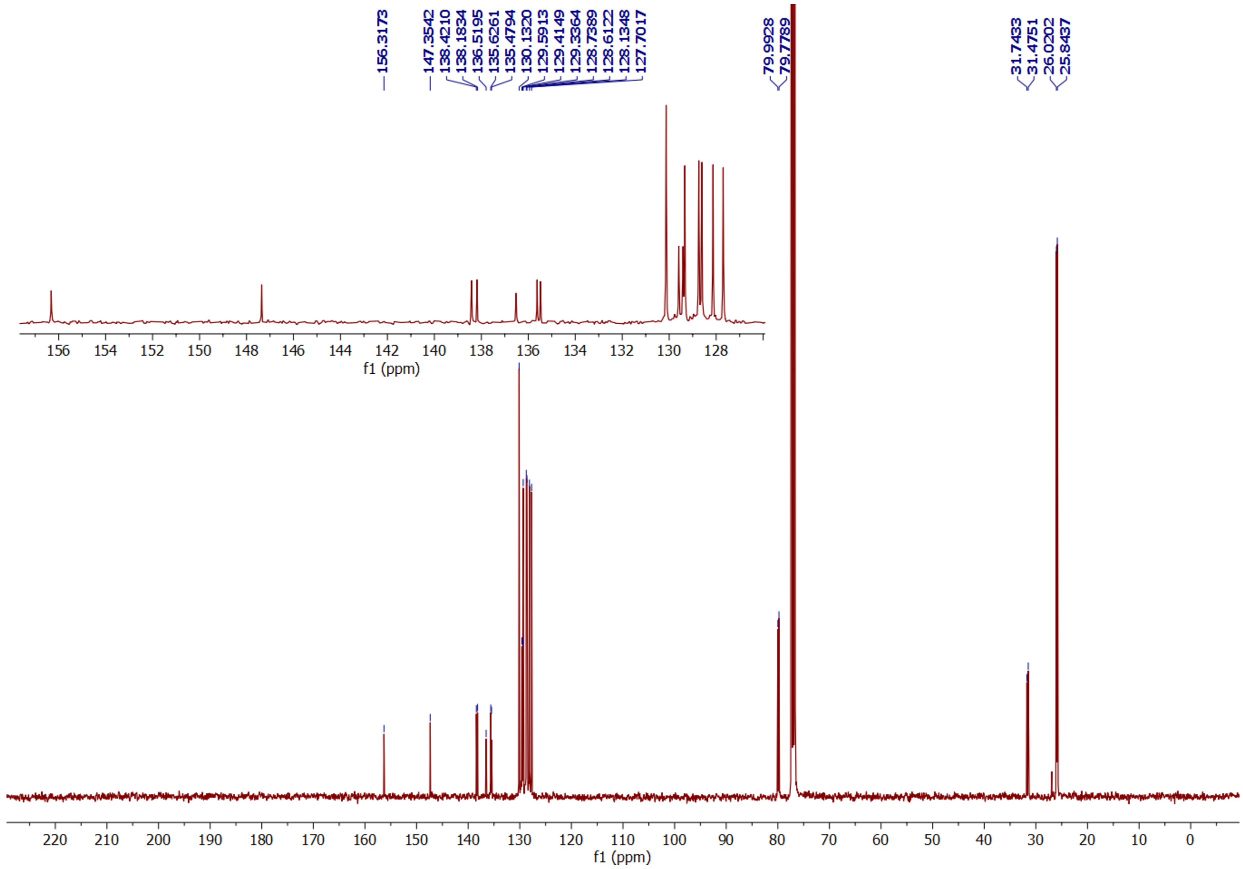

^{1}H NMR (400 MHz, $CDCl_3$) of **4l** (see procedure)

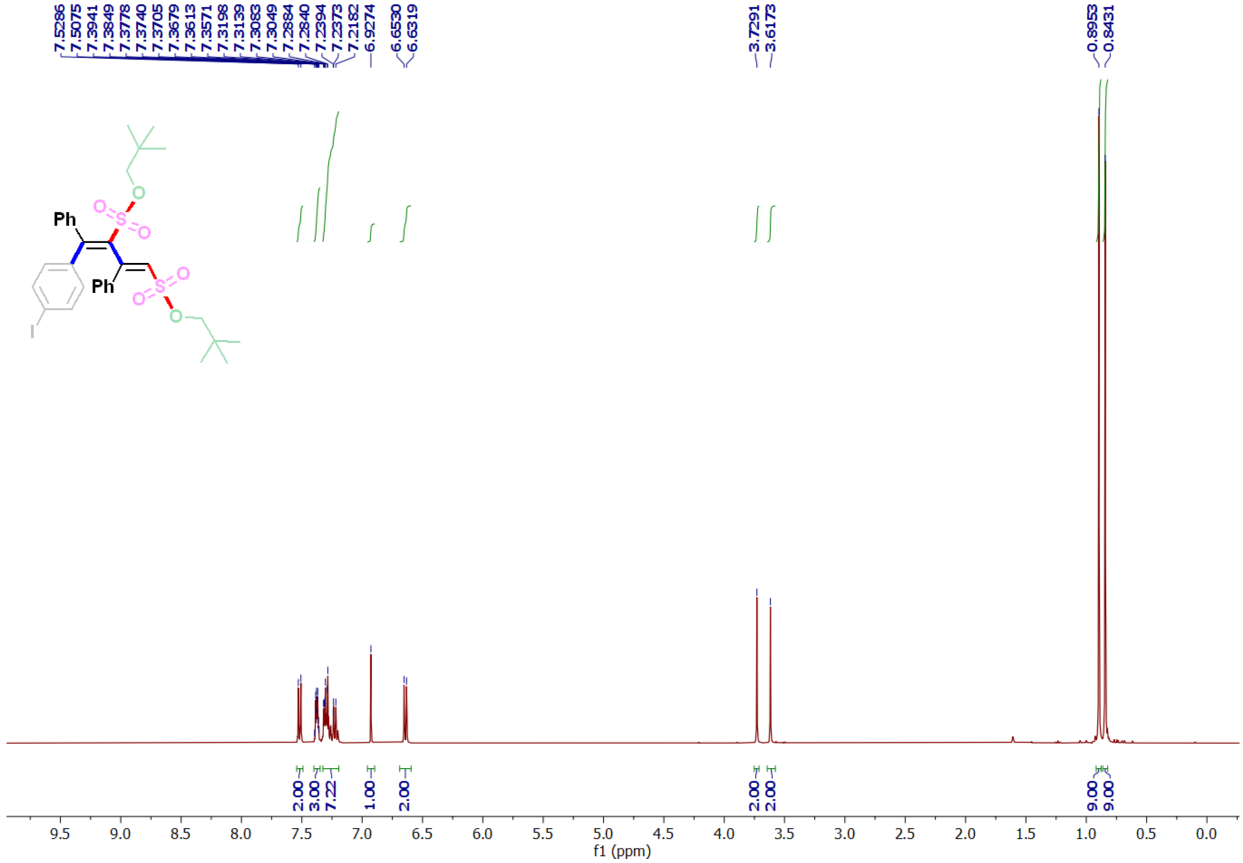

^{13}C NMR (101MHz, $CDCl_3$) of **4l**

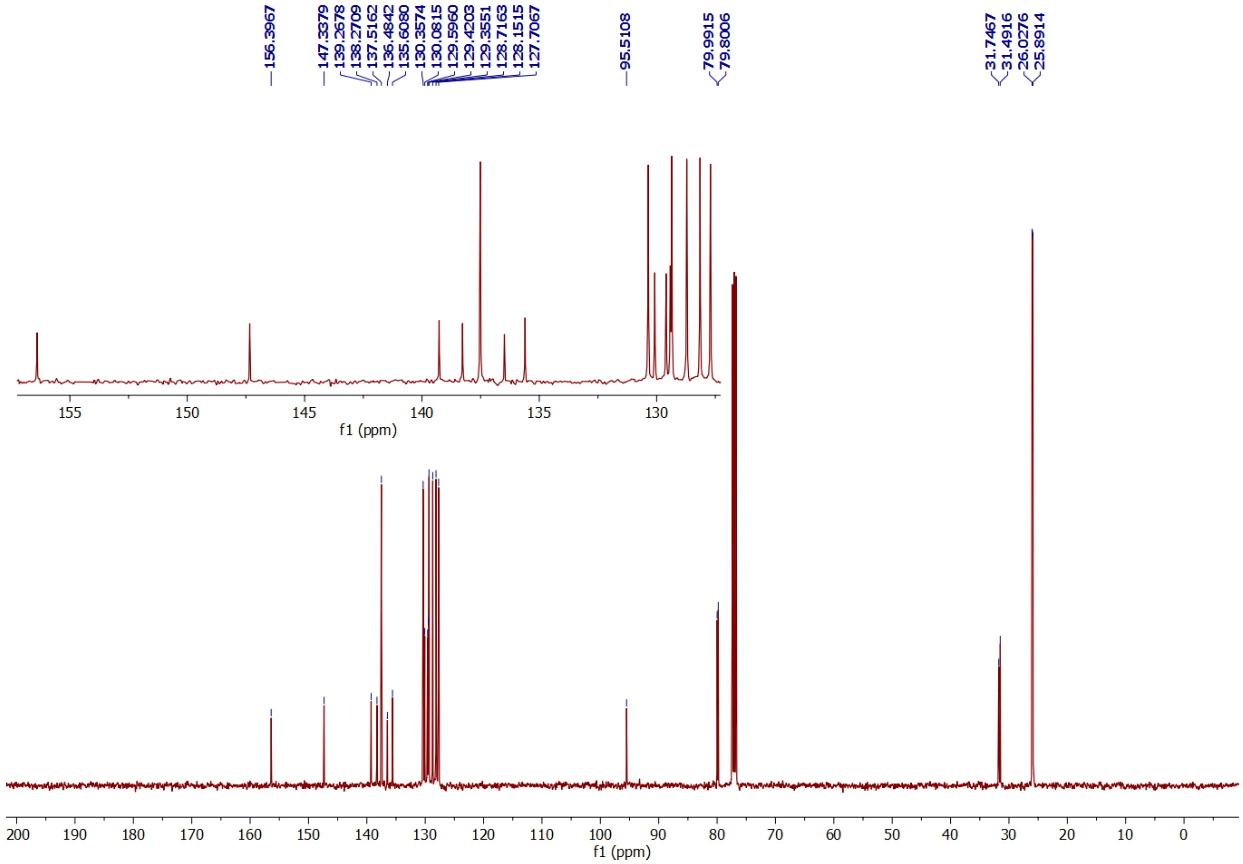

^1H NMR (300 MHz, $CDCl_3$) of **4l-D1** (see procedure)

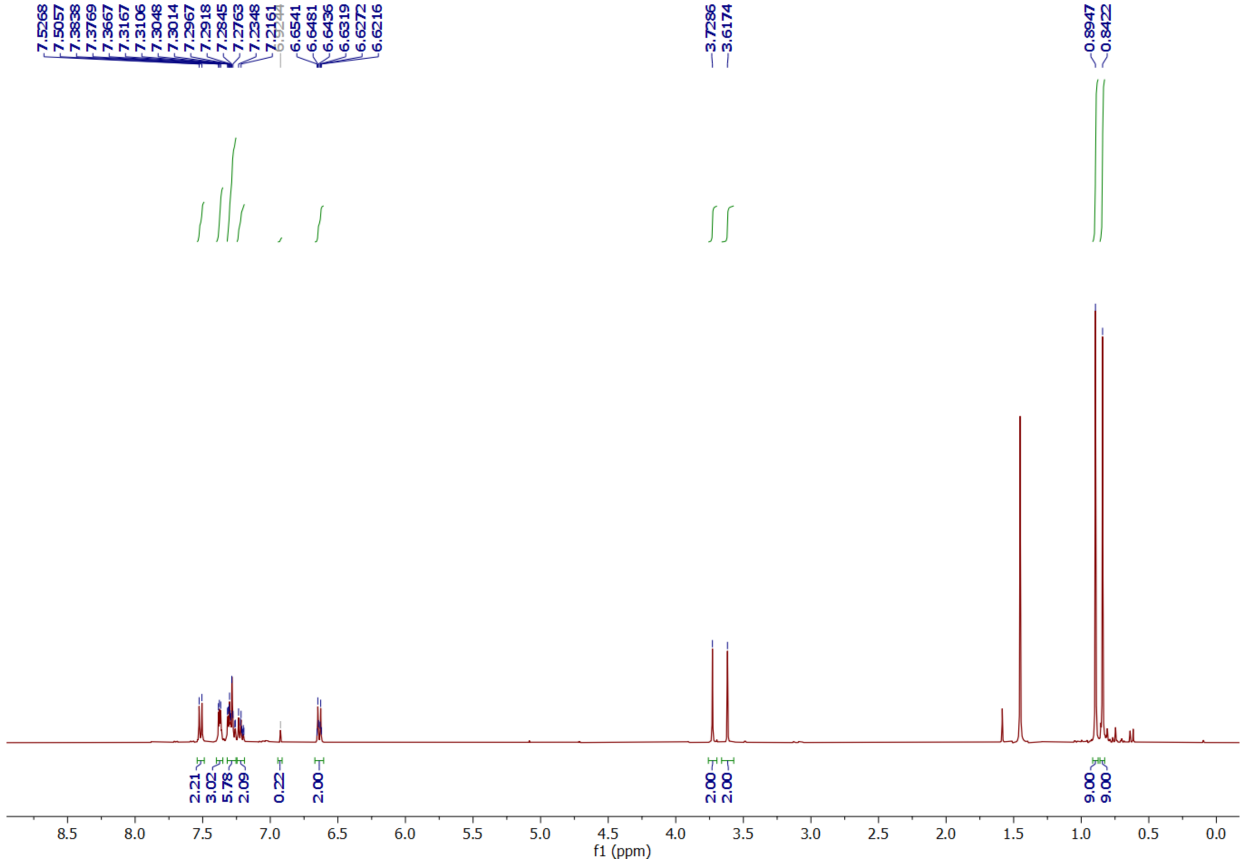

^1H NMR (300 MHz, $CDCl_3$) of **4l-D2** (see procedure)

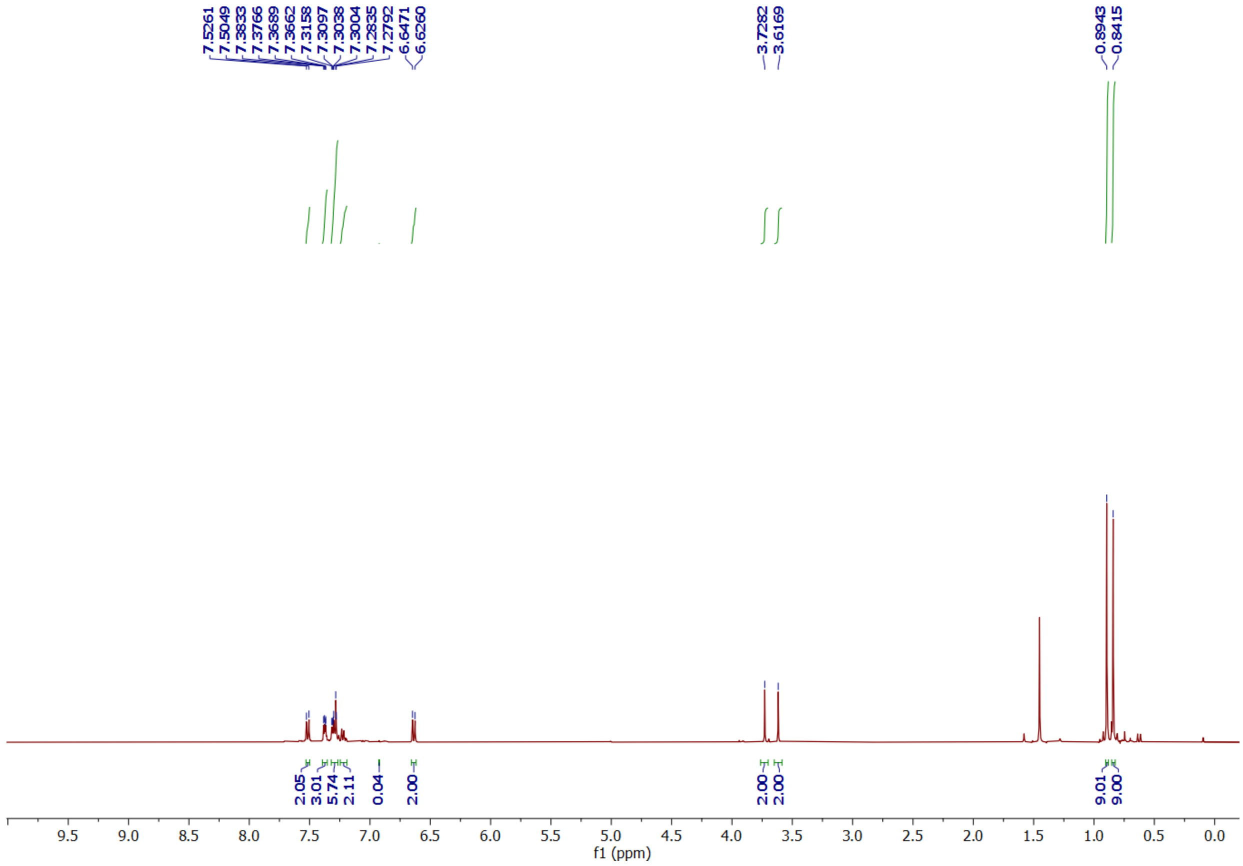

^{1}H NMR (400 MHz, $CDCl_3$) of **4m** (see procedure)

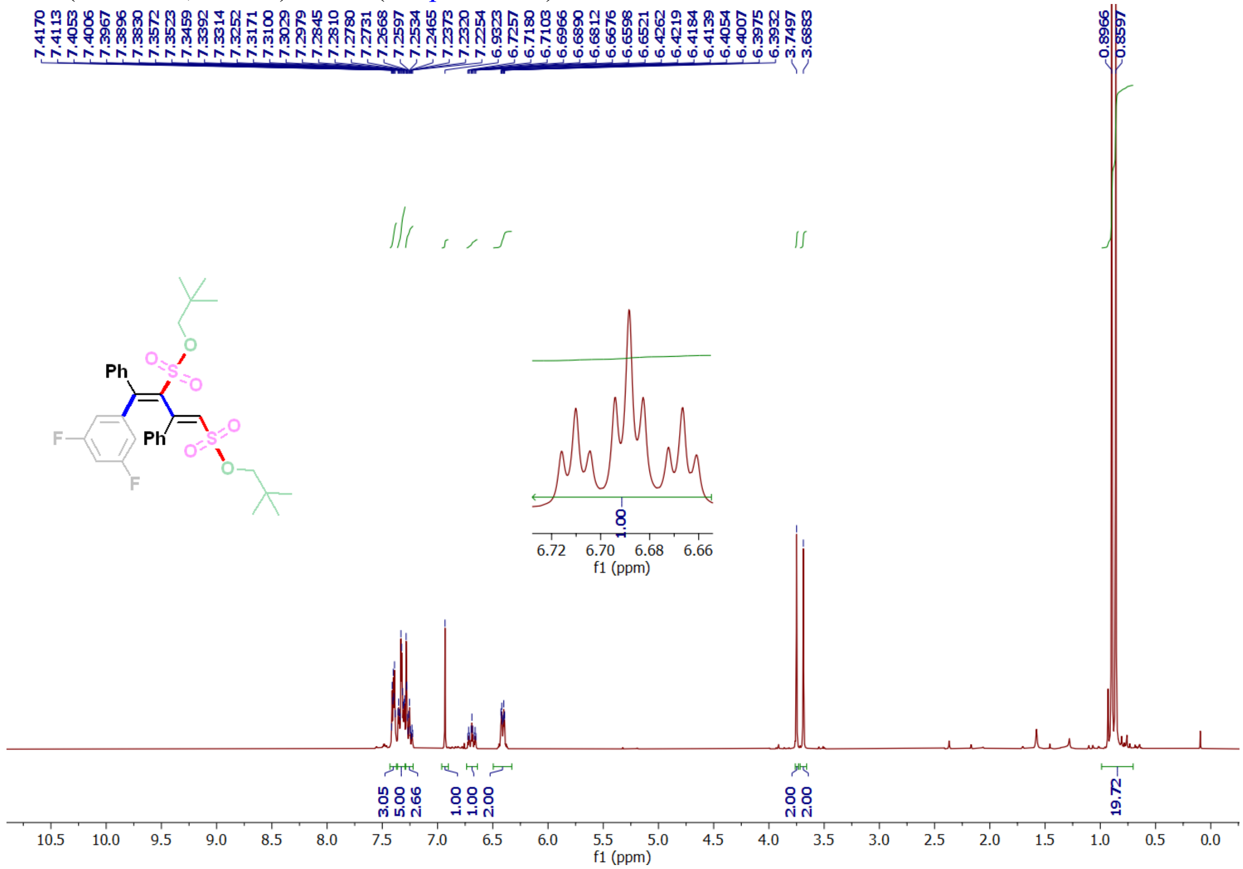

^{13}C NMR (101MHz, $CDCl_3$) of **4m**

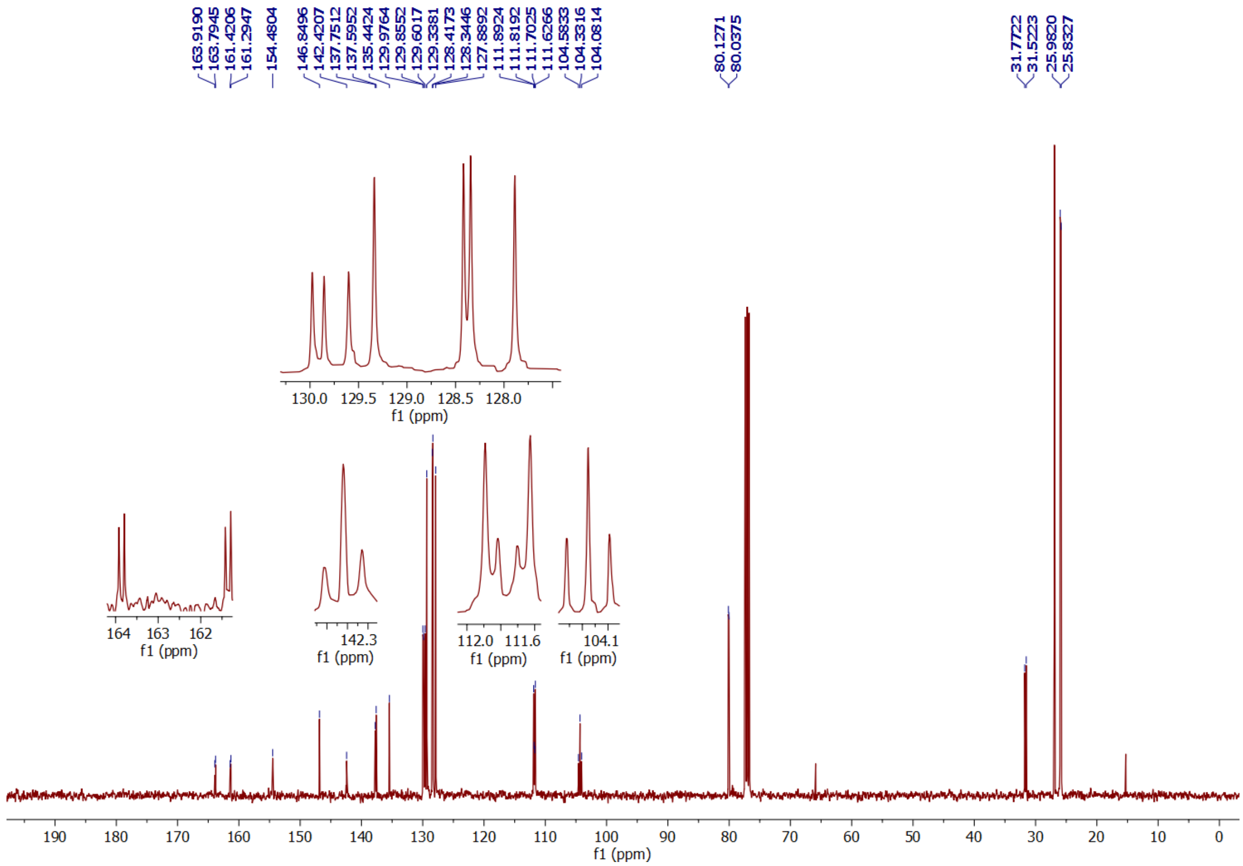

^{19}F NMR (282 MHz, $CDCl_3$) of **4m**

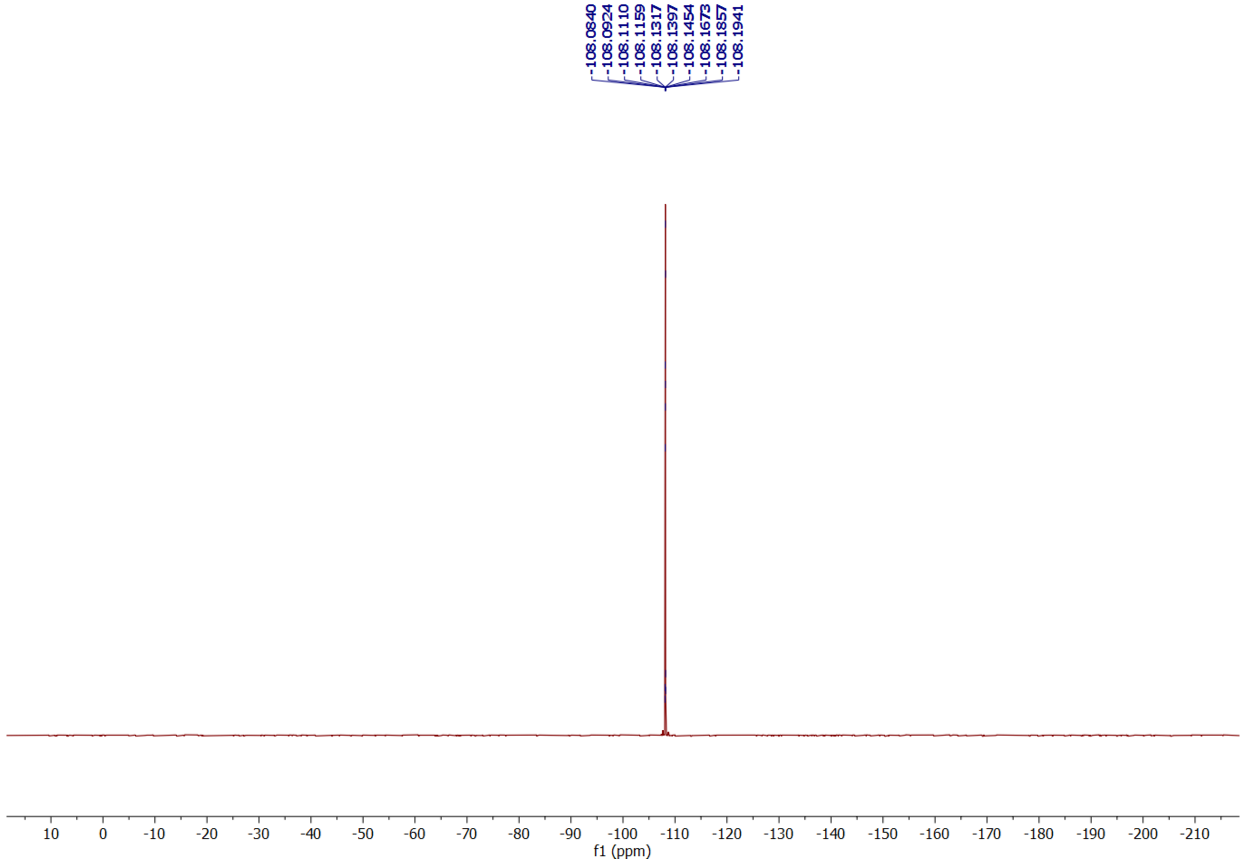

^{1}H NMR (400 MHz, $CDCl_3$) of **4n** (see procedure)

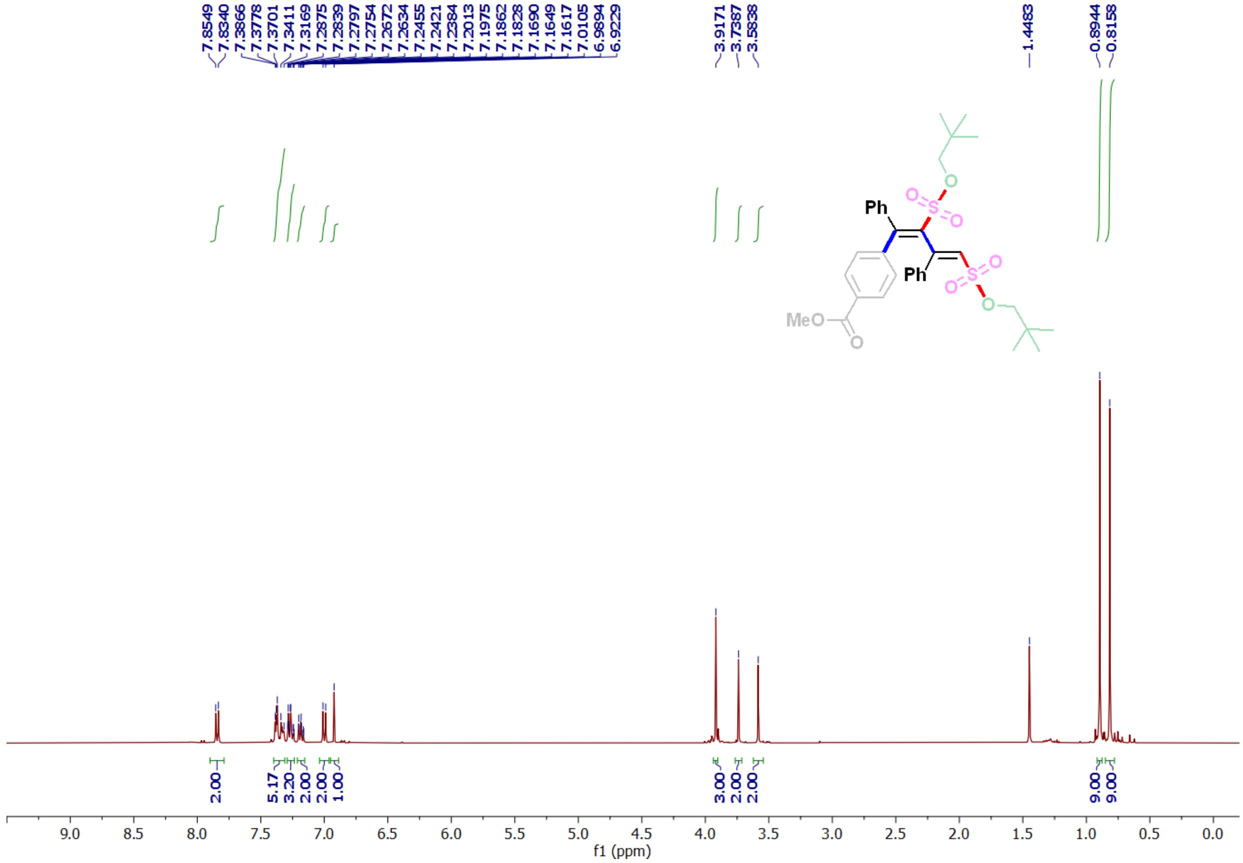

^{13}C NMR (101MHz, $CDCl_3$) of **4n**

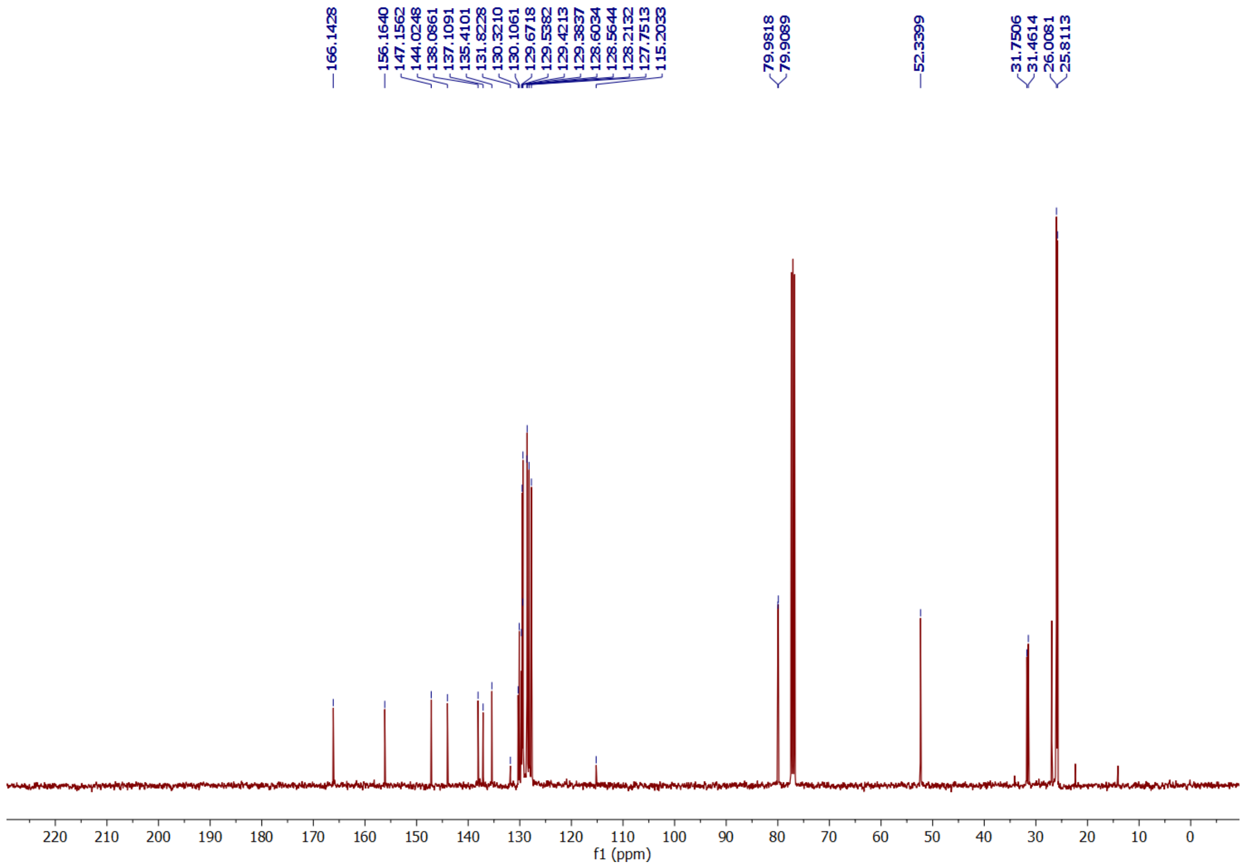

^{1}H NMR (400 MHz, $CDCl_3$) of **4o** (see procedure)

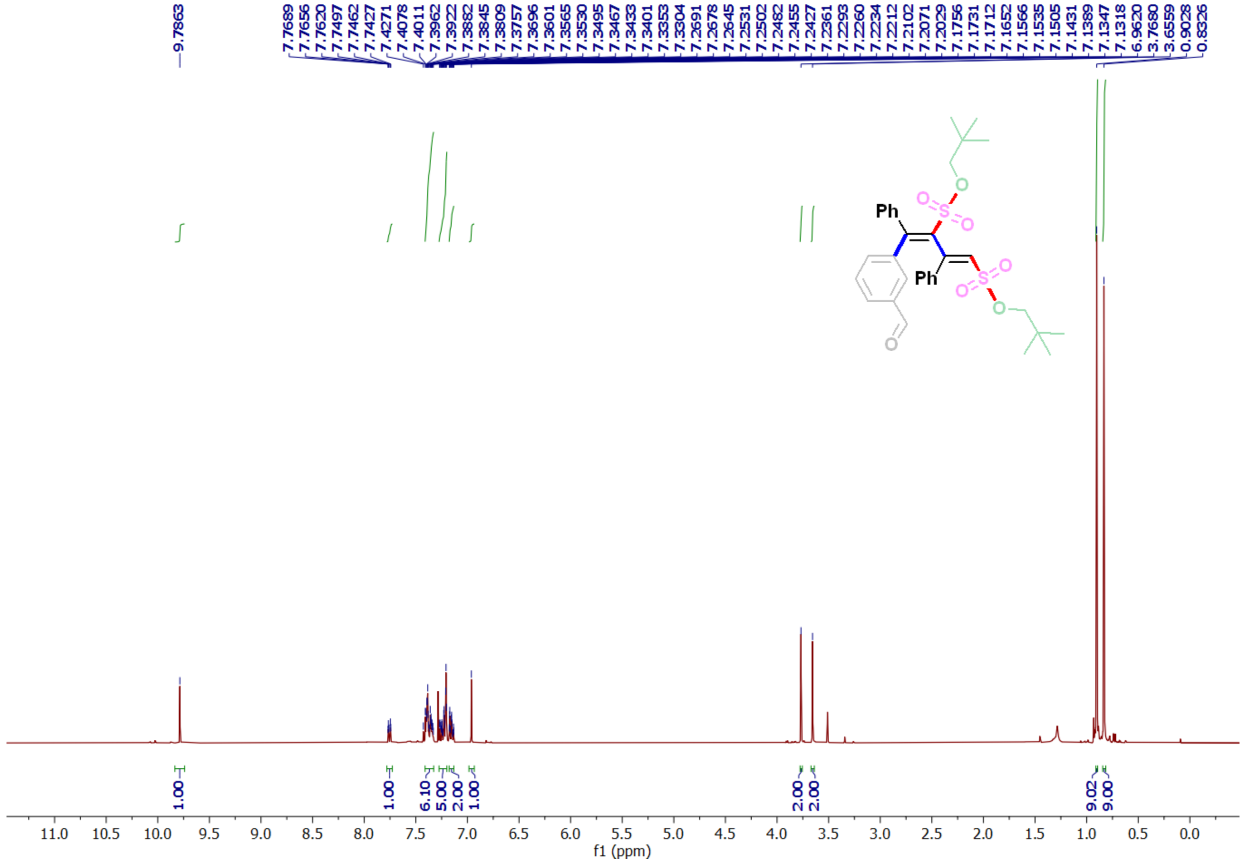

^{13}C NMR (101MHz, $CDCl_3$) of **4o**

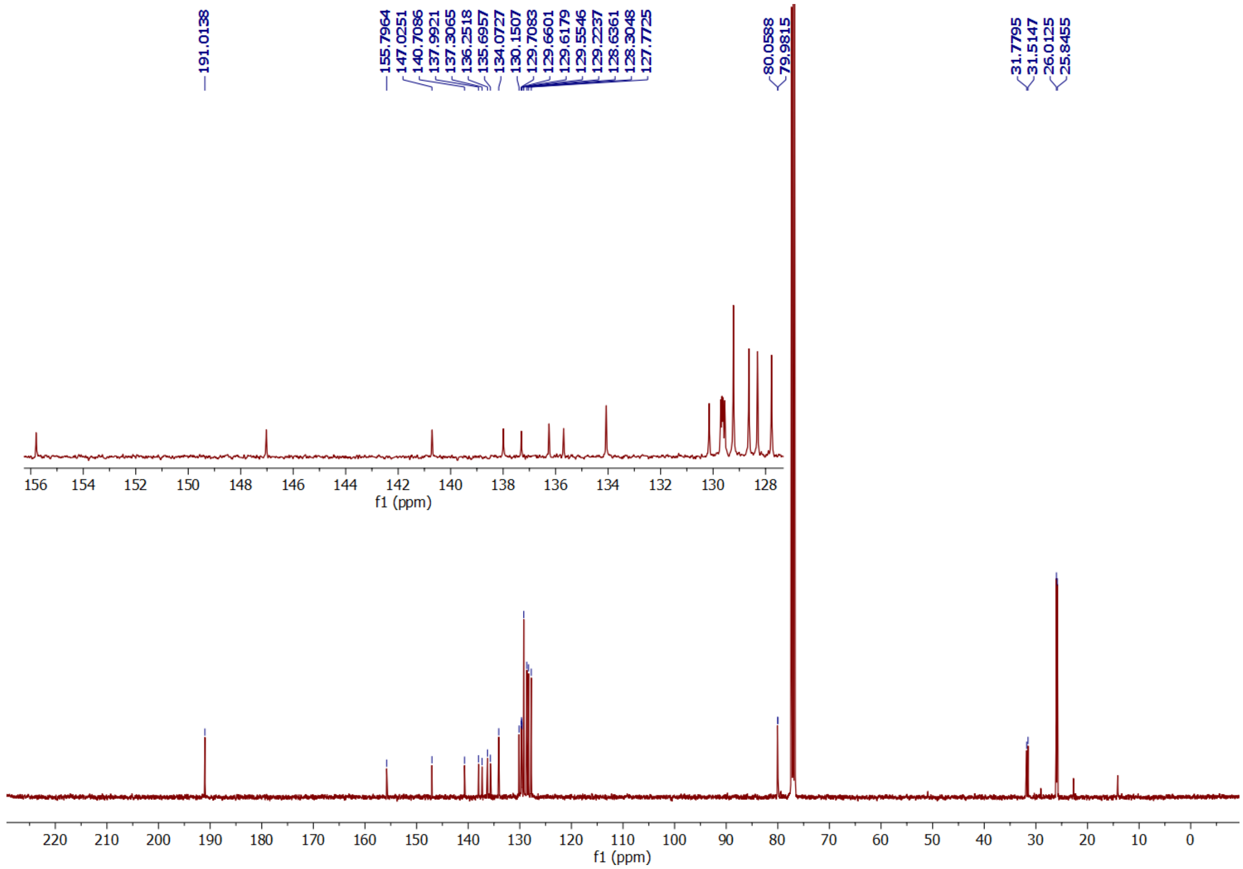

^{1}H NMR (400 MHz, $CDCl_3$) of **4p** (see procedure)

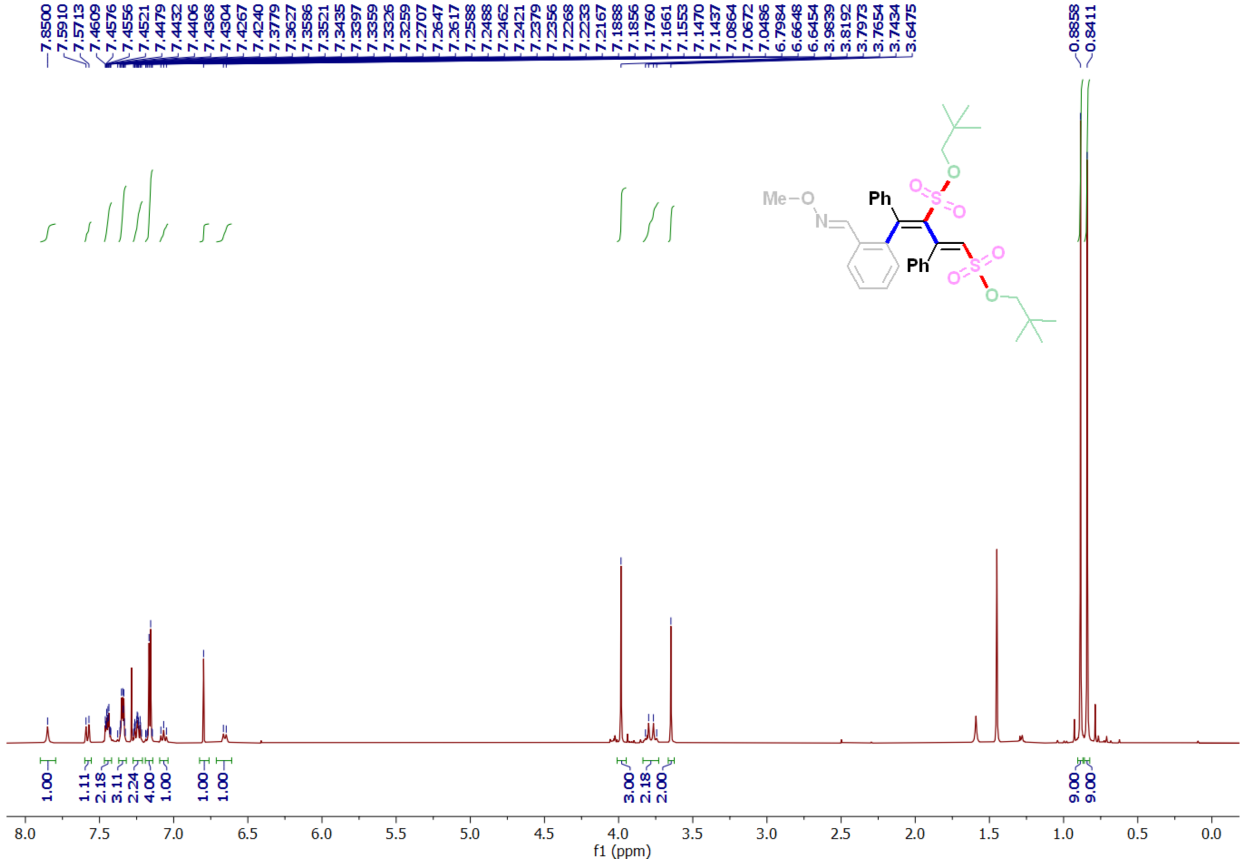

^{13}C NMR (101MHz, $CDCl_3$) of **4p**

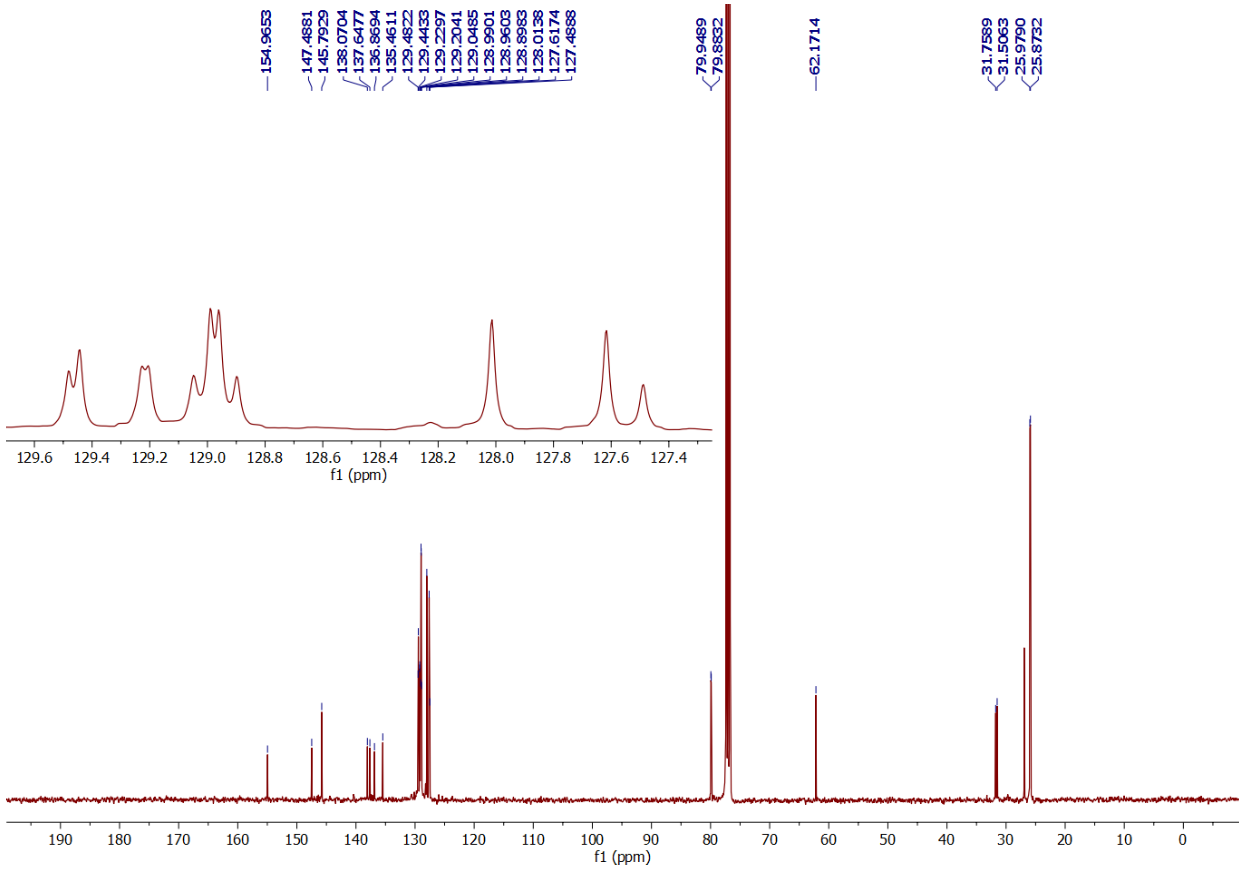

^{1}H NMR (400 MHz, $CDCl_3$) of **4q** (see procedure)

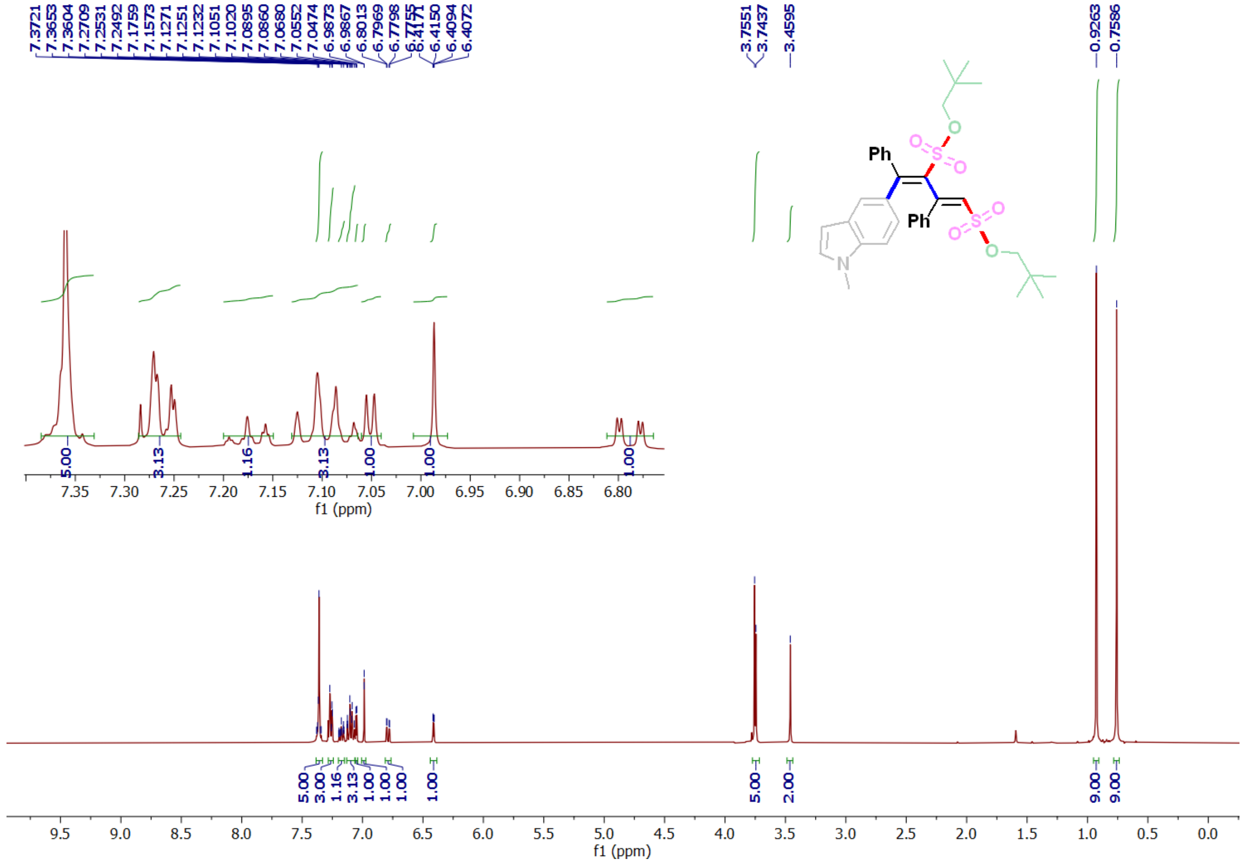

^{13}C NMR (101MHz, $CDCl_3$) of **4q**

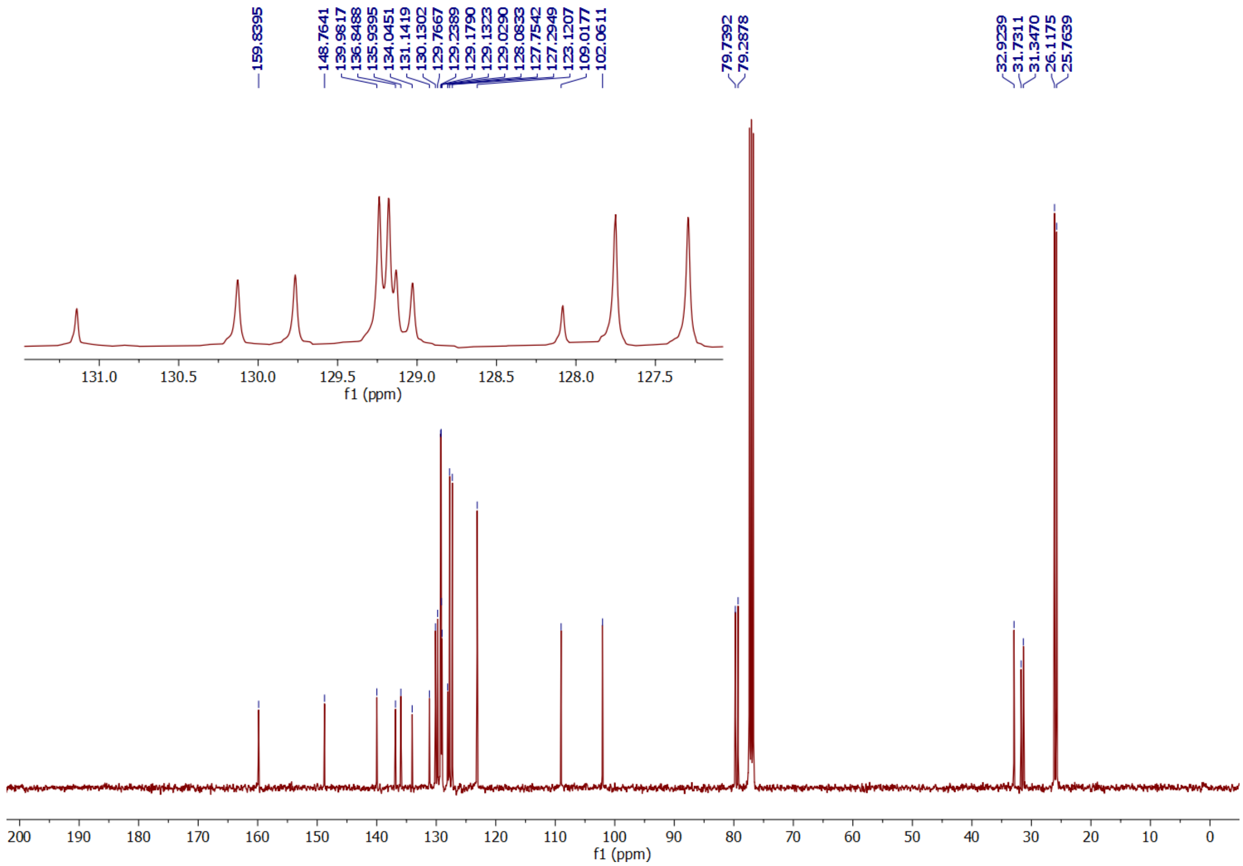

^{1}H NMR (400 MHz, $CDCl_3$) of **4r** (see procedure)

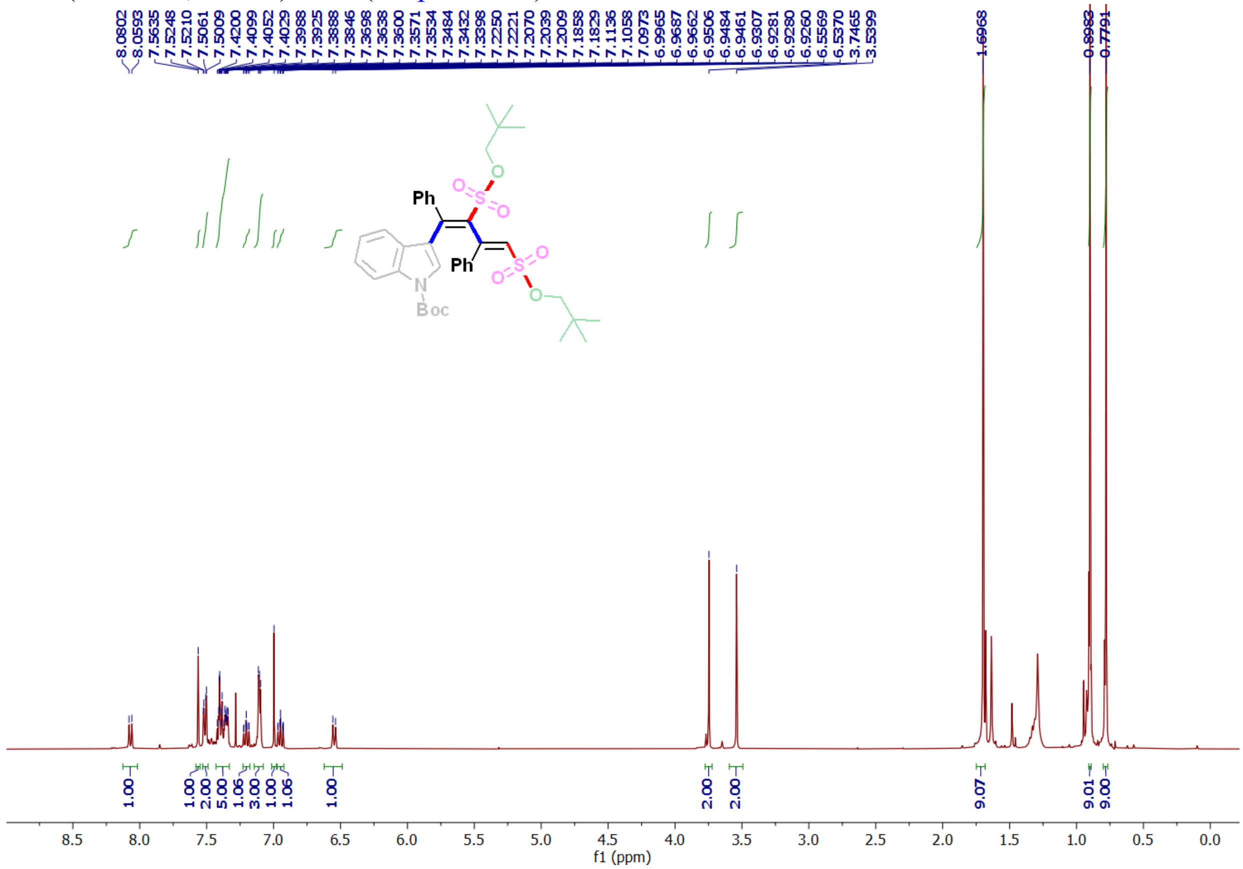

^{13}C NMR (101MHz, $CDCl_3$) of **4r**

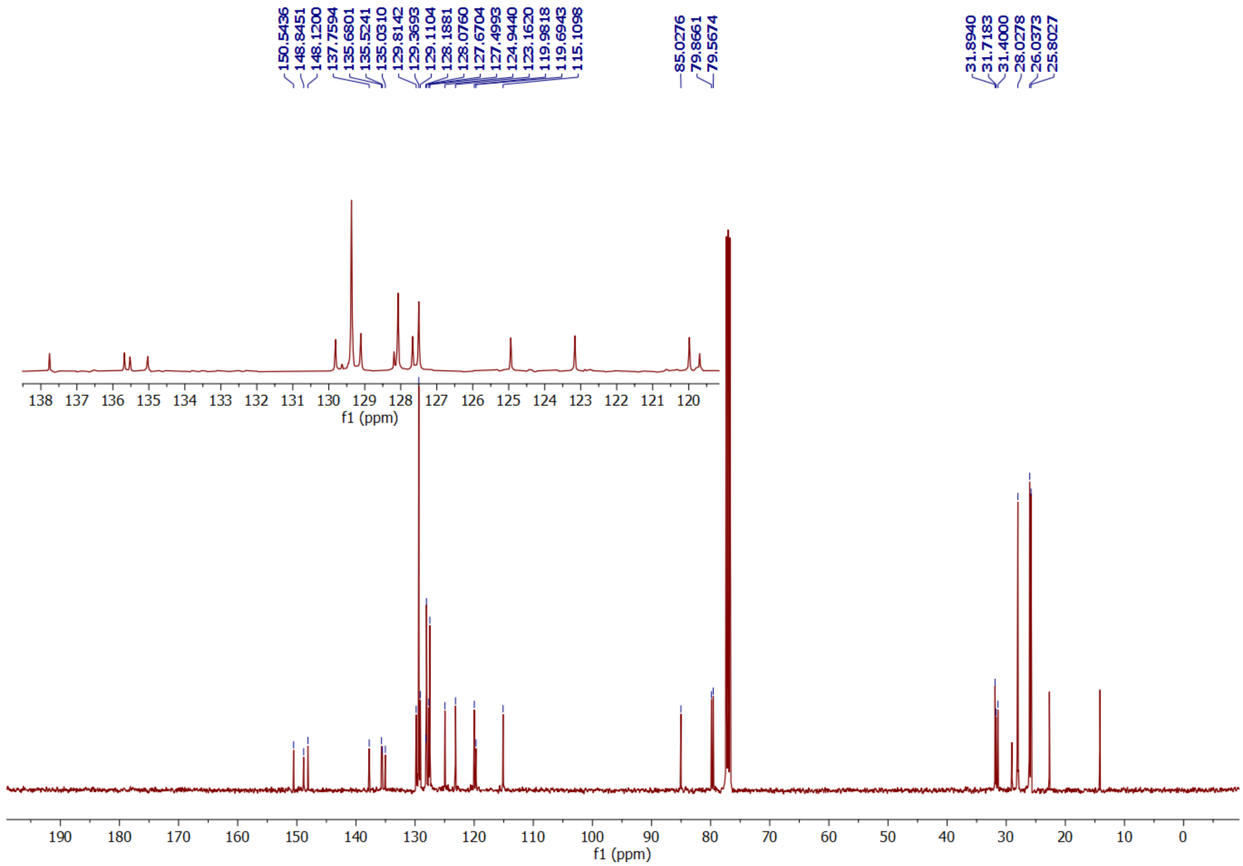

^{1}H NMR (400 MHz, $CDCl_3$) of **4s** (see procedure)

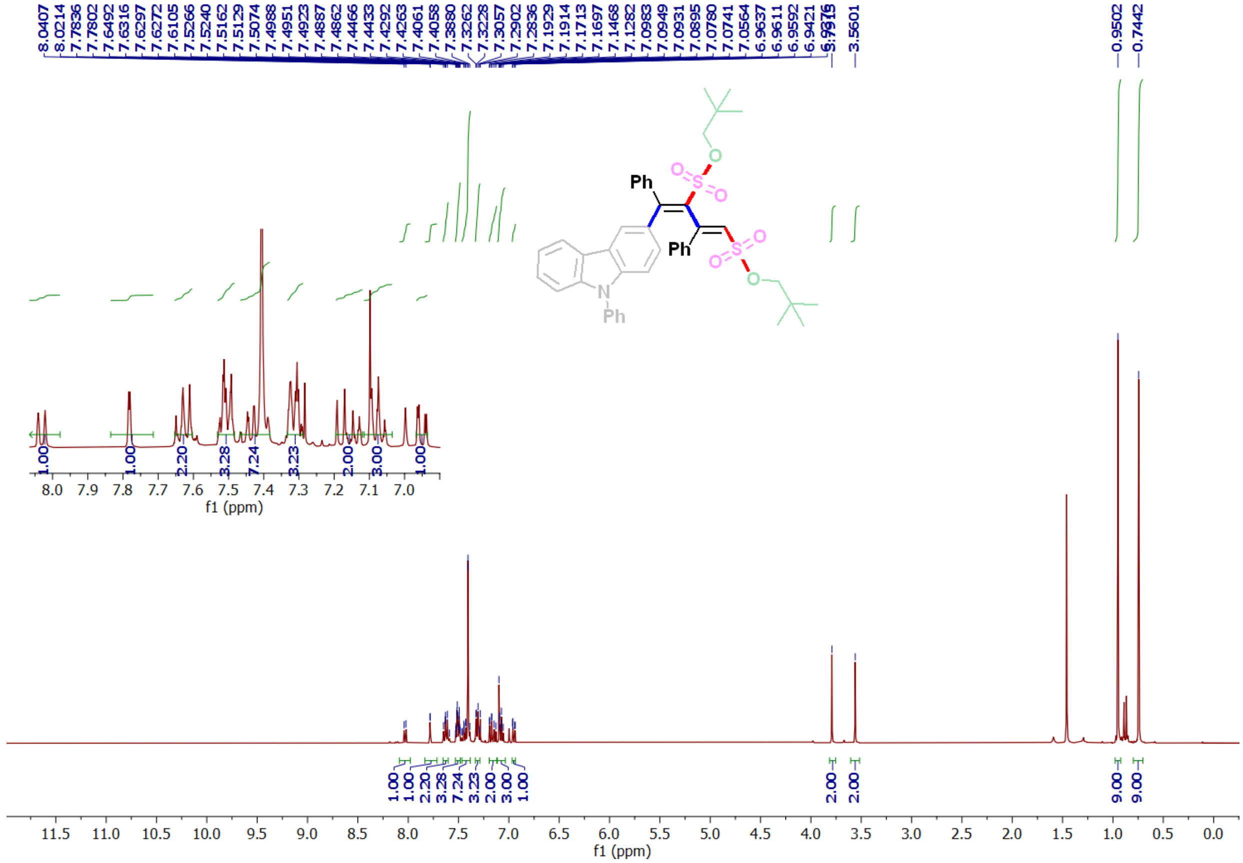

^{13}C NMR (101MHz, $CDCl_3$) of **4s**

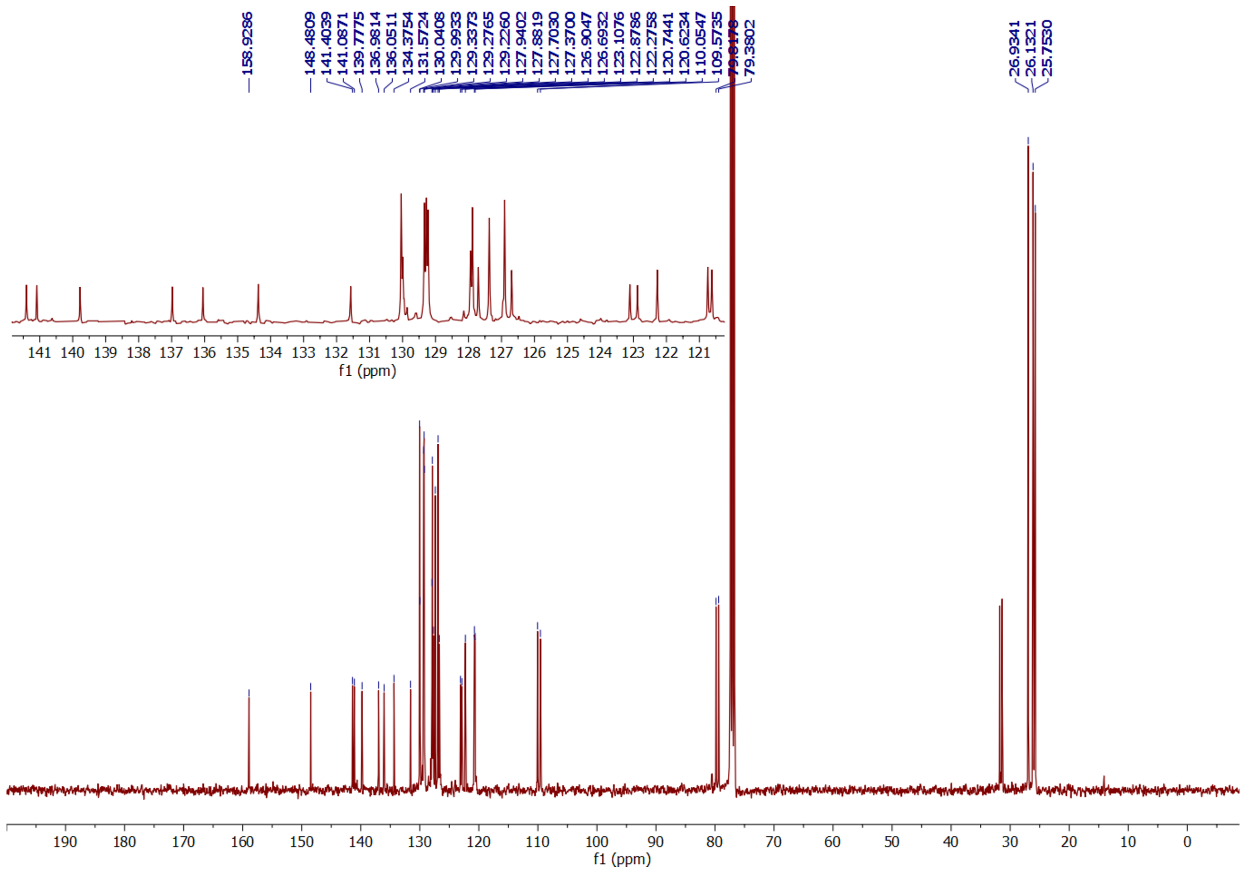

^{1}H NMR (300 MHz, $CDCl_3$) of **4t** (see procedure)

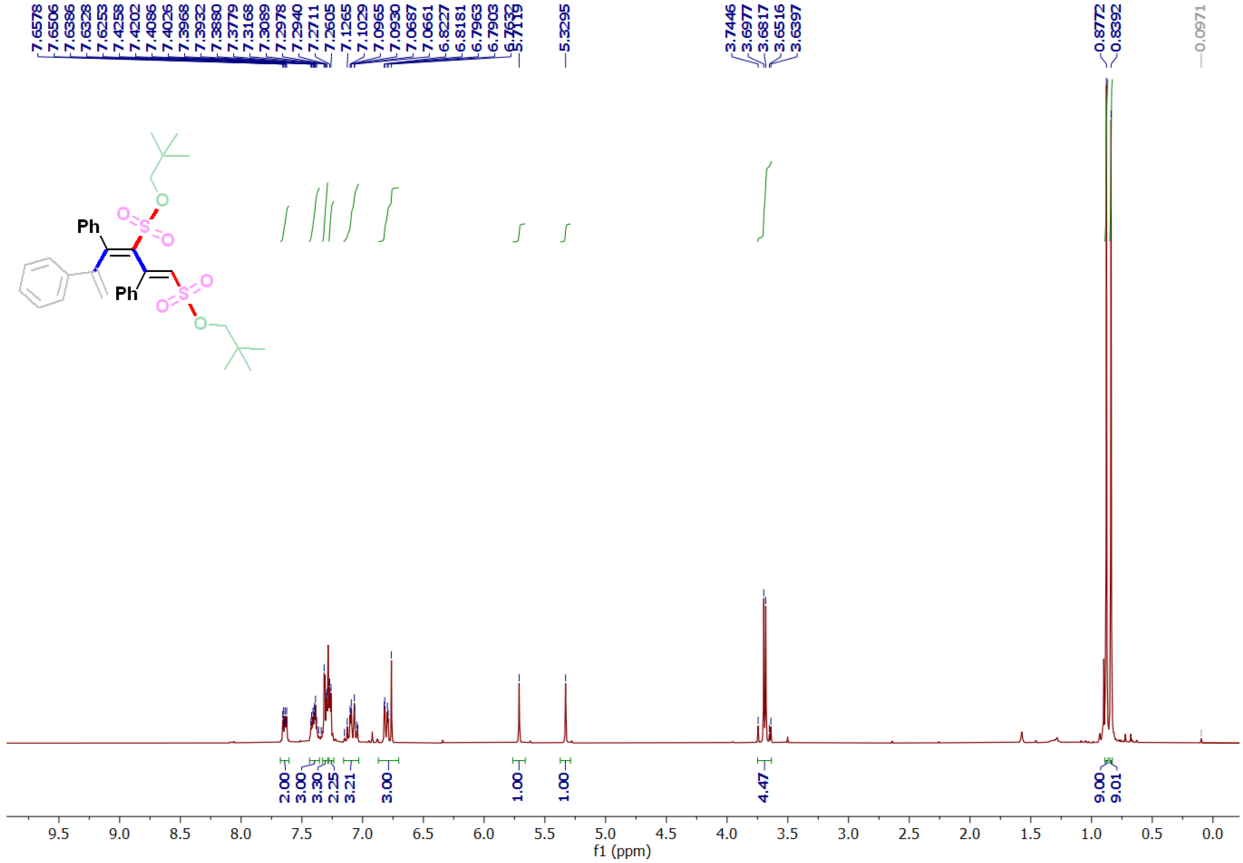

^{13}C NMR (75 MHz, $CDCl_3$) of **4t**

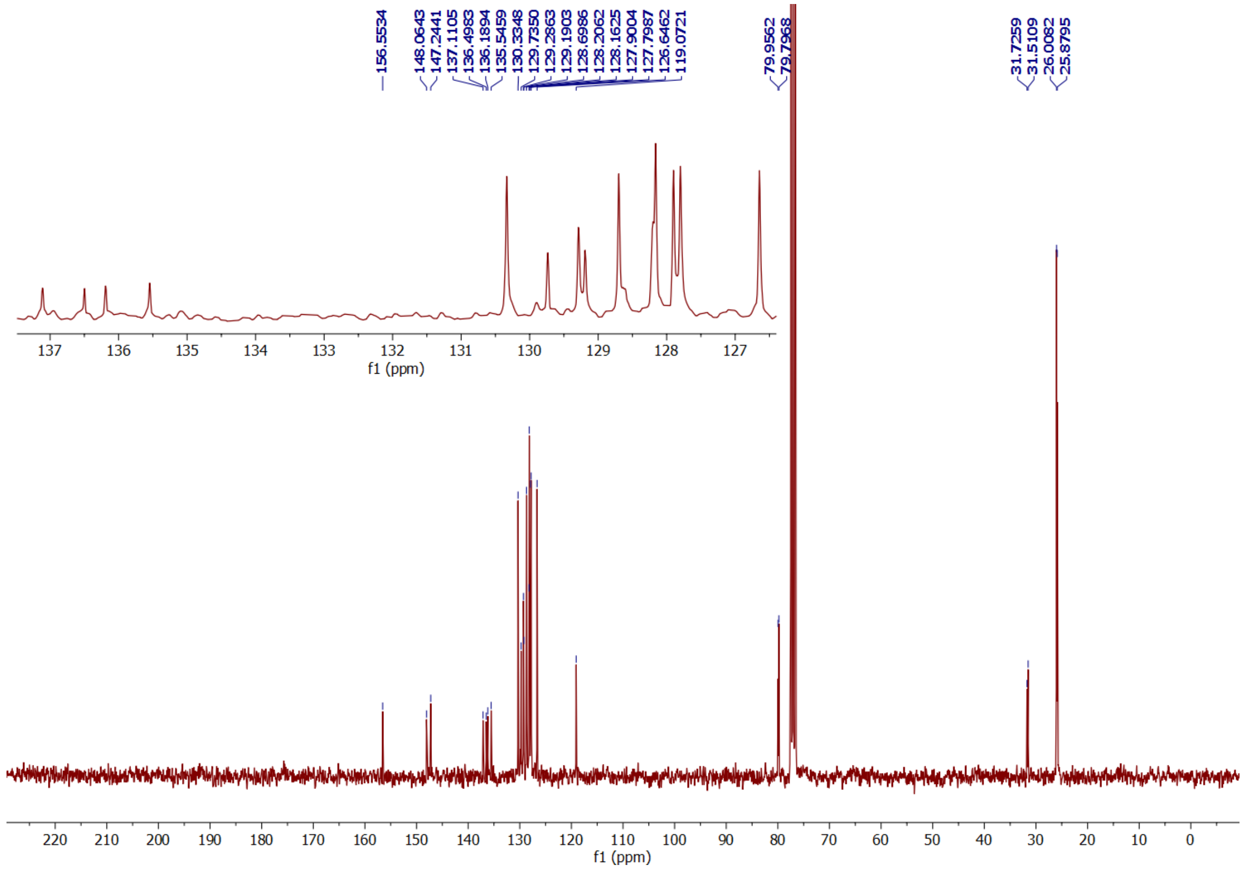

^{1}H NMR (300 MHz, $CDCl_3$) of **4u** (see procedure)

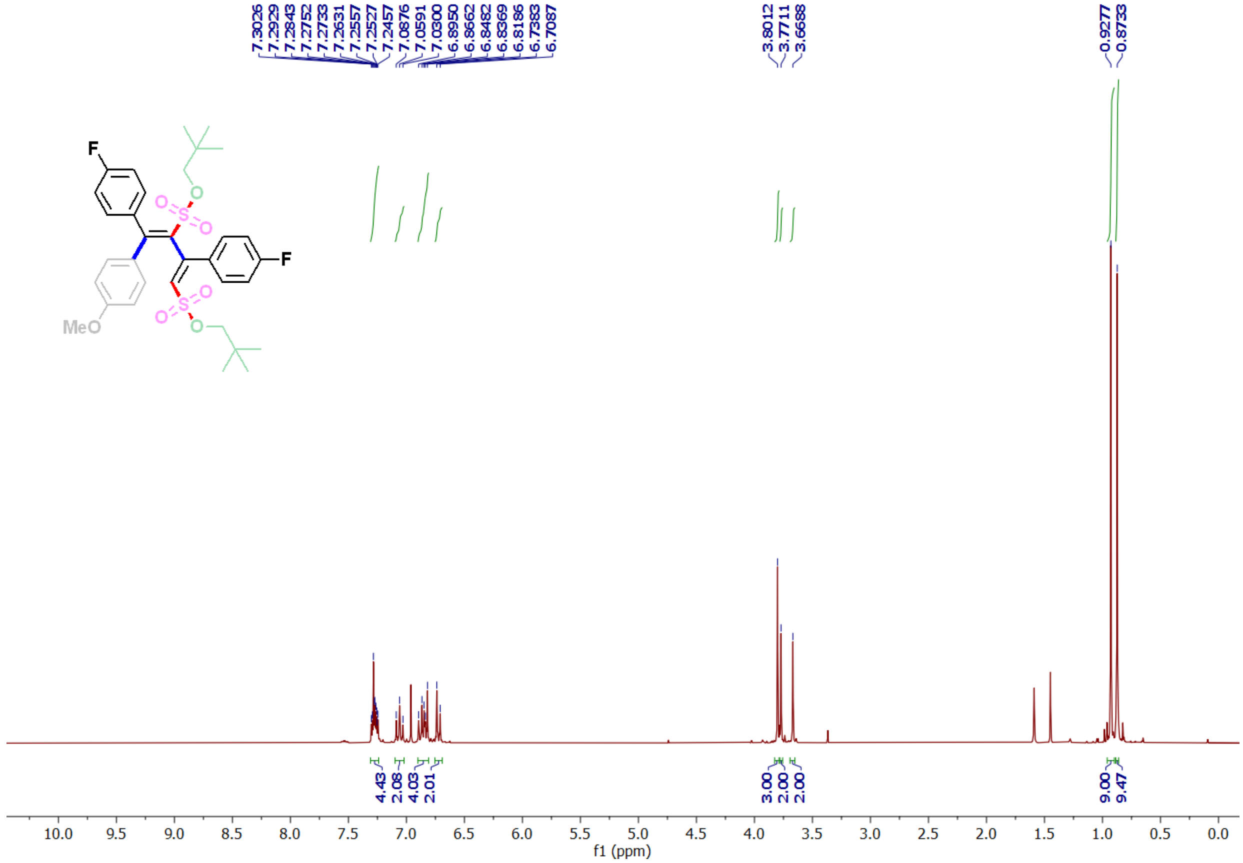

^{13}C NMR (75 MHz, $CDCl_3$) of **4u**

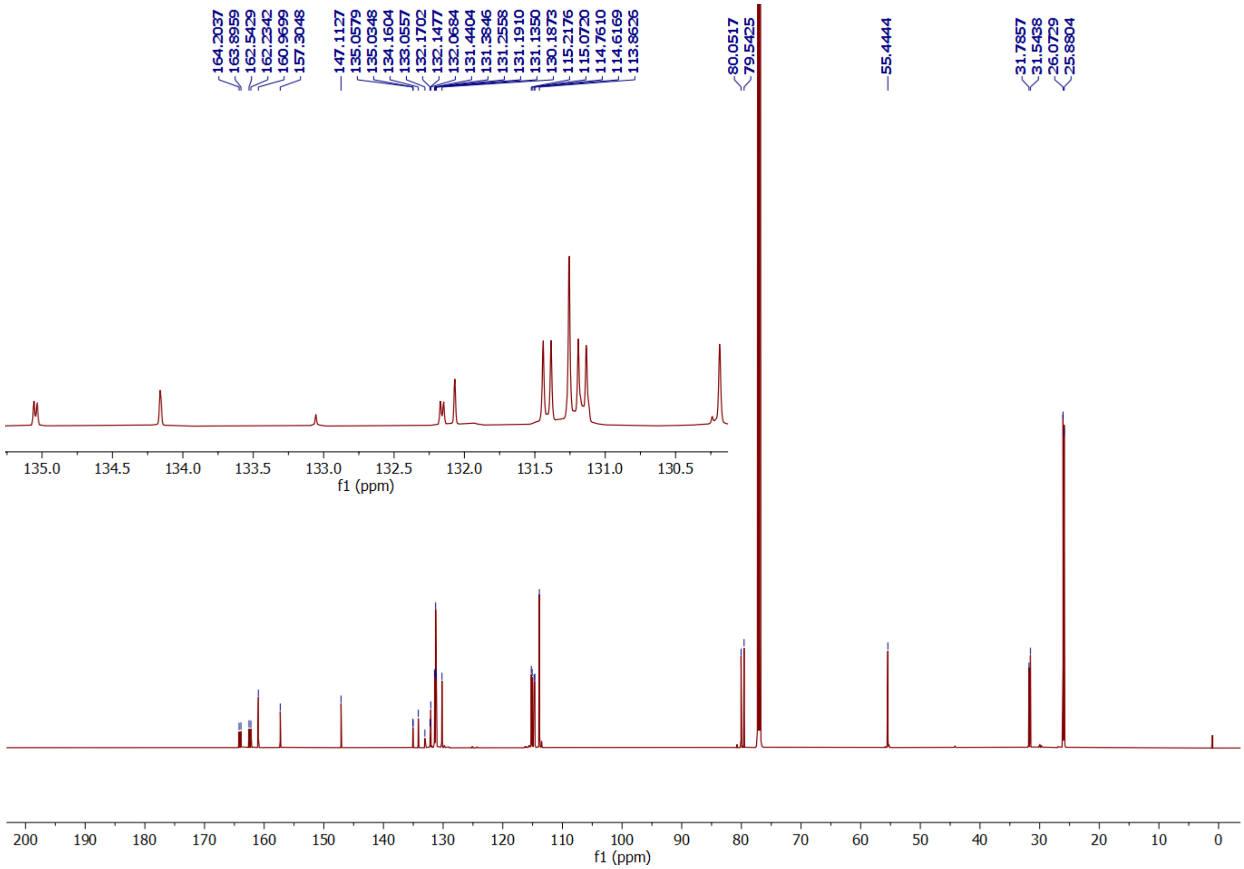

^{19}F NMR (282 MHz, $CDCl_3$) of **4u**

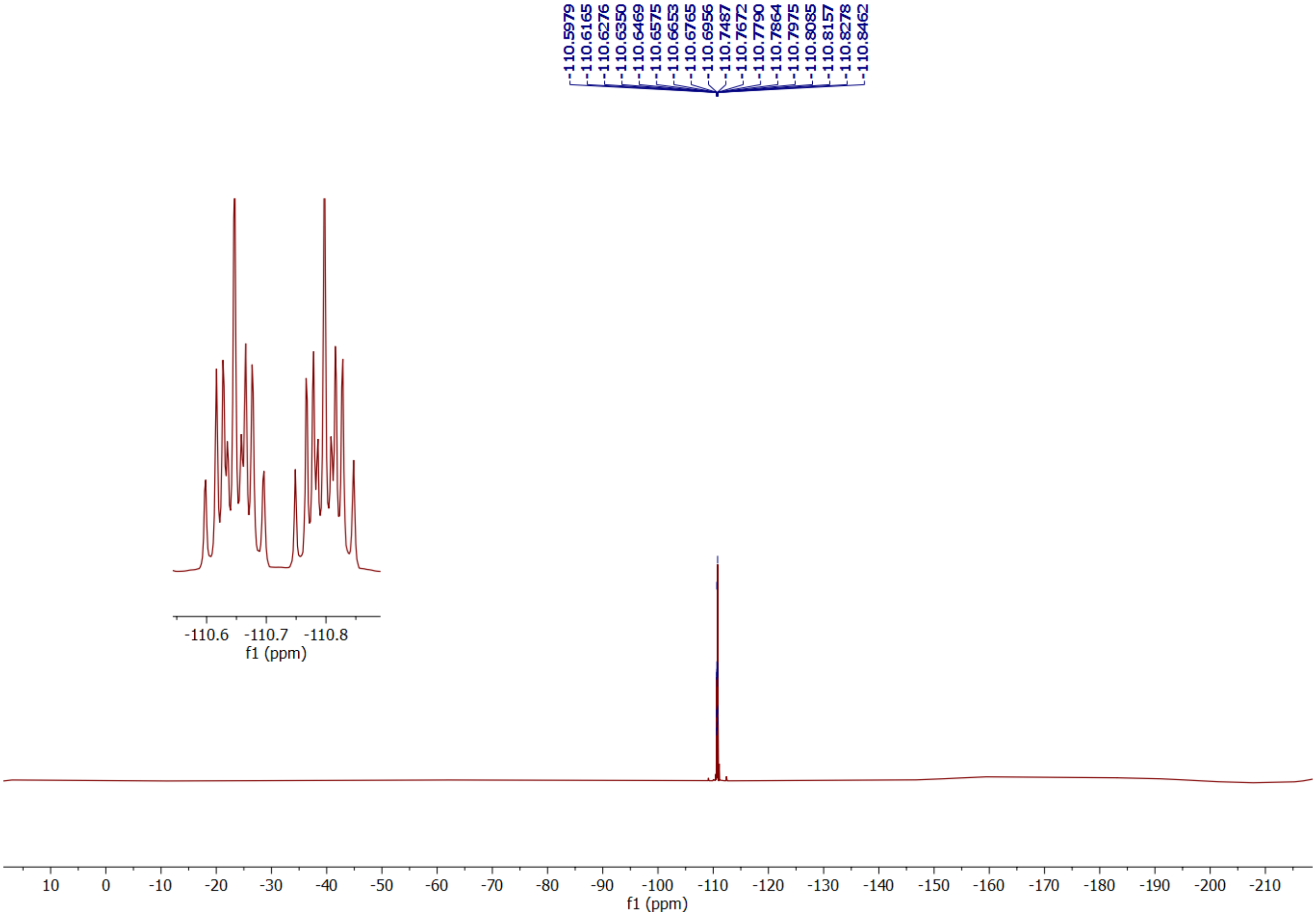

^{1}H NMR (300 MHz, $CDCl_3$) of **4v** (see procedure)

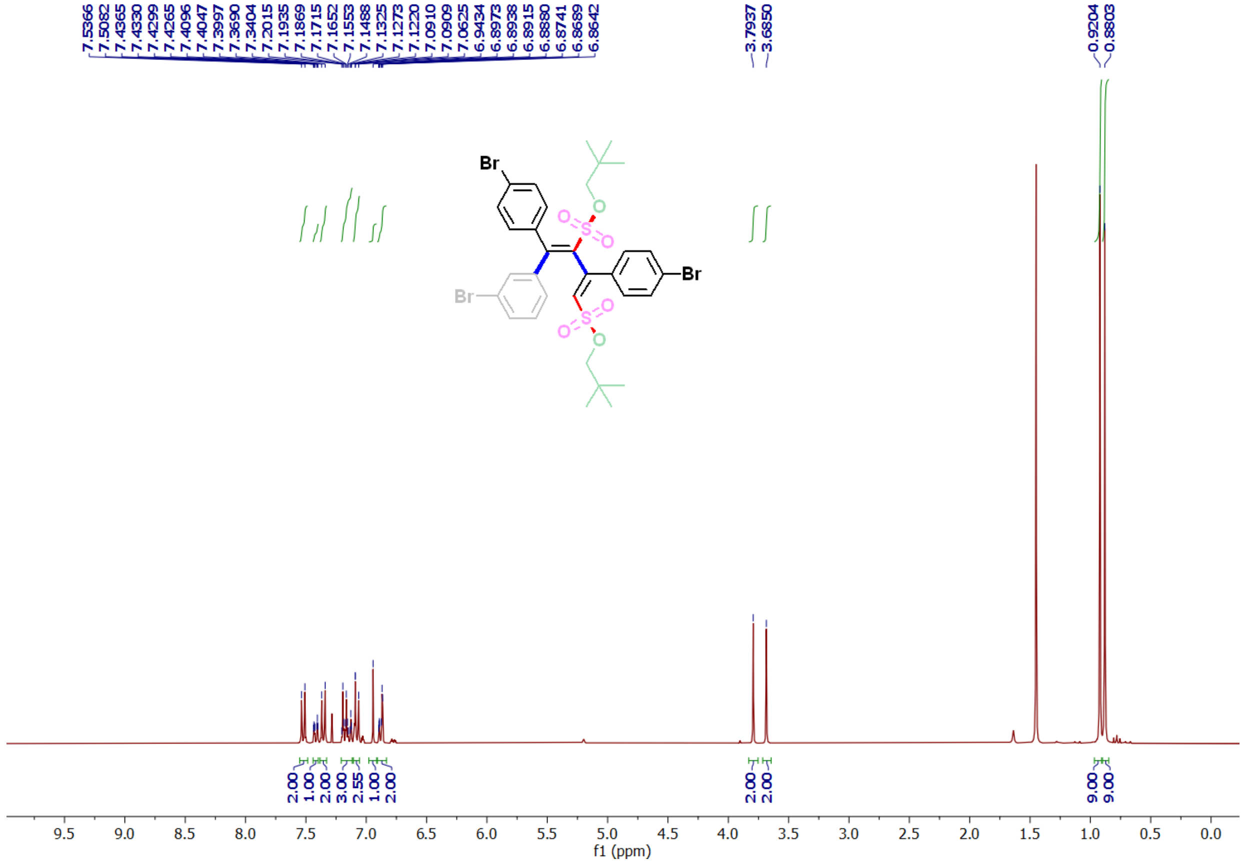

^{13}C NMR (75 MHz, $CDCl_3$) of **4v**

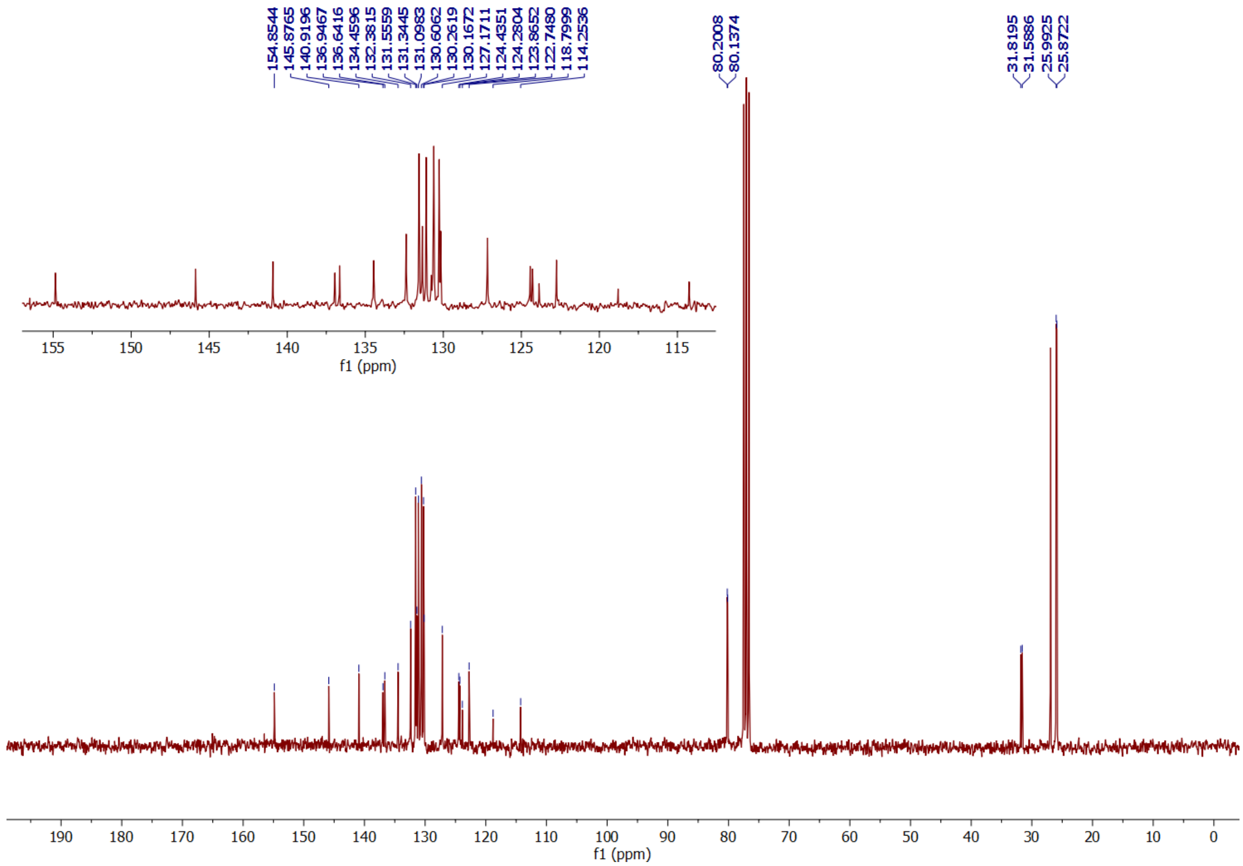

^{1}H NMR (400 MHz, $CDCl_3$) of **5a** (see procedure)

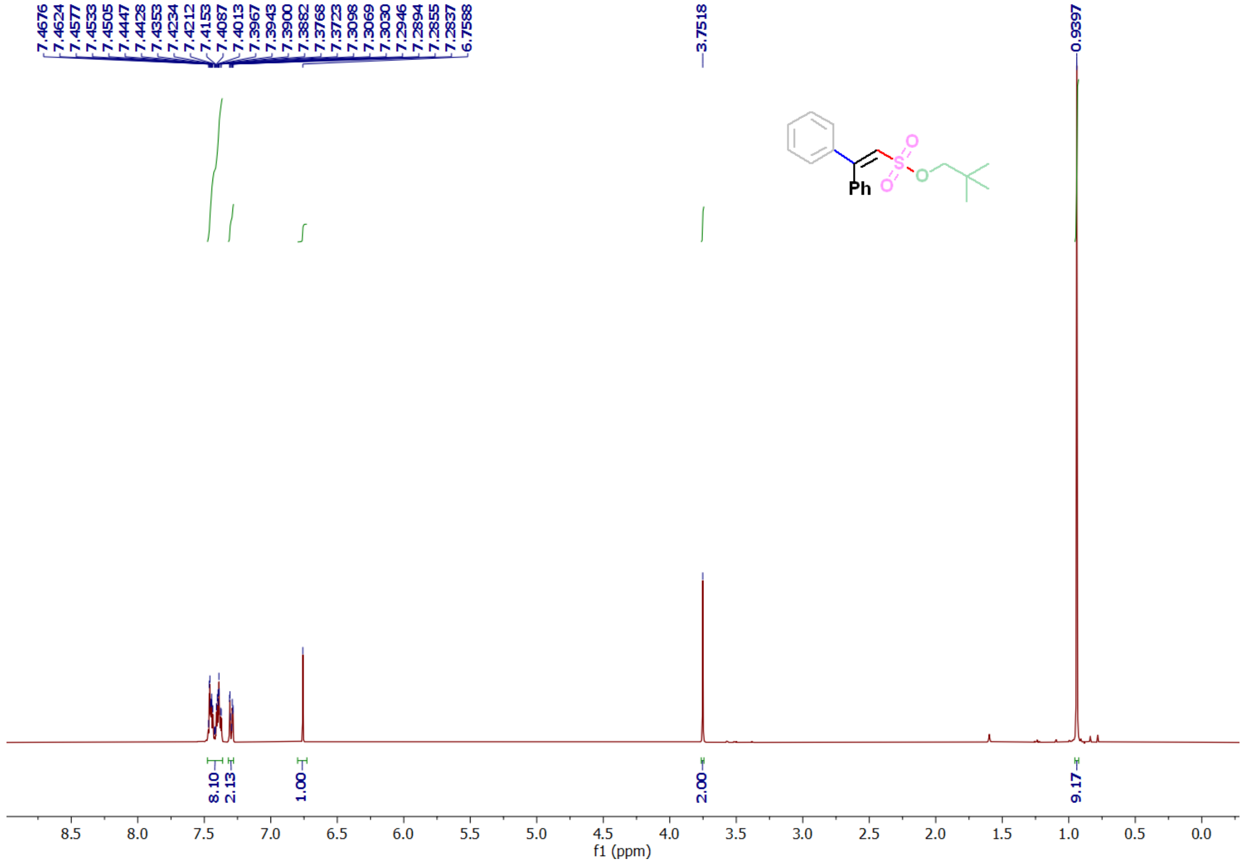

^{13}C NMR (101MHz, $CDCl_3$) of **5a**

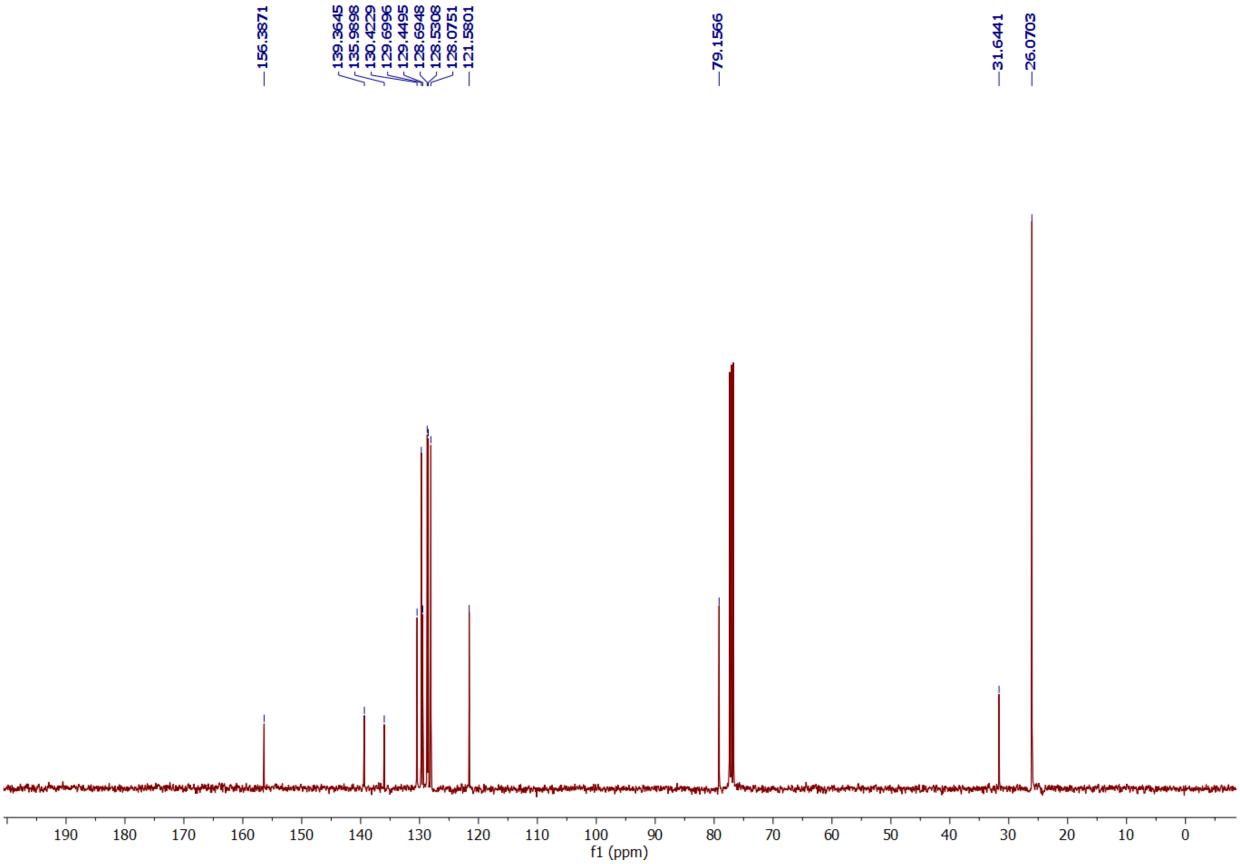

^{1}H NMR (400 MHz, $CDCl_3$) of **5b** (see procedure)

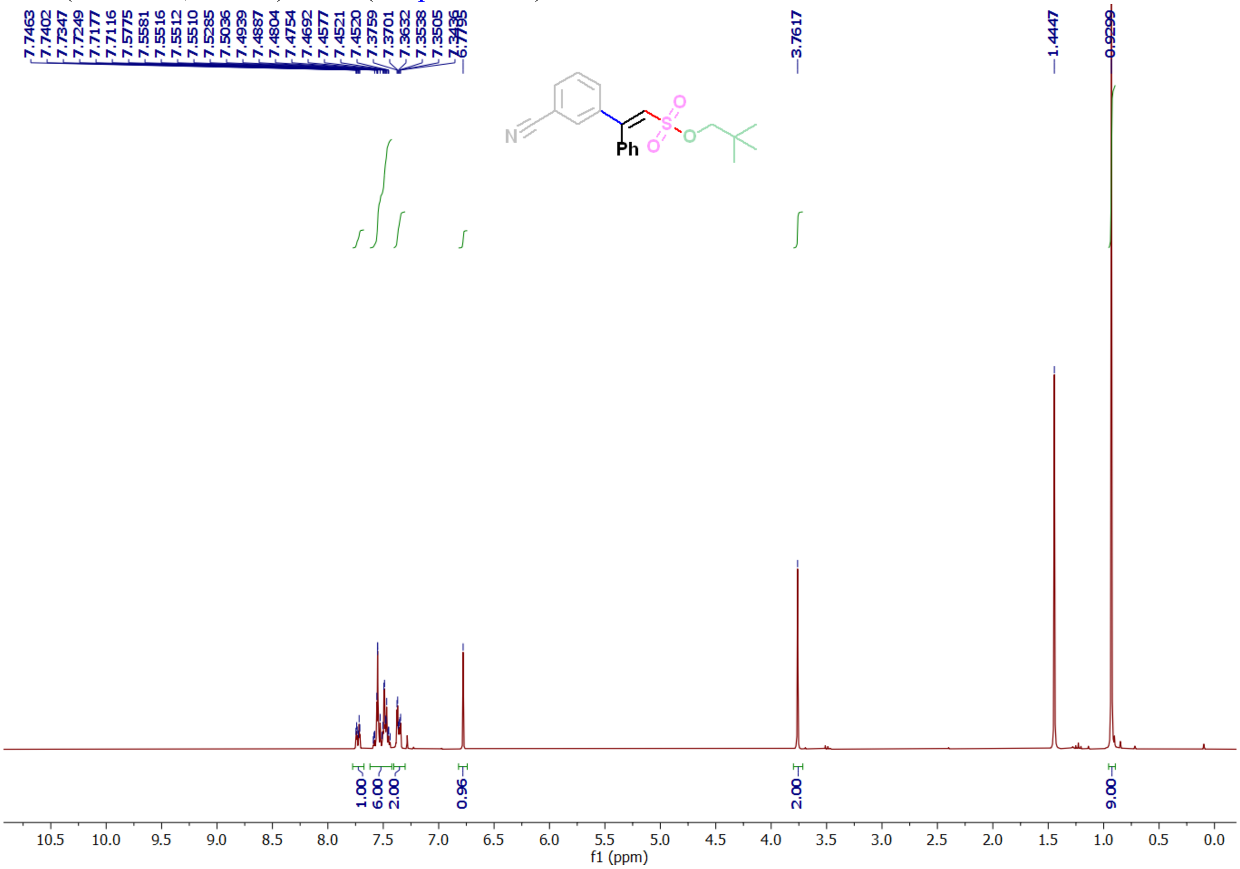

^{13}C NMR (101MHz, $CDCl_3$) of **5b**

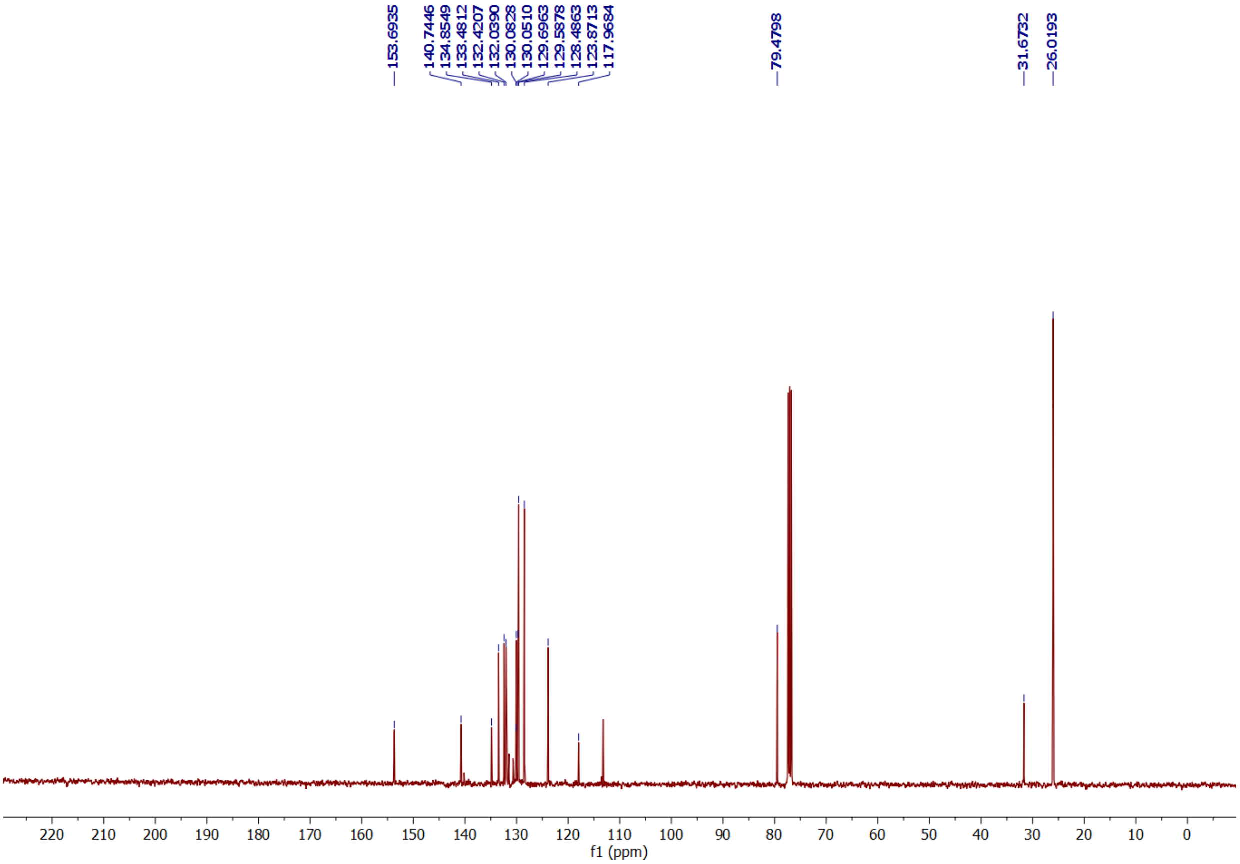

^{1}H NMR (400 MHz, $CDCl_3$) of **5c** (see procedure)

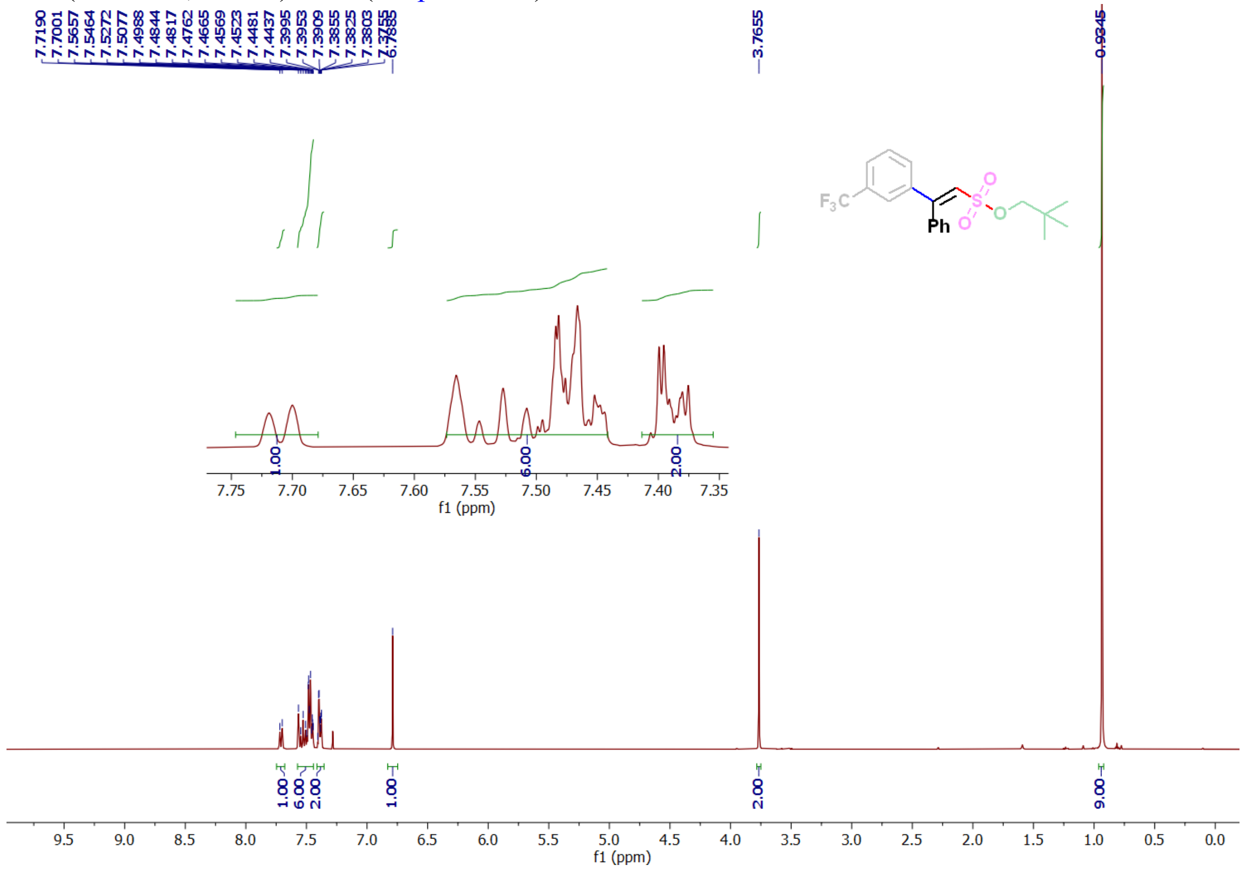

^{13}C NMR (101MHz, $CDCl_3$) of **5c**

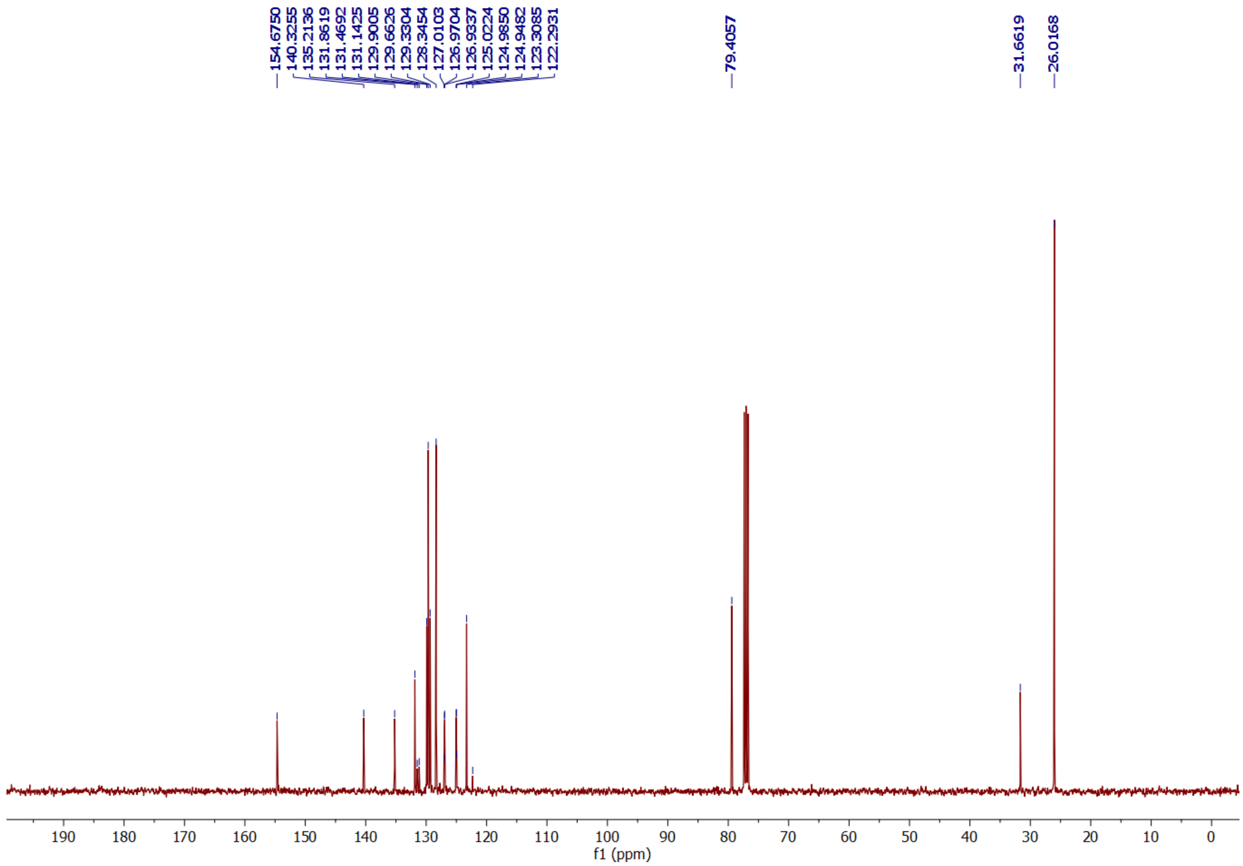

^{19}F NMR (282 MHz, $CDCl_3$) of **5c**

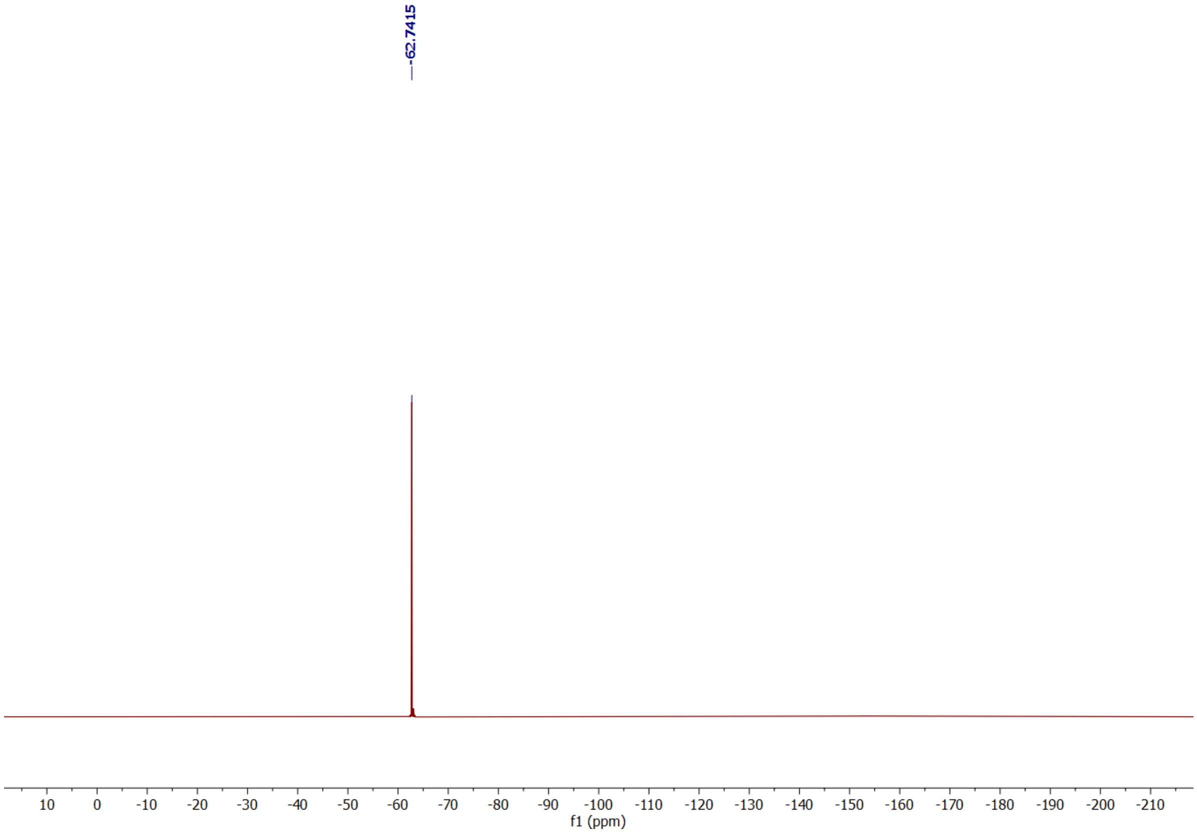

NOE Spectrum for **5c**

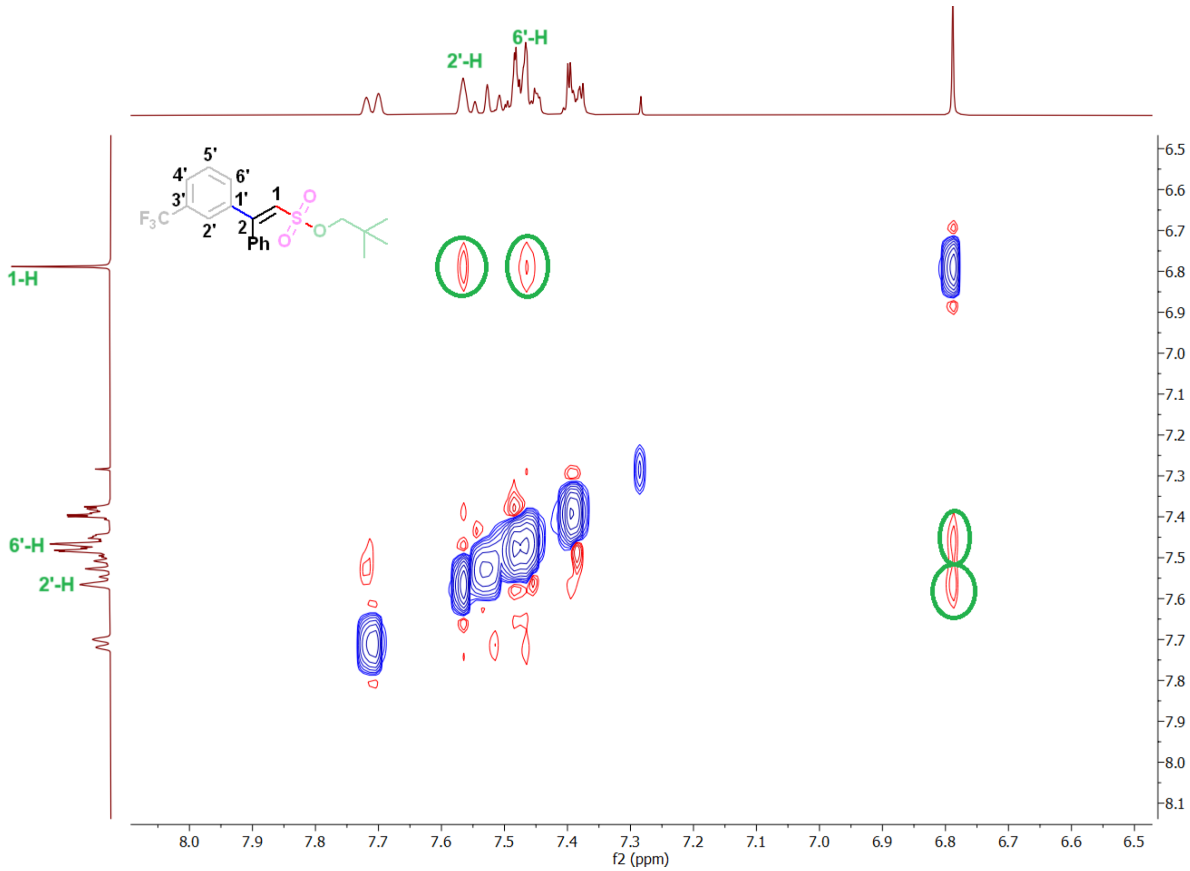

^{1}H NMR (300 MHz, $CDCl_3$) of **5c-D1** (see procedure)

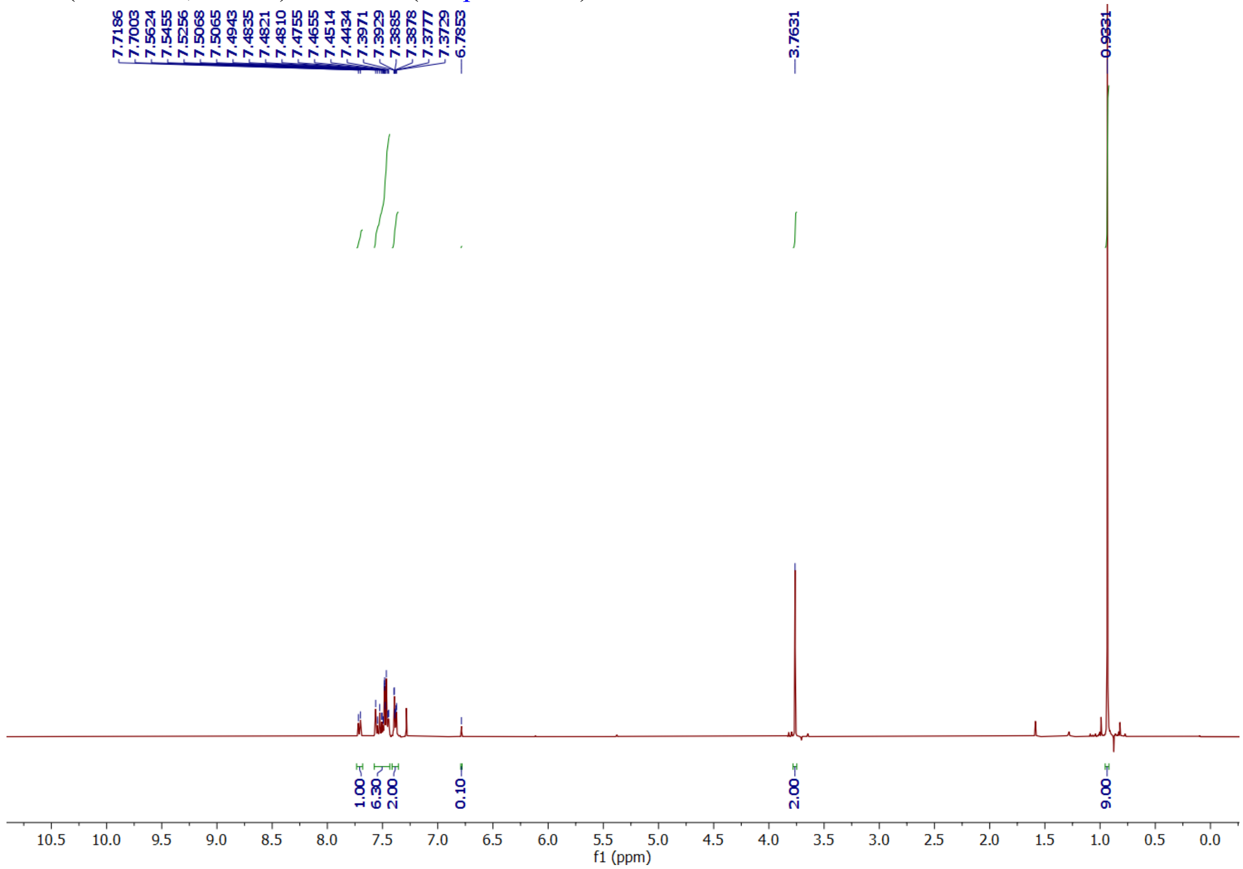

^{1}H NMR (400 MHz, $CDCl_3$) of **5d** (see procedure)

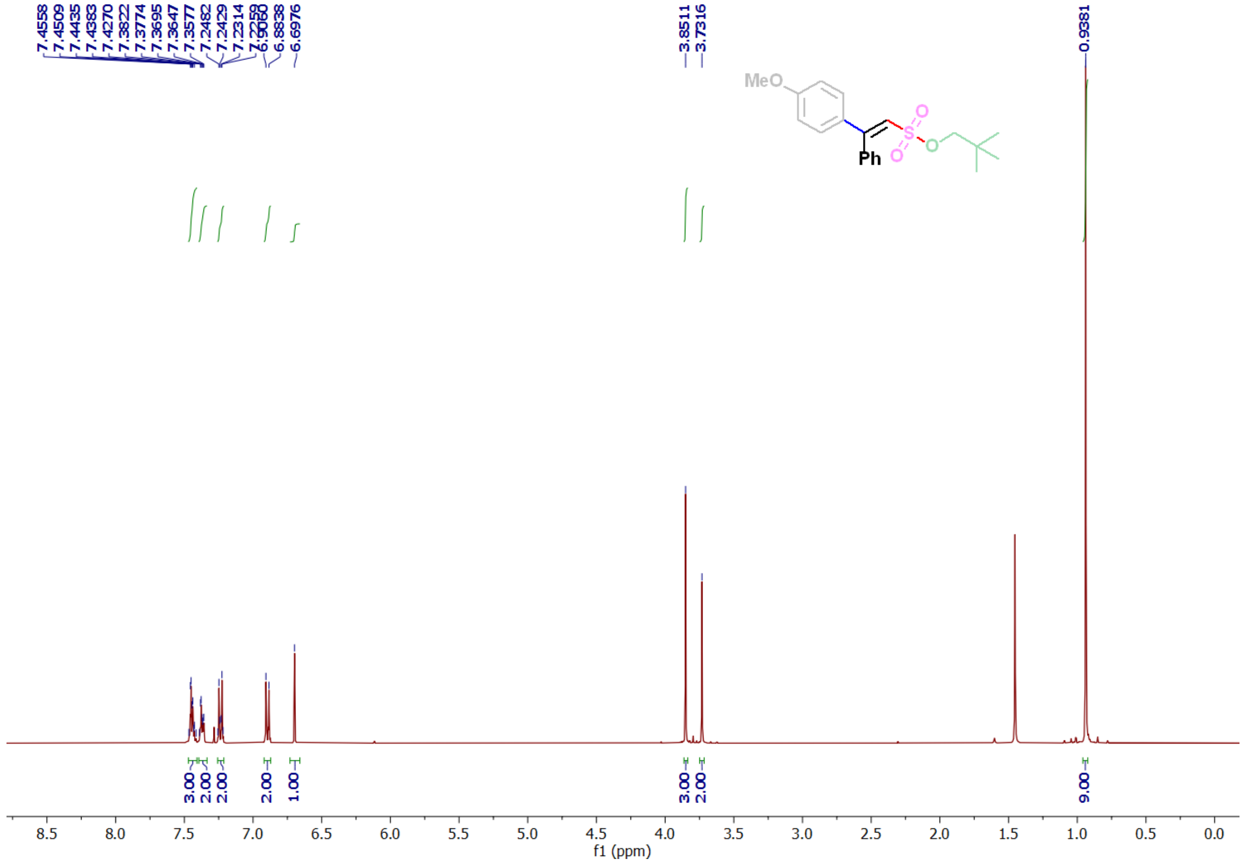

^{13}C NMR (101MHz, $CDCl_3$) of **5d**

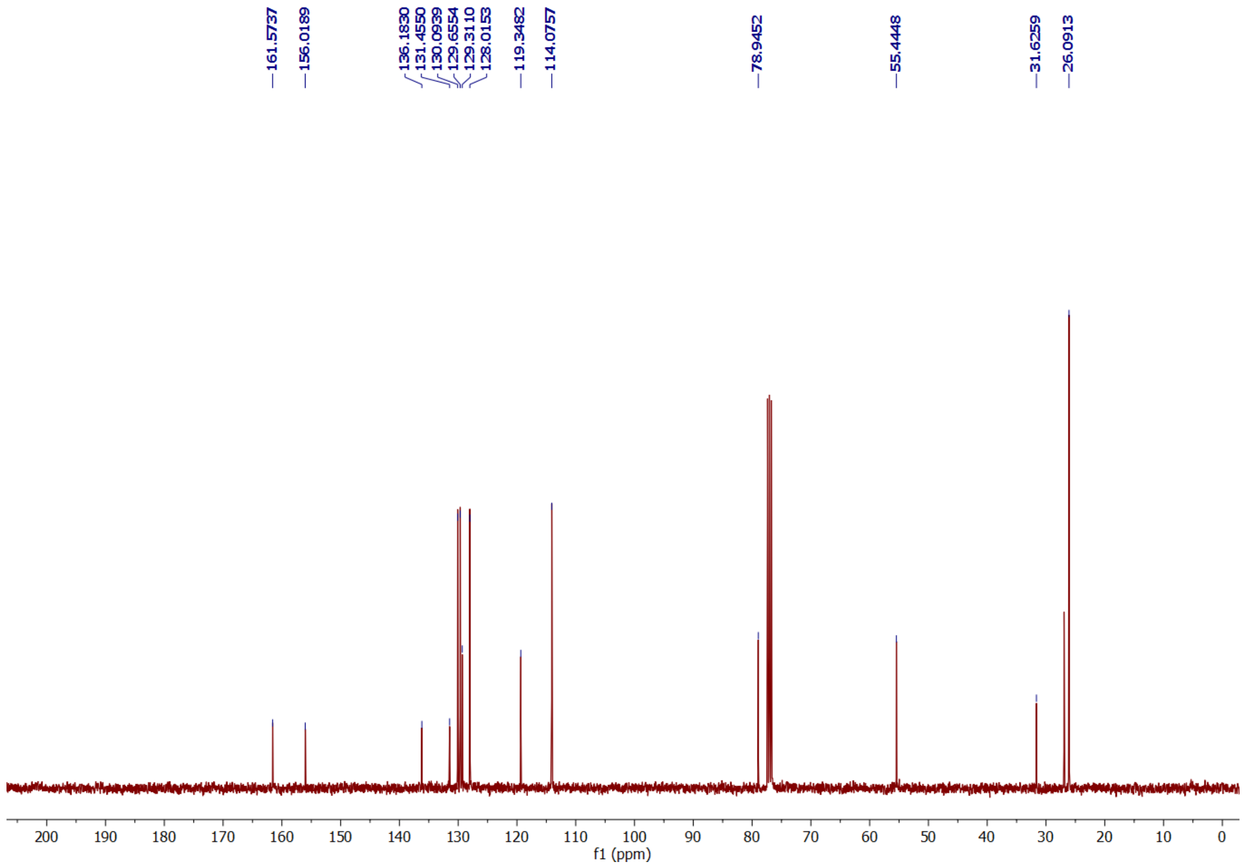

NOE Spectrum for **5d**

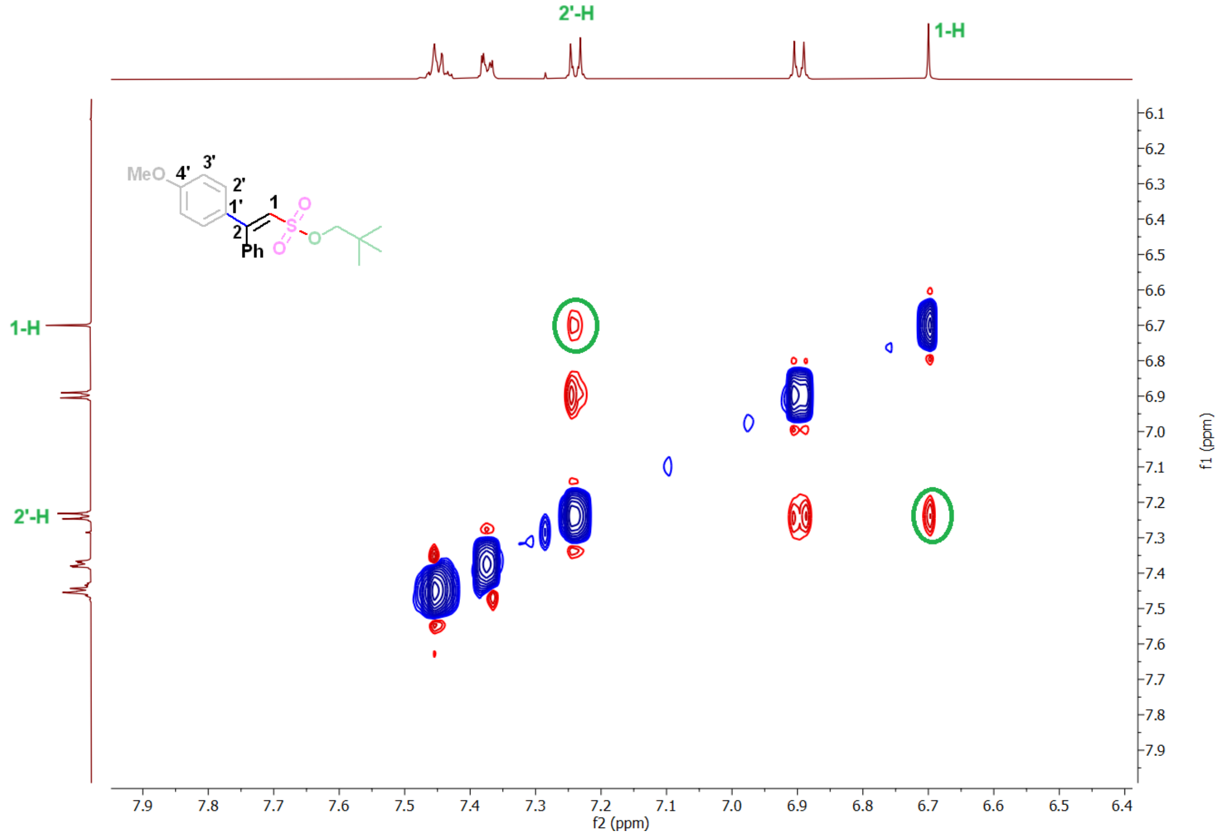

^{1}H NMR (400 MHz, $CDCl_3$) of **5e** (see procedure)

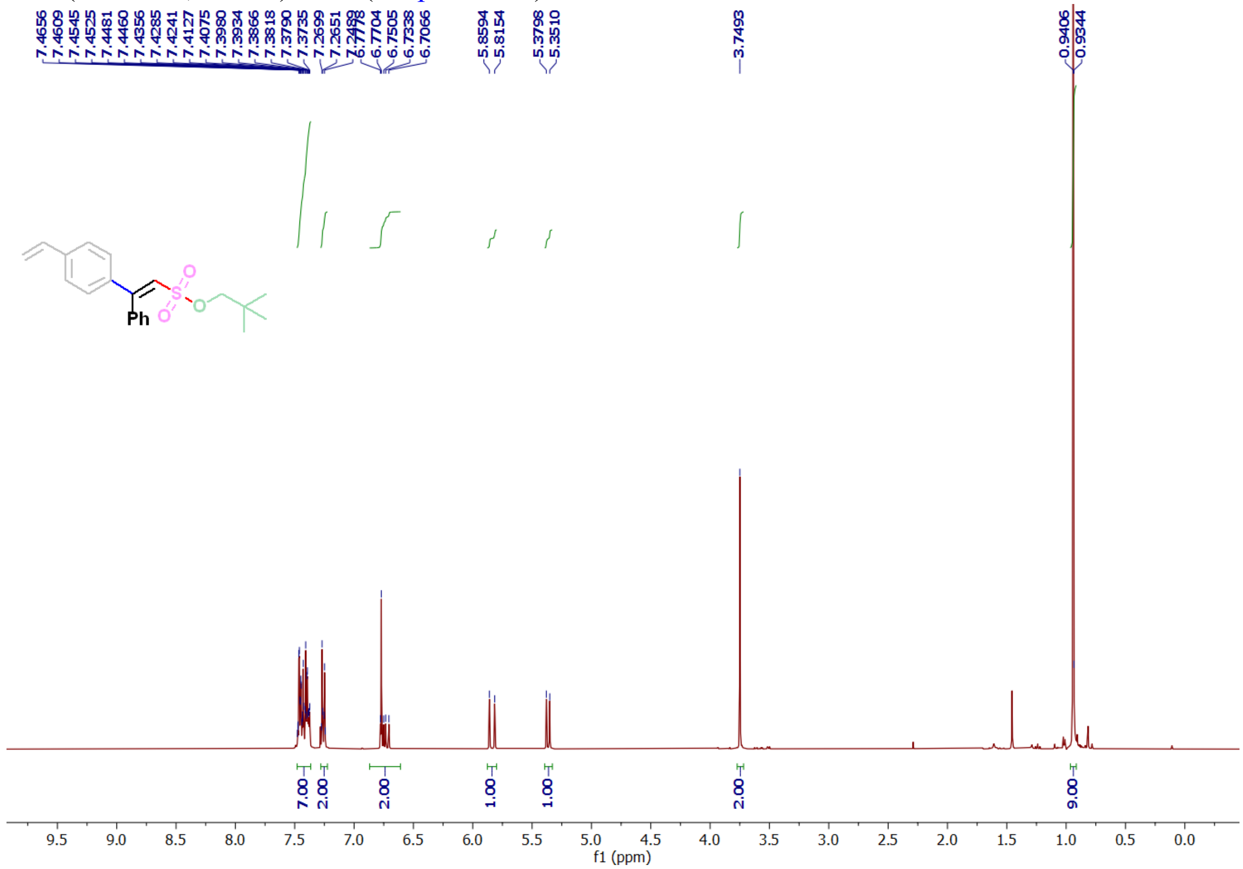

^{13}C NMR (101MHz, $CDCl_3$) of **5e**

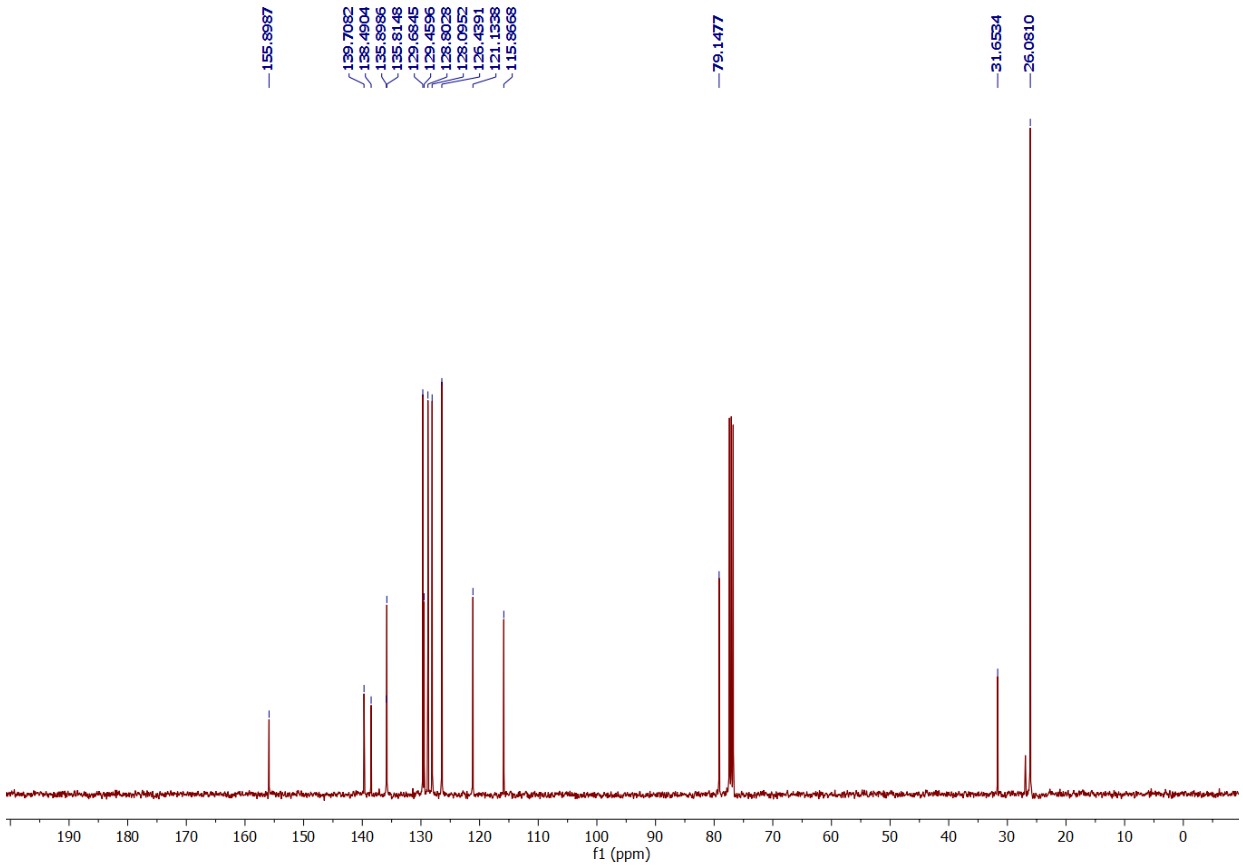

NOE Spectrum for **5e**

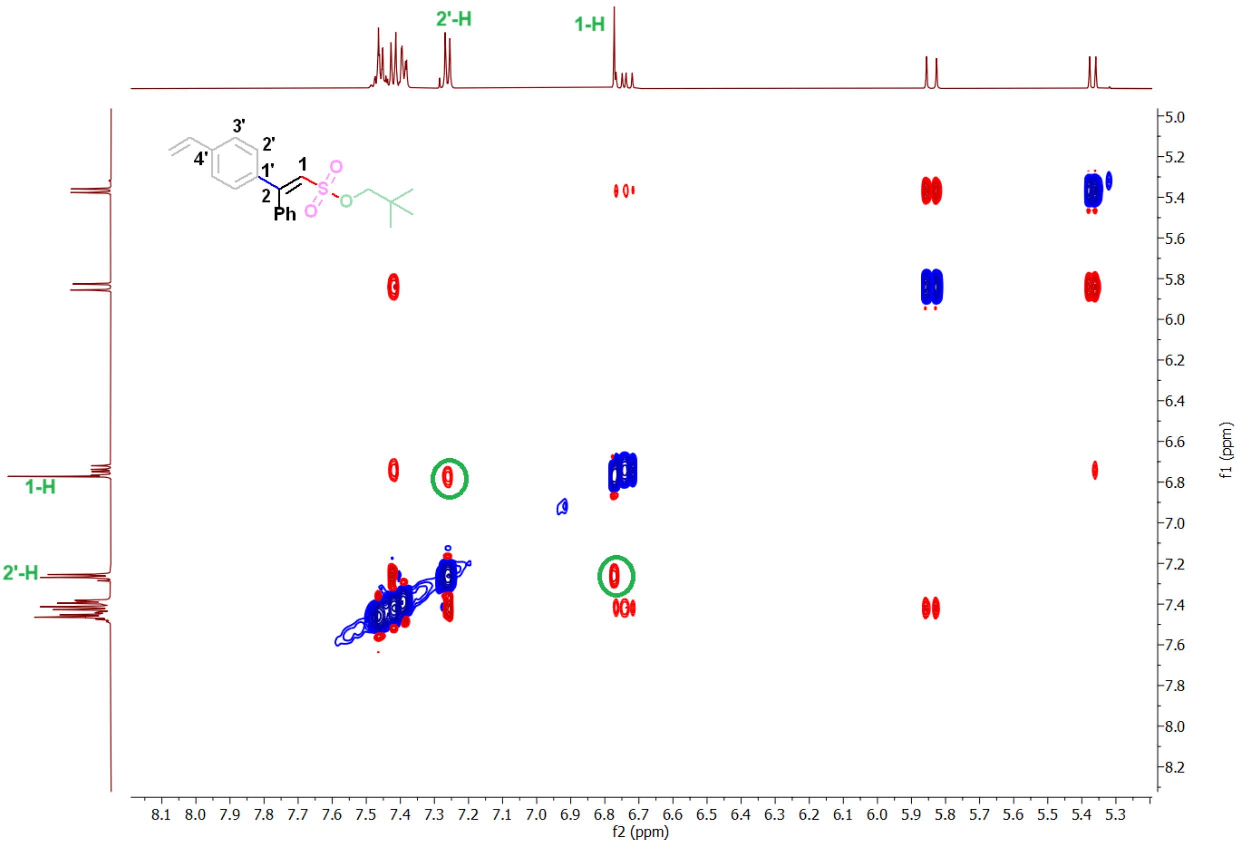

^{1}H NMR (400 MHz, $CDCl_3$) of **5f** (see procedure)

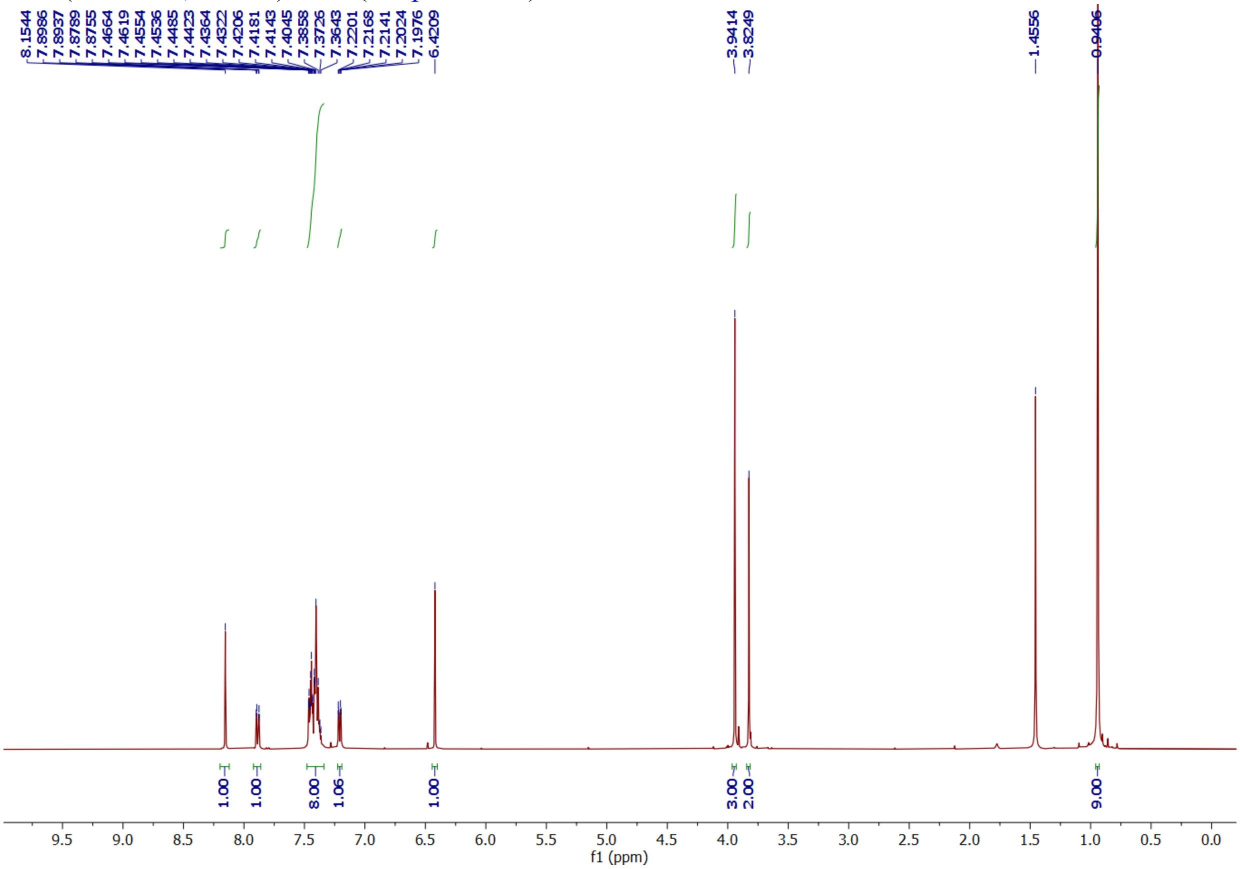

^{13}C NMR (101MHz, $CDCl_3$) of **5f**

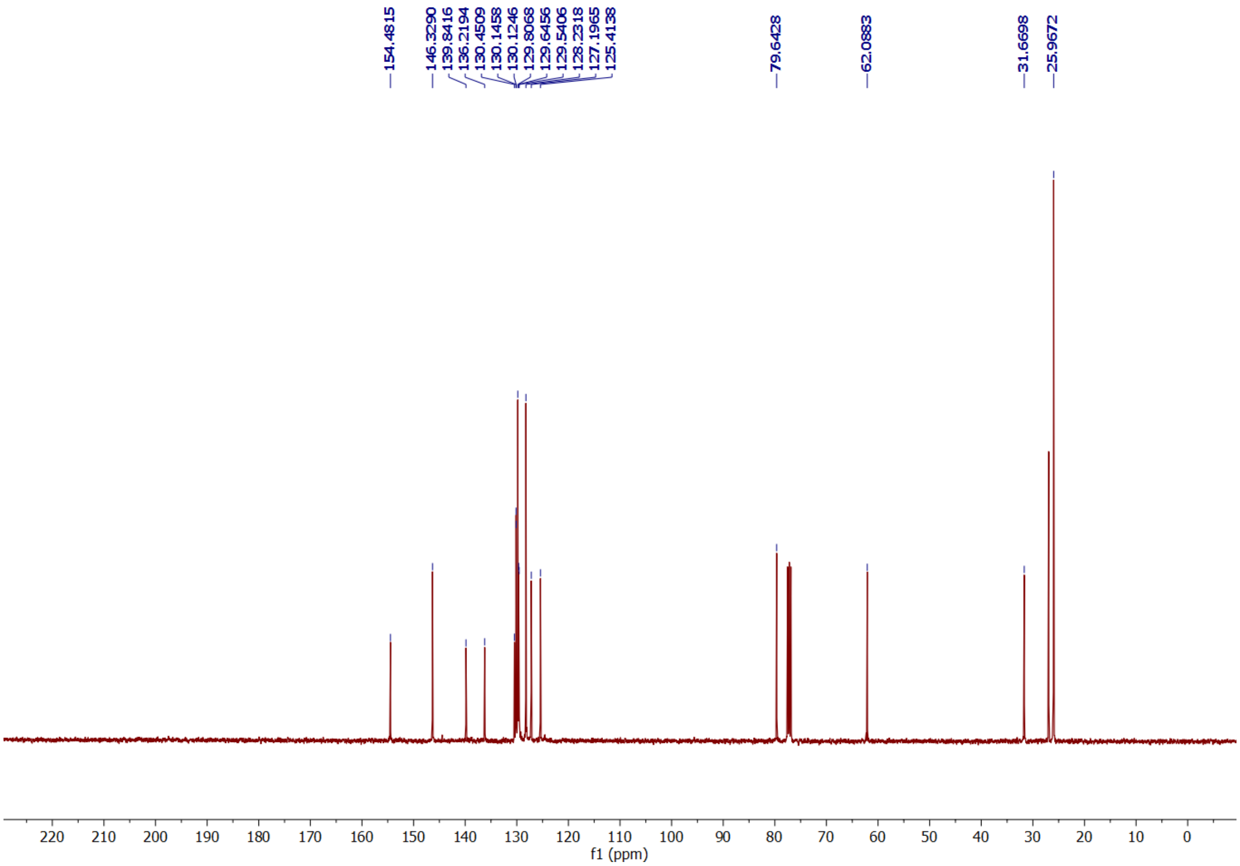

NOE Spectrum for **5f**

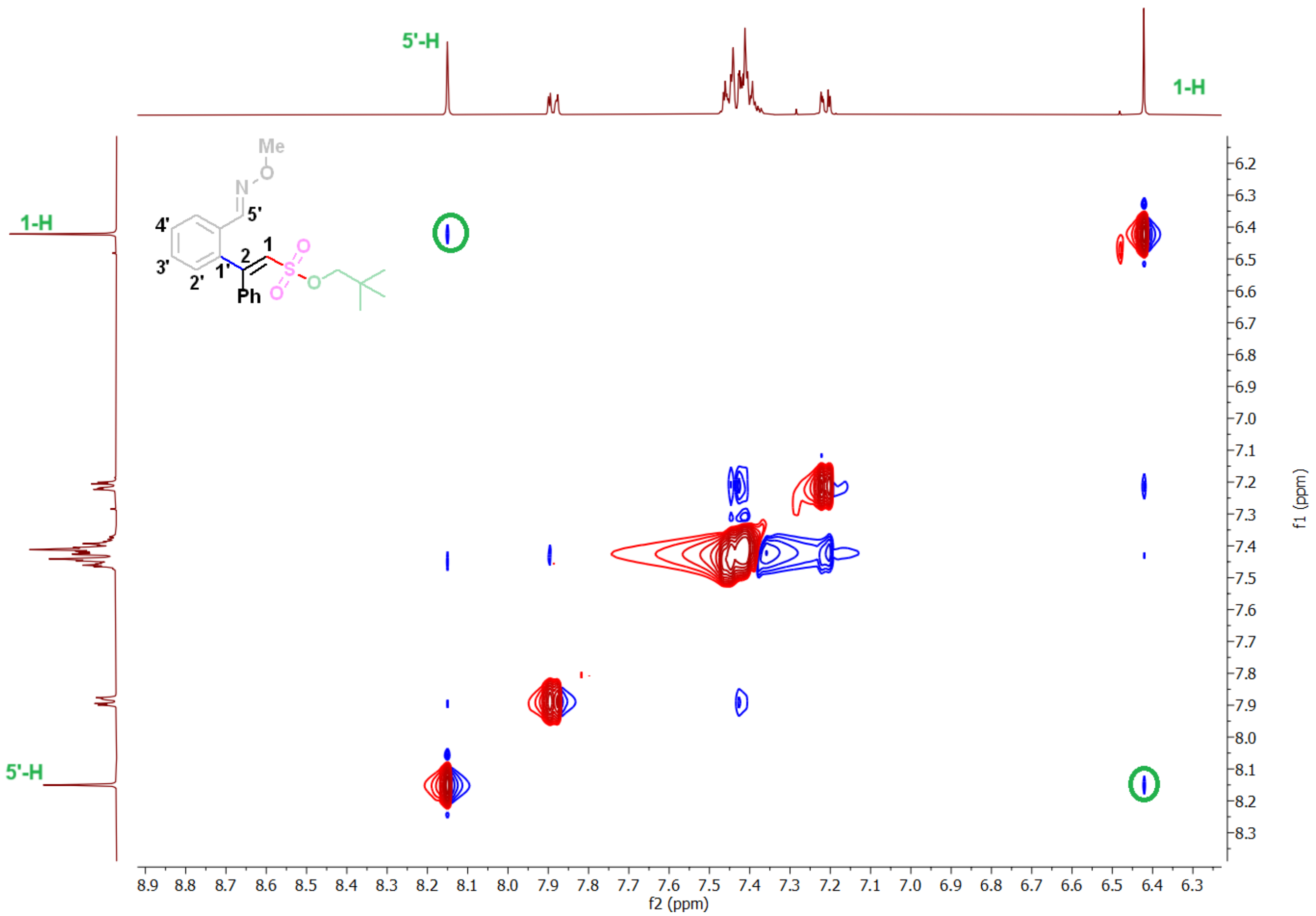

^{1}H NMR (400 MHz, $CDCl_3$) of **5g** (see procedure)

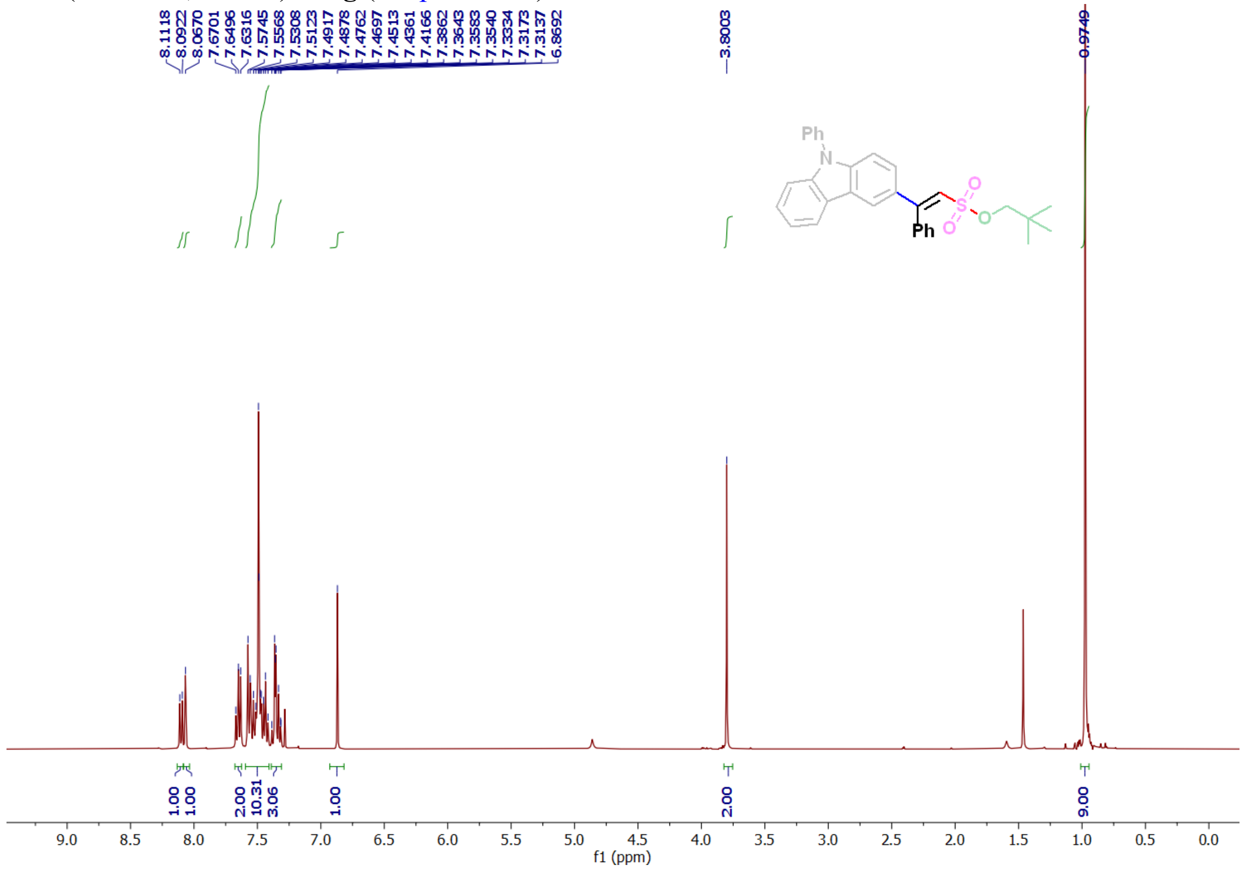

^{13}C NMR (101MHz, $CDCl_3$) of **5g**

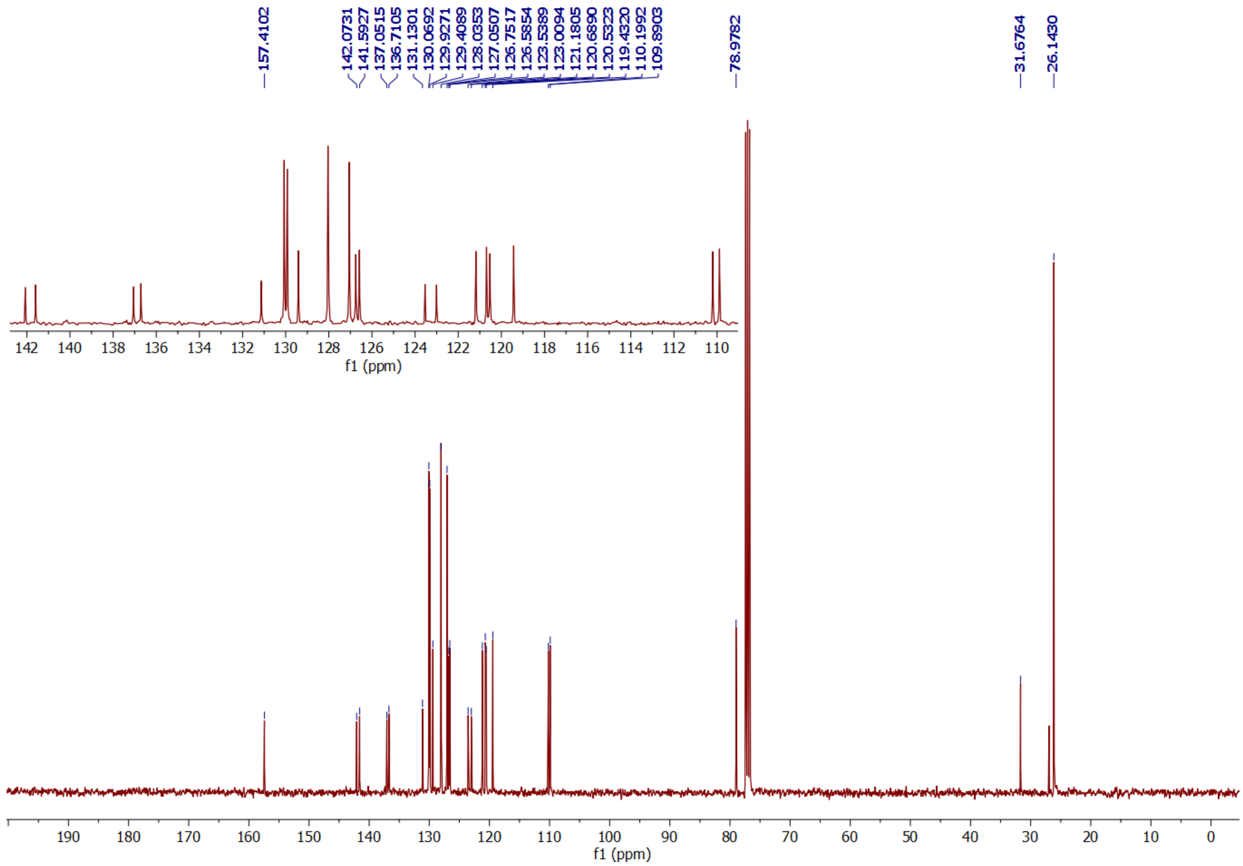

NOE Spectrum for **5g**

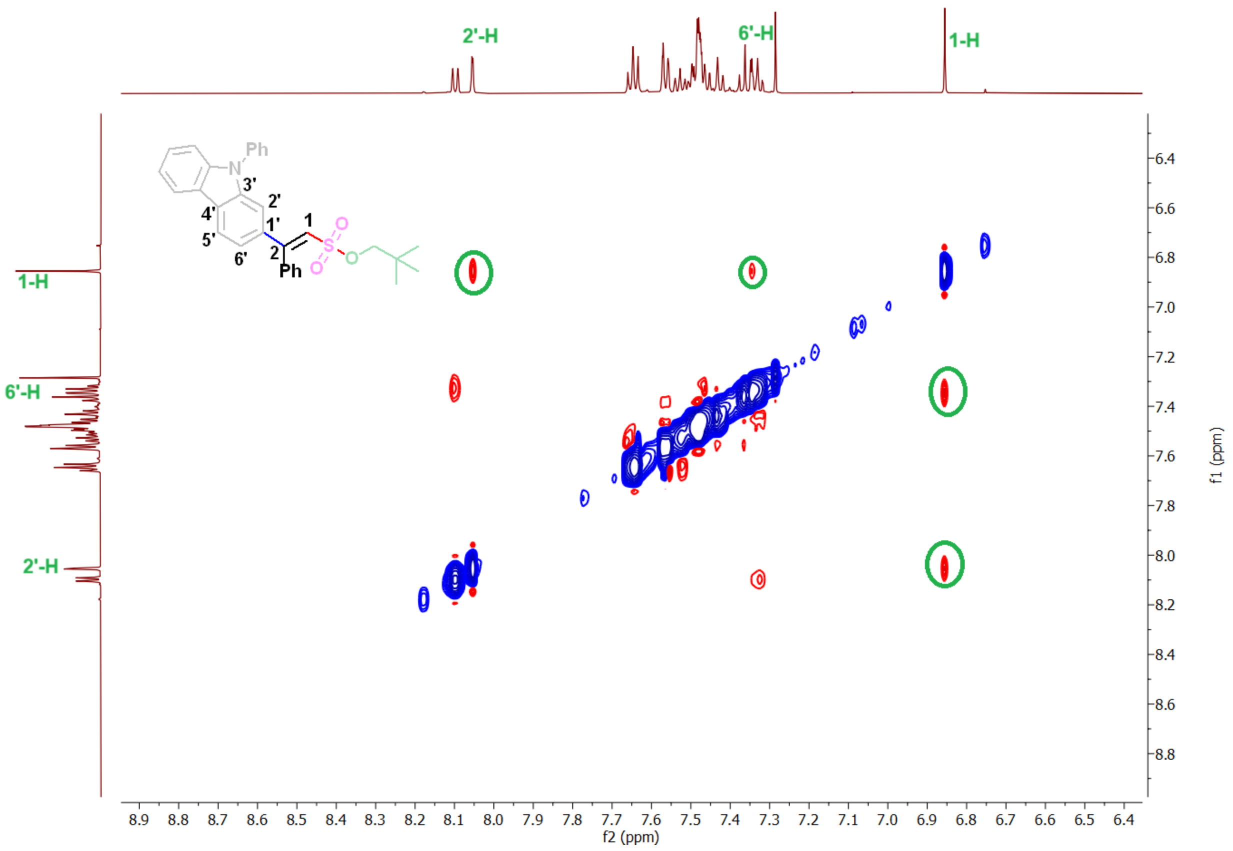

^{1}H NMR (400 MHz, $CDCl_3$) of **5h** (see procedure)

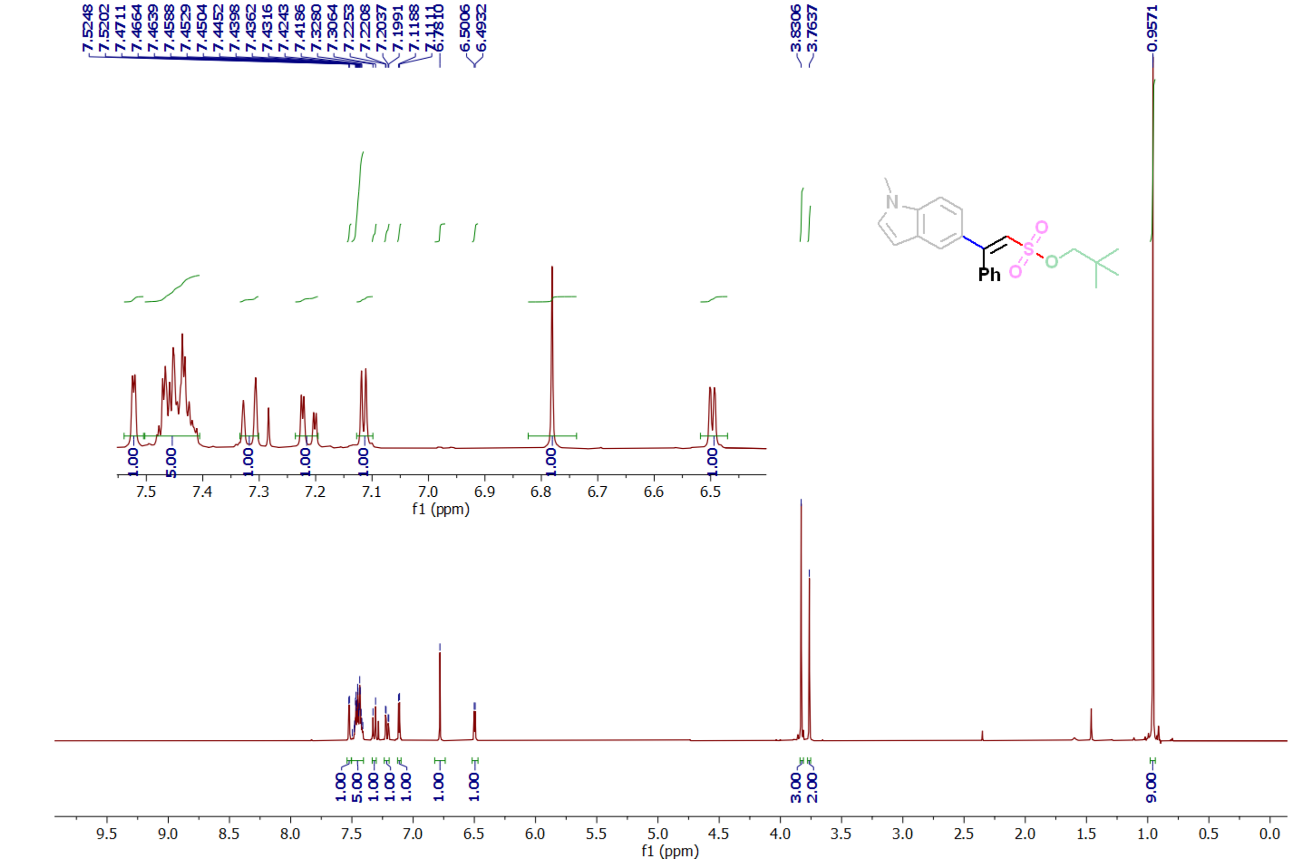

^{13}C NMR (101MHz, $CDCl_3$) of **5h**

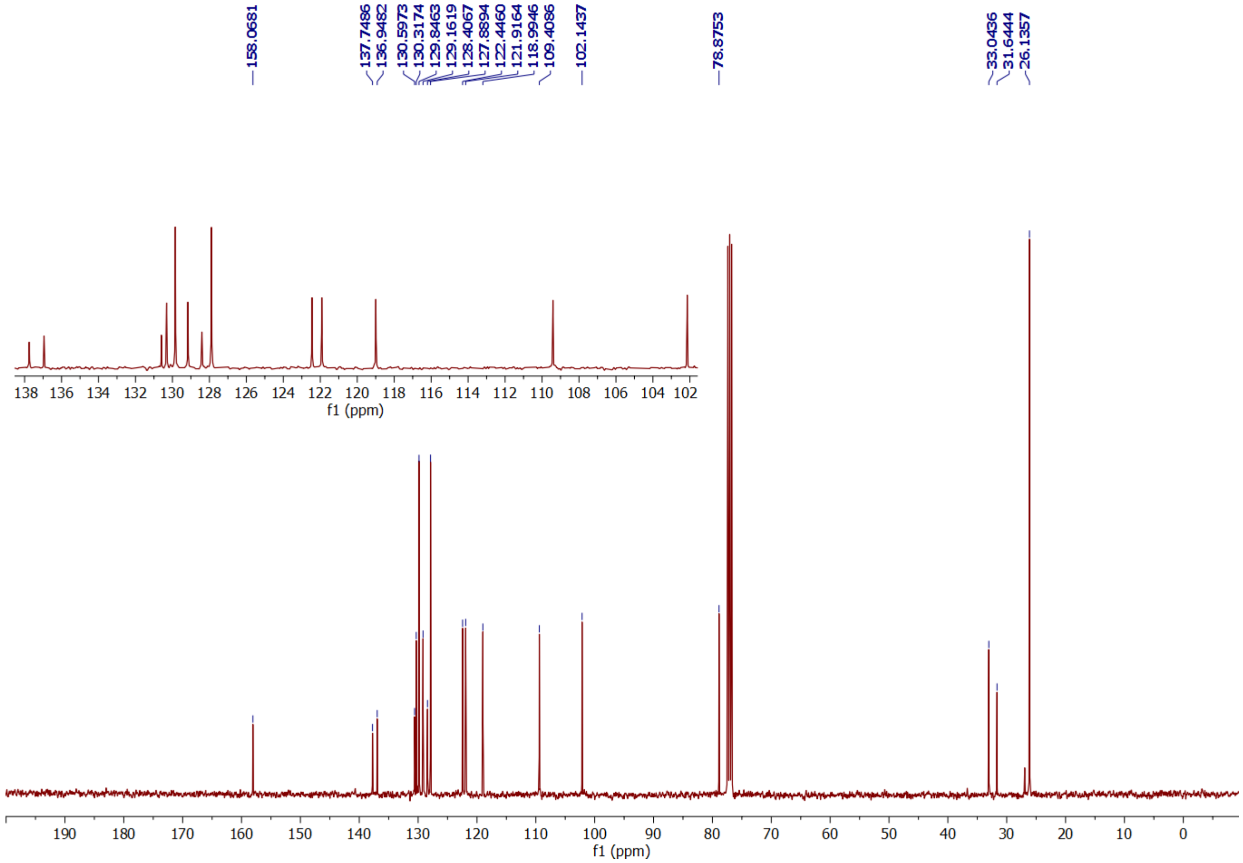

NOE Spectrum for **5h**

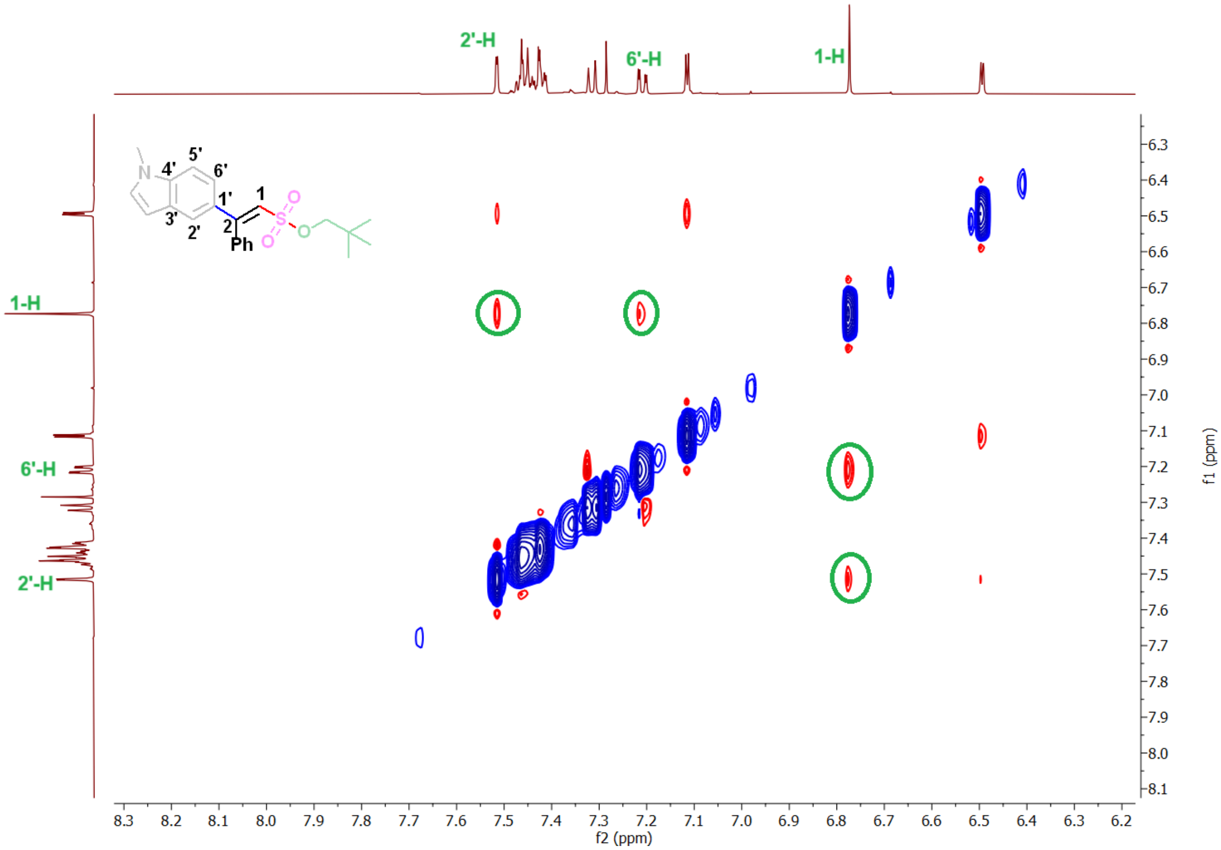

^{1}H NMR (300 MHz, $CDCl_3$) of **5i** (see procedure)

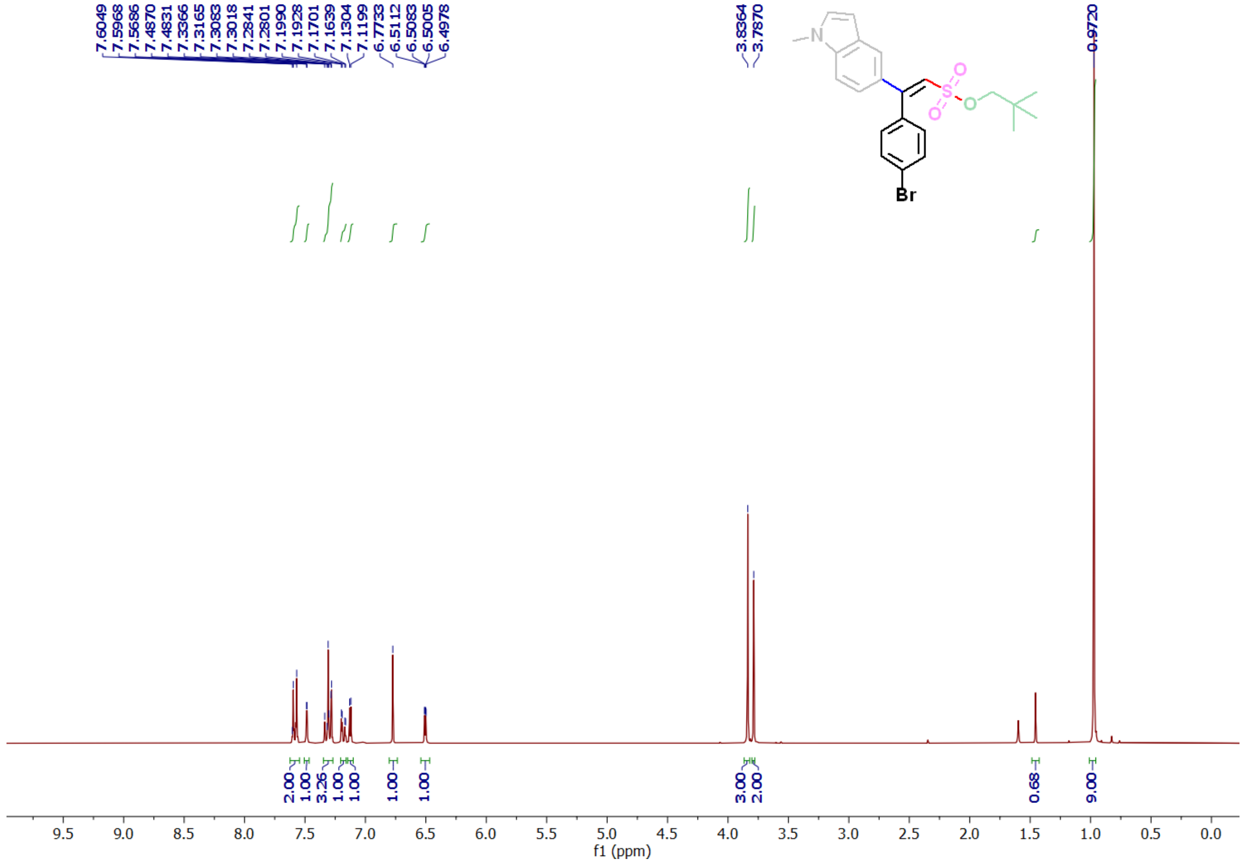

^{13}C NMR (75 MHz, $CDCl_3$) of **5i**

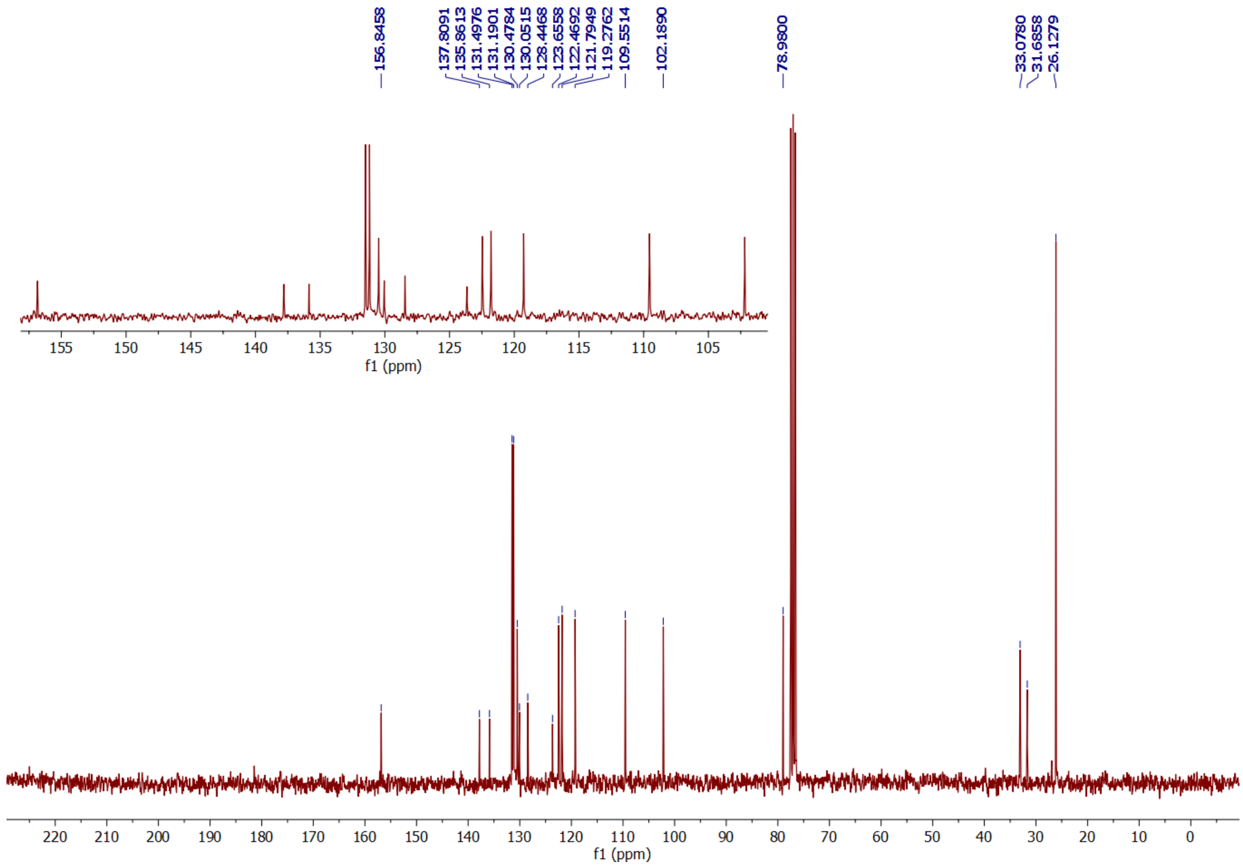

NOE Spectrum for **5i**

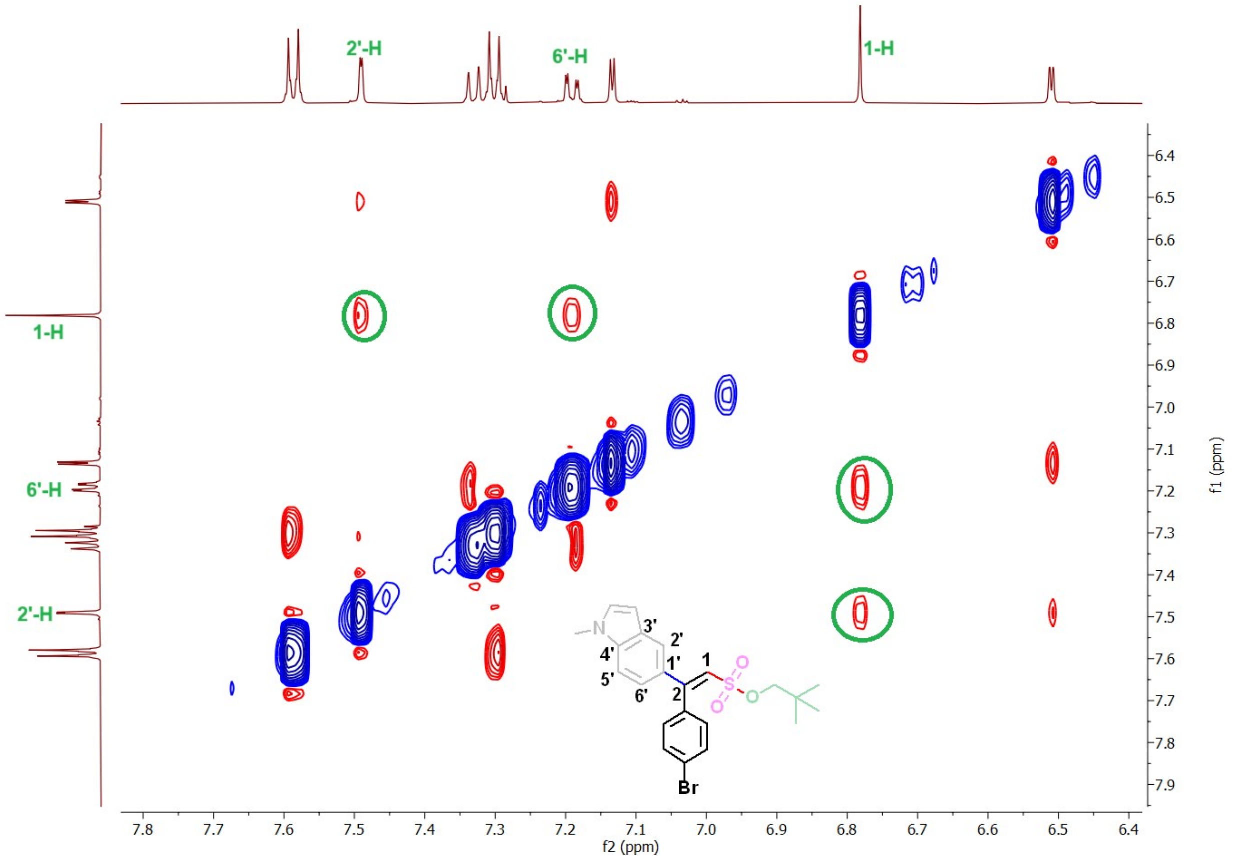

^{1}H NMR (400 MHz, $CDCl_3$) of **5j** (see procedure)

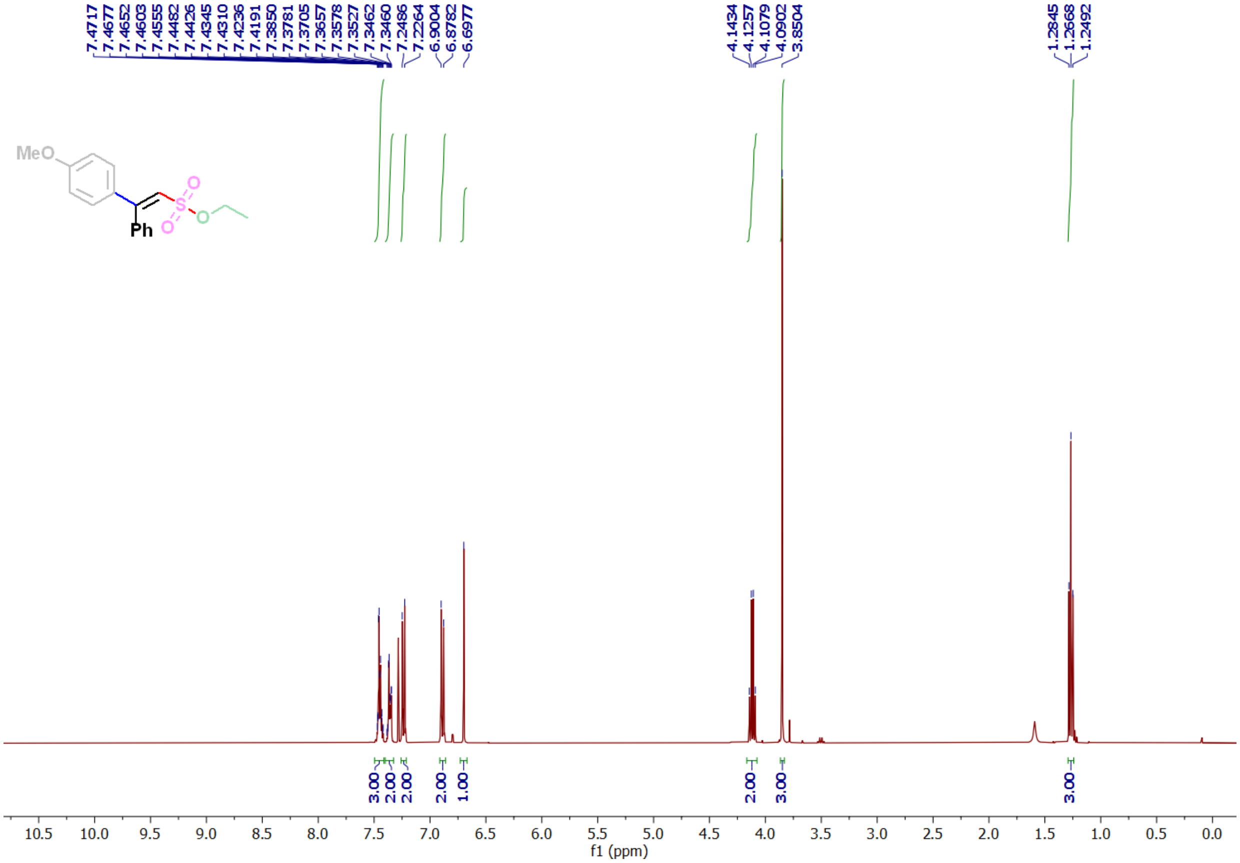

^{13}C NMR (101MHz, $CDCl_3$) of **5j**

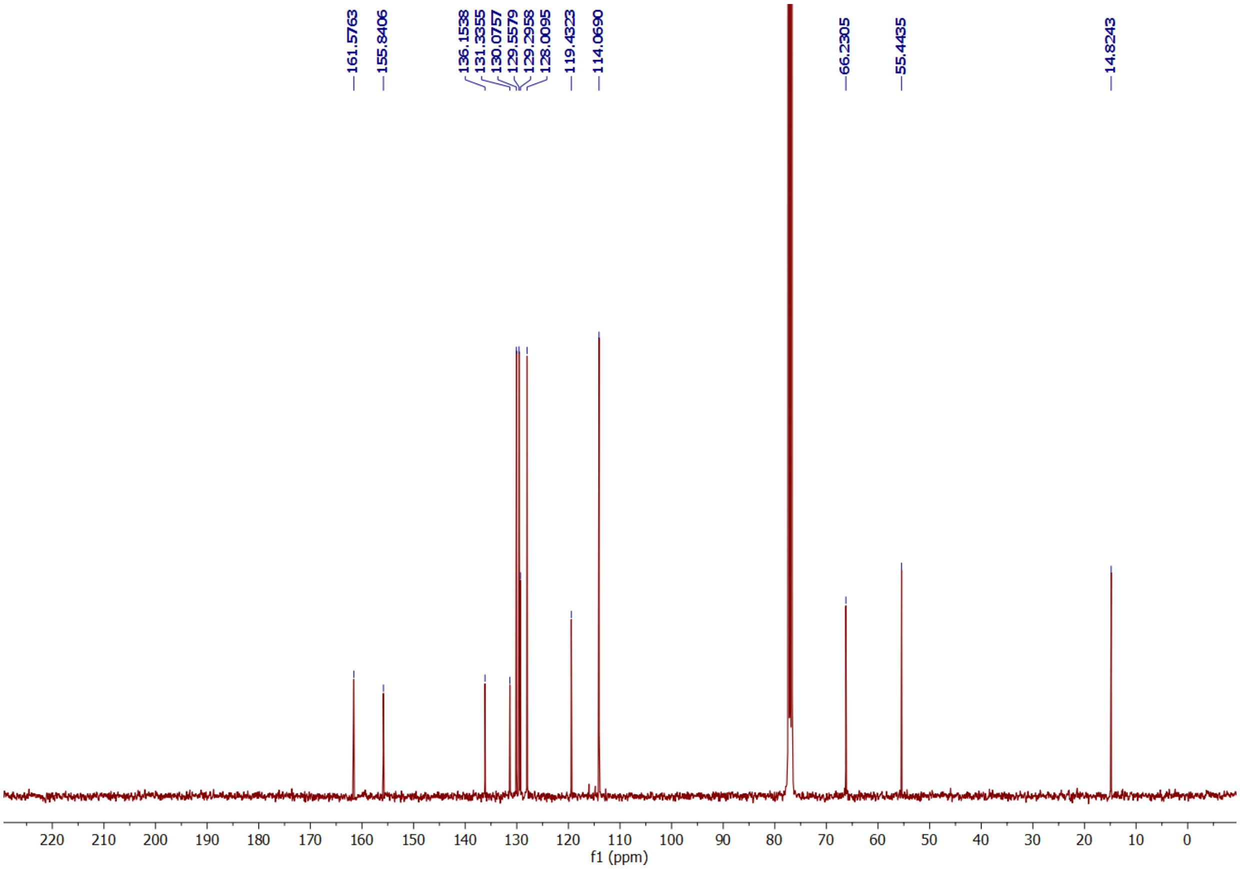

NOE Spectrum for **5j**

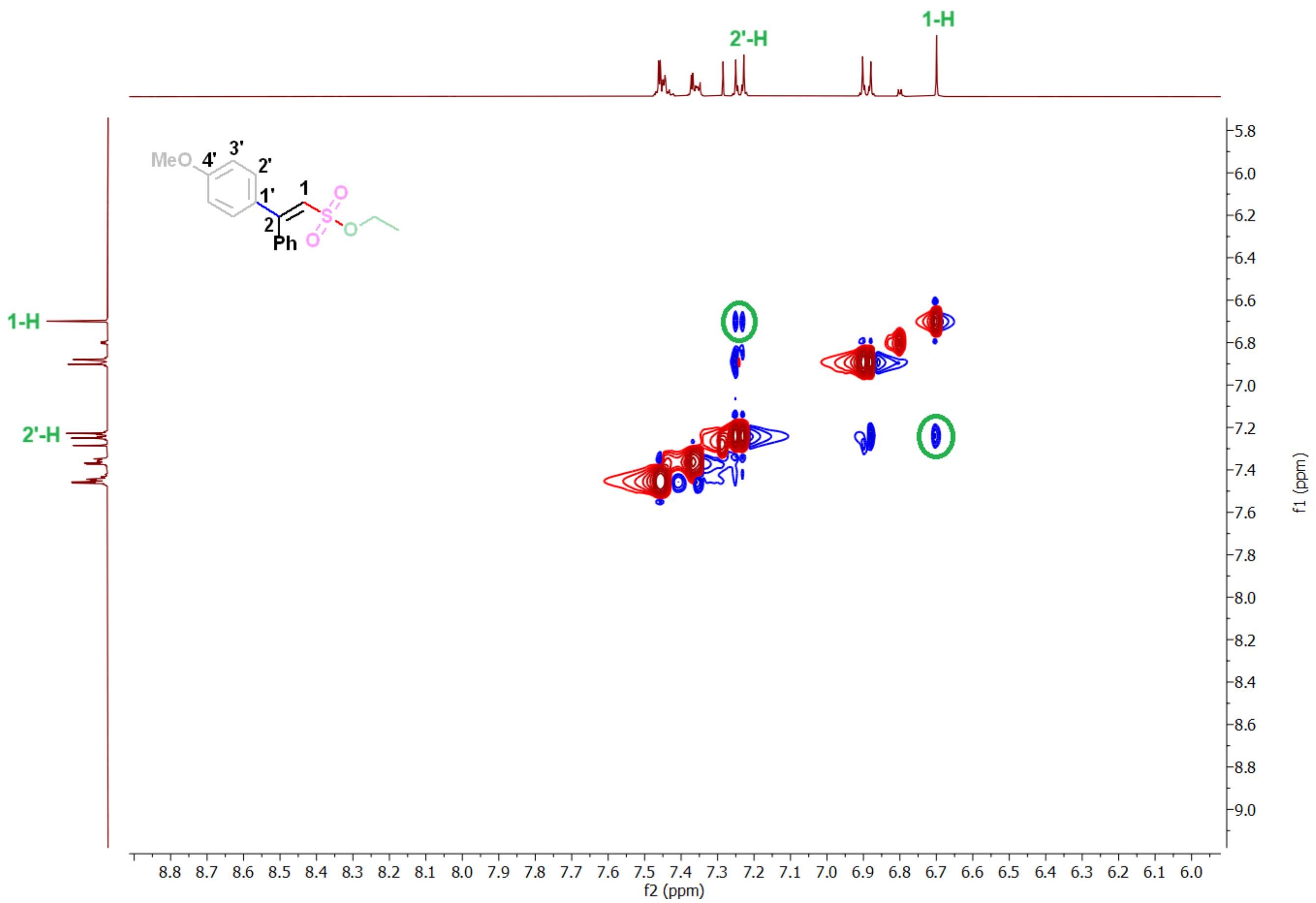

^{1}H NMR (400 MHz, $CDCl_3$) of **5k** (see procedure)

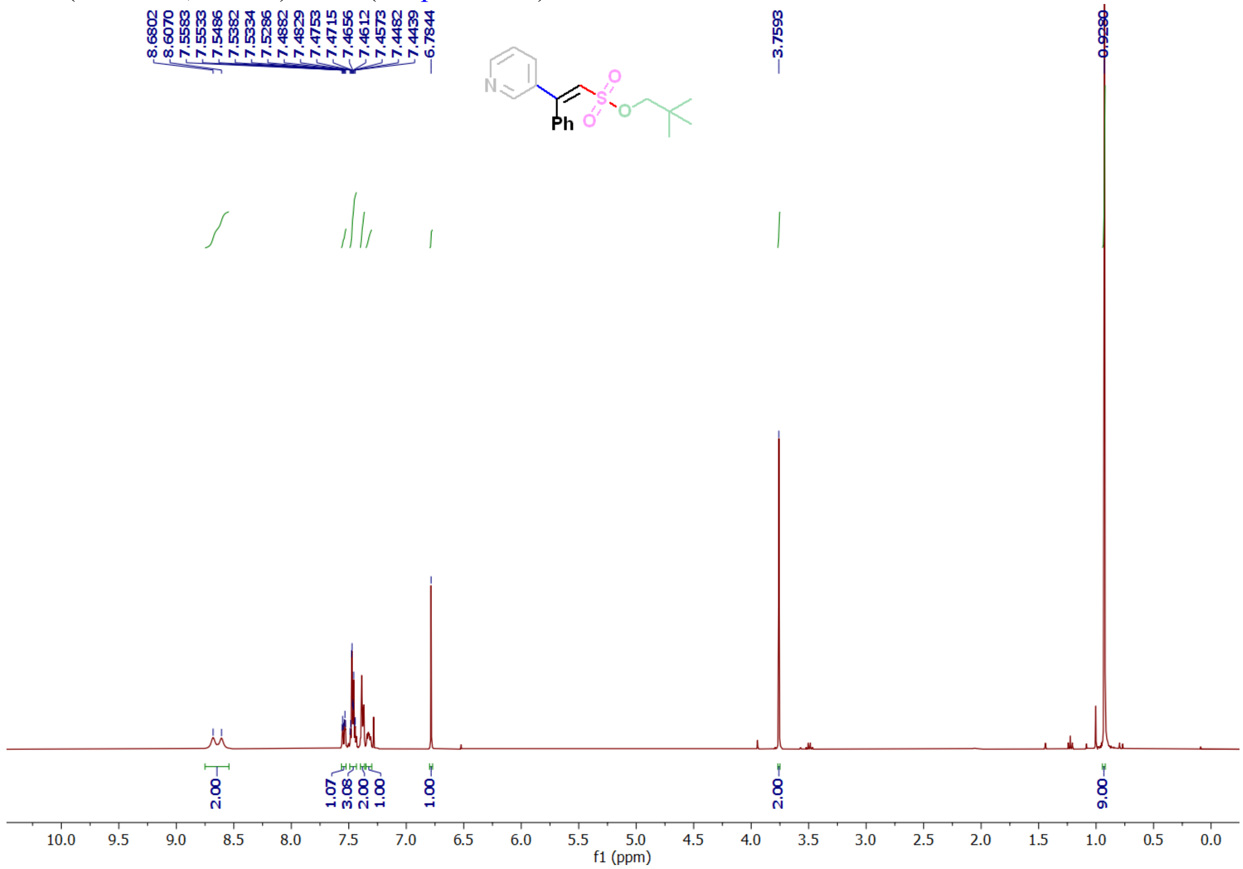

^{13}C NMR (101MHz, $CDCl_3$) of **5k**

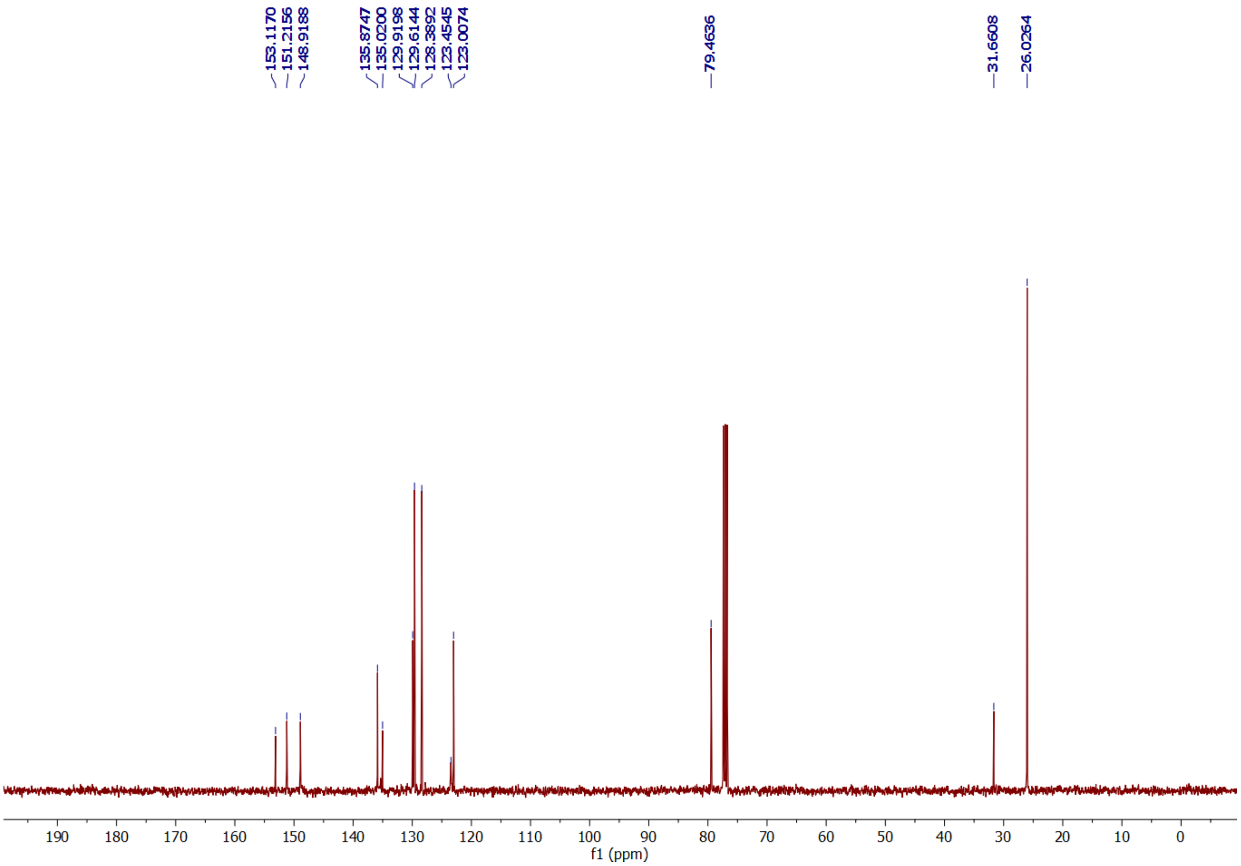

^{1}H NMR (400 MHz, $CDCl_3$) of **5l** (see procedure)

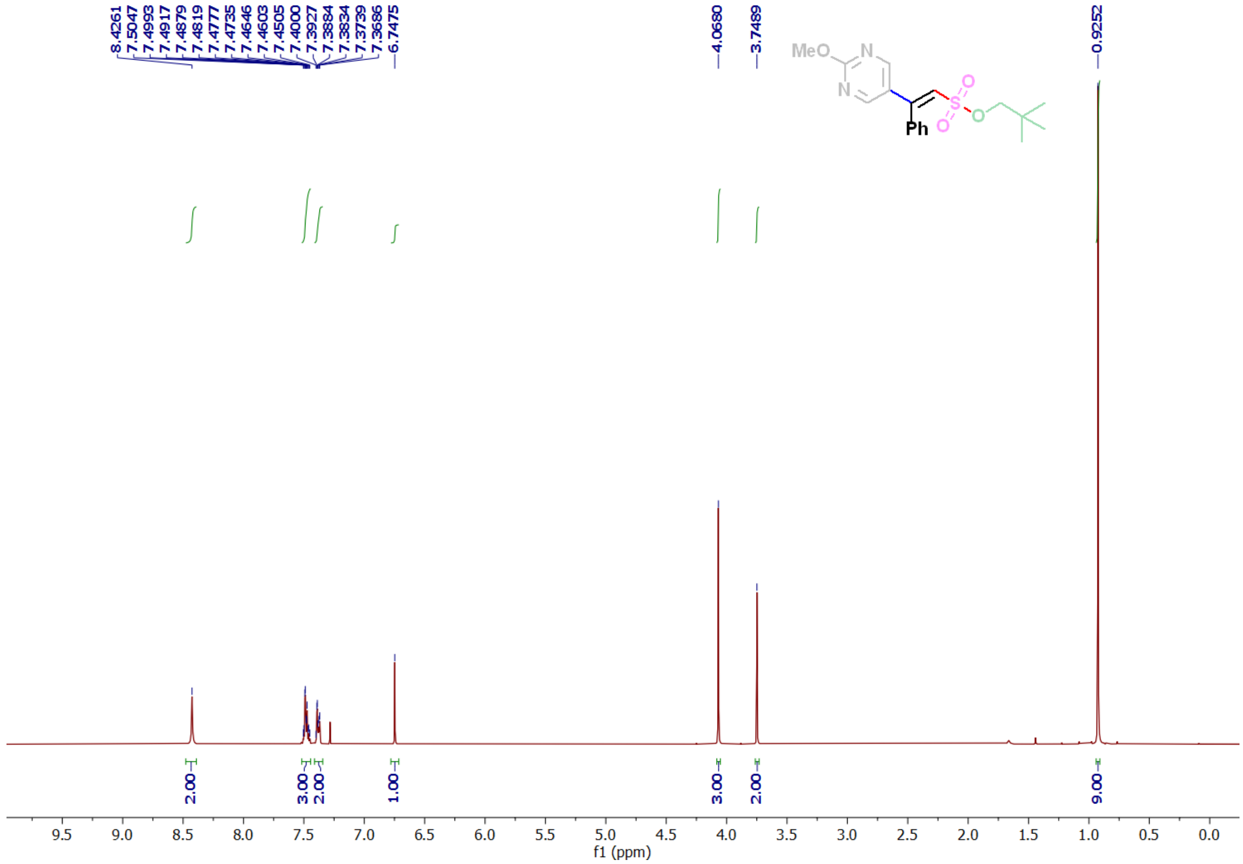

^{13}C NMR (101MHz, $CDCl_3$) of **5l**

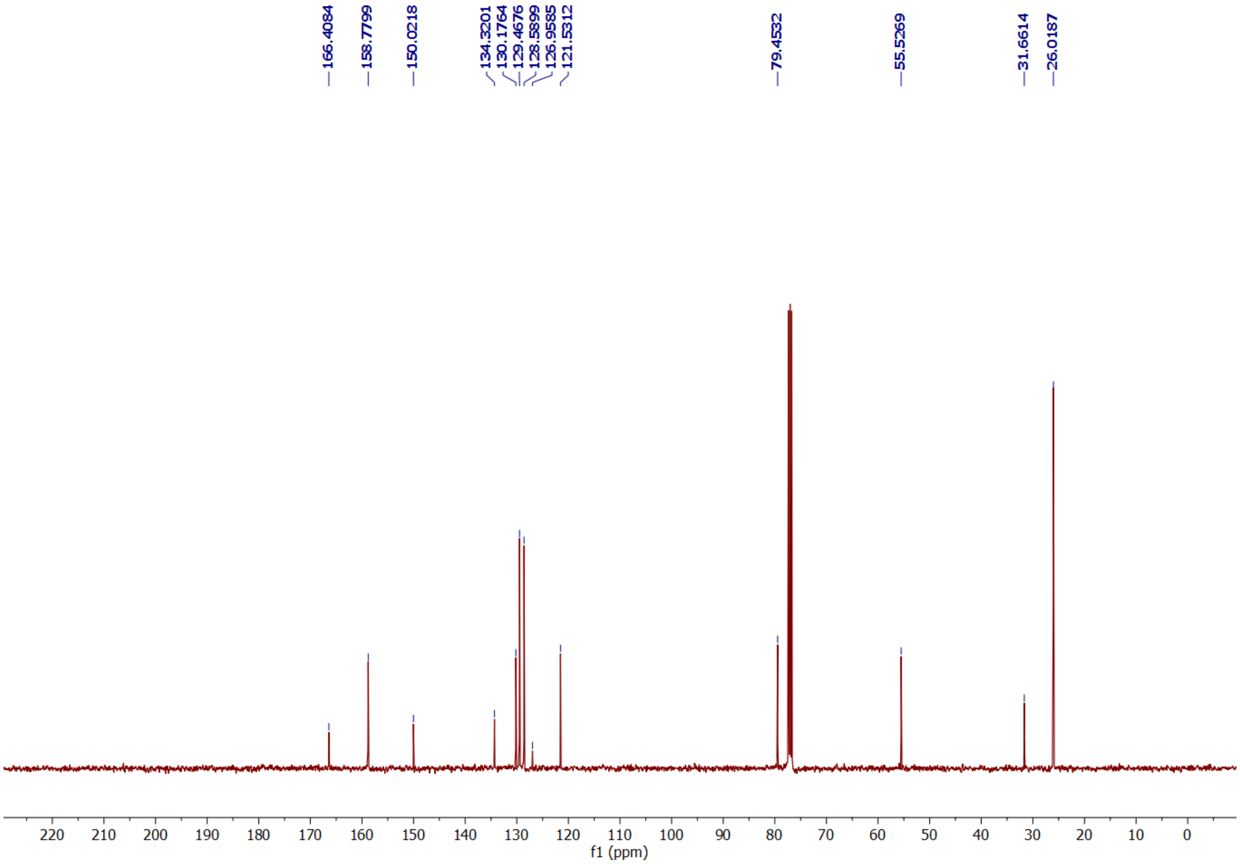

NOE Spectrum for **5l**

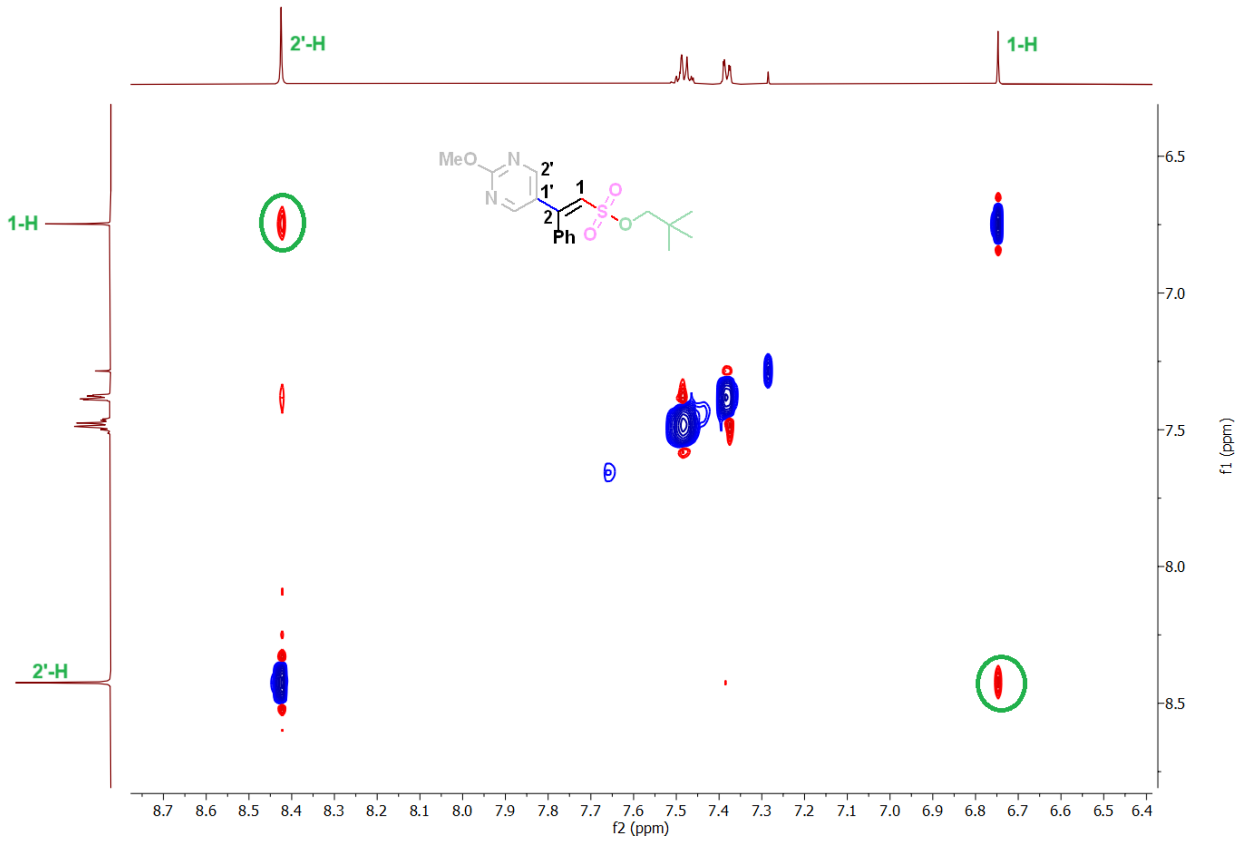

^{1}H NMR (400 MHz, $CDCl_3$) of **5m** (see procedure)

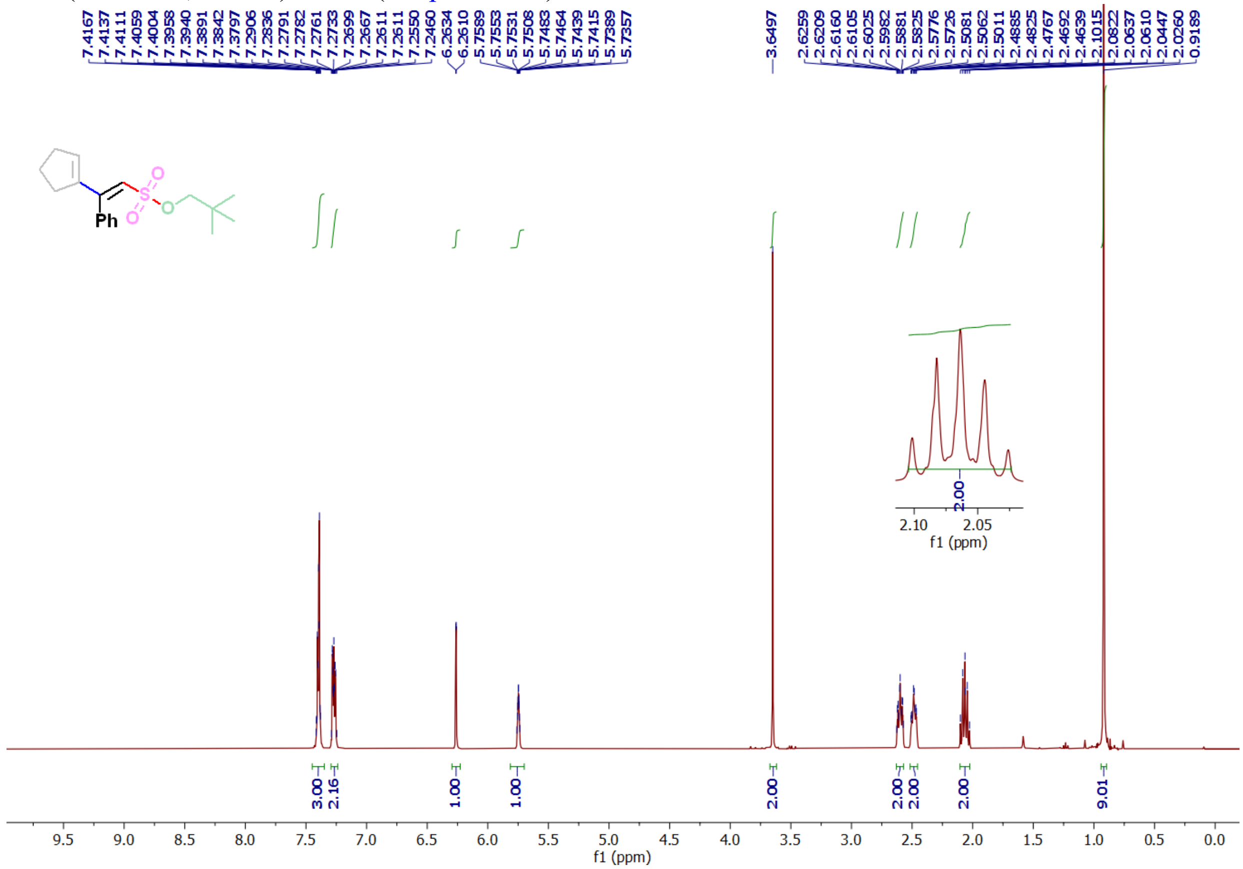

^{13}C NMR (101MHz, $CDCl_3$) of **5m**

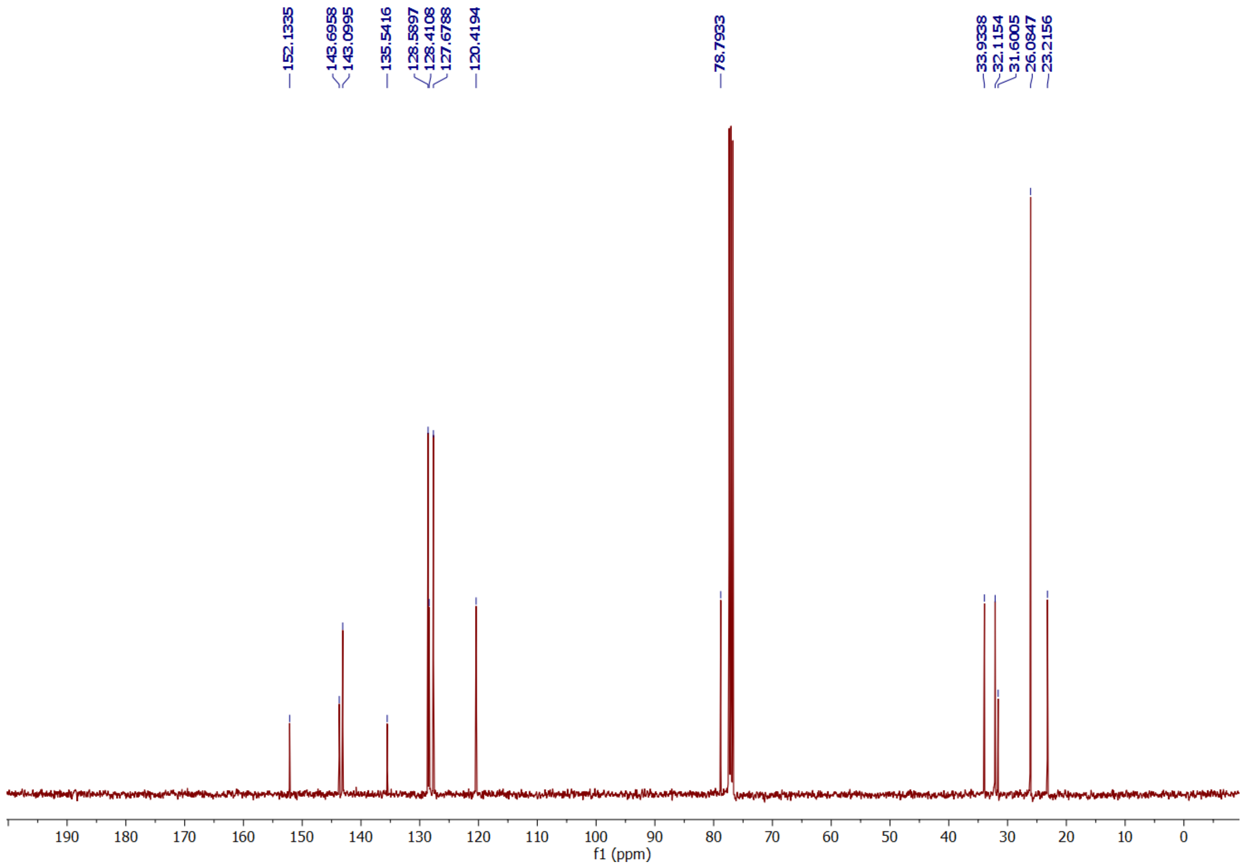

NOE Spectrum for **5m**

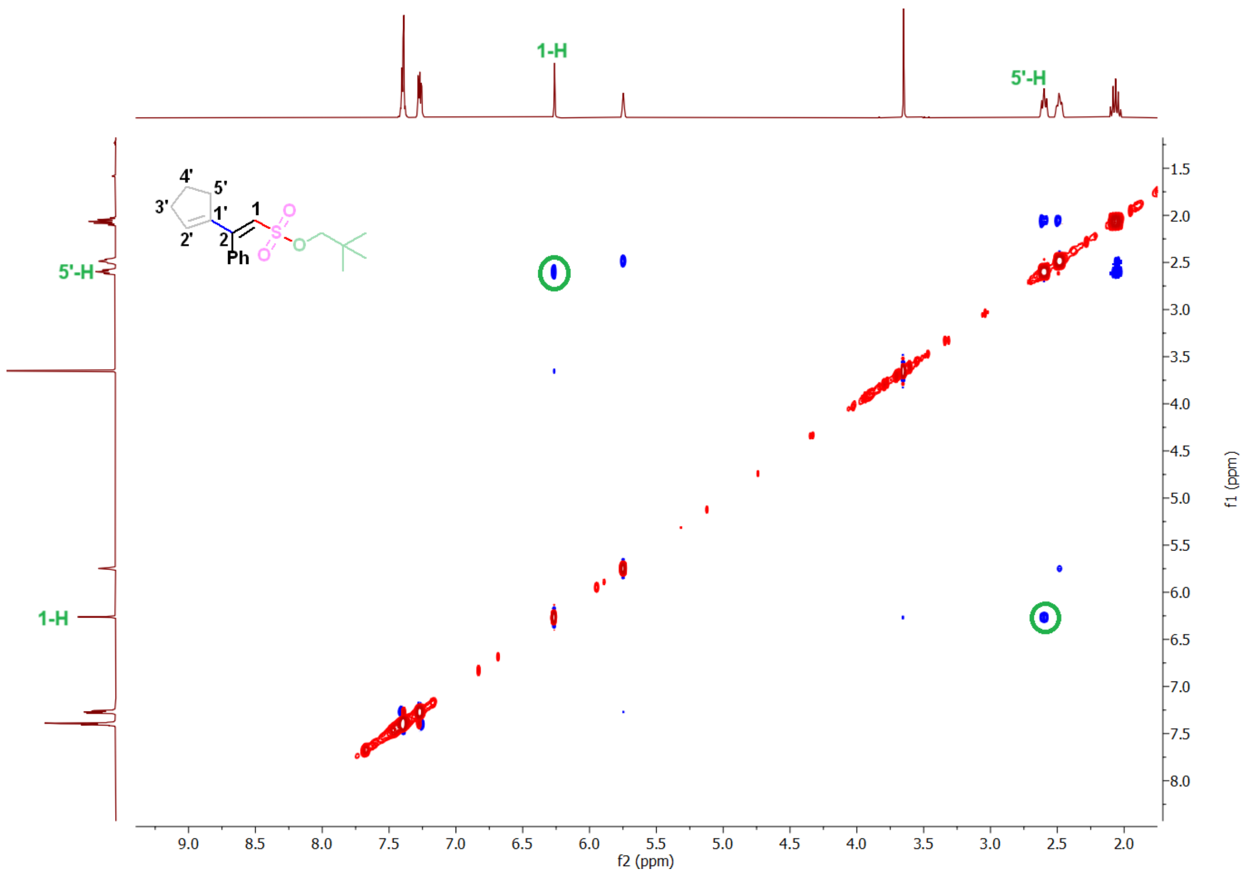

^{1}H NMR (400 MHz, $CDCl_3$) of **5n** (see procedure)

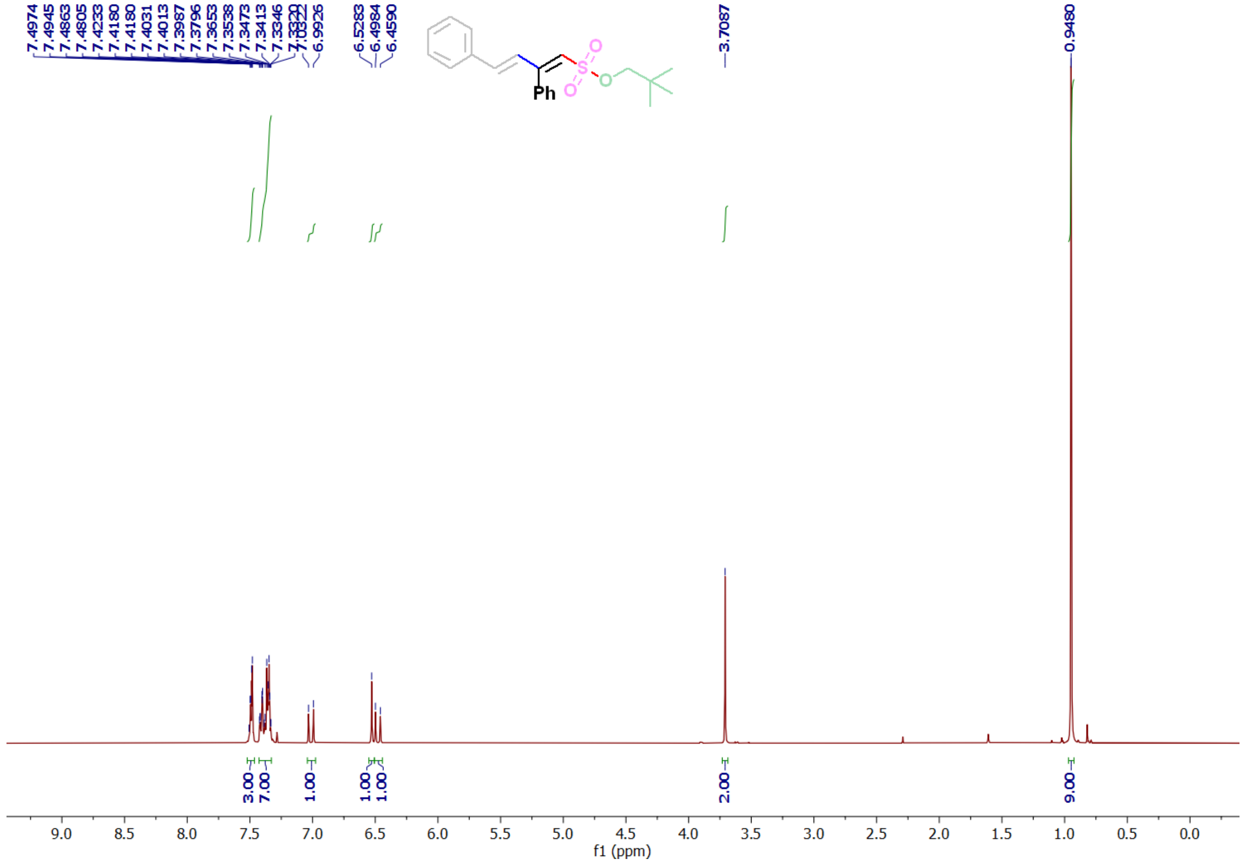

^{13}C NMR (101MHz, $CDCl_3$) of **5n**

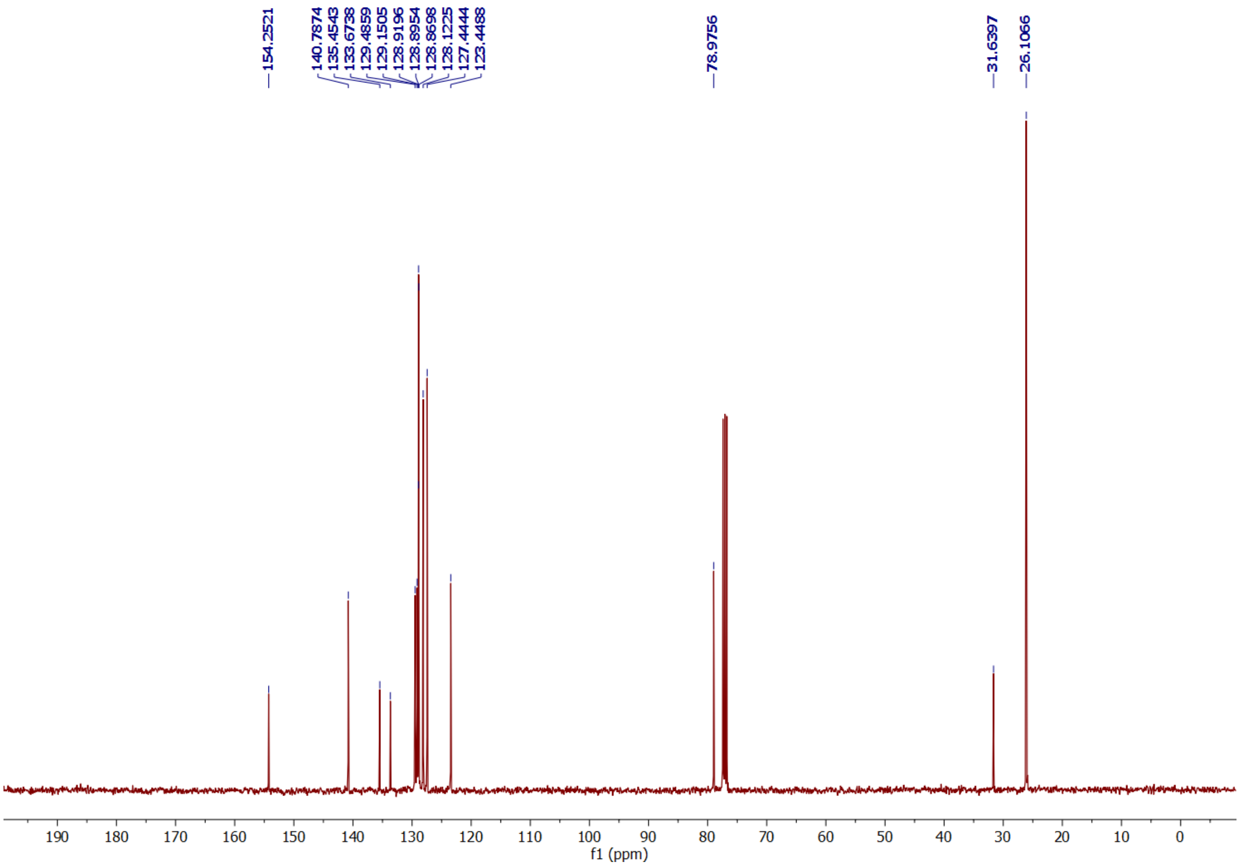

NOE Spectrum for **5n**

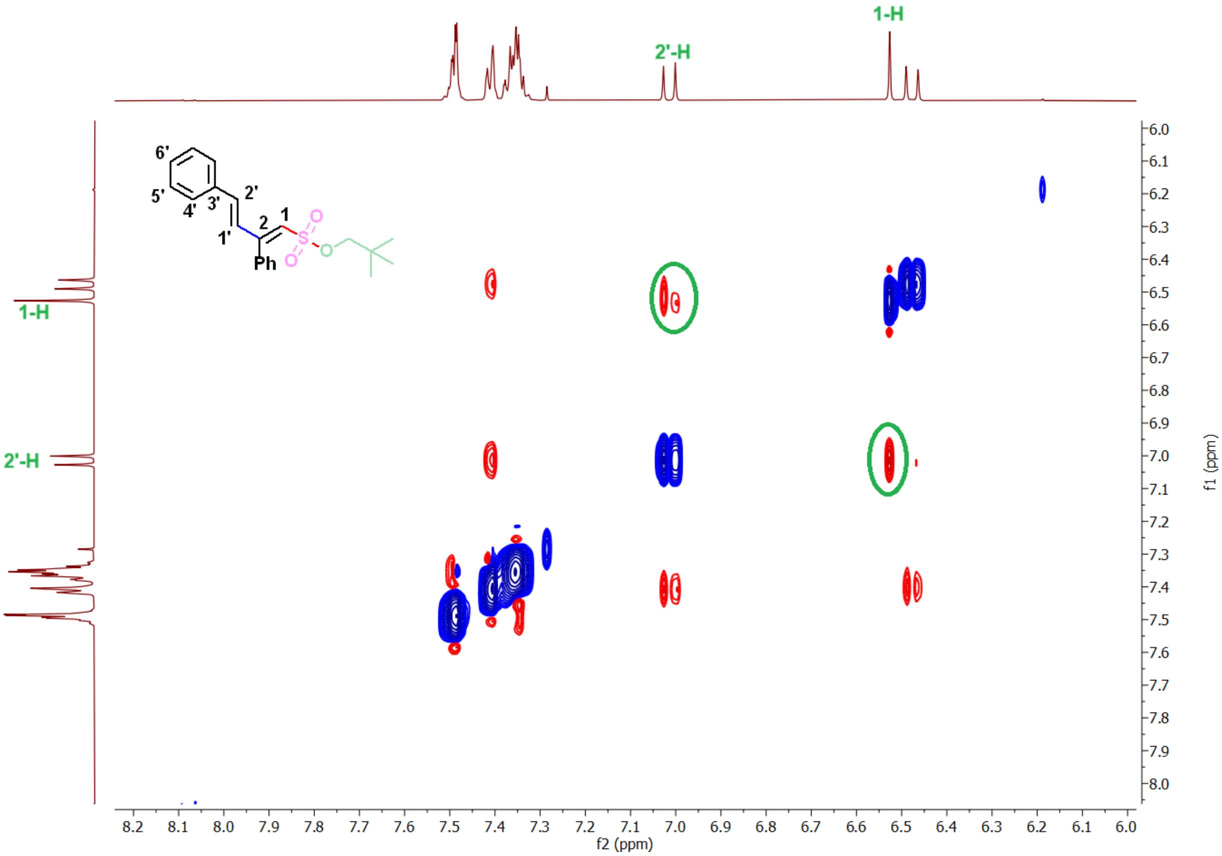

^{1}H NMR (400 MHz, $CDCl_3$) of **5o** (see procedure)

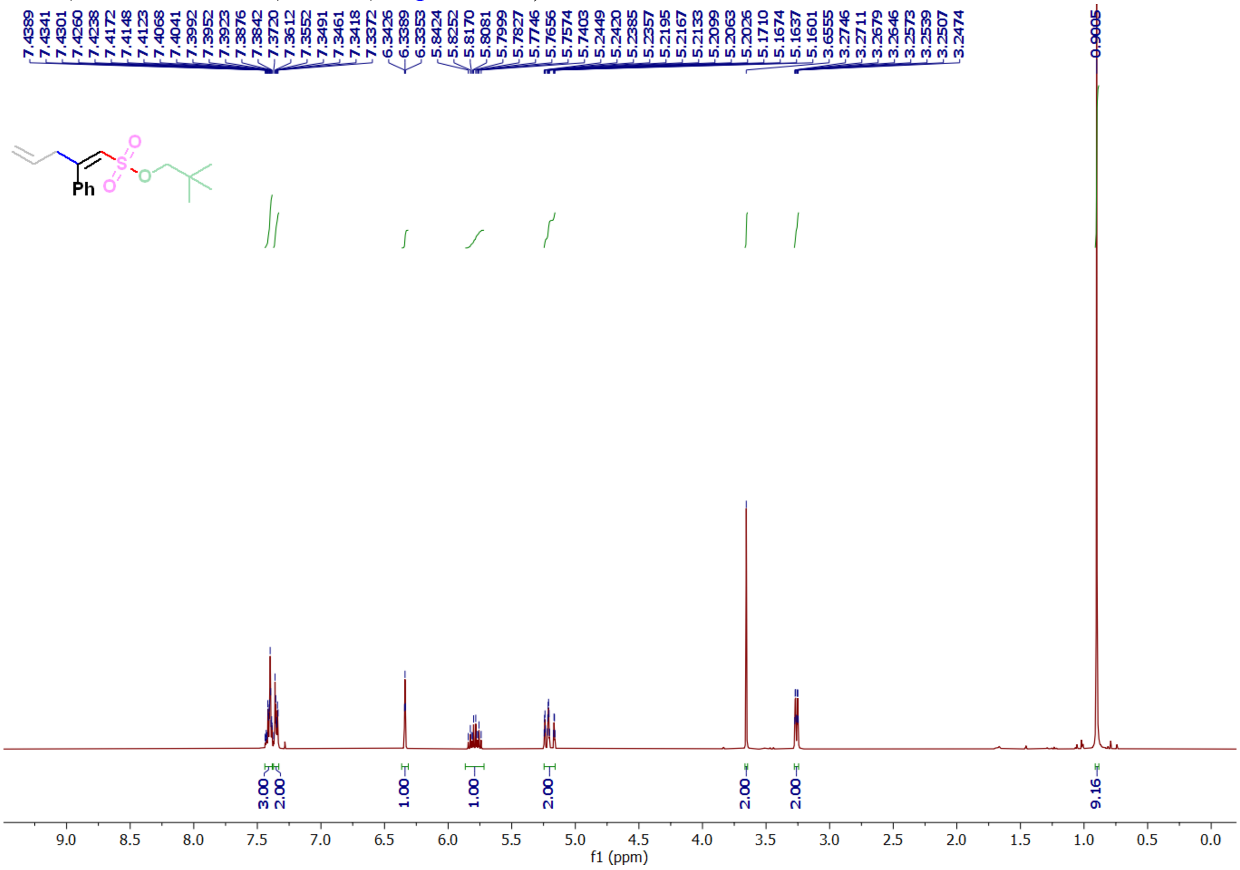

^{13}C NMR (101MHz, $CDCl_3$) of **5o**

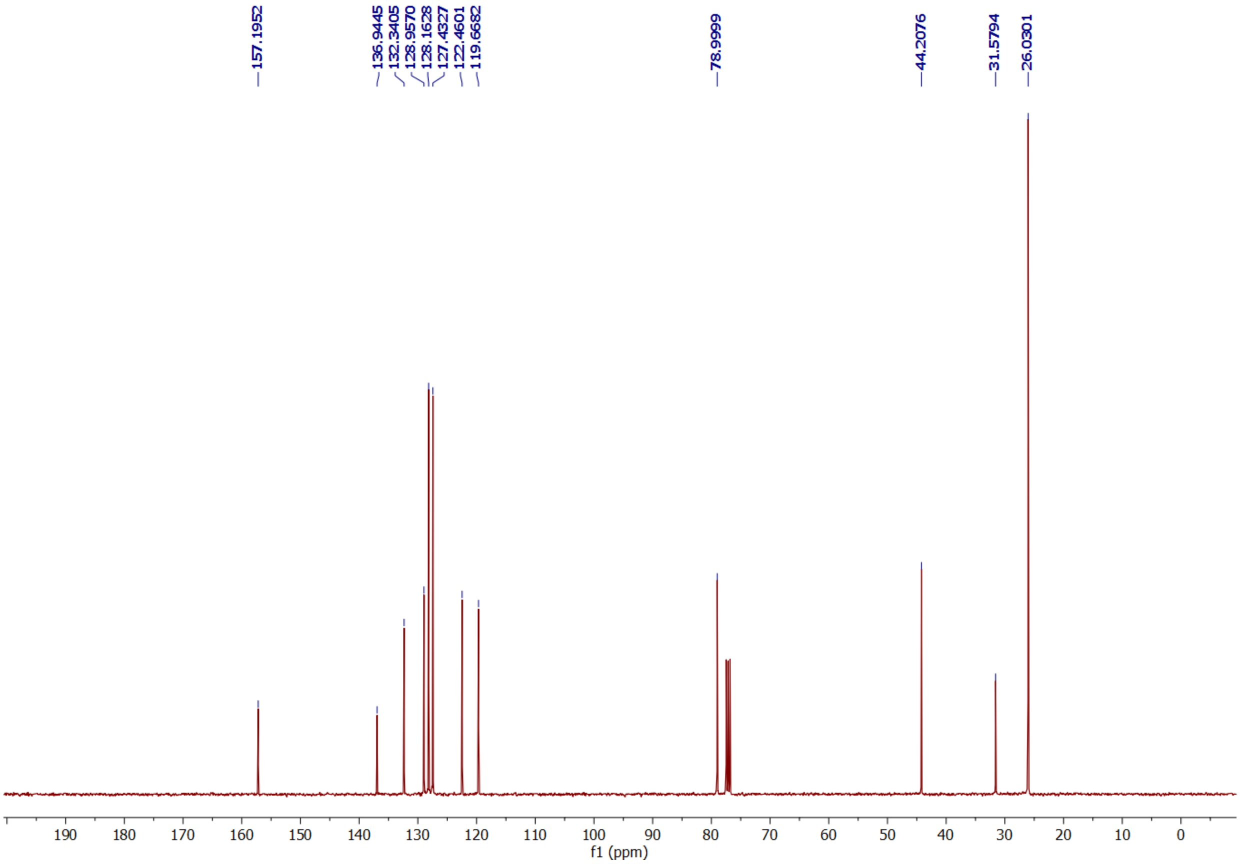

NOE Spectrum for **5o**

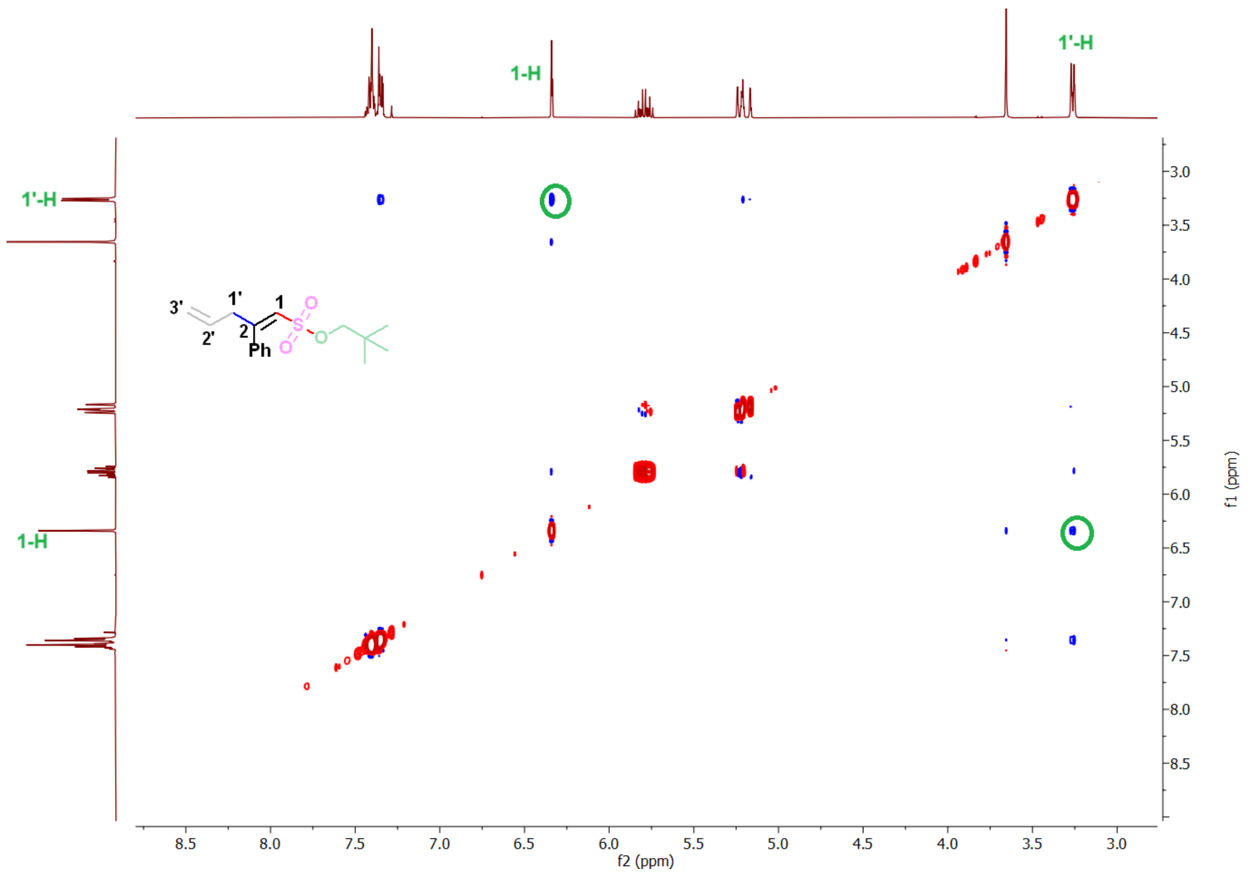

^{1}H NMR (400 MHz, $CDCl_3$) of **5p** (see procedure)

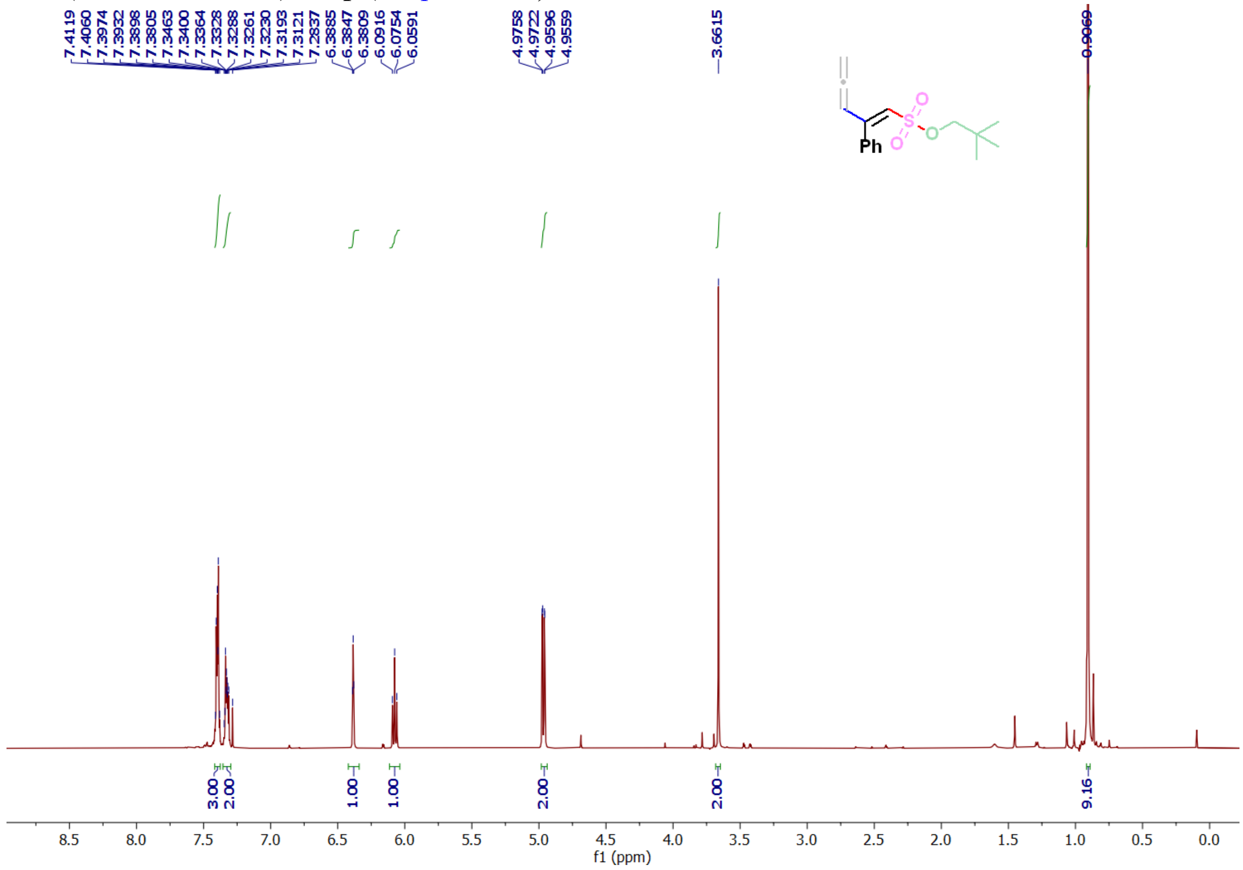

^{13}C NMR (101MHz, $CDCl_3$) of **5p**

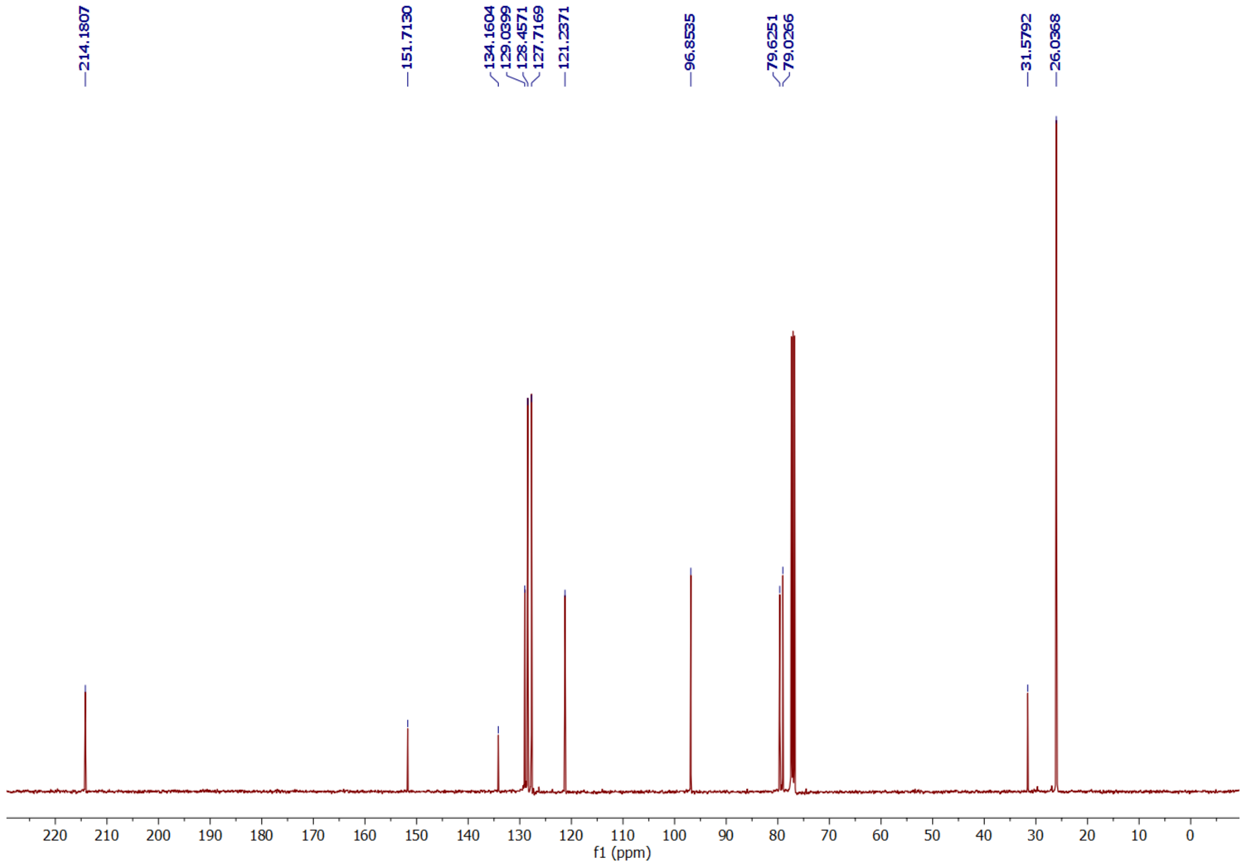

NOE Spectrum for **5p**

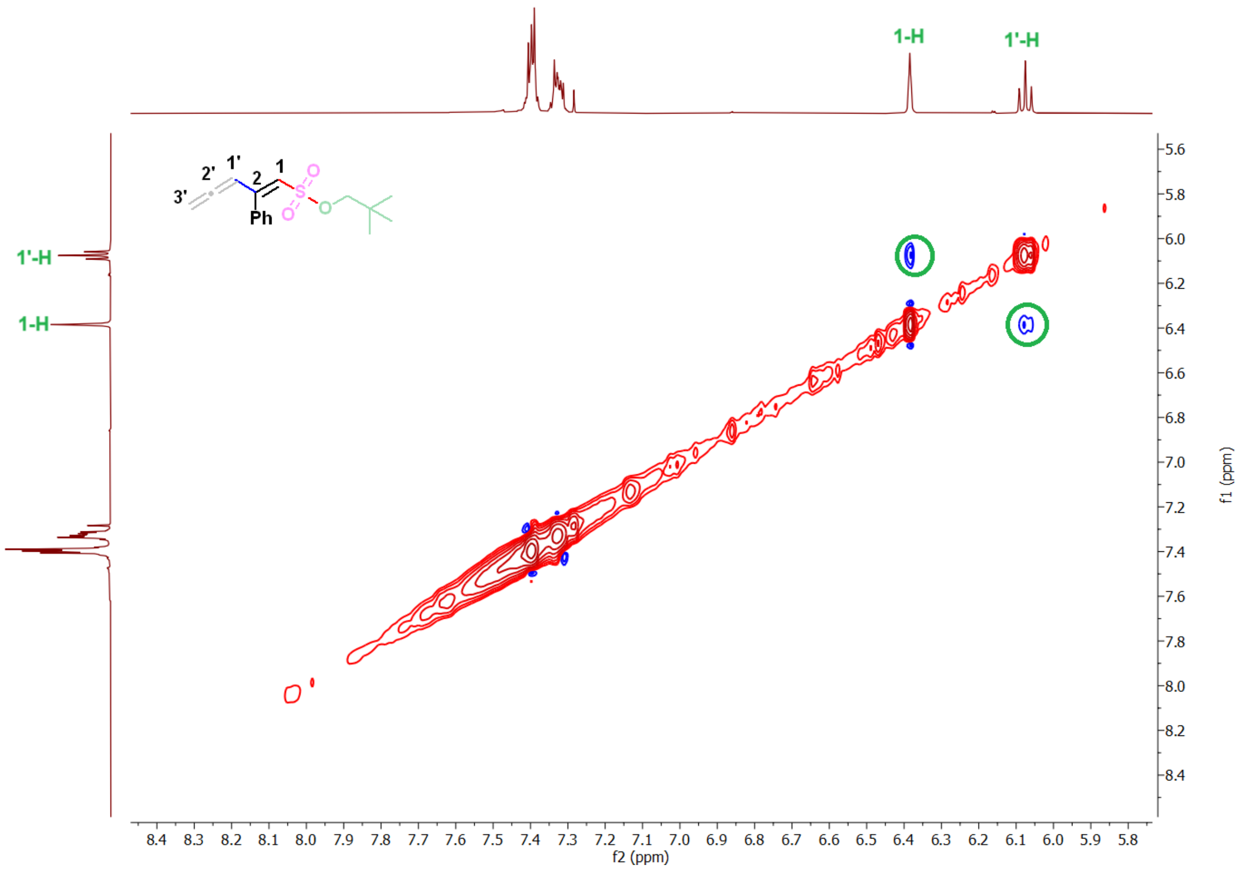

^{1}H NMR (400 MHz, $CDCl_3$) of **5q** (see procedure)

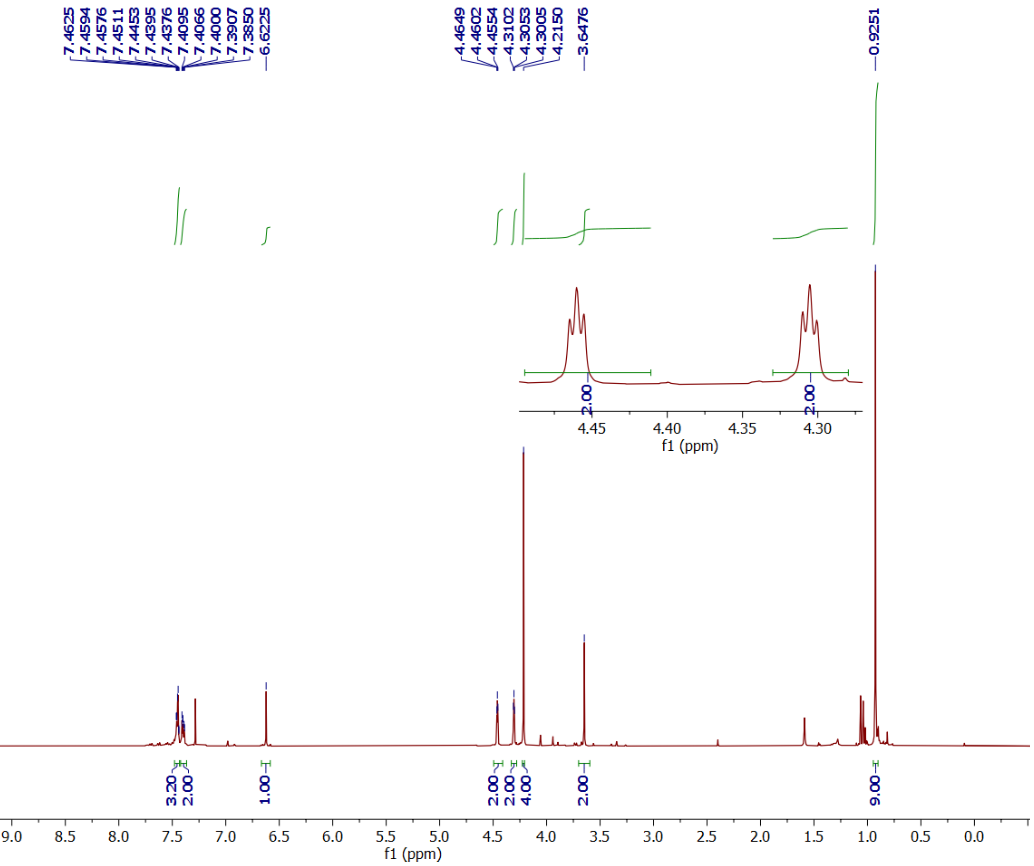

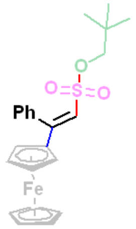

^{13}C NMR (101MHz, $CDCl_3$) of **5q**

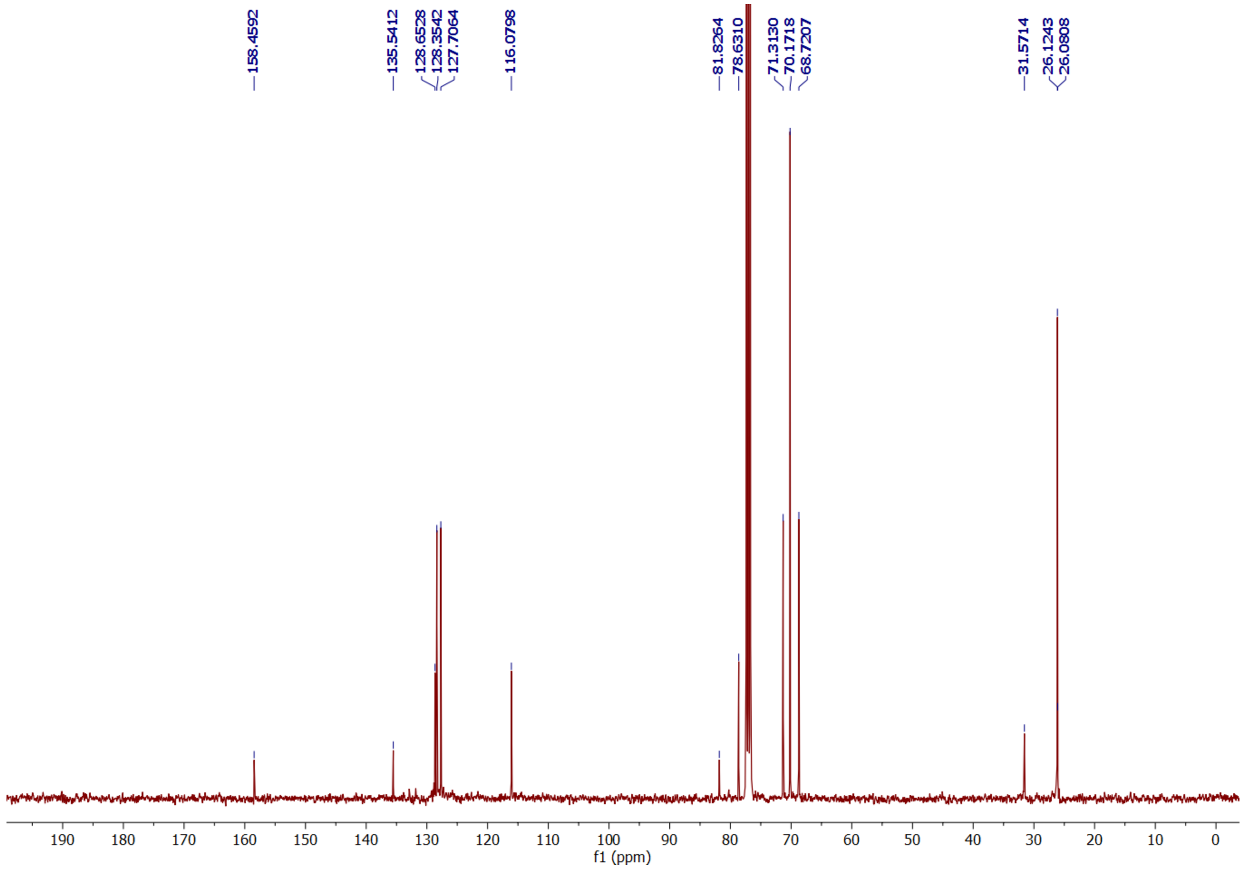

NOE Spectrum for **5q**

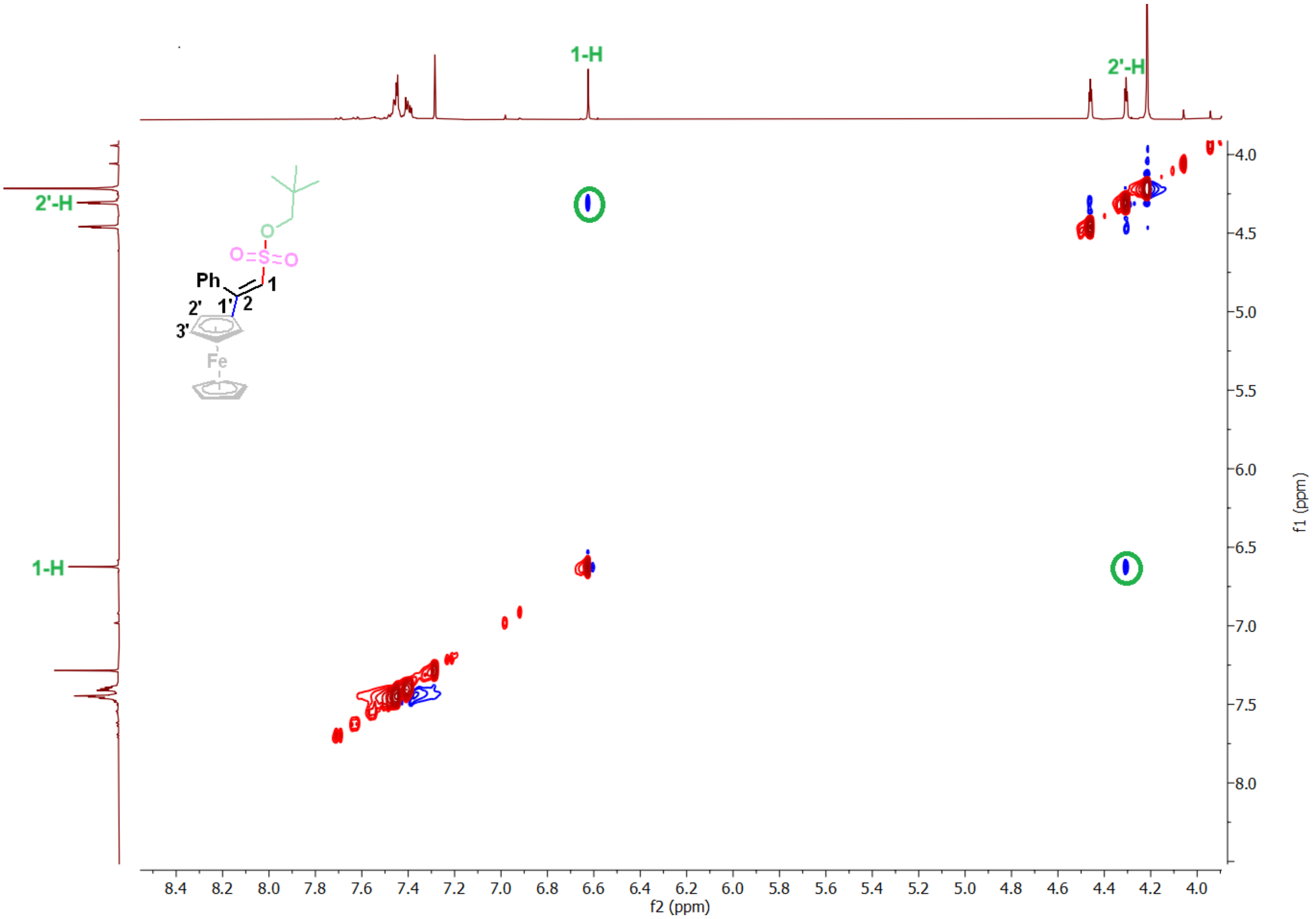

^{1}H NMR (300 MHz, $CDCl_3$) of **5r** (see procedure)

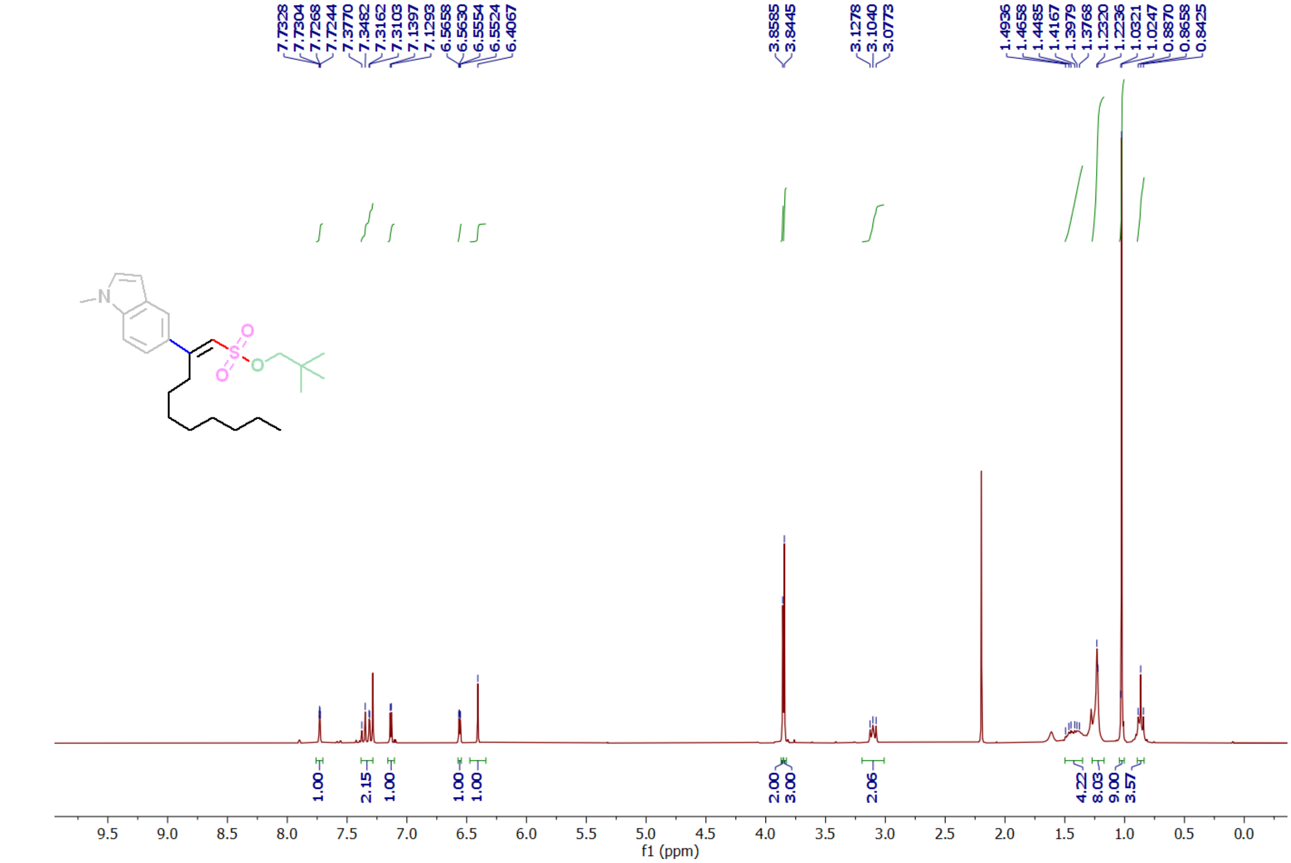

^{13}C NMR (75MHz, $CDCl_3$) of **5r**

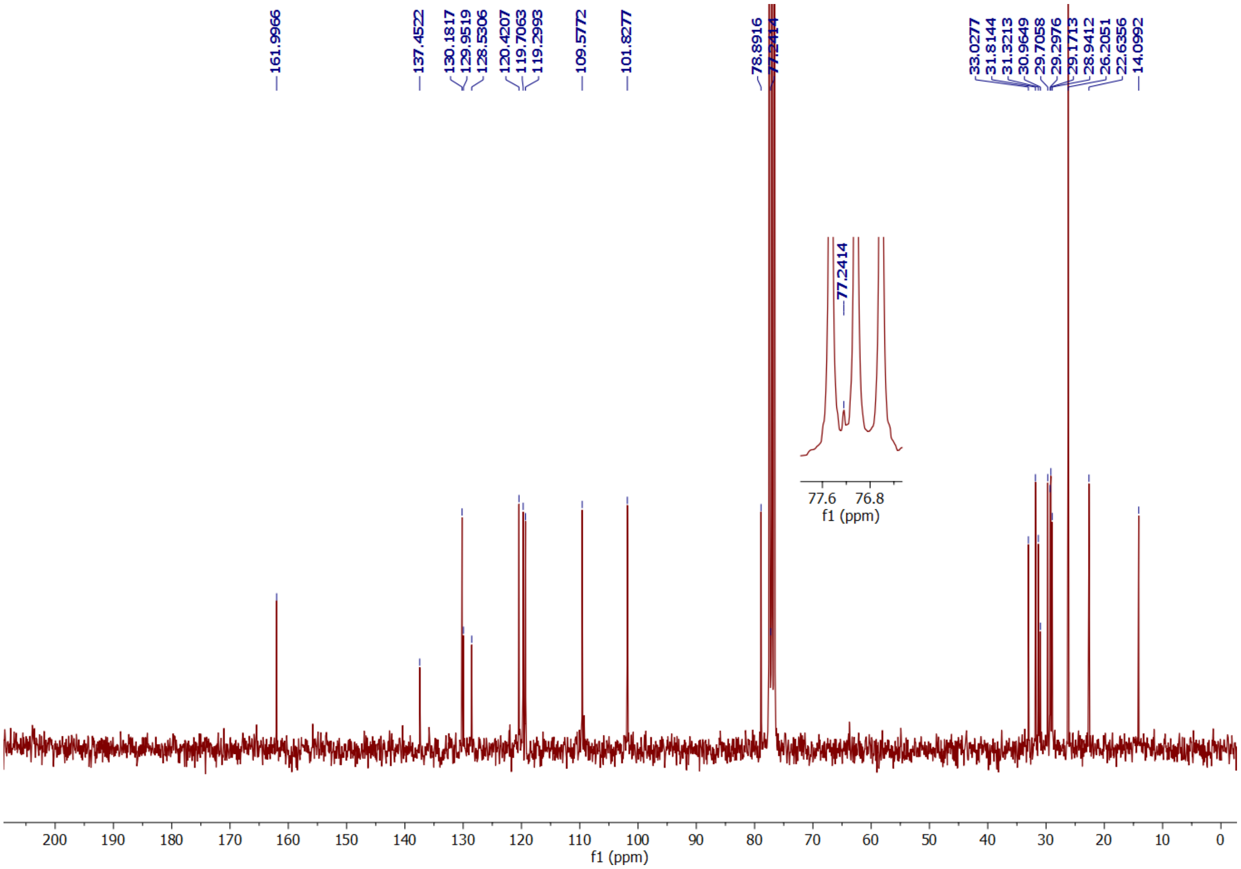

NOE Spectrum for **5r**

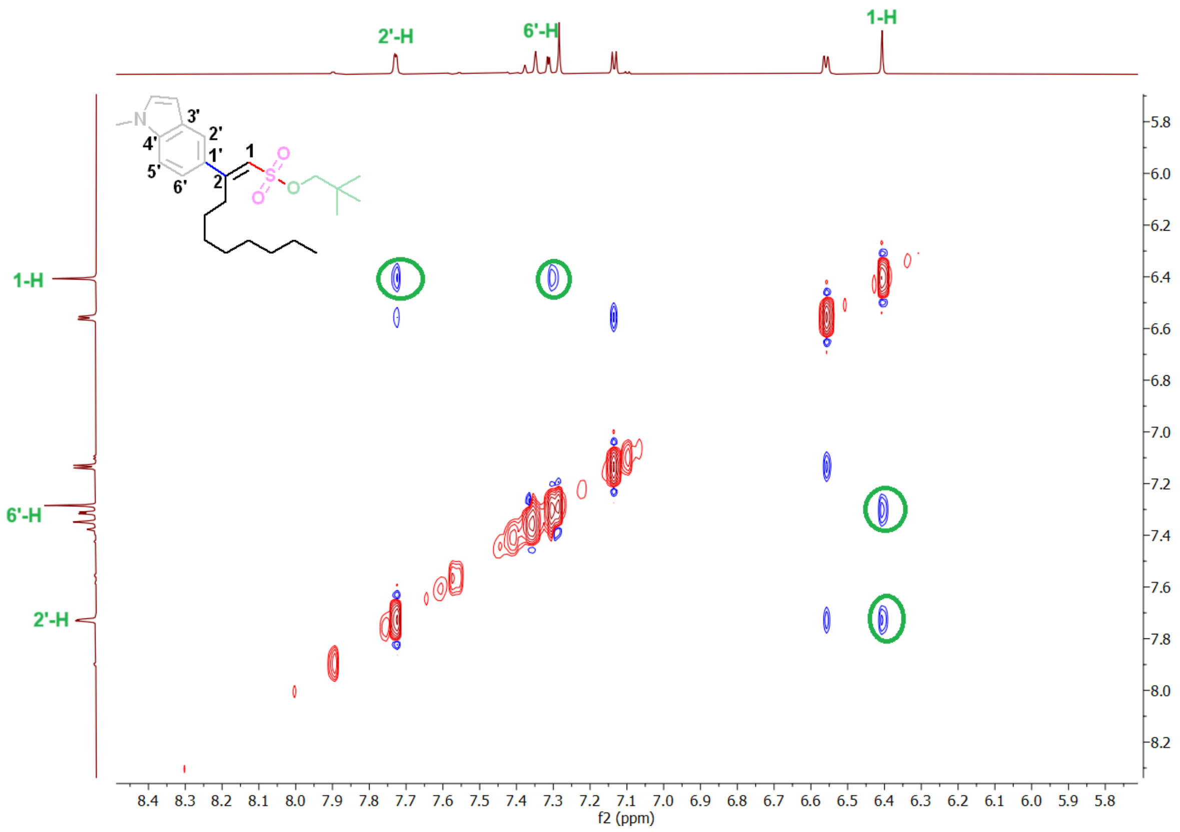

^{1}H NMR (400 MHz, $CDCl_3$) of **5s** (see procedure)

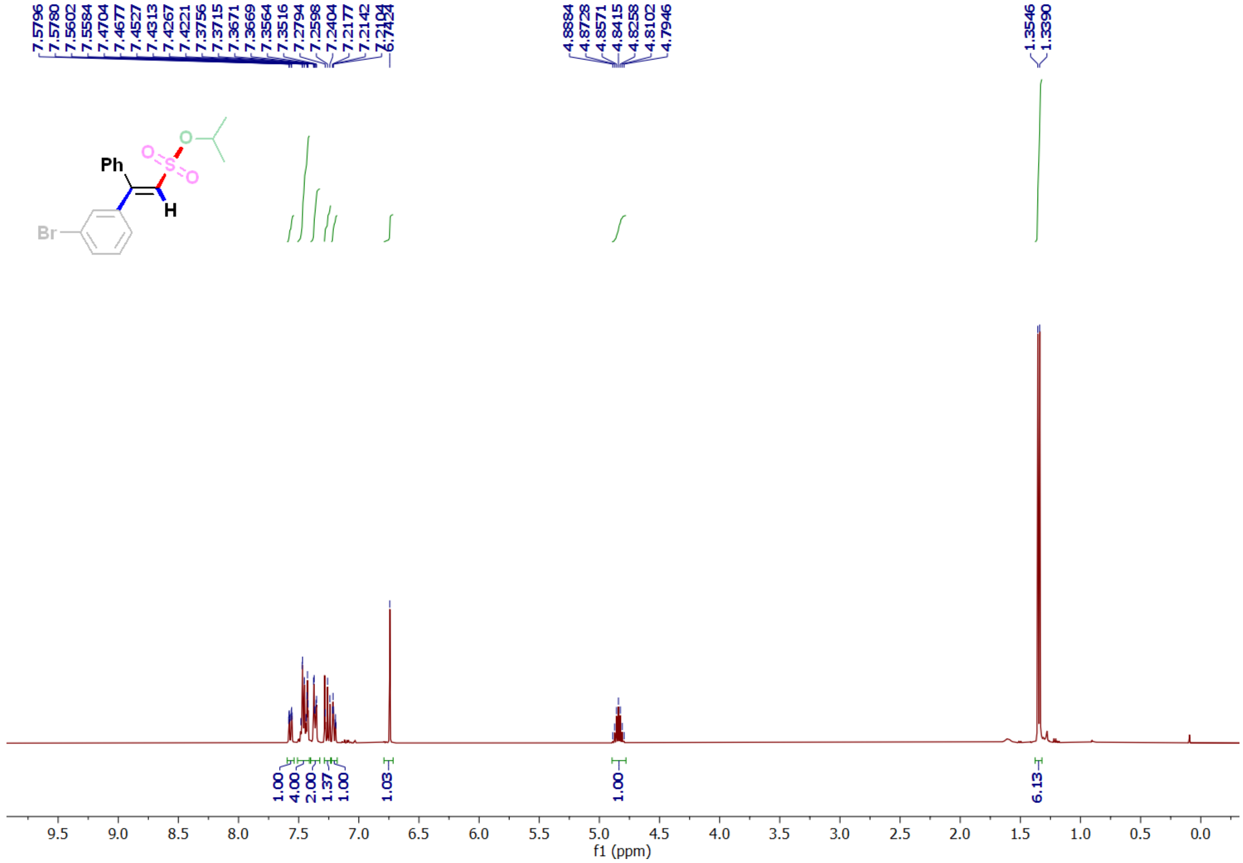

^{13}C NMR (101MHz, $CDCl_3$) of **5s**

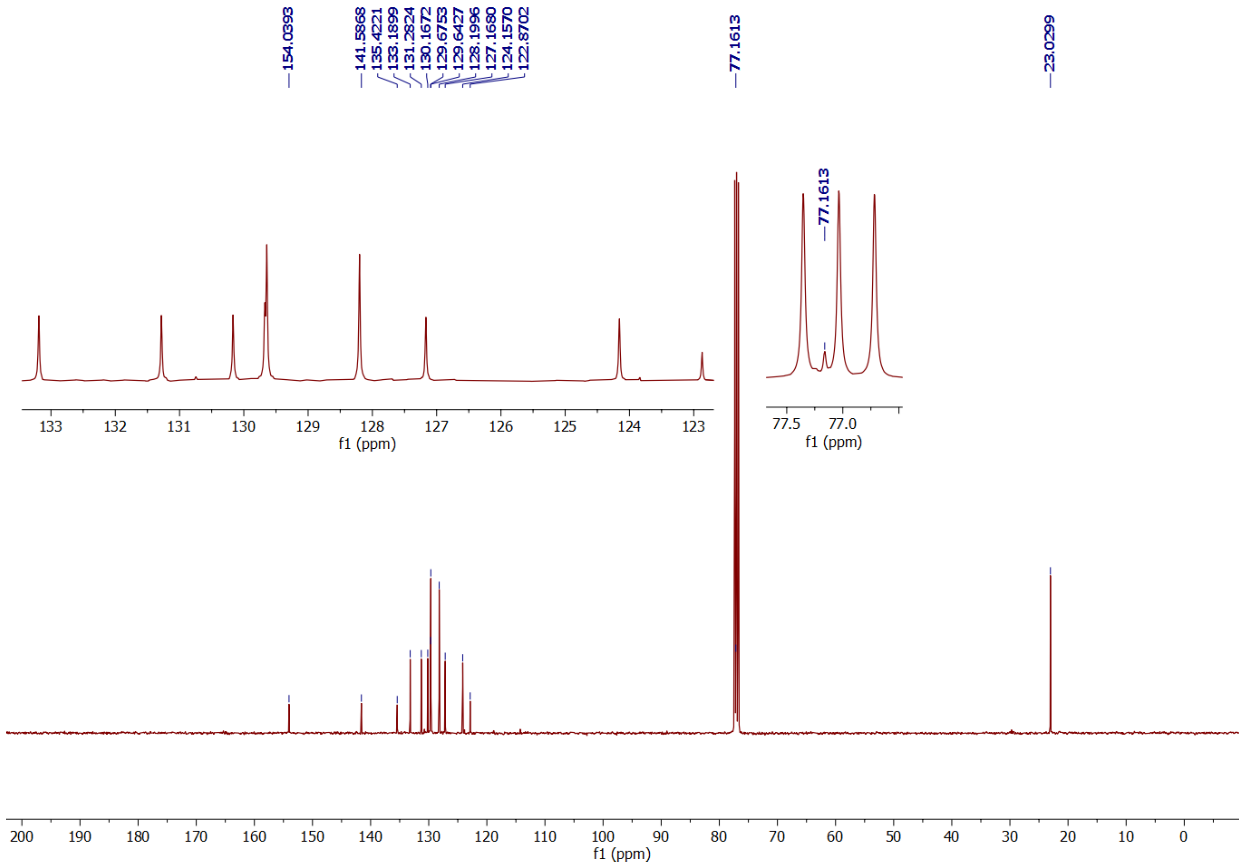

NOE Spectrum for **5s**

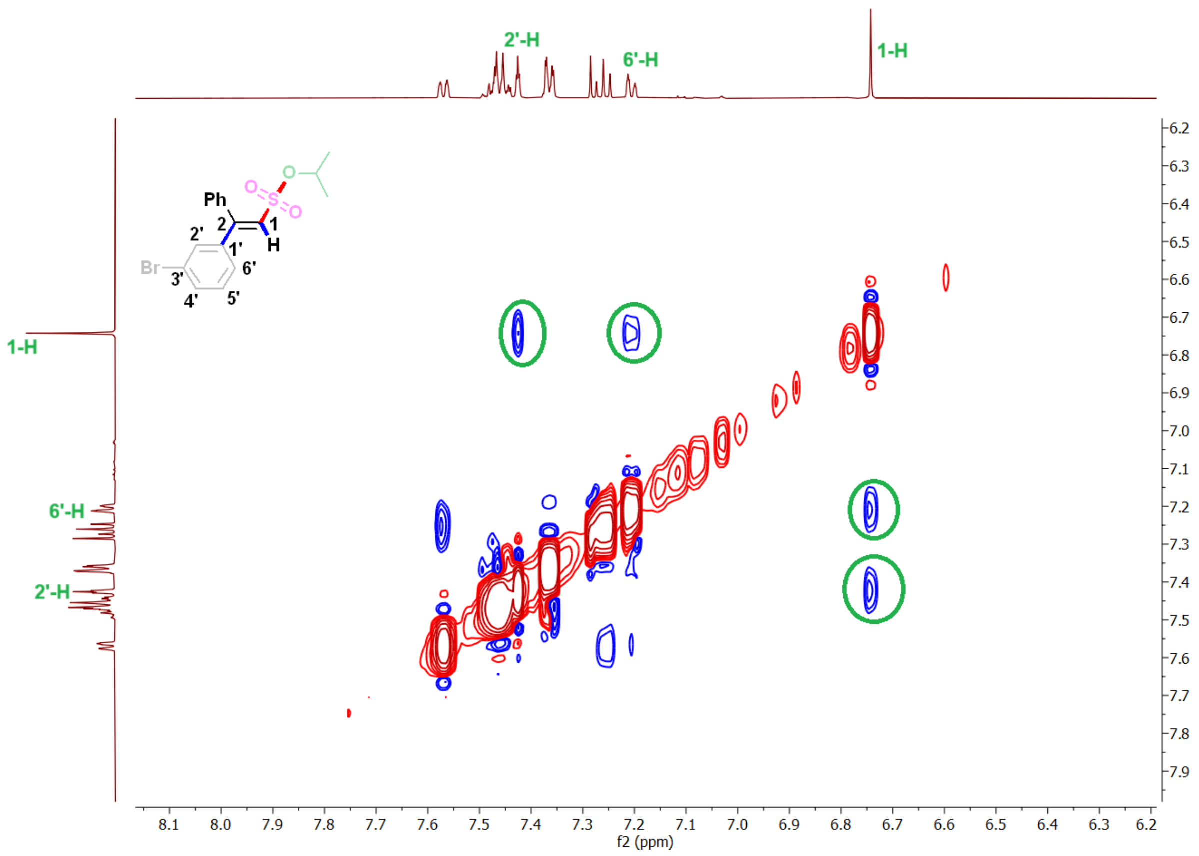

^{1}H NMR (400 MHz, $CDCl_3$) of **5t** (see procedure)

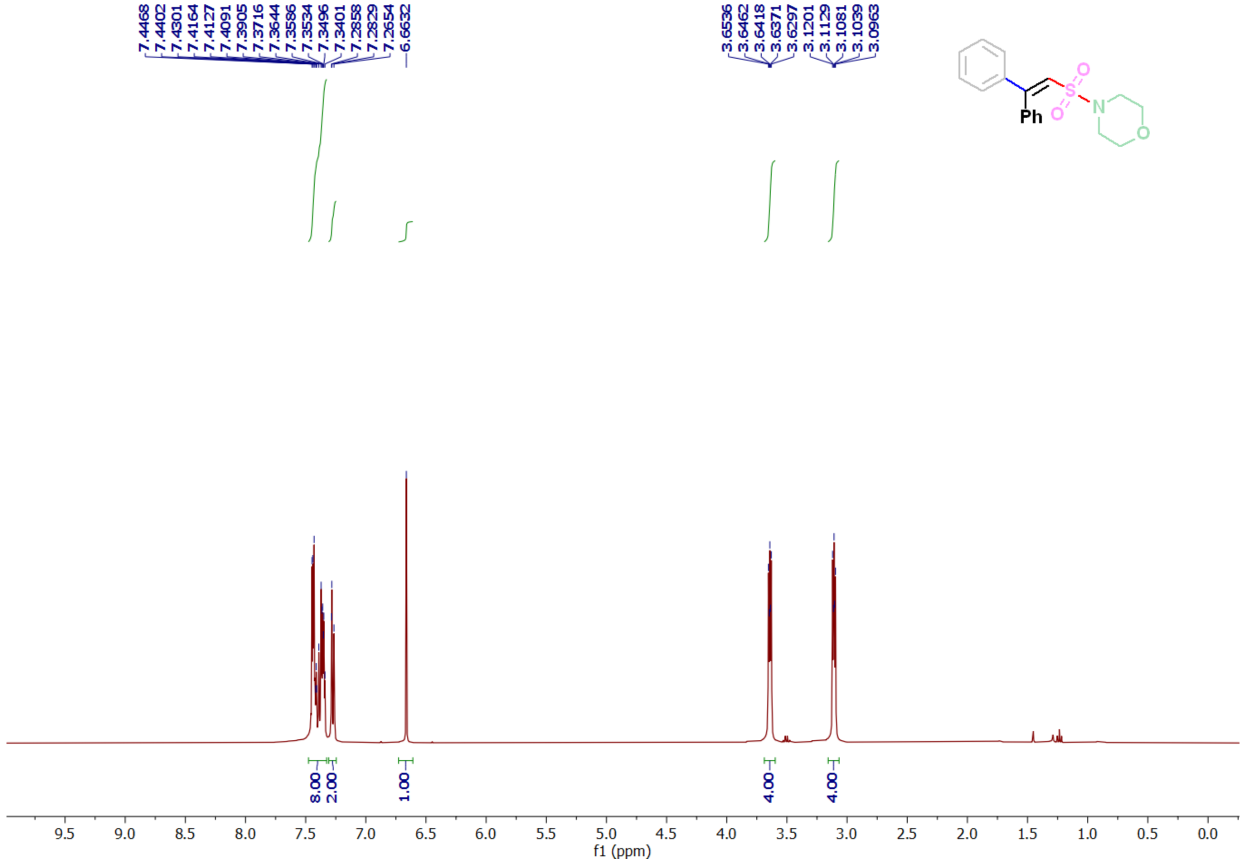

^{13}C NMR (101MHz, $CDCl_3$) of **5t**

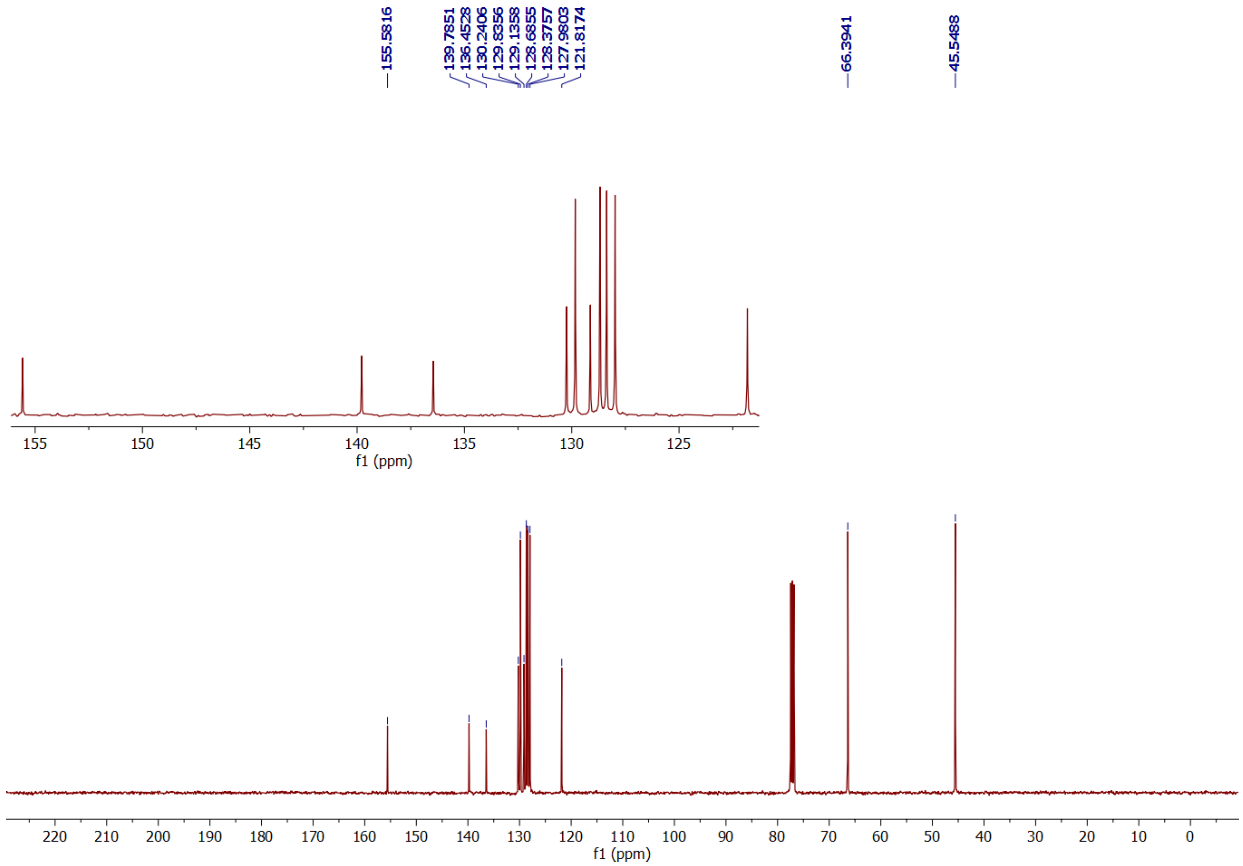

^{1}H NMR (400 MHz, $CDCl_3$) of **6a** (see procedure)

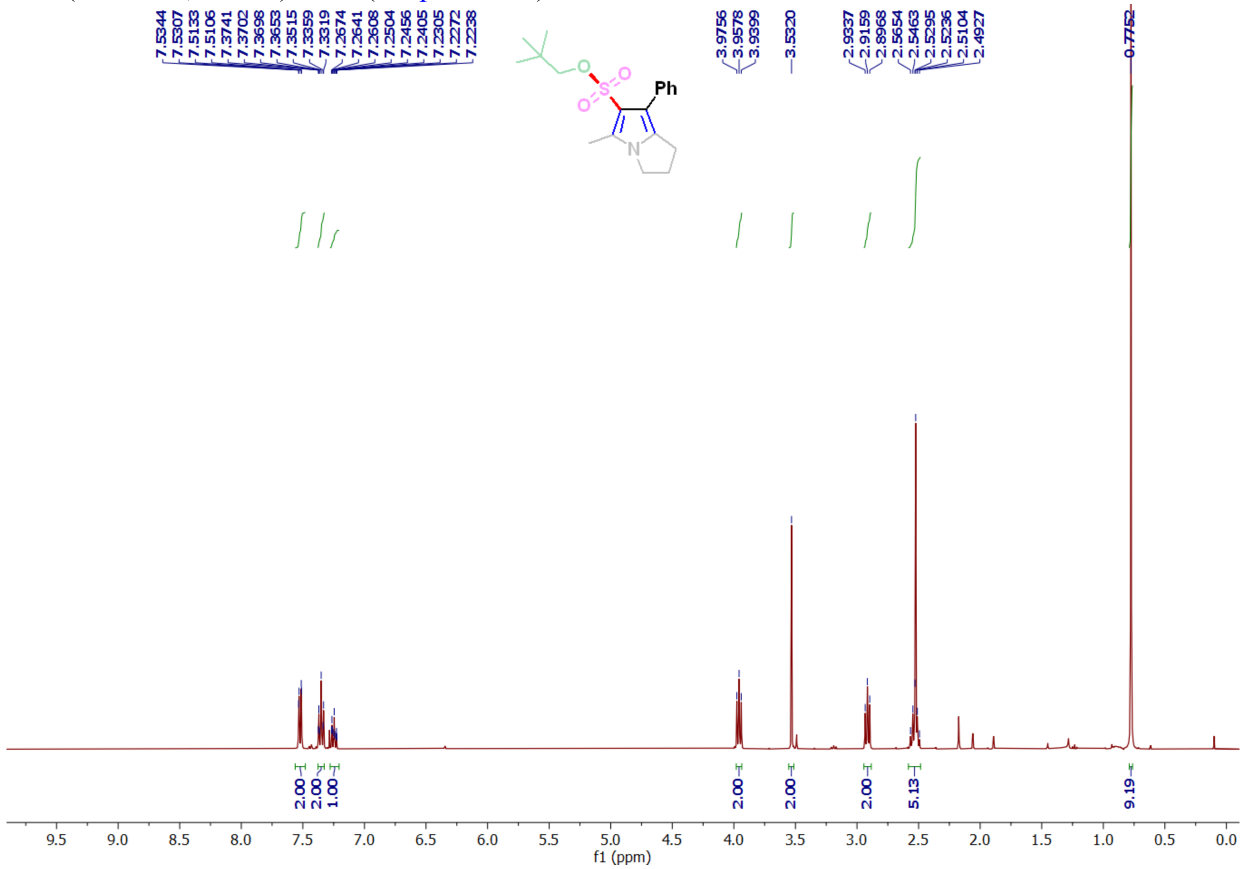

^{13}C NMR (101MHz, $CDCl_3$) of **6a**

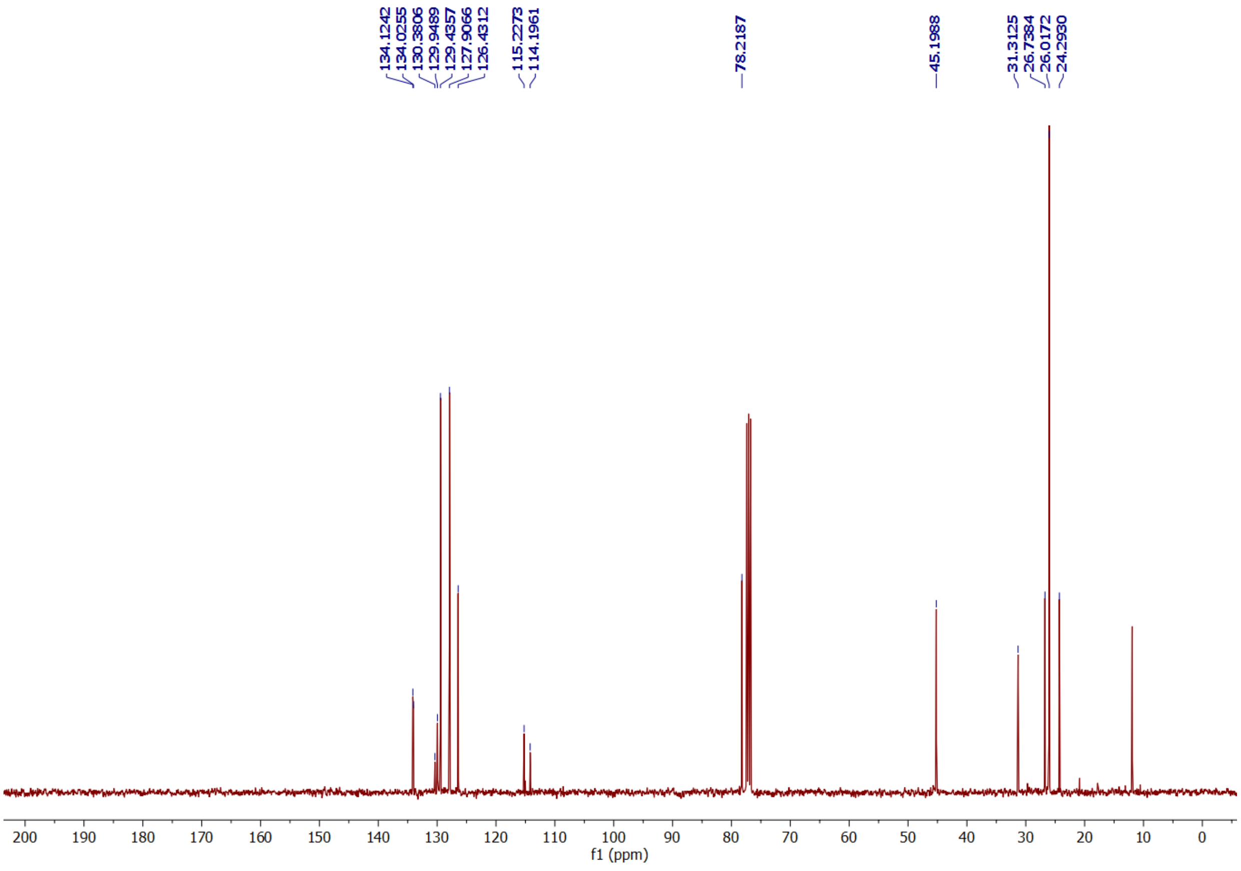

^{1}H NMR (300 MHz, $CDCl_3$) of **6b** (see procedure)

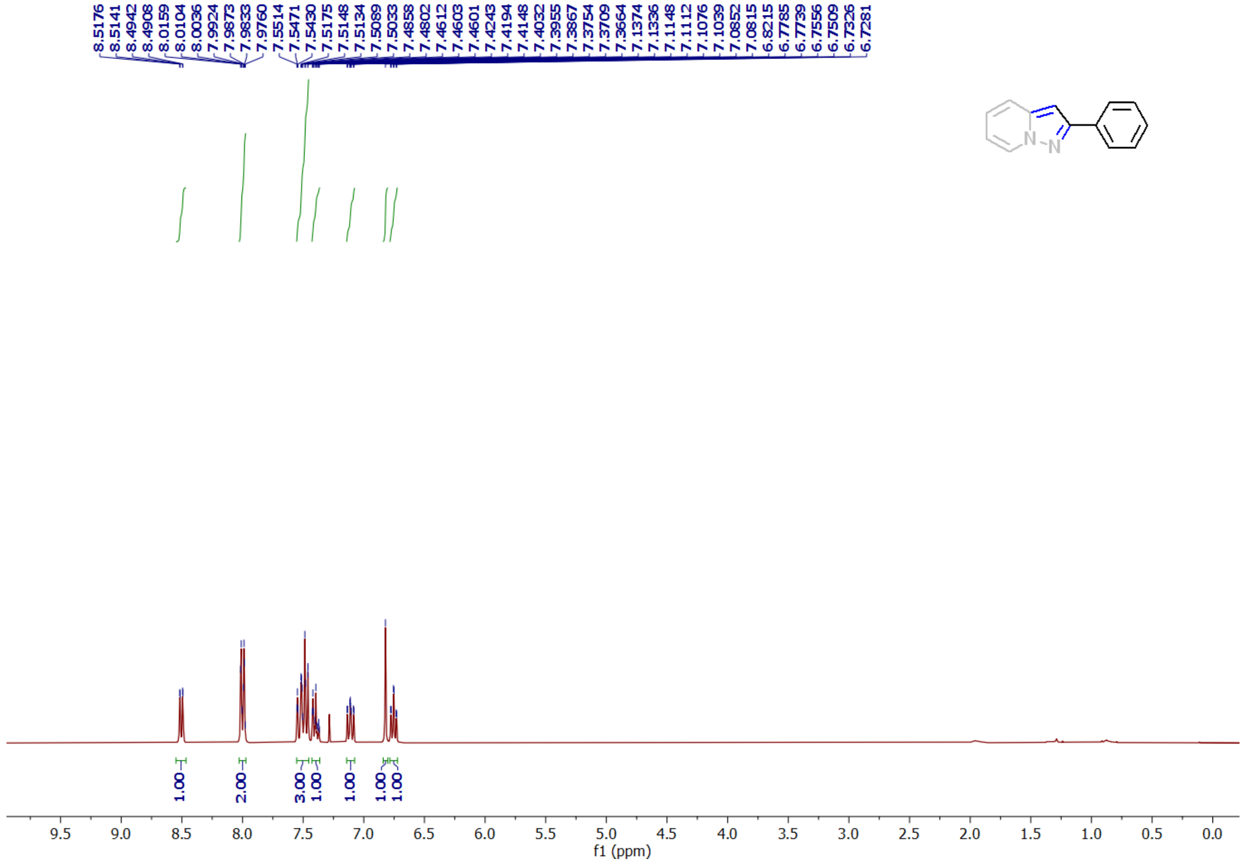

^{13}C NMR (101MHz, $CDCl_3$) of **6b**

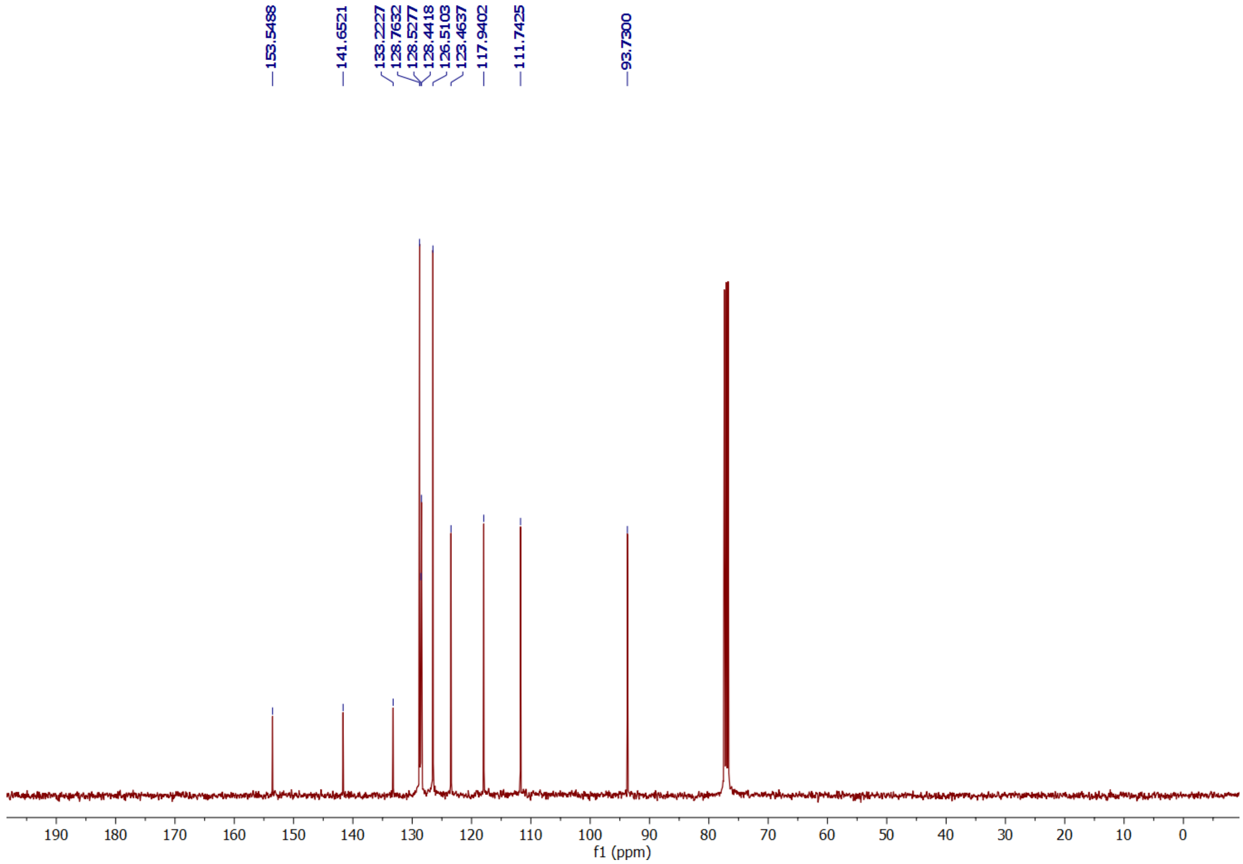

^{1}H NMR (300 MHz, $CDCl_3$) of **6c** (see procedure)

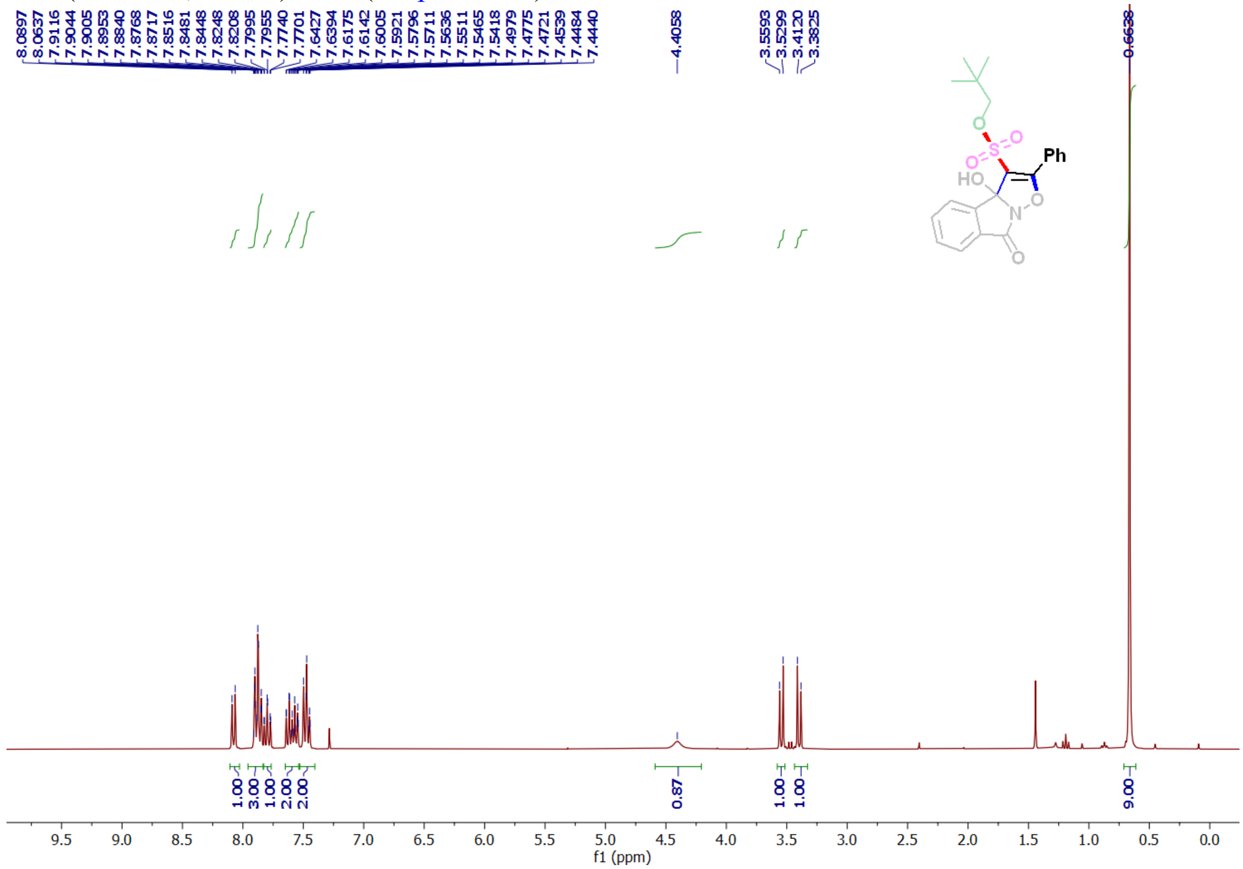

^{13}C NMR (75 MHz, $CDCl_3$) of **6c**

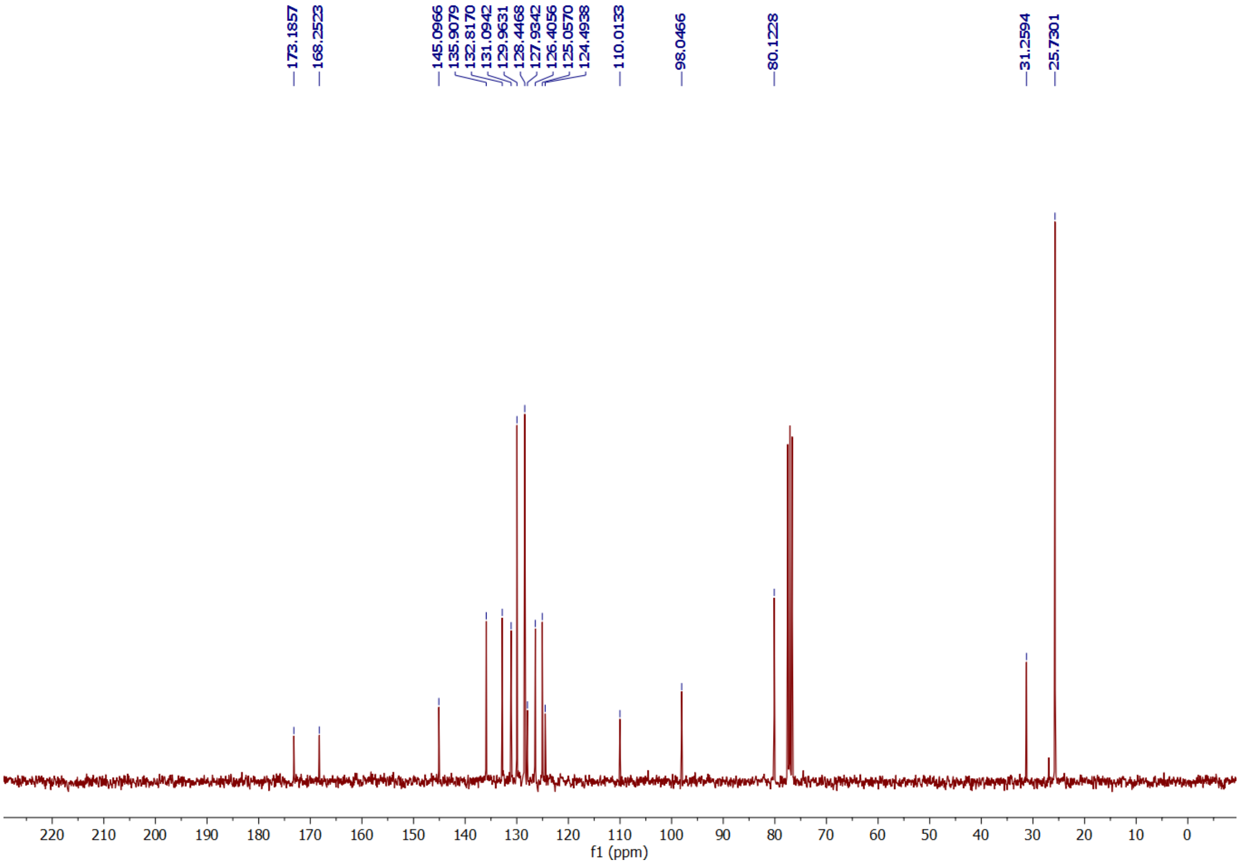

^{1}H NMR (300 MHz, $CDCl_3$) of **6d** (see procedure)

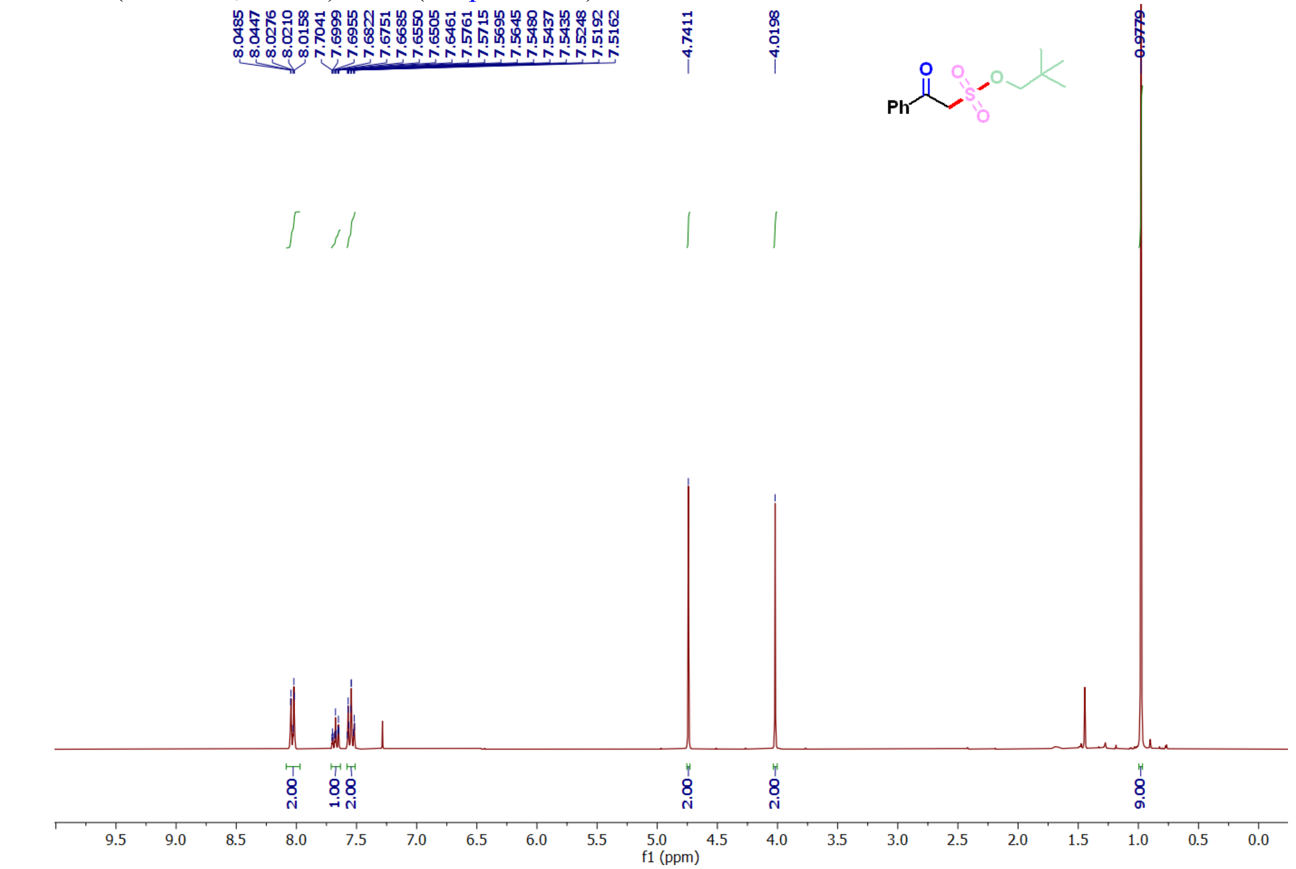

^{13}C NMR (75 MHz, $CDCl_3$) of **6d**

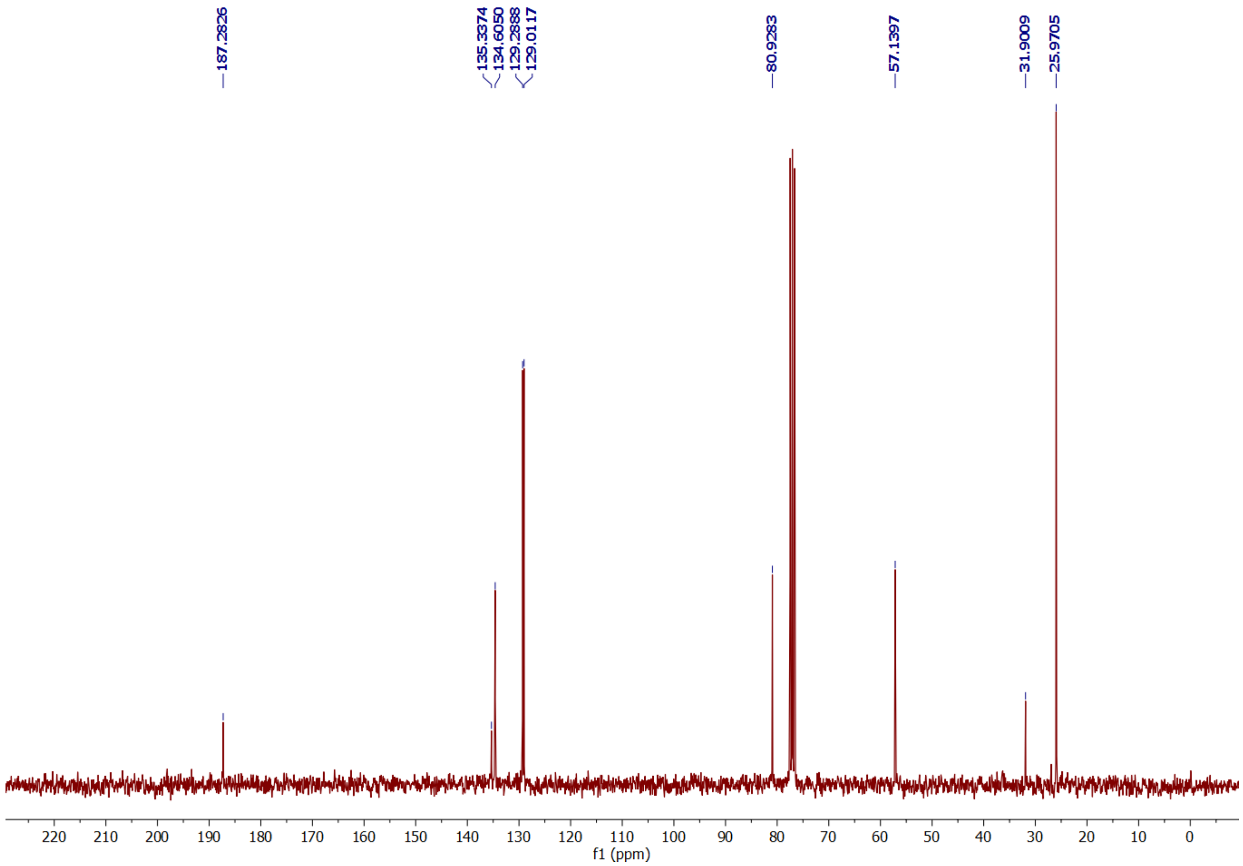

^{1}H NMR (300 MHz, $CDCl_3$) of **6e** (see procedure)

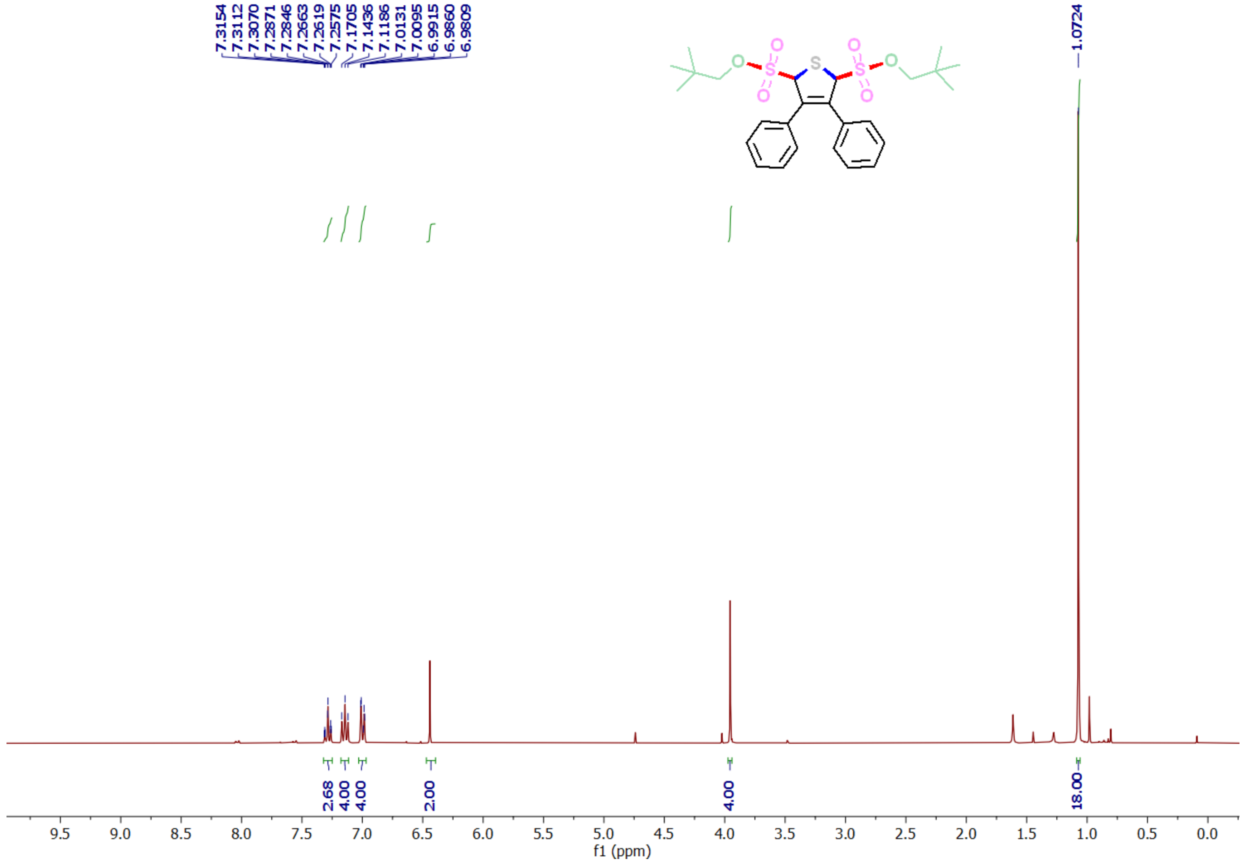

^{13}C NMR (75 MHz, $CDCl_3$) of **6e**

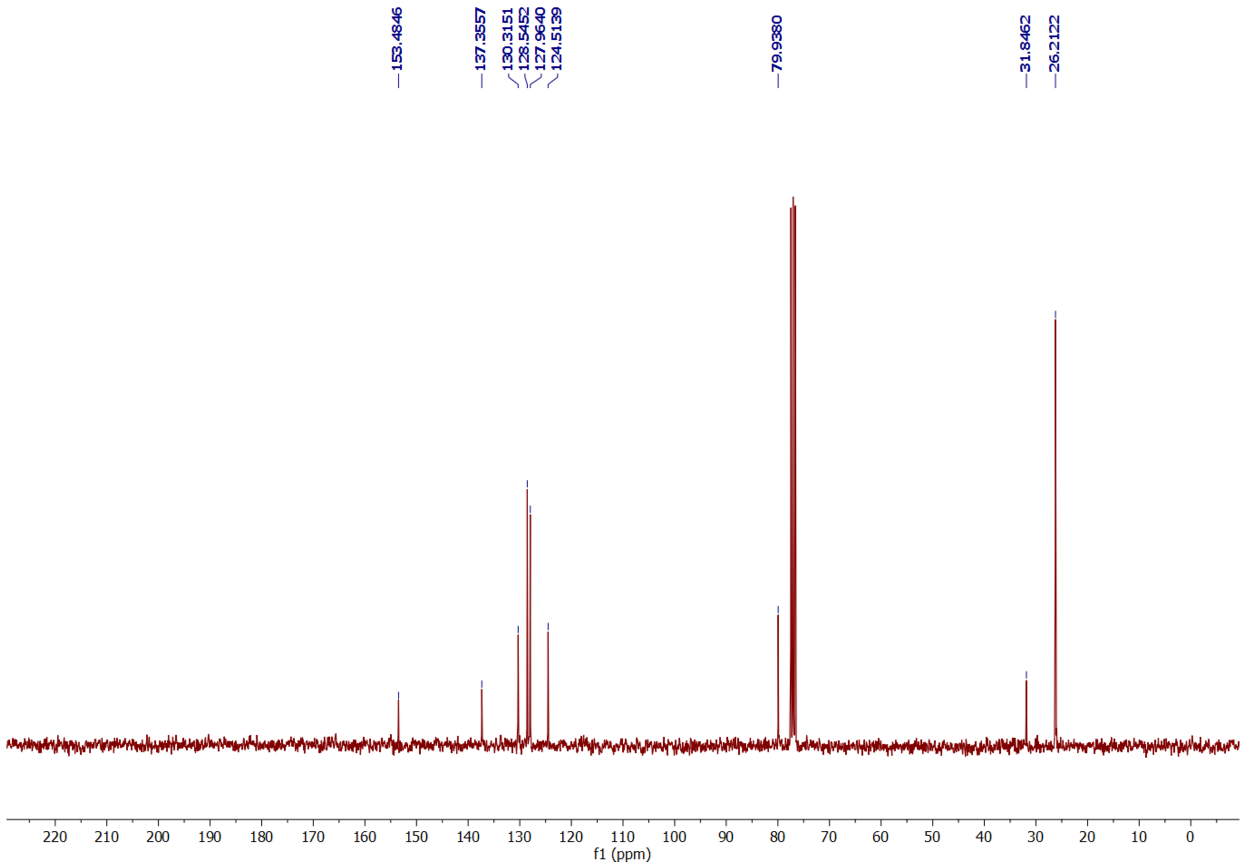

^{1}H NMR (300 MHz, $CDCl_3$) of **6f** (see procedure)

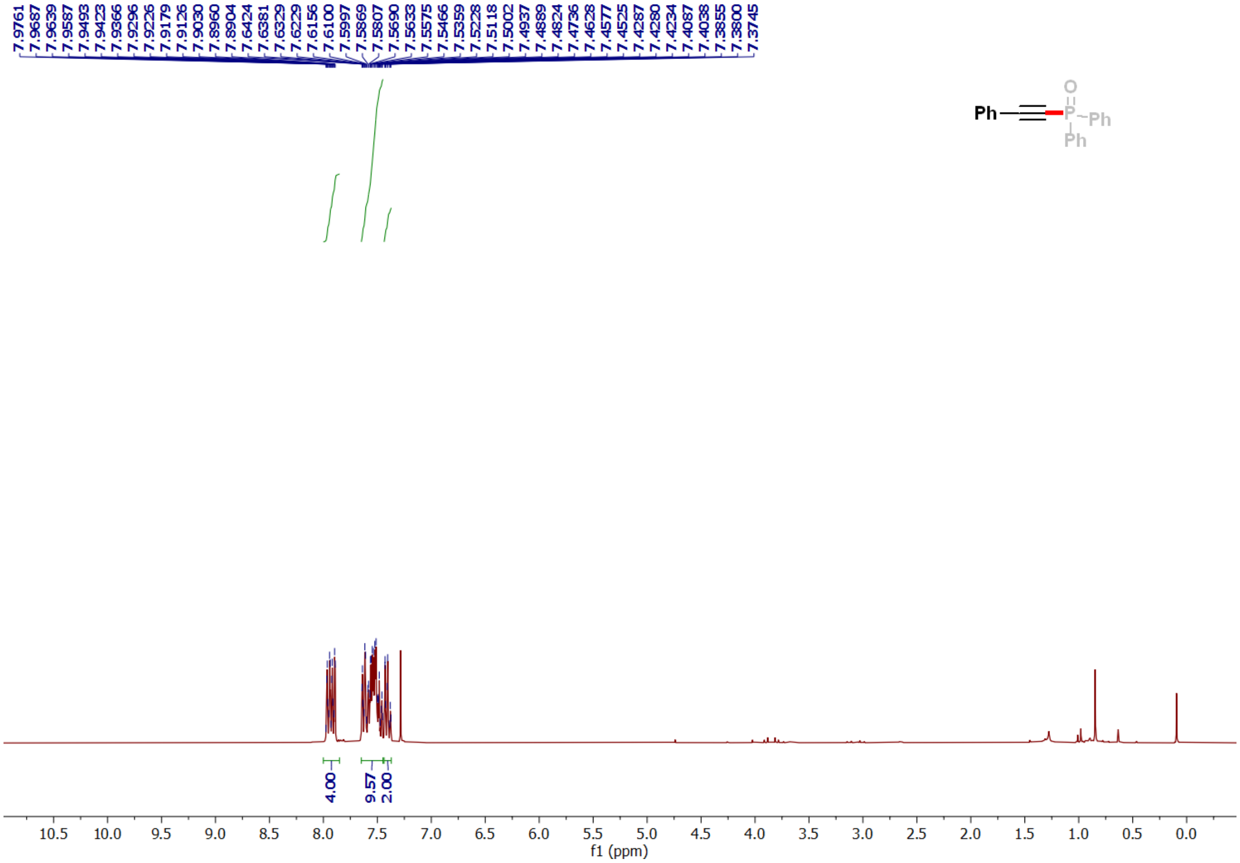

^{13}C NMR (75 MHz, $CDCl_3$) of **6f**

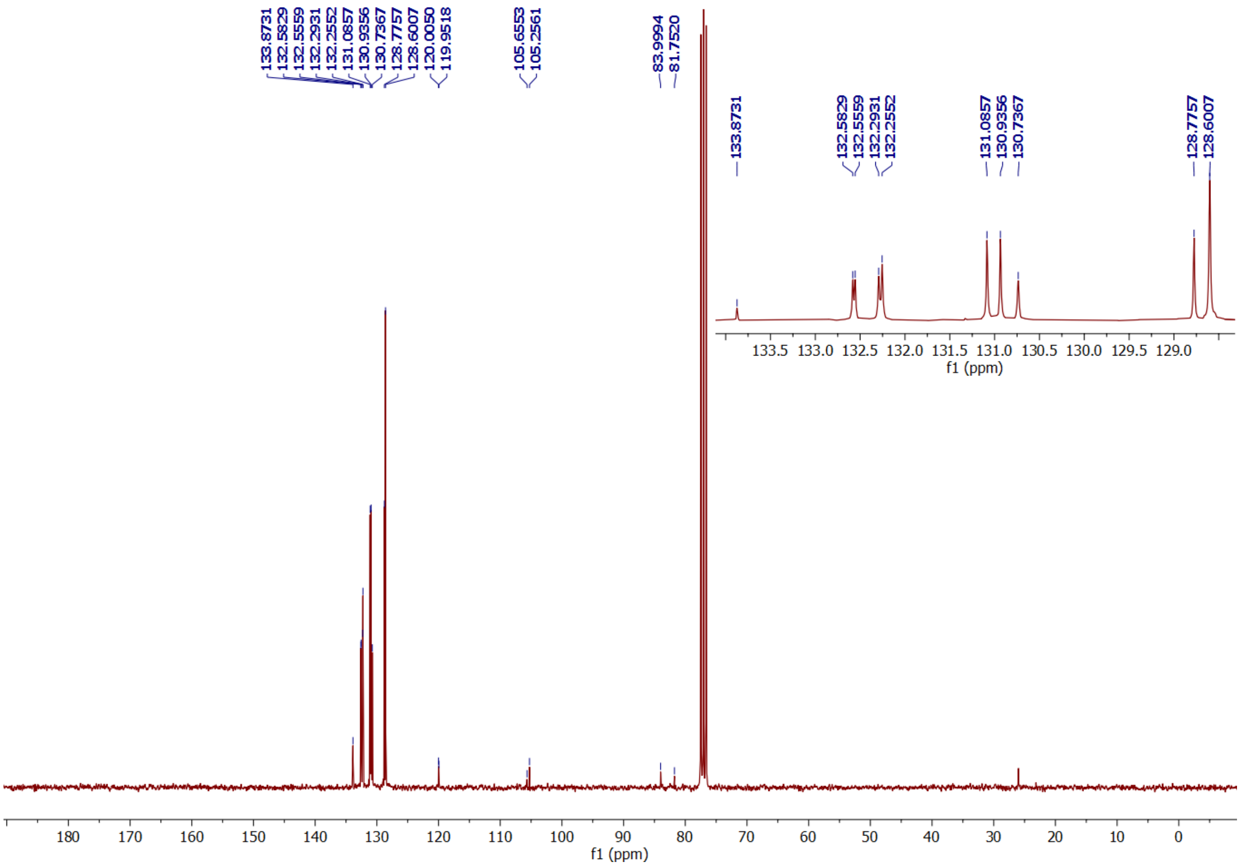

^{1}H NMR (400 MHz, $CDCl_3$) of **6g** (see procedure)

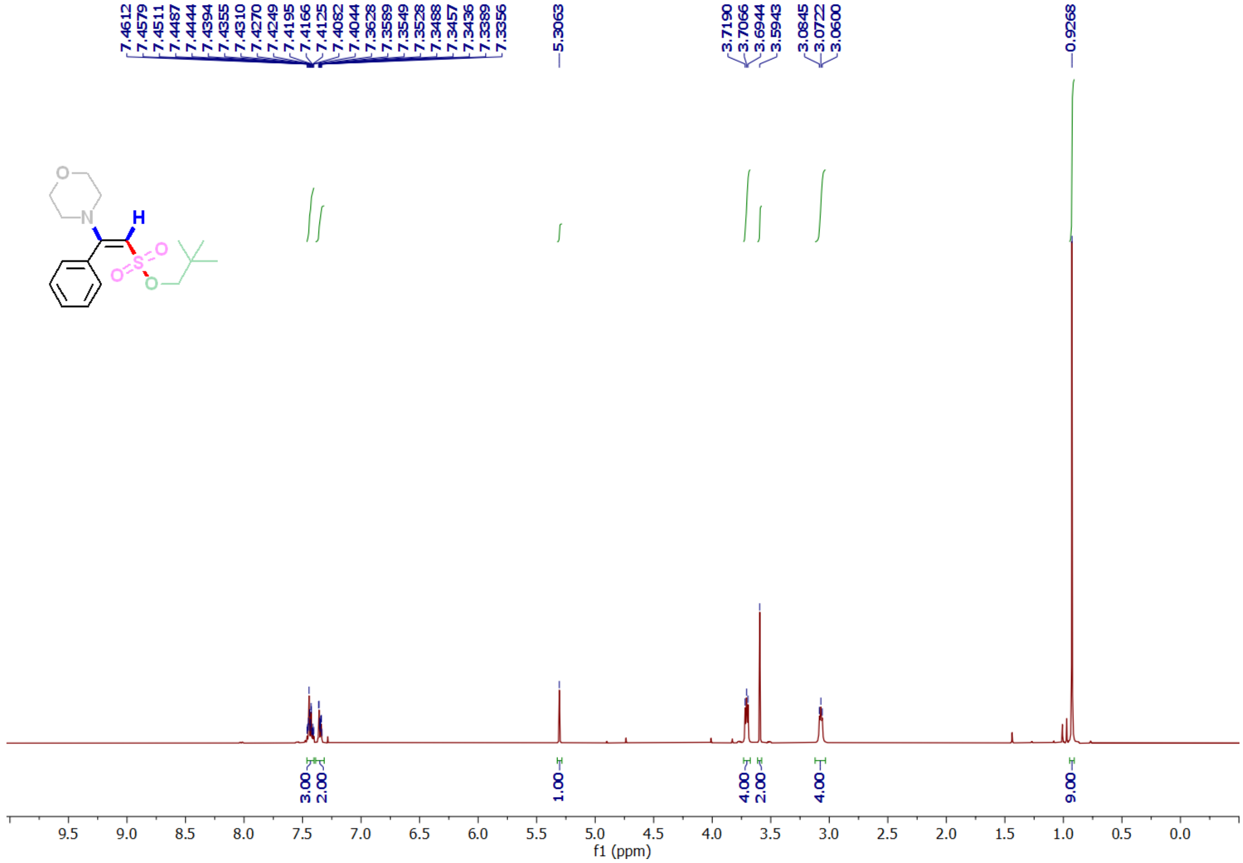

^{13}C NMR (101MHz, $CDCl_3$) of **6g**

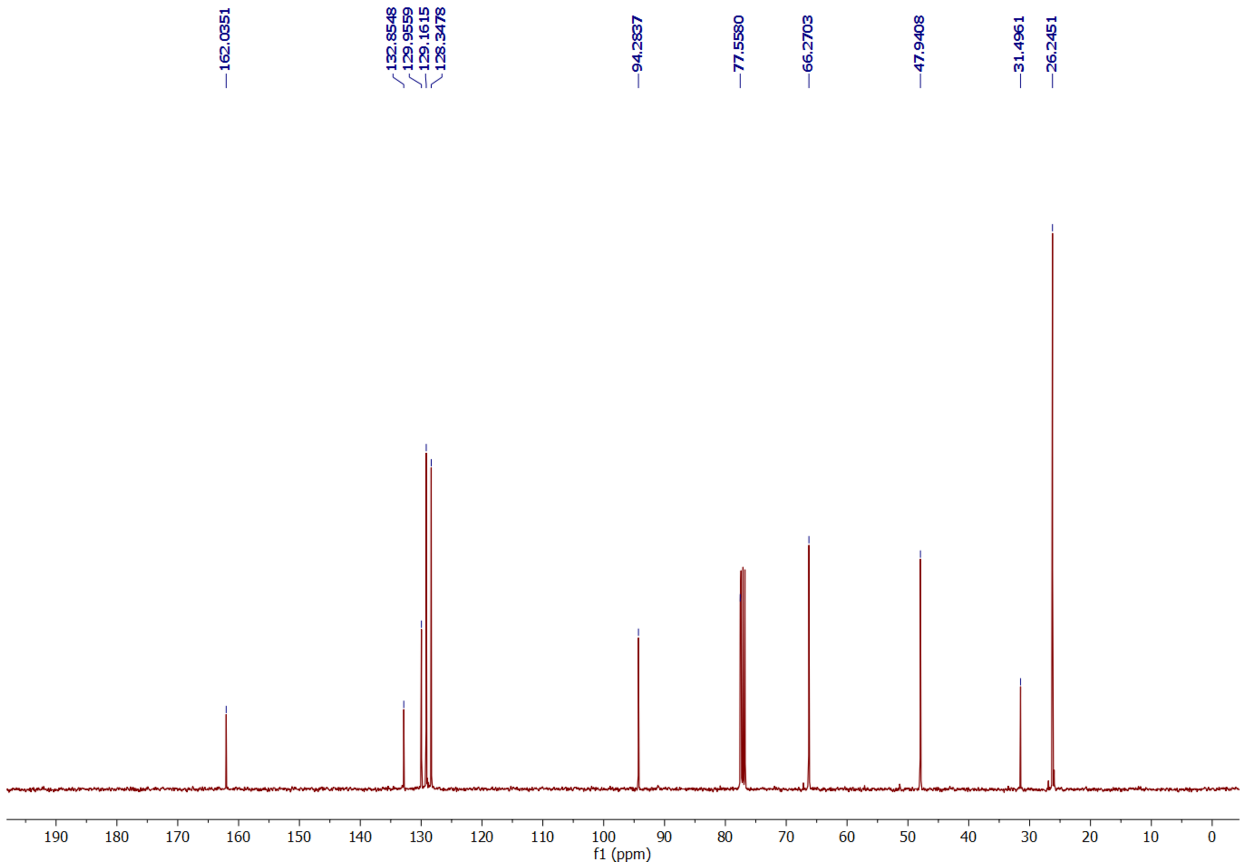

NOE Spectrum for **6g**

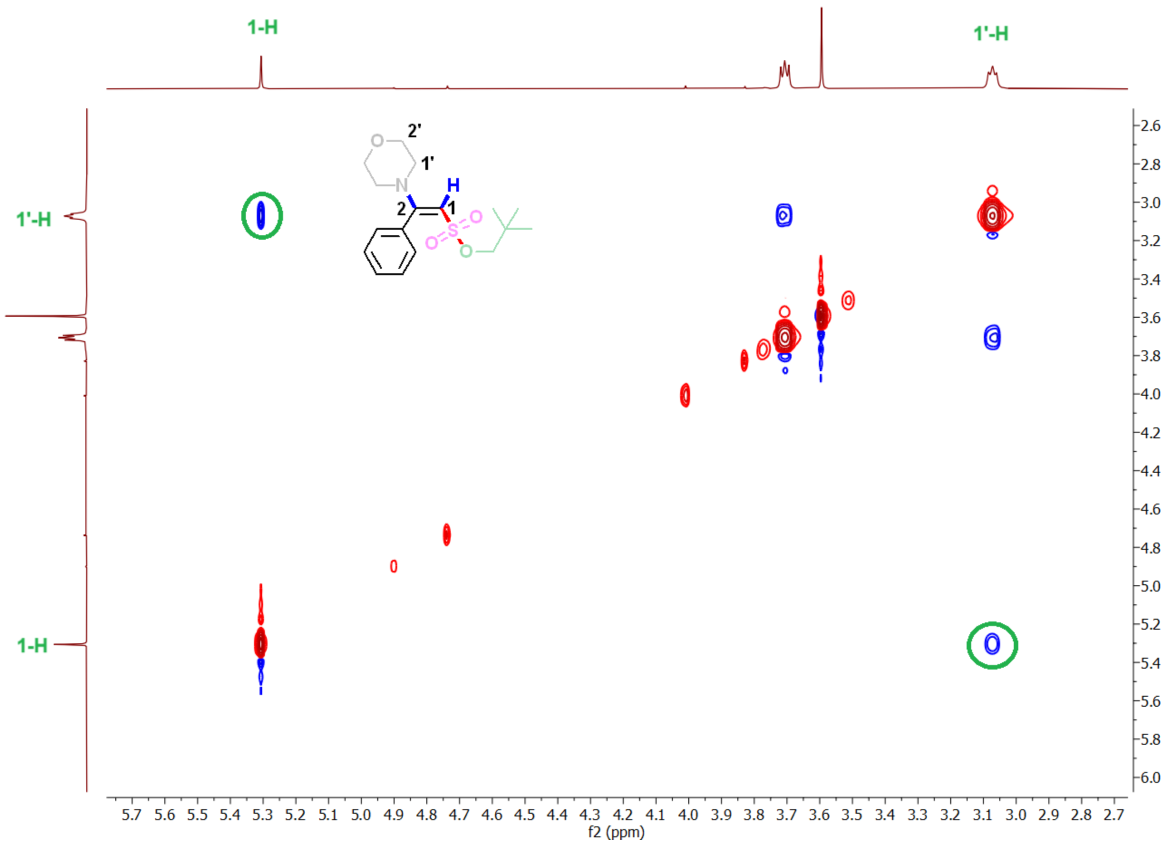

^{1}H NMR (300 MHz, $CDCl_3$) of **6h** (see procedure)

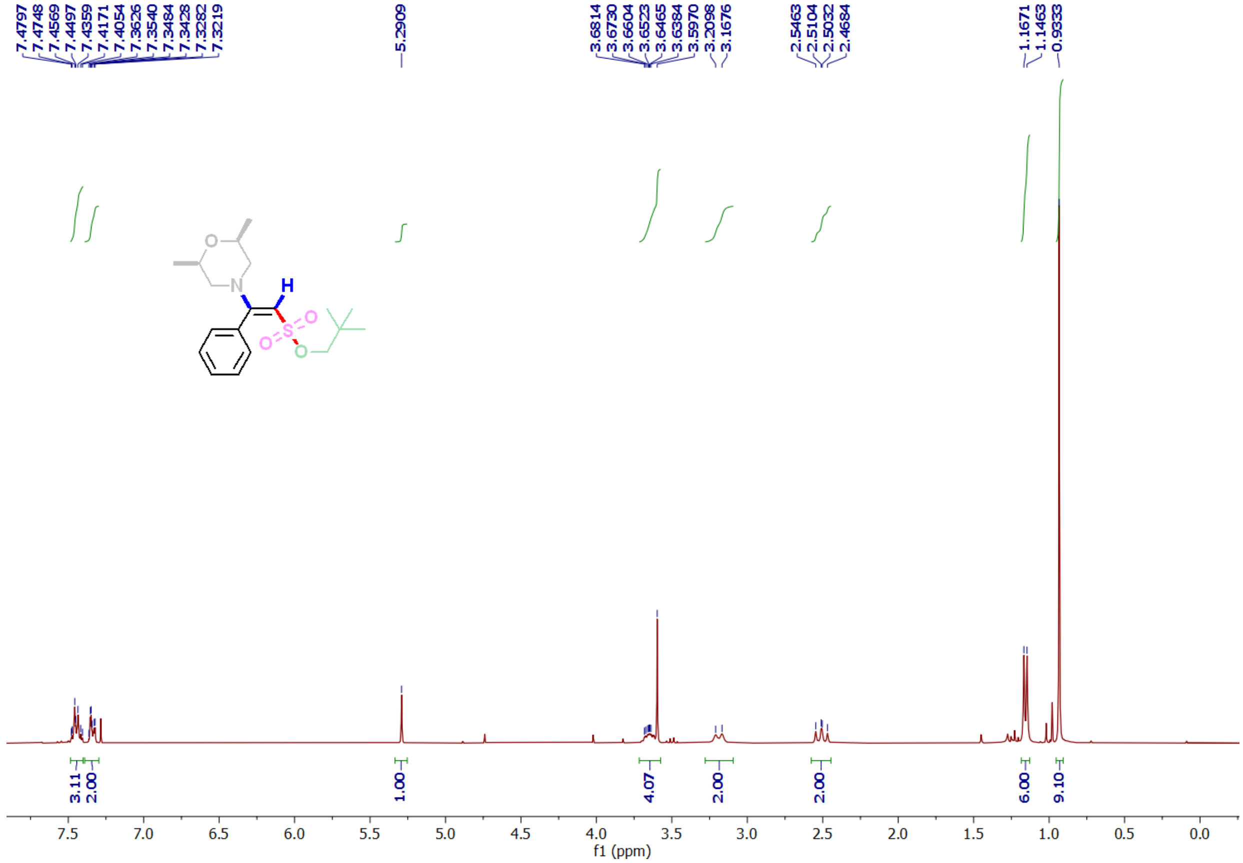

^{13}C NMR (75 MHz, $CDCl_3$) of **6h**

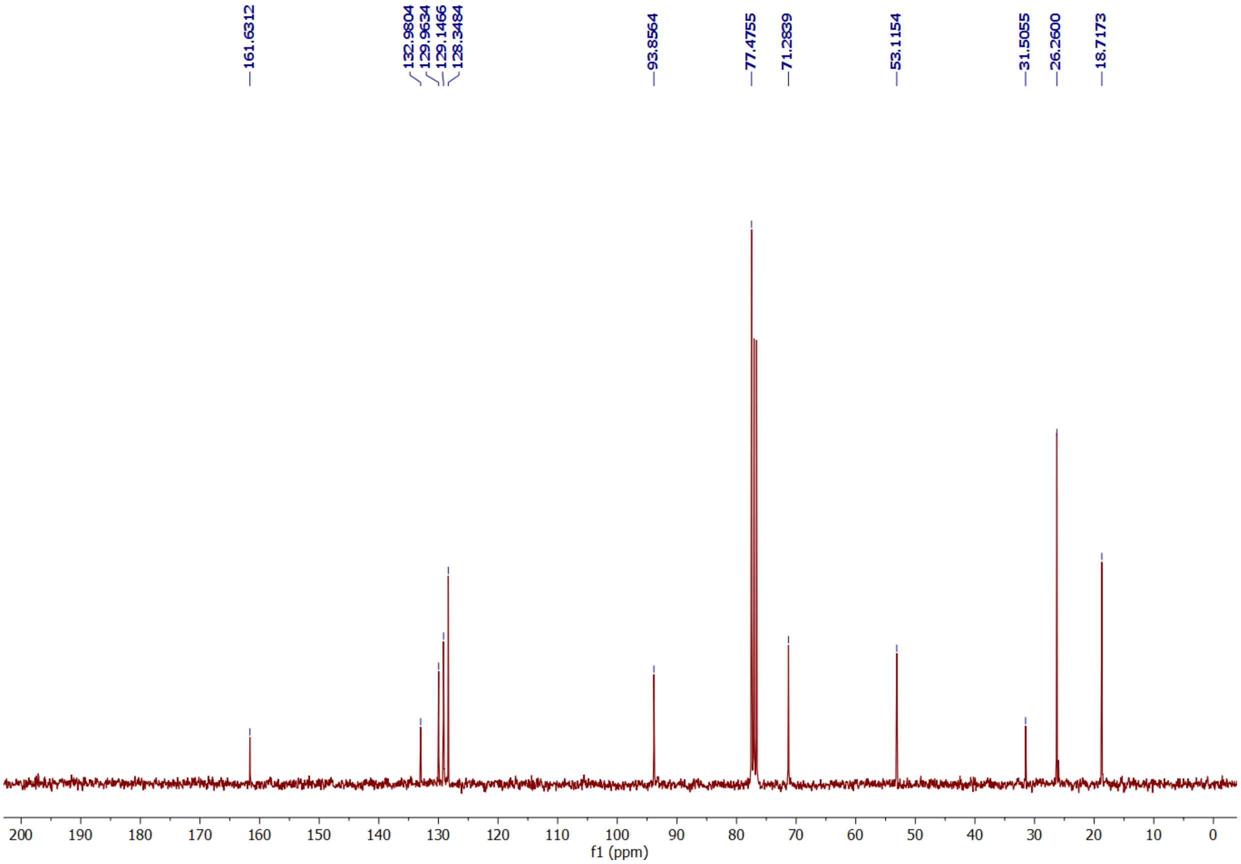

NOE Spectrum for **6h**

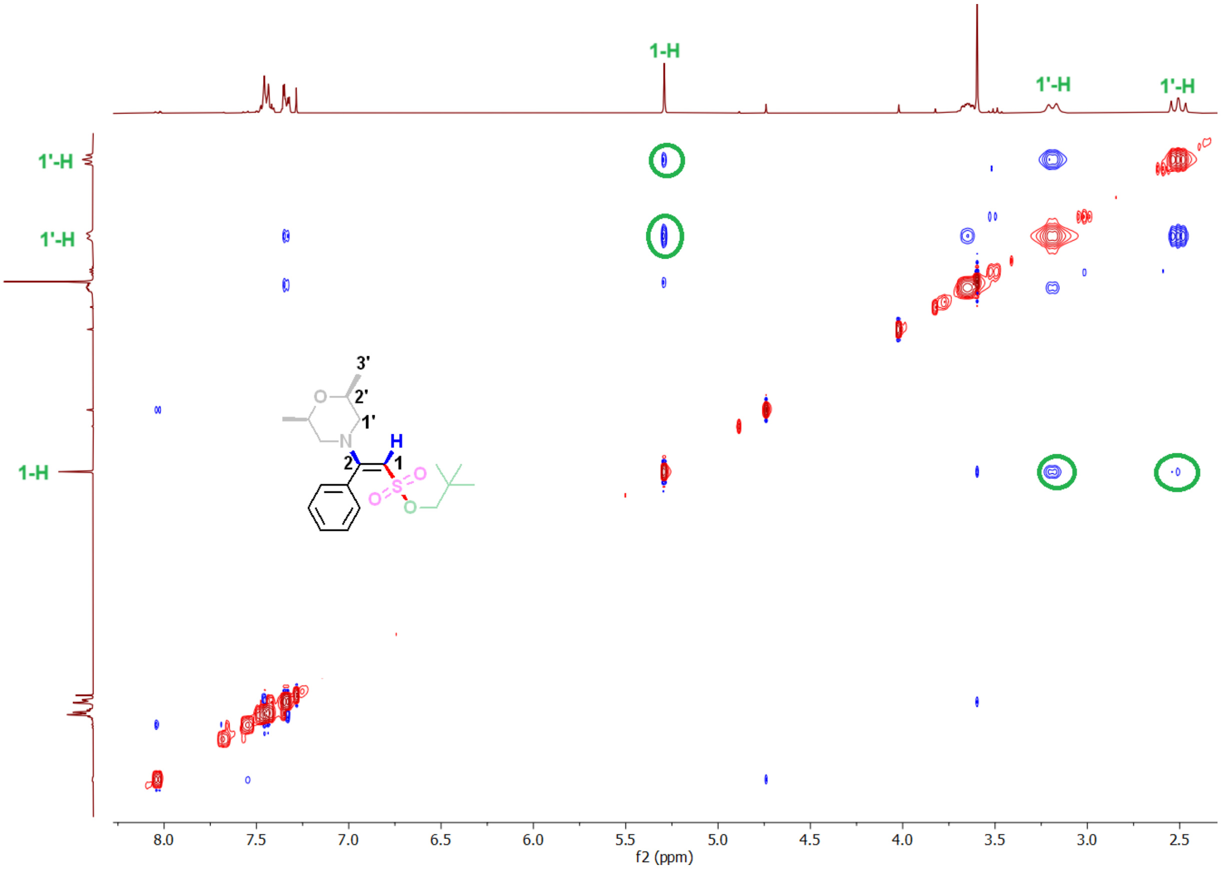

^{1}H NMR (300 MHz, $CDCl_3$) of **6i** (see procedure)

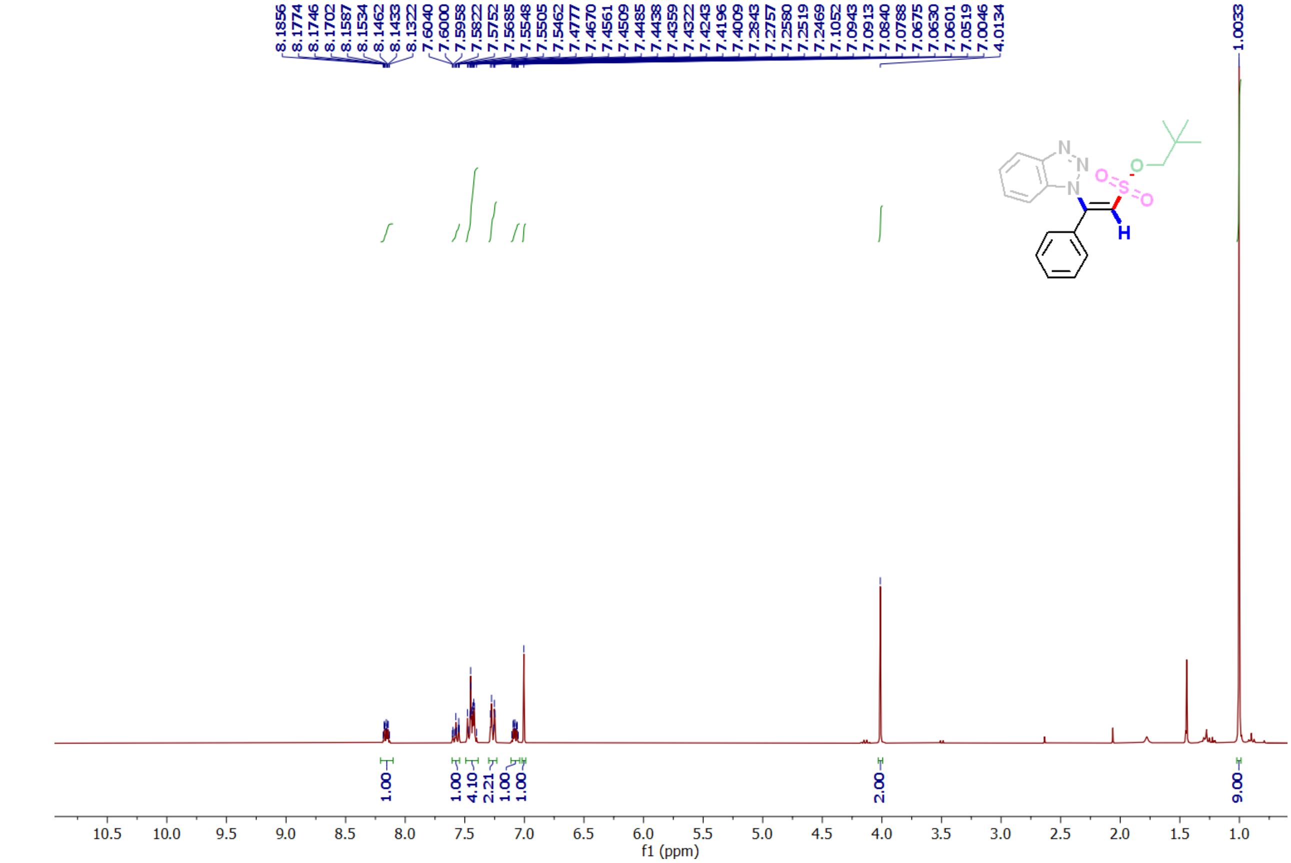

^{13}C NMR (75 MHz, $CDCl_3$) of **6i**

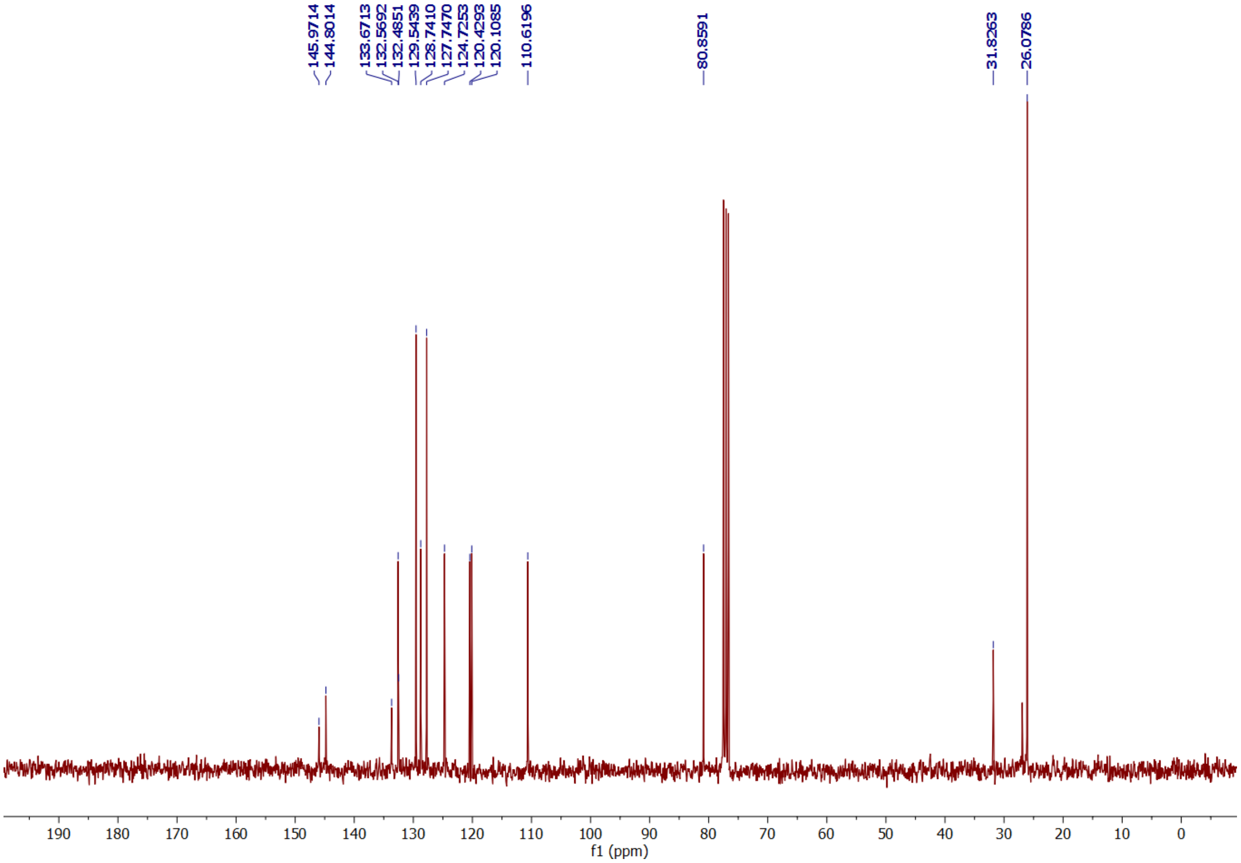

NOE Spectrum for **6i**

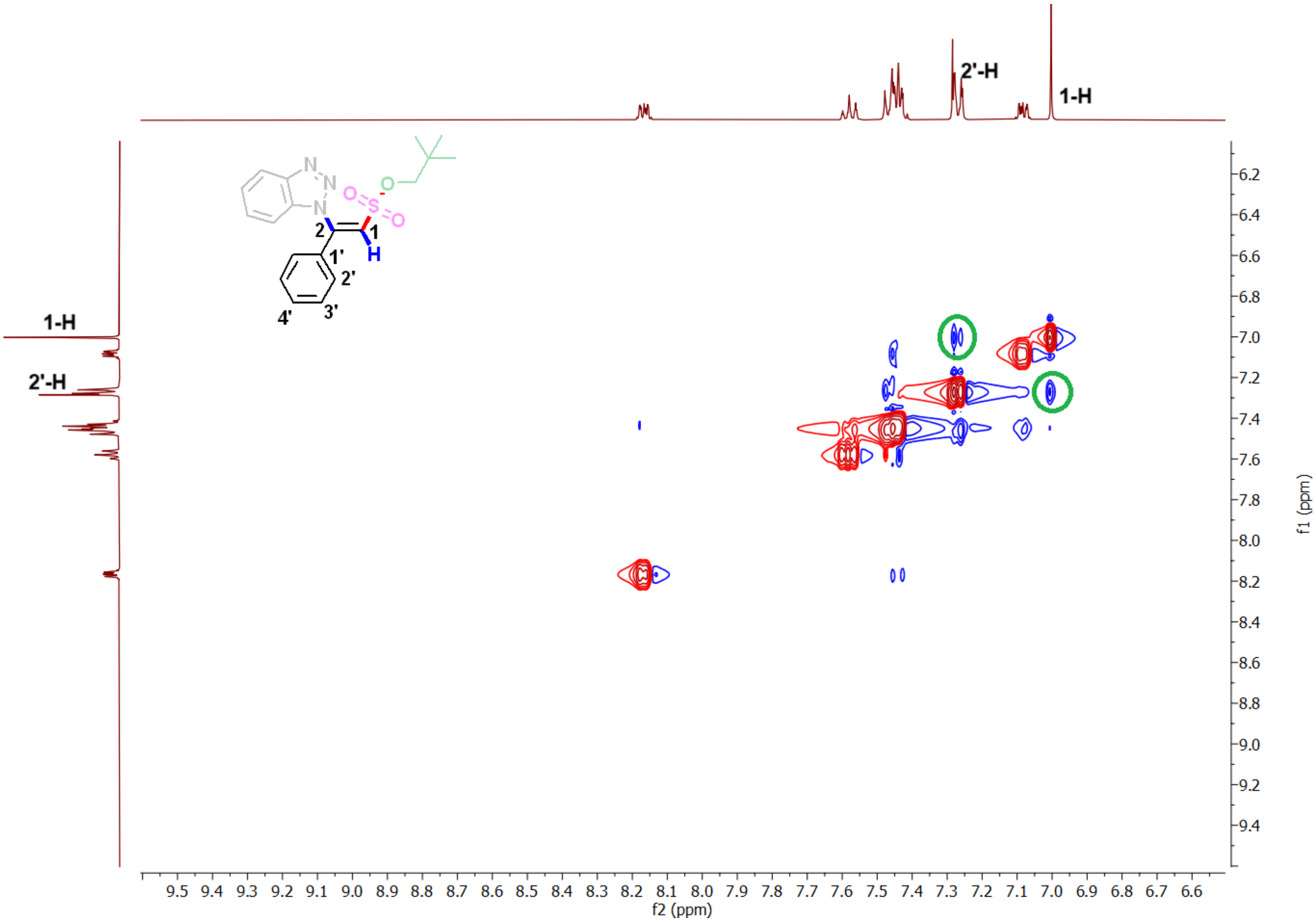

^{1}H NMR (400 MHz, $CDCl_3$) of **6j** (see procedure)

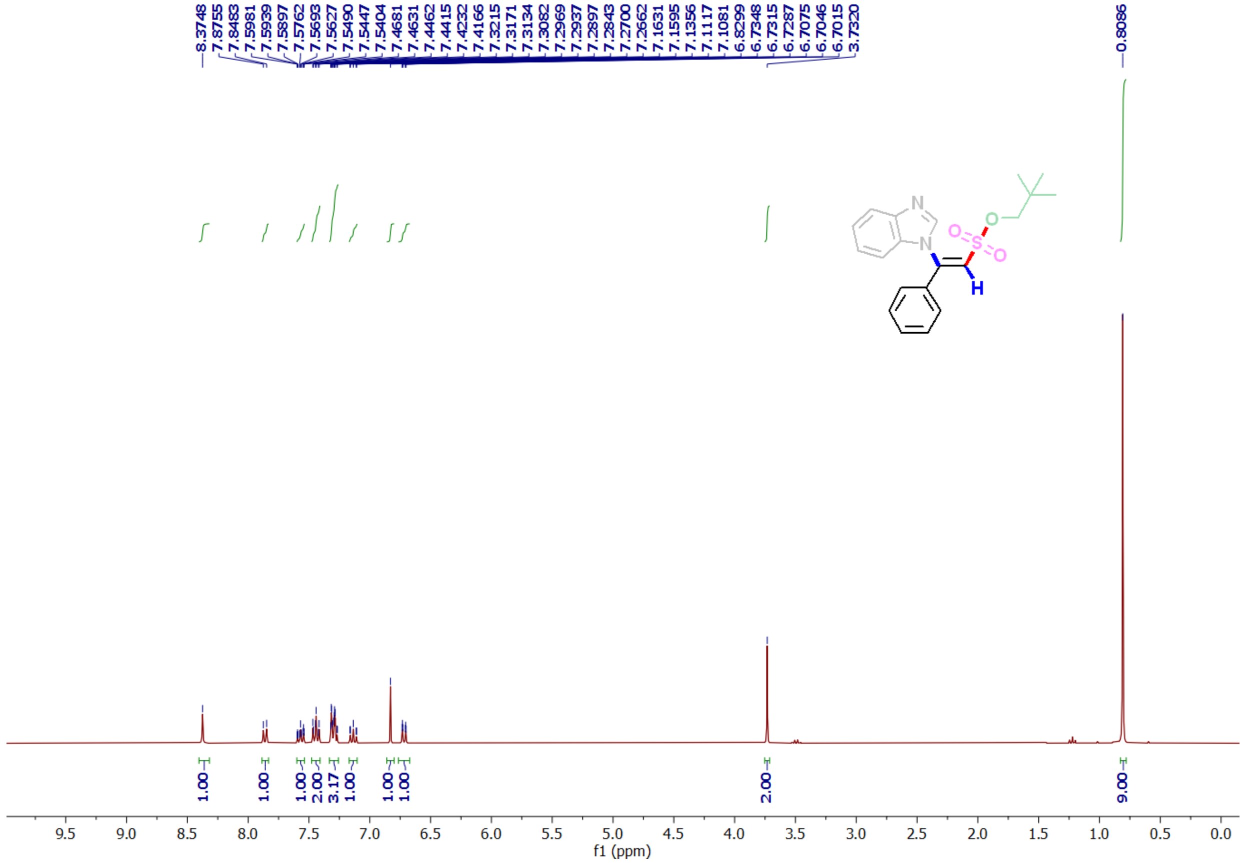

^{13}C NMR (101MHz, $CDCl_3$) of **6j**

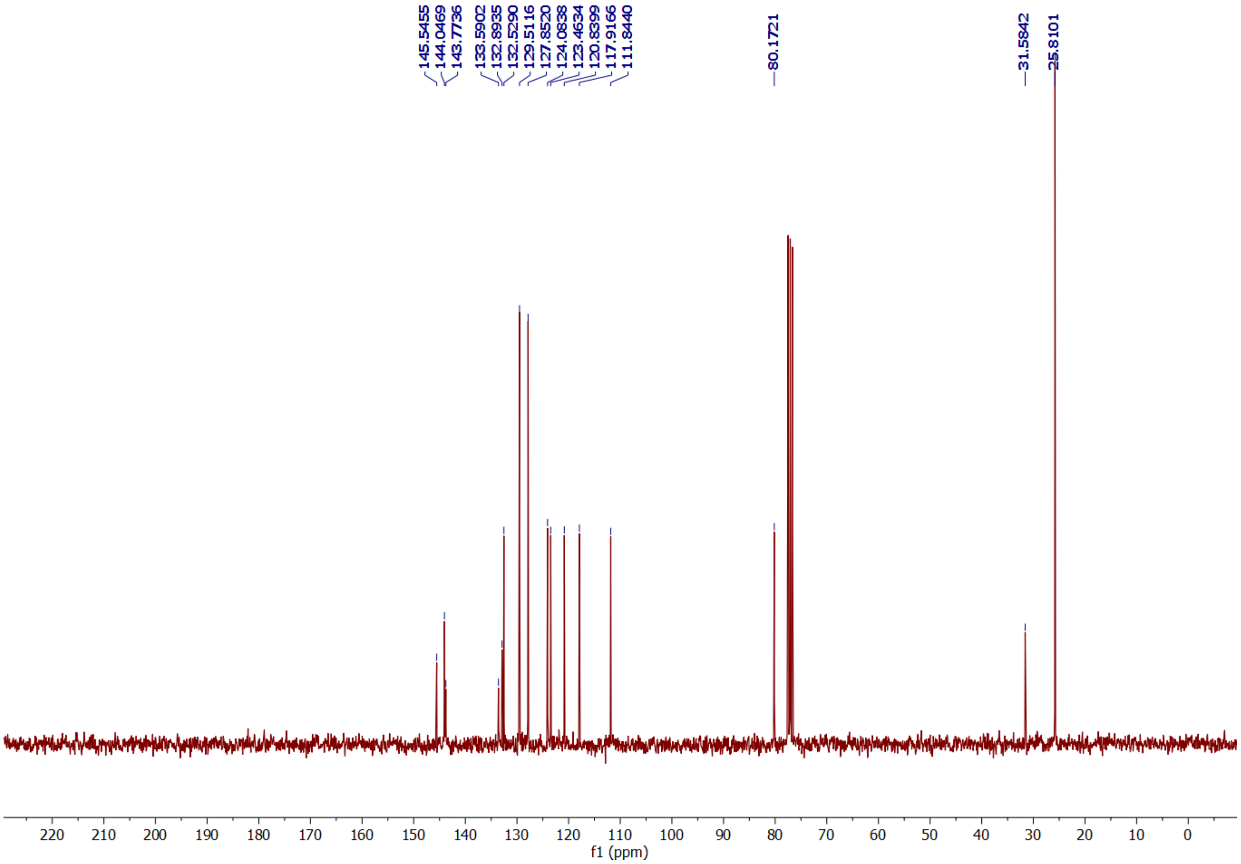

NOE Spectrum for **6j**

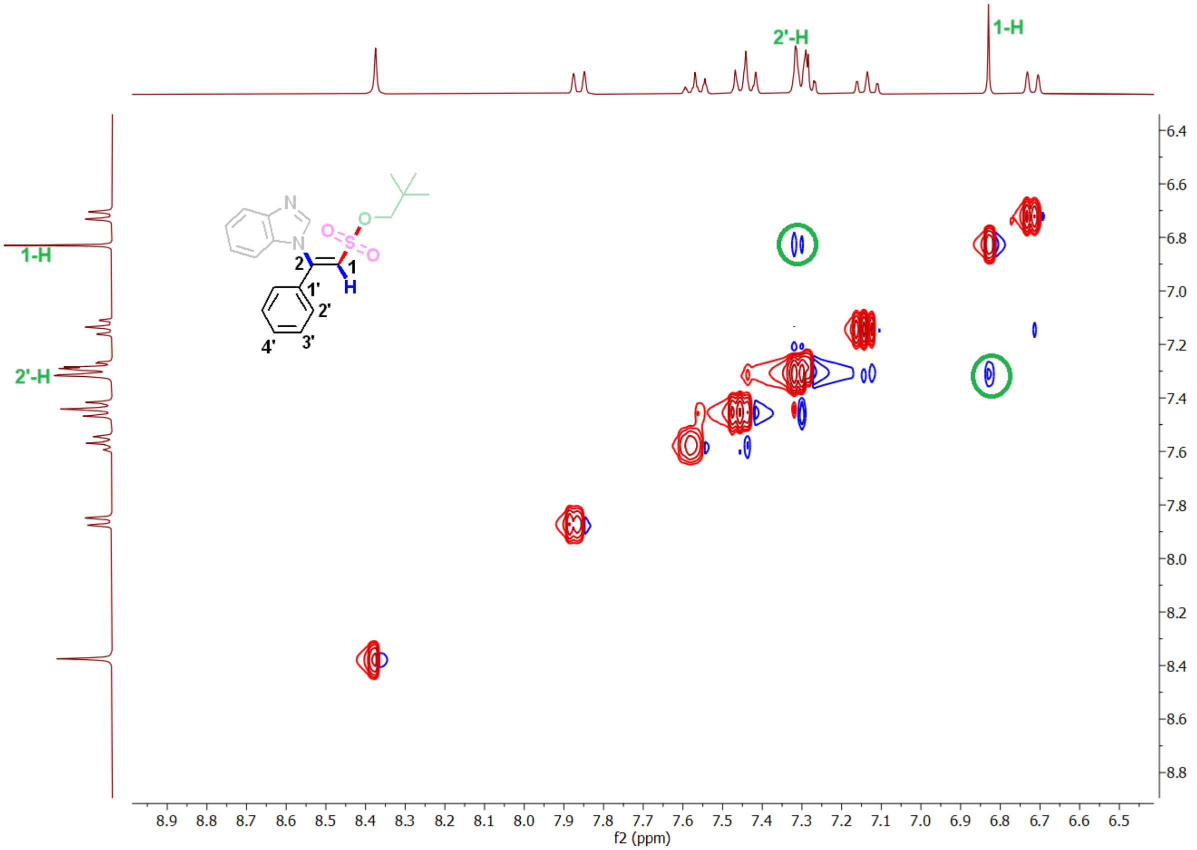

^{1}H NMR (400 MHz, $CDCl_3$) of **6k** (see procedure)

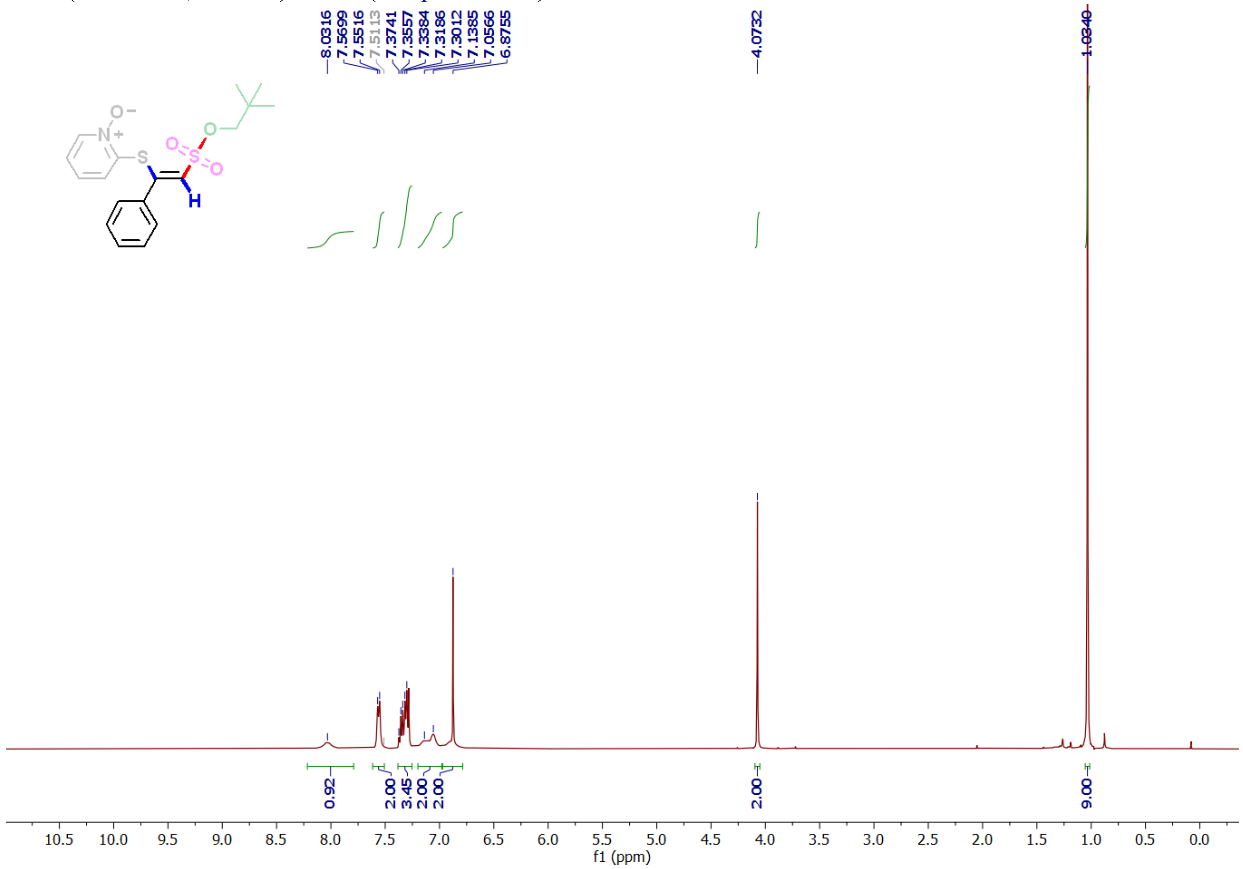

^{1}H NMR (400 MHz, pyridine-*d5*) of **6k**

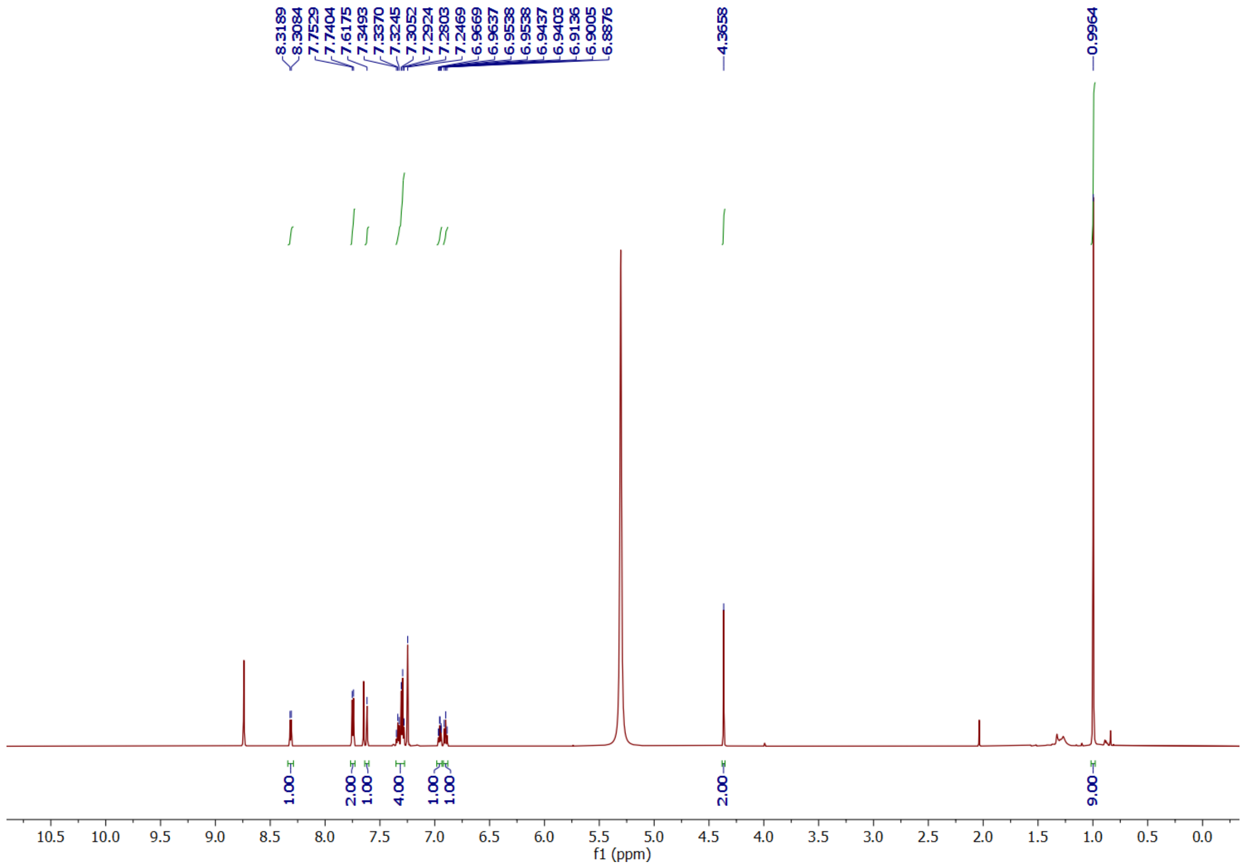

^{13}C NMR (101 MHz, pyridine-*d5*) of **6k**

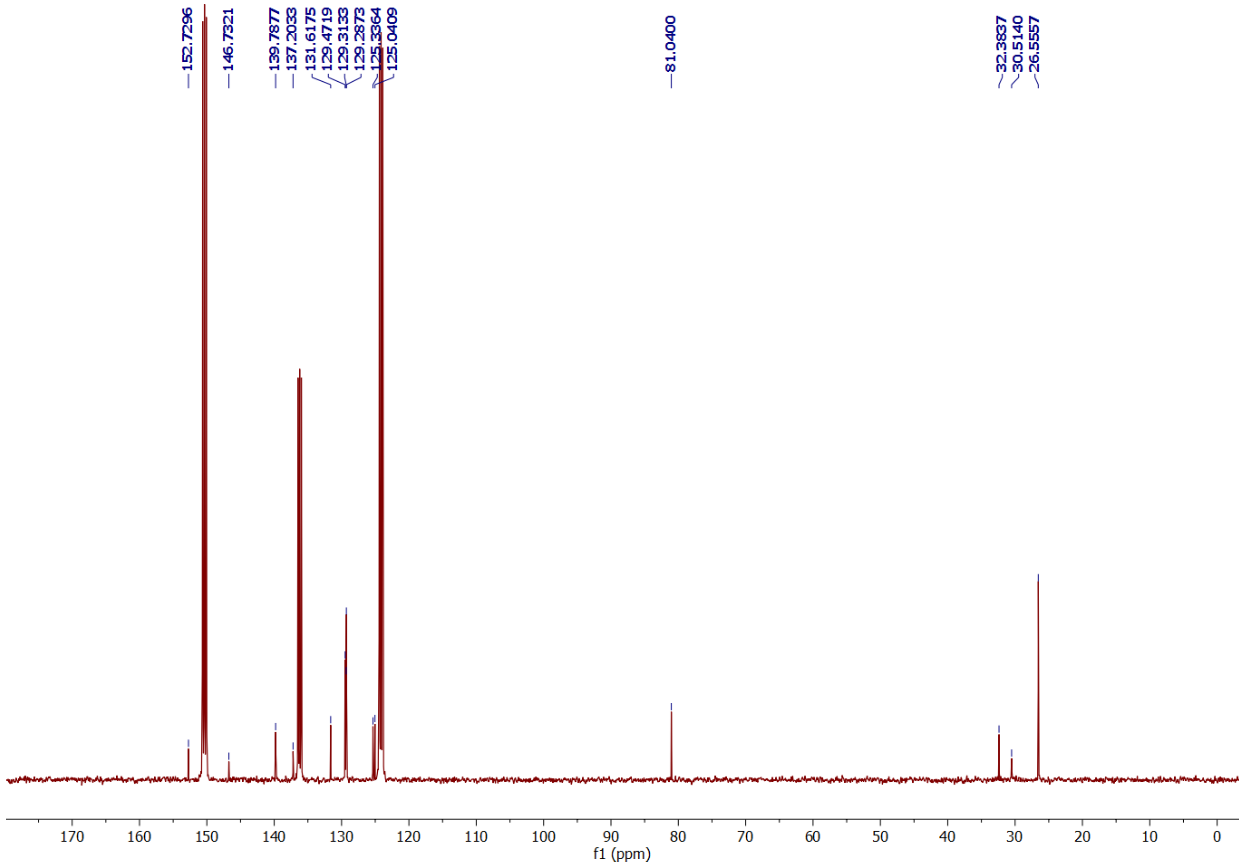

NOE Spectrum for **6k**

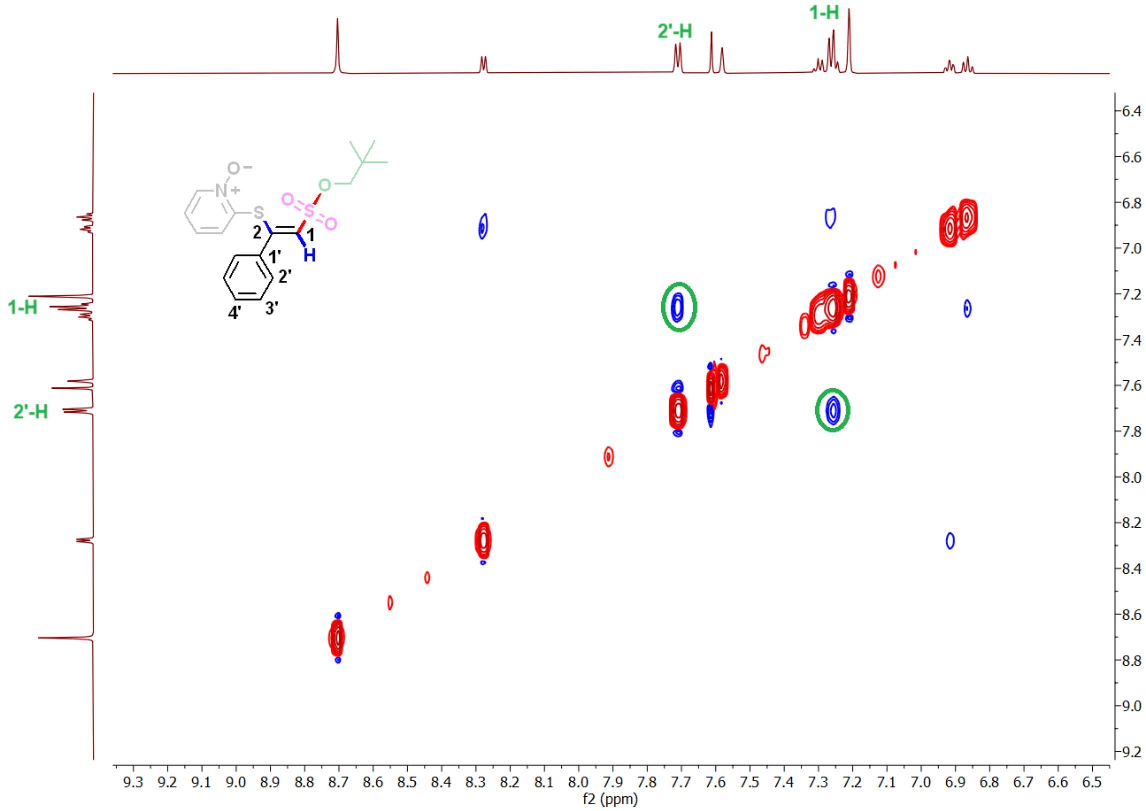

^{1}H NMR (300 MHz, $CDCl_3$) of **6l** (see procedure)

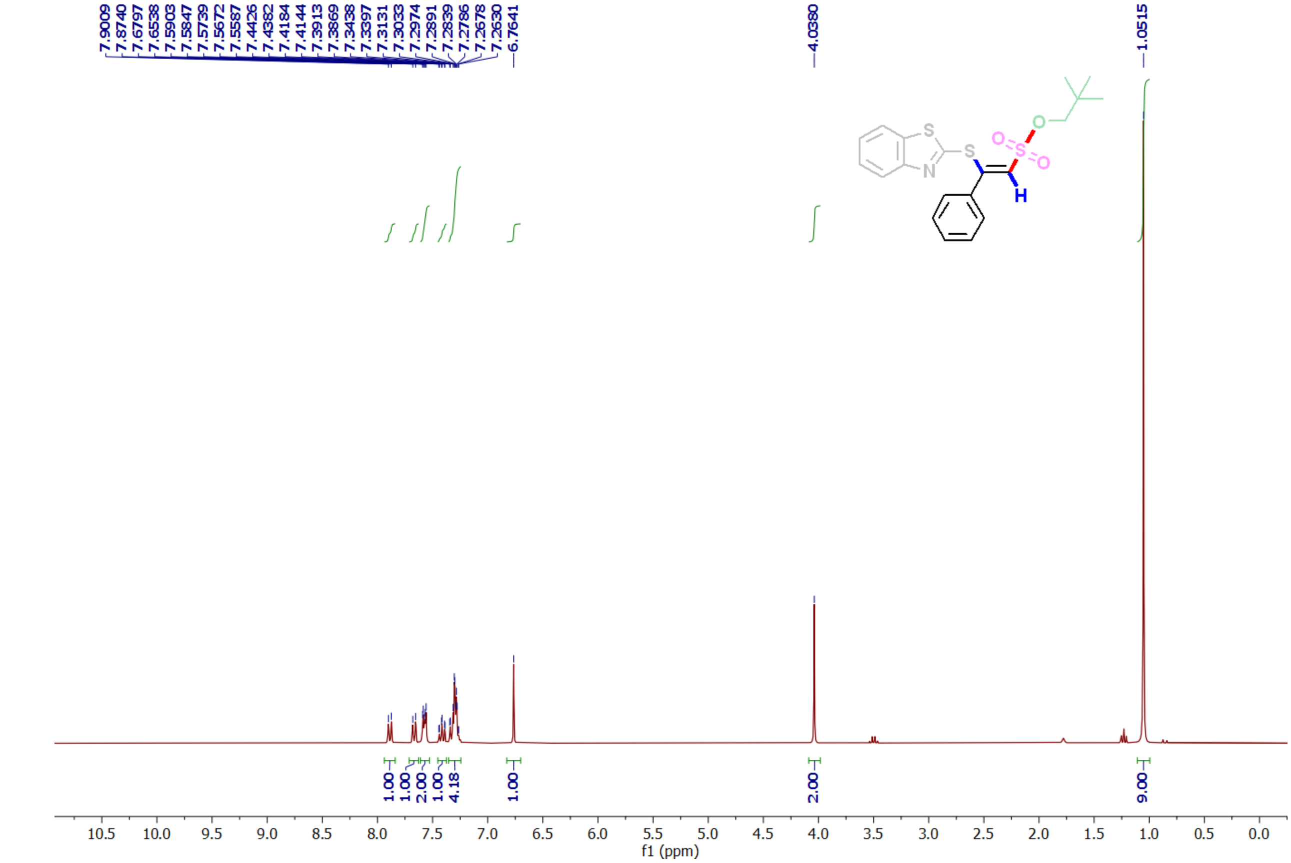

^{13}C NMR (75 MHz, $CDCl_3$) of **6l**

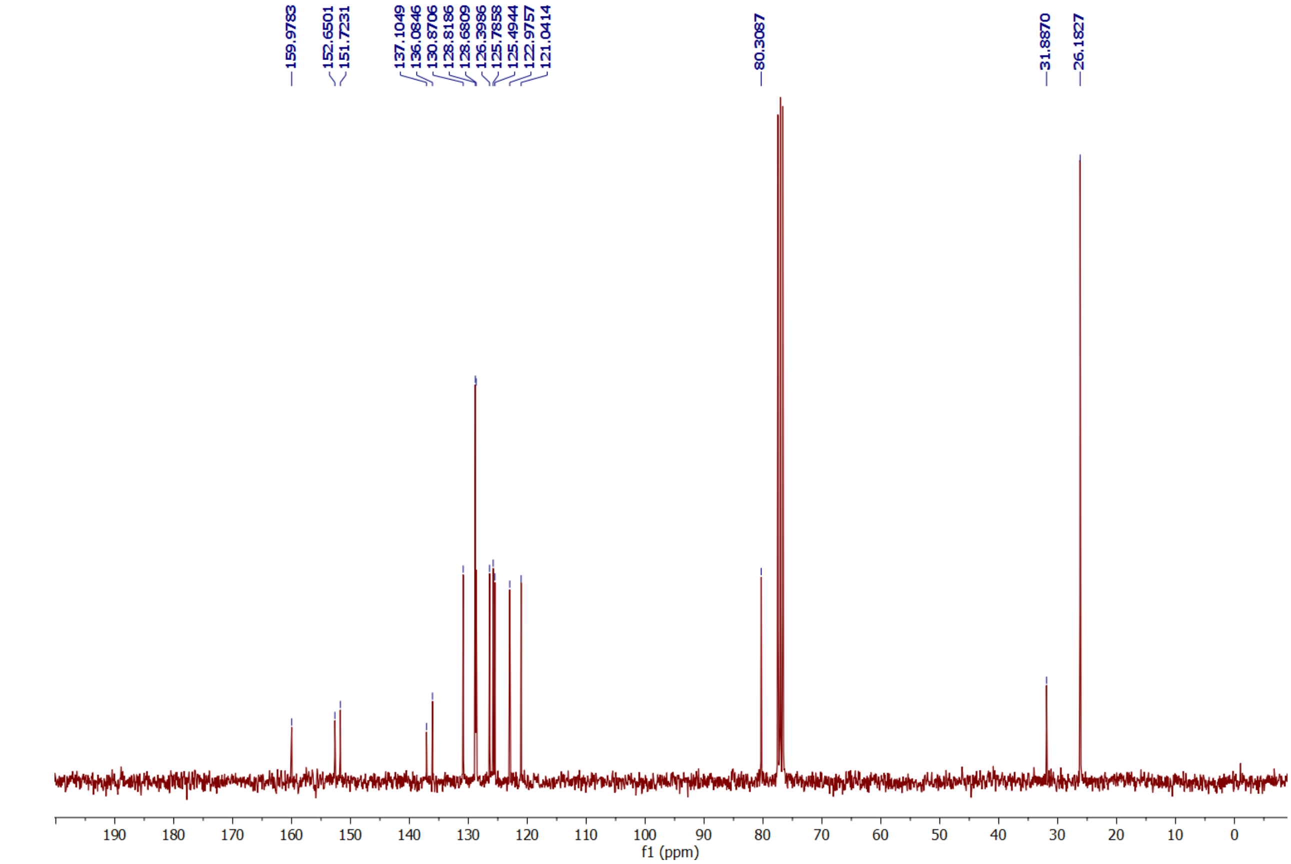

NOE Spectrum for **6l**

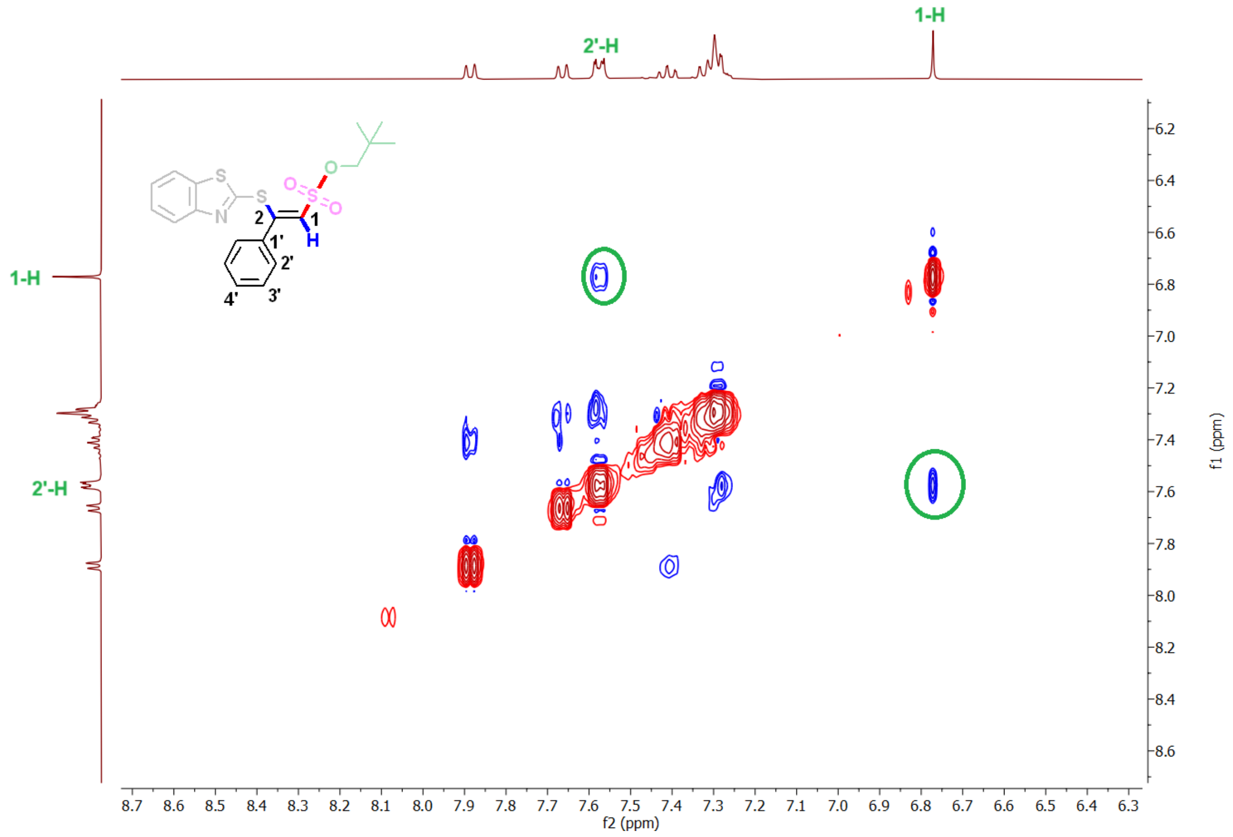

^{1}H NMR (300 MHz, $CDCl_3$) of **6m** (see procedure)

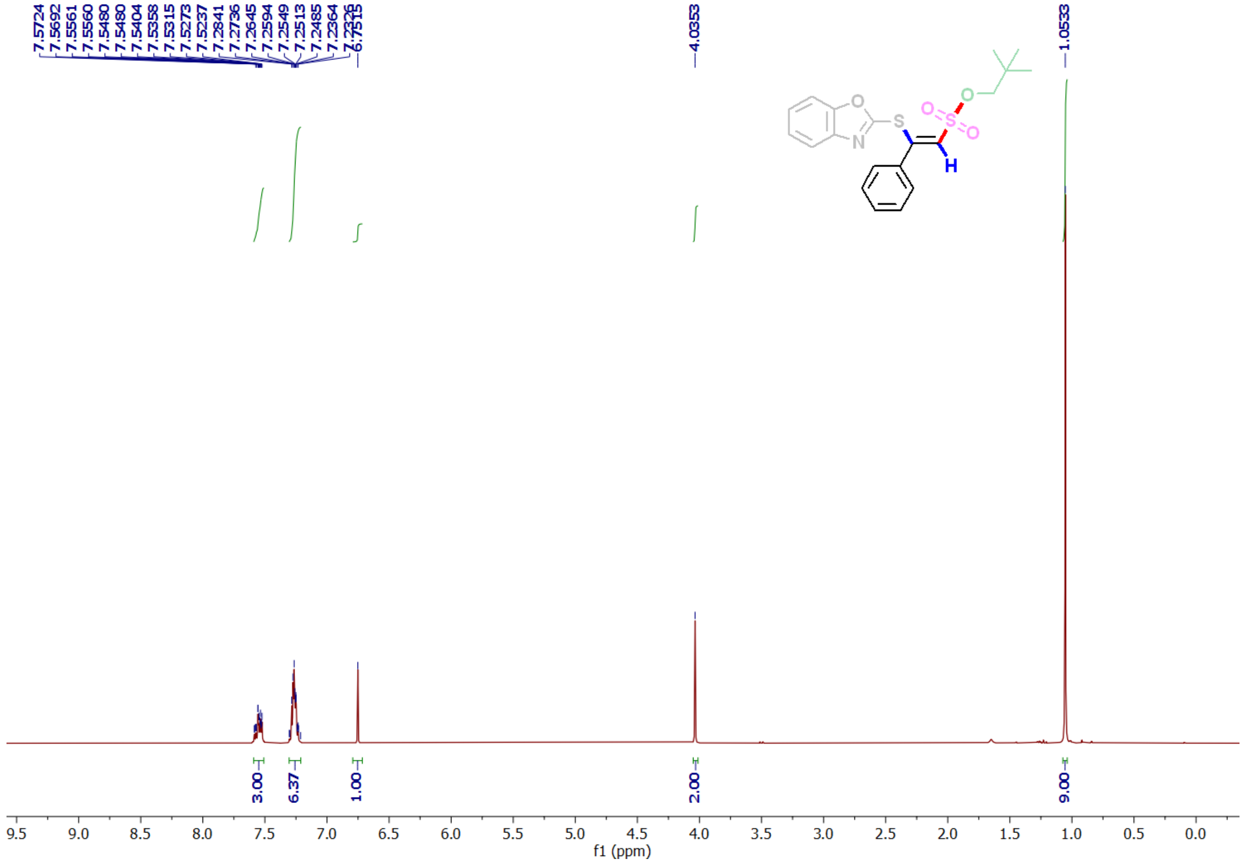

^{13}C NMR (75 MHz, $CDCl_3$) of **6m**

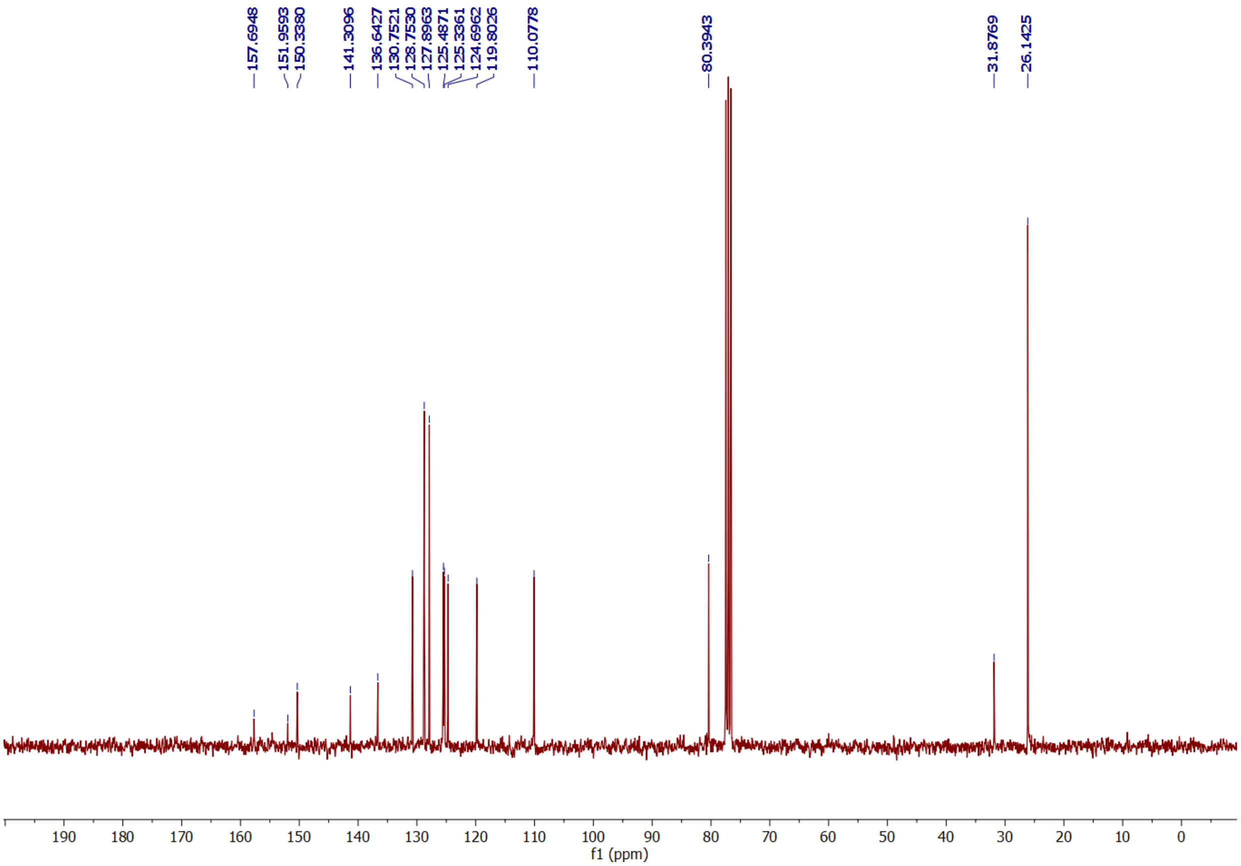

NOE Spectrum for **6m**

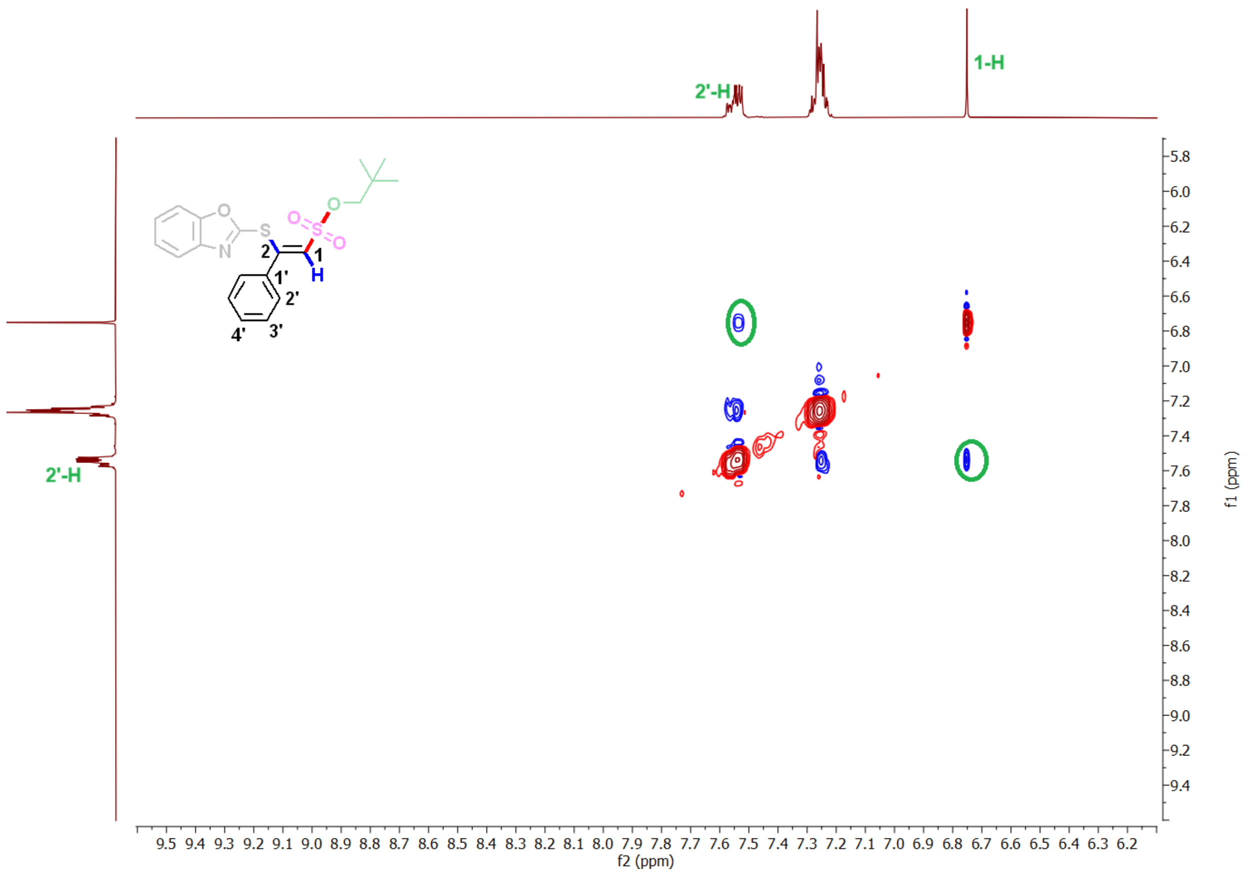

^{1}H NMR (300 MHz, $CDCl_3$) of **6m-D1** (see procedure)

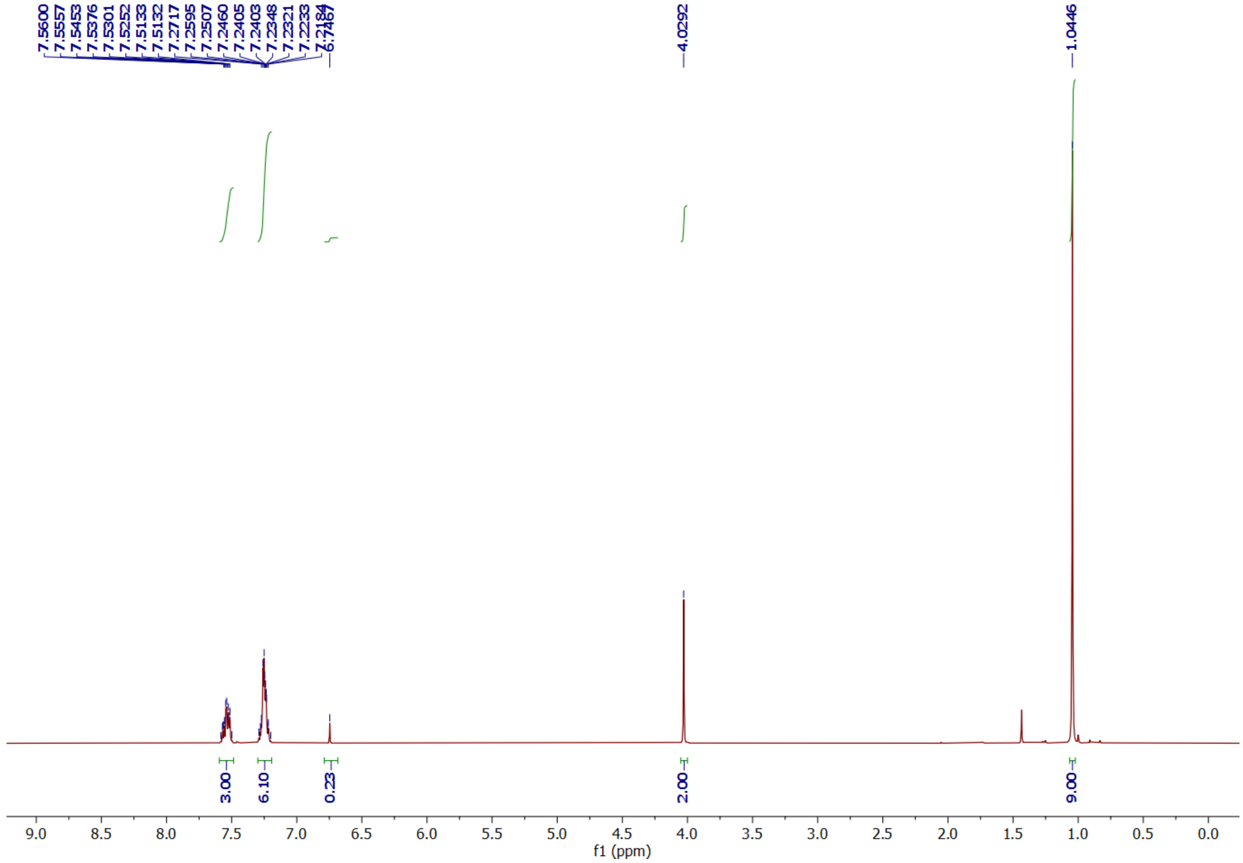

^{1}H NMR (300 MHz, $CDCl_3$) of **6m-D2** (see procedure)

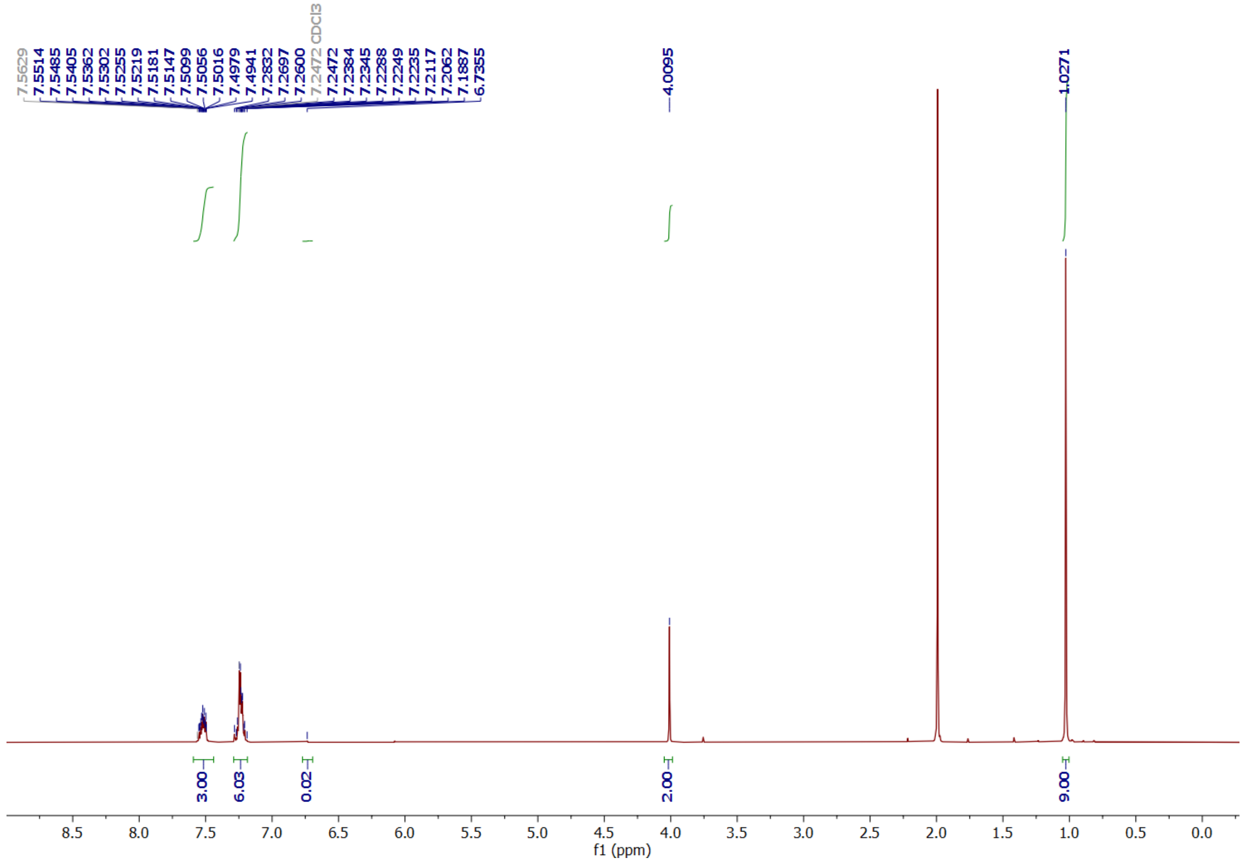

^{1}H NMR (400 MHz, DMSO-d_6) of **6n** (see procedure)

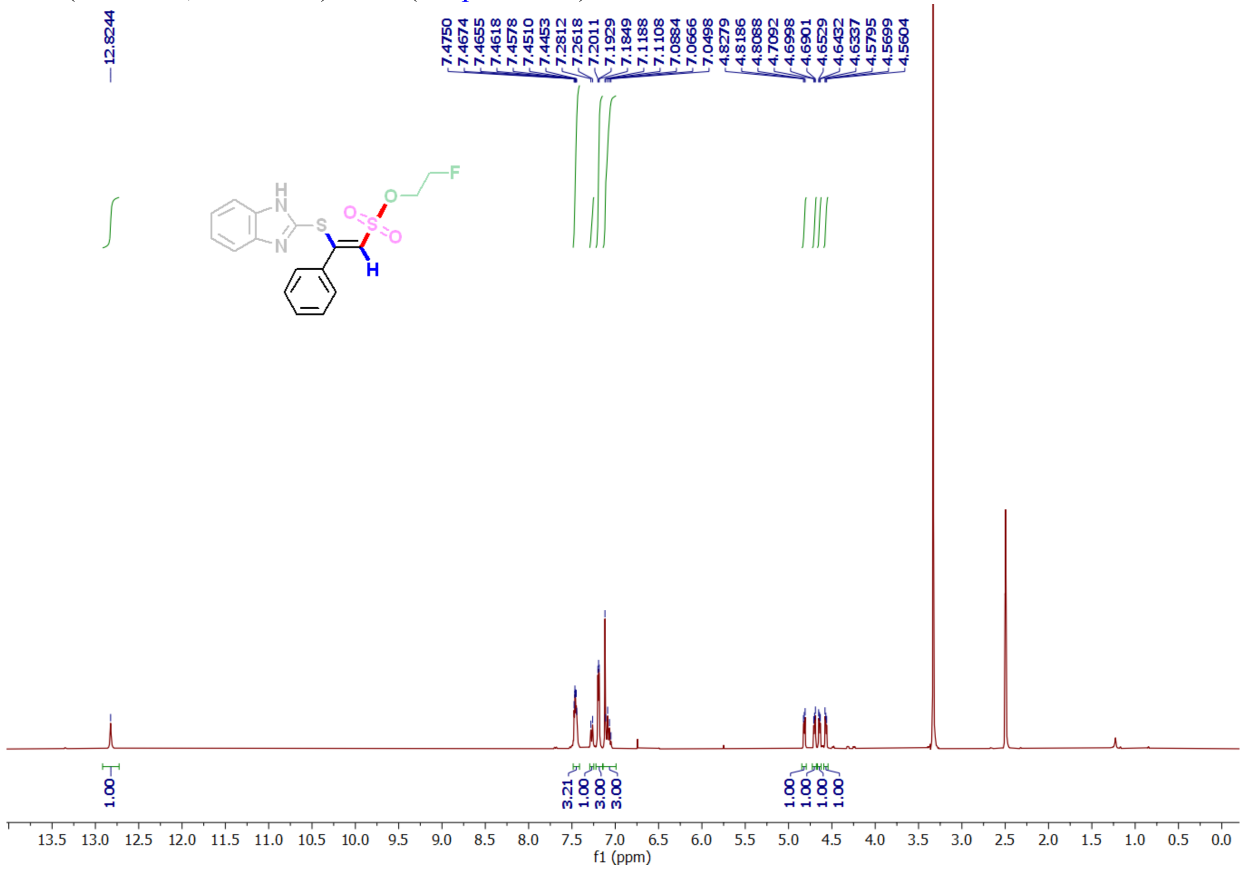

^{13}C NMR (101 MHz, DMSO-d_6) of **6n**

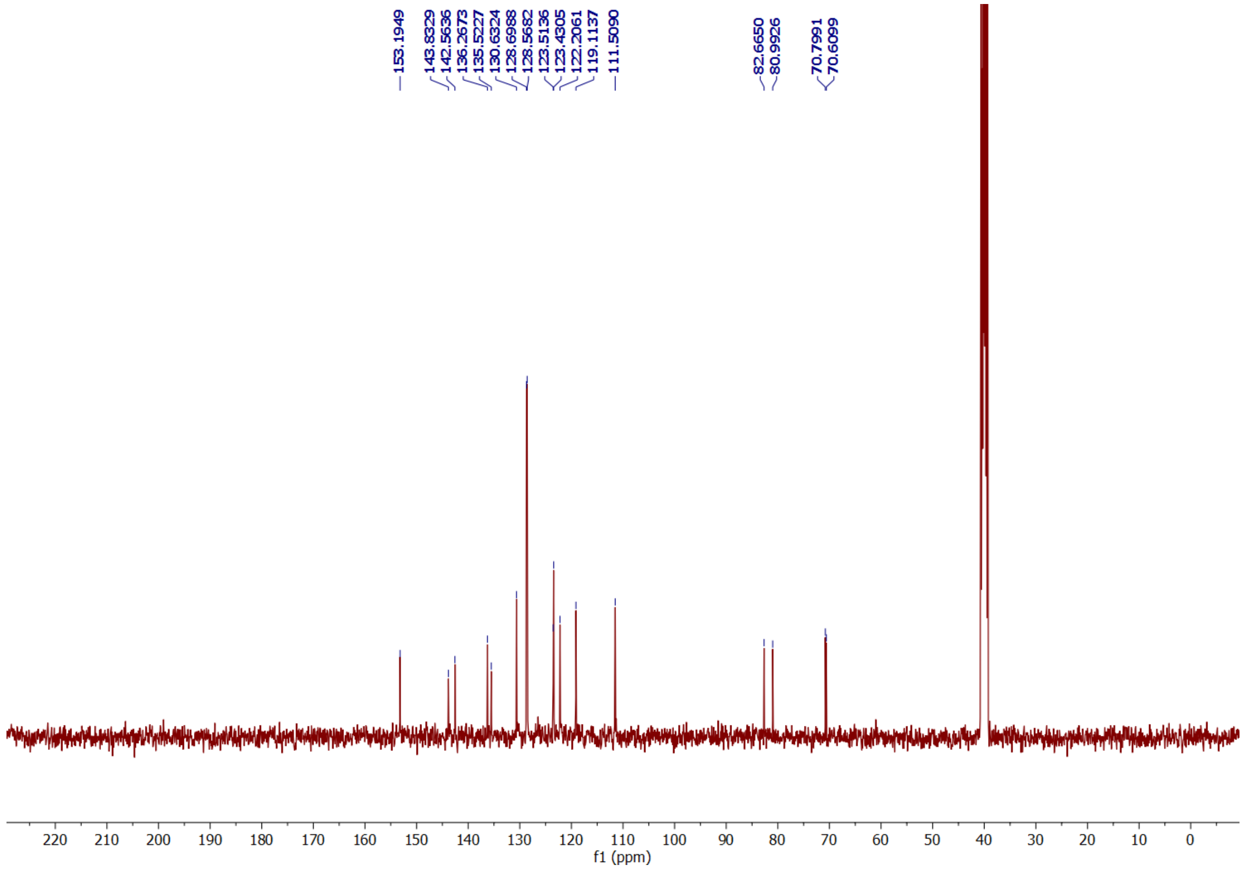

NOE Spectrum for **6n**

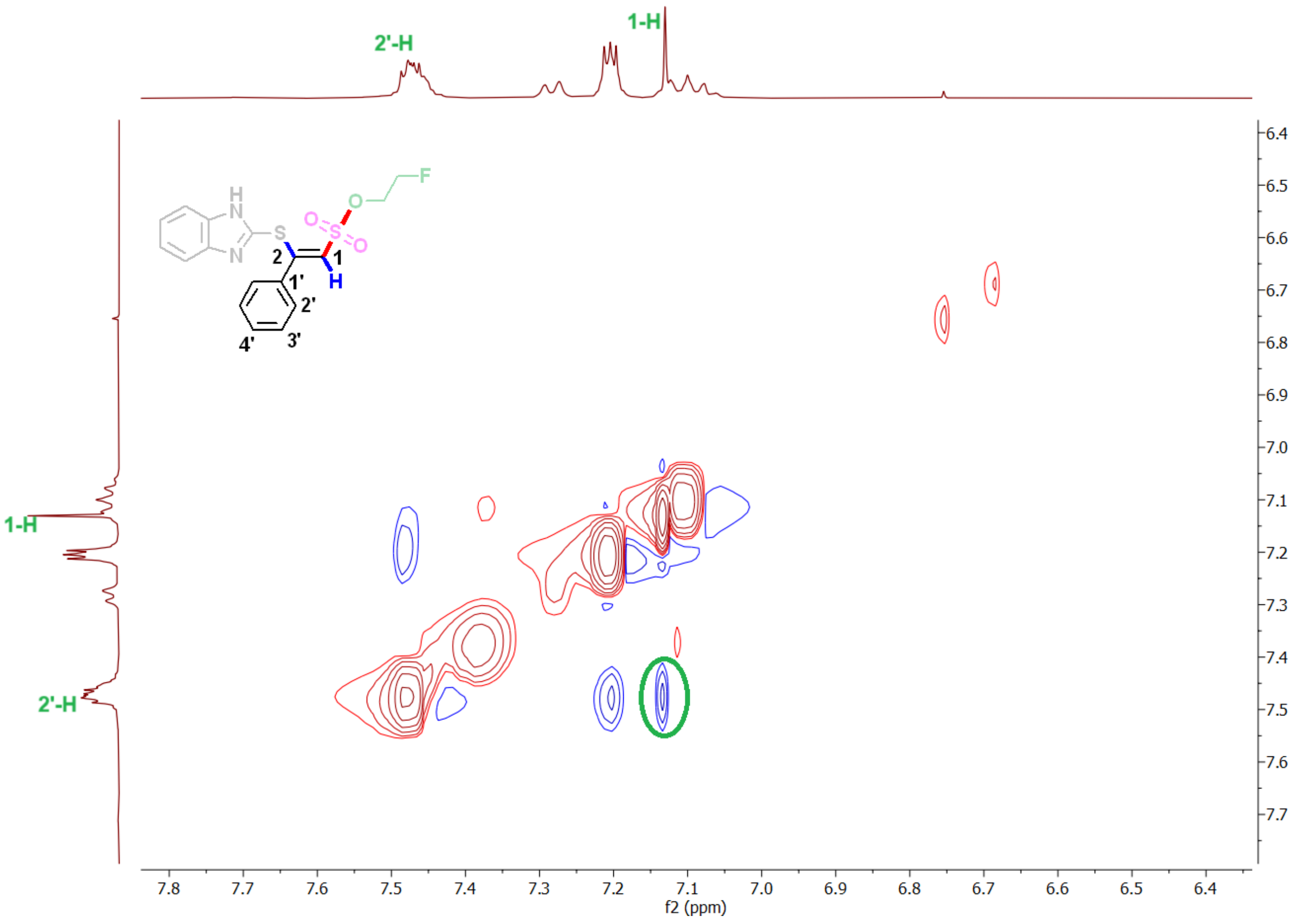

^{1}H NMR (300 MHz, $CDCl_3$) of **6o** (see procedure)

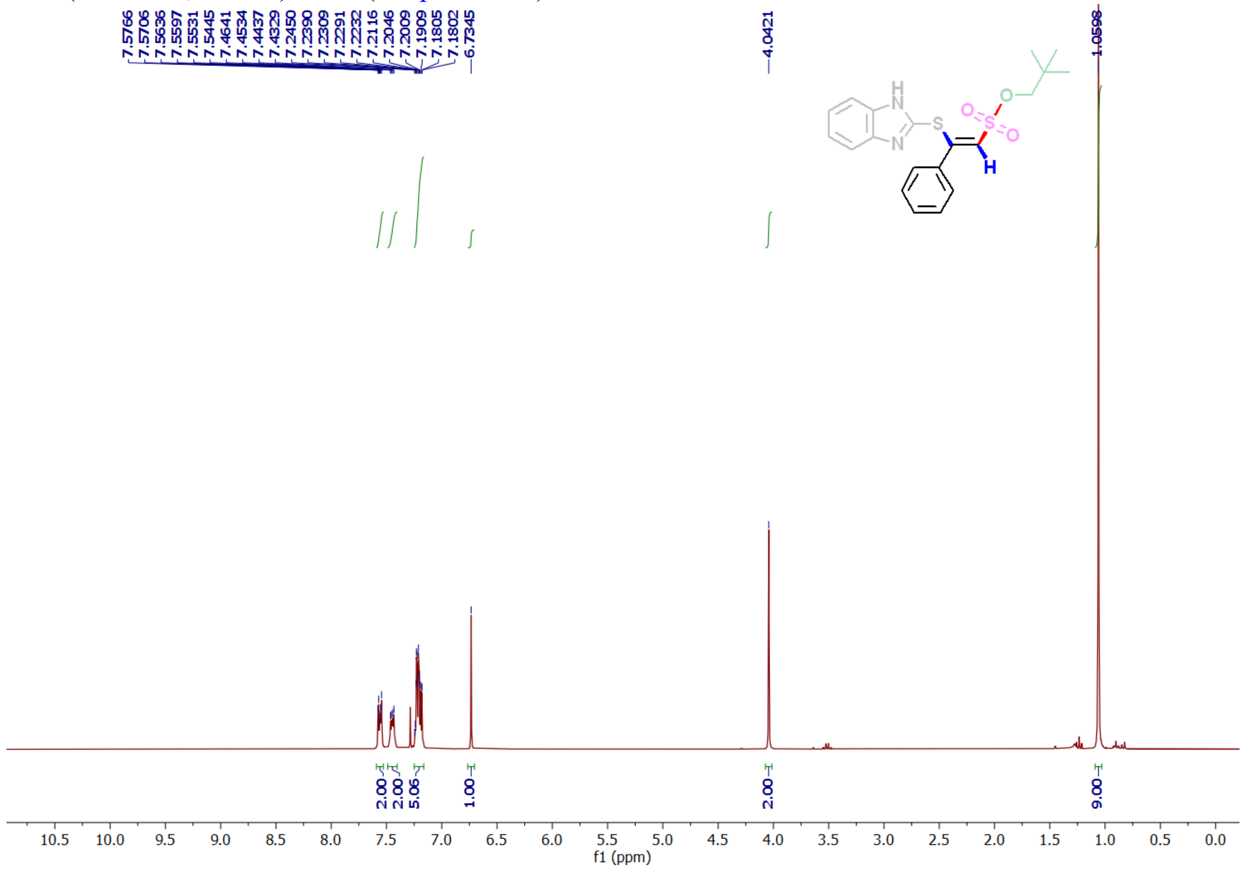

^{13}C NMR (75 MHz, $CDCl_3$) of **6o**

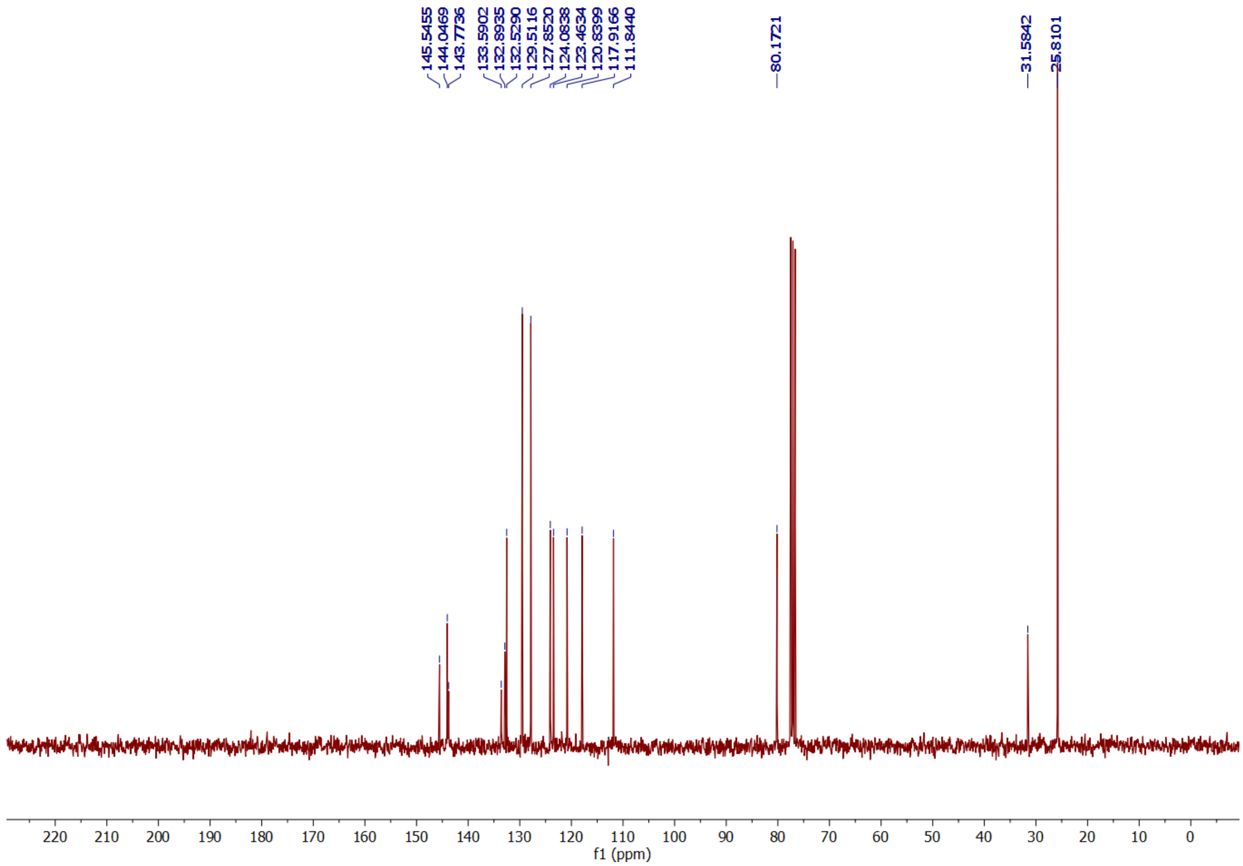

NOE Spectrum for **6o**

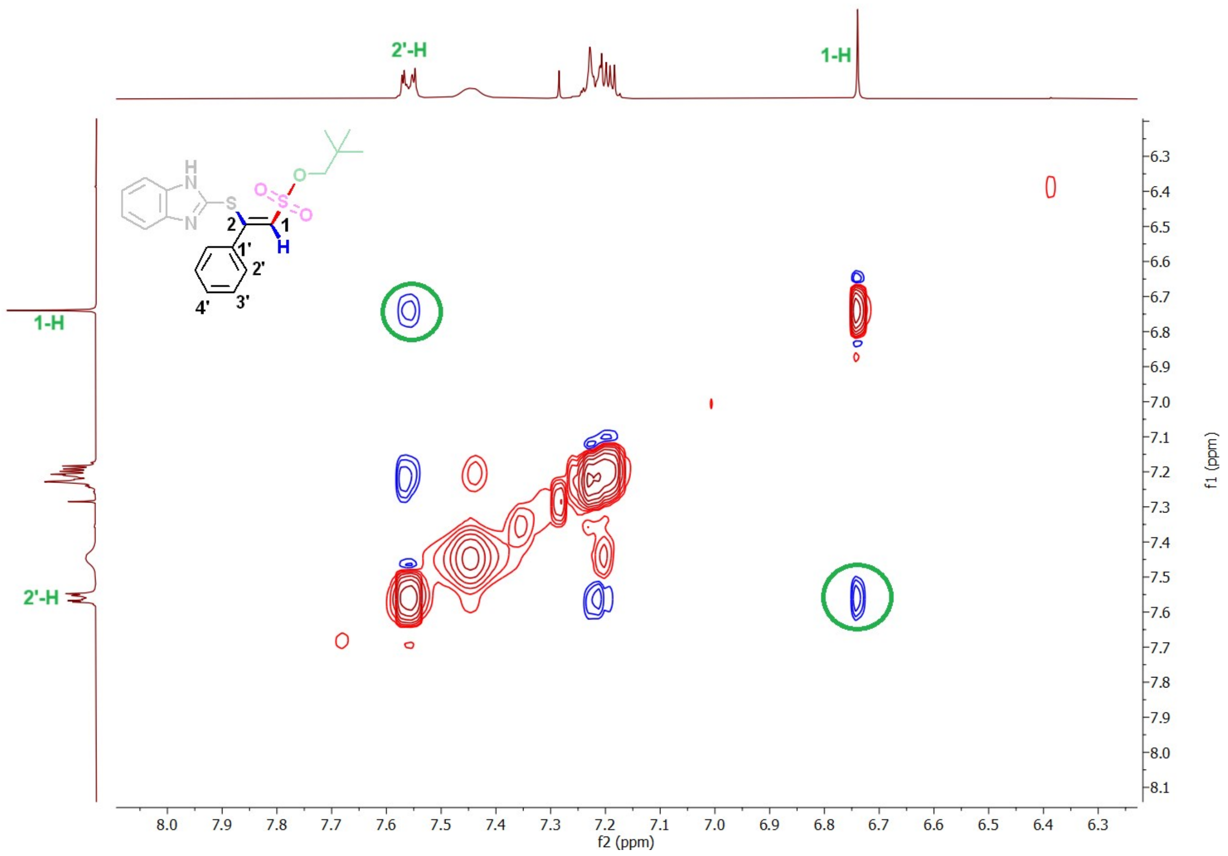

^{1}H NMR (400 MHz, $CDCl_3$) of **7a** (see procedure)

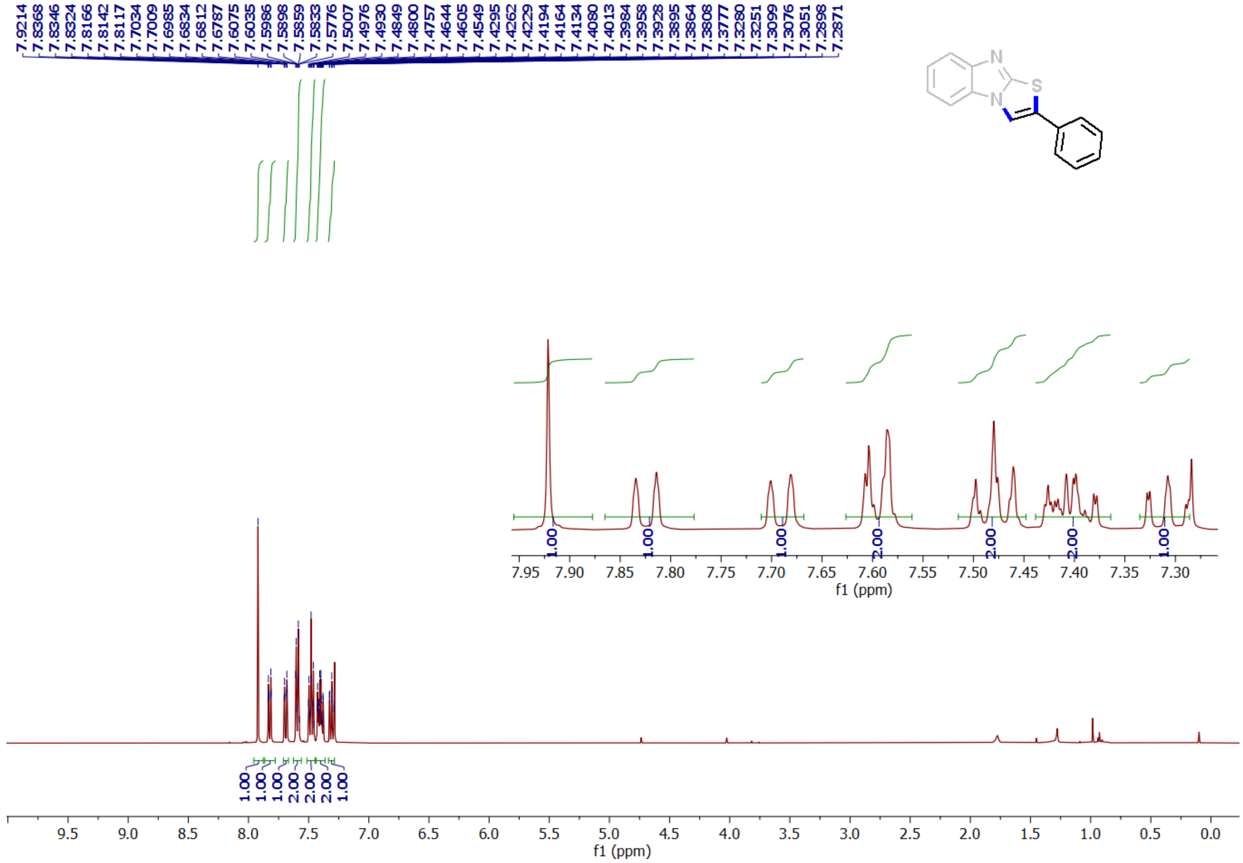

^{13}C NMR (101MHz, $CDCl_3$) of **7a**

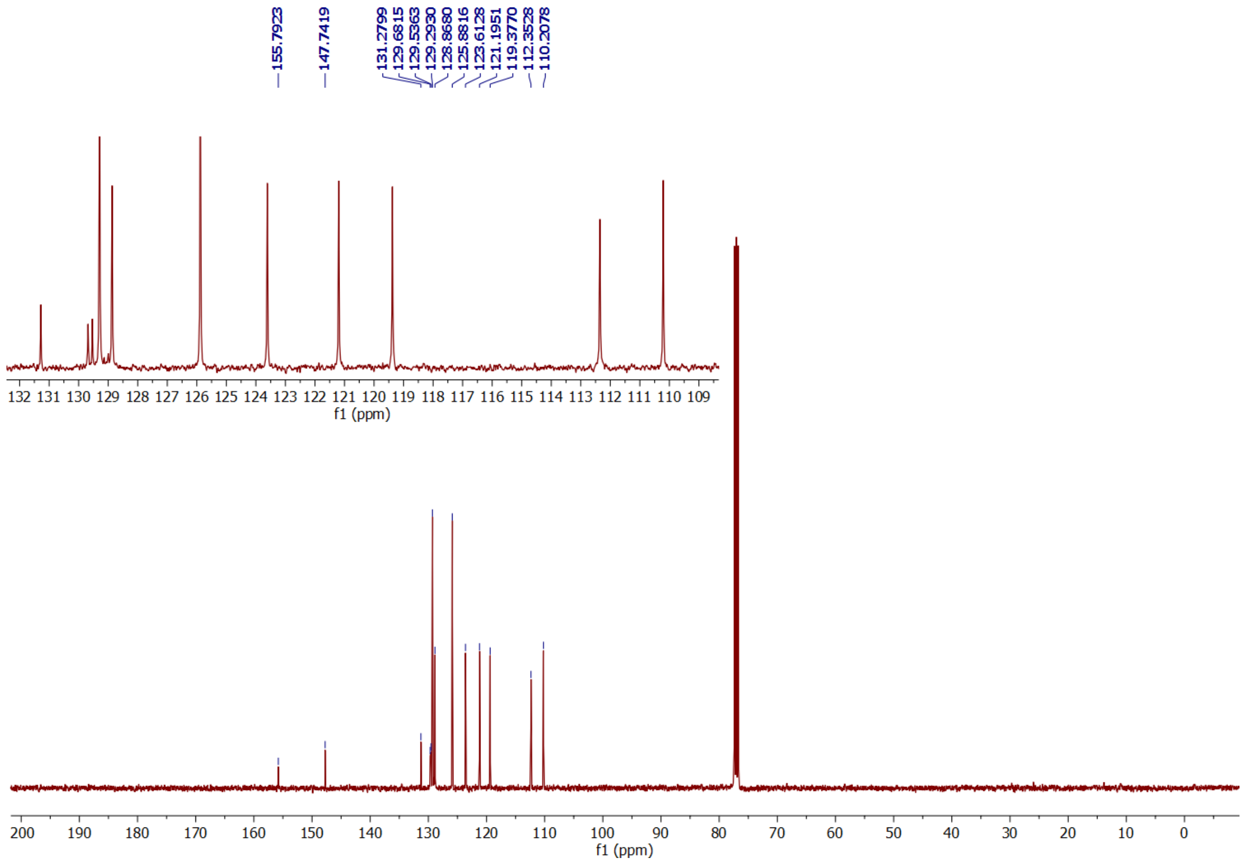

^{1}H NMR (400 MHz, $CDCl_3$) of **6p** (see procedure)

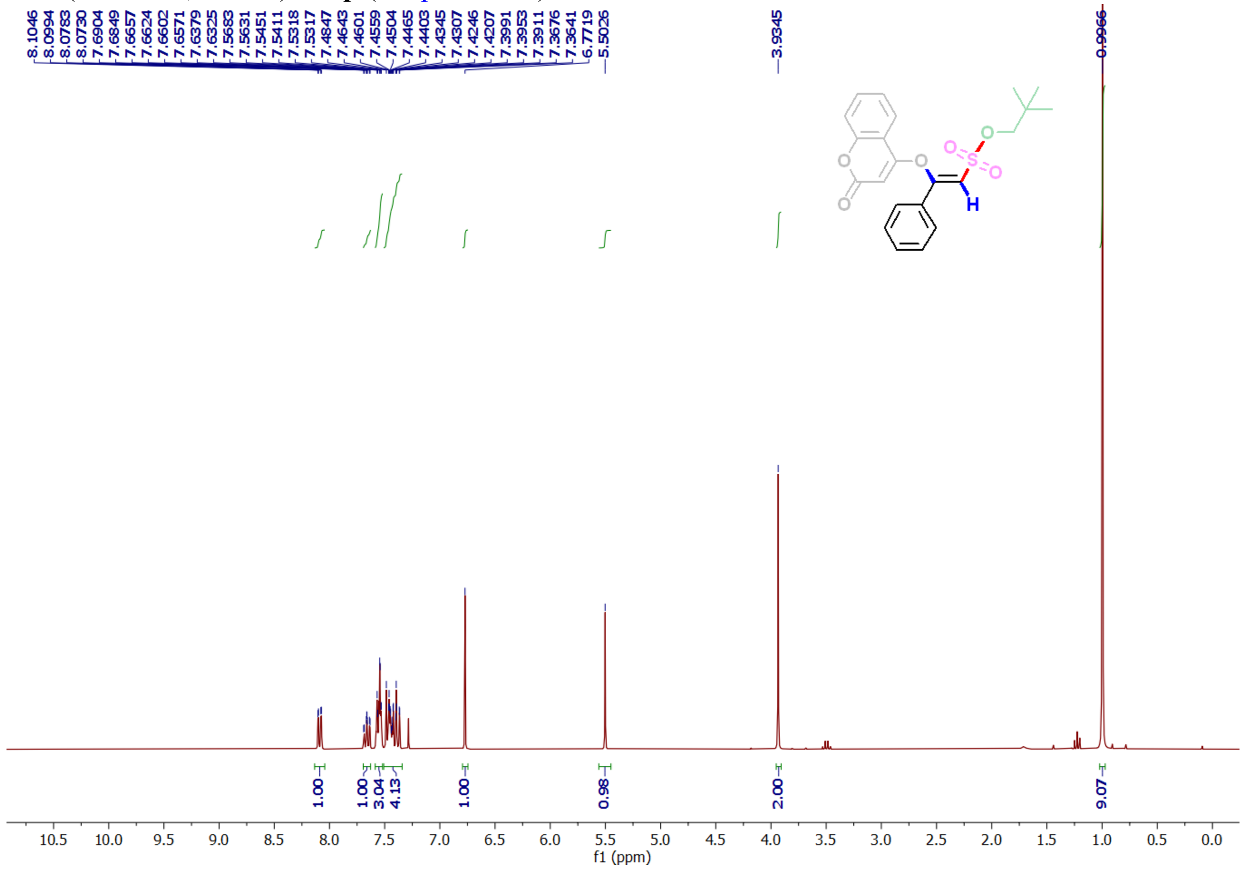

^{13}C NMR (101MHz, $CDCl_3$) of **6p**

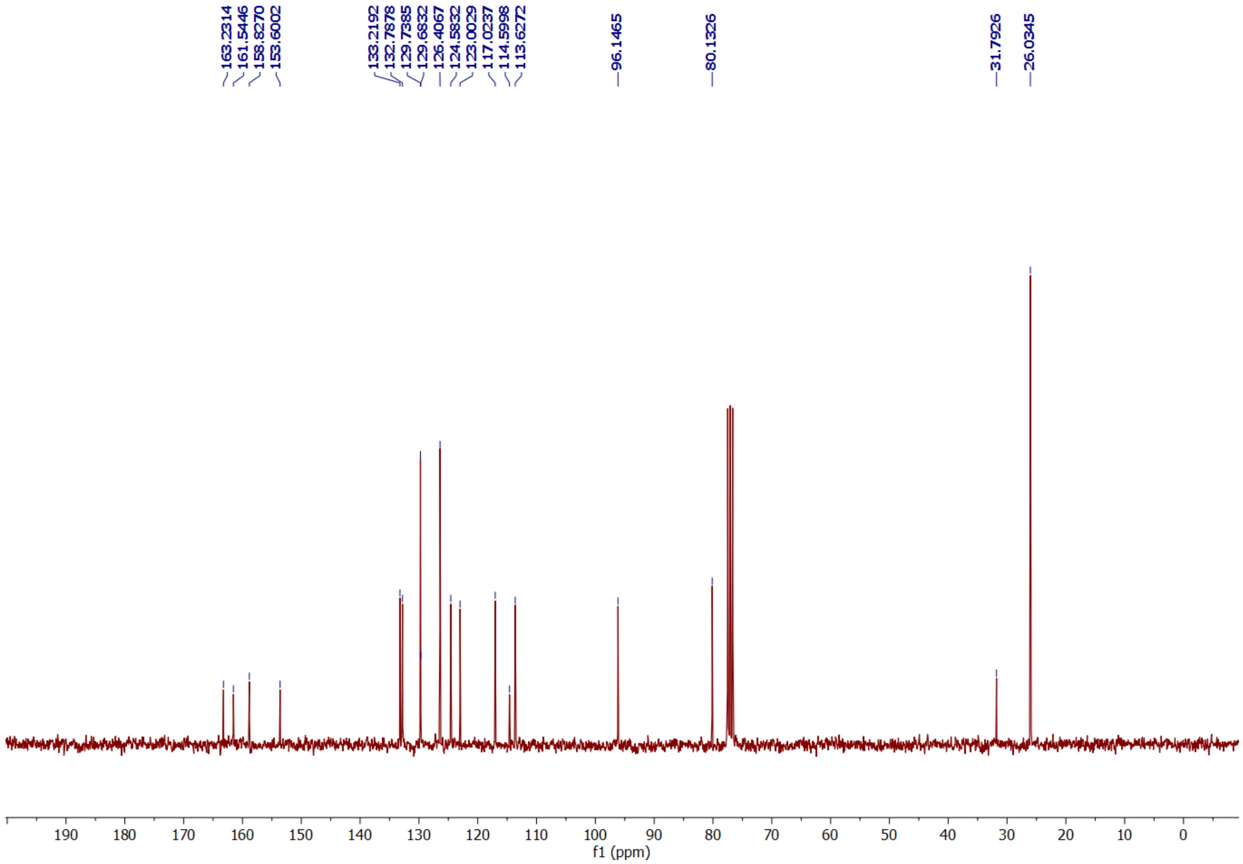

NOE Spectrum for **6p**

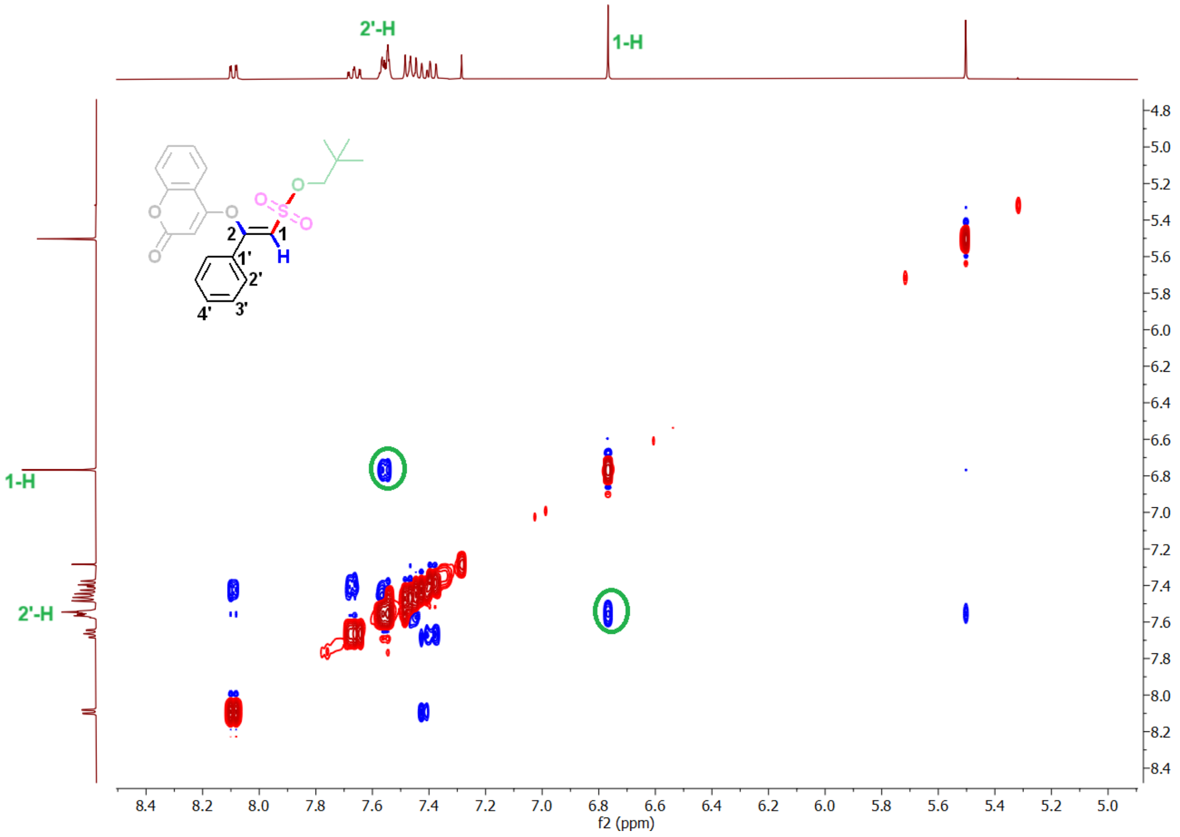

^{1}H NMR (400 MHz, $CDCl_3$) of **6q** (see procedure)

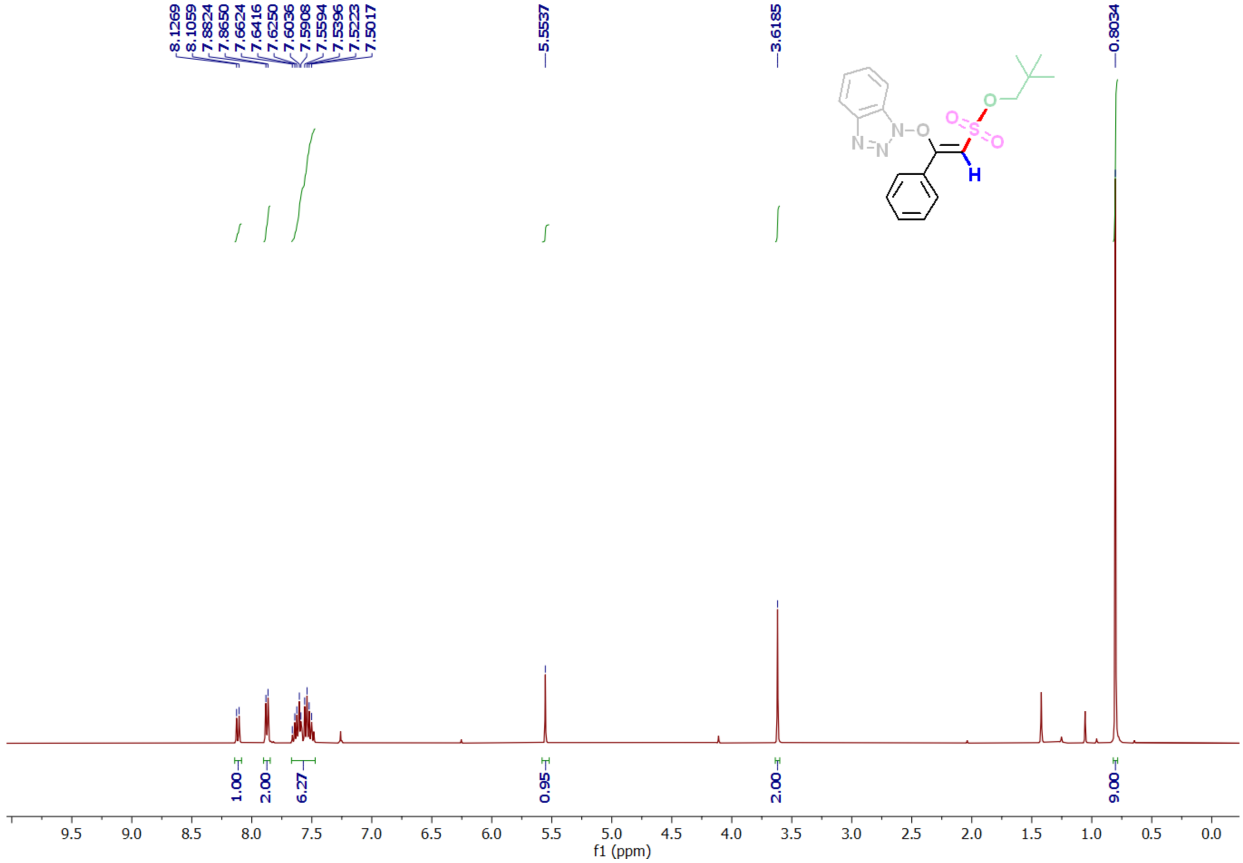

^{13}C NMR (101MHz, $CDCl_3$) of **6q**

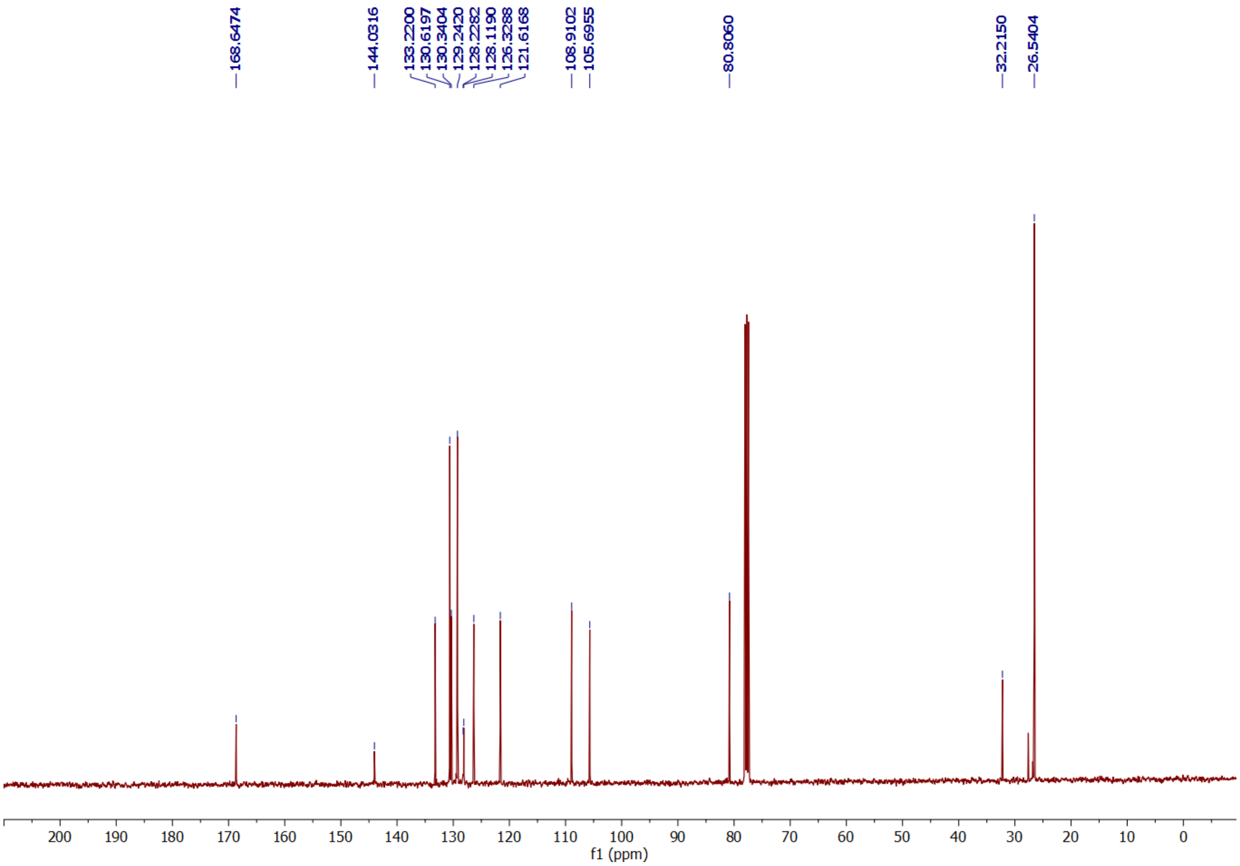

^{1}H NMR (300 MHz, $CDCl_3$) of **6r** (see procedure)

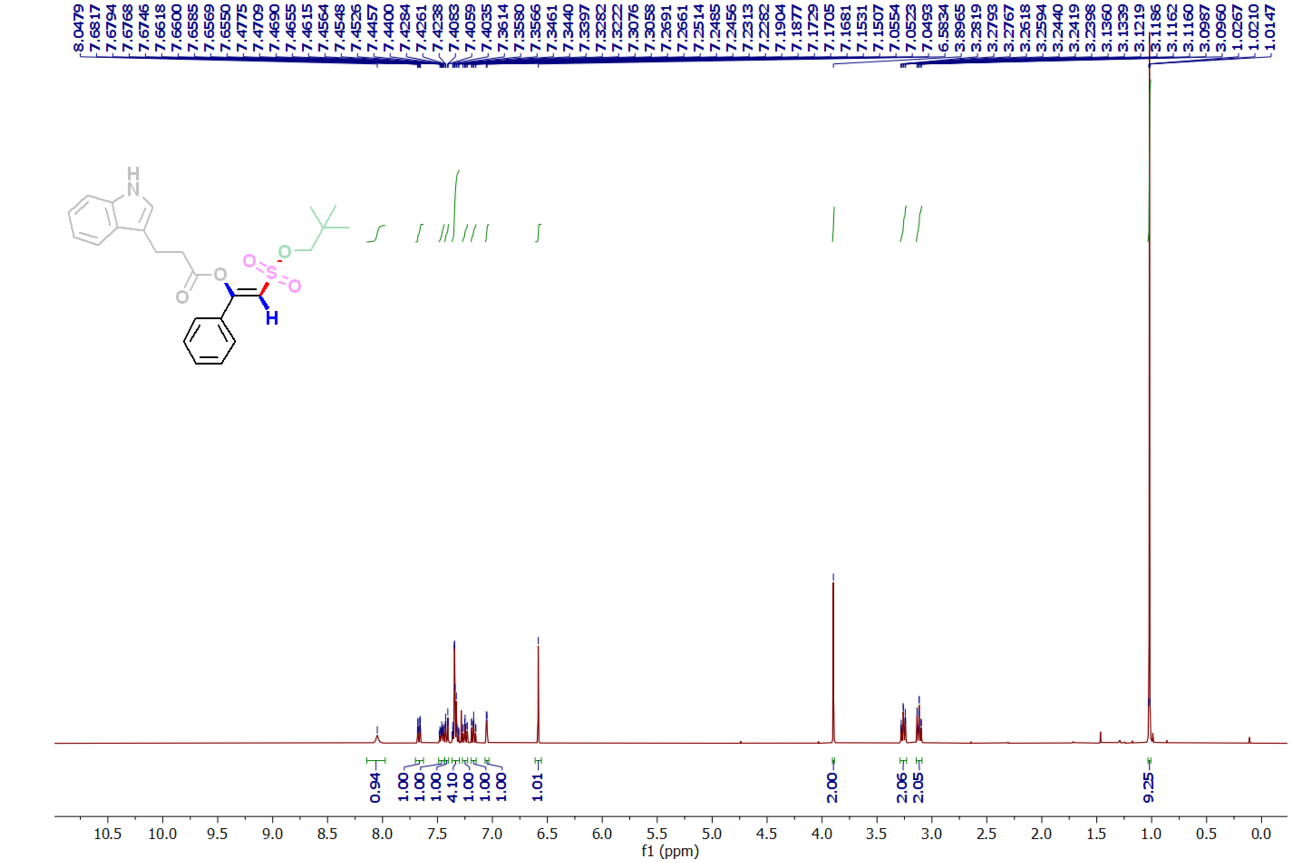

^{13}C NMR (75 MHz, $CDCl_3$) of **6r**

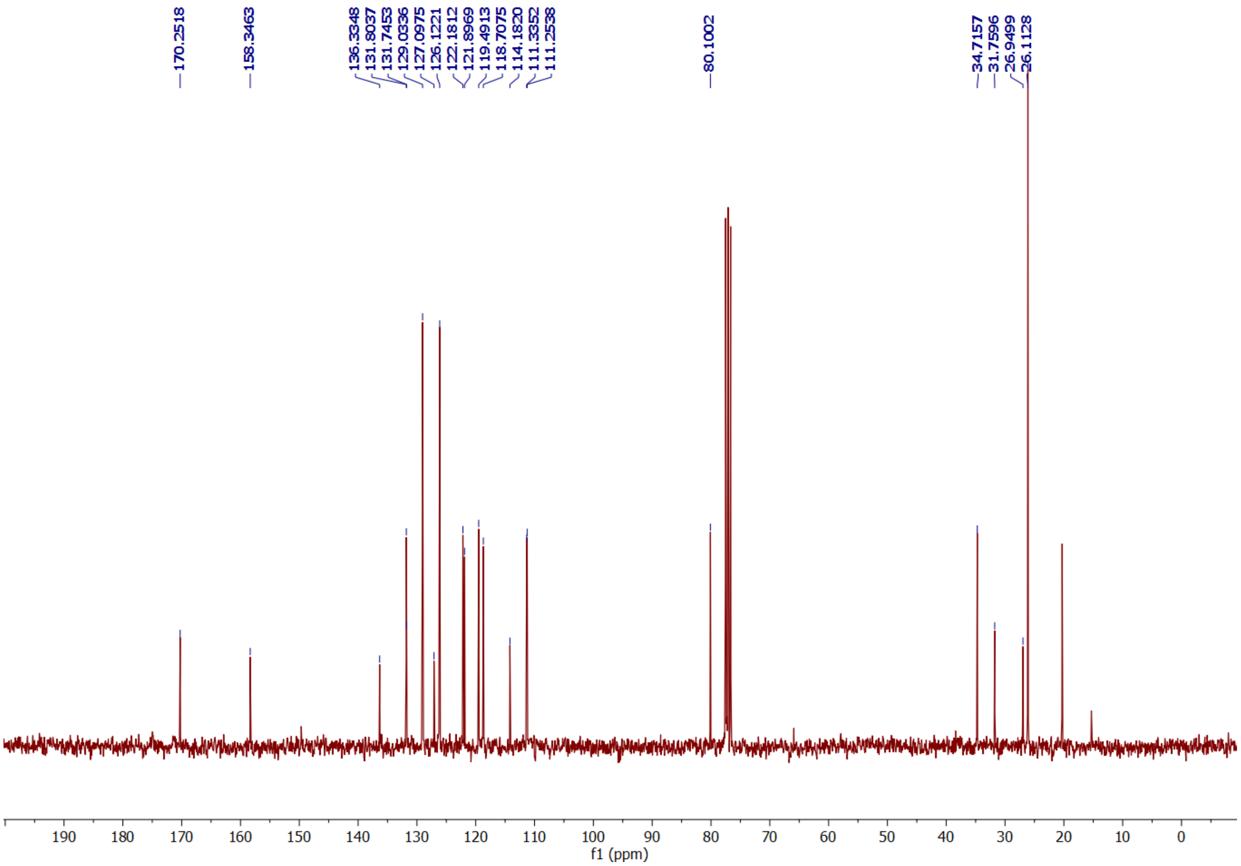

NOE Spectrum for **6r**

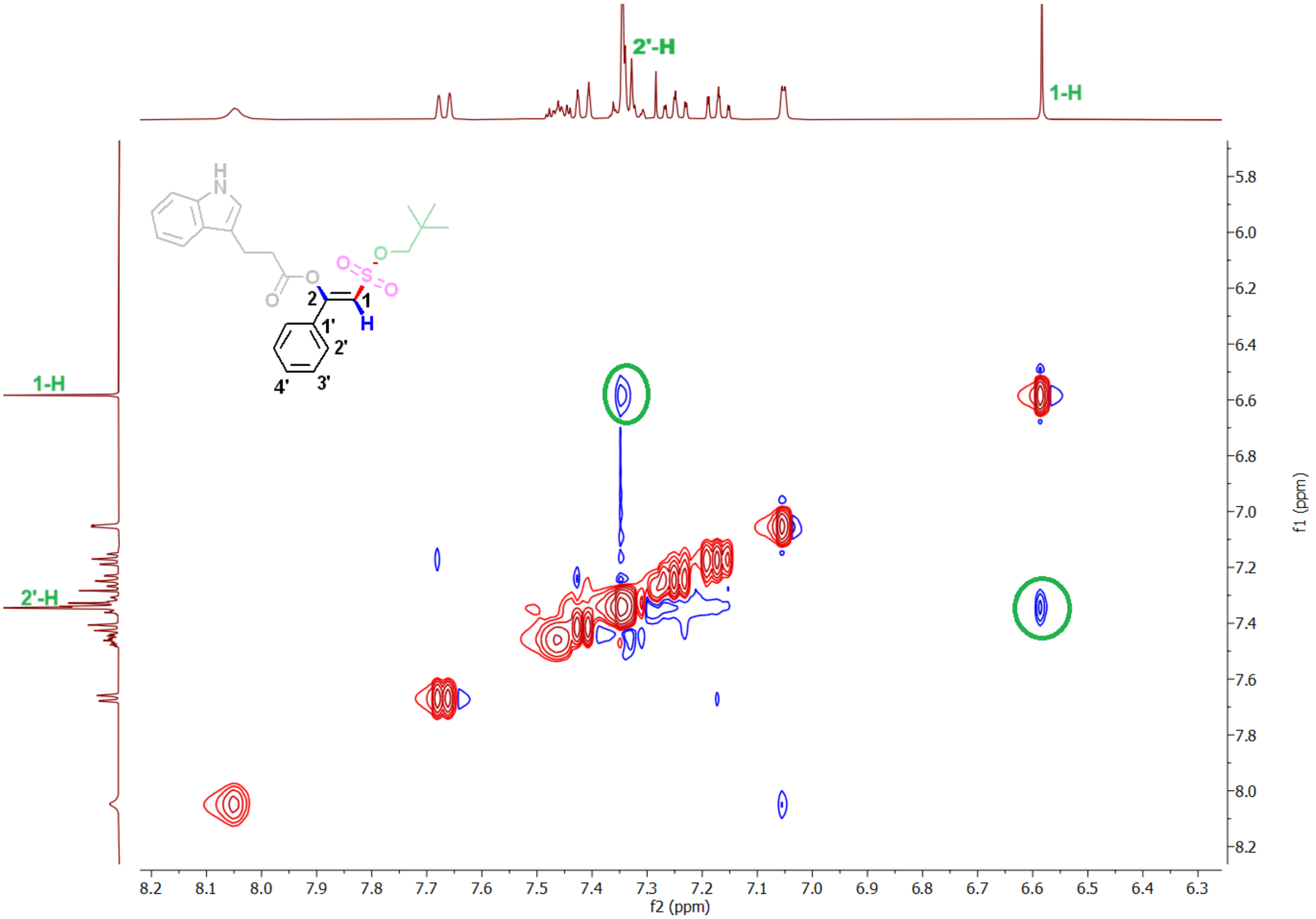

^{1}H NMR (300 MHz, $CDCl_3$) of **6s** (see procedure)

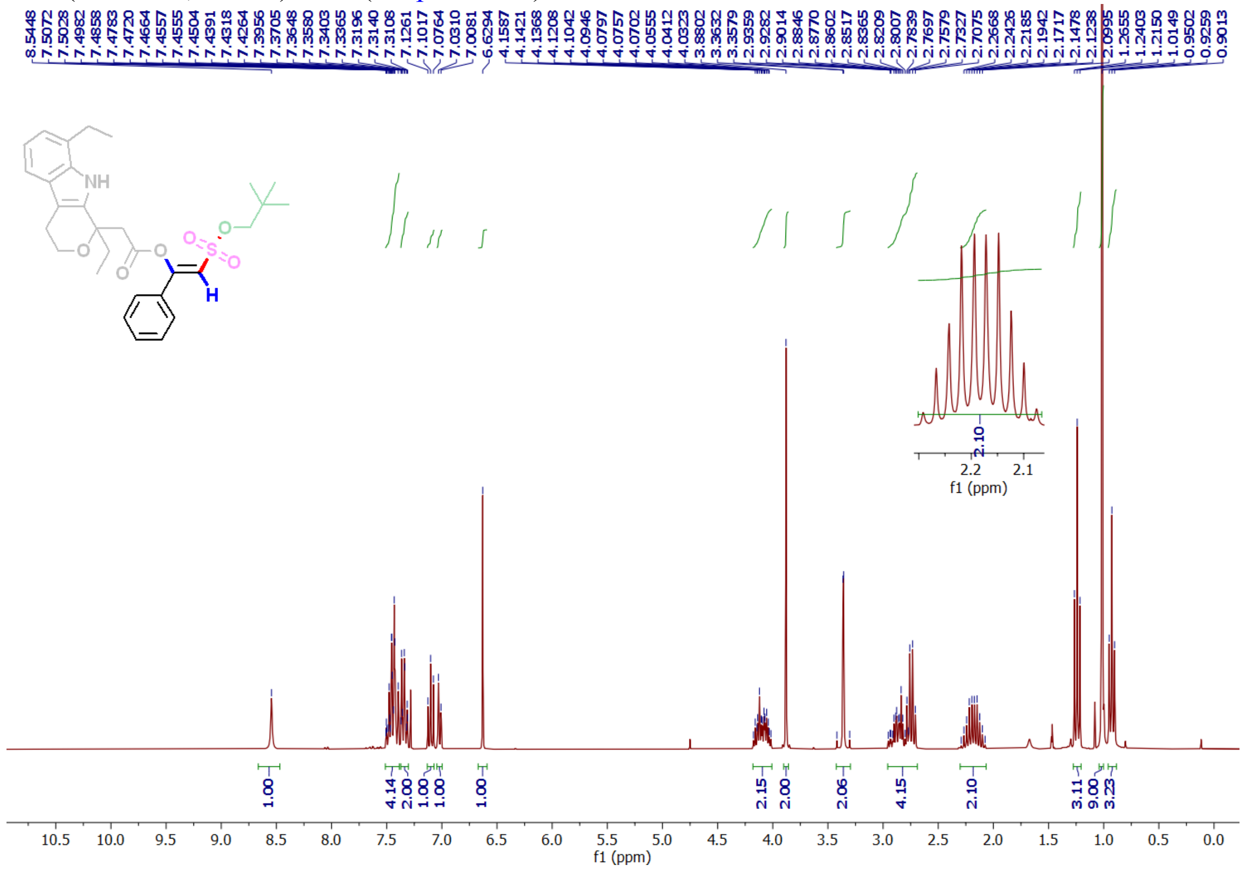

^{13}C NMR (75MHz, $CDCl_3$) of **6s**

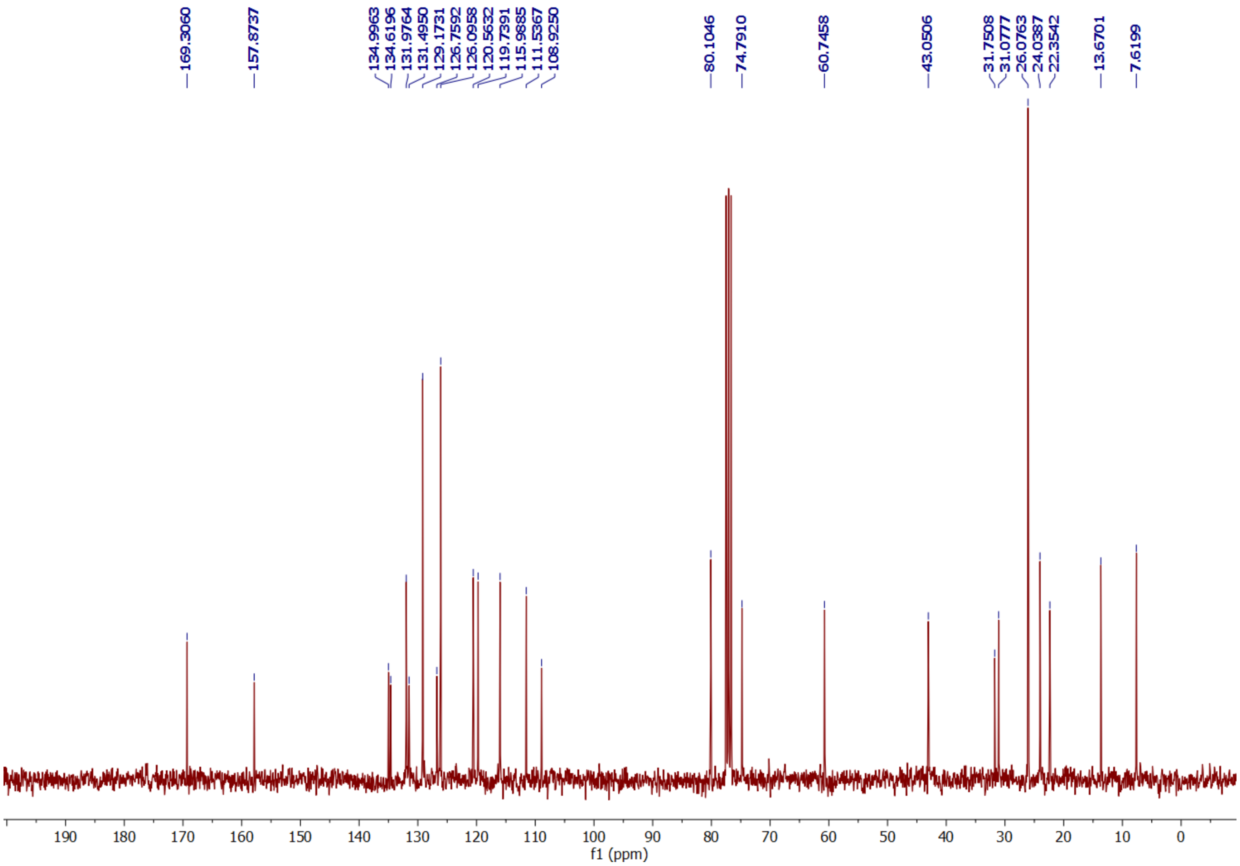

NOE Spectrum for **6s**

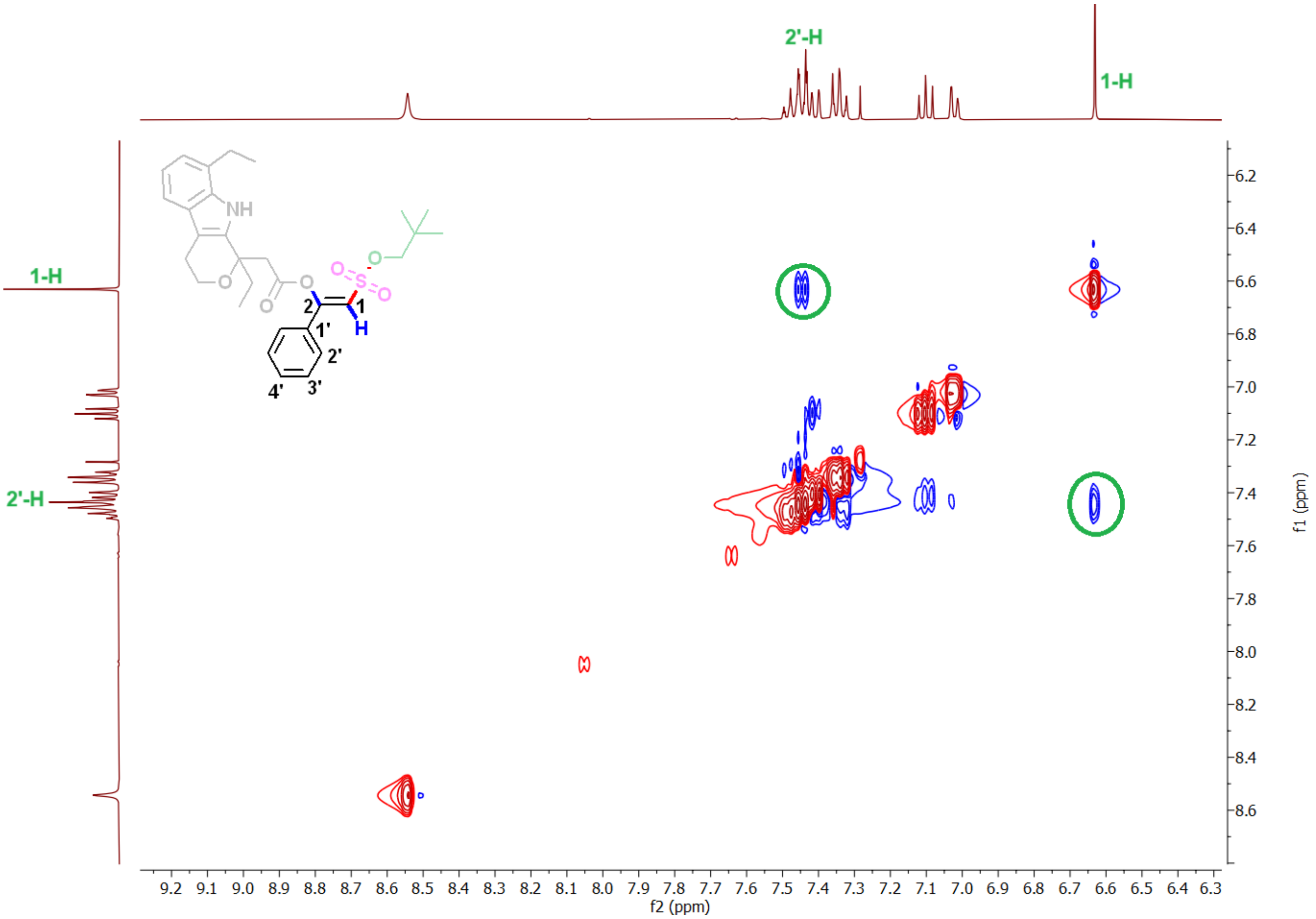

^{1}H NMR (300 MHz, $CDCl_3$) of **6t** (see procedure)

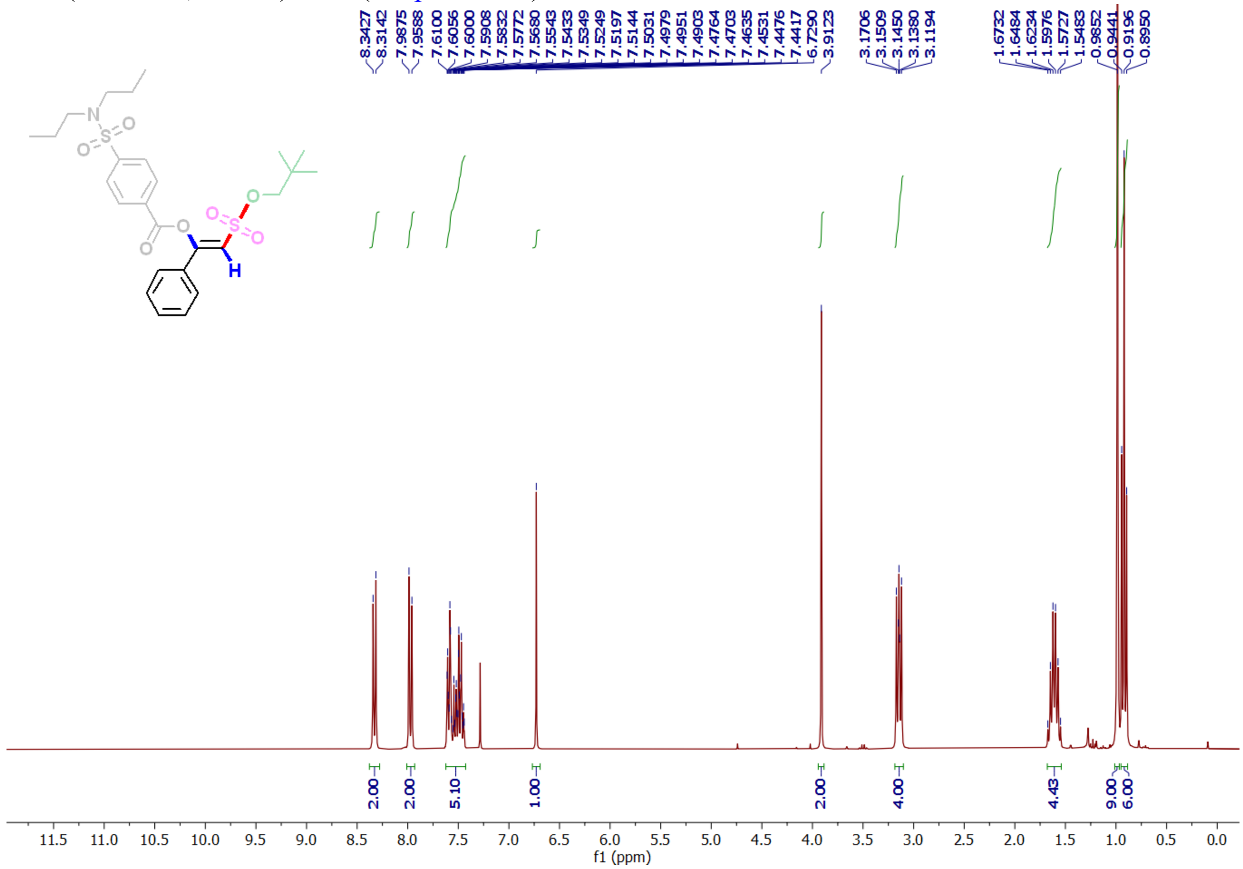

^{13}C NMR (75 MHz, $CDCl_3$) of **6t**

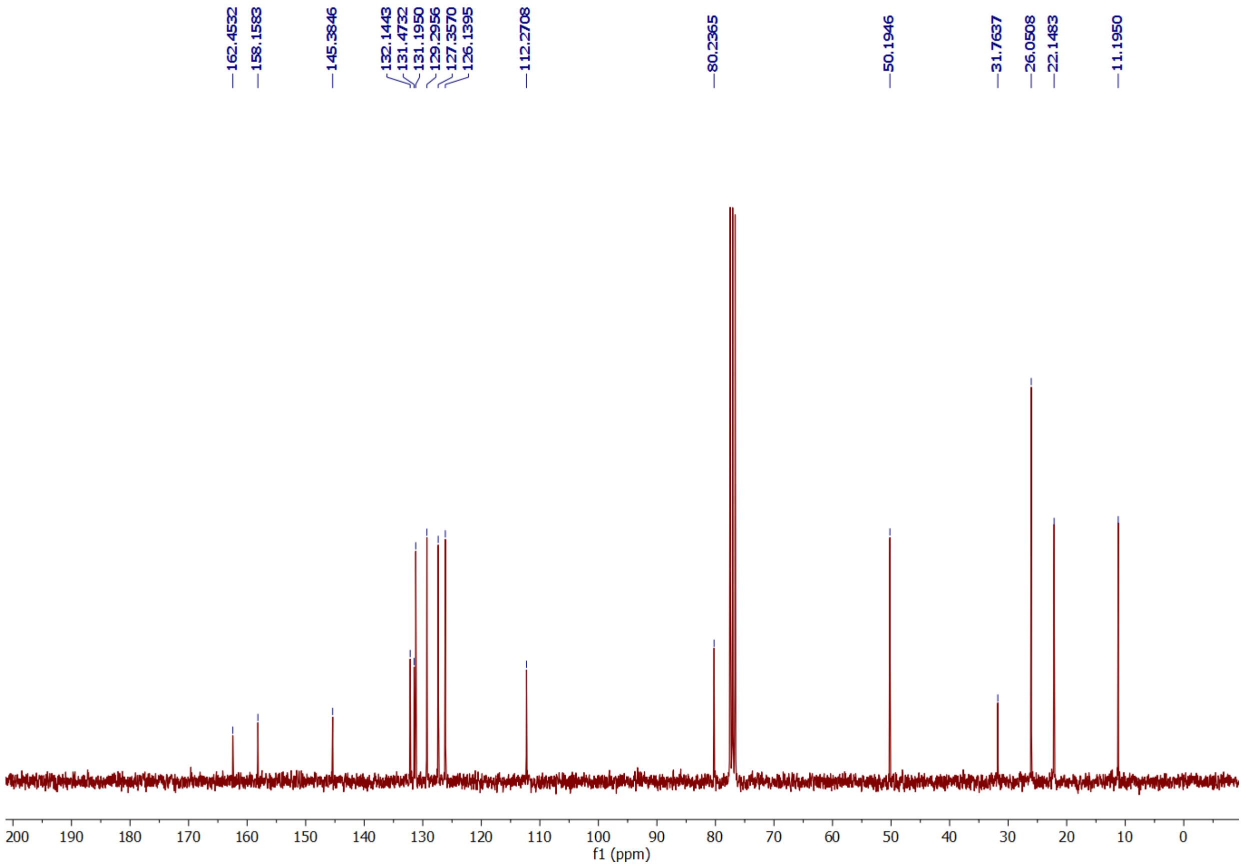

NOE Spectrum for **6t**

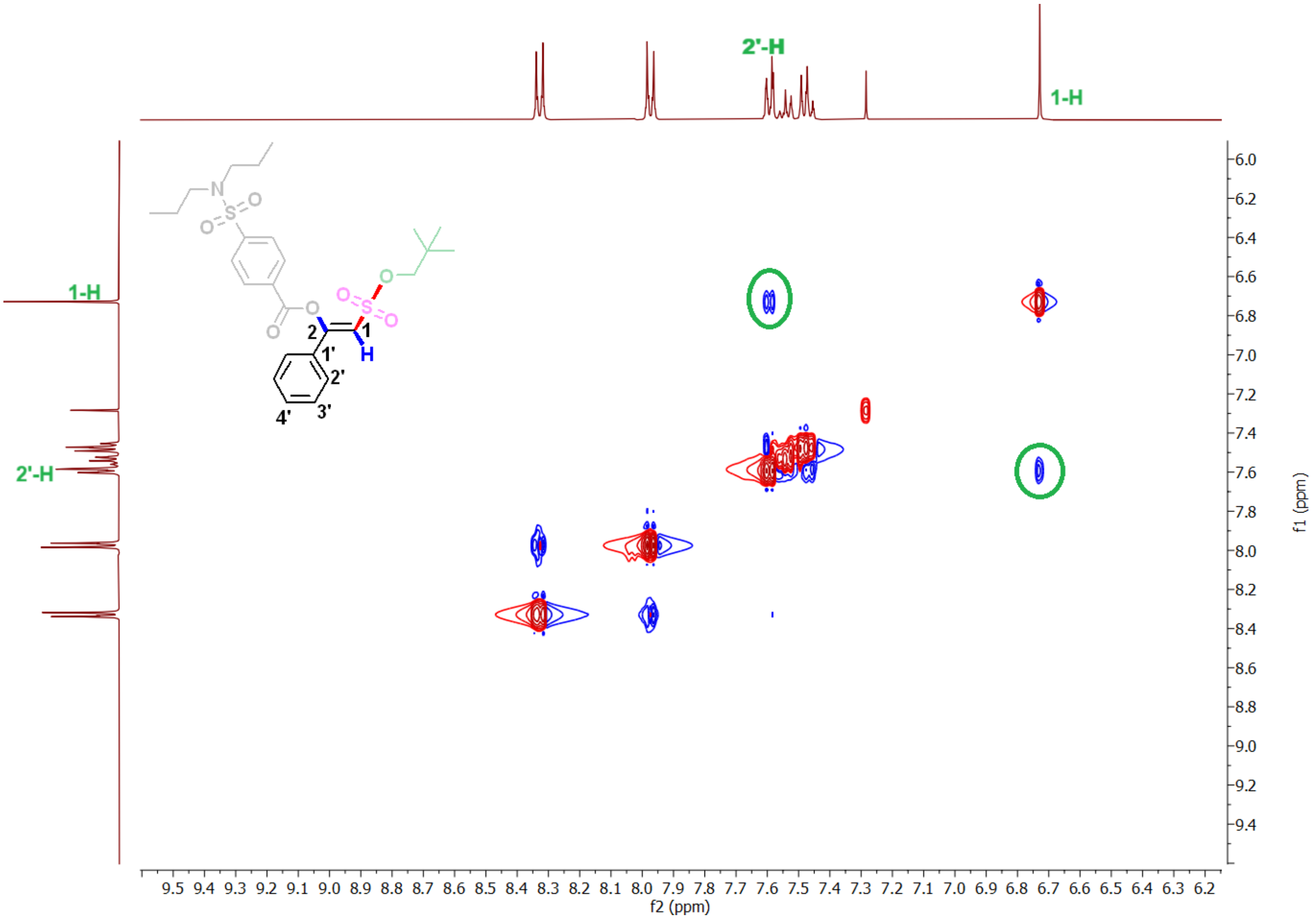

^{1}H NMR (300 MHz, $CDCl_3$) of **6u** (see procedure)

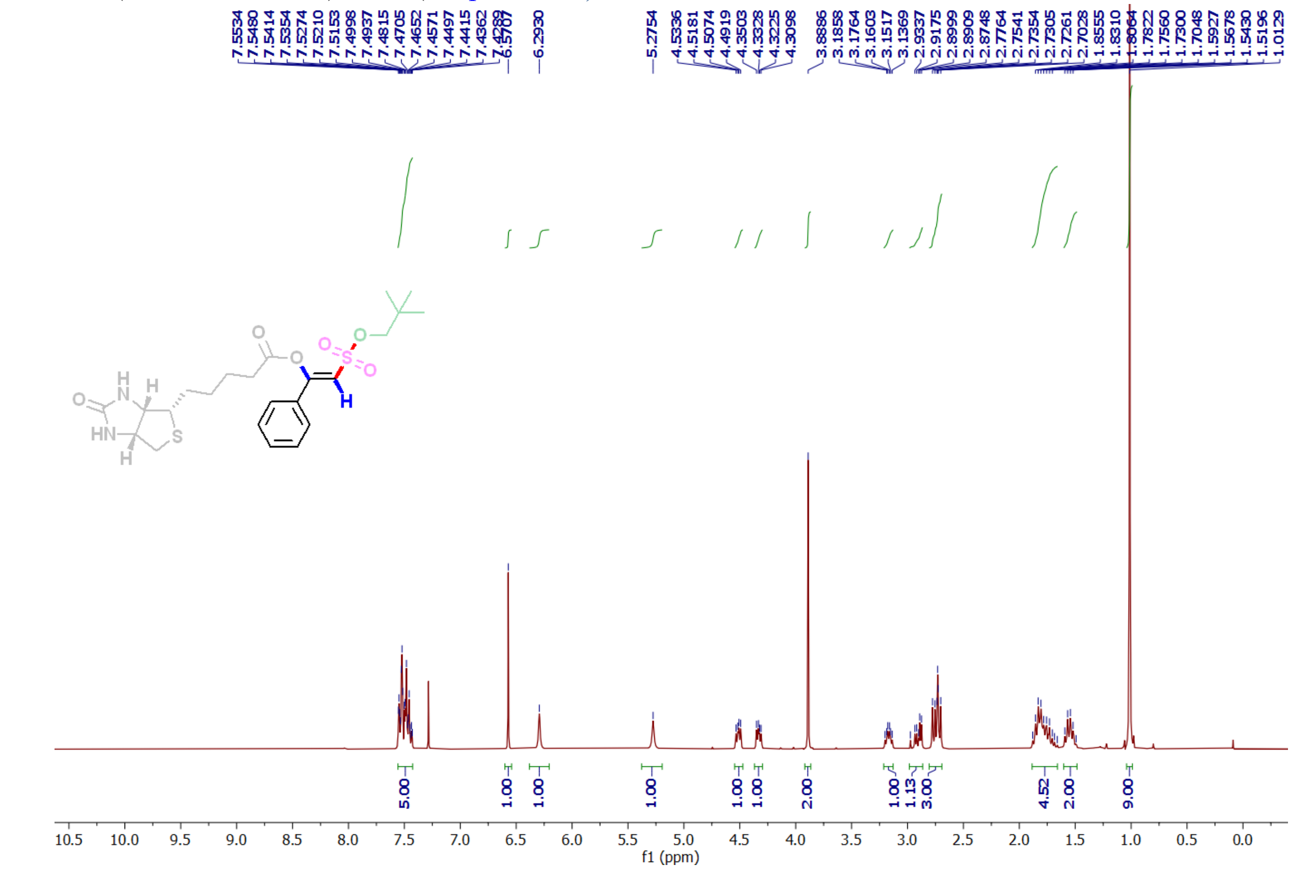

^{13}C NMR (75MHz, $CDCl_3$) of **6u**

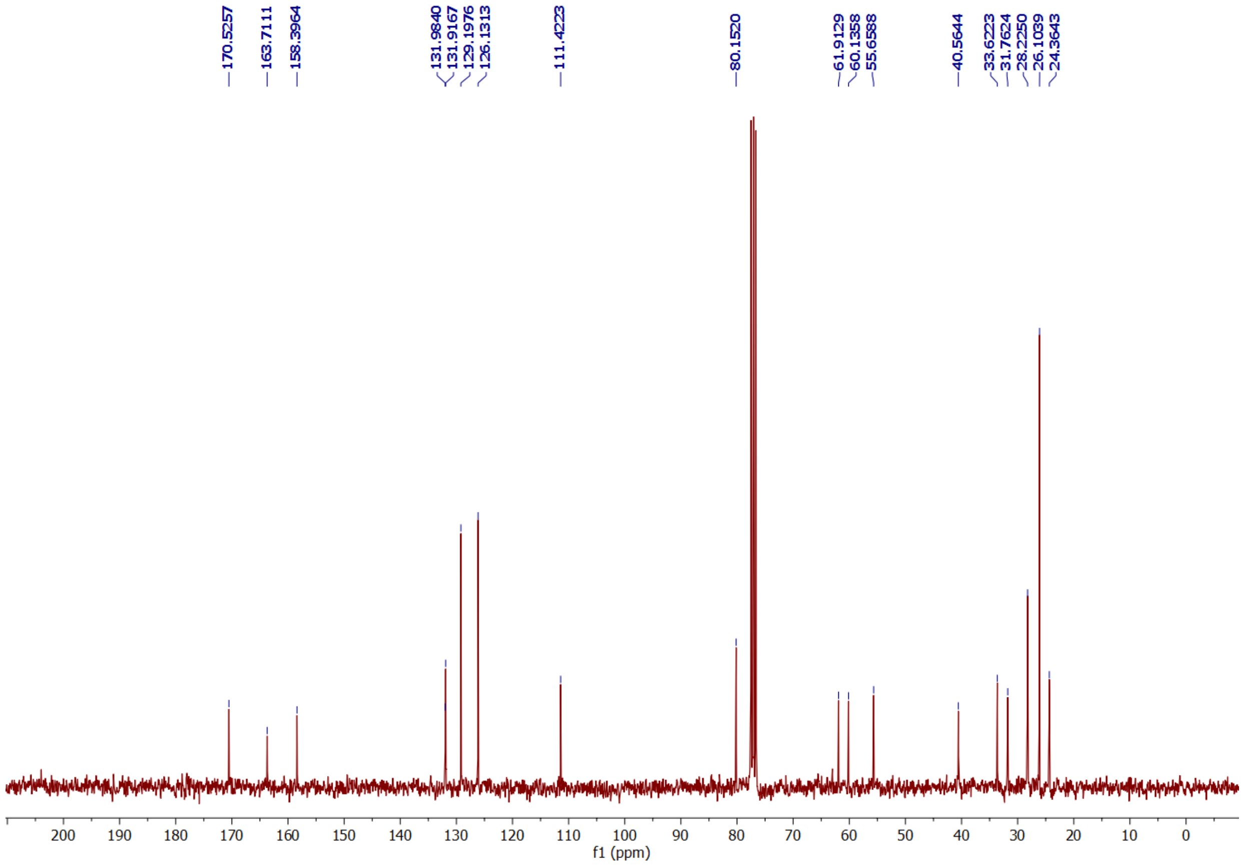

NOE Spectrum for **6u**

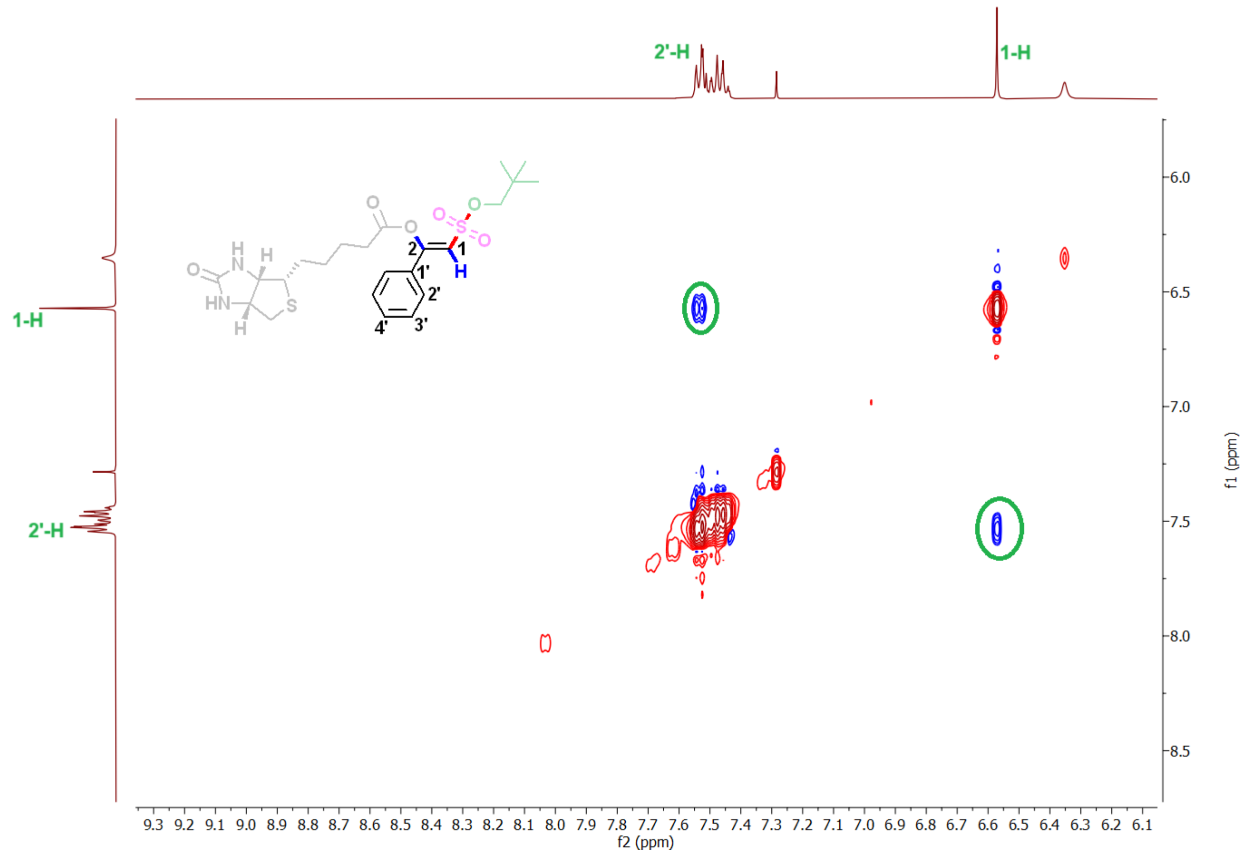

^{1}H NMR (300 MHz, $CDCl_3$) of **6v** (see procedure)

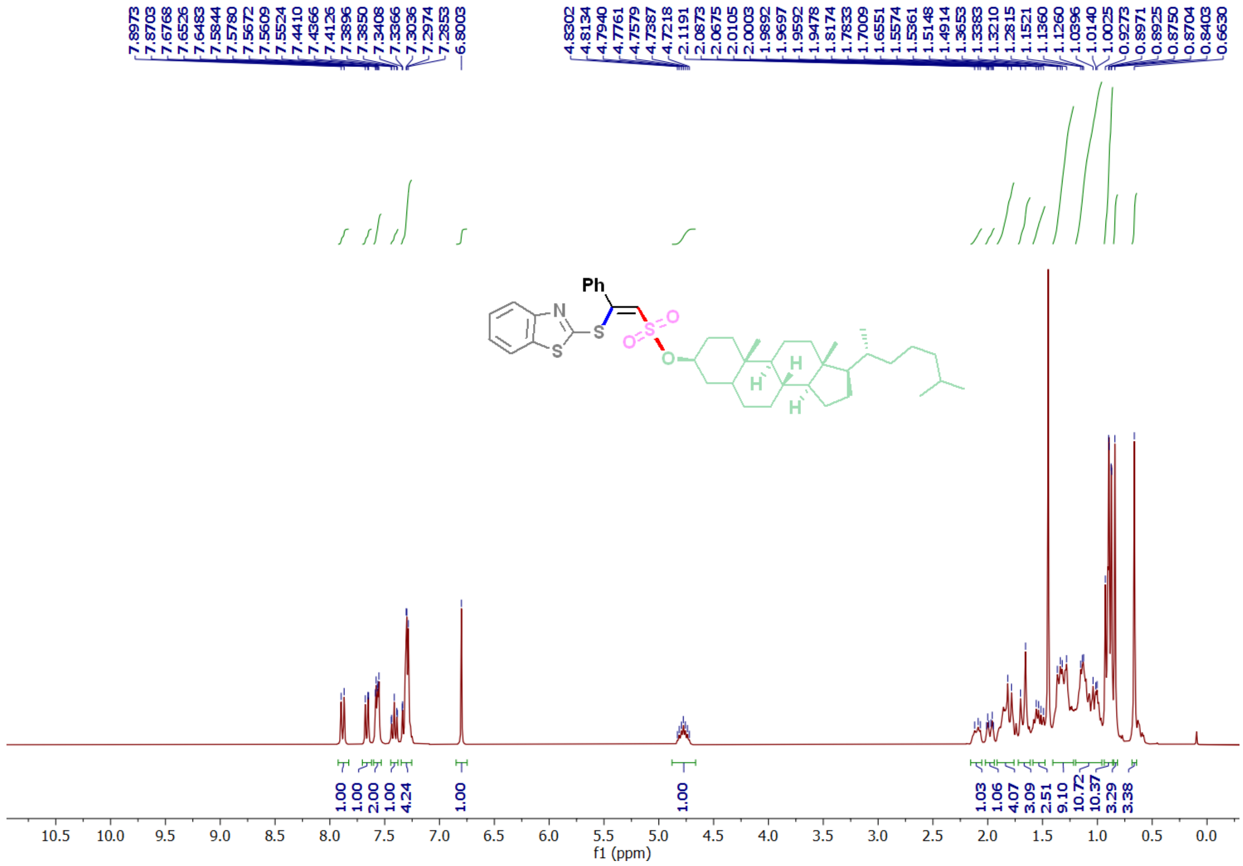

^{13}C NMR (101MHz, $CDCl_3$) of **6v**

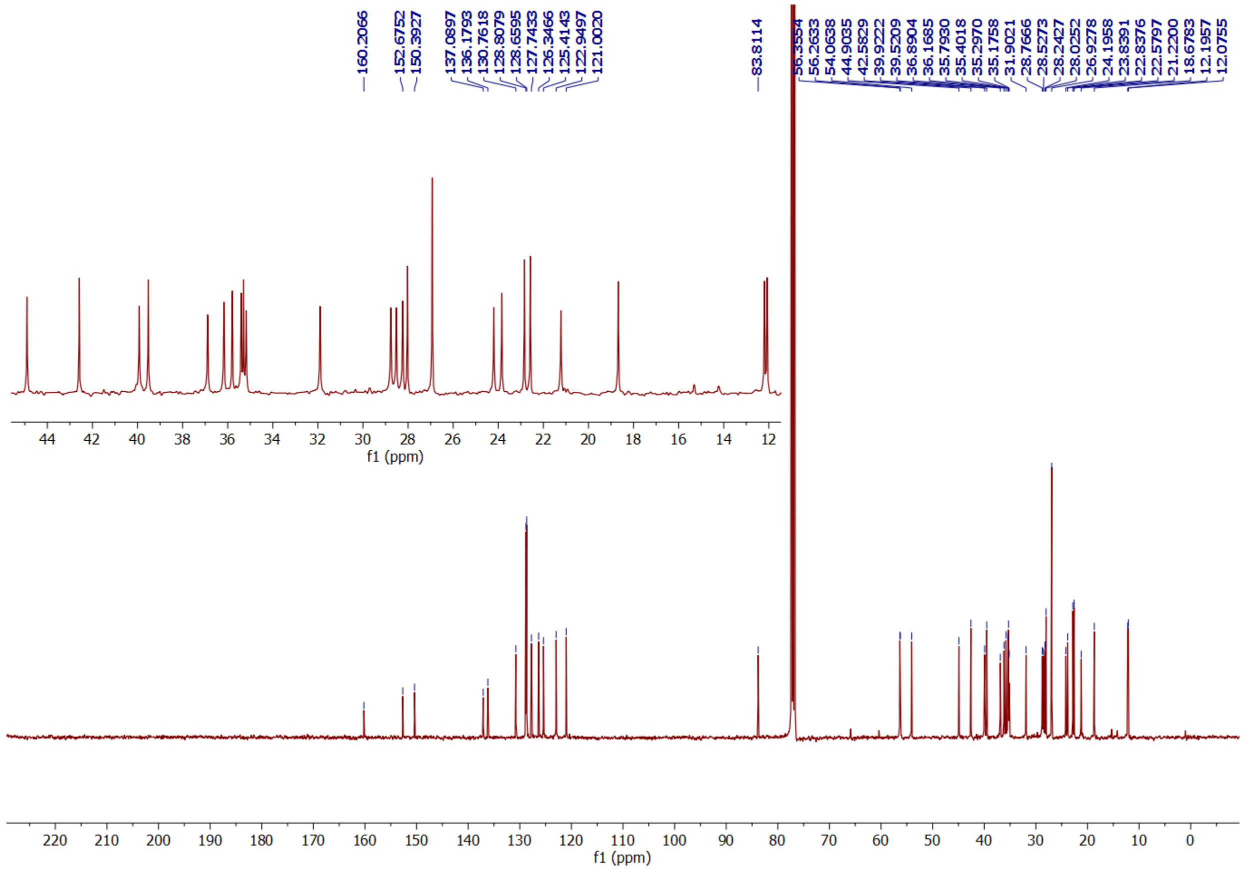

NOE Spectrum for **6v**

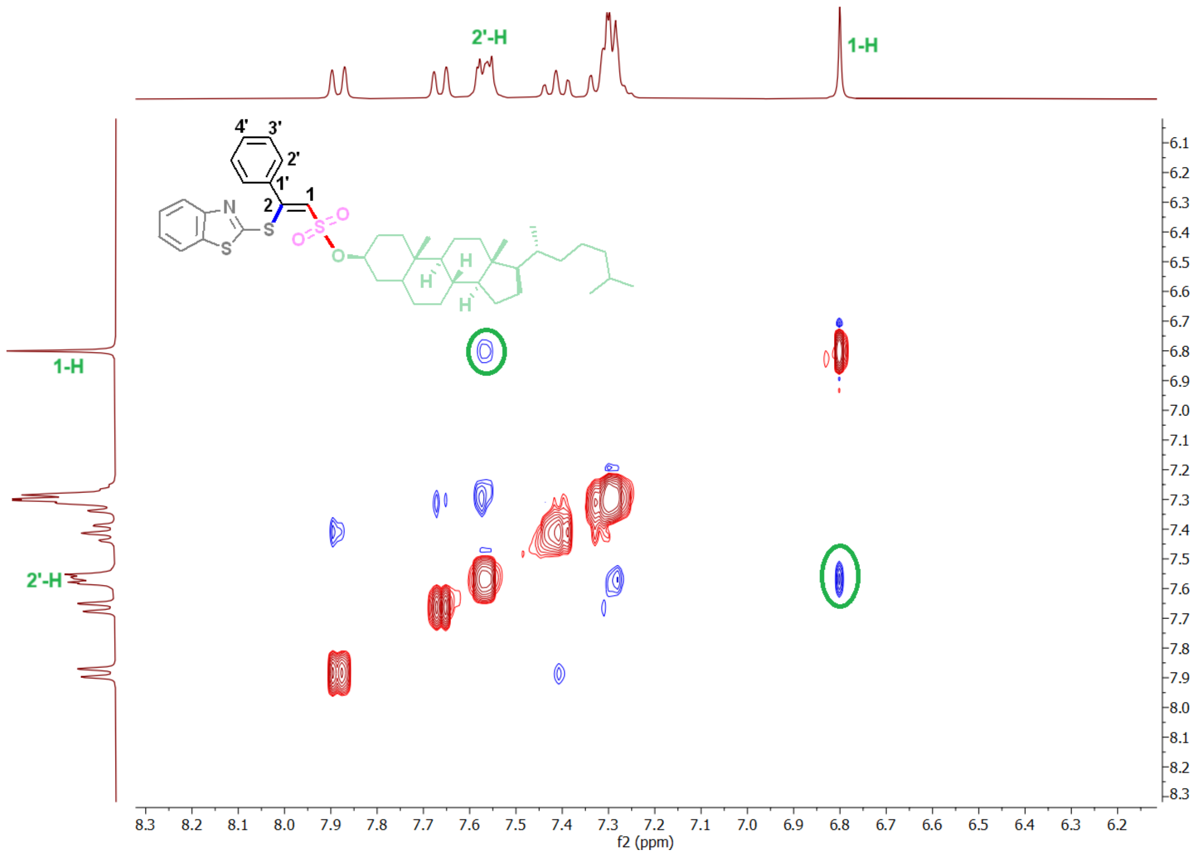

^{1}H NMR (300 MHz, $CDCl_3$) of **6w** (see procedure)

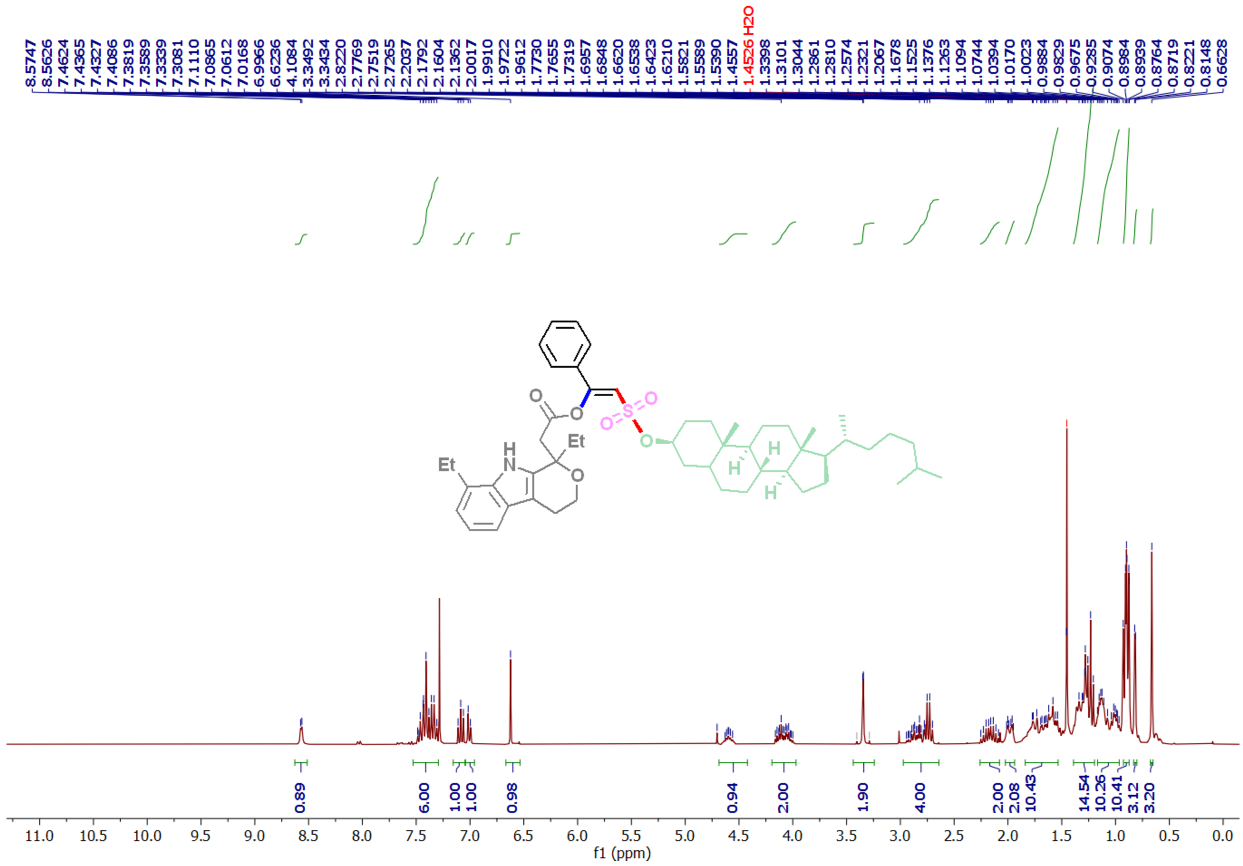

^{13}C NMR (101MHz, $CDCl_3$) of **6w**

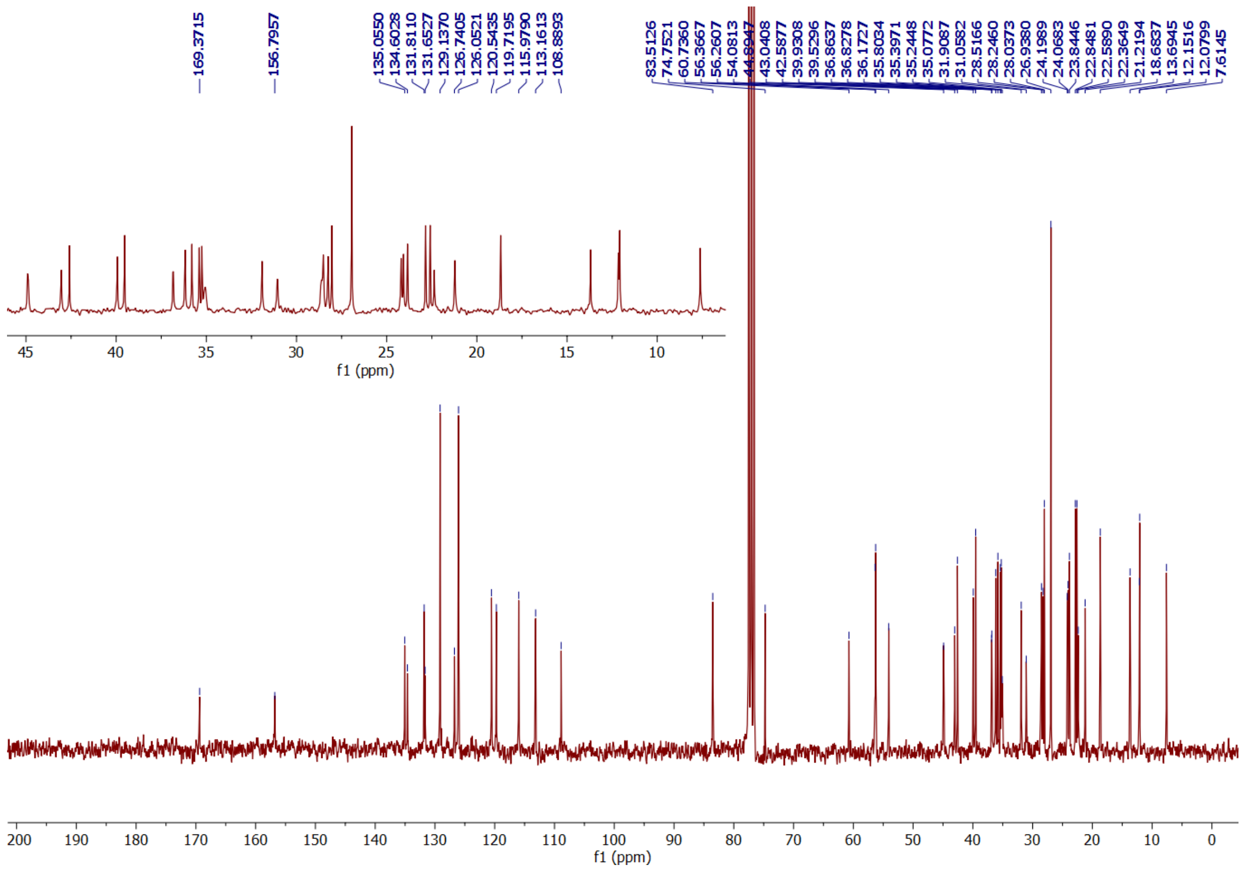

NOE Spectrum for **6w**

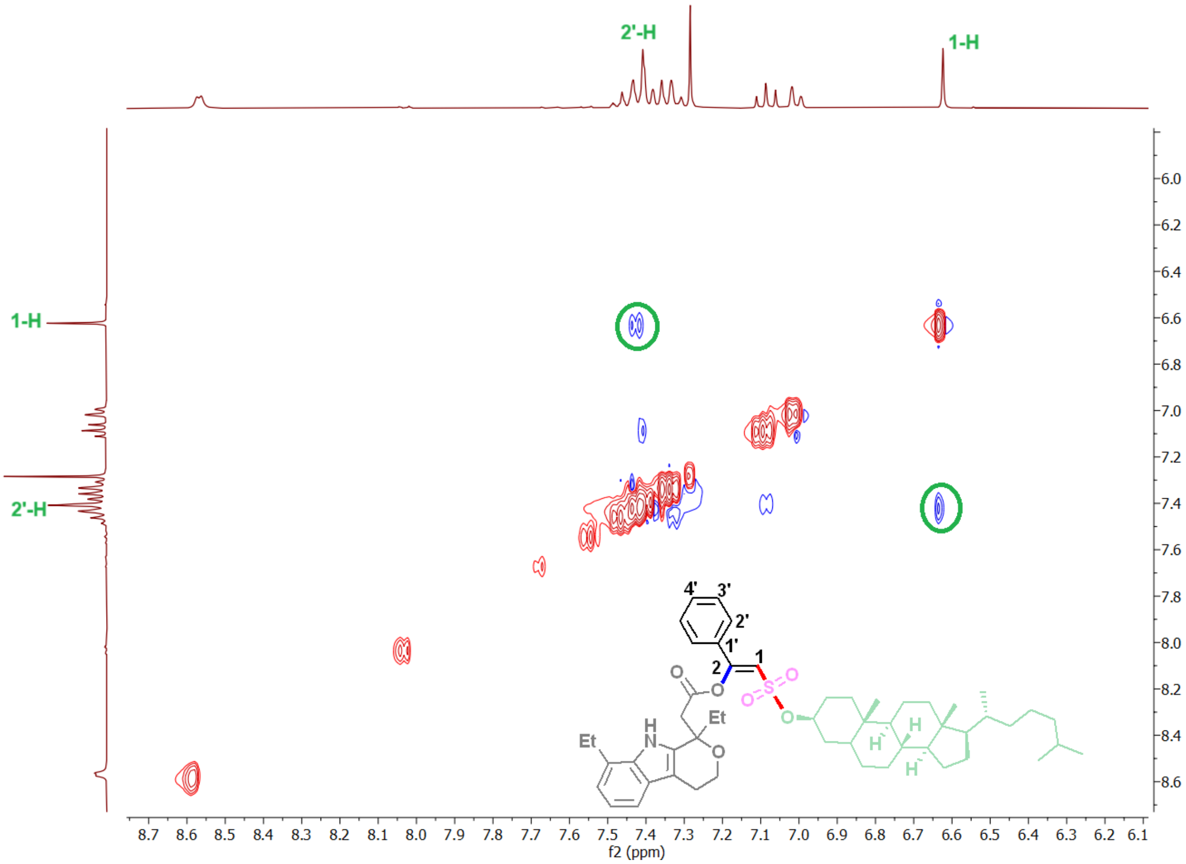

^{1}H NMR (300 MHz, $CDCl_3$) of **2r-step1** (see procedure)

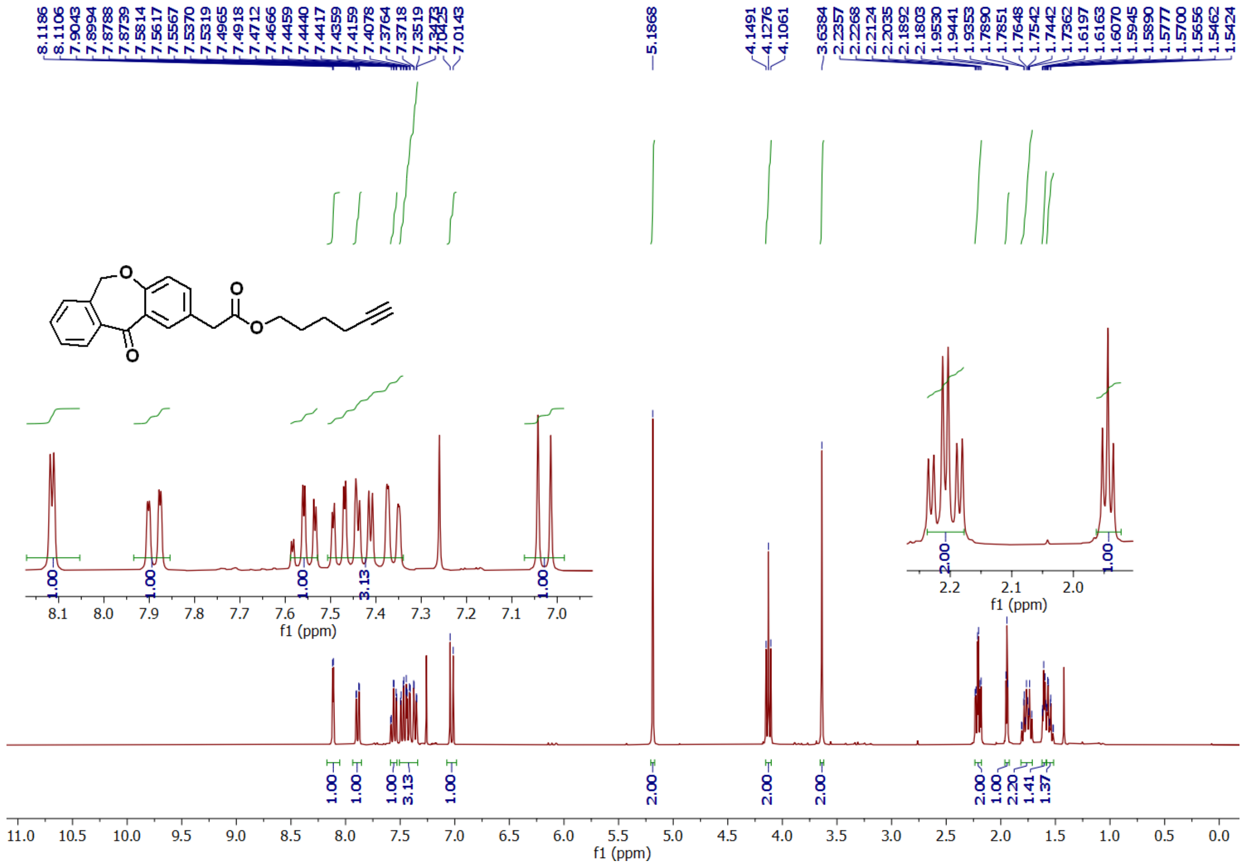

^{13}C NMR (75 MHz, $CDCl_3$) of **2r-step1**

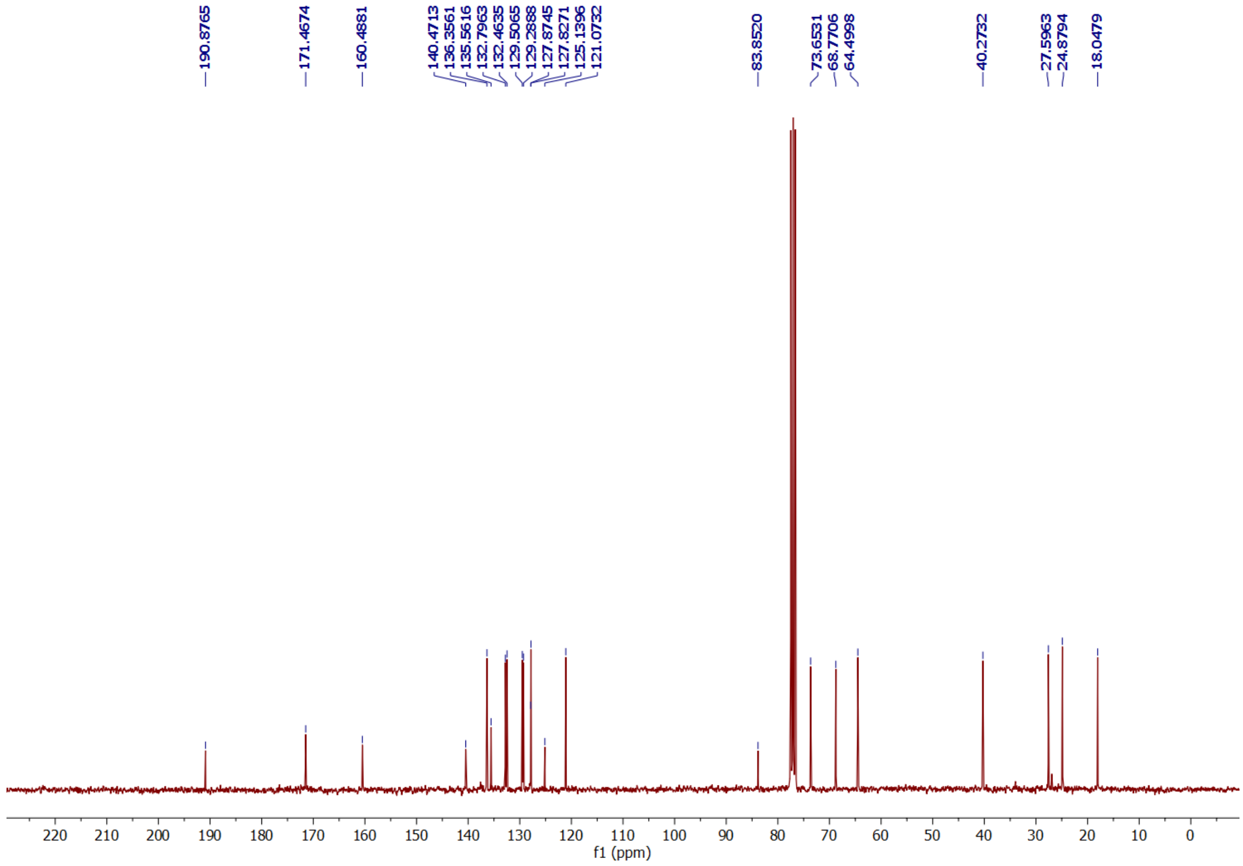

^{1}H NMR (400 MHz, $CDCl_3$) of **2r** (see procedure)

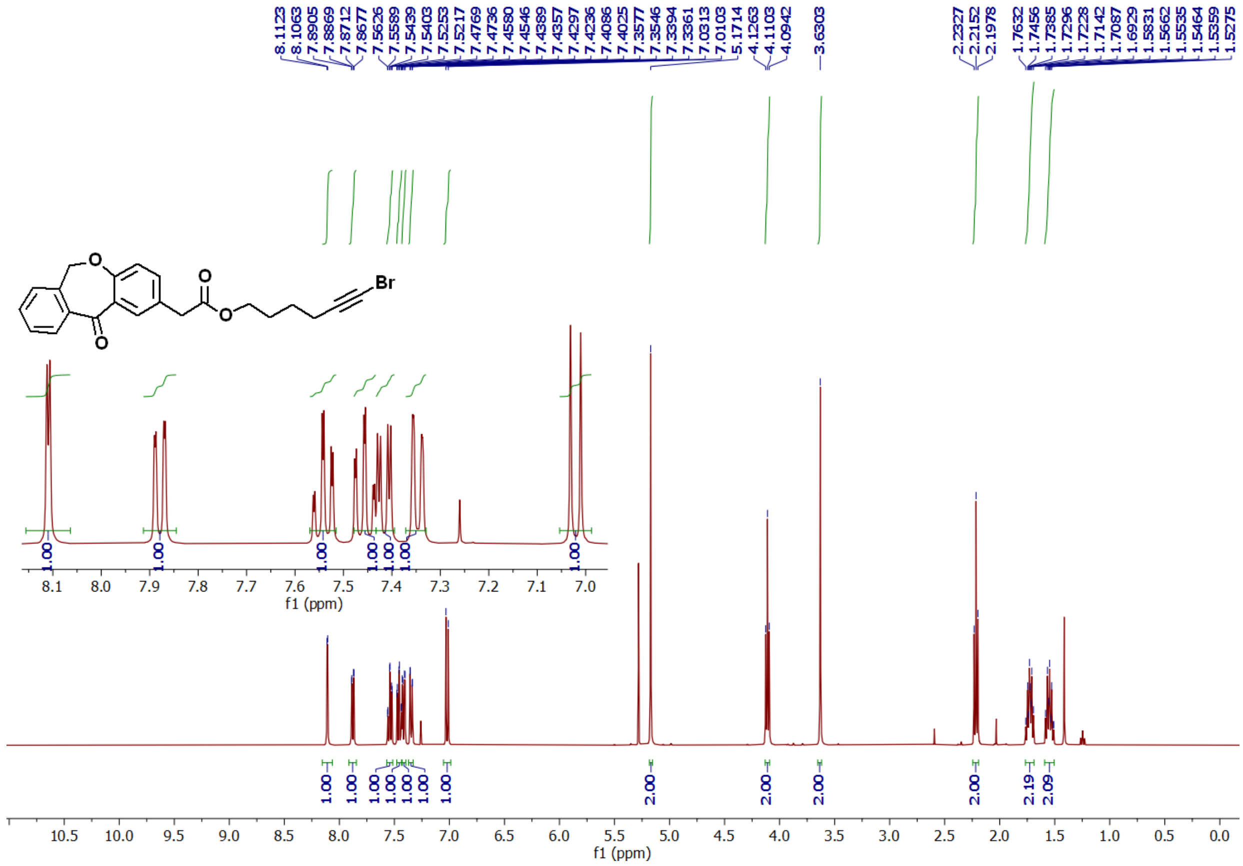

^{13}C NMR (101 MHz, $CDCl_3$) of **2r**

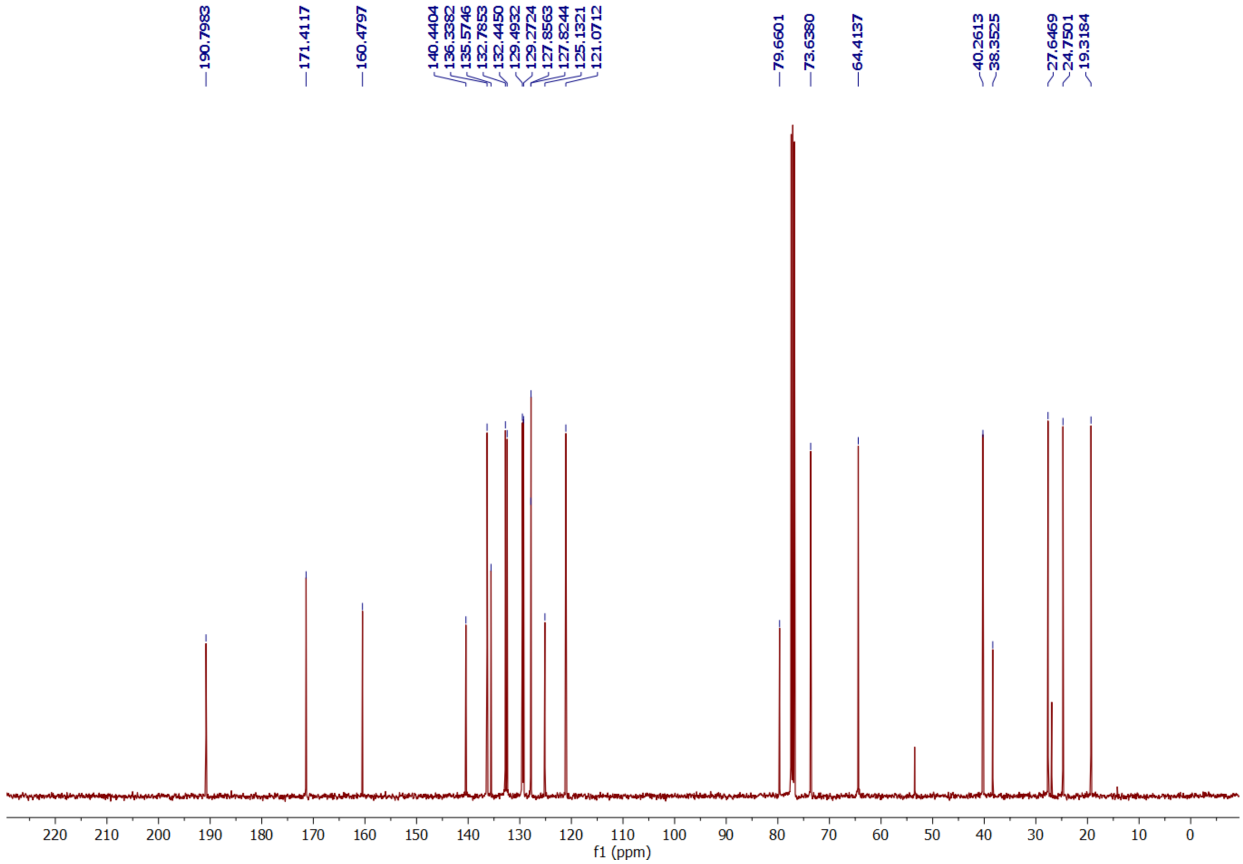

^{1}H NMR (300 MHz, $CDCl_3$) of **2s-step1** (see procedure)

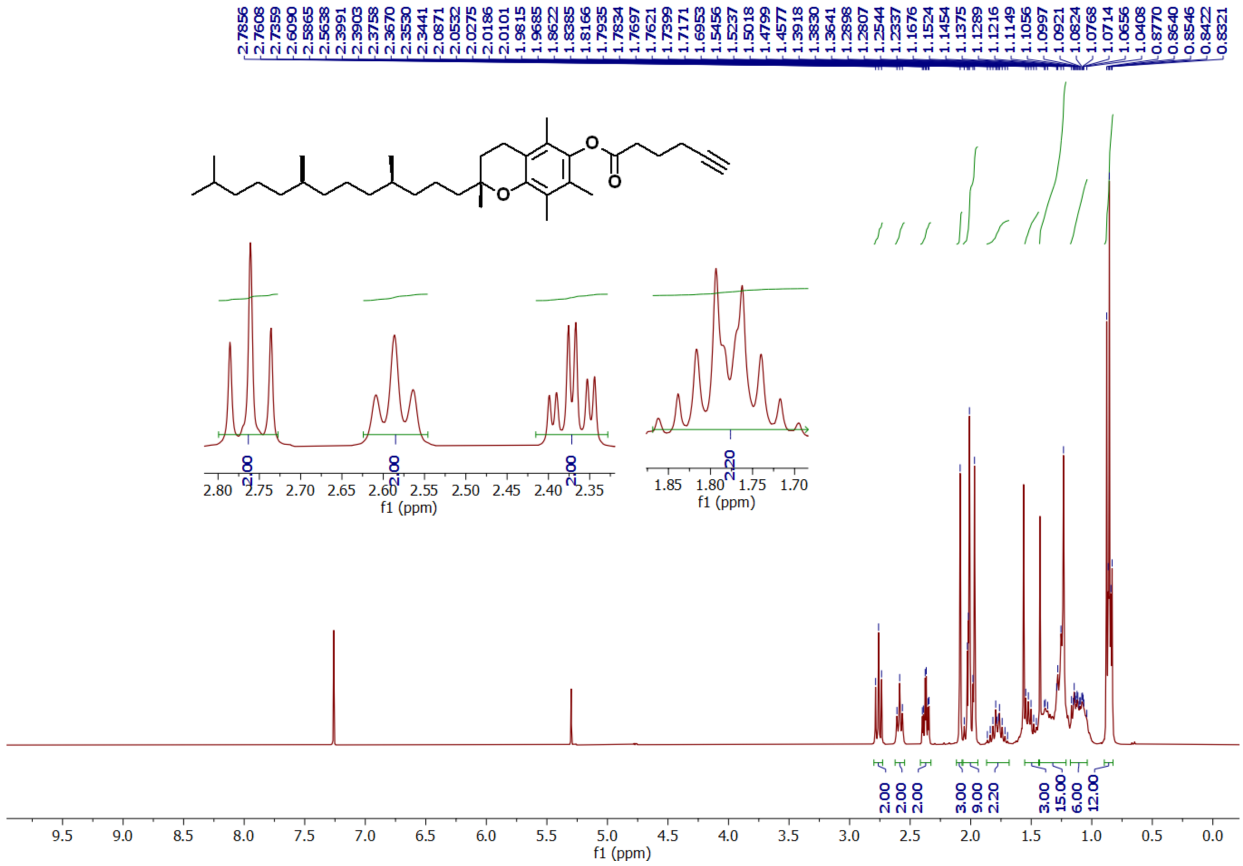

^{13}C NMR (75 MHz, $CDCl_3$) of **2s-step1**

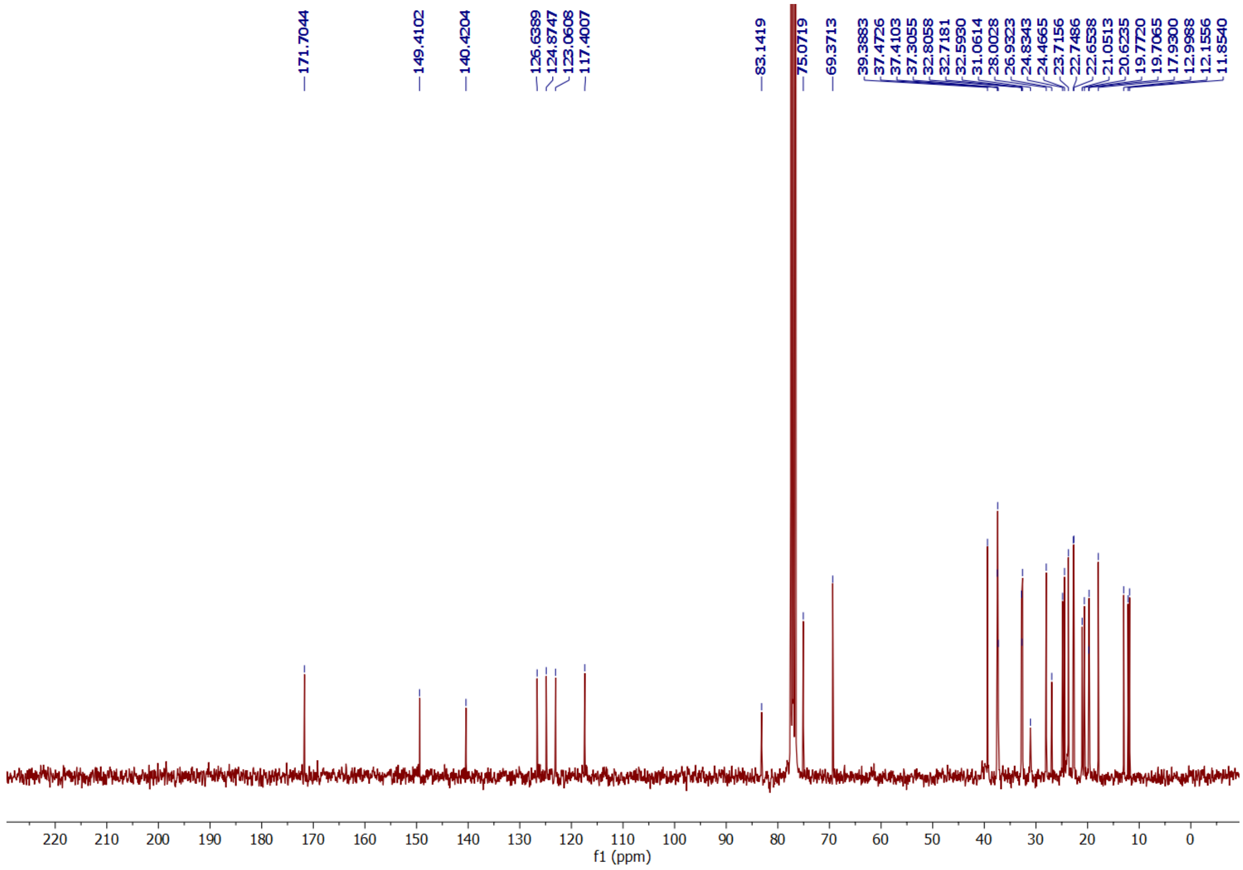

^{1}H NMR (300 MHz, $CDCl_3$) of **2s** (see procedure)

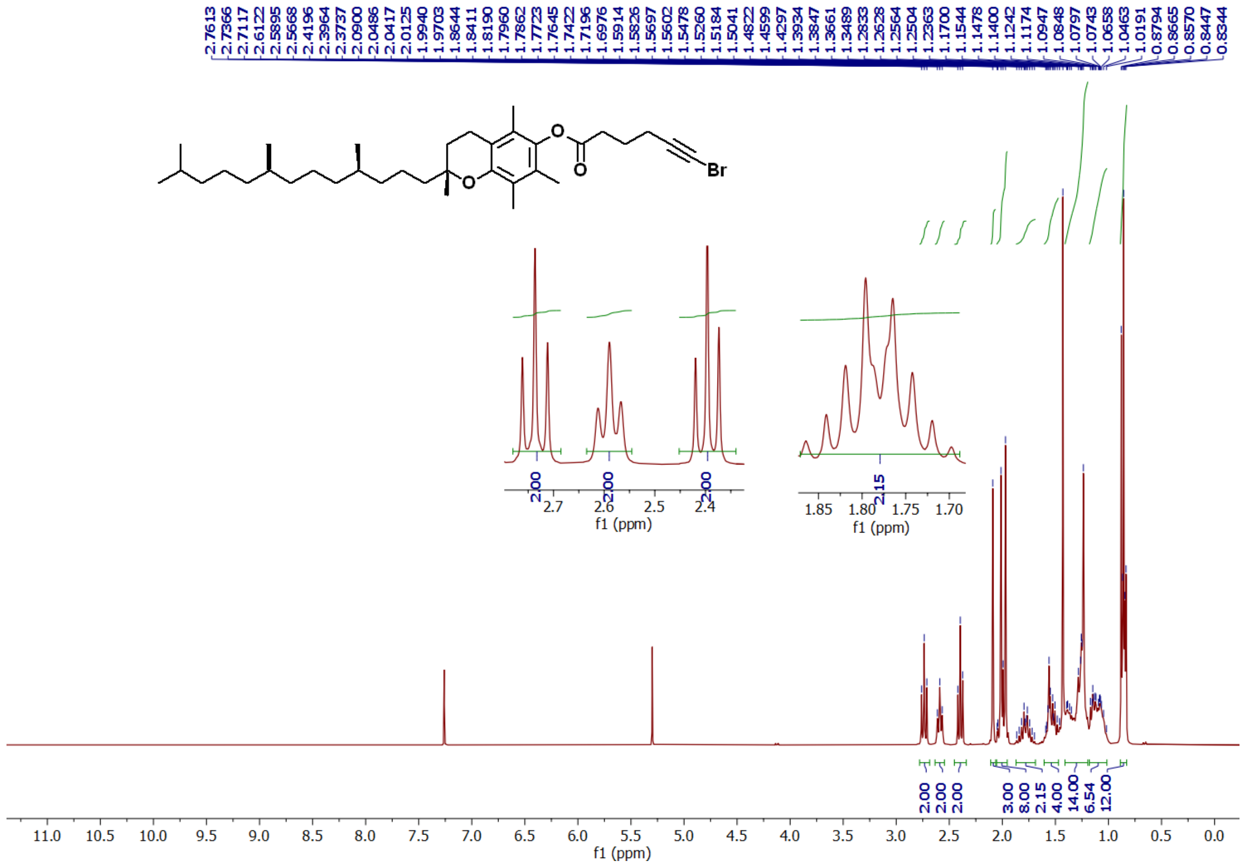

^{13}C NMR (75 MHz, $CDCl_3$) of **2s**

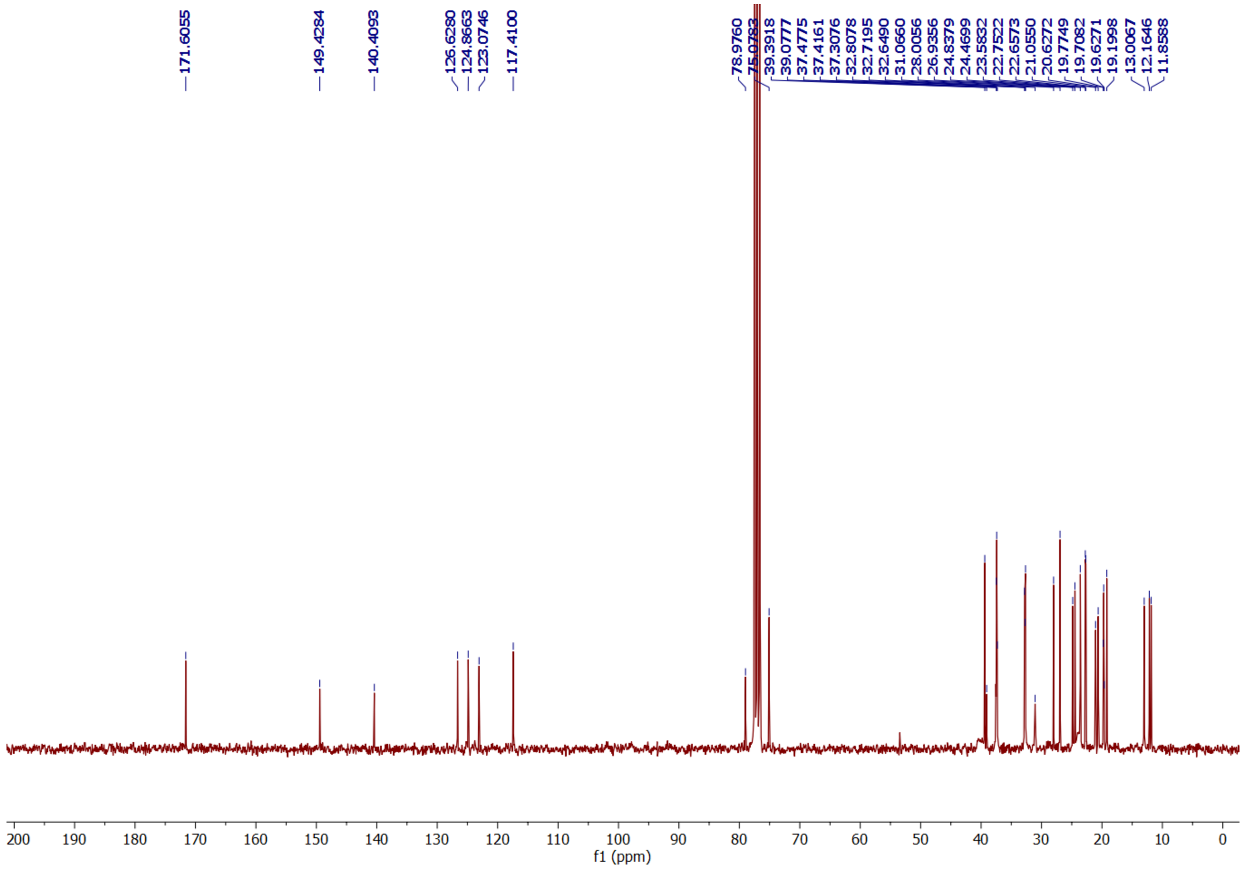

References

[1] J. B. Ferguson, *J. Am. Chem. Soc*. **1917**, 39, 364–373.

[2] P.-C. Chien, F. A. Breitschaft, H. Kelm, S. R. Waldvogel, G. Manolikakes, *ChemSusChem*, **2025**, e202500186.

[3] A. Maity, A. K. Sahoo, *J. Org. Chem*. **2024**, 89,852–863.

[4] D. S. Müller, I. Marek, *Chem. Soc. Rev*. **2016**, 45,4552–4566.
